# Identification of hub genes involved in cisplatin resistance in head and neck cancer

**DOI:** 10.1186/s43141-023-00468-y

**Published:** 2023-01-30

**Authors:** Raushan Kumar Chaudhary, Pukar Khanal, Uday Venkat Mateti, C. S. Shastry, Jayarama Shetty

**Affiliations:** 1 Department of Pharmacy Practice, NGSM Institute of Pharmaceutical Sciences (NGSMIPS), Nitte (Deemed to be University), Deralakatte, Mangaluru, Karnataka 575018 India; 2 Department of Pharmacology, NGSM Institute of Pharmaceutical Sciences (NGSMIPS), Nitte (Deemed to be University), Deralakatte, Mangaluru, Karnataka 575018 India; 3 Department of Radiation Therapy and Oncology, K.S. Hegde Medical Academy (KSHEMA), Justice K.S. Hegde Charitable Hospital, Nitte (Deemed to be University), Deralakatte, Mangaluru, Karnataka 575018 India

**Keywords:** Cisplatin-resistance, Drug–gene interaction, Gene expression, HNSCC, Hub genes, Overall survival, Protein–protein interactions

## Abstract

**Background:**

Cisplatin resistance is one of the major contributors to the poor survival rate among head and neck cancer (HNC) patients. Focusing on the protein–protein interaction rather than a single protein could provide a better understanding of drug resistance. Thus, this study aimed to identify hub genes in a complex network of cisplatin resistance associated genes in HNC chemotherapy via a series of bioinformatic tools.

**Methods:**

The genes involved in cisplatin resistance were retrieved from the NCBI gene database using “head and neck cancer” and “cisplatin resistance” as key words. The human genes retrieved were analyzed for their interactions and enriched using the STRING database. The interaction between KEGG pathways and genes was visualized in Cytoscape 3.7.2. Further, the hub gene was identified using the Cytohubba plugin of Cytoscape and validated using UALCAN and Human Protein Atlas database. Validated genes were investigated for the drug–gene interaction using the DGIbd database.

**Results:**

Out of 137 genes obtained using key words, 133 were associated with cisplatin resistance in the human species. A total of 150 KEGG pathways, 82 cellular components, 123 molecular functions, and 1752 biological processes were modulated on enrichment analysis. Out of 37 hub genes, CCND1, AXL, CDKN2A, TERT, and EXH2 genes were found to have significant (*p* < 0.05) mRNA expression and effect on overall survival whereas protein expression was found to be positive for all the significant genes except TERT. Thus, they can be targeted with palbociclib, methotrexate, bortezomib and fluorouracil, sorafenib, dasatinib, carboplatin, paclitaxel, gemcitabine, imatinib, doxorubicin, and vorinostat.

**Conclusion:**

As the pathogenesis of head and neck cancer is complex, targeting hub genes and associated pathways involved in cisplatin resistance could bring a milestone change in the drug discovery and management of drug resistance which might uplift overall survival among HNC patients.

**Supplementary Information:**

The online version contains supplementary material available at 10.1186/s43141-023-00468-y.

## Background

Head and neck cancer (HNC) is the sixth most prevalent cancer globally with 890,000 new cases and 450,000 deaths and includes the malignancy of the head and neck region of the body such as the oral cavity, oropharynx, nasopharynx, hypopharynx, pharynx, and larynx [1, 2]. HNC constitutes about 30–40% of the total cancer in India. According to the GLOBOCAN 2018 India, lip and oral cavity cancer were reported to be the second most highly incident (10.4%) with the third highest 5-year prevalence rate (19.59%) followed by laryngeal cancer (2.5% with 48%), hypopharynx (2.2% with 2.52%), oropharynx (1.5% with 3.18%), salivary gland (0.66% with 1.14%), and nasopharynx (0.44% with 0.93%) [3]. The main etiological factors behind the occurrence of HNC include the use of tobacco (smoked or smokeless) and alcohol consumption. In addition, other risk factors are human papillomavirus (HPV) infections, Epstein bar virus (EBV), poor oral hygiene, alteration in oncogenes (PIK3CA, RAS), and tumor suppressor genes (TP53, CDKN2A, FAT1, NOTCH1, KMT2D, NSD1, and TGFBR2) [2]. The approach to manage the HNC includes surgery, radiotherapy, chemotherapy, and immunotherapy. Chemotherapy serves as the backbone of cancer management either alone or along with radiation therapy among locally advanced tumor [4]. Cisplatin is one of the most widely used chemotherapeutic agents to manage HNC, possess an anticancer effect by forming deoxyribonucleic acid (DNA) adduct, and arrest cell cycles leading to cell death [5, 6].

Cisplatin is often administered in the locally advanced tumor (stages III and IV) as a concurrent chemoradiation therapy (CRT) either alone/after surgery and as an induction therapy followed by CRT. In cisplatin-based CRT, cisplatin is administered at a dose of 100 mg/m^2^ IV on days 1, 22, and 43 or cisplatin 30–40 mg/m^2^ IV weekly for 6 to 7 weeks. Out of these, both cisplatin-based protocols, cisplatin 100 mg/m^2^ has been reported to be significantly effective (*p* = 0.014) in a 2-year locoregional control (73.1% > 58.5%) but associated with several toxicities (hyponatremia, leukopenia, neutropenia, and lymphocytopenia) as compared to weekly doses. Thus, weekly dosing is most frequently popular among physicians [4, 7]. Similarly, EORTC 22,931 and RTOG 9501 trial has reported improved locoregional control (LRC): 69 vs. 82%, progression-free survival (PFS); 36 vs 47%, overall survival (OS); 40 vs 53% and 5-year locoregional control (LRC); 68 vs 81%, disease-free survival (DFS): 25 vs 35%, but without significant OS: 37 vs 45%, respectively [8]. Besides this, cisplatin is also combined at wide range of doses with other anti-cancerous agents like paclitaxel, docetaxel, 5-fluorouracil (5-FU) (TPF regimen), hydroxyurea, etoposide, pembrolizumab, nivolumab, and cetuximab, etc., to manage the locally advanced stage, recurrent, or metastatic stage of head and neck cancer [7]. Despite all this treatment modality, only around 40% of locally advanced HNC patients respond to therapy [9], about 65% of HNC patients present with recurrence or metastasis (R/M HNSCC) stage, and about 70–90% of R/M HNSCC patients do not show response to immune checkpoint inhibitors (ICI) [4]. However, a combination of pembrolizumab alone or in combination with platinum (cisplatin or carboplatin) and 5-FU is the first line of therapy for the R/M HNSCC. Further, nivolumab can be used in disease progression on or after platinum therapy. Similarly, other combination preferred for the R/M HNC  is the combination of cetuximab/platinum (cisplatin or carboplatin)/5-FU (EXTREME trial) [7].

Despite the progress to manage HNC, a 5-year survival rate among HNC patients remains to be 50%. Thus, it is often combined with other anti-cancerous agents [10, 11]. According to Surveillance, Epidemiology, and End Results (SEER) registry, the 5-year survival has increased from 55% (1992–1996) to 66% (2002–2006) [2]. Further, the death rate among Indian patients is about 28% of the world and 71% of South East Asia due to HNC [12] which might be attributed to the high exposure to etiological agents, high incidence and prevalence, lack of treatment facilities and resources, presentation of disease at an advanced stage at diagnosis (66.6%), and poor response to therapy at a locally advanced stage (65%) [2, 3, 13].

Cisplatin resistance occurs by intrinsic or extrinsic pathways. Primarily, deregulated drug transport (influx/efflux transport), increased DNA repair, enzymatic detoxification of drug, default in autophagy, and apoptosis are responsible for cisplatin resistance. Also, damaged DNAs are repaired through various pathways such as nucleotide excision repair (NER), homologous recombination (HR), mismatch repair (MMR), and non-homologous end joining (NHEJ) out of which NER is chiefly responsible to clear cisplatin–DNA adducts as well as increased DNA repair are contributing reason to the cisplatin resistance leading to poor outcome among patients [5, 6, 14].

Proteins are the essential biological macromolecules that are involved in all the various cellular processes. Knowledge and understanding of the protein expression and its interactions give insight into the complex molecular pathways involved in drug resistance. The protein–protein interaction (PPI) is the indirect phenomenon that is responsible for the various cellular functions [15]. The transient PPI is generally responsible for signaling pathways whereas the permanent PPI forms a protein complex. It has been reported that around 80% of protein functions through the PPI rather than independently. Thus, rather than focusing on a single protein involved in drug resistance, assessing a protein complex or a network of proteins may help to combat drug resistance more effectively. Further, the PPI interactions help to predict the function of proteins that has been untraced previously. In addition, PPI may be used to trace proteins in drug resistance by establishing its role in cancer drug therapy [16]. Hence, the present study aimed to identify the complex network interactions of cisplatin-associated resistance in HNC chemotherapy with a series of bioinformatic approaches.

## Methods

### Retrieval of gene

The National Center for Biotechnology Information gene (NCBI gene) database (https://www.ncbi.nlm.nih.gov/gene) was queried to identify genes involved in cisplatin resistance among HNC [17]. The queried term included “head and neck cancer” and “cisplatin resistance” using “AND” as a Boolean operator. This search resulted in total of 137 items which consist of specific genes related to *Homo sapiens* (133), *Mus musculus* (3), and human papillomavirus type 16 (1). The genes involved in cisplatin resistance in *Homo sapiens* (133) were used for this study (Supplementary file 1). The search details for this study includes: (Head and Neck cancer [All Fields], (Cisplatin [All Fields], Resistance [All Fields])), “*Homo sapiens*”[porgn], and alive [prop].

### Gene ontology analysis

The retrieved human genes associated with cisplatin resistance among HNC patients were evaluated for the gene–gene interactions using the STRING database (Szklarczyk et al., 2019; https://string-db.org/) [18]. The pathways modulated by PPI were identified using the KEGG pathway. Further, the genes were enriched in the STRING to identify pathways modulated and 3 GO terms, i.e., cellular component, biological process, and molecular function. The commonly regulated genes among 3 gene ontology terms along with the KEGG pathways were visualized using venny 2.1 (Oliveros, 2007, 2015; https://bioinfogp.cnb.csic.es/tools/venny/) [19]. The network between protein and pathways was constructed in Cytoscape version 3.7.2 and analyzed by treating it as undirected and setting the node size “low values to small size” and “low values to bright colors” based on edge count for both settings.

### Identification and validation of hub genes

The top ten hub genes were identified via 12 different topological analysis methods of the Cytohubba plugin of Cytoscape 7.3.2. Further, the total hub genes identified through all the topological methods were investigated for their mRNA expression, the effect of the expression on overall survival, and protein level expression. The mRNA expression and survival analysis of hub genes involved in cisplatin resistance were evaluated using the UALCAN (http://ualcan.path.uab.edu/) [20], and the hub genes with statistically significant mRNA expression as well as overall survival were further investigated for protein level expression using the Human Protein Atlas (http://www.proteinatlas.org) [21].

### Drug–gene interaction for the hub genes

**Fig. 1:**
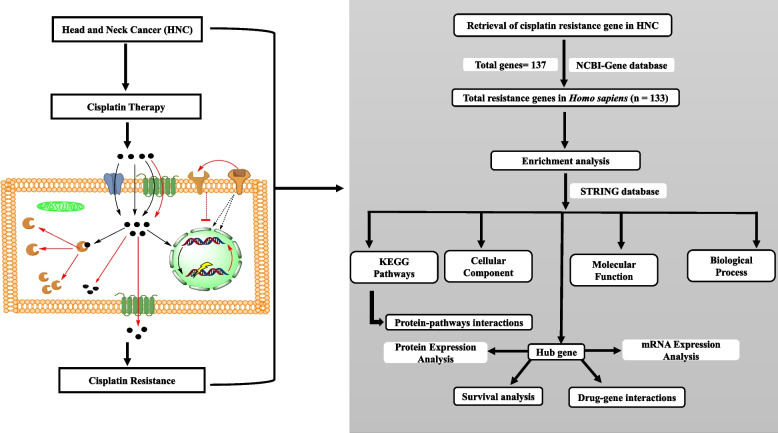
Workflow for the identification of hub genes involved in cisplatin resistance

Hub genes are considered as one of the important drug targets in drug discovery. The statistically significant hub genes were further explored for their interaction using the Drug–Gene Interaction database (v4.2.0—sha1 afd9f30b), (https://dgidb.genome.wustl.edu/). The drug-significant hub gene interaction was visualized using Cytoscape 3.7.2. The detailed methodological flow chart for the investigation of hub genes involved in cisplatin resistance is depicted in Fig. 1.

### Statistical analysis

The enrichment analysis of PPI was assessed via the whole genome statistical background. The expression in tumor/normal tissue and Kalpan-Meier curve for independent genes were considered to be significant if *p* < 0.05.

## Results

**Fig. 2:**
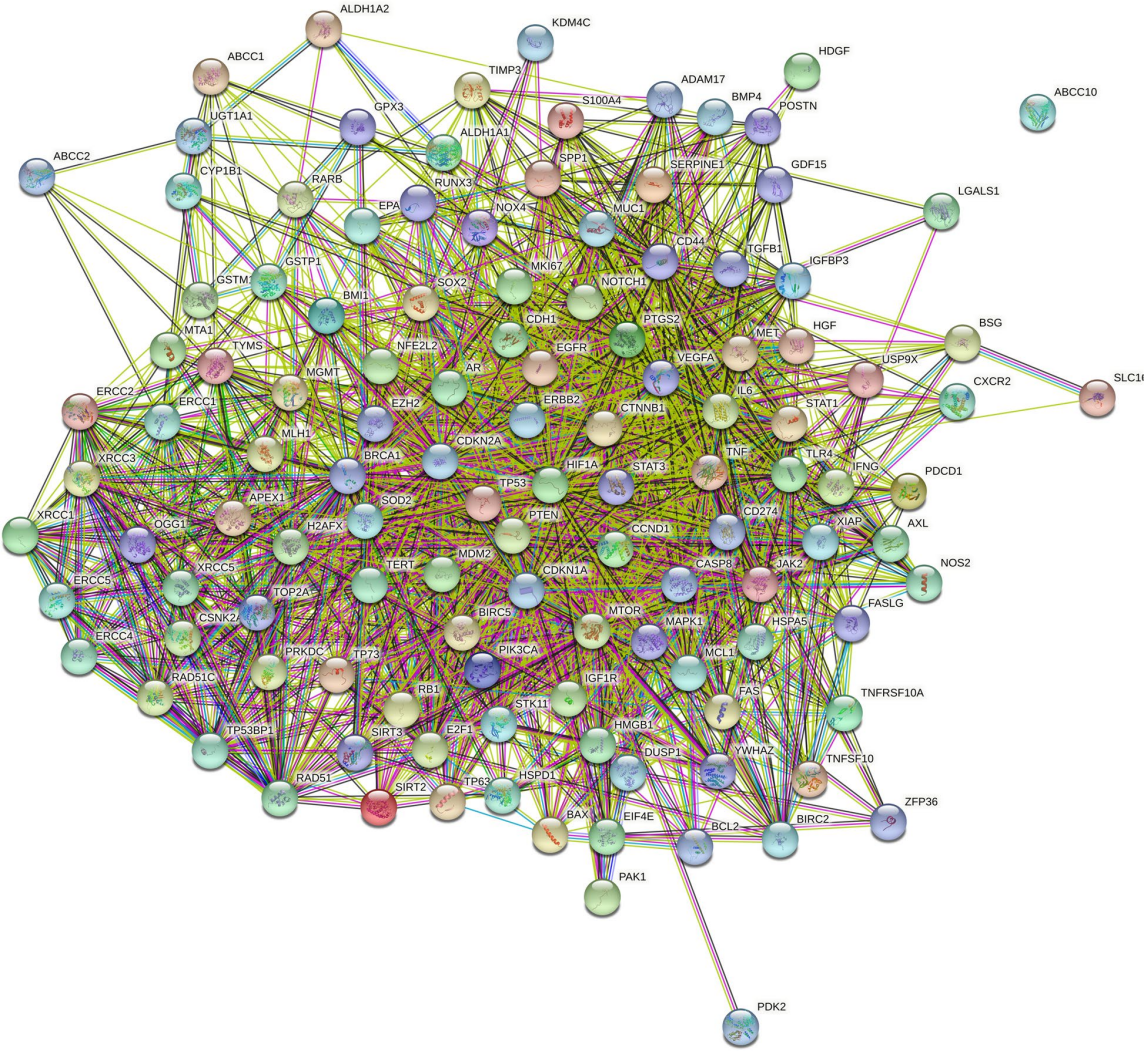
Protein–protein interaction of cisplatin-resistance associated genes

Out of 137 retrieved genes, 133 genes were associated with cisplatin resistance in HNC among the *Homo sapiens*. These 133 genes were evaluated for 3 gene ontology terms including KEGG pathways in the STRING: the protein–protein interactions for cisplatin-associated genes in HNC (Fig. 2) where nodes and edges in the network represent proteins and protein–protein association, respectively. These interactions were based on node color, i.e., colored nodes (query proteins and first shell of interactions), white node (second shell of interactions), and node content, i.e., empty node protein of unknown 3D structure), and filled node (some 3D structure is known). Further, it was also based on the known interactions (curated databases and experimentally determined), predicted interactions (gene neighborhood, gene fusions, and gene co-occurrence), and others (text mining, co-expression, and protein homology).

### KEGG pathways

**Fig. 3:**
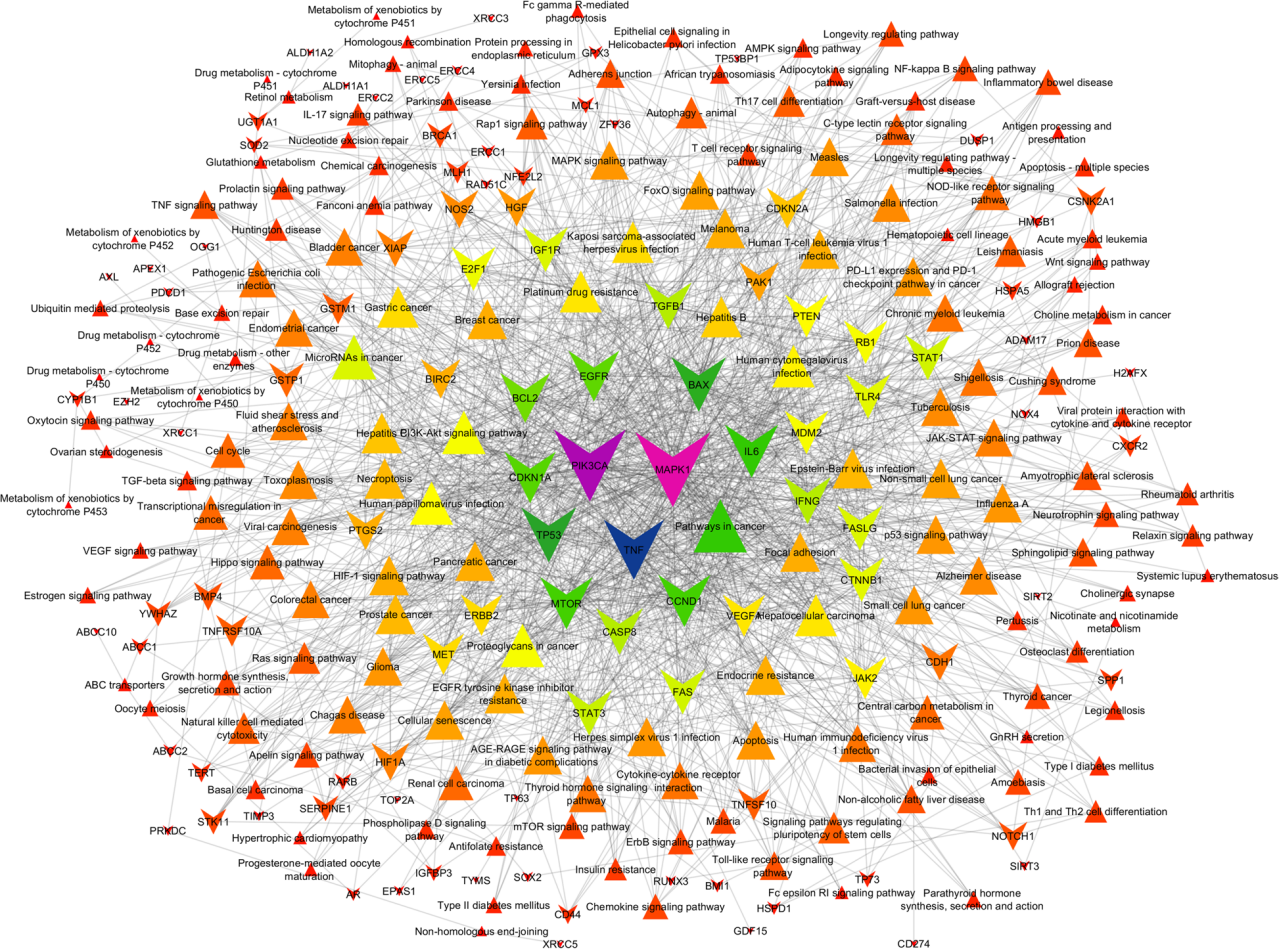
Genes-KEGG pathway interactions

The PPI modulates 150 KEGG pathways via the regulation of 103 different genes (Fig. 3 and Supplementary file 2). Further, the top ten highly modulated pathways in KEGG include pathways in cancer, microRNAs in cancer, platinum drug resistance, proteoglycans in cancer, hepatocellular carcinoma, gastric cancer, human cytomegalovirus infection, melanoma, pancreatic cancer, and prostate cancer (Table 1). Out of these 10 pathways, the most highly modulated pathway, i.e., pathway in cancer (*KEGG entry: hsa05200*) via regulation of 45 genes against 517 background genes with 1.15 strength, at a false discovery rate of 5.82E − 36. Apart from this, the most important pathway observed to be modulated was platinum drug resistance (*KEGG entry: hsa01524*) via regulation of 21 genes (ERCC1, MAPK1, MLH1, MDM2, PIK3CA, TP53, ERBB2, BAX, GSTM1, FAS, CASP8, FASLG, ABCC2, XIAP, BCL2, GSTP1, CDKN1A, TOP2A, CDKN2A, BRCA1, BIRC2) against 70 background genes with 1.69 strength, at false discovery rate of 8.50E − 26.

**Table 1:**
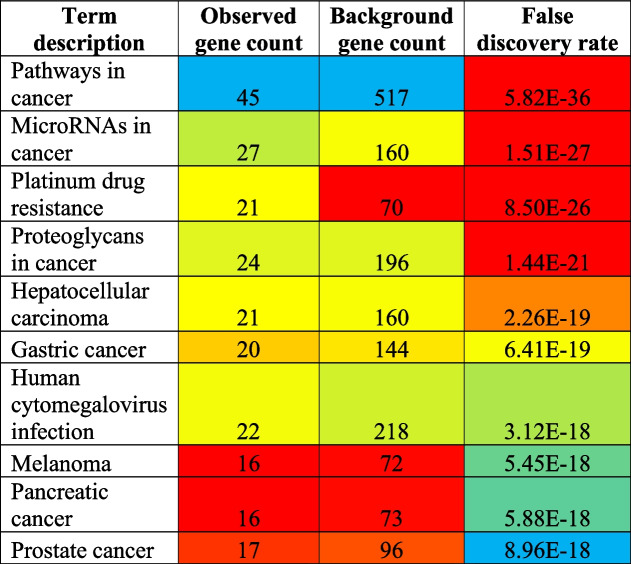
Top 10 KEGG pathways involved in cisplatin-resistance

### Protein–pathway interactions

In the protein–pathway interactions, the node PRKDC was observed to have a maximum average shortest path length and closeness centrality. Further, the node of the pathways in cancer was observed to possess maximum edge count and indegree, i.e., 45 whereas the node of pathways such as metabolism of xenobiotics by cytochrome P453, drug metabolism–cytochrome P451, metabolism of xenobiotics by cytochrome P450, drug metabolism–cytochrome P450, metabolism of xenobiotics by cytochrome P451, metabolism of xenobiotics by cytochrome P452, and drug metabolism–cytochrome P452 have a minimum edge count and indegree, i.e., 1. However, all the nodes were having zero outdegree. Likewise, the node of the Fc epsilon RI signaling pathway had the maximum neighborhood connectivity of 78.67 whereas the nodes of pathways like Base excision repair, nicotinate, and nicotinamide metabolism and non-homologous end-joining had a minimum neighborhood connectivity of 1.5 (Supplementary file 3).

### Gene ontology analysis

#### Cellular components

Similarly, 82 different cellular components were modulated via the regulation of total of 120 different genes (Supplementary file 4). The top ten highly modulated cellular components were intracellular organelle lumen, protein–containing complex, nucleoplasm, chromosome, nuclear chromosome, membrane-bounded organelle, nuclear lumen, nucleus, intracellular membrane-bounded organelle, and organelle (Table 2) out of which the most highly modulated cellular component is intracellular organelle lumen (GO:0070013) via regulation of 85 genes against 5857 background genes with 0.37 strength, at false discovery rate of 7.41E − 17.

**Table 2:**
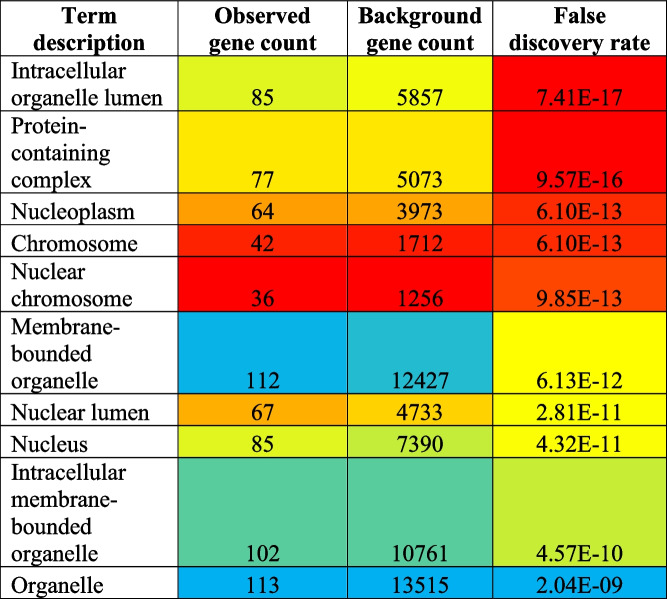
Top 10 cellular components ﻿involved in cisplatin-resistance

#### Molecular function

Furthermore, 123 different molecular functions were modulated via the regulation of total of 118 different genes (Supplementary file 5). The top ten highly modulated molecular functions were protein binding, enzyme binding, identical protein binding, binding, transcription factor binding, DNA binding, organic cyclic compound binding, damaged DNA binding, heterocyclic compound binding, and double-stranded DNA binding (Table 3) out of which the most highly modulated molecular function is protein binding (GO:0005515) via regulation of 106 genes against 7026 background genes with 0.39 strength, at false discovery rate of 5.10E − 29.

**Table 3:**
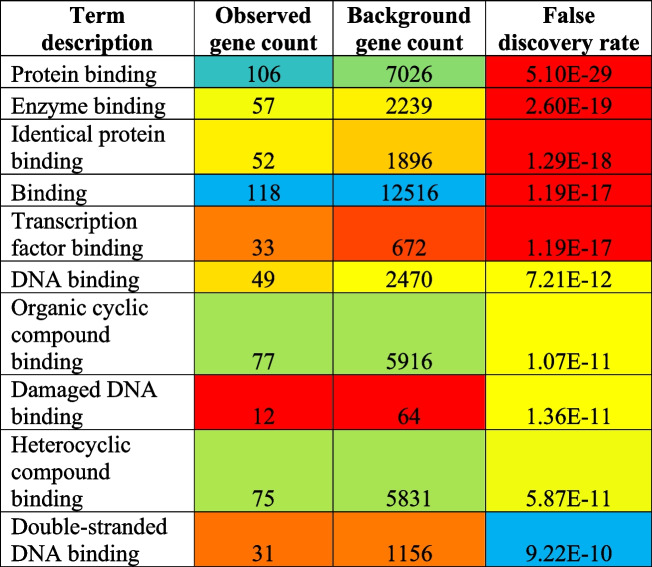
Top 10 molecular functions ﻿involved in cisplatin-resistance

#### Biological process

**Fig. 4:**
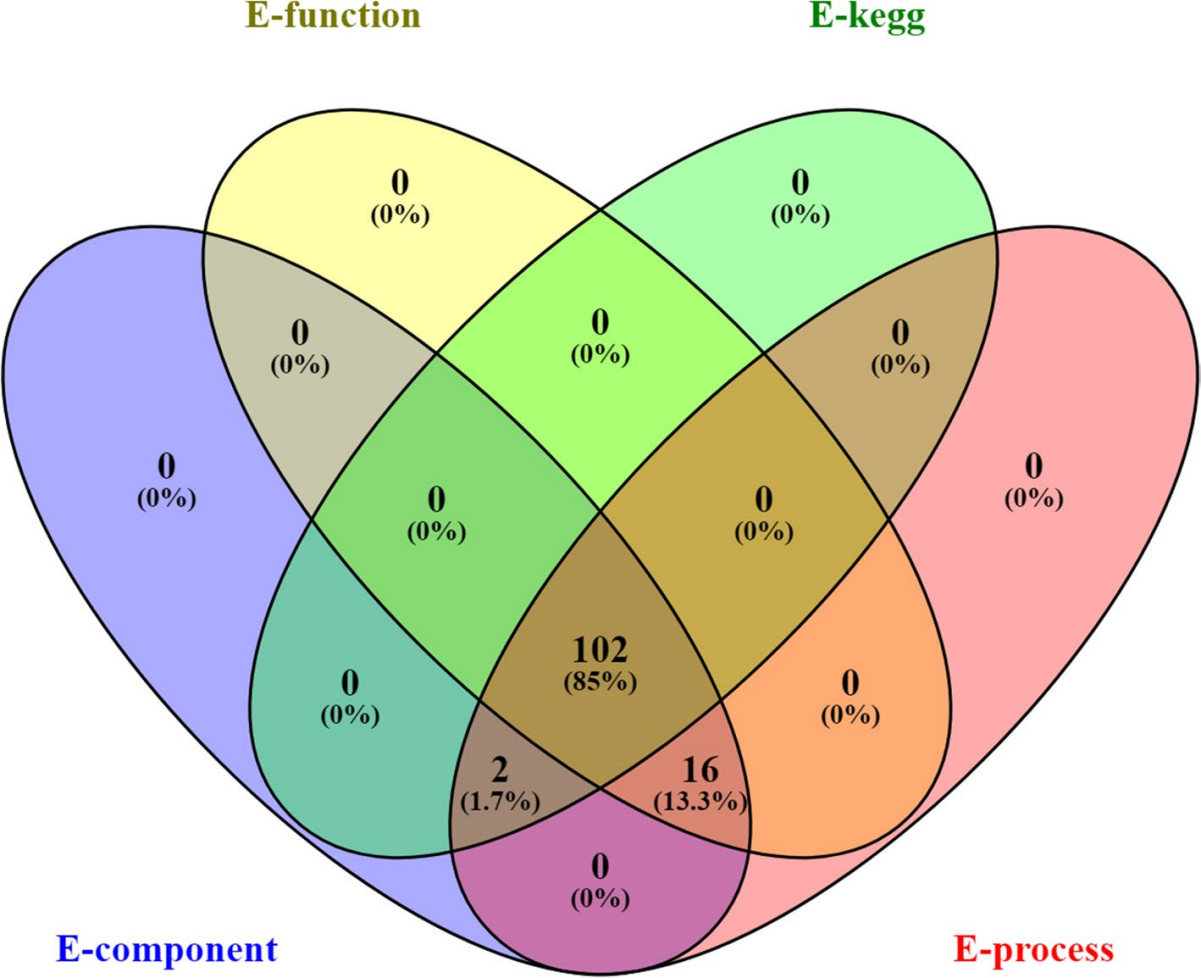
Common genes between KEGG pathways and GO terms

Additionally, 1752 different biological processes were modulated via the regulation of a total of 120 different genes (Supplementary file 6). The top ten highly modulated biological processes were the responses to organic substance, regulation of cell death, regulation of programmed cell death, regulation of the apoptotic process, negative regulation of the biological process, negative regulation of the cellular process, cellular response to an organic substance, cellular response to chemical stimulus, positive regulation of the cellular process, and positive regulation of biological process (Table 4) out of which the most highly modulated biological process was the response to an organic substance (GO:0010033) via regulation of 91 genes against 3011 background genes with 0.69 strength, at false discovery rate of 1.83E − 44. Eighty-five percent of genes were found to be common which contributed towards the modulation of the KEGG pathway, cellular component, molecular function, and biological process (Fig. 4).

**Table 4:**
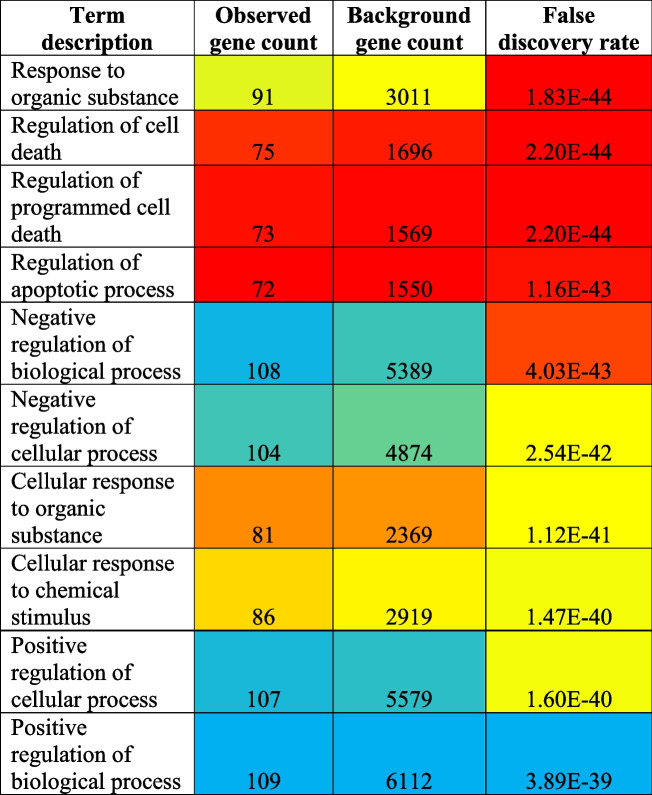
Top 10 biological process ﻿involved in cisplatin-resistance

#### Identification and validation of hub genes

A total of 37 genes were involved in the top ten hub genes obtained by the 12 different topological analysis methods as depicted in Table 5 (Supplementary files 7 and 8). Out of these 37 genes, 31 genes including (*VEGFA*, *CTNNB1*,* EGFR*,* HIF1A*, *TP53*,* TNF*, *PTEN*, *CCND1*, *CDH1*,* ERCC4*, *ERCC5*, *AXL*,* MTOR*, *CD44*, *CDKN2A*,* UGT1A1*,* HDGF*,* CXCR2*,* SLC16A1*, *PAK1*,* ABCC2*,* KDM4C*,* IGFBP3*,* JAK2*,* IGF1R*, *MET*, *MUC1*,* TERT*,* PIK3CA*, *EZH2*, and *AR*) were identified as the significant genes in terms of expression. However, only 7 hub genes were found to have a significant effect on overall survival, i.e., *CCND1*,* IL6*,* ERBB2*,* AXL*,* CDKN2A*,* TERT*, and* EZH2* (Supplementary file 9).

**Table 5:** Common hub genes between the top ten hub genes obtained by 12 different topological analysis methods

Names	Total	Genes
Betweenness, bottle neck, closeness, degree, EPC, EcCentricity, MCC, MNC, radiality, and stress	2	VEGFA, CTNNB1
Betweenness, bottle neck, closeness, degree, EPC, MCC, MNC, radiality, and stress	3	EGFR, HIF1A, TP53
Betweenness, closeness, degree, EPC, EcCentricity, MCC, MNC, radiality, and stress	1	STAT3
Betweenness, bottle neck, closeness, degree, EcCentricity, MNC, radiality, and stress	1	TNF
Betweenness, closeness, degree, EPC, MCC, MNC, radiality, and stress	1	PTEN
Closeness, degree, EPC, EcCentricity, MCC, MNC, and radiality	1	CCND1
Betweenness, bottle neck, degree, EPC, MNC, and stress	1	IL6
Closeness, MCC, radiality, and stress	1	ERBB2
EPC, EcCentricity and MCC	1	CDH1
Clustering coefficient and DMNC	3	ERCC4, ERCC5, AXL
Betweenness	1	MTOR
Bottle neck	3	CD44, CDKN2A, UGT1A1
Clustering coefficient	7	HDGF, CXCR2, SLC16A1, PAK1, ABCC2, GDF15, KDM4C
DMNC	7	BMP4, IGFBP3, JAK2, IGF1R, MET, MUC1, HGF
EcCentricity	4	TERT, PIK3CA, EZH2, AR

**Fig. 5:**
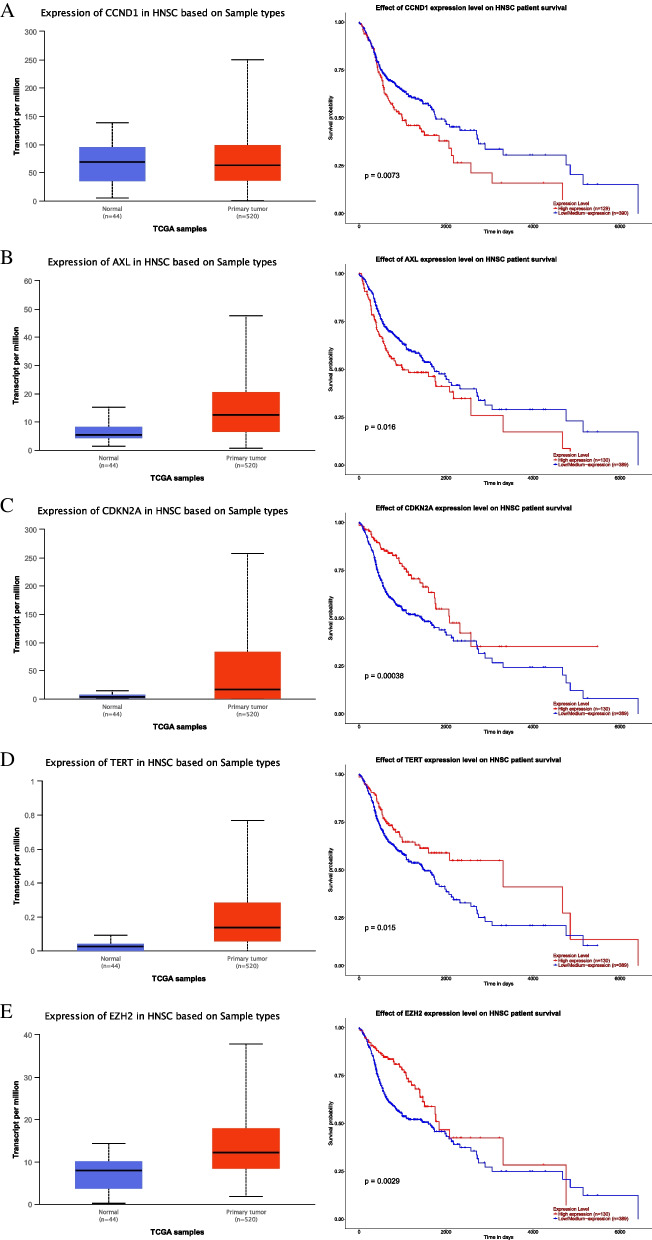
Expression and survival analysis of significant hub genes. **A** Expression and survival analysis of CCND1. **B** Expression and survival analysis of AXL. **C** Expression and survival analysis of CDKN2A. **D** Expression and survival analysis of TERT. **E** Expression and survival analysis of EZH2

Out of 37 genes, 5 genes such as *CCND1*,* AXL*,* CDKN2A*,* TERT*, and *EZH2* were found to have both significant expressions (4.31E − 0.9, 1.63E − 12, < 1E − 12, < 1E − 12, and < 1E − 12) as well as overall survival value (0.0073, 0.016, 0.00038, 0.015, 0.0029) (Fig. 5). Out of these 5 hub genes, only *CCND1* have high median mRNA expression in normal patients than primary tumor whereas *AXL*,* CDKN2A*,* TERT*, and *EZH2* have high expression in the primary tumor. Similarly, the high expression of *CCND1* and *AXL* was found to be associated with poor overall survival whereas high expression of *CDKN2A*,* TERT*, and *EZH2* was found to contribute towards increased overall survival (at least 5-year OS).

**Fig. 6:**
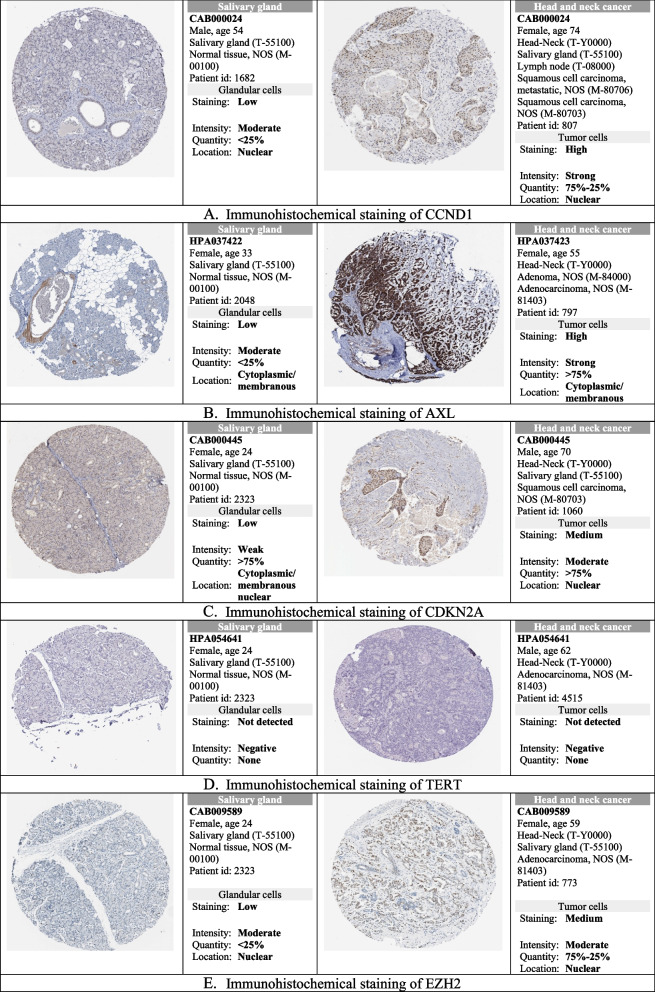
Immunohistochemistry of significant hub genes. **A** Immunohistochemical staining of CCND1. **B** Immunohistochemical staining of AXL. **C** Immunohistochemical staining of CDKN2A. **D** Immunohistochemical staining of TERT. **E** Immunohistochemical staining of EZH2

Further, these 5 significant were investigated for the immunohistochemistry staining which revealed that the protein level expression of these genes was present in the head and neck tumor tissues. Out of 5 genes, the protein expression of *CCND1*,* AXL*,* CDKN2A*, and *EZH2* were found to show a wide range of staining (low-medium–high) based on the types of head and neck tissue such as glandular tissue, squamous cell, oral mucosa, and nasopharynx whereas the protein level was not detected for the *TERT* (Supplementary file: S10). The significant protein level expression in the tumor tissue compared to the normal tissue for the hub genes such as *CCND1*,* AXL*,* CDKN2A*, and *EZH2* is depicted in Fig. 6 as per availability in the database (http://www.proteinatlas.org).

#### Drug-hub gene interactions using DGIdB

**Fig. 7:**
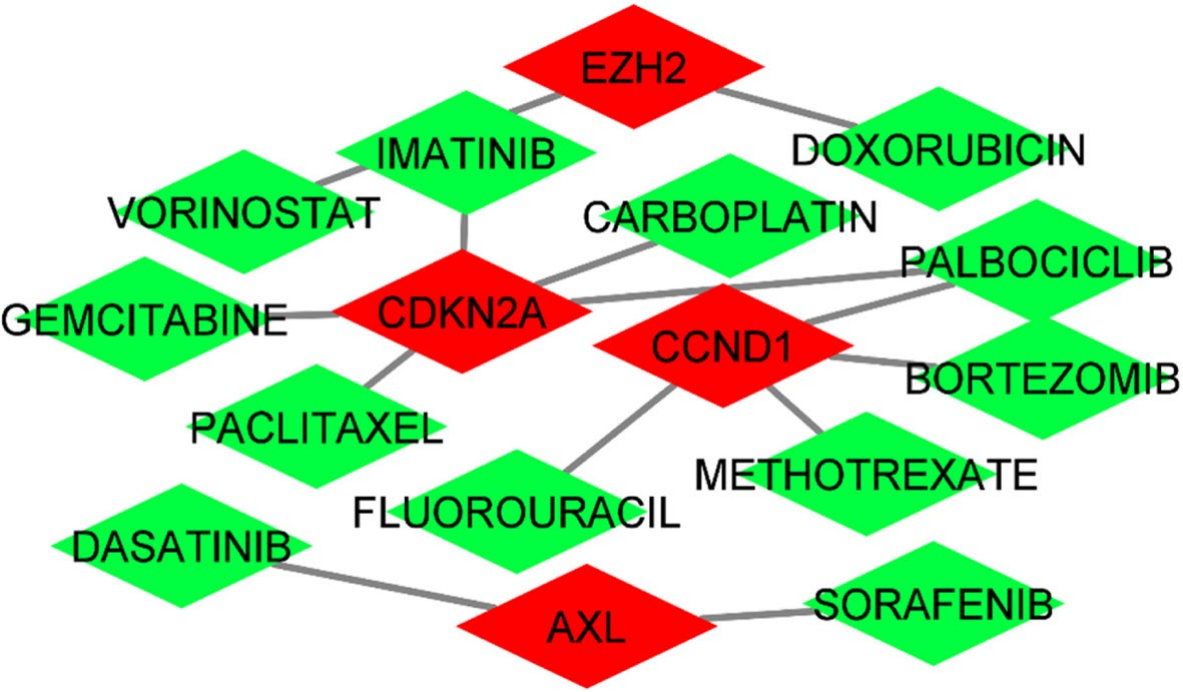
Drug–hub gene interactions

A total of 13 different drug candidates were predicted to interact with hub genes such as *CCND1*,* AXL*,* CDKN2A*, and *EZH2* whereas *TERT* did not interact with any drug (Fig. 7). Further, palbociclib, methotrexate, bortezomib, and fluorouracil were predicted to interact with *CCND1*. Sorafenib, dasatinib and palbociclib, carboplatin, paclitaxel, gemcitabine, and imatinib were predicted to interact with *AXL* and *CDKN2A*, respectively. Likewise, doxorubicin and vorinostat were found to interact with *EZH2*. Thus, these drugs could be useful to target particular hub genes irrespective of the types of cancer and may be added to the therapy to counteract cisplatin resistance once clinical significance is established via pre-clinical/clinical studies.

## Discussion

It has been stated that similar homologous protein from the phylogenic evolution has conserved the protein domain and is similar in them. This indicates that the domain-conserved proteins interact with each other when triggered by any external factors, e.g., xenobiotics. Similarly, in drug resistance, it has been indicated that targeting a single protein can also stimulate or inhibit the function of the homologous protein to fulfill the compromised protein function over the targeted protein. However, the protein–protein interactions of the trigger genes associated with cisplatin resistance in HNC have to be more clarified and declared. Hence, the present study aimed to trace the protein–protein interaction among the genes and pathways associated with cisplatin resistance in HNC.

A total of 150 different KEGG pathways were modulated via the regulation of 103 different genes. The molecular pathophysiology of HNC involves the IL-6-mediated activation of the JAK-STAT pathway via the IL-6 receptor, epidermal growth factor (EGF)-mediated activation of RAS, RAF, MEK, and MAPK signaling molecule via the epidermal growth factor receptor (EGFR) [22]. In our study, the JAK-STAT signaling pathway was predicted to be modulated via the regulation of 12 genes (CCND1, IFNG, PIK3CA, STAT3, EGFR, STAT1, MTOR, MCL1, JAK2, BCL2, CDKN1A, IL6). Further, the MAPK signaling pathway and Ras signaling pathway were modulated via the regulation of 9 genes (MAPK1, HGF, PIK3CA, IGF1R, EGFR, PAK1, MET, FASLG, VEGFA) and 14 genes (MAPK1, TGFB1, HGF, DUSP1, IGF1R, TP53, ERBB2, EGFR, PAK1, MET, FAS, FASLG, TNF, VEGFA), respectively (Supplementary 2).

Additionally, the PI3K-AKT pathway was also modulated via the regulation of 24 genes (Supplementary 2) (MAPK1, HGF, CCND1, MDM2, PIK3CA, IGF1R, TP53, ERBB2, EGFR, MET, STK11, MTOR, FASLG, MCL1, PTEN, TLR4, JAK2, SPP1, YWHAZ, BCL2, CDKN1A, IL6, BRCA1, VEGFA) which is the downstream pathway in the head and neck cancer for the EGF substrate, insulin growth factor I/II, hepatocyte growth factor (HGF) which acts through the EGFR, IGFRI, and c-MET, respectively. Previously, HGF has been reported to mediate signaling through the MAPK pathway [22]. These molecular pathways contribute to the proliferation, invasion, migration, angiogenesis, and survival of tumor cells. Apart from this, the signaling molecule STAT3 leads to VEGF expression which is responsible for angiogenesis by binding to the VEGF receptor on the endothelial lining [22]. We predicted the modulation of the VEGF signaling pathway via the regulation of 4 genes (MAPK1, PIK3CA, PTGS2, VEGFA) (Supplementary 2).

Further, Wnt ligands (wnt1, wnt3a, wnt5a, wnt5b, wnt7a, wnt7b, wnt10b, wnt11, etc.) act on the frizzled receptor which is responsible for the phosphorylation of beta-catenin which mediates cisplatin resistance via wnt-GSK-3β/β-catenin pathway by the expression of MDR1/MRP1 [23, 24]. Wnt signaling pathway (canonical or non-canonical) helps in the proliferation and evade apoptosis of tumor cells via regulation of survivin, c-Myc, and cyclin D1 [23]. We predicted the modulation Wnt signaling pathway via the regulation of 4 genes such as CSNK2A1, CCND1, TP53, and CTNNB1 (Supplementary 2). In addition, Mahopatra et al. investigated the role of CMTM6 in HNC via wnt signaling. It has been reported that CMTM6 maintains the expression of PD-L1 and the regulation of anti-tumor immunity [11].

Our study reported the modulation of PD-L1 expression and PD-1 checkpoint pathway in cancer via 14 different genes (MAPK1, CSNK2A1, IFNG, PIK3CA, STAT3, EGFR, PDCD1, STAT1, MTOR, PTEN, TLR4, CD274, JAK2, and HIF1A) (Supplementary 2). Similarly, the TGF-β signaling pathway is another contributor of chemoresistance for DNA damaging anti-cancerous agents via SMAD-dependent pathway as well as the upregulation of SOX2 and ABCG2 by inhibiting FOXO3a tumor suppressor via AKT pathway [25] whereas we report the modulation of TGF-β signaling pathway via regulation 5 genes (MAPK1, TGFB1, IFNG, BMP4, TNF) through our study (Supplementary 2).

Additionally, the microRNA is also found to be associated with cisplatin resistance. The upregulation of microRNAs such as miR-23a and miR-645-5p activates the JNK-TWIST pathway and inhibits the GRAP-RAS-MAPK pathway respectively leading to cisplatin resistance in HNC. Likewise, the downregulation of microRNA such as miR-15b, miR-24, miR-125a, miR-125b, miR-181a, miR-222, and miR-132 leads to cisplatin resistance among HNC via several pathways [26]. We found the involvement modulation microRNA via the involvement of 27 genes in our study (Supplementary 2). Thus, regulation of all these genes contributes for cisplatin resistance via the modulation of specific pathways among HNC patients.

As the etiopathology of HNC revolves around tobacco products, viral infections, chemical carcinogenesis, etc., the pathway associated with these etiologic agents in the progression of the disease is equally responsible for cisplatin resistance as that of molecular pathways [2]. The carcinogenic compounds from tobacco are converted into electrophilic entities by cytochrome P450 which form adducts with DNA leading to the either deletion (CDKN2A), mutation (TP53), or amplification (PIK3CA) of tumor suppressor genes. This carcinogen might alter the PI3K–AKT–mTOR and RAS–MAPK pathway genes and lead to a poor prognosis of the disease [2, 27].

Similarly, viral infections such as human papillomavirus (HPV) and Epstein Barr virus (EBV) are the causative agents for head and neck cancer [2, 28] which are involved in cisplatin resistance via the regulation of 23 and 17 genes, respectively (Supplementary 2). Early genes such as E6 and E7 of HPV are associated with oncogenic properties. The E6 degrades the p53 by forming a complex with it whereas E7 regulates the destruction of RB1 protein and drives the cell cycle with the help of E2F protein through the G1-S checkpoint [2]. Likewise, the Epstein Barr virus (EBV) is responsible for HNC modulating NF-kB, STAT, and API pathways; activating of PI3K-AKT pathway; and downregulating TP53 [28]. Additionally, the inflammatory mediators (TGF-β and NF-kB) have roles in the transformation, proliferation, invasion, angiogenesis, and metastasis. Our study predicted the modulation of the NF-kB pathway via the regulation of 7 genes (CSNK2A1, PTGS2, XIAP, TLR4, BCL2, TNF, and BIRC2) (Supplementary 2). EGFR/FAK/NF-kB is reported as an important signaling pathway utilized by cancer cells to resist cisplatin [29]. EGFR leads to cisplatin resistance via KRAS/MEK/ERK/ETS-1/β-catenin and PI3K/AKT/MTOR/NF-kB pathways through the expression of MDR1/MRPs and cell proliferation, respectively [30, 31]. Activated FAK acts via three main signaling pathways such as SRC/ERK/ETS-1/β-catenin, PI3K/AKT/MTOR/NF-kB, and P53 suppression resulting in cisplatin resistance through MDR1 overexpression, promoting cell proliferation, and inhibiting apoptosis, respectively [32–34].

Cisplatin targets and destroy DNA to exert its anti-cancerous property [14, 35]. Thus, resistance to cisplatin can be categorized based on drug targets such as pre-target resistance, on-target resistance, post-target resistance, and off-target resistance. The pre-target resistance of cisplatin is due to reduced uptake of cisplatin in the tumor cell, increased efflux of cisplatin from the tumor cell, and increased enzymatic detoxification. The under-expression of SLC22A1, SLC22A2, SLC47A1, CTR1, and CTR2 leads to reduce uptake of cisplatin whereas the upregulation of ATP7A, ATP7B, MRP1, MRP2, and MRP4 are responsible for increased efflux of cisplatin out of the tumor cell. Further, the deactivation of cisplatin by glutathione and metallothionein is another pre-target mechanism of cisplatin resistance [14, 35]. Our study predicted the regulation of ABC transporter via the regulation of 3 genes (ABCC1, ABCC2, ABCC10) (Supplementary 2).

Similarly, on-target resistance occurs due to the increased (NER) nucleotide excision repair capacity (ERCC1, ERCC3, ERCC4, ERCC5), increased translesion synthesis (POLH, REV3, REV7), increased homologous recombination ability (BRCA1, BRCA2), mismatch repair deficiency (MLH1, MSH2/3/6), and cisplatin-binding protein (VDAC). Further, damaged DNA is repaired by non-homologous end joining (NHEJ) via XRCC4 and base excision repair (BER) via XRCC1, APEX1 which confers to cisplatin resistance [14, 35, 36]. We predicted the involvement of DNA repair by modulating NER (ERCC1, ERCC4, ERCC5, ERCC2), homologous recombination (RAD51C, BRCA1, XRCC3), NHEJ (PRKDC, XRCC5), and BER (APEX1, XRCC1, OGG1, HMGB1) pathways (Supplementary 2).

The post-target resistance occurs due to the deficiency in proapoptotic proteins (BAX, BAK), overexpression of anti-apoptotic proteins (BCL-2, BCL-XL, BCL-w, MCL-1), overexpression of BIRC5, and mutation in TP53, etc. [14, 37]. Our study predicted the modulation of the apoptosis pathway via the regulation of 14 different genes (MAPK1, TNFRSF10A, TNFSF10, PIK3CA, TP53, BAX, FAS, CASP8, FASLG, MCL1, XIAP, BCL2, TNF, BIRC2) (Supplementary 2). Additionally, the off-target resistance occurs via deregulated autophagy, ERBB2 overexpression, heat shock proteins (HSPs), and TMEM205 expression [38, 39]. The regulation of PI3K-AKT-MTOR, beclin1, BCL-2, RAS, P53, DUSP1, GFRA1, and HMGB1 are involved in autophagy process. The increased autophagy contributes to cisplatin resistance [14, 38]. ERBB2 (HER2) mediates cisplatin resistance through the PI3K-PDK-AKT pathway due to overexpression of BIRC5 and by phosphorylating p21 which arrests apoptosis [39]. Further, ERBB2 also indirectly inhibits the BAD protein and increases the BCL-2, BCL-XL via ERK. Our study identified the modulation of the ERBB signaling pathway via the regulation of 7 genes such as MAPK1, PIK3CA, ERBB2, EGFR, PAK1, MTOR, and CDKN1A (Supplementary 2). Recently emerging perspectives focused on physical and biological aspects of cisplatin resistance from the micro-environment of a tumor. The parameter pertaining to physical aspects includes the physical barrier to penetration of cisplatin into tumor cells due to high cell density, activation of PI3K-AKT, and ABC transporter due to fluidic shear stress and reduced diffusion of cisplatin from the extracellular matrix. Similarly, biological aspects consist of hypoxia-induced increased stemness of tumor cell and MRP transporter, acidity-induced expression of multidrug transporter, cytokine, and growth factors (IL-6, IL-8, IL-11, EGF, VEGF, HGF, IGF1, TGF-β) that are released by the tumor-associated fibroblast and cytokines secreted by a tumor-associated macrophage (TAM) in M2 polarization states such as IL-6 and type I interferon. Hypoxia in a tumor cell is related to apoptosis as hypoxia-inducible factor-alpha (HIF-α) regulates the apoptotic genes (BCL-2, BAX, caspase 3, caspase 8) as well as a survival signaling pathway, i.e., NF-kB. Thus, overexpression of HIF1α and HIF2α is responsible for the cisplatin resistance [14, 35]. Our study showed the involvement of HIF-1 signaling pathway via the regulation of 16 genes such as MAPK1, SERPINE1, IFNG, PIK3CA, STAT3, IGF1R, ERBB2, EGFR, NOS2, MTOR, TLR4, BCL2, CDKN1A, IL6, HIF1A, and VEGFA (Supplementary 2). Apart from this, the upregulation of RAB8, GCF2, PCAF, G-catenin, Nrf2, HSP (10, 27, 60, 70, 90), SIRT1, and TWIST, etc., promotes cisplatin resistance in tumor cell [39].

TP53 is a tumor suppression gene that is responsible to activate the cell cycle checkpoint, DNA repair, and apoptosis [14]. Any stress to the cell (damage to DNA) or anomalous growth signal activates the p53 gene which induces the expression of the P21 protein leading to cell cycle arrest via inhibition of cyclin-dependent kinase (CDKs) [22]. Similarly, P53 is upregulated by the cisplatin leading to the expression BAX and BID which in turn is responsible for the release of cytochrome-c and caspase activator. These both molecules are activated by caspase 3, 7, and 9 through the apoptosomes and inhibitors of apoptosis protein leading to intrinsic apoptosis. Thus, mutation of TP53 genes is associated with loss of TP53 function which leads to the failure in a checkpoint in the cell cycle, senescence of the cell cycle, and apoptosis resulting in poor clinical outcomes among patients [2, 14, 40]. Further, the downregulation of FAS leads to the failure of cisplatin therapy via suppressed caspase activity (CASP3, CASP8). We predicted the involvement of TP53 signaling pathway modulation via the regulation of 14 different genes such as TNFRSF10A, SERPINE1, CCND1, MDM2, TP53, BAX, FAS, CASP8, PTEN, TP73, IGFBP3, BCL2, CDKN1A, and CDKN2A (Supplementary 2). It has been found that about 72% of squamous cell carcinoma of the head and neck involves the mutation of the TP53 gene which could be one of the major contributing factors toward the resistance to cisplatin chemotherapy [2]. Similarly, mutation of TERT also serves as a prognostic factor for head and neck cancer which reduces the efficacy of cisplatin treatment [41]. Additionally, hub genes such as AXL and EZH2 were also found to be responsible for tumor growth and anti-neoplastic drug resistance/sensitivity [42, 43].

The protein–protein interaction reflects a complex network which is difficult to analyze. However, the understanding of signal flow in the network gives insight into how the expression of a particular gene modulates pathways to cause drug resistance. Targeting the hub gene involved in drug resistance could be one of the best approaches for tackling drug resistance. Although targeting the protein–protein interaction is a challenging task, the drug-targeting PPI could potentially interact with multiple targets to overcome drug resistance by modulating multiple pathways. The challenges pertaining to target PPI include binding of PPI inhibitor to the protein, PPI inhibitor should not change its properties even if the interacting proteins undergo extensive selection and PPI inhibitor should be exerting its action not merely to the proposed target but also its paralog [44]. Despite the challenges, various pathways have been targeted to tackle the cisplatin resistance in the HNC, e.g., wnt/β-catenin inhibitor (WNT974), intracellular apoptotic protein inhibitor (birinapant + carboplatin, DEBIO1143 + cisplatin), EGFR inhibitor (cetuximab + cisplatin), VEGR inhibitor (sorafenib + cisplatin/5-FU, bevacizumab + cisplatin/IMRT), and blockade of PD-1 (nivolumab + cisplatin, pembrolizumab + platinum/5-FU) [45] which is in parallel to the finding of the drug–gene interactions in our study. Thus, various other pathways regulated by the hub gene can be targeted for drug design in the future.

## Conclusion

We conclude that *CCND1*,* AXL*,* CDKN2A*,* TERT*, and *EZH2* are the hub genes involved in cisplatin resistance in head and neck cancer that have significant mRNA expression and effect on overall survival. While only *CCND1*,* AXL*,* CDKN2A*, and *EZH2* have significant protein level expression in the tumor tissue and were predicted to be targeted with various drugs such as palbociclib, methotrexate, bortezomib and fluorouracil, sorafenib, dasatinib, carboplatin, paclitaxel, gemcitabine, imatinib, doxorubicin, and vorinostat which needs to be further investigated via pre-clinical/clinical studies to establish its clinical significance. Cisplatin resistance is a major hurdle in the management of head and neck cancer which occurs via the regulation of multiple genes modulating multiple pathways. Targeting gene-pathway networks may have the potential to overcome cisplatin resistance.

## Supplementary Information

**Additional file 1: S1.** List of genes involved in cisplatin resistance in HNC. **S2.** KEGG pathways. **S3.** Protein-pathway interactions. **S4.** Cellular components. **S5.** Molecular functions. **S6.** Biological components. **S7.** Top ten hub genes via 12 different topological analysis methods. **S8.** Cytohubba calculation using 12 different topological analysis methods. **S9.** *P* value for expression and survival analysis of hub genes. **S10.** Protein expression of hub genes for different tissues of the head and neck. **Figure S1.** Immunohistochemistry of significant hub genes.

## Data Availability

All data generated or analyzed during this study are included in this published article and its supplementary information files.

## Abbreviations

CRT: Chemoradiation therapy
DMNC: Density of maximum neighborhood component
DNA: Deoxyribonucleic acid
EPC: Edge-percolated component
GO: Gene ontology
HNC: Head and neck cancer
R/M HNSCC: Recurrent/metastatic head and neck squamous cell carcinoma
KEGG: Kyoto Encyclopaedia of Genes and Genomes
MCC: Maximal clique centrality
MNC: Maximum neighborhood component
PPI: Protein–protein interactions
