# Supplementary material for: Identification of hub genes involved in cisplatin resistance in head and neck cancer
Source: J Genet Eng Biotechnol. 2023 Jan 30;21:9. doi: 10.1186/s43141-023-00468-y (PMC9886788; doi:10.1186/s43141-023-00468-y)
Supplement: Supplementary file 1 — Additional file 1: S1. List of genes involved in cisplatin resistance in HNC. S2. KEGG pathways. S3. Protein-pathway interactions. S4. Cellular components. S5. Molecular functions. S6. Biological components. S7. Top ten hub genes via 12 different topological analysis methods. S8. Cytohubba calculation using 12 different topological analysis methods. S9. P value for expression and survival analysis of hub genes. S10. Protein expression of hub genes for different tissues of the head and neck. Figure S1. Immunohistochemistry of significant hub genes. [file 43141_2023_468_MOESM1_ESM.docx]

S1: List of Genes involved in cisplatin resistance in HNC

| S No: | GeneID | Symbol | description |
| --- | --- | --- | --- |
|  | 4363 | ABCC1 | ATP binding cassette subfamily C member 1 |
|  | 89845 | ABCC10 | ATP binding cassette subfamily C member 10 |
|  | 1244 | ABCC2 | ATP binding cassette subfamily C member 2 |
|  | 6868 | ADAM17 | ADAM metallopeptidase domain 17 |
|  | 216 | ALDH1A1 | aldehyde dehydrogenase 1 family member A1 |
|  | 8854 | ALDH1A2 | aldehyde dehydrogenase 1 family member A2 |
|  | 328 | APEX1 | apurinic/apyrimidinic endodeoxyribonuclease 1 |
|  | 367 | AR | androgen receptor |
|  | 558 | AXL | AXL receptor tyrosine kinase |
|  | 581 | BAX | BCL2 associated X, apoptosis regulator |
|  | 596 | BCL2 | BCL2 apoptosis regulator |
|  | 329 | BIRC2 | baculoviral IAP repeat containing 2 |
|  | 332 | BIRC5 | baculoviral IAP repeat containing 5 |
|  | 648 | BMI1 | BMI1 proto-oncogene, polycomb ring finger |
|  | 652 | BMP4 | bone morphogenetic protein 4 |
|  | 672 | BRCA1 | BRCA1 DNA repair associated |
|  | 682 | BSG | basigin (Ok blood group) |
|  | 841 | CASP8 | caspase 8 |
|  | 595 | CCND1 | cyclin D1 |
|  | 29126 | CD274 | CD274 molecule |
|  | 960 | CD44 | CD44 molecule (Indian blood group) |
|  | 999 | CDH1 | cadherin 1 |
|  | 1026 | CDKN1A | cyclin dependent kinase inhibitor 1A |
|  | 1029 | CDKN2A | cyclin dependent kinase inhibitor 2A |
|  | 1457 | CSNK2A1 | casein kinase 2 alpha 1 |
|  | 1499 | CTNNB1 | catenin beta 1 |
|  | 3579 | CXCR2 | C-X-C motif chemokine receptor 2 |
|  | 1545 | CYP1B1 | cytochrome P450 family 1 subfamily B member 1 |
|  | 1843 | DUSP1 | dual specificity phosphatase 1 |
|  | 1869 | E2F1 | E2F transcription factor 1 |
|  | 1956 | EGFR | epidermal growth factor receptor |
|  | 1977 | EIF4E | eukaryotic translation initiation factor 4E |
|  | 2034 | EPAS1 | endothelial PAS domain protein 1 |
|  | 2064 | ERBB2 | erb-b2 receptor tyrosine kinase 2 |
|  | 2067 | ERCC1 | ERCC excision repair 1, endonuclease non-catalytic subunit |
|  | 2068 | ERCC2 | ERCC excision repair 2, TFIIH core complex helicase subunit |
|  | 2072 | ERCC4 | ERCC excision repair 4, endonuclease catalytic subunit |
|  | 2073 | ERCC5 | ERCC excision repair 5, endonuclease |
|  | 2146 | EZH2 | enhancer of zeste 2 polycomb repressive complex 2 subunit |
|  | 355 | FAS | Fas cell surface death receptor |
|  | 356 | FASLG | Fas ligand |
|  | 9518 | GDF15 | growth differentiation factor 15 |
|  | 2878 | GPX3 | glutathione peroxidase 3 |
|  | 2944 | GSTM1 | glutathione S-transferase mu 1 |
|  | 2950 | GSTP1 | glutathione S-transferase pi 1 |
|  | 2952 | GSTT1 | glutathione S-transferase theta 1 |
|  | 3014 | H2AX | H2A.X variant histone |
|  | 3068 | HDGF | heparin binding growth factor |
|  | 3082 | HGF | hepatocyte growth factor |
|  | 3091 | HIF1A | hypoxia inducible factor 1 subunit alpha |
|  | 3146 | HMGB1 | high mobility group box 1 |
|  | 3309 | HSPA5 | heat shock protein family A (Hsp70) member 5 |
|  | 3329 | HSPD1 | heat shock protein family D (Hsp60) member 1 |
|  | 3458 | IFNG | interferon gamma |
|  | 3480 | IGF1R | insulin like growth factor 1 receptor |
|  | 3486 | IGFBP3 | insulin like growth factor binding protein 3 |
|  | 3569 | IL6 | interleukin 6 |
|  | 3717 | JAK2 | Janus kinase 2 |
|  | 23081 | KDM4C | lysine demethylase 4C |
|  | 3956 | LGALS1 | galectin 1 |
|  | 5594 | MAPK1 | mitogen-activated protein kinase 1 |
|  | 4170 | MCL1 | MCL1 apoptosis regulator, BCL2 family member |
|  | 4193 | MDM2 | MDM2 proto-oncogene |
|  | 4233 | MET | MET proto-oncogene, receptor tyrosine kinase |
|  | 4255 | MGMT | O-6-methylguanine-DNA methyltransferase |
|  | 406892 | MIR100 | microRNA 100 |
|  | 406928 | MIR137 | microRNA 137 |
|  | 406938 | MIR146A | microRNA 146a |
|  | 406986 | MIR203A | microRNA 203a |
|  | 406988 | MIR205 | microRNA 205 |
|  | 407018 | MIR27A | microRNA 27a |
|  | 407029 | MIR30A | microRNA 30a |
|  | 407040 | MIR34A | microRNA 34a |
|  | 574031 | MIR363 | microRNA 363 |
|  | 406881 | MIRLET7A1 | microRNA let-7a-1 |
|  | 406888 | MIRLET7F1 | microRNA let-7f-1 |
|  | 406891 | MIRLET7I | microRNA let-7i |
|  | 4288 | MKI67 | marker of proliferation Ki-67 |
|  | 4292 | MLH1 | mutL homolog 1 |
|  | 9112 | MTA1 | metastasis associated 1 |
|  | 2475 | MTOR | mechanistic target of rapamycin kinase |
|  | 4582 | MUC1 | mucin 1, cell surface associated |
|  | 4780 | NFE2L2 | NFE2 like bZIP transcription factor 2 |
|  | 4843 | NOS2 | nitric oxide synthase 2 |
|  | 4851 | NOTCH1 | notch receptor 1 |
|  | 50507 | NOX4 | NADPH oxidase 4 |
|  | 4968 | OGG1 | 8-oxoguanine DNA glycosylase |
|  | 5058 | PAK1 | p21 (RAC1) activated kinase 1 |
|  | 5133 | PDCD1 | programmed cell death 1 |
|  | 5164 | PDK2 | pyruvate dehydrogenase kinase 2 |
|  | 5290 | PIK3CA | phosphatidylinositol-4,5-bisphosphate 3-kinase catalytic subunit alpha |
|  | 10631 | POSTN | periostin |
|  | 5591 | PRKDC | protein kinase, DNA-activated, catalytic subunit |
|  | 5728 | PTEN | phosphatase and tensin homolog |
|  | 5743 | PTGS2 | prostaglandin-endoperoxide synthase 2 |
|  | 5888 | RAD51 | RAD51 recombinase |
|  | 5889 | RAD51C | RAD51 paralog C |
|  | 5915 | RARB | retinoic acid receptor beta |
|  | 5925 | RB1 | RB transcriptional corepressor 1 |
|  | 864 | RUNX3 | RUNX family transcription factor 3 |
|  | 6275 | S100A4 | S100 calcium binding protein A4 |
|  | 5054 | SERPINE1 | serpin family E member 1 |
|  | 22933 | SIRT2 | sirtuin 2 |
|  | 23410 | SIRT3 | sirtuin 3 |
|  | 6566 | SLC16A1 | solute carrier family 16 member 1 |
|  | 6648 | SOD2 | superoxide dismutase 2 |
|  | 6657 | SOX2 | SRY-box transcription factor 2 |
|  | 6696 | SPP1 | secreted phosphoprotein 1 |
|  | 6772 | STAT1 | signal transducer and activator of transcription 1 |
|  | 6774 | STAT3 | signal transducer and activator of transcription 3 |
|  | 6794 | STK11 | serine/threonine kinase 11 |
|  | 7015 | TERT | telomerase reverse transcriptase |
|  | 7040 | TGFB1 | transforming growth factor beta 1 |
|  | 7078 | TIMP3 | TIMP metallopeptidase inhibitor 3 |
|  | 7099 | TLR4 | toll like receptor 4 |
|  | 7124 | TNF | tumor necrosis factor |
|  | 8797 | TNFRSF10A | TNF receptor superfamily member 10a |
|  | 8743 | TNFSF10 | TNF superfamily member 10 |
|  | 7153 | TOP2A | DNA topoisomerase II alpha |
|  | 7157 | TP53 | tumor protein p53 |
|  | 7158 | TP53BP1 | tumor protein p53 binding protein 1 |
|  | 8626 | TP63 | tumor protein p63 |
|  | 7161 | TP73 | tumor protein p73 |
|  | 7298 | TYMS | thymidylate synthetase |
|  | 54658 | UGT1A1 | UDP glucuronosyltransferase family 1 member A1 |
|  | 8239 | USP9X | ubiquitin specific peptidase 9 X-linked |
|  | 7422 | VEGFA | vascular endothelial growth factor A |
|  | 331 | XIAP | X-linked inhibitor of apoptosis |
|  | 7515 | XRCC1 | X-ray repair cross complementing 1 |
|  | 7517 | XRCC3 | X-ray repair cross complementing 3 |
|  | 7520 | XRCC5 | X-ray repair cross complementing 5 |
|  | 7534 | YWHAZ | tyrosine 3-monooxygenase/tryptophan 5-monooxygenase activation protein zeta |
|  | 7538 | ZFP36 | ZFP36 ring finger protein |

S2: KEGG Pathways

| #term ID | term description | observed gene count | background gene count | false discovery rate | matching proteins in your network (labels) |
| --- | --- | --- | --- | --- | --- |
| GO:0005515 | Protein binding | 106 | 7026 | 5.10E-29 | ERCC1,MAPK1,LGALS1,APEX1,CSNK2A1,TNFRSF10A,TGFB1,HGF,SERPINE1,  CCND1,IFNG,MLH1,DUSP1,TNFSF10,BMP4,SIRT2,GDF15,MDM2,CDH1,XRCC1,NOX4,EPAS1,PIK3CA,STAT3,TP63,TIMP3,RB1,IGF1R,TP53,ERBB2,EGFR,NOTCH1,PAK1,BAX,AXL,BIRC5,OGG1,TERT,ADAM17,ERCC4,GSTM1,PRKDC,TYMS,USP9X,MET,CXCR2,HSPA5,STK11,NOS2,RARB,MTA1,BSG,CTNNB1,HMGB1,E2F1,ERCC5,FAS,CASP8,STAT1,MTOR,PTGS2,FASLG,HDGF,MKI67,S100A4,MCL1,ABCC2,XIAP,PTEN,UGT1A1,TLR4,AR,BMI1,TP73,POSTN,IGFBP3,KDM4C,JAK2,TP53BP1,RAD51,SIRT3,GPX3,HSPD1,ERCC2,XRCC5,SPP1,YWHAZ,NFE2L2,BCL2,GSTP1,CDKN1A,IL6,TNF,TOP2A,CDKN2A,BRCA1,PDK2,EIF4E,H2AFX,HIF1A,SLC16A1,SOD2,ZFP36,BIRC2,VEGFA,MUC1 |
| GO:0019899 | Enzyme binding | 57 | 2239 | 2.60E-19 | MAPK1,TNFRSF10A,TGFB1,SERPINE1,CCND1,MLH1,DUSP1,SIRT2,MDM2,XRCC1,NOX4,EPAS1,STAT3,TIMP3,RB1,TP53,ERBB2,EGFR,NOTCH1,PAK1,BIRC5,GSTM1,PRKDC,MET,HSPA5,MTA1,CTNNB1,HMGB1,E2F1,ERCC5,FAS,CASP8,STAT1,MTOR,PTGS2,PTEN,UGT1A1,AR,TP73,KDM4C,JAK2,RAD51,SIRT3,HSPD1,XRCC5,YWHAZ,BCL2,GSTP1,CDKN1A,TNF,TOP2A,CDKN2A,BRCA1,EIF4E,H2AFX,HIF1A,ZFP36 |
| GO:0042802 | Identical protein binding | 52 | 1896 | 1.29E-18 | MAPK1,LGALS1,CSNK2A1,TNFRSF10A,TGFB1,HGF,TNFSF10,GDF15,MDM2,CDH1,STAT3,TP63,RB1,IGF1R,TP53,ERBB2,EGFR,PAK1,BAX,BIRC5,TERT,ERCC4,GSTM1,TYMS,MET,NOS2,ERCC5,FAS,CASP8,STAT1,MTOR,PTGS2,S100A4,MCL1,XIAP,PTEN,UGT1A1,TLR4,TP73,JAK2,RAD51,GPX3,YWHAZ,BCL2,TNF,TOP2A,BRCA1,PDK2,SLC16A1,SOD2,BIRC2,VEGFA |
| GO:0005488 | Binding | 118 | 12516 | 1.19E-17 | ERCC1,MAPK1,LGALS1,APEX1,CSNK2A1,TNFRSF10A,TGFB1,HGF,SERPINE1,CCND1,IFNG,MLH1,DUSP1,TNFSF10,BMP4,SIRT2,ALDH1A2,GDF15,MDM2,CDH1,XRCC1,NOX4,EPAS1,PIK3CA,STAT3,TP63,TIMP3,RB1,IGF1R,TP53,ERBB2,EGFR,NOTCH1,PAK1,BAX,ALDH1A1,AXL,BIRC5,MGMT,OGG1,TERT,ADAM17,ERCC4,GSTM1,PRKDC,TYMS,USP9X,MET,CXCR2,EZH2,SOX2,HSPA5,STK11,NOS2,RARB,MTA1,BSG,RAD51C,CTNNB1,HMGB1,E2F1,ERCC5,FAS,CASP8,STAT1,MTOR,PTGS2,FASLG,HDGF,MKI67,S100A4,MCL1,ABCC2,XIAP,PTEN,ABCC10,UGT1A1,TLR4,AR,BMI1,TP73,POSTN,IGFBP3,KDM4C,JAK2,TP53BP1,RAD51,SIRT3,GPX3,HSPD1,ERCC2,XRCC5,SPP1,YWHAZ,NFE2L2,BCL2,GSTP1,ABCC1,RUNX3,CDKN1A,IL6,CD44,TNF,TOP2A,CDKN2A,BRCA1,PDK2,EIF4E,H2AFX,HIF1A,SLC16A1,SOD2,XRCC3,ZFP36,BIRC2,CYP1B1,VEGFA,MUC1 |
| GO:0008134 | Transcription factor binding | 33 | 672 | 1.19E-17 | ERCC1,MAPK1,APEX1,TNFRSF10A,CCND1,SIRT2,EPAS1,STAT3,RB1,TP53,TERT,  ERCC4,PRKDC,RARB,MTA1,CTNNB1,HMGB1,E2F1,STAT1,MTOR,HDGF,AR,TP73,KDM4C,TP53BP1,GPX3,YWHAZ,NFE2L2,BCL2,CDKN2A,BRCA1,EIF4E,HIF1A |
| GO:0003677 | DNA binding | 49 | 2470 | 7.21E-12 | ERCC1,MAPK1,APEX1,MLH1,XRCC1,EPAS1,STAT3,TP63,RB1,TP53,EGFR,NOTCH1,MGMT,OGG1,TERT,ERCC4,PRKDC,EZH2,SOX2,RARB,MTA1,RAD51C,HMGB1,E2F1,ERCC5,STAT1,MTOR,HDGF,MKI67,AR,TP73,TP53BP1,RAD51,SIRT3,HSPD1,ERCC2,XRCC5,NFE2L2,BCL2,RUNX3,TNF,TOP2A,CDKN2A,BRCA1,H2AFX,HIF1A,XRCC3,ZFP36,MUC1 |
| GO:0097159 | Organic cyclic compound binding | 77 | 5916 | 1.07E-11 | ERCC1,MAPK1,LGALS1,APEX1,CSNK2A1,MLH1,SIRT2,MDM2,XRCC1,NOX4,EPAS1,PIK3CA,STAT3,TP63,RB1,IGF1R,TP53,ERBB2,EGFR,NOTCH1,PAK1,ALDH1A1,AXL,MGMT,OGG1,TERT,ERCC4,PRKDC,TYMS,MET,EZH2,SOX2,HSPA5,STK11,NOS2,RARB,MTA1,RAD51C,HMGB1,E2F1,ERCC5,STAT1,MTOR,PTGS2,HDGF,MKI67,S100A4,ABCC2,ABCC10,UGT1A1,AR,TP73,JAK2,TP53BP1,RAD51,SIRT3,HSPD1,ERCC2,XRCC5,YWHAZ,NFE2L2,BCL2,ABCC1,RUNX3,TNF,TOP2A,CDKN2A,BRCA1,PDK2,EIF4E,H2AFX,HIF1A,SLC16A1,XRCC3,ZFP36,CYP1B1,MUC1 |
| GO:0003684 | Damaged dna binding | 12 | 64 | 1.36E-11 | ERCC1,APEX1,XRCC1,TP63,OGG1,ERCC4,HMGB1,TP53BP1,ERCC2,XRCC5,BRCA1,H2AFX |
| GO:1901363 | Heterocyclic compound binding | 75 | 5831 | 5.87E-11 | ERCC1,MAPK1,LGALS1,APEX1,CSNK2A1,MLH1,SIRT2,MDM2,XRCC1,NOX4,EPAS1,PIK3CA,STAT3,TP63,RB1,IGF1R,TP53,ERBB2,EGFR,NOTCH1,PAK1,ALDH1A1,AXL,MGMT,OGG1,TERT,ERCC4,PRKDC,TYMS,MET,EZH2,SOX2,HSPA5,STK11,NOS2,RARB,MTA1,RAD51C,HMGB1,E2F1,ERCC5,STAT1,MTOR,PTGS2,HDGF,MKI67,S100A4,ABCC2,ABCC10,AR,TP73,JAK2,TP53BP1,RAD51,SIRT3,HSPD1,ERCC2,XRCC5,YWHAZ,NFE2L2,BCL2,ABCC1,RUNX3,TNF,TOP2A,CDKN2A,BRCA1,PDK2,EIF4E,H2AFX,HIF1A,XRCC3,ZFP36,CYP1B1,MUC1 |
| GO:0003690 | Double-stranded dna binding | 31 | 1156 | 9.22E-10 | MAPK1,APEX1,MLH1,EPAS1,STAT3,TP63,RB1,TP53,EGFR,NOTCH1,PRKDC,EZH2,SOX2,RARB,MTA1,HMGB1,E2F1,ERCC5,STAT1,MTOR,AR,TP73,RAD51,HSPD1,XRCC5,NFE2L2,RUNX3,TNF,BRCA1,HIF1A,MUC1 |
| GO:0005102 | Signaling receptor binding | 35 | 1581 | 4.64E-09 | TGFB1,HGF,SERPINE1,IFNG,TNFSF10,BMP4,GDF15,MDM2,STAT3,RB1,IGF1R,TP53,ERBB2,EGFR,NOTCH1,ADAM17,RARB,CTNNB1,HMGB1,CASP8,STAT1,FASLG,HDGF,S100A4,PTEN,TLR4,AR,KDM4C,JAK2,SPP1,IL6,TNF,BRCA1,HIF1A,VEGFA |
| GO:0019904 | Protein domain specific binding | 24 | 716 | 4.64E-09 | ERCC1,MDM2,TP63,RB1,TP53,BAX,ADAM17,PRKDC,HSPA5,STK11,CTNNB1,CASP8,MTOR,MCL1,ABCC2,PTEN,AR,BMI1,JAK2,YWHAZ,NFE2L2,BCL2,CDKN2A,HIF1A |
| GO:0002039 | p53 binding | 10 | 69 | 1.22E-08 | MDM2,TP63,TP53,STK11,TP73,TP53BP1,HSPD1,CDKN2A,HIF1A,MUC1 |
| GO:0098772 | Molecular function regulator | 51 | 3422 | 3.97E-08 | APEX1,TGFB1,HGF,SERPINE1,CCND1,IFNG,TNFSF10,BMP4,GDF15,EPAS1,PIK3CA,STAT3,TP63,TIMP3,RB1,TP53,NOTCH1,ALDH1A1,BIRC5,EZH2,SOX2,STK11,RARB,MTA1,CTNNB1,HMGB1,E2F1,STAT1,FASLG,HDGF,XIAP,UGT1A1,AR,TP73,IGFBP3,TP53BP1,XRCC5,SPP1,NFE2L2,BCL2,GSTP1,RUNX3,CDKN1A,IL6,TNF,CDKN2A,BRCA1,HIF1A,BIRC2,VEGFA,MUC1 |
| GO:0019900 | Kinase binding | 23 | 742 | 4.30E-08 | MAPK1,CCND1,DUSP1,NOX4,STAT3,RB1,TP53,EGFR,PAK1,CTNNB1,E2F1,FAS,MTOR,PTEN,TP73,JAK2,YWHAZ,GSTP1,CDKN1A,TOP2A,CDKN2A,HIF1A,ZFP36 |
| GO:0003676 | Nucleic acid binding | 55 | 3947 | 5.36E-08 | ERCC1,MAPK1,LGALS1,APEX1,MLH1,MDM2,XRCC1,EPAS1,STAT3,TP63,RB1,TP53,EGFR,NOTCH1,MGMT,OGG1,TERT,ERCC4,PRKDC,TYMS,EZH2,SOX2,RARB,MTA1,RAD51C,HMGB1,E2F1,ERCC5,STAT1,MTOR,HDGF,MKI67,S100A4,AR,TP73,TP53BP1,RAD51,SIRT3,HSPD1,ERCC2,XRCC5,YWHAZ,NFE2L2,BCL2,RUNX3,TNF,TOP2A,CDKN2A,BRCA1,EIF4E,H2AFX,HIF1A,XRCC3,ZFP36,MUC1 |
| GO:0036094 | Small molecule binding | 42 | 2516 | 1.05E-07 | MAPK1,CSNK2A1,MLH1,SIRT2,ALDH1A2,NOX4,PIK3CA,IGF1R,TP53,ERBB2,EGFR,PAK1,ALDH1A1,AXL,PRKDC,TYMS,MET,HSPA5,STK11,NOS2,BSG,RAD51C,MTOR,HDGF,MKI67,ABCC2,ABCC10,UGT1A1,JAK2,RAD51,SIRT3,HSPD1,ERCC2,XRCC5,GSTP1,ABCC1,RUNX3,CD44,TOP2A,PDK2,XRCC3,CYP1B1 |
| GO:0043167 | Ion binding | 71 | 6188 | 1.05E-07 | MAPK1,APEX1,CSNK2A1,MLH1,TNFSF10,BMP4,SIRT2,MDM2,CDH1,NOX4,PIK3CA,TP63,TIMP3,IGF1R,TP53,ERBB2,EGFR,NOTCH1,PAK1,AXL,BIRC5,MGMT,TERT,ADAM17,GSTM1,PRKDC,TYMS,MET,HSPA5,STK11,NOS2,RARB,MTA1,RAD51C,HMGB1,ERCC5,MTOR,PTGS2,HDGF,MKI67,S100A4,ABCC2,XIAP,ABCC10,UGT1A1,AR,BMI1,TP73,POSTN,IGFBP3,KDM4C,JAK2,RAD51,SIRT3,HSPD1,ERCC2,XRCC5,GSTP1,ABCC1,RUNX3,CDKN1A,CD44,TOP2A,BRCA1,PDK2,SOD2,XRCC3,ZFP36,BIRC2,CYP1B1,VEGFA |
| GO:0019901 | Protein kinase binding | 21 | 653 | 1.22E-07 | MAPK1,CCND1,DUSP1,NOX4,STAT3,TP53,EGFR,PAK1,CTNNB1,E2F1,MTOR,PTEN,TP73,JAK2,YWHAZ,GSTP1,CDKN1A,TOP2A,CDKN2A,HIF1A,ZFP36 |
| GO:0140297 | DNA-binding transcription factor binding | 16 | 366 | 2.37E-07 | APEX1,STAT3,RB1,RARB,MTA1,CTNNB1,HMGB1,STAT1,KDM4C,TP53BP1,NFE2L2,BCL2,CDKN2A,BRCA1,EIF4E,HIF1A |
| GO:0003824 | Catalytic activity | 65 | 5486 | 2.89E-07 | ERCC1,MAPK1,APEX1,CSNK2A1,HGF,CCND1,MLH1,DUSP1,SIRT2,ALDH1A2,MDM2,XRCC1,NOX4,PIK3CA,IGF1R,ERBB2,EGFR,PAK1,ALDH1A1,AXL,MGMT,OGG1,TERT,ADAM17,ERCC4,GSTM1,PRKDC,TYMS,USP9X,MET,EZH2,HSPA5,STK11,NOS2,RAD51C,HMGB1,ERCC5,CASP8,MTOR,PTGS2,ABCC2,XIAP,PTEN,ABCC10,UGT1A1,TLR4,KDM4C,JAK2,RAD51,SIRT3,GPX3,HSPD1,ERCC2,XRCC5,GSTP1,ABCC1,CDKN1A,TOP2A,CDKN2A,BRCA1,PDK2,SOD2,XRCC3,BIRC2,CYP1B1 |
| GO:0043565 | Sequence-specific dna binding | 29 | 1331 | 2.92E-07 | APEX1,EPAS1,STAT3,TP63,RB1,TP53,NOTCH1,TERT,EZH2,SOX2,RARB,MTA1,HMGB1,E2F1,STAT1,MTOR,AR,TP73,TP53BP1,SIRT3,HSPD1,XRCC5,NFE2L2,BCL2,RUNX3,TNF,BRCA1,HIF1A,MUC1 |
| GO:0003682 | Chromatin binding | 19 | 570 | 3.93E-07 | ERCC1,APEX1,MLH1,SIRT2,STAT3,TP63,TP53,EGFR,NOTCH1,ERCC4,EZH2,MTA1,CTNNB1,STAT1,AR,BMI1,KDM4C,RAD51,TOP2A |
| GO:0140097 | Catalytic activity, acting on dna | 12 | 184 | 3.93E-07 | APEX1,XRCC1,MGMT,OGG1,TERT,ERCC4,RAD51C,ERCC5,RAD51,ERCC2,XRCC5,TOP2A |
| GO:0043168 | Anion binding | 43 | 2805 | 5.72E-07 | MAPK1,CSNK2A1,MLH1,BMP4,SIRT2,NOX4,PIK3CA,IGF1R,TP53,ERBB2,EGFR,PAK1,AXL,GSTM1,PRKDC,TYMS,MET,HSPA5,STK11,NOS2,RAD51C,HMGB1,MTOR,HDGF,MKI67,ABCC2,ABCC10,UGT1A1,POSTN,JAK2,RAD51,SIRT3,HSPD1,ERCC2,XRCC5,GSTP1,ABCC1,RUNX3,CD44,TOP2A,PDK2,XRCC3,VEGFA |
| GO:0000166 | Nucleotide binding | 36 | 2119 | 1.25E-06 | MAPK1,CSNK2A1,MLH1,SIRT2,NOX4,PIK3CA,IGF1R,TP53,ERBB2,EGFR,PAK1,ALDH1A1,AXL,PRKDC,TYMS,MET,HSPA5,STK11,NOS2,RAD51C,MTOR,HDGF,MKI67,ABCC2,ABCC10,JAK2,RAD51,SIRT3,HSPD1,ERCC2,XRCC5,ABCC1,RUNX3,TOP2A,PDK2,XRCC3 |
| GO:0097367 | Carbohydrate derivative binding | 37 | 2226 | 1.25E-06 | MAPK1,CSNK2A1,MLH1,BMP4,PIK3CA,IGF1R,TP53,ERBB2,EGFR,PAK1,AXL,PRKDC,MET,HSPA5,STK11,NOS2,RAD51C,HMGB1,MTOR,HDGF,MKI67,ABCC2,ABCC10,TLR4,POSTN,JAK2,RAD51,HSPD1,ERCC2,XRCC5,ABCC1,RUNX3,CD44,TOP2A,PDK2,XRCC3,VEGFA |
| GO:0044389 | Ubiquitin-like protein ligase binding | 14 | 312 | 1.36E-06 | MDM2,RB1,TP53,EGFR,HSPA5,CASP8,STAT1,HSPD1,XRCC5,YWHAZ,BCL2,CDKN1A,BRCA1,HIF1A |
| GO:0005524 | ATP binding | 29 | 1464 | 1.75E-06 | MAPK1,CSNK2A1,MLH1,PIK3CA,IGF1R,TP53,ERBB2,EGFR,PAK1,AXL,PRKDC,MET,HSPA5,STK11,RAD51C,MTOR,MKI67,ABCC2,ABCC10,JAK2,RAD51,HSPD1,ERCC2,XRCC5,ABCC1,RUNX3,TOP2A,PDK2,XRCC3 |
| GO:0061629 | RNA polymerase II-specific DNA-binding transcription factor binding | 13 | 283 | 3.35E-06 | APEX1,STAT3,RB1,RARB,MTA1,CTNNB1,STAT1,KDM4C,TP53BP1,NFE2L2,CDKN2A,BRCA1,HIF1A |
| GO:1990837 | Sequence-specific double-stranded dna binding | 24 | 1068 | 3.69E-06 | EPAS1,STAT3,TP63,RB1,TP53,NOTCH1,EZH2,SOX2,RARB,MTA1,HMGB1,E2F1,STAT1,MTOR,AR,TP73,HSPD1,XRCC5,NFE2L2,RUNX3,TNF,BRCA1,HIF1A,MUC1 |
| GO:0031625 | Ubiquitin protein ligase binding | 13 | 296 | 4.92E-06 | MDM2,RB1,TP53,EGFR,HSPA5,CASP8,HSPD1,XRCC5,YWHAZ,BCL2,CDKN1A,BRCA1,HIF1A |
| GO:0008022 | Protein c-terminus binding | 11 | 199 | 5.92E-06 | ERCC1,ERBB2,TERT,ERCC4,CTNNB1,MKI67,JAK2,RAD51,ERCC2,XRCC5,TOP2A |
| GO:0000976 | Transcription regulatory region sequence-specific dna binding | 23 | 1028 | 6.98E-06 | EPAS1,STAT3,TP63,RB1,TP53,NOTCH1,EZH2,SOX2,RARB,MTA1,HMGB1,E2F1,STAT1,MTOR,AR,TP73,XRCC5,NFE2L2,RUNX3,TNF,BRCA1,HIF1A,MUC1 |
| GO:0046983 | Protein dimerization activity | 23 | 1037 | 7.72E-06 | GDF15,EPAS1,STAT3,TP53,ERBB2,BAX,BIRC5,TERT,GSTM1,NOS2,E2F1,ERCC5,STAT1,PTGS2,MCL1,UGT1A1,TLR4,BCL2,TOP2A,PDK2,H2AFX,HIF1A,VEGFA |
| GO:0044877 | Protein-containing complex binding | 25 | 1216 | 7.90E-06 | ERCC1,APEX1,HGF,CCND1,MDM2,IGF1R,TP53,EGFR,PAK1,ADAM17,ERCC4,EZH2,HSPA5,RARB,HMGB1,ERCC5,CASP8,MTOR,PTEN,HSPD1,XRCC5,SPP1,CDKN1A,CD44,BIRC2 |
| GO:0005126 | Cytokine receptor binding | 12 | 264 | 9.86E-06 | TGFB1,IFNG,TNFSF10,STAT3,ADAM17,CASP8,STAT1,FASLG,JAK2,IL6,TNF,VEGFA |
| GO:0005125 | Cytokine activity | 11 | 233 | 2.31E-05 | TGFB1,IFNG,TNFSF10,BMP4,GDF15,HMGB1,FASLG,SPP1,IL6,TNF,VEGFA |
| GO:0004672 | Protein kinase activity | 16 | 568 | 3.95E-05 | MAPK1,CSNK2A1,CCND1,PIK3CA,IGF1R,ERBB2,EGFR,PAK1,AXL,PRKDC,MET,STK11,MTOR,JAK2,CDKN1A,PDK2 |
| GO:0070491 | Repressing transcription factor binding | 7 | 73 | 4.24E-05 | STAT3,MTA1,CTNNB1,HMGB1,STAT1,BCL2,EIF4E |
| GO:0047485 | Protein n-terminus binding | 8 | 110 | 4.53E-05 | CSNK2A1,MDM2,TP53,TERT,ERCC4,ERCC5,ERCC2,BIRC2 |
| GO:0097371 | MDM2/MDM4 family protein binding | 4 | 9 | 6.33E-05 | TP63,TP53,TP73,CDKN2A |
| GO:0032553 | Ribonucleotide binding | 30 | 1880 | 6.34E-05 | MAPK1,CSNK2A1,MLH1,PIK3CA,IGF1R,TP53,ERBB2,EGFR,PAK1,AXL,PRKDC,MET,HSPA5,STK11,NOS2,RAD51C,MTOR,MKI67,ABCC2,ABCC10,JAK2,RAD51,HSPD1,ERCC2,XRCC5,ABCC1,RUNX3,TOP2A,PDK2,XRCC3 |
| GO:0046982 | Protein heterodimerization activity | 12 | 338 | 0.0001 | EPAS1,TP53,ERBB2,BAX,BIRC5,MCL1,UGT1A1,TLR4,BCL2,TOP2A,H2AFX,HIF1A |
| GO:0035035 | Histone acetyltransferase binding | 5 | 29 | 0.00012 | SIRT2,EPAS1,TP53,STAT1,HIF1A |
| GO:0005164 | Tumor necrosis factor receptor binding | 5 | 31 | 0.00016 | TNFSF10,CASP8,STAT1,FASLG,TNF |
| GO:0002020 | Protease binding | 8 | 138 | 0.00019 | TNFRSF10A,SERPINE1,TIMP3,TP53,PTEN,HSPD1,BCL2,TNF |
| GO:0051427 | Hormone receptor binding | 9 | 188 | 0.0002 | STAT3,RB1,RARB,CTNNB1,STAT1,KDM4C,JAK2,BRCA1,HIF1A |
| GO:0140096 | Catalytic activity, acting on a protein | 31 | 2116 | 0.0002 | MAPK1,CSNK2A1,HGF,CCND1,DUSP1,SIRT2,MDM2,PIK3CA,IGF1R,ERBB2,EGFR,PAK1,AXL,ADAM17,PRKDC,USP9X,MET,EZH2,STK11,CASP8,MTOR,XIAP,PTEN,KDM4C,JAK2,SIRT3,CDKN1A,CDKN2A,BRCA1,PDK2,BIRC2 |
| GO:1990841 | Promoter-specific chromatin binding | 6 | 61 | 0.0002 | ERCC1,TP53,ERCC4,EZH2,STAT1,BMI1 |
| GO:0097718 | Disordered domain specific binding | 5 | 34 | 0.00021 | MDM2,RB1,TP53,CTNNB1,CDKN2A |
| GO:0019902 | Phosphatase binding | 9 | 194 | 0.00023 | MAPK1,STAT3,TP53,ERBB2,EGFR,MET,CTNNB1,STAT1,BCL2 |
| GO:0042803 | Protein homodimerization activity | 16 | 673 | 0.00024 | GDF15,STAT3,BAX,BIRC5,TERT,GSTM1,NOS2,ERCC5,STAT1,PTGS2,MCL1,UGT1A1,BCL2,TOP2A,PDK2,VEGFA |
| GO:0004520 | Endodeoxyribonuclease activity | 5 | 36 | 0.00025 | APEX1,XRCC1,ERCC4,RAD51C,ERCC5 |
| GO:0019903 | Protein phosphatase binding | 8 | 149 | 0.00028 | STAT3,TP53,ERBB2,EGFR,MET,CTNNB1,STAT1,BCL2 |
| GO:0051087 | Chaperone binding | 7 | 105 | 0.00028 | TP53,BAX,BIRC5,TERT,HSPA5,HSPD1,BIRC2 |
| GO:0016887 | ATPase activity | 12 | 393 | 0.00035 | MLH1,HSPA5,RAD51C,ABCC2,ABCC10,RAD51,HSPD1,ERCC2,XRCC5,ABCC1,TOP2A,XRCC3 |
| GO:0035257 | Nuclear hormone receptor binding | 8 | 155 | 0.00035 | STAT3,RB1,RARB,CTNNB1,STAT1,KDM4C,BRCA1,HIF1A |
| GO:0140110 | Transcription regulator activity | 26 | 1657 | 0.00038 | APEX1,CCND1,EPAS1,STAT3,TP63,RB1,TP53,NOTCH1,EZH2,SOX2,RARB,MTA1,CTNNB1,HMGB1,E2F1,STAT1,HDGF,AR,TP73,TP53BP1,NFE2L2,RUNX3,BRCA1,HIF1A,BIRC2,MUC1 |
| GO:0140296 | General transcription initiation factor binding | 5 | 41 | 0.00041 | ERCC1,TP53,ERCC4,MTOR,AR |
| GO:0001091 | RNA polymerase II general transcription initiation factor binding | 4 | 19 | 0.0005 | ERCC1,TP53,ERCC4,AR |
| GO:0048018 | Receptor ligand activity | 13 | 490 | 0.00057 | TGFB1,HGF,IFNG,TNFSF10,BMP4,GDF15,HMGB1,FASLG,HDGF,SPP1,IL6,TNF,VEGFA |
| GO:0004519 | Endonuclease activity | 7 | 120 | 0.00058 | ERCC1,APEX1,XRCC1,OGG1,ERCC4,RAD51C,ERCC5 |
| GO:0016740 | Transferase activity | 30 | 2170 | 0.00071 | MAPK1,CSNK2A1,CCND1,SIRT2,MDM2,PIK3CA,IGF1R,ERBB2,EGFR,PAK1,AXL,MGMT,TERT,GSTM1,PRKDC,TYMS,MET,EZH2,STK11,MTOR,XIAP,UGT1A1,JAK2,SIRT3,GSTP1,CDKN1A,CDKN2A,BRCA1,PDK2,BIRC2 |
| GO:0051434 | BH3 domain binding | 3 | 6 | 0.00077 | BAX,MCL1,BCL2 |
| GO:0001216 | DNA-binding transcription activator activity | 12 | 452 | 0.0011 | EPAS1,STAT3,TP63,TP53,NOTCH1,SOX2,E2F1,STAT1,AR,TP73,NFE2L2,HIF1A |
| GO:0072341 | Modified amino acid binding | 6 | 90 | 0.0011 | NOX4,AXL,GSTM1,TYMS,HMGB1,GSTP1 |
| GO:0000987 | Cis-regulatory region sequence-specific dna binding | 15 | 701 | 0.0012 | EPAS1,STAT3,RB1,TP53,NOTCH1,EZH2,MTA1,E2F1,STAT1,MTOR,AR,TP73,NFE2L2,HIF1A,MUC1 |
| GO:0001099 | Basal rna polymerase ii transcription machinery binding | 5 | 54 | 0.0012 | ERCC1,TP53,ERCC4,ERCC5,AR |
| GO:0008094 | DNA-dependent ATPase activity | 6 | 93 | 0.0012 | RAD51C,RAD51,ERCC2,XRCC5,TOP2A,XRCC3 |
| GO:0016772 | Transferase activity, transferring phosphorus-containing groups | 17 | 875 | 0.0012 | MAPK1,CSNK2A1,CCND1,PIK3CA,IGF1R,ERBB2,EGFR,PAK1,AXL,TERT,PRKDC,MET,STK11,MTOR,JAK2,CDKN1A,PDK2 |
| GO:0004714 | Transmembrane receptor protein tyrosine kinase activity | 5 | 63 | 0.0022 | IGF1R,ERBB2,EGFR,AXL,MET |
| GO:0043560 | Insulin receptor substrate binding | 3 | 10 | 0.0022 | PIK3CA,IGF1R,JAK2 |
| GO:0001094 | TFIID-class transcription factor complex binding | 3 | 11 | 0.0028 | ERCC1,TP53,ERCC4 |
| GO:0008083 | Growth factor activity | 7 | 161 | 0.0028 | TGFB1,HGF,BMP4,GDF15,HDGF,IL6,VEGFA |
| GO:0003697 | Single-stranded dna binding | 6 | 113 | 0.0032 | ERCC1,ERCC4,HMGB1,ERCC5,RAD51,HSPD1 |
| GO:0016922 | Nuclear receptor binding | 6 | 114 | 0.0033 | STAT3,RB1,RARB,CTNNB1,KDM4C,BRCA1 |
| GO:0042826 | Histone deacetylase binding | 6 | 114 | 0.0033 | CCND1,SIRT2,TP53,MTA1,TOP2A,HIF1A |
| GO:0042162 | Telomeric dna binding | 4 | 36 | 0.0035 | APEX1,TERT,TP53BP1,XRCC5 |
| GO:0000977 | RNA polymerase II transcription regulatory region sequence-specific DNA binding | 16 | 878 | 0.0036 | EPAS1,STAT3,TP63,RB1,TP53,NOTCH1,EZH2,RARB,MTA1,STAT1,AR,TP73,NFE2L2,RUNX3,HIF1A,MUC1 |
| GO:0001228 | DNA-binding transcription activator activity, RNA polymerase II-specific | 11 | 449 | 0.004 | EPAS1,STAT3,TP63,TP53,NOTCH1,SOX2,STAT1,AR,TP73,NFE2L2,HIF1A |
| GO:0042056 | Chemoattractant activity | 4 | 41 | 0.0053 | HGF,BMP4,HMGB1,VEGFA |
| GO:0050681 | Androgen receptor binding | 4 | 44 | 0.0067 | RB1,CTNNB1,KDM4C,BRCA1 |
| GO:0003712 | Transcription coregulator activity | 12 | 571 | 0.0075 | APEX1,CCND1,RB1,EZH2,MTA1,CTNNB1,HMGB1,HDGF,TP53BP1,BRCA1,BIRC2,MUC1 |
| GO:0030234 | Enzyme regulator activity | 17 | 1044 | 0.0075 | SERPINE1,CCND1,PIK3CA,TIMP3,TP53,NOTCH1,ALDH1A1,BIRC5,STK11,XIAP,UGT1A1,IGFBP3,XRCC5,GSTP1,CDKN1A,CDKN2A,BIRC2 |
| GO:0042562 | Hormone binding | 5 | 86 | 0.0075 | IGF1R,EGFR,ALDH1A1,AR,HSPD1 |
| GO:0004713 | Protein tyrosine kinase activity | 6 | 137 | 0.0076 | IGF1R,ERBB2,EGFR,AXL,MET,JAK2 |
| GO:0035258 | Steroid hormone receptor binding | 5 | 93 | 0.0102 | STAT3,RB1,CTNNB1,KDM4C,BRCA1 |
| GO:0001102 | RNA polymerase II activating transcription factor binding | 4 | 53 | 0.0122 | RB1,CTNNB1,TP53BP1,NFE2L2 |
| GO:0003723 | RNA binding | 22 | 1649 | 0.0134 | LGALS1,APEX1,MDM2,STAT3,TP53,TERT,PRKDC,TYMS,EZH2,SOX2,HMGB1,HDGF,MKI67,S100A4,HSPD1,XRCC5,YWHAZ,TOP2A,CDKN2A,BRCA1,EIF4E,ZFP36 |
| GO:0005497 | Androgen binding | 2 | 4 | 0.016 | ALDH1A1,AR |
| GO:0008559 | ATPase-coupled xenobiotic transmembrane transporter activity | 2 | 4 | 0.016 | ABCC2,ABCC1 |
| GO:0050839 | Cell adhesion molecule binding | 11 | 538 | 0.016 | CDH1,EGFR,ADAM17,HSPA5,BSG,CTNNB1,HMGB1,STAT1,POSTN,SPP1,YWHAZ |
| GO:0004857 | Enzyme inhibitor activity | 9 | 377 | 0.0174 | SERPINE1,TIMP3,NOTCH1,BIRC5,XIAP,UGT1A1,CDKN1A,CDKN2A,BIRC2 |
| GO:0043027 | Cysteine-type endopeptidase inhibitor activity involved in apoptotic process | 3 | 25 | 0.0185 | BIRC5,XIAP,BIRC2 |
| GO:0070063 | RNA polymerase binding | 4 | 61 | 0.0189 | ERBB2,ERCC5,BRCA1,ZFP36 |
| GO:0005138 | interleukin-6 receptor binding | 2 | 5 | 0.0214 | ADAM17,IL6 |
| GO:0045569 | TRAIL binding | 2 | 5 | 0.0214 | TNFRSF10A,TNFSF10 |
| GO:0000978 | RNA polymerase II cis-regulatory region sequence-specific DNA binding | 12 | 672 | 0.0267 | EPAS1,STAT3,TP53,NOTCH1,EZH2,MTA1,STAT1,AR,TP73,NFE2L2,HIF1A,MUC1 |
| GO:0046914 | Transition metal ion binding | 16 | 1076 | 0.0267 | TNFSF10,SIRT2,MDM2,TP53,BIRC5,RARB,MTA1,S100A4,AR,BMI1,KDM4C,SIRT3,BRCA1,SOD2,BIRC2,CYP1B1 |
| GO:0031072 | Heat shock protein binding | 5 | 120 | 0.0274 | CSNK2A1,BAX,HSPA5,HIF1A,ZFP36 |
| GO:0043559 | Insulin binding | 2 | 6 | 0.0274 | IGF1R,HSPD1 |
| GO:0019956 | Chemokine binding | 3 | 30 | 0.0282 | CXCR2,HMGB1,ZFP36 |
| GO:0043548 | Phosphatidylinositol 3-kinase binding | 3 | 30 | 0.0282 | IGF1R,AXL,JAK2 |
| GO:0051721 | Protein phosphatase 2a binding | 3 | 32 | 0.033 | TP53,STAT1,BCL2 |
| GO:0019958 | C-X-C chemokine binding | 2 | 7 | 0.0337 | CXCR2,HMGB1 |
| GO:0031730 | CCR5 chemokine receptor binding | 2 | 7 | 0.0337 | STAT3,STAT1 |
| GO:0016209 | Antioxidant activity | 4 | 74 | 0.034 | PTGS2,GPX3,GSTP1,SOD2 |
| GO:0016787 | Hydrolase activity | 27 | 2419 | 0.037 | ERCC1,APEX1,HGF,MLH1,DUSP1,SIRT2,XRCC1,OGG1,ADAM17,ERCC4,USP9X,HSPA5,RAD51C,ERCC5,CASP8,ABCC2,PTEN,ABCC10,TLR4,RAD51,SIRT3,HSPD1,ERCC2,XRCC5,ABCC1,TOP2A,XRCC3 |
| GO:0001530 | Lipopolysaccharide binding | 3 | 34 | 0.0373 | HMGB1,TLR4,HSPD1 |
| GO:0016799 | Hydrolase activity, hydrolyzing n-glycosyl compounds | 3 | 34 | 0.0373 | APEX1,OGG1,TLR4 |
| GO:0043130 | Ubiquitin binding | 4 | 77 | 0.0379 | SIRT2,MDM2,TOP2A,BIRC2 |
| GO:0008270 | Zinc ion binding | 13 | 811 | 0.0384 | TNFSF10,SIRT2,MDM2,TP53,BIRC5,RARB,MTA1,AR,BMI1,KDM4C,SIRT3,BRCA1,BIRC2 |
| GO:0000014 | Single-stranded dna endodeoxyribonuclease activity | 2 | 8 | 0.0393 | XRCC1,ERCC4 |
| GO:0000405 | Bubble dna binding | 2 | 8 | 0.0393 | HMGB1,ERCC5 |
| GO:0000981 | DNA-binding transcription factor activity, RNA polymerase II-specific | 15 | 1022 | 0.0393 | EPAS1,STAT3,TP63,TP53,NOTCH1,EZH2,SOX2,RARB,E2F1,STAT1,AR,TP73,NFE2L2,RUNX3,HIF1A |
| GO:0001758 | Retinal dehydrogenase activity | 2 | 8 | 0.0393 | ALDH1A2,ALDH1A1 |
| GO:0004674 | Protein serine/threonine kinase activity | 9 | 437 | 0.0393 | MAPK1,CSNK2A1,PIK3CA,EGFR,PAK1,PRKDC,STK11,MTOR,PDK2 |
| GO:0020037 | Heme binding | 5 | 134 | 0.0393 | NOX4,NOS2,PTGS2,JAK2,CYP1B1 |
| GO:0031490 | Chromatin dna binding | 4 | 79 | 0.0393 | APEX1,STAT3,NOTCH1,EZH2 |
| GO:0032356 | Oxidized dna binding | 2 | 8 | 0.0393 | XRCC1,OGG1 |
| GO:0070851 | Growth factor receptor binding | 5 | 138 | 0.0426 | ADAM17,PTEN,JAK2,IL6,VEGFA |
| GO:0034511 | U3 snoRNA binding | 2 | 9 | 0.0449 | PRKDC,XRCC5 |

S3: Protein-pathways interactions

| name | Average shortest path length | Clustering co-efficient | Closeness Centrality | partner of multiedge node pairs | self loops | Eccentricity | stress | edge count | indegree | outdegree | Betweenness centrality | Neighborhood Connectivity | Degree layout |
| --- | --- | --- | --- | --- | --- | --- | --- | --- | --- | --- | --- | --- | --- |
| Non-small cell lung cancer | 0 | 0 | 0 | 0 | 0 | 0 | 0 | 15 | 15 | 0 | 0 | 36.7333 | 15 |
| AGE-RAGE signaling pathway in diabetic complications | 0 | 0 | 0 | 0 | 0 | 0 | 0 | 14 | 14 | 0 | 0 | 39.2857 | 14 |
| Th1 and Th2 cell differentiation | 0 | 0 | 0 | 0 | 0 | 0 | 0 | 6 | 6 | 0 | 0 | 30.3333 | 6 |
| Antifolate resistance | 0 | 0 | 0 | 0 | 0 | 0 | 0 | 5 | 5 | 0 | 0 | 22.6 | 5 |
| Chronic myeloid leukemia | 0 | 0 | 0 | 0 | 0 | 0 | 0 | 11 | 11 | 0 | 0 | 43.5455 | 11 |
| HIF-1 signaling pathway | 0 | 0 | 0 | 0 | 0 | 0 | 0 | 16 | 16 | 0 | 0 | 35.5625 | 16 |
| Colorectal cancer | 0 | 0 | 0 | 0 | 0 | 0 | 0 | 12 | 12 | 0 | 0 | 45.0833 | 12 |
| Thyroid cancer | 0 | 0 | 0 | 0 | 0 | 0 | 0 | 7 | 7 | 0 | 0 | 44.1429 | 7 |
| Renal cell carcinoma | 0 | 0 | 0 | 0 | 0 | 0 | 0 | 10 | 10 | 0 | 0 | 33.2 | 10 |
| AMPK signaling pathway | 0 | 0 | 0 | 0 | 0 | 0 | 0 | 5 | 5 | 0 | 0 | 41 | 5 |
| Ubiquitin mediated proteolysis | 0 | 0 | 0 | 0 | 0 | 0 | 0 | 4 | 4 | 0 | 0 | 14.75 | 4 |
| Cholinergic synapse | 0 | 0 | 0 | 0 | 0 | 0 | 0 | 4 | 4 | 0 | 0 | 59 | 4 |
| Osteoclast differentiation | 0 | 0 | 0 | 0 | 0 | 0 | 0 | 6 | 6 | 0 | 0 | 54.3333 | 6 |
| Metabolism of xenobiotics by cytochrome P453 | 0 | 0 | 0 | 0 | 0 | 0 | 0 | 1 | 1 | 0 | 0 | 4 | 1 |
| GnRH secretion | 0 | 0 | 0 | 0 | 0 | 0 | 0 | 3 | 3 | 0 | 0 | 60.6667 | 3 |
| Progesterone-mediated oocyte maturation | 0 | 0 | 0 | 0 | 0 | 0 | 0 | 3 | 3 | 0 | 0 | 67.6667 | 3 |
| Apelin signaling pathway | 0 | 0 | 0 | 0 | 0 | 0 | 0 | 7 | 7 | 0 | 0 | 30.5714 | 7 |
| Graft-versus-host disease | 0 | 0 | 0 | 0 | 0 | 0 | 0 | 5 | 5 | 0 | 0 | 38.2 | 5 |
| Epstein-Barr virus infection | 0 | 0 | 0 | 0 | 0 | 0 | 0 | 17 | 17 | 0 | 0 | 35.5882 | 17 |
| Adherens junction | 0 | 0 | 0 | 0 | 0 | 0 | 0 | 8 | 8 | 0 | 0 | 30.375 | 8 |
| Hematopoietic cell lineage | 0 | 0 | 0 | 0 | 0 | 0 | 0 | 3 | 3 | 0 | 0 | 36.6667 | 3 |
| Alzheimer disease | 0 | 0 | 0 | 0 | 0 | 0 | 0 | 13 | 13 | 0 | 0 | 34.6154 | 13 |
| Prolactin signaling pathway | 0 | 0 | 0 | 0 | 0 | 0 | 0 | 6 | 6 | 0 | 0 | 49.6667 | 6 |
| Choline metabolism in cancer | 0 | 0 | 0 | 0 | 0 | 0 | 0 | 5 | 5 | 0 | 0 | 54.2 | 5 |
| Legionellosis | 0 | 0 | 0 | 0 | 0 | 0 | 0 | 5 | 5 | 0 | 0 | 33 | 5 |
| Herpes simplex virus 1 infection | 0 | 0 | 0 | 0 | 0 | 0 | 0 | 14 | 14 | 0 | 0 | 39.3571 | 14 |
| Oocyte meiosis | 0 | 0 | 0 | 0 | 0 | 0 | 0 | 4 | 4 | 0 | 0 | 32 | 4 |
| p53 signaling pathway | 0 | 0 | 0 | 0 | 0 | 0 | 0 | 14 | 14 | 0 | 0 | 26.2143 | 14 |
| Hypertrophic cardiomyopathy | 0 | 0 | 0 | 0 | 0 | 0 | 0 | 3 | 3 | 0 | 0 | 45.3333 | 3 |
| ABC transporters | 0 | 0 | 0 | 0 | 0 | 0 | 0 | 3 | 3 | 0 | 0 | 2.66667 | 3 |
| Endometrial cancer | 0 | 0 | 0 | 0 | 0 | 0 | 0 | 12 | 12 | 0 | 0 | 40.25 | 12 |
| Shigellosis | 0 | 0 | 0 | 0 | 0 | 0 | 0 | 11 | 11 | 0 | 0 | 46.2727 | 11 |
| Chemokine signaling pathway | 0 | 0 | 0 | 0 | 0 | 0 | 0 | 7 | 7 | 0 | 0 | 39.4286 | 7 |
| Human immunodeficiency virus 1 infection | 0 | 0 | 0 | 0 | 0 | 0 | 0 | 11 | 11 | 0 | 0 | 44.7273 | 11 |
| Melanoma | 0 | 0 | 0 | 0 | 0 | 0 | 0 | 16 | 16 | 0 | 0 | 36.4375 | 16 |
| Non-alcoholic fatty liver disease | 0 | 0 | 0 | 0 | 0 | 0 | 0 | 8 | 8 | 0 | 0 | 44.375 | 8 |
| Epithelial cell signaling in Helicobacter pylori infection | 0 | 0 | 0 | 0 | 0 | 0 | 0 | 5 | 5 | 0 | 0 | 16.6 | 5 |
| JAK-STAT signaling pathway | 0 | 0 | 0 | 0 | 0 | 0 | 0 | 12 | 12 | 0 | 0 | 37.4167 | 12 |
| Bacterial invasion of epithelial cells | 0 | 0 | 0 | 0 | 0 | 0 | 0 | 4 | 4 | 0 | 0 | 35.5 | 4 |
| Apoptosis | 0 | 0 | 0 | 0 | 0 | 0 | 0 | 14 | 14 | 0 | 0 | 36.2143 | 14 |
| Ras signaling pathway | 0 | 0 | 0 | 0 | 0 | 0 | 0 | 9 | 9 | 0 | 0 | 37.7778 | 9 |
| Longevity regulating pathway - multiple species | 0 | 0 | 0 | 0 | 0 | 0 | 0 | 4 | 4 | 0 | 0 | 39.75 | 4 |
| Allograft rejection | 0 | 0 | 0 | 0 | 0 | 0 | 0 | 4 | 4 | 0 | 0 | 36.5 | 4 |
| FoxO signaling pathway | 0 | 0 | 0 | 0 | 0 | 0 | 0 | 15 | 15 | 0 | 0 | 35.0667 | 15 |
| Antigen processing and presentation | 0 | 0 | 0 | 0 | 0 | 0 | 0 | 3 | 3 | 0 | 0 | 32 | 3 |
| Influenza A | 0 | 0 | 0 | 0 | 0 | 0 | 0 | 14 | 14 | 0 | 0 | 38.5714 | 14 |
| Breast cancer | 0 | 0 | 0 | 0 | 0 | 0 | 0 | 16 | 16 | 0 | 0 | 37.5 | 16 |
| Rheumatoid arthritis | 0 | 0 | 0 | 0 | 0 | 0 | 0 | 6 | 6 | 0 | 0 | 35.6667 | 6 |
| Necroptosis | 0 | 0 | 0 | 0 | 0 | 0 | 0 | 17 | 17 | 0 | 0 | 24.6471 | 17 |
| Systemic lupus erythematosus | 0 | 0 | 0 | 0 | 0 | 0 | 0 | 3 | 3 | 0 | 0 | 31 | 3 |
| Fc epsilon RI signaling pathway | 0 | 0 | 0 | 0 | 0 | 0 | 0 | 3 | 3 | 0 | 0 | 78.6667 | 3 |
| PRKDC | 1 | 0 | 1 | 0 | 0 | 1 | 0 | 2 | 0 | 2 | 0 | 6 | 2 |
| Toll-like receptor signaling pathway | 0 | 0 | 0 | 0 | 0 | 0 | 0 | 8 | 8 | 0 | 0 | 46.5 | 8 |
| Amyotrophic lateral sclerosis | 0 | 0 | 0 | 0 | 0 | 0 | 0 | 8 | 8 | 0 | 0 | 32.375 | 8 |
| Acute myeloid leukemia | 0 | 0 | 0 | 0 | 0 | 0 | 0 | 5 | 5 | 0 | 0 | 58.2 | 5 |
| Oxytocin signaling pathway | 0 | 0 | 0 | 0 | 0 | 0 | 0 | 5 | 5 | 0 | 0 | 46.2 | 5 |
| Nucleotide excision repair | 0 | 0 | 0 | 0 | 0 | 0 | 0 | 4 | 4 | 0 | 0 | 1.75 | 4 |
| Fanconi anemia pathway | 0 | 0 | 0 | 0 | 0 | 0 | 0 | 5 | 5 | 0 | 0 | 4 | 5 |
| Drug metabolism - other enzymes | 0 | 0 | 0 | 0 | 0 | 0 | 0 | 3 | 3 | 0 | 0 | 8 | 3 |
| Type I diabetes mellitus | 0 | 0 | 0 | 0 | 0 | 0 | 0 | 5 | 5 | 0 | 0 | 29.8 | 5 |
| Focal adhesion | 0 | 0 | 0 | 0 | 0 | 0 | 0 | 16 | 16 | 0 | 0 | 31.0625 | 16 |
| Human T-cell leukemia virus 1 infection | 0 | 0 | 0 | 0 | 0 | 0 | 0 | 16 | 16 | 0 | 0 | 37.625 | 16 |
| Ovarian steroidogenesis | 0 | 0 | 0 | 0 | 0 | 0 | 0 | 3 | 3 | 0 | 0 | 15.6667 | 3 |
| Insulin resistance | 0 | 0 | 0 | 0 | 0 | 0 | 0 | 6 | 6 | 0 | 0 | 47.5 | 6 |
| ErbB signaling pathway | 0 | 0 | 0 | 0 | 0 | 0 | 0 | 7 | 7 | 0 | 0 | 47.8571 | 7 |
| MicroRNAs in cancer | 0 | 0 | 0 | 0 | 0 | 0 | 0 | 27 | 27 | 0 | 0 | 24.6296 | 27 |
| PD-L1 expression and PD-1 checkpoint pathway in cancer | 0 | 0 | 0 | 0 | 0 | 0 | 0 | 14 | 14 | 0 | 0 | 31.5 | 14 |
| Protein processing in endoplasmic reticulum | 0 | 0 | 0 | 0 | 0 | 0 | 0 | 4 | 4 | 0 | 0 | 24 | 4 |
| VEGF signaling pathway | 0 | 0 | 0 | 0 | 0 | 0 | 0 | 4 | 4 | 0 | 0 | 53.25 | 4 |
| Cytokine-cytokine receptor interaction | 0 | 0 | 0 | 0 | 0 | 0 | 0 | 11 | 11 | 0 | 0 | 23.1818 | 11 |
| Rap1 signaling pathway | 0 | 0 | 0 | 0 | 0 | 0 | 0 | 9 | 9 | 0 | 0 | 37.2222 | 9 |
| Pancreatic cancer | 0 | 0 | 0 | 0 | 0 | 0 | 0 | 16 | 16 | 0 | 0 | 39.75 | 16 |
| IL-17 signaling pathway | 0 | 0 | 0 | 0 | 0 | 0 | 0 | 6 | 6 | 0 | 0 | 45.6667 | 6 |
| Tuberculosis | 0 | 0 | 0 | 0 | 0 | 0 | 0 | 13 | 13 | 0 | 0 | 35.9231 | 13 |
| Hepatitis C | 0 | 0 | 0 | 0 | 0 | 0 | 0 | 18 | 18 | 0 | 0 | 39.6111 | 18 |
| Natural killer cell mediated cytotoxicity | 0 | 0 | 0 | 0 | 0 | 0 | 0 | 9 | 9 | 0 | 0 | 39.4444 | 9 |
| TGF-beta signaling pathway | 0 | 0 | 0 | 0 | 0 | 0 | 0 | 5 | 5 | 0 | 0 | 44.2 | 5 |
| MAPK signaling pathway | 0 | 0 | 0 | 0 | 0 | 0 | 0 | 14 | 14 | 0 | 0 | 31.8571 | 14 |
| Estrogen signaling pathway | 0 | 0 | 0 | 0 | 0 | 0 | 0 | 4 | 4 | 0 | 0 | 63.5 | 4 |
| Chemical carcinogenesis | 0 | 0 | 0 | 0 | 0 | 0 | 0 | 5 | 5 | 0 | 0 | 8.8 | 5 |
| Drug metabolism - cytochrome P451 | 0 | 0 | 0 | 0 | 0 | 0 | 0 | 1 | 1 | 0 | 0 | 5 | 1 |
| Longevity regulating pathway | 0 | 0 | 0 | 0 | 0 | 0 | 0 | 7 | 7 | 0 | 0 | 37.5714 | 7 |
| African trypanosomiasis | 0 | 0 | 0 | 0 | 0 | 0 | 0 | 5 | 5 | 0 | 0 | 38.2 | 5 |
| Viral carcinogenesis | 0 | 0 | 0 | 0 | 0 | 0 | 0 | 12 | 12 | 0 | 0 | 40.8333 | 12 |
| Metabolism of xenobiotics by cytochrome P450 | 0 | 0 | 0 | 0 | 0 | 0 | 0 | 1 | 1 | 0 | 0 | 9 | 1 |
| PI3K-Akt signaling pathway | 0 | 0 | 0 | 0 | 0 | 0 | 0 | 24 | 24 | 0 | 0 | 30.4167 | 24 |
| Chagas disease | 0 | 0 | 0 | 0 | 0 | 0 | 0 | 12 | 12 | 0 | 0 | 39.5833 | 12 |
| Malaria | 0 | 0 | 0 | 0 | 0 | 0 | 0 | 7 | 7 | 0 | 0 | 32.4286 | 7 |
| Endocrine resistance | 0 | 0 | 0 | 0 | 0 | 0 | 0 | 16 | 16 | 0 | 0 | 39 | 16 |
| Fluid shear stress and atherosclerosis | 0 | 0 | 0 | 0 | 0 | 0 | 0 | 12 | 12 | 0 | 0 | 28.75 | 12 |
| Kaposi sarcoma-associated herpesvirus infection | 0 | 0 | 0 | 0 | 0 | 0 | 0 | 20 | 20 | 0 | 0 | 35.45 | 20 |
| Drug metabolism - cytochrome P450 | 0 | 0 | 0 | 0 | 0 | 0 | 0 | 1 | 1 | 0 | 0 | 9 | 1 |
| Human papillomavirus infection | 0 | 0 | 0 | 0 | 0 | 0 | 0 | 23 | 23 | 0 | 0 | 34.4348 | 23 |
| Glutathione metabolism | 0 | 0 | 0 | 0 | 0 | 0 | 0 | 3 | 3 | 0 | 0 | 7.33333 | 3 |
| Base excision repair | 0 | 0 | 0 | 0 | 0 | 0 | 0 | 4 | 4 | 0 | 0 | 1.5 | 4 |
| Platinum drug resistance | 0 | 0 | 0 | 0 | 0 | 0 | 0 | 21 | 21 | 0 | 0 | 27 | 21 |
| Autophagy - animal | 0 | 0 | 0 | 0 | 0 | 0 | 0 | 9 | 9 | 0 | 0 | 36.5556 | 9 |
| Pathogenic Escherichia coli infection | 0 | 0 | 0 | 0 | 0 | 0 | 0 | 11 | 11 | 0 | 0 | 35.3636 | 11 |
| Metabolism of xenobiotics by cytochrome P451 | 0 | 0 | 0 | 0 | 0 | 0 | 0 | 1 | 1 | 0 | 0 | 5 | 1 |
| Nicotinate and nicotinamide metabolism | 0 | 0 | 0 | 0 | 0 | 0 | 0 | 2 | 2 | 0 | 0 | 1.5 | 2 |
| Prion disease | 0 | 0 | 0 | 0 | 0 | 0 | 0 | 8 | 8 | 0 | 0 | 43.75 | 8 |
| Non-homologous end-joining | 0 | 0 | 0 | 0 | 0 | 0 | 0 | 2 | 2 | 0 | 0 | 1.5 | 2 |
| Wnt signaling pathway | 0 | 0 | 0 | 0 | 0 | 0 | 0 | 4 | 4 | 0 | 0 | 31.5 | 4 |
| Toxoplasmosis | 0 | 0 | 0 | 0 | 0 | 0 | 0 | 13 | 13 | 0 | 0 | 32.9231 | 13 |
| Inflammatory bowel disease | 0 | 0 | 0 | 0 | 0 | 0 | 0 | 7 | 7 | 0 | 0 | 35.7143 | 7 |
| T cell receptor signaling pathway | 0 | 0 | 0 | 0 | 0 | 0 | 0 | 6 | 6 | 0 | 0 | 47.3333 | 6 |
| Neurotrophin signaling pathway | 0 | 0 | 0 | 0 | 0 | 0 | 0 | 7 | 7 | 0 | 0 | 48.8571 | 7 |
| Human cytomegalovirus infection | 0 | 0 | 0 | 0 | 0 | 0 | 0 | 22 | 22 | 0 | 0 | 37.2727 | 22 |
| Apoptosis - multiple species | 0 | 0 | 0 | 0 | 0 | 0 | 0 | 5 | 5 | 0 | 0 | 29 | 5 |
| Transcriptional misregulation in cancer | 0 | 0 | 0 | 0 | 0 | 0 | 0 | 10 | 10 | 0 | 0 | 27.7 | 10 |
| Salmonella infection | 0 | 0 | 0 | 0 | 0 | 0 | 0 | 13 | 13 | 0 | 0 | 38.3846 | 13 |
| Adipocytokine signaling pathway | 0 | 0 | 0 | 0 | 0 | 0 | 0 | 5 | 5 | 0 | 0 | 32.2 | 5 |
| TNF signaling pathway | 0 | 0 | 0 | 0 | 0 | 0 | 0 | 8 | 8 | 0 | 0 | 46.5 | 8 |
| Gastric cancer | 0 | 0 | 0 | 0 | 0 | 0 | 0 | 20 | 20 | 0 | 0 | 33.2 | 20 |
| Cellular senescence | 0 | 0 | 0 | 0 | 0 | 0 | 0 | 15 | 15 | 0 | 0 | 36.8667 | 15 |
| Central carbon metabolism in cancer | 0 | 0 | 0 | 0 | 0 | 0 | 0 | 10 | 10 | 0 | 0 | 38.5 | 10 |
| NF-kappa B signaling pathway | 0 | 0 | 0 | 0 | 0 | 0 | 0 | 7 | 7 | 0 | 0 | 25.1429 | 7 |
| Proteoglycans in cancer | 0 | 0 | 0 | 0 | 0 | 0 | 0 | 24 | 24 | 0 | 0 | 32.4583 | 24 |
| EGFR tyrosine kinase inhibitor resistance | 0 | 0 | 0 | 0 | 0 | 0 | 0 | 16 | 16 | 0 | 0 | 35.4375 | 16 |
| Small cell lung cancer | 0 | 0 | 0 | 0 | 0 | 0 | 0 | 14 | 14 | 0 | 0 | 31.2143 | 14 |
| Yersinia infection | 0 | 0 | 0 | 0 | 0 | 0 | 0 | 5 | 5 | 0 | 0 | 61.4 | 5 |
| mTOR signaling pathway | 0 | 0 | 0 | 0 | 0 | 0 | 0 | 7 | 7 | 0 | 0 | 48 | 7 |
| Prostate cancer | 0 | 0 | 0 | 0 | 0 | 0 | 0 | 17 | 17 | 0 | 0 | 36 | 17 |
| Hippo signaling pathway | 0 | 0 | 0 | 0 | 0 | 0 | 0 | 10 | 10 | 0 | 0 | 15.5 | 10 |
| Parathyroid hormone synthesis, secretion and action | 0 | 0 | 0 | 0 | 0 | 0 | 0 | 4 | 4 | 0 | 0 | 52.5 | 4 |
| Metabolism of xenobiotics by cytochrome P452 | 0 | 0 | 0 | 0 | 0 | 0 | 0 | 1 | 1 | 0 | 0 | 10 | 1 |
| Cell cycle | 0 | 0 | 0 | 0 | 0 | 0 | 0 | 10 | 10 | 0 | 0 | 26.4 | 10 |
| Hepatitis B | 0 | 0 | 0 | 0 | 0 | 0 | 0 | 19 | 19 | 0 | 0 | 38.6842 | 19 |
| Th17 cell differentiation | 0 | 0 | 0 | 0 | 0 | 0 | 0 | 9 | 9 | 0 | 0 | 36.8889 | 9 |
| Pertussis | 0 | 0 | 0 | 0 | 0 | 0 | 0 | 5 | 5 | 0 | 0 | 47 | 5 |
| Viral protein interaction with cytokine and cytokine receptor | 0 | 0 | 0 | 0 | 0 | 0 | 0 | 5 | 5 | 0 | 0 | 25.8 | 5 |
| Measles | 0 | 0 | 0 | 0 | 0 | 0 | 0 | 14 | 14 | 0 | 0 | 34.9286 | 14 |
| Leishmaniasis | 0 | 0 | 0 | 0 | 0 | 0 | 0 | 9 | 9 | 0 | 0 | 35.3333 | 9 |
| Huntington disease | 0 | 0 | 0 | 0 | 0 | 0 | 0 | 6 | 6 | 0 | 0 | 29.6667 | 6 |
| Sphingolipid signaling pathway | 0 | 0 | 0 | 0 | 0 | 0 | 0 | 8 | 8 | 0 | 0 | 49.75 | 8 |
| Glioma | 0 | 0 | 0 | 0 | 0 | 0 | 0 | 14 | 14 | 0 | 0 | 41.5 | 14 |
| Bladder cancer | 0 | 0 | 0 | 0 | 0 | 0 | 0 | 12 | 12 | 0 | 0 | 33.9167 | 12 |
| Thyroid hormone signaling pathway | 0 | 0 | 0 | 0 | 0 | 0 | 0 | 11 | 11 | 0 | 0 | 37.9091 | 11 |
| Drug metabolism - cytochrome P452 | 0 | 0 | 0 | 0 | 0 | 0 | 0 | 1 | 1 | 0 | 0 | 10 | 1 |
| Amoebiasis | 0 | 0 | 0 | 0 | 0 | 0 | 0 | 7 | 7 | 0 | 0 | 41.5714 | 7 |
| NOD-like receptor signaling pathway | 0 | 0 | 0 | 0 | 0 | 0 | 0 | 10 | 10 | 0 | 0 | 34.8 | 10 |
| Basal cell carcinoma | 0 | 0 | 0 | 0 | 0 | 0 | 0 | 5 | 5 | 0 | 0 | 34.4 | 5 |
| Hepatocellular carcinoma | 0 | 0 | 0 | 0 | 0 | 0 | 0 | 21 | 21 | 0 | 0 | 32.2381 | 21 |
| Mitophagy - animal | 0 | 0 | 0 | 0 | 0 | 0 | 0 | 4 | 4 | 0 | 0 | 23.5 | 4 |
| Type II diabetes mellitus | 0 | 0 | 0 | 0 | 0 | 0 | 0 | 4 | 4 | 0 | 0 | 69.75 | 4 |
| Phospholipase D signaling pathway | 0 | 0 | 0 | 0 | 0 | 0 | 0 | 5 | 5 | 0 | 0 | 53 | 5 |
| C-type lectin receptor signaling pathway | 0 | 0 | 0 | 0 | 0 | 0 | 0 | 9 | 9 | 0 | 0 | 43.8889 | 9 |
| Cushing syndrome | 0 | 0 | 0 | 0 | 0 | 0 | 0 | 8 | 8 | 0 | 0 | 38.5 | 8 |
| Signaling pathways regulating pluripotency of stem cells | 0 | 0 | 0 | 0 | 0 | 0 | 0 | 9 | 9 | 0 | 0 | 32.5556 | 9 |
| Relaxin signaling pathway | 0 | 0 | 0 | 0 | 0 | 0 | 0 | 6 | 6 | 0 | 0 | 46.8333 | 6 |
| Homologous recombination | 0 | 0 | 0 | 0 | 0 | 0 | 0 | 3 | 3 | 0 | 0 | 3.33333 | 3 |
| Growth hormone synthesis, secretion and action | 0 | 0 | 0 | 0 | 0 | 0 | 0 | 7 | 7 | 0 | 0 | 43.1429 | 7 |
| Retinol metabolism | 0 | 0 | 0 | 0 | 0 | 0 | 0 | 3 | 3 | 0 | 0 | 2.33333 | 3 |
| Pathways in cancer | 0 | 0 | 0 | 0 | 0 | 0 | 0 | 45 | 45 | 0 | 0 | 25.1556 | 45 |
| Fc gamma R-mediated phagocytosis | 0 | 0 | 0 | 0 | 0 | 0 | 0 | 3 | 3 | 0 | 0 | 63.6667 | 3 |
| Parkinson disease | 0 | 0 | 0 | 0 | 0 | 0 | 0 | 5 | 5 | 0 | 0 | 22 | 5 |

S4: Cellular Components

| #term ID | term description | observed gene count | background gene count | false discovery rate | matching proteins in your network (labels) |
| --- | --- | --- | --- | --- | --- |
| GO:0070013 | Intracellular organelle lumen | 85 | 5857 | 7.41E-17 | ERCC1,MAPK1,LGALS1,APEX1,CSNK2A1,TGFB1,HGF,SERPINE1,CCND1,MLH1,BMP4,SIRT2,MDM2,XRCC1,NOX4,EPAS1,STAT3,TP63,TIMP3,RB1,TP53,EGFR,NOTCH1,PAK1,BIRC5,MGMT,OGG1,TERT,ERCC4,PRKDC,TYMS,CXCR2,EZH2,SOX2,HSPA5,STK11,NOS2,RARB,MTA1,RAD51C,CTNNB1,HMGB1,E2F1,ERCC5,FAS,CASP8,STAT1,MTOR,PTGS2,FASLG,HDGF,MKI67,MCL1,XIAP,PTEN,AR,BMI1,TP73,IGFBP3,KDM4C,JAK2,TP53BP1,RAD51,SIRT3,HSPD1,ERCC2,XRCC5,SPP1,YWHAZ,NFE2L2,BCL2,GSTP1,RUNX3,CDKN1A,IL6,TOP2A,CDKN2A,BRCA1,PDK2,H2AFX,HIF1A,SOD2,XRCC3,VEGFA,MUC1 |
| GO:0032991 | Protein-containing complex | 77 | 5073 | 9.57E-16 | ERCC1,MAPK1,APEX1,CSNK2A1,CCND1,MLH1,SIRT2,MDM2,CDH1,XRCC1,NOX4,EPAS1,PIK3CA,STAT3,TP63,RB1,IGF1R,TP53,ERBB2,EGFR,NOTCH1,PAK1,BAX,AXL,BIRC5,OGG1,TERT,ERCC4,PRKDC,MET,EZH2,SOX2,HSPA5,RARB,MTA1,RAD51C,CTNNB1,HMGB1,E2F1,ERCC5,FAS,CASP8,STAT1,MTOR,PTGS2,HDGF,MCL1,UGT1A1,TLR4,AR,BMI1,IGFBP3,KDM4C,TP53BP1,RAD51,SIRT3,HSPD1,ERCC2,XRCC5,NFE2L2,BCL2,GSTP1,RUNX3,CDKN1A,IL6,CD44,TNF,TOP2A,CDKN2A,BRCA1,PDK2,EIF4E,H2AFX,HIF1A,XRCC3,ZFP36,BIRC2 |
| GO:0005654 | Nucleoplasm | 64 | 3973 | 6.10E-13 | ERCC1,MAPK1,APEX1,CSNK2A1,CCND1,MLH1,MDM2,XRCC1,NOX4,EPAS1,STAT3,TP63,RB1,TP53,NOTCH1,PAK1,BIRC5,MGMT,OGG1,TERT,ERCC4,PRKDC,CXCR2,EZH2,SOX2,STK11,RARB,MTA1,RAD51C,CTNNB1,HMGB1,E2F1,ERCC5,FAS,CASP8,STAT1,MTOR,HDGF,MKI67,MCL1,XIAP,PTEN,AR,BMI1,TP73,KDM4C,JAK2,TP53BP1,RAD51,SIRT3,ERCC2,XRCC5,YWHAZ,NFE2L2,BCL2,RUNX3,CDKN1A,TOP2A,CDKN2A,BRCA1,PDK2,H2AFX,HIF1A,XRCC3 |
| GO:0005694 | Chromosome | 42 | 1712 | 6.10E-13 | ERCC1,APEX1,CSNK2A1,MLH1,SIRT2,XRCC1,EPAS1,STAT3,TP63,RB1,TP53,PAK1,BIRC5,TERT,ERCC4,PRKDC,EZH2,SOX2,RARB,MTA1,RAD51C,CTNNB1,HMGB1,E2F1,STAT1,MKI67,AR,TP73,KDM4C,TP53BP1,RAD51,XRCC5,NFE2L2,RUNX3,TOP2A,CDKN2A,BRCA1,H2AFX,HIF1A,XRCC3,BIRC2,MUC1 |
| GO:0000228 | Nuclear chromosome | 36 | 1256 | 9.85E-13 | ERCC1,APEX1,CSNK2A1,MLH1,SIRT2,XRCC1,EPAS1,STAT3,TP63,RB1,TP53,BIRC5,TERT,ERCC4,PRKDC,EZH2,SOX2,RARB,MTA1,CTNNB1,HMGB1,E2F1,STAT1,AR,TP73,KDM4C,TP53BP1,RAD51,XRCC5,NFE2L2,RUNX3,BRCA1,H2AFX,HIF1A,XRCC3,MUC1 |
| GO:0043227 | Membrane-bounded organelle | 112 | 12427 | 6.13E-12 | ERCC1,MAPK1,LGALS1,APEX1,CSNK2A1,TNFRSF10A,TGFB1,HGF,SERPINE1,CCND1,MLH1,DUSP1,TNFSF10,BMP4,SIRT2,GDF15,MDM2,CDH1,XRCC1,NOX4,EPAS1,STAT3,TP63,TIMP3,RB1,IGF1R,TP53,ERBB2,EGFR,NOTCH1,PAK1,BAX,ALDH1A1,AXL,BIRC5,MGMT,OGG1,TERT,ERCC4,PRKDC,TYMS,USP9X,CXCR2,EZH2,SOX2,HSPA5,STK11,NOS2,RARB,MTA1,BSG,RAD51C,CTNNB1,HMGB1,E2F1,ERCC5,FAS,CASP8,STAT1,MTOR,PTGS2,FASLG,HDGF,MKI67,S100A4,MCL1,XIAP,PTEN,ABCC10,UGT1A1,TLR4,AR,BMI1,TP73,POSTN,IGFBP3,KDM4C,CD274,JAK2,TP53BP1,RAD51,SIRT3,GPX3,HSPD1,ERCC2,XRCC5,SPP1,YWHAZ,NFE2L2,BCL2,GSTP1,ABCC1,RUNX3,CDKN1A,IL6,CD44,TNF,TOP2A,CDKN2A,BRCA1,PDK2,EIF4E,H2AFX,HIF1A,SLC16A1,SOD2,XRCC3,ZFP36,BIRC2,CYP1B1,VEGFA,MUC1 |
| GO:0031981 | Nuclear lumen | 67 | 4733 | 2.81E-11 | ERCC1,MAPK1,APEX1,CSNK2A1,CCND1,MLH1,SIRT2,MDM2,XRCC1,NOX4,EPAS1,STAT3,TP63,RB1,TP53,NOTCH1,PAK1,BIRC5,MGMT,OGG1,TERT,ERCC4,PRKDC,TYMS,CXCR2,EZH2,SOX2,STK11,RARB,MTA1,RAD51C,CTNNB1,HMGB1,E2F1,ERCC5,FAS,CASP8,STAT1,MTOR,HDGF,MKI67,MCL1,XIAP,PTEN,AR,BMI1,TP73,KDM4C,JAK2,TP53BP1,RAD51,SIRT3,ERCC2,XRCC5,YWHAZ,NFE2L2,BCL2,RUNX3,CDKN1A,TOP2A,CDKN2A,BRCA1,PDK2,H2AFX,HIF1A,XRCC3,MUC1 |
| GO:0005634 | Nucleus | 85 | 7390 | 4.32E-11 | ERCC1,MAPK1,LGALS1,APEX1,CSNK2A1,TGFB1,CCND1,MLH1,DUSP1,SIRT2,GDF15,MDM2,XRCC1,NOX4,EPAS1,STAT3,TP63,TIMP3,RB1,TP53,ERBB2,EGFR,NOTCH1,PAK1,BAX,BIRC5,MGMT,OGG1,TERT,ERCC4,PRKDC,TYMS,USP9X,CXCR2,EZH2,SOX2,HSPA5,STK11,NOS2,RARB,MTA1,RAD51C,CTNNB1,HMGB1,E2F1,ERCC5,FAS,CASP8,STAT1,MTOR,PTGS2,FASLG,HDGF,MKI67,S100A4,MCL1,XIAP,PTEN,AR,BMI1,TP73,IGFBP3,KDM4C,JAK2,TP53BP1,RAD51,SIRT3,ERCC2,XRCC5,YWHAZ,NFE2L2,BCL2,GSTP1,RUNX3,CDKN1A,TOP2A,CDKN2A,BRCA1,PDK2,H2AFX,HIF1A,XRCC3,ZFP36,BIRC2,MUC1 |
| GO:0043231 | Intracellular membrane-bounded organelle | 102 | 10761 | 4.57E-10 | ERCC1,MAPK1,LGALS1,APEX1,CSNK2A1,TNFRSF10A,TGFB1,CCND1,MLH1,DUSP1,BMP4,SIRT2,GDF15,MDM2,CDH1,XRCC1,NOX4,EPAS1,STAT3,TP63,TIMP3,RB1,IGF1R,TP53,ERBB2,EGFR,NOTCH1,PAK1,BAX,AXL,BIRC5,MGMT,OGG1,TERT,ERCC4,PRKDC,TYMS,USP9X,CXCR2,EZH2,SOX2,HSPA5,STK11,NOS2,RARB,MTA1,BSG,RAD51C,CTNNB1,HMGB1,E2F1,ERCC5,FAS,CASP8,STAT1,MTOR,PTGS2,FASLG,HDGF,MKI67,S100A4,MCL1,XIAP,PTEN,ABCC10,UGT1A1,TLR4,AR,BMI1,TP73,POSTN,IGFBP3,KDM4C,JAK2,TP53BP1,RAD51,SIRT3,HSPD1,ERCC2,XRCC5,SPP1,YWHAZ,NFE2L2,BCL2,GSTP1,RUNX3,CDKN1A,IL6,CD44,TOP2A,CDKN2A,BRCA1,PDK2,H2AFX,HIF1A,SLC16A1,SOD2,XRCC3,ZFP36,BIRC2,CYP1B1,MUC1 |
| GO:0043226 | Organelle | 113 | 13515 | 2.04E-09 | ERCC1,MAPK1,LGALS1,APEX1,CSNK2A1,TNFRSF10A,TGFB1,HGF,SERPINE1,CCND1,MLH1,DUSP1,TNFSF10,BMP4,SIRT2,GDF15,MDM2,CDH1,XRCC1,NOX4,EPAS1,STAT3,TP63,TIMP3,RB1,IGF1R,TP53,ERBB2,EGFR,NOTCH1,PAK1,BAX,ALDH1A1,AXL,BIRC5,MGMT,OGG1,TERT,ADAM17,ERCC4,PRKDC,TYMS,USP9X,CXCR2,EZH2,SOX2,HSPA5,STK11,NOS2,RARB,MTA1,BSG,RAD51C,CTNNB1,HMGB1,E2F1,ERCC5,FAS,CASP8,STAT1,MTOR,PTGS2,FASLG,HDGF,MKI67,S100A4,MCL1,XIAP,PTEN,ABCC10,UGT1A1,TLR4,AR,BMI1,TP73,POSTN,IGFBP3,KDM4C,CD274,JAK2,TP53BP1,RAD51,SIRT3,GPX3,HSPD1,ERCC2,XRCC5,SPP1,YWHAZ,NFE2L2,BCL2,GSTP1,ABCC1,RUNX3,CDKN1A,IL6,CD44,TNF,TOP2A,CDKN2A,BRCA1,PDK2,EIF4E,H2AFX,HIF1A,SLC16A1,SOD2,XRCC3,ZFP36,BIRC2,CYP1B1,VEGFA,MUC1 |
| GO:0043229 | Intracellular organelle | 109 | 12528 | 2.12E-09 | ERCC1,MAPK1,LGALS1,APEX1,CSNK2A1,TNFRSF10A,TGFB1,HGF,SERPINE1,CCND1,MLH1,DUSP1,BMP4,SIRT2,GDF15,MDM2,CDH1,XRCC1,NOX4,EPAS1,STAT3,TP63,TIMP3,RB1,IGF1R,TP53,ERBB2,EGFR,NOTCH1,PAK1,BAX,AXL,BIRC5,MGMT,OGG1,TERT,ADAM17,ERCC4,PRKDC,TYMS,USP9X,CXCR2,EZH2,SOX2,HSPA5,STK11,NOS2,RARB,MTA1,BSG,RAD51C,CTNNB1,HMGB1,E2F1,ERCC5,FAS,CASP8,STAT1,MTOR,PTGS2,FASLG,HDGF,MKI67,S100A4,MCL1,XIAP,PTEN,ABCC10,UGT1A1,TLR4,AR,BMI1,TP73,POSTN,IGFBP3,KDM4C,CD274,JAK2,TP53BP1,RAD51,SIRT3,HSPD1,ERCC2,XRCC5,SPP1,YWHAZ,NFE2L2,BCL2,GSTP1,RUNX3,CDKN1A,IL6,CD44,TNF,TOP2A,CDKN2A,BRCA1,PDK2,EIF4E,H2AFX,HIF1A,SLC16A1,SOD2,XRCC3,ZFP36,BIRC2,CYP1B1,VEGFA,MUC1 |
| GO:0000781 | Chromosome, telomeric region | 12 | 134 | 1.13E-08 | ERCC1,APEX1,SIRT2,XRCC1,TERT,ERCC4,PRKDC,EZH2,TP53BP1,RAD51,XRCC5,XRCC3 |
| GO:1990391 | DNA repair complex | 8 | 34 | 1.69E-08 | ERCC1,MLH1,XRCC1,ERCC4,PRKDC,ERCC5,TP53BP1,XRCC5 |
| GO:0005667 | Transcription regulator complex | 18 | 431 | 2.71E-08 | APEX1,CSNK2A1,CCND1,EPAS1,STAT3,RB1,TP53,PRKDC,SOX2,RARB,MTA1,CTNNB1,HMGB1,E2F1,HDGF,ERCC2,RUNX3,HIF1A |
| GO:0005737 | Cytoplasm | 101 | 11428 | 1.30E-07 | ERCC1,MAPK1,LGALS1,APEX1,CSNK2A1,TNFRSF10A,TGFB1,HGF,SERPINE1,CCND1,DUSP1,BMP4,SIRT2,ALDH1A2,GDF15,MDM2,CDH1,NOX4,EPAS1,PIK3CA,STAT3,TP63,TIMP3,TP53,ERBB2,EGFR,NOTCH1,PAK1,BAX,ALDH1A1,BIRC5,OGG1,TERT,ADAM17,GSTM1,PRKDC,TYMS,USP9X,CXCR2,EZH2,SOX2,HSPA5,STK11,NOS2,RARB,MTA1,BSG,RAD51C,CTNNB1,HMGB1,E2F1,FAS,CASP8,STAT1,MTOR,PTGS2,FASLG,HDGF,S100A4,MCL1,XIAP,PTEN,ABCC10,UGT1A1,TLR4,AR,BMI1,TP73,POSTN,IGFBP3,CD274,JAK2,TP53BP1,RAD51,SIRT3,HSPD1,ERCC2,XRCC5,SPP1,YWHAZ,NFE2L2,BCL2,GSTP1,RUNX3,CDKN1A,IL6,CD44,TNF,TOP2A,CDKN2A,BRCA1,PDK2,EIF4E,HIF1A,SOD2,XRCC3,ZFP36,BIRC2,CYP1B1,VEGFA,MUC1 |
| GO:0000784 | Nuclear chromosome, telomeric region | 10 | 102 | 1.53E-07 | ERCC1,APEX1,XRCC1,TERT,ERCC4,PRKDC,TP53BP1,RAD51,XRCC5,XRCC3 |
| GO:0005739 | Mitochondrion | 32 | 1611 | 1.98E-07 | MAPK1,APEX1,SIRT2,NOX4,STAT3,TP63,TP53,BAX,OGG1,TERT,TYMS,HSPA5,STK11,BSG,RAD51C,E2F1,CASP8,MTOR,MCL1,PTEN,TP73,RAD51,SIRT3,HSPD1,YWHAZ,BCL2,GSTP1,CDKN2A,PDK2,SOD2,XRCC3,CYP1B1 |
| GO:0045121 | Membrane raft | 15 | 324 | 2.00E-07 | MAPK1,TNFRSF10A,CDH1,EGFR,ADAM17,BSG,CTNNB1,FAS,CASP8,PTGS2,FASLG,JAK2,HSPD1,TNF,BIRC2 |
| GO:0005622 | Intracellular | 113 | 14276 | 2.14E-07 | ERCC1,MAPK1,LGALS1,APEX1,CSNK2A1,TNFRSF10A,TGFB1,HGF,SERPINE1,CCND1,MLH1,DUSP1,BMP4,SIRT2,ALDH1A2,GDF15,MDM2,CDH1,XRCC1,NOX4,EPAS1,PIK3CA,STAT3,TP63,TIMP3,RB1,IGF1R,TP53,ERBB2,EGFR,NOTCH1,PAK1,BAX,ALDH1A1,AXL,BIRC5,MGMT,OGG1,TERT,ADAM17,ERCC4,GSTM1,PRKDC,TYMS,USP9X,CXCR2,EZH2,SOX2,HSPA5,STK11,NOS2,RARB,MTA1,BSG,RAD51C,CTNNB1,HMGB1,E2F1,ERCC5,FAS,CASP8,STAT1,MTOR,PTGS2,FASLG,HDGF,MKI67,S100A4,MCL1,XIAP,PTEN,ABCC10,UGT1A1,TLR4,AR,BMI1,TP73,POSTN,IGFBP3,KDM4C,CD274,JAK2,TP53BP1,RAD51,SIRT3,HSPD1,ERCC2,XRCC5,SPP1,YWHAZ,NFE2L2,BCL2,GSTP1,RUNX3,CDKN1A,IL6,CD44,TNF,TOP2A,CDKN2A,BRCA1,PDK2,EIF4E,H2AFX,HIF1A,SLC16A1,SOD2,XRCC3,ZFP36,BIRC2,CYP1B1,VEGFA,MUC1 |
| GO:0000790 | Nuclear chromatin | 25 | 1048 | 3.98E-07 | CSNK2A1,SIRT2,XRCC1,EPAS1,STAT3,TP63,RB1,TP53,EZH2,SOX2,RARB,MTA1,CTNNB1,HMGB1,E2F1,STAT1,AR,TP73,KDM4C,RAD51,NFE2L2,RUNX3,H2AFX,HIF1A,MUC1 |
| GO:0098687 | Chromosomal region | 14 | 318 | 1.04E-06 | ERCC1,APEX1,SIRT2,XRCC1,BIRC5,TERT,ERCC4,PRKDC,EZH2,KDM4C,TP53BP1,RAD51,XRCC5,XRCC3 |
| GO:0000785 | Chromatin | 26 | 1220 | 1.61E-06 | CSNK2A1,SIRT2,XRCC1,EPAS1,STAT3,TP63,RB1,TP53,EZH2,SOX2,RARB,MTA1,CTNNB1,HMGB1,E2F1,STAT1,AR,TP73,KDM4C,RAD51,NFE2L2,RUNX3,CDKN2A,H2AFX,HIF1A,MUC1 |
| GO:0048471 | Perinuclear region of cytoplasm | 20 | 727 | 1.61E-06 | APEX1,SIRT2,ALDH1A2,CDH1,NOX4,ERBB2,EGFR,NOS2,RAD51C,CTNNB1,STAT1,FASLG,S100A4,UGT1A1,TLR4,RAD51,SPP1,CDKN1A,EIF4E,XRCC3 |
| GO:0043232 | Intracellular non-membrane-bounded organelle | 58 | 4880 | 1.85E-06 | ERCC1,MAPK1,APEX1,CSNK2A1,MLH1,SIRT2,MDM2,CDH1,XRCC1,NOX4,EPAS1,STAT3,TP63,RB1,TP53,PAK1,AXL,BIRC5,TERT,ADAM17,ERCC4,PRKDC,TYMS,CXCR2,EZH2,SOX2,NOS2,RARB,MTA1,RAD51C,CTNNB1,HMGB1,E2F1,CASP8,STAT1,MKI67,AR,TP73,KDM4C,JAK2,TP53BP1,RAD51,ERCC2,XRCC5,NFE2L2,RUNX3,CDKN1A,TOP2A,CDKN2A,BRCA1,EIF4E,H2AFX,HIF1A,SLC16A1,XRCC3,ZFP36,BIRC2,MUC1 |
| GO:0009986 | Cell surface | 21 | 824 | 2.15E-06 | LGALS1,TNFRSF10A,TGFB1,EGFR,NOTCH1,AXL,ADAM17,MET,CXCR2,HSPA5,PDCD1,HMGB1,FAS,FASLG,ABCC2,TLR4,CD274,HSPD1,CD44,TNF,VEGFA |
| GO:0005829 | Cytosol | 59 | 5193 | 6.23E-06 | MAPK1,LGALS1,CSNK2A1,TNFRSF10A,CCND1,SIRT2,ALDH1A2,MDM2,EPAS1,PIK3CA,STAT3,TP53,ERBB2,NOTCH1,PAK1,BAX,ALDH1A1,BIRC5,TERT,ADAM17,GSTM1,PRKDC,TYMS,USP9X,SOX2,HSPA5,STK11,NOS2,MTA1,RAD51C,CTNNB1,FAS,CASP8,STAT1,MTOR,MCL1,XIAP,PTEN,AR,BMI1,TP73,JAK2,HSPD1,ERCC2,XRCC5,YWHAZ,NFE2L2,BCL2,GSTP1,RUNX3,CDKN1A,CD44,CDKN2A,PDK2,EIF4E,HIF1A,XRCC3,ZFP36,BIRC2 |
| GO:0005615 | Extracellular space | 41 | 3195 | 8.37E-05 | LGALS1,TGFB1,HGF,SERPINE1,IFNG,TNFSF10,BMP4,GDF15,CDH1,TIMP3,EGFR,BAX,ALDH1A1,AXL,HSPA5,STK11,BSG,CTNNB1,HMGB1,FAS,FASLG,HDGF,S100A4,POSTN,IGFBP3,CD274,GPX3,HSPD1,SPP1,YWHAZ,GSTP1,ABCC1,IL6,CD44,TNF,EIF4E,H2AFX,SLC16A1,SOD2,VEGFA,MUC1 |
| GO:0000109 | Nucleotide-excision repair complex | 4 | 11 | 8.76E-05 | ERCC1,XRCC1,ERCC4,ERCC5 |
| GO:0016604 | Nuclear body | 18 | 789 | 9.43E-05 | APEX1,EPAS1,RB1,TP53,OGG1,TERT,FAS,MTOR,MKI67,PTEN,AR,BMI1,TP53BP1,RAD51,CDKN1A,BRCA1,H2AFX,HIF1A |
| GO:0070522 | ERCC4-ERCC1 complex | 3 | 3 | 0.00021 | ERCC1,XRCC1,ERCC4 |
| GO:0005576 | Extracellular region | 47 | 4166 | 0.00034 | MAPK1,LGALS1,TGFB1,HGF,SERPINE1,IFNG,TNFSF10,BMP4,GDF15,CDH1,TIMP3,EGFR,NOTCH1,BAX,ALDH1A1,AXL,GSTM1,MET,HSPA5,STK11,BSG,CTNNB1,HMGB1,FAS,FASLG,HDGF,S100A4,PTEN,POSTN,IGFBP3,CD274,GPX3,HSPD1,XRCC5,SPP1,YWHAZ,GSTP1,ABCC1,IL6,CD44,TNF,EIF4E,H2AFX,SLC16A1,SOD2,VEGFA,MUC1 |
| GO:0031983 | Vesicle lumen | 11 | 330 | 0.00036 | MAPK1,TGFB1,HGF,SERPINE1,TIMP3,EGFR,HMGB1,FASLG,XRCC5,GSTP1,VEGFA |
| GO:0090575 | RNA polymerase II transcription regulator complex | 8 | 163 | 0.00045 | STAT3,RB1,RARB,CTNNB1,E2F1,ERCC2,RUNX3,HIF1A |
| GO:0110165 | Cellular anatomical entity | 120 | 17788 | 0.00049 | ERCC1,MAPK1,LGALS1,APEX1,CSNK2A1,TNFRSF10A,TGFB1,HGF,SERPINE1,CCND1,IFNG,MLH1,DUSP1,TNFSF10,BMP4,SIRT2,ALDH1A2,GDF15,MDM2,CDH1,XRCC1,NOX4,EPAS1,PIK3CA,STAT3,TP63,TIMP3,RB1,IGF1R,TP53,ERBB2,EGFR,NOTCH1,PAK1,BAX,ALDH1A1,AXL,BIRC5,MGMT,OGG1,TERT,ADAM17,ERCC4,GSTM1,PRKDC,TYMS,USP9X,MET,CXCR2,EZH2,SOX2,HSPA5,STK11,NOS2,RARB,MTA1,BSG,PDCD1,RAD51C,CTNNB1,HMGB1,E2F1,ERCC5,FAS,CASP8,STAT1,MTOR,PTGS2,FASLG,HDGF,MKI67,S100A4,MCL1,ABCC2,XIAP,PTEN,ABCC10,UGT1A1,TLR4,AR,BMI1,TP73,POSTN,IGFBP3,KDM4C,CD274,JAK2,TP53BP1,RAD51,SIRT3,GPX3,HSPD1,ERCC2,XRCC5,SPP1,YWHAZ,NFE2L2,BCL2,GSTP1,ABCC1,RUNX3,CDKN1A,IL6,CD44,TNF,TOP2A,CDKN2A,BRCA1,PDK2,EIF4E,H2AFX,HIF1A,SLC16A1,SOD2,XRCC3,ZFP36,BIRC2,CYP1B1,VEGFA,MUC1 |
| GO:0005901 | Caveola | 6 | 78 | 0.00053 | MAPK1,CDH1,CTNNB1,PTGS2,FASLG,JAK2 |
| GO:0031982 | Vesicle | 44 | 3879 | 0.0006 | MAPK1,LGALS1,TGFB1,HGF,SERPINE1,TNFSF10,GDF15,MDM2,CDH1,TIMP3,ERBB2,EGFR,NOTCH1,BAX,ALDH1A1,AXL,CXCR2,HSPA5,STK11,NOS2,BSG,CTNNB1,HMGB1,FAS,FASLG,S100A4,TLR4,CD274,JAK2,GPX3,HSPD1,XRCC5,SPP1,YWHAZ,GSTP1,ABCC1,CD44,TNF,EIF4E,H2AFX,SLC16A1,SOD2,VEGFA,MUC1 |
| GO:0098805 | Whole membrane | 26 | 1715 | 0.0006 | MAPK1,TNFRSF10A,MDM2,CDH1,ERBB2,EGFR,BAX,ADAM17,CXCR2,BSG,CTNNB1,FAS,CASP8,MTOR,PTGS2,FASLG,MCL1,ABCC10,TLR4,CD274,JAK2,HSPD1,BCL2,CD44,TNF,BIRC2 |
| GO:0043235 | Receptor complex | 11 | 381 | 0.0011 | IGF1R,ERBB2,EGFR,NOTCH1,AXL,MET,TLR4,HSPD1,IL6,CD44,BIRC2 |
| GO:0060205 | Cytoplasmic vesicle lumen | 10 | 328 | 0.0016 | MAPK1,TGFB1,HGF,SERPINE1,TIMP3,HMGB1,FASLG,XRCC5,GSTP1,VEGFA |
| GO:0016605 | PML body | 6 | 101 | 0.0018 | RB1,TP53,TERT,MTOR,PTEN,RAD51 |
| GO:0070161 | Anchoring junction | 16 | 820 | 0.0018 | MAPK1,CCND1,SIRT2,CDH1,NOX4,EGFR,NOTCH1,PAK1,HSPA5,BSG,CTNNB1,ABCC2,JAK2,YWHAZ,CD44,VEGFA |
| GO:0098590 | Plasma membrane region | 20 | 1219 | 0.0021 | MAPK1,CDH1,NOX4,ERBB2,EGFR,NOTCH1,PAK1,ADAM17,MET,CTNNB1,PTGS2,FASLG,ABCC2,PTEN,JAK2,HSPD1,ABCC1,CD44,SLC16A1,MUC1 |
| GO:0005657 | Replication fork | 5 | 64 | 0.0022 | TP53,RAD51C,TP53BP1,H2AFX,XRCC3 |
| GO:0070062 | Extracellular exosome | 28 | 2099 | 0.0022 | LGALS1,SERPINE1,TNFSF10,GDF15,CDH1,BAX,ALDH1A1,AXL,HSPA5,STK11,BSG,CTNNB1,FAS,FASLG,S100A4,CD274,GPX3,HSPD1,SPP1,YWHAZ,GSTP1,ABCC1,CD44,EIF4E,H2AFX,SLC16A1,SOD2,MUC1 |
| GO:0000793 | Condensed chromosome | 8 | 216 | 0.0023 | MLH1,BIRC5,HMGB1,TP53BP1,RAD51,TOP2A,BRCA1,H2AFX |
| GO:0045177 | Apical part of cell | 11 | 420 | 0.0023 | NOX4,ERBB2,EGFR,NOTCH1,ADAM17,CTNNB1,ABCC2,PTEN,CD44,SLC16A1,MUC1 |
| GO:0016324 | Apical plasma membrane | 10 | 350 | 0.0024 | NOX4,ERBB2,EGFR,NOTCH1,ADAM17,ABCC2,PTEN,CD44,SLC16A1,MUC1 |
| GO:0005886 | Plasma membrane | 52 | 5314 | 0.0032 | MAPK1,CSNK2A1,TNFRSF10A,TGFB1,SERPINE1,TNFSF10,SIRT2,MDM2,CDH1,NOX4,PIK3CA,STAT3,IGF1R,ERBB2,EGFR,NOTCH1,PAK1,AXL,TERT,ADAM17,MET,CXCR2,HSPA5,NOS2,BSG,PDCD1,CTNNB1,HMGB1,FAS,CASP8,PTGS2,FASLG,ABCC2,PTEN,ABCC10,UGT1A1,TLR4,AR,CD274,JAK2,HSPD1,XRCC5,NFE2L2,GSTP1,ABCC1,IL6,CD44,TNF,BRCA1,SLC16A1,BIRC2,MUC1 |
| GO:0017053 | Transcription repressor complex | 5 | 76 | 0.0041 | CSNK2A1,CCND1,MTA1,HMGB1,HDGF |
| GO:0012505 | Endomembrane system | 46 | 4542 | 0.0046 | MAPK1,LGALS1,APEX1,TNFRSF10A,TGFB1,HGF,SERPINE1,CCND1,BMP4,GDF15,CDH1,NOX4,TP63,TIMP3,TP53,ERBB2,EGFR,NOTCH1,PAK1,BAX,CXCR2,HSPA5,MTA1,BSG,HMGB1,MTOR,PTGS2,UGT1A1,TLR4,TP73,POSTN,IGFBP3,CD274,JAK2,HSPD1,XRCC5,SPP1,NFE2L2,BCL2,GSTP1,IL6,CD44,TNF,CYP1B1,VEGFA,MUC1 |
| GO:0005730 | Nucleolus | 16 | 924 | 0.0054 | APEX1,SIRT2,MDM2,XRCC1,NOX4,TP53,TERT,PRKDC,TYMS,STAT1,MKI67,XRCC5,RUNX3,CDKN1A,TOP2A,CDKN2A |
| GO:0090734 | Site of dna damage | 5 | 82 | 0.0054 | TP53,TP53BP1,RAD51,XRCC5,H2AFX |
| GO:0009925 | Basal plasma membrane | 4 | 44 | 0.0056 | ERBB2,EGFR,MET,ABCC1 |
| GO:0034774 | Secretory granule lumen | 9 | 324 | 0.0056 | MAPK1,TGFB1,HGF,SERPINE1,TIMP3,HMGB1,XRCC5,GSTP1,VEGFA |
| GO:0033065 | Rad51C-XRCC3 complex | 2 | 2 | 0.0058 | RAD51C,XRCC3 |
| GO:0035189 | Rb-E2F complex | 2 | 2 | 0.0058 | RB1,E2F1 |
| GO:0005925 | Focal adhesion | 10 | 405 | 0.0059 | MAPK1,NOX4,EGFR,PAK1,HSPA5,BSG,CTNNB1,JAK2,YWHAZ,CD44 |
| GO:0043209 | Myelin sheath | 4 | 46 | 0.0062 | SIRT2,ERBB2,PTEN,BCL2 |
| GO:0099524 | Postsynaptic cytosol | 3 | 18 | 0.0067 | MTOR,PTEN,EIF4E |
| GO:0000110 | Nucleotide-excision repair factor 1 complex | 2 | 3 | 0.0088 | ERCC1,ERCC4 |
| GO:0098797 | Plasma membrane protein complex | 11 | 547 | 0.0147 | CDH1,NOX4,IGF1R,ERBB2,EGFR,CTNNB1,HMGB1,FAS,CASP8,IL6,BIRC2 |
| GO:0035861 | Site of double-strand break | 4 | 60 | 0.0148 | TP53,TP53BP1,RAD51,H2AFX |
| GO:0016323 | Basolateral plasma membrane | 7 | 237 | 0.0169 | ERBB2,EGFR,MET,CTNNB1,ABCC1,CD44,SLC16A1 |
| GO:0030054 | Cell junction | 25 | 2075 | 0.0169 | MAPK1,CCND1,SIRT2,MDM2,CDH1,NOX4,STAT3,EGFR,NOTCH1,PAK1,HSPA5,BSG,RAD51C,CTNNB1,MTOR,ABCC2,PTEN,TP73,POSTN,JAK2,YWHAZ,CD44,EIF4E,SLC16A1,VEGFA |
| GO:0030141 | Secretory granule | 14 | 845 | 0.0169 | MAPK1,TGFB1,HGF,SERPINE1,TIMP3,NOTCH1,CXCR2,BSG,HMGB1,HSPD1,XRCC5,GSTP1,CD44,VEGFA |
| GO:0046696 | Lipopolysaccharide receptor complex | 2 | 5 | 0.0169 | TLR4,HSPD1 |
| GO:0005887 | Integral component of plasma membrane | 21 | 1623 | 0.0195 | TNFSF10,NOX4,IGF1R,ERBB2,EGFR,AXL,ADAM17,MET,CXCR2,BSG,FASLG,ABCC2,UGT1A1,TLR4,ABCC1,IL6,CD44,TNF,SLC16A1,BIRC2,MUC1 |
| GO:0031093 | Platelet alpha granule lumen | 4 | 68 | 0.0208 | TGFB1,HGF,SERPINE1,VEGFA |
| GO:0031265 | CD95 death-inducing signaling complex | 2 | 6 | 0.0211 | FAS,CASP8 |
| GO:0097136 | Bcl-2 family protein complex | 2 | 7 | 0.0267 | BAX,MCL1 |
| GO:0032993 | protein-DNA complex | 6 | 195 | 0.03 | TERT,PRKDC,CTNNB1,XRCC5,NFE2L2,H2AFX |
| GO:0016600 | Flotillin complex | 2 | 8 | 0.0325 | CDH1,CTNNB1 |
| GO:0098552 | Side of membrane | 10 | 531 | 0.0344 | CDH1,CXCR2,PDCD1,FAS,FASLG,PTEN,TLR4,CD274,TNF,BIRC2 |
| GO:0098796 | Membrane protein complex | 16 | 1141 | 0.0344 | CDH1,NOX4,PIK3CA,IGF1R,ERBB2,EGFR,BAX,CTNNB1,HMGB1,FAS,CASP8,TLR4,HSPD1,BCL2,IL6,BIRC2 |
| GO:0033643 | Host cell part | 6 | 205 | 0.0358 | CCND1,AXL,RARB,AR,NFE2L2,TOP2A |
| GO:0070419 | Nonhomologous end joining complex | 2 | 9 | 0.0369 | PRKDC,XRCC5 |
| GO:0000795 | Synaptonemal complex | 3 | 41 | 0.045 | MLH1,RAD51,BRCA1 |
| GO:0031968 | Organelle outer membrane | 6 | 220 | 0.0477 | BAX,CASP8,MTOR,PTGS2,MCL1,BCL2 |
| GO:0005815 | Microtubule organizing center | 12 | 761 | 0.0482 | MAPK1,APEX1,SIRT2,TP53,BIRC5,CTNNB1,E2F1,RAD51,NFE2L2,BRCA1,H2AFX,SLC16A1 |
| GO:0005635 | Nuclear envelope | 9 | 472 | 0.0483 | CCND1,TP53,EGFR,PAK1,BAX,MTA1,MTOR,PTGS2,BCL2 |
| GO:0034663 | Endoplasmic reticulum chaperone complex | 2 | 11 | 0.0483 | HSPA5,UGT1A1 |
| GO:0043220 | Schmidt-Lanterman incisure | 2 | 11 | 0.0483 | SIRT2,PTEN |

**S5:**

| #term ID | term description | observed gene count | background gene count | false discovery rate | matching proteins in your network (labels) |
| --- | --- | --- | --- | --- | --- |
| GO:0005515 | Protein binding | 106 | 7026 | 5.10E-29 | ERCC1,MAPK1,LGALS1,APEX1,CSNK2A1,TNFRSF10A,TGFB1,HGF,SERPINE1,CCND1,IFNG,MLH1,DUSP1,TNFSF10,BMP4,SIRT2,GDF15,MDM2,CDH1,XRCC1,NOX4,EPAS1,PIK3CA,STAT3,TP63,TIMP3,RB1,IGF1R,TP53,ERBB2,EGFR,NOTCH1,PAK1,BAX,AXL,BIRC5,OGG1,TERT,ADAM17,ERCC4,GSTM1,PRKDC,TYMS,USP9X,MET,CXCR2,HSPA5,STK11,NOS2,RARB,MTA1,BSG,CTNNB1,HMGB1,E2F1,ERCC5,FAS,CASP8,STAT1,MTOR,PTGS2,FASLG,HDGF,MKI67,S100A4,MCL1,ABCC2,XIAP,PTEN,UGT1A1,TLR4,AR,BMI1,TP73,POSTN,IGFBP3,KDM4C,JAK2,TP53BP1,RAD51,SIRT3,GPX3,HSPD1,ERCC2,XRCC5,SPP1,YWHAZ,NFE2L2,BCL2,GSTP1,CDKN1A,IL6,TNF,TOP2A,CDKN2A,BRCA1,PDK2,EIF4E,H2AFX,HIF1A,SLC16A1,SOD2,ZFP36,BIRC2,VEGFA,MUC1 |
| GO:0019899 | Enzyme binding | 57 | 2239 | 2.60E-19 | MAPK1,TNFRSF10A,TGFB1,SERPINE1,CCND1,MLH1,DUSP1,SIRT2,MDM2,XRCC1,NOX4,EPAS1,STAT3,TIMP3,RB1,TP53,ERBB2,EGFR,NOTCH1,PAK1,BIRC5,GSTM1,PRKDC,MET,HSPA5,MTA1,CTNNB1,HMGB1,E2F1,ERCC5,FAS,CASP8,STAT1,MTOR,PTGS2,PTEN,UGT1A1,AR,TP73,KDM4C,JAK2,RAD51,SIRT3,HSPD1,XRCC5,YWHAZ,BCL2,GSTP1,CDKN1A,TNF,TOP2A,CDKN2A,BRCA1,EIF4E,H2AFX,HIF1A,ZFP36 |
| GO:0042802 | Identical protein binding | 52 | 1896 | 1.29E-18 | MAPK1,LGALS1,CSNK2A1,TNFRSF10A,TGFB1,HGF,TNFSF10,GDF15,MDM2,CDH1,STAT3,TP63,RB1,IGF1R,TP53,ERBB2,EGFR,PAK1,BAX,BIRC5,TERT,ERCC4,GSTM1,TYMS,MET,NOS2,ERCC5,FAS,CASP8,STAT1,MTOR,PTGS2,S100A4,MCL1,XIAP,PTEN,UGT1A1,TLR4,TP73,JAK2,RAD51,GPX3,YWHAZ,BCL2,TNF,TOP2A,BRCA1,PDK2,SLC16A1,SOD2,BIRC2,VEGFA |
| GO:0005488 | Binding | 118 | 12516 | 1.19E-17 | ERCC1,MAPK1,LGALS1,APEX1,CSNK2A1,TNFRSF10A,TGFB1,HGF,SERPINE1,CCND1,IFNG,MLH1,DUSP1,TNFSF10,BMP4,SIRT2,ALDH1A2,GDF15,MDM2,CDH1,XRCC1,NOX4,EPAS1,PIK3CA,STAT3,TP63,TIMP3,RB1,IGF1R,TP53,ERBB2,EGFR,NOTCH1,PAK1,BAX,ALDH1A1,AXL,BIRC5,MGMT,OGG1,TERT,ADAM17,ERCC4,GSTM1,PRKDC,TYMS,USP9X,MET,CXCR2,EZH2,SOX2,HSPA5,STK11,NOS2,RARB,MTA1,BSG,RAD51C,CTNNB1,HMGB1,E2F1,ERCC5,FAS,CASP8,STAT1,MTOR,PTGS2,FASLG,HDGF,MKI67,S100A4,MCL1,ABCC2,XIAP,PTEN,ABCC10,UGT1A1,TLR4,AR,BMI1,TP73,POSTN,IGFBP3,KDM4C,JAK2,TP53BP1,RAD51,SIRT3,GPX3,HSPD1,ERCC2,XRCC5,SPP1,YWHAZ,NFE2L2,BCL2,GSTP1,ABCC1,RUNX3,CDKN1A,IL6,CD44,TNF,TOP2A,CDKN2A,BRCA1,PDK2,EIF4E,H2AFX,HIF1A,SLC16A1,SOD2,XRCC3,ZFP36,BIRC2,CYP1B1,VEGFA,MUC1 |
| GO:0008134 | Transcription factor binding | 33 | 672 | 1.19E-17 | ERCC1,MAPK1,APEX1,TNFRSF10A,CCND1,SIRT2,EPAS1,STAT3,RB1,TP53,TERT,ERCC4,PRKDC,RARB,MTA1,CTNNB1,HMGB1,E2F1,STAT1,MTOR,HDGF,AR,TP73,KDM4C,TP53BP1,GPX3,YWHAZ,NFE2L2,BCL2,CDKN2A,BRCA1,EIF4E,HIF1A |
| GO:0003677 | DNA binding | 49 | 2470 | 7.21E-12 | ERCC1,MAPK1,APEX1,MLH1,XRCC1,EPAS1,STAT3,TP63,RB1,TP53,EGFR,NOTCH1,MGMT,OGG1,TERT,ERCC4,PRKDC,EZH2,SOX2,RARB,MTA1,RAD51C,HMGB1,E2F1,ERCC5,STAT1,MTOR,HDGF,MKI67,AR,TP73,TP53BP1,RAD51,SIRT3,HSPD1,ERCC2,XRCC5,NFE2L2,BCL2,RUNX3,TNF,TOP2A,CDKN2A,BRCA1,H2AFX,HIF1A,XRCC3,ZFP36,MUC1 |
| GO:0097159 | Organic cyclic compound binding | 77 | 5916 | 1.07E-11 | ERCC1,MAPK1,LGALS1,APEX1,CSNK2A1,MLH1,SIRT2,MDM2,XRCC1,NOX4,EPAS1,PIK3CA,STAT3,TP63,RB1,IGF1R,TP53,ERBB2,EGFR,NOTCH1,PAK1,ALDH1A1,AXL,MGMT,OGG1,TERT,ERCC4,PRKDC,TYMS,MET,EZH2,SOX2,HSPA5,STK11,NOS2,RARB,MTA1,RAD51C,HMGB1,E2F1,ERCC5,STAT1,MTOR,PTGS2,HDGF,MKI67,S100A4,ABCC2,ABCC10,UGT1A1,AR,TP73,JAK2,TP53BP1,RAD51,SIRT3,HSPD1,ERCC2,XRCC5,YWHAZ,NFE2L2,BCL2,ABCC1,RUNX3,TNF,TOP2A,CDKN2A,BRCA1,PDK2,EIF4E,H2AFX,HIF1A,SLC16A1,XRCC3,ZFP36,CYP1B1,MUC1 |
| GO:0003684 | Damaged dna binding | 12 | 64 | 1.36E-11 | ERCC1,APEX1,XRCC1,TP63,OGG1,ERCC4,HMGB1,TP53BP1,ERCC2,XRCC5,BRCA1,H2AFX |
| GO:1901363 | Heterocyclic compound binding | 75 | 5831 | 5.87E-11 | ERCC1,MAPK1,LGALS1,APEX1,CSNK2A1,MLH1,SIRT2,MDM2,XRCC1,NOX4,EPAS1,PIK3CA,STAT3,TP63,RB1,IGF1R,TP53,ERBB2,EGFR,NOTCH1,PAK1,ALDH1A1,AXL,MGMT,OGG1,TERT,ERCC4,PRKDC,TYMS,MET,EZH2,SOX2,HSPA5,STK11,NOS2,RARB,MTA1,RAD51C,HMGB1,E2F1,ERCC5,STAT1,MTOR,PTGS2,HDGF,MKI67,S100A4,ABCC2,ABCC10,AR,TP73,JAK2,TP53BP1,RAD51,SIRT3,HSPD1,ERCC2,XRCC5,YWHAZ,NFE2L2,BCL2,ABCC1,RUNX3,TNF,TOP2A,CDKN2A,BRCA1,PDK2,EIF4E,H2AFX,HIF1A,XRCC3,ZFP36,CYP1B1,MUC1 |
| GO:0003690 | Double-stranded dna binding | 31 | 1156 | 9.22E-10 | MAPK1,APEX1,MLH1,EPAS1,STAT3,TP63,RB1,TP53,EGFR,NOTCH1,PRKDC,EZH2,SOX2,RARB,MTA1,HMGB1,E2F1,ERCC5,STAT1,MTOR,AR,TP73,RAD51,HSPD1,XRCC5,NFE2L2,RUNX3,TNF,BRCA1,HIF1A,MUC1 |
| GO:0005102 | Signaling receptor binding | 35 | 1581 | 4.64E-09 | TGFB1,HGF,SERPINE1,IFNG,TNFSF10,BMP4,GDF15,MDM2,STAT3,RB1,IGF1R,TP53,ERBB2,EGFR,NOTCH1,ADAM17,RARB,CTNNB1,HMGB1,CASP8,STAT1,FASLG,HDGF,S100A4,PTEN,TLR4,AR,KDM4C,JAK2,SPP1,IL6,TNF,BRCA1,HIF1A,VEGFA |
| GO:0019904 | Protein domain specific binding | 24 | 716 | 4.64E-09 | ERCC1,MDM2,TP63,RB1,TP53,BAX,ADAM17,PRKDC,HSPA5,STK11,CTNNB1,CASP8,MTOR,MCL1,ABCC2,PTEN,AR,BMI1,JAK2,YWHAZ,NFE2L2,BCL2,CDKN2A,HIF1A |
| GO:0002039 | p53 binding | 10 | 69 | 1.22E-08 | MDM2,TP63,TP53,STK11,TP73,TP53BP1,HSPD1,CDKN2A,HIF1A,MUC1 |
| GO:0098772 | Molecular function regulator | 51 | 3422 | 3.97E-08 | APEX1,TGFB1,HGF,SERPINE1,CCND1,IFNG,TNFSF10,BMP4,GDF15,EPAS1,PIK3CA,STAT3,TP63,TIMP3,RB1,TP53,NOTCH1,ALDH1A1,BIRC5,EZH2,SOX2,STK11,RARB,MTA1,CTNNB1,HMGB1,E2F1,STAT1,FASLG,HDGF,XIAP,UGT1A1,AR,TP73,IGFBP3,TP53BP1,XRCC5,SPP1,NFE2L2,BCL2,GSTP1,RUNX3,CDKN1A,IL6,TNF,CDKN2A,BRCA1,HIF1A,BIRC2,VEGFA,MUC1 |
| GO:0019900 | Kinase binding | 23 | 742 | 4.30E-08 | MAPK1,CCND1,DUSP1,NOX4,STAT3,RB1,TP53,EGFR,PAK1,CTNNB1,E2F1,FAS,MTOR,PTEN,TP73,JAK2,YWHAZ,GSTP1,CDKN1A,TOP2A,CDKN2A,HIF1A,ZFP36 |
| GO:0003676 | Nucleic acid binding | 55 | 3947 | 5.36E-08 | ERCC1,MAPK1,LGALS1,APEX1,MLH1,MDM2,XRCC1,EPAS1,STAT3,TP63,RB1,TP53,EGFR,NOTCH1,MGMT,OGG1,TERT,ERCC4,PRKDC,TYMS,EZH2,SOX2,RARB,MTA1,RAD51C,HMGB1,E2F1,ERCC5,STAT1,MTOR,HDGF,MKI67,S100A4,AR,TP73,TP53BP1,RAD51,SIRT3,HSPD1,ERCC2,XRCC5,YWHAZ,NFE2L2,BCL2,RUNX3,TNF,TOP2A,CDKN2A,BRCA1,EIF4E,H2AFX,HIF1A,XRCC3,ZFP36,MUC1 |
| GO:0036094 | Small molecule binding | 42 | 2516 | 1.05E-07 | MAPK1,CSNK2A1,MLH1,SIRT2,ALDH1A2,NOX4,PIK3CA,IGF1R,TP53,ERBB2,EGFR,PAK1,ALDH1A1,AXL,PRKDC,TYMS,MET,HSPA5,STK11,NOS2,BSG,RAD51C,MTOR,HDGF,MKI67,ABCC2,ABCC10,UGT1A1,JAK2,RAD51,SIRT3,HSPD1,ERCC2,XRCC5,GSTP1,ABCC1,RUNX3,CD44,TOP2A,PDK2,XRCC3,CYP1B1 |
| GO:0043167 | Ion binding | 71 | 6188 | 1.05E-07 | MAPK1,APEX1,CSNK2A1,MLH1,TNFSF10,BMP4,SIRT2,MDM2,CDH1,NOX4,PIK3CA,TP63,TIMP3,IGF1R,TP53,ERBB2,EGFR,NOTCH1,PAK1,AXL,BIRC5,MGMT,TERT,ADAM17,GSTM1,PRKDC,TYMS,MET,HSPA5,STK11,NOS2,RARB,MTA1,RAD51C,HMGB1,ERCC5,MTOR,PTGS2,HDGF,MKI67,S100A4,ABCC2,XIAP,ABCC10,UGT1A1,AR,BMI1,TP73,POSTN,IGFBP3,KDM4C,JAK2,RAD51,SIRT3,HSPD1,ERCC2,XRCC5,GSTP1,ABCC1,RUNX3,CDKN1A,CD44,TOP2A,BRCA1,PDK2,SOD2,XRCC3,ZFP36,BIRC2,CYP1B1,VEGFA |
| GO:0019901 | Protein kinase binding | 21 | 653 | 1.22E-07 | MAPK1,CCND1,DUSP1,NOX4,STAT3,TP53,EGFR,PAK1,CTNNB1,E2F1,MTOR,PTEN,TP73,JAK2,YWHAZ,GSTP1,CDKN1A,TOP2A,CDKN2A,HIF1A,ZFP36 |
| GO:0140297 | DNA-binding transcription factor binding | 16 | 366 | 2.37E-07 | APEX1,STAT3,RB1,RARB,MTA1,CTNNB1,HMGB1,STAT1,KDM4C,TP53BP1,NFE2L2,BCL2,CDKN2A,BRCA1,EIF4E,HIF1A |
| GO:0003824 | Catalytic activity | 65 | 5486 | 2.89E-07 | ERCC1,MAPK1,APEX1,CSNK2A1,HGF,CCND1,MLH1,DUSP1,SIRT2,ALDH1A2,MDM2,XRCC1,NOX4,PIK3CA,IGF1R,ERBB2,EGFR,PAK1,ALDH1A1,AXL,MGMT,OGG1,TERT,ADAM17,ERCC4,GSTM1,PRKDC,TYMS,USP9X,MET,EZH2,HSPA5,STK11,NOS2,RAD51C,HMGB1,ERCC5,CASP8,MTOR,PTGS2,ABCC2,XIAP,PTEN,ABCC10,UGT1A1,TLR4,KDM4C,JAK2,RAD51,SIRT3,GPX3,HSPD1,ERCC2,XRCC5,GSTP1,ABCC1,CDKN1A,TOP2A,CDKN2A,BRCA1,PDK2,SOD2,XRCC3,BIRC2,CYP1B1 |
| GO:0043565 | Sequence-specific dna binding | 29 | 1331 | 2.92E-07 | APEX1,EPAS1,STAT3,TP63,RB1,TP53,NOTCH1,TERT,EZH2,SOX2,RARB,MTA1,HMGB1,E2F1,STAT1,MTOR,AR,TP73,TP53BP1,SIRT3,HSPD1,XRCC5,NFE2L2,BCL2,RUNX3,TNF,BRCA1,HIF1A,MUC1 |
| GO:0003682 | Chromatin binding | 19 | 570 | 3.93E-07 | ERCC1,APEX1,MLH1,SIRT2,STAT3,TP63,TP53,EGFR,NOTCH1,ERCC4,EZH2,MTA1,CTNNB1,STAT1,AR,BMI1,KDM4C,RAD51,TOP2A |
| GO:0140097 | Catalytic activity, acting on dna | 12 | 184 | 3.93E-07 | APEX1,XRCC1,MGMT,OGG1,TERT,ERCC4,RAD51C,ERCC5,RAD51,ERCC2,XRCC5,TOP2A |
| GO:0043168 | Anion binding | 43 | 2805 | 5.72E-07 | MAPK1,CSNK2A1,MLH1,BMP4,SIRT2,NOX4,PIK3CA,IGF1R,TP53,ERBB2,EGFR,PAK1,AXL,GSTM1,PRKDC,TYMS,MET,HSPA5,STK11,NOS2,RAD51C,HMGB1,MTOR,HDGF,MKI67,ABCC2,ABCC10,UGT1A1,POSTN,JAK2,RAD51,SIRT3,HSPD1,ERCC2,XRCC5,GSTP1,ABCC1,RUNX3,CD44,TOP2A,PDK2,XRCC3,VEGFA |
| GO:0000166 | Nucleotide binding | 36 | 2119 | 1.25E-06 | MAPK1,CSNK2A1,MLH1,SIRT2,NOX4,PIK3CA,IGF1R,TP53,ERBB2,EGFR,PAK1,ALDH1A1,AXL,PRKDC,TYMS,MET,HSPA5,STK11,NOS2,RAD51C,MTOR,HDGF,MKI67,ABCC2,ABCC10,JAK2,RAD51,SIRT3,HSPD1,ERCC2,XRCC5,ABCC1,RUNX3,TOP2A,PDK2,XRCC3 |
| GO:0097367 | Carbohydrate derivative binding | 37 | 2226 | 1.25E-06 | MAPK1,CSNK2A1,MLH1,BMP4,PIK3CA,IGF1R,TP53,ERBB2,EGFR,PAK1,AXL,PRKDC,MET,HSPA5,STK11,NOS2,RAD51C,HMGB1,MTOR,HDGF,MKI67,ABCC2,ABCC10,TLR4,POSTN,JAK2,RAD51,HSPD1,ERCC2,XRCC5,ABCC1,RUNX3,CD44,TOP2A,PDK2,XRCC3,VEGFA |
| GO:0044389 | Ubiquitin-like protein ligase binding | 14 | 312 | 1.36E-06 | MDM2,RB1,TP53,EGFR,HSPA5,CASP8,STAT1,HSPD1,XRCC5,YWHAZ,BCL2,CDKN1A,BRCA1,HIF1A |
| GO:0005524 | ATP binding | 29 | 1464 | 1.75E-06 | MAPK1,CSNK2A1,MLH1,PIK3CA,IGF1R,TP53,ERBB2,EGFR,PAK1,AXL,PRKDC,MET,HSPA5,STK11,RAD51C,MTOR,MKI67,ABCC2,ABCC10,JAK2,RAD51,HSPD1,ERCC2,XRCC5,ABCC1,RUNX3,TOP2A,PDK2,XRCC3 |
| GO:0061629 | RNA polymerase II-specific DNA-binding transcription factor binding | 13 | 283 | 3.35E-06 | APEX1,STAT3,RB1,RARB,MTA1,CTNNB1,STAT1,KDM4C,TP53BP1,NFE2L2,CDKN2A,BRCA1,HIF1A |
| GO:1990837 | Sequence-specific double-stranded dna binding | 24 | 1068 | 3.69E-06 | EPAS1,STAT3,TP63,RB1,TP53,NOTCH1,EZH2,SOX2,RARB,MTA1,HMGB1,E2F1,STAT1,MTOR,AR,TP73,HSPD1,XRCC5,NFE2L2,RUNX3,TNF,BRCA1,HIF1A,MUC1 |
| GO:0031625 | Ubiquitin protein ligase binding | 13 | 296 | 4.92E-06 | MDM2,RB1,TP53,EGFR,HSPA5,CASP8,HSPD1,XRCC5,YWHAZ,BCL2,CDKN1A,BRCA1,HIF1A |
| GO:0008022 | Protein c-terminus binding | 11 | 199 | 5.92E-06 | ERCC1,ERBB2,TERT,ERCC4,CTNNB1,MKI67,JAK2,RAD51,ERCC2,XRCC5,TOP2A |
| GO:0000976 | Transcription regulatory region sequence-specific dna binding | 23 | 1028 | 6.98E-06 | EPAS1,STAT3,TP63,RB1,TP53,NOTCH1,EZH2,SOX2,RARB,MTA1,HMGB1,E2F1,STAT1,MTOR,AR,TP73,XRCC5,NFE2L2,RUNX3,TNF,BRCA1,HIF1A,MUC1 |
| GO:0046983 | Protein dimerization activity | 23 | 1037 | 7.72E-06 | GDF15,EPAS1,STAT3,TP53,ERBB2,BAX,BIRC5,TERT,GSTM1,NOS2,E2F1,ERCC5,STAT1,PTGS2,MCL1,UGT1A1,TLR4,BCL2,TOP2A,PDK2,H2AFX,HIF1A,VEGFA |
| GO:0044877 | Protein-containing complex binding | 25 | 1216 | 7.90E-06 | ERCC1,APEX1,HGF,CCND1,MDM2,IGF1R,TP53,EGFR,PAK1,ADAM17,ERCC4,EZH2,HSPA5,RARB,HMGB1,ERCC5,CASP8,MTOR,PTEN,HSPD1,XRCC5,SPP1,CDKN1A,CD44,BIRC2 |
| GO:0005126 | Cytokine receptor binding | 12 | 264 | 9.86E-06 | TGFB1,IFNG,TNFSF10,STAT3,ADAM17,CASP8,STAT1,FASLG,JAK2,IL6,TNF,VEGFA |
| GO:0005125 | Cytokine activity | 11 | 233 | 2.31E-05 | TGFB1,IFNG,TNFSF10,BMP4,GDF15,HMGB1,FASLG,SPP1,IL6,TNF,VEGFA |
| GO:0004672 | Protein kinase activity | 16 | 568 | 3.95E-05 | MAPK1,CSNK2A1,CCND1,PIK3CA,IGF1R,ERBB2,EGFR,PAK1,AXL,PRKDC,MET,STK11,MTOR,JAK2,CDKN1A,PDK2 |
| GO:0070491 | Repressing transcription factor binding | 7 | 73 | 4.24E-05 | STAT3,MTA1,CTNNB1,HMGB1,STAT1,BCL2,EIF4E |
| GO:0047485 | Protein n-terminus binding | 8 | 110 | 4.53E-05 | CSNK2A1,MDM2,TP53,TERT,ERCC4,ERCC5,ERCC2,BIRC2 |
| GO:0097371 | MDM2/MDM4 family protein binding | 4 | 9 | 6.33E-05 | TP63,TP53,TP73,CDKN2A |
| GO:0032553 | Ribonucleotide binding | 30 | 1880 | 6.34E-05 | MAPK1,CSNK2A1,MLH1,PIK3CA,IGF1R,TP53,ERBB2,EGFR,PAK1,AXL,PRKDC,MET,HSPA5,STK11,NOS2,RAD51C,MTOR,MKI67,ABCC2,ABCC10,JAK2,RAD51,HSPD1,ERCC2,XRCC5,ABCC1,RUNX3,TOP2A,PDK2,XRCC3 |
| GO:0046982 | Protein heterodimerization activity | 12 | 338 | 0.0001 | EPAS1,TP53,ERBB2,BAX,BIRC5,MCL1,UGT1A1,TLR4,BCL2,TOP2A,H2AFX,HIF1A |
| GO:0035035 | Histone acetyltransferase binding | 5 | 29 | 0.00012 | SIRT2,EPAS1,TP53,STAT1,HIF1A |
| GO:0005164 | Tumor necrosis factor receptor binding | 5 | 31 | 0.00016 | TNFSF10,CASP8,STAT1,FASLG,TNF |
| GO:0002020 | Protease binding | 8 | 138 | 0.00019 | TNFRSF10A,SERPINE1,TIMP3,TP53,PTEN,HSPD1,BCL2,TNF |
| GO:0051427 | Hormone receptor binding | 9 | 188 | 0.0002 | STAT3,RB1,RARB,CTNNB1,STAT1,KDM4C,JAK2,BRCA1,HIF1A |
| GO:0140096 | Catalytic activity, acting on a protein | 31 | 2116 | 0.0002 | MAPK1,CSNK2A1,HGF,CCND1,DUSP1,SIRT2,MDM2,PIK3CA,IGF1R,ERBB2,EGFR,PAK1,AXL,ADAM17,PRKDC,USP9X,MET,EZH2,STK11,CASP8,MTOR,XIAP,PTEN,KDM4C,JAK2,SIRT3,CDKN1A,CDKN2A,BRCA1,PDK2,BIRC2 |
| GO:1990841 | Promoter-specific chromatin binding | 6 | 61 | 0.0002 | ERCC1,TP53,ERCC4,EZH2,STAT1,BMI1 |
| GO:0097718 | Disordered domain specific binding | 5 | 34 | 0.00021 | MDM2,RB1,TP53,CTNNB1,CDKN2A |
| GO:0019902 | Phosphatase binding | 9 | 194 | 0.00023 | MAPK1,STAT3,TP53,ERBB2,EGFR,MET,CTNNB1,STAT1,BCL2 |
| GO:0042803 | Protein homodimerization activity | 16 | 673 | 0.00024 | GDF15,STAT3,BAX,BIRC5,TERT,GSTM1,NOS2,ERCC5,STAT1,PTGS2,MCL1,UGT1A1,BCL2,TOP2A,PDK2,VEGFA |
| GO:0004520 | Endodeoxyribonuclease activity | 5 | 36 | 0.00025 | APEX1,XRCC1,ERCC4,RAD51C,ERCC5 |
| GO:0019903 | Protein phosphatase binding | 8 | 149 | 0.00028 | STAT3,TP53,ERBB2,EGFR,MET,CTNNB1,STAT1,BCL2 |
| GO:0051087 | Chaperone binding | 7 | 105 | 0.00028 | TP53,BAX,BIRC5,TERT,HSPA5,HSPD1,BIRC2 |
| GO:0016887 | ATPase activity | 12 | 393 | 0.00035 | MLH1,HSPA5,RAD51C,ABCC2,ABCC10,RAD51,HSPD1,ERCC2,XRCC5,ABCC1,TOP2A,XRCC3 |
| GO:0035257 | Nuclear hormone receptor binding | 8 | 155 | 0.00035 | STAT3,RB1,RARB,CTNNB1,STAT1,KDM4C,BRCA1,HIF1A |
| GO:0140110 | Transcription regulator activity | 26 | 1657 | 0.00038 | APEX1,CCND1,EPAS1,STAT3,TP63,RB1,TP53,NOTCH1,EZH2,SOX2,RARB,MTA1,CTNNB1,HMGB1,E2F1,STAT1,HDGF,AR,TP73,TP53BP1,NFE2L2,RUNX3,BRCA1,HIF1A,BIRC2,MUC1 |
| GO:0140296 | General transcription initiation factor binding | 5 | 41 | 0.00041 | ERCC1,TP53,ERCC4,MTOR,AR |
| GO:0001091 | RNA polymerase II general transcription initiation factor binding | 4 | 19 | 0.0005 | ERCC1,TP53,ERCC4,AR |
| GO:0048018 | Receptor ligand activity | 13 | 490 | 0.00057 | TGFB1,HGF,IFNG,TNFSF10,BMP4,GDF15,HMGB1,FASLG,HDGF,SPP1,IL6,TNF,VEGFA |
| GO:0004519 | Endonuclease activity | 7 | 120 | 0.00058 | ERCC1,APEX1,XRCC1,OGG1,ERCC4,RAD51C,ERCC5 |
| GO:0016740 | Transferase activity | 30 | 2170 | 0.00071 | MAPK1,CSNK2A1,CCND1,SIRT2,MDM2,PIK3CA,IGF1R,ERBB2,EGFR,PAK1,AXL,MGMT,TERT,GSTM1,PRKDC,TYMS,MET,EZH2,STK11,MTOR,XIAP,UGT1A1,JAK2,SIRT3,GSTP1,CDKN1A,CDKN2A,BRCA1,PDK2,BIRC2 |
| GO:0051434 | BH3 domain binding | 3 | 6 | 0.00077 | BAX,MCL1,BCL2 |
| GO:0001216 | DNA-binding transcription activator activity | 12 | 452 | 0.0011 | EPAS1,STAT3,TP63,TP53,NOTCH1,SOX2,E2F1,STAT1,AR,TP73,NFE2L2,HIF1A |
| GO:0072341 | Modified amino acid binding | 6 | 90 | 0.0011 | NOX4,AXL,GSTM1,TYMS,HMGB1,GSTP1 |
| GO:0000987 | Cis-regulatory region sequence-specific dna binding | 15 | 701 | 0.0012 | EPAS1,STAT3,RB1,TP53,NOTCH1,EZH2,MTA1,E2F1,STAT1,MTOR,AR,TP73,NFE2L2,HIF1A,MUC1 |
| GO:0001099 | Basal rna polymerase ii transcription machinery binding | 5 | 54 | 0.0012 | ERCC1,TP53,ERCC4,ERCC5,AR |
| GO:0008094 | DNA-dependent ATPase activity | 6 | 93 | 0.0012 | RAD51C,RAD51,ERCC2,XRCC5,TOP2A,XRCC3 |
| GO:0016772 | Transferase activity, transferring phosphorus-containing groups | 17 | 875 | 0.0012 | MAPK1,CSNK2A1,CCND1,PIK3CA,IGF1R,ERBB2,EGFR,PAK1,AXL,TERT,PRKDC,MET,STK11,MTOR,JAK2,CDKN1A,PDK2 |
| GO:0004714 | Transmembrane receptor protein tyrosine kinase activity | 5 | 63 | 0.0022 | IGF1R,ERBB2,EGFR,AXL,MET |
| GO:0043560 | Insulin receptor substrate binding | 3 | 10 | 0.0022 | PIK3CA,IGF1R,JAK2 |
| GO:0001094 | TFIID-class transcription factor complex binding | 3 | 11 | 0.0028 | ERCC1,TP53,ERCC4 |
| GO:0008083 | Growth factor activity | 7 | 161 | 0.0028 | TGFB1,HGF,BMP4,GDF15,HDGF,IL6,VEGFA |
| GO:0003697 | Single-stranded dna binding | 6 | 113 | 0.0032 | ERCC1,ERCC4,HMGB1,ERCC5,RAD51,HSPD1 |
| GO:0016922 | Nuclear receptor binding | 6 | 114 | 0.0033 | STAT3,RB1,RARB,CTNNB1,KDM4C,BRCA1 |
| GO:0042826 | Histone deacetylase binding | 6 | 114 | 0.0033 | CCND1,SIRT2,TP53,MTA1,TOP2A,HIF1A |
| GO:0042162 | Telomeric dna binding | 4 | 36 | 0.0035 | APEX1,TERT,TP53BP1,XRCC5 |
| GO:0000977 | RNA polymerase II transcription regulatory region sequence-specific DNA binding | 16 | 878 | 0.0036 | EPAS1,STAT3,TP63,RB1,TP53,NOTCH1,EZH2,RARB,MTA1,STAT1,AR,TP73,NFE2L2,RUNX3,HIF1A,MUC1 |
| GO:0001228 | DNA-binding transcription activator activity, RNA polymerase II-specific | 11 | 449 | 0.004 | EPAS1,STAT3,TP63,TP53,NOTCH1,SOX2,STAT1,AR,TP73,NFE2L2,HIF1A |
| GO:0042056 | Chemoattractant activity | 4 | 41 | 0.0053 | HGF,BMP4,HMGB1,VEGFA |
| GO:0050681 | Androgen receptor binding | 4 | 44 | 0.0067 | RB1,CTNNB1,KDM4C,BRCA1 |
| GO:0003712 | Transcription coregulator activity | 12 | 571 | 0.0075 | APEX1,CCND1,RB1,EZH2,MTA1,CTNNB1,HMGB1,HDGF,TP53BP1,BRCA1,BIRC2,MUC1 |
| GO:0030234 | Enzyme regulator activity | 17 | 1044 | 0.0075 | SERPINE1,CCND1,PIK3CA,TIMP3,TP53,NOTCH1,ALDH1A1,BIRC5,STK11,XIAP,UGT1A1,IGFBP3,XRCC5,GSTP1,CDKN1A,CDKN2A,BIRC2 |
| GO:0042562 | Hormone binding | 5 | 86 | 0.0075 | IGF1R,EGFR,ALDH1A1,AR,HSPD1 |
| GO:0004713 | Protein tyrosine kinase activity | 6 | 137 | 0.0076 | IGF1R,ERBB2,EGFR,AXL,MET,JAK2 |
| GO:0035258 | Steroid hormone receptor binding | 5 | 93 | 0.0102 | STAT3,RB1,CTNNB1,KDM4C,BRCA1 |
| GO:0001102 | RNA polymerase II activating transcription factor binding | 4 | 53 | 0.0122 | RB1,CTNNB1,TP53BP1,NFE2L2 |
| GO:0003723 | RNA binding | 22 | 1649 | 0.0134 | LGALS1,APEX1,MDM2,STAT3,TP53,TERT,PRKDC,TYMS,EZH2,SOX2,HMGB1,HDGF,MKI67,S100A4,HSPD1,XRCC5,YWHAZ,TOP2A,CDKN2A,BRCA1,EIF4E,ZFP36 |
| GO:0005497 | Androgen binding | 2 | 4 | 0.016 | ALDH1A1,AR |
| GO:0008559 | ATPase-coupled xenobiotic transmembrane transporter activity | 2 | 4 | 0.016 | ABCC2,ABCC1 |
| GO:0050839 | Cell adhesion molecule binding | 11 | 538 | 0.016 | CDH1,EGFR,ADAM17,HSPA5,BSG,CTNNB1,HMGB1,STAT1,POSTN,SPP1,YWHAZ |
| GO:0004857 | Enzyme inhibitor activity | 9 | 377 | 0.0174 | SERPINE1,TIMP3,NOTCH1,BIRC5,XIAP,UGT1A1,CDKN1A,CDKN2A,BIRC2 |
| GO:0043027 | Cysteine-type endopeptidase inhibitor activity involved in apoptotic process | 3 | 25 | 0.0185 | BIRC5,XIAP,BIRC2 |
| GO:0070063 | RNA polymerase binding | 4 | 61 | 0.0189 | ERBB2,ERCC5,BRCA1,ZFP36 |
| GO:0005138 | interleukin-6 receptor binding | 2 | 5 | 0.0214 | ADAM17,IL6 |
| GO:0045569 | TRAIL binding | 2 | 5 | 0.0214 | TNFRSF10A,TNFSF10 |
| GO:0000978 | RNA polymerase II cis-regulatory region sequence-specific DNA binding | 12 | 672 | 0.0267 | EPAS1,STAT3,TP53,NOTCH1,EZH2,MTA1,STAT1,AR,TP73,NFE2L2,HIF1A,MUC1 |
| GO:0046914 | Transition metal ion binding | 16 | 1076 | 0.0267 | TNFSF10,SIRT2,MDM2,TP53,BIRC5,RARB,MTA1,S100A4,AR,BMI1,KDM4C,SIRT3,BRCA1,SOD2,BIRC2,CYP1B1 |
| GO:0031072 | Heat shock protein binding | 5 | 120 | 0.0274 | CSNK2A1,BAX,HSPA5,HIF1A,ZFP36 |
| GO:0043559 | Insulin binding | 2 | 6 | 0.0274 | IGF1R,HSPD1 |
| GO:0019956 | Chemokine binding | 3 | 30 | 0.0282 | CXCR2,HMGB1,ZFP36 |
| GO:0043548 | Phosphatidylinositol 3-kinase binding | 3 | 30 | 0.0282 | IGF1R,AXL,JAK2 |
| GO:0051721 | Protein phosphatase 2a binding | 3 | 32 | 0.033 | TP53,STAT1,BCL2 |
| GO:0019958 | C-X-C chemokine binding | 2 | 7 | 0.0337 | CXCR2,HMGB1 |
| GO:0031730 | CCR5 chemokine receptor binding | 2 | 7 | 0.0337 | STAT3,STAT1 |
| GO:0016209 | Antioxidant activity | 4 | 74 | 0.034 | PTGS2,GPX3,GSTP1,SOD2 |
| GO:0016787 | Hydrolase activity | 27 | 2419 | 0.037 | ERCC1,APEX1,HGF,MLH1,DUSP1,SIRT2,XRCC1,OGG1,ADAM17,ERCC4,USP9X,HSPA5,RAD51C,ERCC5,CASP8,ABCC2,PTEN,ABCC10,TLR4,RAD51,SIRT3,HSPD1,ERCC2,XRCC5,ABCC1,TOP2A,XRCC3 |
| GO:0001530 | Lipopolysaccharide binding | 3 | 34 | 0.0373 | HMGB1,TLR4,HSPD1 |
| GO:0016799 | Hydrolase activity, hydrolyzing n-glycosyl compounds | 3 | 34 | 0.0373 | APEX1,OGG1,TLR4 |
| GO:0043130 | Ubiquitin binding | 4 | 77 | 0.0379 | SIRT2,MDM2,TOP2A,BIRC2 |
| GO:0008270 | Zinc ion binding | 13 | 811 | 0.0384 | TNFSF10,SIRT2,MDM2,TP53,BIRC5,RARB,MTA1,AR,BMI1,KDM4C,SIRT3,BRCA1,BIRC2 |
| GO:0000014 | Single-stranded dna endodeoxyribonuclease activity | 2 | 8 | 0.0393 | XRCC1,ERCC4 |
| GO:0000405 | Bubble dna binding | 2 | 8 | 0.0393 | HMGB1,ERCC5 |
| GO:0000981 | DNA-binding transcription factor activity, RNA polymerase II-specific | 15 | 1022 | 0.0393 | EPAS1,STAT3,TP63,TP53,NOTCH1,EZH2,SOX2,RARB,E2F1,STAT1,AR,TP73,NFE2L2,RUNX3,HIF1A |
| GO:0001758 | Retinal dehydrogenase activity | 2 | 8 | 0.0393 | ALDH1A2,ALDH1A1 |
| GO:0004674 | Protein serine/threonine kinase activity | 9 | 437 | 0.0393 | MAPK1,CSNK2A1,PIK3CA,EGFR,PAK1,PRKDC,STK11,MTOR,PDK2 |
| GO:0020037 | Heme binding | 5 | 134 | 0.0393 | NOX4,NOS2,PTGS2,JAK2,CYP1B1 |
| GO:0031490 | Chromatin dna binding | 4 | 79 | 0.0393 | APEX1,STAT3,NOTCH1,EZH2 |
| GO:0032356 | Oxidized dna binding | 2 | 8 | 0.0393 | XRCC1,OGG1 |
| GO:0070851 | Growth factor receptor binding | 5 | 138 | 0.0426 | ADAM17,PTEN,JAK2,IL6,VEGFA |
| GO:0034511 | U3 snoRNA binding | 2 | 9 | 0.0449 | PRKDC,XRCC5 |

**S5: Molecular functions**

| #term ID | term description | observed gene count | background gene count | false discovery rate | matching proteins in your network (labels) |
| --- | --- | --- | --- | --- | --- |
| GO:0005515 | Protein binding | 106 | 7026 | 5.10E-29 | ERCC1,MAPK1,LGALS1,APEX1,CSNK2A1,TNFRSF10A,TGFB1,HGF,SERPINE1,CCND1,IFNG,MLH1,DUSP1,TNFSF10,BMP4,SIRT2,GDF15,MDM2,CDH1,XRCC1,NOX4,EPAS1,PIK3CA,STAT3,TP63,TIMP3,RB1,IGF1R,TP53,ERBB2,EGFR,NOTCH1,PAK1,BAX,AXL,BIRC5,OGG1,TERT,ADAM17,ERCC4,GSTM1,PRKDC,TYMS,USP9X,MET,CXCR2,HSPA5,STK11,NOS2,RARB,MTA1,BSG,CTNNB1,HMGB1,E2F1,ERCC5,FAS,CASP8,STAT1,MTOR,PTGS2,FASLG,HDGF,MKI67,S100A4,MCL1,ABCC2,XIAP,PTEN,UGT1A1,TLR4,AR,BMI1,TP73,POSTN,IGFBP3,KDM4C,JAK2,TP53BP1,RAD51,SIRT3,GPX3,HSPD1,ERCC2,XRCC5,SPP1,YWHAZ,NFE2L2,BCL2,GSTP1,CDKN1A,IL6,TNF,TOP2A,CDKN2A,BRCA1,PDK2,EIF4E,H2AFX,HIF1A,SLC16A1,SOD2,ZFP36,BIRC2,VEGFA,MUC1 |
| GO:0019899 | Enzyme binding | 57 | 2239 | 2.60E-19 | MAPK1,TNFRSF10A,TGFB1,SERPINE1,CCND1,MLH1,DUSP1,SIRT2,MDM2,XRCC1,NOX4,EPAS1,STAT3,TIMP3,RB1,TP53,ERBB2,EGFR,NOTCH1,PAK1,BIRC5,GSTM1,PRKDC,MET,HSPA5,MTA1,CTNNB1,HMGB1,E2F1,ERCC5,FAS,CASP8,STAT1,MTOR,PTGS2,PTEN,UGT1A1,AR,TP73,KDM4C,JAK2,RAD51,SIRT3,HSPD1,XRCC5,YWHAZ,BCL2,GSTP1,CDKN1A,TNF,TOP2A,CDKN2A,BRCA1,EIF4E,H2AFX,HIF1A,ZFP36 |
| GO:0042802 | Identical protein binding | 52 | 1896 | 1.29E-18 | MAPK1,LGALS1,CSNK2A1,TNFRSF10A,TGFB1,HGF,TNFSF10,GDF15,MDM2,CDH1,STAT3,TP63,RB1,IGF1R,TP53,ERBB2,EGFR,PAK1,BAX,BIRC5,TERT,ERCC4,GSTM1,TYMS,MET,NOS2,ERCC5,FAS,CASP8,STAT1,MTOR,PTGS2,S100A4,MCL1,XIAP,PTEN,UGT1A1,TLR4,TP73,JAK2,RAD51,GPX3,YWHAZ,BCL2,TNF,TOP2A,BRCA1,PDK2,SLC16A1,SOD2,BIRC2,VEGFA |
| GO:0005488 | Binding | 118 | 12516 | 1.19E-17 | ERCC1,MAPK1,LGALS1,APEX1,CSNK2A1,TNFRSF10A,TGFB1,HGF,SERPINE1,CCND1,IFNG,MLH1,DUSP1,TNFSF10,BMP4,SIRT2,ALDH1A2,GDF15,MDM2,CDH1,XRCC1,NOX4,EPAS1,PIK3CA,STAT3,TP63,TIMP3,RB1,IGF1R,TP53,ERBB2,EGFR,NOTCH1,PAK1,BAX,ALDH1A1,AXL,BIRC5,MGMT,OGG1,TERT,ADAM17,ERCC4,GSTM1,PRKDC,TYMS,USP9X,MET,CXCR2,EZH2,SOX2,HSPA5,STK11,NOS2,RARB,MTA1,BSG,RAD51C,CTNNB1,HMGB1,E2F1,ERCC5,FAS,CASP8,STAT1,MTOR,PTGS2,FASLG,HDGF,MKI67,S100A4,MCL1,ABCC2,XIAP,PTEN,ABCC10,UGT1A1,TLR4,AR,BMI1,TP73,POSTN,IGFBP3,KDM4C,JAK2,TP53BP1,RAD51,SIRT3,GPX3,HSPD1,ERCC2,XRCC5,SPP1,YWHAZ,NFE2L2,BCL2,GSTP1,ABCC1,RUNX3,CDKN1A,IL6,CD44,TNF,TOP2A,CDKN2A,BRCA1,PDK2,EIF4E,H2AFX,HIF1A,SLC16A1,SOD2,XRCC3,ZFP36,BIRC2,CYP1B1,VEGFA,MUC1 |
| GO:0008134 | Transcription factor binding | 33 | 672 | 1.19E-17 | ERCC1,MAPK1,APEX1,TNFRSF10A,CCND1,SIRT2,EPAS1,STAT3,RB1,TP53,TERT,ERCC4,PRKDC,RARB,MTA1,CTNNB1,HMGB1,E2F1,STAT1,MTOR,HDGF,AR,TP73,KDM4C,TP53BP1,GPX3,YWHAZ,NFE2L2,BCL2,CDKN2A,BRCA1,EIF4E,HIF1A |
| GO:0003677 | DNA binding | 49 | 2470 | 7.21E-12 | ERCC1,MAPK1,APEX1,MLH1,XRCC1,EPAS1,STAT3,TP63,RB1,TP53,EGFR,NOTCH1,MGMT,OGG1,TERT,ERCC4,PRKDC,EZH2,SOX2,RARB,MTA1,RAD51C,HMGB1,E2F1,ERCC5,STAT1,MTOR,HDGF,MKI67,AR,TP73,TP53BP1,RAD51,SIRT3,HSPD1,ERCC2,XRCC5,NFE2L2,BCL2,RUNX3,TNF,TOP2A,CDKN2A,BRCA1,H2AFX,HIF1A,XRCC3,ZFP36,MUC1 |
| GO:0097159 | Organic cyclic compound binding | 77 | 5916 | 1.07E-11 | ERCC1,MAPK1,LGALS1,APEX1,CSNK2A1,MLH1,SIRT2,MDM2,XRCC1,NOX4,EPAS1,PIK3CA,STAT3,TP63,RB1,IGF1R,TP53,ERBB2,EGFR,NOTCH1,PAK1,ALDH1A1,AXL,MGMT,OGG1,TERT,ERCC4,PRKDC,TYMS,MET,EZH2,SOX2,HSPA5,STK11,NOS2,RARB,MTA1,RAD51C,HMGB1,E2F1,ERCC5,STAT1,MTOR,PTGS2,HDGF,MKI67,S100A4,ABCC2,ABCC10,UGT1A1,AR,TP73,JAK2,TP53BP1,RAD51,SIRT3,HSPD1,ERCC2,XRCC5,YWHAZ,NFE2L2,BCL2,ABCC1,RUNX3,TNF,TOP2A,CDKN2A,BRCA1,PDK2,EIF4E,H2AFX,HIF1A,SLC16A1,XRCC3,ZFP36,CYP1B1,MUC1 |
| GO:0003684 | Damaged dna binding | 12 | 64 | 1.36E-11 | ERCC1,APEX1,XRCC1,TP63,OGG1,ERCC4,HMGB1,TP53BP1,ERCC2,XRCC5,BRCA1,H2AFX |
| GO:1901363 | Heterocyclic compound binding | 75 | 5831 | 5.87E-11 | ERCC1,MAPK1,LGALS1,APEX1,CSNK2A1,MLH1,SIRT2,MDM2,XRCC1,NOX4,EPAS1,PIK3CA,STAT3,TP63,RB1,IGF1R,TP53,ERBB2,EGFR,NOTCH1,PAK1,ALDH1A1,AXL,MGMT,OGG1,TERT,ERCC4,PRKDC,TYMS,MET,EZH2,SOX2,HSPA5,STK11,NOS2,RARB,MTA1,RAD51C,HMGB1,E2F1,ERCC5,STAT1,MTOR,PTGS2,HDGF,MKI67,S100A4,ABCC2,ABCC10,AR,TP73,JAK2,TP53BP1,RAD51,SIRT3,HSPD1,ERCC2,XRCC5,YWHAZ,NFE2L2,BCL2,ABCC1,RUNX3,TNF,TOP2A,CDKN2A,BRCA1,PDK2,EIF4E,H2AFX,HIF1A,XRCC3,ZFP36,CYP1B1,MUC1 |
| GO:0003690 | Double-stranded dna binding | 31 | 1156 | 9.22E-10 | MAPK1,APEX1,MLH1,EPAS1,STAT3,TP63,RB1,TP53,EGFR,NOTCH1,PRKDC,EZH2,SOX2,RARB,MTA1,HMGB1,E2F1,ERCC5,STAT1,MTOR,AR,TP73,RAD51,HSPD1,XRCC5,NFE2L2,RUNX3,TNF,BRCA1,HIF1A,MUC1 |
| GO:0005102 | Signaling receptor binding | 35 | 1581 | 4.64E-09 | TGFB1,HGF,SERPINE1,IFNG,TNFSF10,BMP4,GDF15,MDM2,STAT3,RB1,IGF1R,TP53,ERBB2,EGFR,NOTCH1,ADAM17,RARB,CTNNB1,HMGB1,CASP8,STAT1,FASLG,HDGF,S100A4,PTEN,TLR4,AR,KDM4C,JAK2,SPP1,IL6,TNF,BRCA1,HIF1A,VEGFA |
| GO:0019904 | Protein domain specific binding | 24 | 716 | 4.64E-09 | ERCC1,MDM2,TP63,RB1,TP53,BAX,ADAM17,PRKDC,HSPA5,STK11,CTNNB1,CASP8,MTOR,MCL1,ABCC2,PTEN,AR,BMI1,JAK2,YWHAZ,NFE2L2,BCL2,CDKN2A,HIF1A |
| GO:0002039 | p53 binding | 10 | 69 | 1.22E-08 | MDM2,TP63,TP53,STK11,TP73,TP53BP1,HSPD1,CDKN2A,HIF1A,MUC1 |
| GO:0098772 | Molecular function regulator | 51 | 3422 | 3.97E-08 | APEX1,TGFB1,HGF,SERPINE1,CCND1,IFNG,TNFSF10,BMP4,GDF15,EPAS1,PIK3CA,STAT3,TP63,TIMP3,RB1,TP53,NOTCH1,ALDH1A1,BIRC5,EZH2,SOX2,STK11,RARB,MTA1,CTNNB1,HMGB1,E2F1,STAT1,FASLG,HDGF,XIAP,UGT1A1,AR,TP73,IGFBP3,TP53BP1,XRCC5,SPP1,NFE2L2,BCL2,GSTP1,RUNX3,CDKN1A,IL6,TNF,CDKN2A,BRCA1,HIF1A,BIRC2,VEGFA,MUC1 |
| GO:0019900 | Kinase binding | 23 | 742 | 4.30E-08 | MAPK1,CCND1,DUSP1,NOX4,STAT3,RB1,TP53,EGFR,PAK1,CTNNB1,E2F1,FAS,MTOR,PTEN,TP73,JAK2,YWHAZ,GSTP1,CDKN1A,TOP2A,CDKN2A,HIF1A,ZFP36 |
| GO:0003676 | Nucleic acid binding | 55 | 3947 | 5.36E-08 | ERCC1,MAPK1,LGALS1,APEX1,MLH1,MDM2,XRCC1,EPAS1,STAT3,TP63,RB1,TP53,EGFR,NOTCH1,MGMT,OGG1,TERT,ERCC4,PRKDC,TYMS,EZH2,SOX2,RARB,MTA1,RAD51C,HMGB1,E2F1,ERCC5,STAT1,MTOR,HDGF,MKI67,S100A4,AR,TP73,TP53BP1,RAD51,SIRT3,HSPD1,ERCC2,XRCC5,YWHAZ,NFE2L2,BCL2,RUNX3,TNF,TOP2A,CDKN2A,BRCA1,EIF4E,H2AFX,HIF1A,XRCC3,ZFP36,MUC1 |
| GO:0036094 | Small molecule binding | 42 | 2516 | 1.05E-07 | MAPK1,CSNK2A1,MLH1,SIRT2,ALDH1A2,NOX4,PIK3CA,IGF1R,TP53,ERBB2,EGFR,PAK1,ALDH1A1,AXL,PRKDC,TYMS,MET,HSPA5,STK11,NOS2,BSG,RAD51C,MTOR,HDGF,MKI67,ABCC2,ABCC10,UGT1A1,JAK2,RAD51,SIRT3,HSPD1,ERCC2,XRCC5,GSTP1,ABCC1,RUNX3,CD44,TOP2A,PDK2,XRCC3,CYP1B1 |
| GO:0043167 | Ion binding | 71 | 6188 | 1.05E-07 | MAPK1,APEX1,CSNK2A1,MLH1,TNFSF10,BMP4,SIRT2,MDM2,CDH1,NOX4,PIK3CA,TP63,TIMP3,IGF1R,TP53,ERBB2,EGFR,NOTCH1,PAK1,AXL,BIRC5,MGMT,TERT,ADAM17,GSTM1,PRKDC,TYMS,MET,HSPA5,STK11,NOS2,RARB,MTA1,RAD51C,HMGB1,ERCC5,MTOR,PTGS2,HDGF,MKI67,S100A4,ABCC2,XIAP,ABCC10,UGT1A1,AR,BMI1,TP73,POSTN,IGFBP3,KDM4C,JAK2,RAD51,SIRT3,HSPD1,ERCC2,XRCC5,GSTP1,ABCC1,RUNX3,CDKN1A,CD44,TOP2A,BRCA1,PDK2,SOD2,XRCC3,ZFP36,BIRC2,CYP1B1,VEGFA |
| GO:0019901 | Protein kinase binding | 21 | 653 | 1.22E-07 | MAPK1,CCND1,DUSP1,NOX4,STAT3,TP53,EGFR,PAK1,CTNNB1,E2F1,MTOR,PTEN,TP73,JAK2,YWHAZ,GSTP1,CDKN1A,TOP2A,CDKN2A,HIF1A,ZFP36 |
| GO:0140297 | DNA-binding transcription factor binding | 16 | 366 | 2.37E-07 | APEX1,STAT3,RB1,RARB,MTA1,CTNNB1,HMGB1,STAT1,KDM4C,TP53BP1,NFE2L2,BCL2,CDKN2A,BRCA1,EIF4E,HIF1A |
| GO:0003824 | Catalytic activity | 65 | 5486 | 2.89E-07 | ERCC1,MAPK1,APEX1,CSNK2A1,HGF,CCND1,MLH1,DUSP1,SIRT2,ALDH1A2,MDM2,XRCC1,NOX4,PIK3CA,IGF1R,ERBB2,EGFR,PAK1,ALDH1A1,AXL,MGMT,OGG1,TERT,ADAM17,ERCC4,GSTM1,PRKDC,TYMS,USP9X,MET,EZH2,HSPA5,STK11,NOS2,RAD51C,HMGB1,ERCC5,CASP8,MTOR,PTGS2,ABCC2,XIAP,PTEN,ABCC10,UGT1A1,TLR4,KDM4C,JAK2,RAD51,SIRT3,GPX3,HSPD1,ERCC2,XRCC5,GSTP1,ABCC1,CDKN1A,TOP2A,CDKN2A,BRCA1,PDK2,SOD2,XRCC3,BIRC2,CYP1B1 |
| GO:0043565 | Sequence-specific dna binding | 29 | 1331 | 2.92E-07 | APEX1,EPAS1,STAT3,TP63,RB1,TP53,NOTCH1,TERT,EZH2,SOX2,RARB,MTA1,HMGB1,E2F1,STAT1,MTOR,AR,TP73,TP53BP1,SIRT3,HSPD1,XRCC5,NFE2L2,BCL2,RUNX3,TNF,BRCA1,HIF1A,MUC1 |
| GO:0003682 | Chromatin binding | 19 | 570 | 3.93E-07 | ERCC1,APEX1,MLH1,SIRT2,STAT3,TP63,TP53,EGFR,NOTCH1,ERCC4,EZH2,MTA1,CTNNB1,STAT1,AR,BMI1,KDM4C,RAD51,TOP2A |
| GO:0140097 | Catalytic activity, acting on dna | 12 | 184 | 3.93E-07 | APEX1,XRCC1,MGMT,OGG1,TERT,ERCC4,RAD51C,ERCC5,RAD51,ERCC2,XRCC5,TOP2A |
| GO:0043168 | Anion binding | 43 | 2805 | 5.72E-07 | MAPK1,CSNK2A1,MLH1,BMP4,SIRT2,NOX4,PIK3CA,IGF1R,TP53,ERBB2,EGFR,PAK1,AXL,GSTM1,PRKDC,TYMS,MET,HSPA5,STK11,NOS2,RAD51C,HMGB1,MTOR,HDGF,MKI67,ABCC2,ABCC10,UGT1A1,POSTN,JAK2,RAD51,SIRT3,HSPD1,ERCC2,XRCC5,GSTP1,ABCC1,RUNX3,CD44,TOP2A,PDK2,XRCC3,VEGFA |
| GO:0000166 | Nucleotide binding | 36 | 2119 | 1.25E-06 | MAPK1,CSNK2A1,MLH1,SIRT2,NOX4,PIK3CA,IGF1R,TP53,ERBB2,EGFR,PAK1,ALDH1A1,AXL,PRKDC,TYMS,MET,HSPA5,STK11,NOS2,RAD51C,MTOR,HDGF,MKI67,ABCC2,ABCC10,JAK2,RAD51,SIRT3,HSPD1,ERCC2,XRCC5,ABCC1,RUNX3,TOP2A,PDK2,XRCC3 |
| GO:0097367 | Carbohydrate derivative binding | 37 | 2226 | 1.25E-06 | MAPK1,CSNK2A1,MLH1,BMP4,PIK3CA,IGF1R,TP53,ERBB2,EGFR,PAK1,AXL,PRKDC,MET,HSPA5,STK11,NOS2,RAD51C,HMGB1,MTOR,HDGF,MKI67,ABCC2,ABCC10,TLR4,POSTN,JAK2,RAD51,HSPD1,ERCC2,XRCC5,ABCC1,RUNX3,CD44,TOP2A,PDK2,XRCC3,VEGFA |
| GO:0044389 | Ubiquitin-like protein ligase binding | 14 | 312 | 1.36E-06 | MDM2,RB1,TP53,EGFR,HSPA5,CASP8,STAT1,HSPD1,XRCC5,YWHAZ,BCL2,CDKN1A,BRCA1,HIF1A |
| GO:0005524 | ATP binding | 29 | 1464 | 1.75E-06 | MAPK1,CSNK2A1,MLH1,PIK3CA,IGF1R,TP53,ERBB2,EGFR,PAK1,AXL,PRKDC,MET,HSPA5,STK11,RAD51C,MTOR,MKI67,ABCC2,ABCC10,JAK2,RAD51,HSPD1,ERCC2,XRCC5,ABCC1,RUNX3,TOP2A,PDK2,XRCC3 |
| GO:0061629 | RNA polymerase II-specific DNA-binding transcription factor binding | 13 | 283 | 3.35E-06 | APEX1,STAT3,RB1,RARB,MTA1,CTNNB1,STAT1,KDM4C,TP53BP1,NFE2L2,CDKN2A,BRCA1,HIF1A |
| GO:1990837 | Sequence-specific double-stranded dna binding | 24 | 1068 | 3.69E-06 | EPAS1,STAT3,TP63,RB1,TP53,NOTCH1,EZH2,SOX2,RARB,MTA1,HMGB1,E2F1,STAT1,MTOR,AR,TP73,HSPD1,XRCC5,NFE2L2,RUNX3,TNF,BRCA1,HIF1A,MUC1 |
| GO:0031625 | Ubiquitin protein ligase binding | 13 | 296 | 4.92E-06 | MDM2,RB1,TP53,EGFR,HSPA5,CASP8,HSPD1,XRCC5,YWHAZ,BCL2,CDKN1A,BRCA1,HIF1A |
| GO:0008022 | Protein c-terminus binding | 11 | 199 | 5.92E-06 | ERCC1,ERBB2,TERT,ERCC4,CTNNB1,MKI67,JAK2,RAD51,ERCC2,XRCC5,TOP2A |
| GO:0000976 | Transcription regulatory region sequence-specific dna binding | 23 | 1028 | 6.98E-06 | EPAS1,STAT3,TP63,RB1,TP53,NOTCH1,EZH2,SOX2,RARB,MTA1,HMGB1,E2F1,STAT1,MTOR,AR,TP73,XRCC5,NFE2L2,RUNX3,TNF,BRCA1,HIF1A,MUC1 |
| GO:0046983 | Protein dimerization activity | 23 | 1037 | 7.72E-06 | GDF15,EPAS1,STAT3,TP53,ERBB2,BAX,BIRC5,TERT,GSTM1,NOS2,E2F1,ERCC5,STAT1,PTGS2,MCL1,UGT1A1,TLR4,BCL2,TOP2A,PDK2,H2AFX,HIF1A,VEGFA |
| GO:0044877 | Protein-containing complex binding | 25 | 1216 | 7.90E-06 | ERCC1,APEX1,HGF,CCND1,MDM2,IGF1R,TP53,EGFR,PAK1,ADAM17,ERCC4,EZH2,HSPA5,RARB,HMGB1,ERCC5,CASP8,MTOR,PTEN,HSPD1,XRCC5,SPP1,CDKN1A,CD44,BIRC2 |
| GO:0005126 | Cytokine receptor binding | 12 | 264 | 9.86E-06 | TGFB1,IFNG,TNFSF10,STAT3,ADAM17,CASP8,STAT1,FASLG,JAK2,IL6,TNF,VEGFA |
| GO:0005125 | Cytokine activity | 11 | 233 | 2.31E-05 | TGFB1,IFNG,TNFSF10,BMP4,GDF15,HMGB1,FASLG,SPP1,IL6,TNF,VEGFA |
| GO:0004672 | Protein kinase activity | 16 | 568 | 3.95E-05 | MAPK1,CSNK2A1,CCND1,PIK3CA,IGF1R,ERBB2,EGFR,PAK1,AXL,PRKDC,MET,STK11,MTOR,JAK2,CDKN1A,PDK2 |
| GO:0070491 | Repressing transcription factor binding | 7 | 73 | 4.24E-05 | STAT3,MTA1,CTNNB1,HMGB1,STAT1,BCL2,EIF4E |
| GO:0047485 | Protein n-terminus binding | 8 | 110 | 4.53E-05 | CSNK2A1,MDM2,TP53,TERT,ERCC4,ERCC5,ERCC2,BIRC2 |
| GO:0097371 | MDM2/MDM4 family protein binding | 4 | 9 | 6.33E-05 | TP63,TP53,TP73,CDKN2A |
| GO:0032553 | Ribonucleotide binding | 30 | 1880 | 6.34E-05 | MAPK1,CSNK2A1,MLH1,PIK3CA,IGF1R,TP53,ERBB2,EGFR,PAK1,AXL,PRKDC,MET,HSPA5,STK11,NOS2,RAD51C,MTOR,MKI67,ABCC2,ABCC10,JAK2,RAD51,HSPD1,ERCC2,XRCC5,ABCC1,RUNX3,TOP2A,PDK2,XRCC3 |
| GO:0046982 | Protein heterodimerization activity | 12 | 338 | 0.0001 | EPAS1,TP53,ERBB2,BAX,BIRC5,MCL1,UGT1A1,TLR4,BCL2,TOP2A,H2AFX,HIF1A |
| GO:0035035 | Histone acetyltransferase binding | 5 | 29 | 0.00012 | SIRT2,EPAS1,TP53,STAT1,HIF1A |
| GO:0005164 | Tumor necrosis factor receptor binding | 5 | 31 | 0.00016 | TNFSF10,CASP8,STAT1,FASLG,TNF |
| GO:0002020 | Protease binding | 8 | 138 | 0.00019 | TNFRSF10A,SERPINE1,TIMP3,TP53,PTEN,HSPD1,BCL2,TNF |
| GO:0051427 | Hormone receptor binding | 9 | 188 | 0.0002 | STAT3,RB1,RARB,CTNNB1,STAT1,KDM4C,JAK2,BRCA1,HIF1A |
| GO:0140096 | Catalytic activity, acting on a protein | 31 | 2116 | 0.0002 | MAPK1,CSNK2A1,HGF,CCND1,DUSP1,SIRT2,MDM2,PIK3CA,IGF1R,ERBB2,EGFR,PAK1,AXL,ADAM17,PRKDC,USP9X,MET,EZH2,STK11,CASP8,MTOR,XIAP,PTEN,KDM4C,JAK2,SIRT3,CDKN1A,CDKN2A,BRCA1,PDK2,BIRC2 |
| GO:1990841 | Promoter-specific chromatin binding | 6 | 61 | 0.0002 | ERCC1,TP53,ERCC4,EZH2,STAT1,BMI1 |
| GO:0097718 | Disordered domain specific binding | 5 | 34 | 0.00021 | MDM2,RB1,TP53,CTNNB1,CDKN2A |
| GO:0019902 | Phosphatase binding | 9 | 194 | 0.00023 | MAPK1,STAT3,TP53,ERBB2,EGFR,MET,CTNNB1,STAT1,BCL2 |
| GO:0042803 | Protein homodimerization activity | 16 | 673 | 0.00024 | GDF15,STAT3,BAX,BIRC5,TERT,GSTM1,NOS2,ERCC5,STAT1,PTGS2,MCL1,UGT1A1,BCL2,TOP2A,PDK2,VEGFA |
| GO:0004520 | Endodeoxyribonuclease activity | 5 | 36 | 0.00025 | APEX1,XRCC1,ERCC4,RAD51C,ERCC5 |
| GO:0019903 | Protein phosphatase binding | 8 | 149 | 0.00028 | STAT3,TP53,ERBB2,EGFR,MET,CTNNB1,STAT1,BCL2 |
| GO:0051087 | Chaperone binding | 7 | 105 | 0.00028 | TP53,BAX,BIRC5,TERT,HSPA5,HSPD1,BIRC2 |
| GO:0016887 | ATPase activity | 12 | 393 | 0.00035 | MLH1,HSPA5,RAD51C,ABCC2,ABCC10,RAD51,HSPD1,ERCC2,XRCC5,ABCC1,TOP2A,XRCC3 |
| GO:0035257 | Nuclear hormone receptor binding | 8 | 155 | 0.00035 | STAT3,RB1,RARB,CTNNB1,STAT1,KDM4C,BRCA1,HIF1A |
| GO:0140110 | Transcription regulator activity | 26 | 1657 | 0.00038 | APEX1,CCND1,EPAS1,STAT3,TP63,RB1,TP53,NOTCH1,EZH2,SOX2,RARB,MTA1,CTNNB1,HMGB1,E2F1,STAT1,HDGF,AR,TP73,TP53BP1,NFE2L2,RUNX3,BRCA1,HIF1A,BIRC2,MUC1 |
| GO:0140296 | General transcription initiation factor binding | 5 | 41 | 0.00041 | ERCC1,TP53,ERCC4,MTOR,AR |
| GO:0001091 | RNA polymerase II general transcription initiation factor binding | 4 | 19 | 0.0005 | ERCC1,TP53,ERCC4,AR |
| GO:0048018 | Receptor ligand activity | 13 | 490 | 0.00057 | TGFB1,HGF,IFNG,TNFSF10,BMP4,GDF15,HMGB1,FASLG,HDGF,SPP1,IL6,TNF,VEGFA |
| GO:0004519 | Endonuclease activity | 7 | 120 | 0.00058 | ERCC1,APEX1,XRCC1,OGG1,ERCC4,RAD51C,ERCC5 |
| GO:0016740 | Transferase activity | 30 | 2170 | 0.00071 | MAPK1,CSNK2A1,CCND1,SIRT2,MDM2,PIK3CA,IGF1R,ERBB2,EGFR,PAK1,AXL,MGMT,TERT,GSTM1,PRKDC,TYMS,MET,EZH2,STK11,MTOR,XIAP,UGT1A1,JAK2,SIRT3,GSTP1,CDKN1A,CDKN2A,BRCA1,PDK2,BIRC2 |
| GO:0051434 | BH3 domain binding | 3 | 6 | 0.00077 | BAX,MCL1,BCL2 |
| GO:0001216 | DNA-binding transcription activator activity | 12 | 452 | 0.0011 | EPAS1,STAT3,TP63,TP53,NOTCH1,SOX2,E2F1,STAT1,AR,TP73,NFE2L2,HIF1A |
| GO:0072341 | Modified amino acid binding | 6 | 90 | 0.0011 | NOX4,AXL,GSTM1,TYMS,HMGB1,GSTP1 |
| GO:0000987 | Cis-regulatory region sequence-specific dna binding | 15 | 701 | 0.0012 | EPAS1,STAT3,RB1,TP53,NOTCH1,EZH2,MTA1,E2F1,STAT1,MTOR,AR,TP73,NFE2L2,HIF1A,MUC1 |
| GO:0001099 | Basal rna polymerase ii transcription machinery binding | 5 | 54 | 0.0012 | ERCC1,TP53,ERCC4,ERCC5,AR |
| GO:0008094 | DNA-dependent ATPase activity | 6 | 93 | 0.0012 | RAD51C,RAD51,ERCC2,XRCC5,TOP2A,XRCC3 |
| GO:0016772 | Transferase activity, transferring phosphorus-containing groups | 17 | 875 | 0.0012 | MAPK1,CSNK2A1,CCND1,PIK3CA,IGF1R,ERBB2,EGFR,PAK1,AXL,TERT,PRKDC,MET,STK11,MTOR,JAK2,CDKN1A,PDK2 |
| GO:0004714 | Transmembrane receptor protein tyrosine kinase activity | 5 | 63 | 0.0022 | IGF1R,ERBB2,EGFR,AXL,MET |
| GO:0043560 | Insulin receptor substrate binding | 3 | 10 | 0.0022 | PIK3CA,IGF1R,JAK2 |
| GO:0001094 | TFIID-class transcription factor complex binding | 3 | 11 | 0.0028 | ERCC1,TP53,ERCC4 |
| GO:0008083 | Growth factor activity | 7 | 161 | 0.0028 | TGFB1,HGF,BMP4,GDF15,HDGF,IL6,VEGFA |
| GO:0003697 | Single-stranded dna binding | 6 | 113 | 0.0032 | ERCC1,ERCC4,HMGB1,ERCC5,RAD51,HSPD1 |
| GO:0016922 | Nuclear receptor binding | 6 | 114 | 0.0033 | STAT3,RB1,RARB,CTNNB1,KDM4C,BRCA1 |
| GO:0042826 | Histone deacetylase binding | 6 | 114 | 0.0033 | CCND1,SIRT2,TP53,MTA1,TOP2A,HIF1A |
| GO:0042162 | Telomeric dna binding | 4 | 36 | 0.0035 | APEX1,TERT,TP53BP1,XRCC5 |
| GO:0000977 | RNA polymerase II transcription regulatory region sequence-specific DNA binding | 16 | 878 | 0.0036 | EPAS1,STAT3,TP63,RB1,TP53,NOTCH1,EZH2,RARB,MTA1,STAT1,AR,TP73,NFE2L2,RUNX3,HIF1A,MUC1 |
| GO:0001228 | DNA-binding transcription activator activity, RNA polymerase II-specific | 11 | 449 | 0.004 | EPAS1,STAT3,TP63,TP53,NOTCH1,SOX2,STAT1,AR,TP73,NFE2L2,HIF1A |
| GO:0042056 | Chemoattractant activity | 4 | 41 | 0.0053 | HGF,BMP4,HMGB1,VEGFA |
| GO:0050681 | Androgen receptor binding | 4 | 44 | 0.0067 | RB1,CTNNB1,KDM4C,BRCA1 |
| GO:0003712 | Transcription coregulator activity | 12 | 571 | 0.0075 | APEX1,CCND1,RB1,EZH2,MTA1,CTNNB1,HMGB1,HDGF,TP53BP1,BRCA1,BIRC2,MUC1 |
| GO:0030234 | Enzyme regulator activity | 17 | 1044 | 0.0075 | SERPINE1,CCND1,PIK3CA,TIMP3,TP53,NOTCH1,ALDH1A1,BIRC5,STK11,XIAP,UGT1A1,IGFBP3,XRCC5,GSTP1,CDKN1A,CDKN2A,BIRC2 |
| GO:0042562 | Hormone binding | 5 | 86 | 0.0075 | IGF1R,EGFR,ALDH1A1,AR,HSPD1 |
| GO:0004713 | Protein tyrosine kinase activity | 6 | 137 | 0.0076 | IGF1R,ERBB2,EGFR,AXL,MET,JAK2 |
| GO:0035258 | Steroid hormone receptor binding | 5 | 93 | 0.0102 | STAT3,RB1,CTNNB1,KDM4C,BRCA1 |
| GO:0001102 | RNA polymerase II activating transcription factor binding | 4 | 53 | 0.0122 | RB1,CTNNB1,TP53BP1,NFE2L2 |
| GO:0003723 | RNA binding | 22 | 1649 | 0.0134 | LGALS1,APEX1,MDM2,STAT3,TP53,TERT,PRKDC,TYMS,EZH2,SOX2,HMGB1,HDGF,MKI67,S100A4,HSPD1,XRCC5,YWHAZ,TOP2A,CDKN2A,BRCA1,EIF4E,ZFP36 |
| GO:0005497 | Androgen binding | 2 | 4 | 0.016 | ALDH1A1,AR |
| GO:0008559 | ATPase-coupled xenobiotic transmembrane transporter activity | 2 | 4 | 0.016 | ABCC2,ABCC1 |
| GO:0050839 | Cell adhesion molecule binding | 11 | 538 | 0.016 | CDH1,EGFR,ADAM17,HSPA5,BSG,CTNNB1,HMGB1,STAT1,POSTN,SPP1,YWHAZ |
| GO:0004857 | Enzyme inhibitor activity | 9 | 377 | 0.0174 | SERPINE1,TIMP3,NOTCH1,BIRC5,XIAP,UGT1A1,CDKN1A,CDKN2A,BIRC2 |
| GO:0043027 | Cysteine-type endopeptidase inhibitor activity involved in apoptotic process | 3 | 25 | 0.0185 | BIRC5,XIAP,BIRC2 |
| GO:0070063 | RNA polymerase binding | 4 | 61 | 0.0189 | ERBB2,ERCC5,BRCA1,ZFP36 |
| GO:0005138 | interleukin-6 receptor binding | 2 | 5 | 0.0214 | ADAM17,IL6 |
| GO:0045569 | TRAIL binding | 2 | 5 | 0.0214 | TNFRSF10A,TNFSF10 |
| GO:0000978 | RNA polymerase II cis-regulatory region sequence-specific DNA binding | 12 | 672 | 0.0267 | EPAS1,STAT3,TP53,NOTCH1,EZH2,MTA1,STAT1,AR,TP73,NFE2L2,HIF1A,MUC1 |
| GO:0046914 | Transition metal ion binding | 16 | 1076 | 0.0267 | TNFSF10,SIRT2,MDM2,TP53,BIRC5,RARB,MTA1,S100A4,AR,BMI1,KDM4C,SIRT3,BRCA1,SOD2,BIRC2,CYP1B1 |
| GO:0031072 | Heat shock protein binding | 5 | 120 | 0.0274 | CSNK2A1,BAX,HSPA5,HIF1A,ZFP36 |
| GO:0043559 | Insulin binding | 2 | 6 | 0.0274 | IGF1R,HSPD1 |
| GO:0019956 | Chemokine binding | 3 | 30 | 0.0282 | CXCR2,HMGB1,ZFP36 |
| GO:0043548 | Phosphatidylinositol 3-kinase binding | 3 | 30 | 0.0282 | IGF1R,AXL,JAK2 |
| GO:0051721 | Protein phosphatase 2a binding | 3 | 32 | 0.033 | TP53,STAT1,BCL2 |
| GO:0019958 | C-X-C chemokine binding | 2 | 7 | 0.0337 | CXCR2,HMGB1 |
| GO:0031730 | CCR5 chemokine receptor binding | 2 | 7 | 0.0337 | STAT3,STAT1 |
| GO:0016209 | Antioxidant activity | 4 | 74 | 0.034 | PTGS2,GPX3,GSTP1,SOD2 |
| GO:0016787 | Hydrolase activity | 27 | 2419 | 0.037 | ERCC1,APEX1,HGF,MLH1,DUSP1,SIRT2,XRCC1,OGG1,ADAM17,ERCC4,USP9X,HSPA5,RAD51C,ERCC5,CASP8,ABCC2,PTEN,ABCC10,TLR4,RAD51,SIRT3,HSPD1,ERCC2,XRCC5,ABCC1,TOP2A,XRCC3 |
| GO:0001530 | Lipopolysaccharide binding | 3 | 34 | 0.0373 | HMGB1,TLR4,HSPD1 |
| GO:0016799 | Hydrolase activity, hydrolyzing n-glycosyl compounds | 3 | 34 | 0.0373 | APEX1,OGG1,TLR4 |
| GO:0043130 | Ubiquitin binding | 4 | 77 | 0.0379 | SIRT2,MDM2,TOP2A,BIRC2 |
| GO:0008270 | Zinc ion binding | 13 | 811 | 0.0384 | TNFSF10,SIRT2,MDM2,TP53,BIRC5,RARB,MTA1,AR,BMI1,KDM4C,SIRT3,BRCA1,BIRC2 |
| GO:0000014 | Single-stranded dna endodeoxyribonuclease activity | 2 | 8 | 0.0393 | XRCC1,ERCC4 |
| GO:0000405 | Bubble dna binding | 2 | 8 | 0.0393 | HMGB1,ERCC5 |
| GO:0000981 | DNA-binding transcription factor activity, RNA polymerase II-specific | 15 | 1022 | 0.0393 | EPAS1,STAT3,TP63,TP53,NOTCH1,EZH2,SOX2,RARB,E2F1,STAT1,AR,TP73,NFE2L2,RUNX3,HIF1A |
| GO:0001758 | Retinal dehydrogenase activity | 2 | 8 | 0.0393 | ALDH1A2,ALDH1A1 |
| GO:0004674 | Protein serine/threonine kinase activity | 9 | 437 | 0.0393 | MAPK1,CSNK2A1,PIK3CA,EGFR,PAK1,PRKDC,STK11,MTOR,PDK2 |
| GO:0020037 | Heme binding | 5 | 134 | 0.0393 | NOX4,NOS2,PTGS2,JAK2,CYP1B1 |
| GO:0031490 | Chromatin dna binding | 4 | 79 | 0.0393 | APEX1,STAT3,NOTCH1,EZH2 |
| GO:0032356 | Oxidized dna binding | 2 | 8 | 0.0393 | XRCC1,OGG1 |
| GO:0070851 | Growth factor receptor binding | 5 | 138 | 0.0426 | ADAM17,PTEN,JAK2,IL6,VEGFA |
| GO:0034511 | U3 snoRNA binding | 2 | 9 | 0.0449 | PRKDC,XRCC5 |

**S6: Biological Components**

| #term ID | term description | observed gene count | background gene count | false discovery rate | matching proteins in your network (labels) |
| --- | --- | --- | --- | --- | --- |
| GO:0010033 | Response to organic substance | 91 | 3011 | 1.83E-44 | ERCC1,MAPK1,LGALS1,APEX1,TGFB1,HGF,SERPINE1,CCND1,IFNG,DUSP1,TNFSF10,BMP4,SIRT2,ALDH1A2,GDF15,MDM2,CDH1,XRCC1,NOX4,PIK3CA,STAT3,TIMP3,RB1,IGF1R,TP53,ERBB2,EGFR,NOTCH1,PAK1,BAX,AXL,BIRC5,OGG1,ADAM17,PRKDC,TYMS,USP9X,CXCR2,EZH2,SOX2,HSPA5,NOS2,RARB,BSG,CTNNB1,HMGB1,E2F1,FAS,CASP8,STAT1,MTOR,PTGS2,FASLG,HDGF,MCL1,ABCC2,PTEN,UGT1A1,TLR4,AR,TP73,POSTN,CD274,JAK2,RAD51,GPX3,HSPD1,XRCC5,SPP1,YWHAZ,NFE2L2,BCL2,GSTP1,ABCC1,RUNX3,CDKN1A,IL6,CD44,TNF,BRCA1,PDK2,EIF4E,HIF1A,SLC16A1,SOD2,XRCC3,ZFP36,BIRC2,CYP1B1,VEGFA,MUC1 |
| GO:0010941 | Regulation of cell death | 75 | 1696 | 2.20E-44 | LGALS1,APEX1,CSNK2A1,TNFRSF10A,HGF,SERPINE1,IFNG,DUSP1,TNFSF10,BMP4,SIRT2,ALDH1A2,MDM2,NOX4,PIK3CA,STAT3,TP63,TIMP3,RB1,IGF1R,TP53,EGFR,NOTCH1,BAX,AXL,BIRC5,MGMT,OGG1,TERT,ADAM17,PRKDC,MET,CXCR2,SOX2,HSPA5,RARB,PDCD1,CTNNB1,HMGB1,E2F1,ERCC5,FAS,CASP8,STAT1,MTOR,PTGS2,FASLG,MCL1,XIAP,PTEN,TLR4,AR,TP73,IGFBP3,CD274,JAK2,HSPD1,YWHAZ,NFE2L2,BCL2,GSTP1,CDKN1A,IL6,CD44,TNF,TOP2A,CDKN2A,BRCA1,HIF1A,SOD2,ZFP36,BIRC2,CYP1B1,VEGFA,MUC1 |
| GO:0043067 | Regulation of programmed cell death | 73 | 1569 | 2.20E-44 | LGALS1,APEX1,CSNK2A1,TNFRSF10A,HGF,SERPINE1,IFNG,DUSP1,TNFSF10,BMP4,SIRT2,ALDH1A2,MDM2,NOX4,PIK3CA,TP63,TIMP3,RB1,IGF1R,TP53,EGFR,NOTCH1,BAX,AXL,BIRC5,MGMT,OGG1,TERT,ADAM17,PRKDC,MET,CXCR2,SOX2,HSPA5,RARB,PDCD1,CTNNB1,HMGB1,E2F1,ERCC5,FAS,CASP8,STAT1,MTOR,PTGS2,FASLG,MCL1,XIAP,PTEN,AR,TP73,IGFBP3,CD274,JAK2,HSPD1,YWHAZ,NFE2L2,BCL2,GSTP1,CDKN1A,IL6,CD44,TNF,TOP2A,CDKN2A,BRCA1,HIF1A,SOD2,ZFP36,BIRC2,CYP1B1,VEGFA,MUC1 |
| GO:0042981 | Regulation of apoptotic process | 72 | 1550 | 1.16E-43 | LGALS1,APEX1,CSNK2A1,TNFRSF10A,HGF,SERPINE1,IFNG,DUSP1,TNFSF10,BMP4,SIRT2,ALDH1A2,MDM2,NOX4,PIK3CA,TP63,TIMP3,RB1,IGF1R,TP53,EGFR,NOTCH1,BAX,AXL,BIRC5,MGMT,OGG1,TERT,ADAM17,PRKDC,CXCR2,SOX2,HSPA5,RARB,PDCD1,CTNNB1,HMGB1,E2F1,ERCC5,FAS,CASP8,STAT1,MTOR,PTGS2,FASLG,MCL1,XIAP,PTEN,AR,TP73,IGFBP3,CD274,JAK2,HSPD1,YWHAZ,NFE2L2,BCL2,GSTP1,CDKN1A,IL6,CD44,TNF,TOP2A,CDKN2A,BRCA1,HIF1A,SOD2,ZFP36,BIRC2,CYP1B1,VEGFA,MUC1 |
| GO:0048519 | Negative regulation of biological process | 108 | 5389 | 4.03E-43 | ERCC1,MAPK1,LGALS1,APEX1,CSNK2A1,TNFRSF10A,TGFB1,HGF,SERPINE1,CCND1,IFNG,MLH1,DUSP1,TNFSF10,BMP4,SIRT2,ALDH1A2,GDF15,MDM2,CDH1,XRCC1,NOX4,PIK3CA,STAT3,TP63,TIMP3,RB1,IGF1R,TP53,ERBB2,EGFR,NOTCH1,PAK1,BAX,ALDH1A1,AXL,BIRC5,MGMT,OGG1,TERT,ADAM17,ERCC4,PRKDC,TYMS,USP9X,MET,CXCR2,EZH2,SOX2,HSPA5,STK11,NOS2,RARB,MTA1,PDCD1,CTNNB1,HMGB1,E2F1,ERCC5,FAS,CASP8,STAT1,MTOR,PTGS2,FASLG,HDGF,MCL1,ABCC2,XIAP,PTEN,UGT1A1,TLR4,AR,BMI1,TP73,POSTN,IGFBP3,KDM4C,CD274,JAK2,TP53BP1,RAD51,SIRT3,HSPD1,XRCC5,SPP1,YWHAZ,NFE2L2,BCL2,GSTP1,RUNX3,CDKN1A,IL6,CD44,TNF,TOP2A,CDKN2A,BRCA1,PDK2,EIF4E,H2AFX,HIF1A,SOD2,ZFP36,BIRC2,CYP1B1,VEGFA,MUC1 |
| GO:0048523 | Negative regulation of cellular process | 104 | 4874 | 2.54E-42 | ERCC1,MAPK1,LGALS1,APEX1,CSNK2A1,TNFRSF10A,TGFB1,HGF,SERPINE1,CCND1,IFNG,MLH1,DUSP1,TNFSF10,BMP4,SIRT2,ALDH1A2,GDF15,MDM2,CDH1,XRCC1,NOX4,PIK3CA,STAT3,TP63,TIMP3,RB1,IGF1R,TP53,ERBB2,EGFR,NOTCH1,PAK1,BAX,AXL,BIRC5,MGMT,OGG1,TERT,ADAM17,ERCC4,PRKDC,TYMS,USP9X,MET,CXCR2,EZH2,SOX2,HSPA5,STK11,RARB,MTA1,PDCD1,CTNNB1,HMGB1,E2F1,ERCC5,FAS,CASP8,STAT1,MTOR,PTGS2,FASLG,HDGF,MCL1,XIAP,PTEN,UGT1A1,TLR4,AR,BMI1,TP73,POSTN,IGFBP3,KDM4C,CD274,JAK2,TP53BP1,RAD51,SIRT3,HSPD1,XRCC5,SPP1,YWHAZ,NFE2L2,BCL2,GSTP1,RUNX3,CDKN1A,IL6,CD44,TNF,TOP2A,CDKN2A,BRCA1,EIF4E,H2AFX,HIF1A,SOD2,ZFP36,BIRC2,CYP1B1,VEGFA,MUC1 |
| GO:0071310 | Cellular response to organic substance | 81 | 2369 | 1.12E-41 | MAPK1,LGALS1,APEX1,TGFB1,HGF,SERPINE1,CCND1,IFNG,DUSP1,BMP4,SIRT2,ALDH1A2,GDF15,MDM2,CDH1,NOX4,PIK3CA,STAT3,TIMP3,RB1,IGF1R,TP53,ERBB2,EGFR,NOTCH1,PAK1,BAX,AXL,BIRC5,ADAM17,PRKDC,USP9X,CXCR2,EZH2,SOX2,HSPA5,NOS2,RARB,CTNNB1,HMGB1,E2F1,FAS,CASP8,STAT1,MTOR,PTGS2,FASLG,HDGF,MCL1,ABCC2,PTEN,UGT1A1,TLR4,AR,POSTN,CD274,JAK2,RAD51,HSPD1,XRCC5,SPP1,YWHAZ,NFE2L2,BCL2,GSTP1,ABCC1,CDKN1A,IL6,CD44,TNF,BRCA1,PDK2,EIF4E,HIF1A,SLC16A1,SOD2,ZFP36,BIRC2,CYP1B1,VEGFA,MUC1 |
| GO:0070887 | Cellular response to chemical stimulus | 86 | 2919 | 1.47E-40 | MAPK1,LGALS1,APEX1,TGFB1,HGF,SERPINE1,CCND1,IFNG,DUSP1,BMP4,SIRT2,ALDH1A2,GDF15,MDM2,CDH1,NOX4,EPAS1,PIK3CA,STAT3,TIMP3,RB1,IGF1R,TP53,ERBB2,EGFR,NOTCH1,PAK1,BAX,AXL,BIRC5,OGG1,TERT,ADAM17,GSTM1,PRKDC,USP9X,CXCR2,EZH2,SOX2,HSPA5,NOS2,RARB,CTNNB1,HMGB1,E2F1,FAS,CASP8,STAT1,MTOR,PTGS2,FASLG,HDGF,MCL1,ABCC2,PTEN,UGT1A1,TLR4,AR,POSTN,CD274,JAK2,RAD51,GPX3,HSPD1,XRCC5,SPP1,YWHAZ,NFE2L2,BCL2,GSTP1,ABCC1,CDKN1A,IL6,CD44,TNF,BRCA1,PDK2,EIF4E,HIF1A,SLC16A1,SOD2,ZFP36,BIRC2,CYP1B1,VEGFA,MUC1 |
| GO:0048522 | Positive regulation of cellular process | 107 | 5579 | 1.60E-40 | ERCC1,MAPK1,LGALS1,APEX1,CSNK2A1,TNFRSF10A,TGFB1,HGF,SERPINE1,CCND1,IFNG,MLH1,DUSP1,TNFSF10,BMP4,SIRT2,ALDH1A2,GDF15,MDM2,CDH1,XRCC1,NOX4,EPAS1,PIK3CA,STAT3,TP63,TIMP3,RB1,IGF1R,TP53,ERBB2,EGFR,NOTCH1,PAK1,BAX,AXL,BIRC5,MGMT,TERT,ADAM17,ERCC4,PRKDC,USP9X,MET,CXCR2,EZH2,SOX2,HSPA5,STK11,NOS2,RARB,MTA1,PDCD1,RAD51C,CTNNB1,HMGB1,E2F1,ERCC5,FAS,CASP8,STAT1,MTOR,PTGS2,FASLG,HDGF,S100A4,MCL1,XIAP,PTEN,TLR4,AR,BMI1,TP73,POSTN,IGFBP3,KDM4C,CD274,JAK2,TP53BP1,RAD51,SIRT3,HSPD1,ERCC2,XRCC5,SPP1,YWHAZ,NFE2L2,BCL2,GSTP1,RUNX3,CDKN1A,IL6,CD44,TNF,TOP2A,CDKN2A,BRCA1,EIF4E,H2AFX,HIF1A,SOD2,XRCC3,ZFP36,BIRC2,CYP1B1,VEGFA,MUC1 |
| GO:0048518 | Positive regulation of biological process | 109 | 6112 | 3.89E-39 | ERCC1,MAPK1,LGALS1,APEX1,CSNK2A1,TNFRSF10A,TGFB1,HGF,SERPINE1,CCND1,IFNG,MLH1,DUSP1,TNFSF10,BMP4,SIRT2,ALDH1A2,GDF15,MDM2,CDH1,XRCC1,NOX4,EPAS1,PIK3CA,STAT3,TP63,TIMP3,RB1,IGF1R,TP53,ERBB2,EGFR,NOTCH1,PAK1,BAX,AXL,BIRC5,MGMT,TERT,ADAM17,ERCC4,PRKDC,USP9X,MET,CXCR2,EZH2,SOX2,HSPA5,STK11,NOS2,RARB,MTA1,BSG,PDCD1,RAD51C,CTNNB1,HMGB1,E2F1,ERCC5,FAS,CASP8,STAT1,MTOR,PTGS2,FASLG,HDGF,S100A4,MCL1,XIAP,PTEN,TLR4,AR,BMI1,TP73,POSTN,IGFBP3,KDM4C,CD274,JAK2,TP53BP1,RAD51,SIRT3,HSPD1,ERCC2,XRCC5,SPP1,YWHAZ,NFE2L2,BCL2,GSTP1,ABCC1,RUNX3,CDKN1A,IL6,CD44,TNF,TOP2A,CDKN2A,BRCA1,EIF4E,H2AFX,HIF1A,SOD2,XRCC3,ZFP36,BIRC2,CYP1B1,VEGFA,MUC1 |
| GO:0009893 | Positive regulation of metabolic process | 92 | 3893 | 2.90E-37 | ERCC1,MAPK1,APEX1,CSNK2A1,TNFRSF10A,TGFB1,HGF,SERPINE1,CCND1,IFNG,MLH1,TNFSF10,BMP4,SIRT2,ALDH1A2,GDF15,MDM2,CDH1,XRCC1,NOX4,EPAS1,PIK3CA,STAT3,TP63,RB1,IGF1R,TP53,ERBB2,EGFR,NOTCH1,PAK1,BAX,AXL,MGMT,TERT,ADAM17,PRKDC,USP9X,MET,EZH2,SOX2,HSPA5,STK11,NOS2,RARB,MTA1,BSG,CTNNB1,HMGB1,E2F1,FAS,CASP8,STAT1,MTOR,PTGS2,FASLG,XIAP,PTEN,TLR4,AR,BMI1,TP73,POSTN,IGFBP3,KDM4C,CD274,JAK2,TP53BP1,RAD51,SIRT3,HSPD1,ERCC2,XRCC5,SPP1,NFE2L2,BCL2,GSTP1,RUNX3,CDKN1A,IL6,CD44,TNF,TOP2A,CDKN2A,BRCA1,H2AFX,HIF1A,ZFP36,BIRC2,CYP1B1,VEGFA,MUC1 |
| GO:0006950 | Response to stress | 88 | 3485 | 7.49E-37 | ERCC1,MAPK1,LGALS1,APEX1,TGFB1,SERPINE1,CCND1,IFNG,MLH1,DUSP1,SIRT2,MDM2,XRCC1,NOX4,EPAS1,PIK3CA,STAT3,TP63,TP53,ERBB2,EGFR,NOTCH1,PAK1,BAX,AXL,MGMT,OGG1,TERT,ADAM17,ERCC4,GSTM1,PRKDC,CXCR2,EZH2,SOX2,HSPA5,STK11,NOS2,MTA1,RAD51C,HMGB1,E2F1,ERCC5,FAS,CASP8,STAT1,MTOR,PTGS2,FASLG,HDGF,MCL1,ABCC2,XIAP,PTEN,UGT1A1,TLR4,TP73,POSTN,JAK2,TP53BP1,RAD51,GPX3,HSPD1,ERCC2,XRCC5,SPP1,YWHAZ,NFE2L2,BCL2,GSTP1,CDKN1A,IL6,CD44,TNF,TOP2A,CDKN2A,BRCA1,PDK2,EIF4E,H2AFX,HIF1A,SOD2,XRCC3,ZFP36,BIRC2,CYP1B1,VEGFA,MUC1 |
| GO:1901700 | Response to oxygen-containing compound | 66 | 1567 | 8.12E-37 | ERCC1,MAPK1,LGALS1,APEX1,TGFB1,SERPINE1,CCND1,DUSP1,TNFSF10,SIRT2,ALDH1A2,MDM2,CDH1,XRCC1,NOX4,PIK3CA,STAT3,IGF1R,TP53,EGFR,NOTCH1,PAK1,AXL,OGG1,ADAM17,PRKDC,TYMS,EZH2,HSPA5,NOS2,BSG,CTNNB1,HMGB1,E2F1,CASP8,STAT1,MTOR,PTGS2,FASLG,ABCC2,PTEN,UGT1A1,TLR4,AR,POSTN,CD274,JAK2,RAD51,GPX3,HSPD1,XRCC5,SPP1,NFE2L2,BCL2,GSTP1,ABCC1,CDKN1A,IL6,TNF,BRCA1,PDK2,EIF4E,SOD2,ZFP36,BIRC2,CYP1B1 |
| GO:0042221 | Response to chemical | 95 | 4333 | 1.01E-36 | ERCC1,MAPK1,LGALS1,APEX1,TGFB1,HGF,SERPINE1,CCND1,IFNG,DUSP1,TNFSF10,BMP4,SIRT2,ALDH1A2,GDF15,MDM2,CDH1,XRCC1,NOX4,EPAS1,PIK3CA,STAT3,TIMP3,RB1,IGF1R,TP53,ERBB2,EGFR,NOTCH1,PAK1,BAX,AXL,BIRC5,OGG1,TERT,ADAM17,GSTM1,PRKDC,TYMS,USP9X,MET,CXCR2,EZH2,SOX2,HSPA5,NOS2,RARB,BSG,CTNNB1,HMGB1,E2F1,FAS,CASP8,STAT1,MTOR,PTGS2,FASLG,HDGF,MCL1,ABCC2,PTEN,UGT1A1,TLR4,AR,TP73,POSTN,CD274,JAK2,RAD51,GPX3,HSPD1,XRCC5,SPP1,YWHAZ,NFE2L2,BCL2,GSTP1,ABCC1,RUNX3,CDKN1A,IL6,CD44,TNF,BRCA1,PDK2,EIF4E,HIF1A,SLC16A1,SOD2,XRCC3,ZFP36,BIRC2,CYP1B1,VEGFA,MUC1 |
| GO:0051716 | Cellular response to stimulus | 109 | 6489 | 1.23E-36 | ERCC1,MAPK1,LGALS1,APEX1,CSNK2A1,TNFRSF10A,TGFB1,HGF,SERPINE1,CCND1,IFNG,MLH1,DUSP1,TNFSF10,BMP4,SIRT2,ALDH1A2,GDF15,MDM2,CDH1,XRCC1,NOX4,EPAS1,PIK3CA,STAT3,TP63,TIMP3,RB1,IGF1R,TP53,ERBB2,EGFR,NOTCH1,PAK1,BAX,AXL,BIRC5,MGMT,OGG1,TERT,ADAM17,ERCC4,GSTM1,PRKDC,USP9X,MET,CXCR2,EZH2,SOX2,HSPA5,STK11,NOS2,RARB,MTA1,BSG,RAD51C,CTNNB1,HMGB1,E2F1,ERCC5,FAS,CASP8,STAT1,MTOR,PTGS2,FASLG,HDGF,MCL1,ABCC2,XIAP,PTEN,UGT1A1,TLR4,AR,TP73,POSTN,CD274,JAK2,TP53BP1,RAD51,GPX3,HSPD1,ERCC2,XRCC5,SPP1,YWHAZ,NFE2L2,BCL2,GSTP1,ABCC1,CDKN1A,IL6,CD44,TNF,TOP2A,CDKN2A,BRCA1,PDK2,EIF4E,H2AFX,HIF1A,SLC16A1,SOD2,XRCC3,ZFP36,BIRC2,CYP1B1,VEGFA,MUC1 |
| GO:0010604 | Positive regulation of macromolecule metabolic process | 88 | 3600 | 7.53E-36 | ERCC1,MAPK1,APEX1,CSNK2A1,TNFRSF10A,TGFB1,HGF,SERPINE1,CCND1,IFNG,MLH1,TNFSF10,BMP4,SIRT2,ALDH1A2,GDF15,MDM2,CDH1,XRCC1,NOX4,EPAS1,PIK3CA,STAT3,TP63,RB1,IGF1R,TP53,ERBB2,EGFR,NOTCH1,PAK1,BAX,MGMT,TERT,ADAM17,PRKDC,USP9X,MET,EZH2,SOX2,HSPA5,STK11,RARB,MTA1,BSG,CTNNB1,HMGB1,E2F1,FAS,CASP8,STAT1,MTOR,PTGS2,FASLG,XIAP,PTEN,TLR4,AR,BMI1,TP73,POSTN,IGFBP3,KDM4C,CD274,JAK2,TP53BP1,RAD51,HSPD1,ERCC2,XRCC5,SPP1,NFE2L2,BCL2,RUNX3,CDKN1A,IL6,CD44,TNF,TOP2A,CDKN2A,BRCA1,H2AFX,HIF1A,ZFP36,BIRC2,CYP1B1,VEGFA,MUC1 |
| GO:0051173 | Positive regulation of nitrogen compound metabolic process | 84 | 3239 | 3.48E-35 | ERCC1,MAPK1,APEX1,CSNK2A1,TNFRSF10A,TGFB1,HGF,SERPINE1,CCND1,IFNG,MLH1,TNFSF10,BMP4,SIRT2,GDF15,MDM2,CDH1,XRCC1,NOX4,EPAS1,PIK3CA,STAT3,TP63,RB1,IGF1R,TP53,ERBB2,EGFR,NOTCH1,PAK1,BAX,MGMT,TERT,ADAM17,PRKDC,USP9X,MET,EZH2,SOX2,HSPA5,STK11,NOS2,RARB,MTA1,CTNNB1,HMGB1,E2F1,FAS,CASP8,STAT1,MTOR,PTGS2,FASLG,XIAP,PTEN,TLR4,AR,BMI1,TP73,IGFBP3,JAK2,TP53BP1,RAD51,SIRT3,HSPD1,ERCC2,XRCC5,SPP1,NFE2L2,BCL2,RUNX3,CDKN1A,IL6,CD44,TNF,TOP2A,CDKN2A,BRCA1,H2AFX,HIF1A,ZFP36,BIRC2,VEGFA,MUC1 |
| GO:0043069 | Negative regulation of programmed cell death | 53 | 908 | 1.05E-34 | CSNK2A1,TNFRSF10A,HGF,SERPINE1,DUSP1,TNFSF10,BMP4,MDM2,PIK3CA,RB1,IGF1R,TP53,EGFR,NOTCH1,BAX,AXL,BIRC5,MGMT,OGG1,TERT,PRKDC,MET,CXCR2,HSPA5,RARB,PDCD1,CTNNB1,ERCC5,FAS,CASP8,MTOR,PTGS2,FASLG,MCL1,XIAP,PTEN,AR,JAK2,HSPD1,YWHAZ,NFE2L2,BCL2,GSTP1,CDKN1A,IL6,CD44,TNF,BRCA1,HIF1A,SOD2,BIRC2,VEGFA,MUC1 |
| GO:0031325 | Positive regulation of cellular metabolic process | 85 | 3413 | 1.49E-34 | ERCC1,MAPK1,APEX1,TNFRSF10A,TGFB1,HGF,SERPINE1,CCND1,IFNG,MLH1,TNFSF10,BMP4,SIRT2,GDF15,MDM2,CDH1,XRCC1,NOX4,EPAS1,PIK3CA,STAT3,TP63,RB1,IGF1R,TP53,ERBB2,EGFR,NOTCH1,PAK1,BAX,AXL,MGMT,TERT,ADAM17,PRKDC,USP9X,MET,EZH2,SOX2,HSPA5,STK11,NOS2,RARB,MTA1,CTNNB1,HMGB1,E2F1,FAS,CASP8,STAT1,MTOR,PTGS2,FASLG,XIAP,PTEN,TLR4,AR,BMI1,TP73,IGFBP3,JAK2,TP53BP1,RAD51,SIRT3,HSPD1,ERCC2,XRCC5,SPP1,NFE2L2,BCL2,GSTP1,RUNX3,CDKN1A,IL6,CD44,TNF,TOP2A,CDKN2A,BRCA1,H2AFX,HIF1A,ZFP36,BIRC2,VEGFA,MUC1 |
| GO:0043066 | Negative regulation of apoptotic process | 52 | 893 | 6.57E-34 | CSNK2A1,TNFRSF10A,HGF,SERPINE1,DUSP1,TNFSF10,BMP4,MDM2,PIK3CA,RB1,IGF1R,TP53,EGFR,NOTCH1,BAX,AXL,BIRC5,MGMT,OGG1,TERT,PRKDC,CXCR2,HSPA5,RARB,PDCD1,CTNNB1,ERCC5,FAS,CASP8,MTOR,PTGS2,FASLG,MCL1,XIAP,PTEN,AR,JAK2,HSPD1,YWHAZ,NFE2L2,BCL2,GSTP1,CDKN1A,IL6,CD44,TNF,BRCA1,HIF1A,SOD2,BIRC2,VEGFA,MUC1 |
| GO:0060548 | Negative regulation of cell death | 54 | 999 | 6.57E-34 | CSNK2A1,TNFRSF10A,HGF,SERPINE1,DUSP1,TNFSF10,BMP4,MDM2,PIK3CA,STAT3,RB1,IGF1R,TP53,EGFR,NOTCH1,BAX,AXL,BIRC5,MGMT,OGG1,TERT,PRKDC,MET,CXCR2,HSPA5,RARB,PDCD1,CTNNB1,ERCC5,FAS,CASP8,MTOR,PTGS2,FASLG,MCL1,XIAP,PTEN,AR,JAK2,HSPD1,YWHAZ,NFE2L2,BCL2,GSTP1,CDKN1A,IL6,CD44,TNF,BRCA1,HIF1A,SOD2,BIRC2,VEGFA,MUC1 |
| GO:0048584 | Positive regulation of response to stimulus | 72 | 2257 | 1.07E-33 | MAPK1,LGALS1,CSNK2A1,TNFRSF10A,TGFB1,HGF,SERPINE1,IFNG,MLH1,TNFSF10,BMP4,GDF15,XRCC1,NOX4,PIK3CA,STAT3,TP63,TIMP3,IGF1R,TP53,ERBB2,EGFR,NOTCH1,PAK1,BAX,AXL,MGMT,TERT,ADAM17,PRKDC,MET,CXCR2,EZH2,SOX2,STK11,CTNNB1,HMGB1,E2F1,FAS,CASP8,MTOR,PTGS2,FASLG,S100A4,MCL1,XIAP,PTEN,TLR4,AR,TP73,IGFBP3,CD274,JAK2,TP53BP1,SIRT3,HSPD1,XRCC5,YWHAZ,NFE2L2,BCL2,ABCC1,IL6,CD44,TNF,CDKN2A,BRCA1,H2AFX,HIF1A,BIRC2,CYP1B1,VEGFA,MUC1 |
| GO:0009892 | Negative regulation of metabolic process | 81 | 3124 | 2.35E-33 | ERCC1,APEX1,CSNK2A1,TGFB1,HGF,SERPINE1,CCND1,IFNG,MLH1,DUSP1,BMP4,SIRT2,MDM2,XRCC1,PIK3CA,STAT3,TP63,TIMP3,RB1,IGF1R,TP53,EGFR,NOTCH1,BAX,ALDH1A1,AXL,BIRC5,OGG1,TERT,ADAM17,ERCC4,PRKDC,TYMS,USP9X,MET,EZH2,SOX2,STK11,NOS2,RARB,MTA1,CTNNB1,HMGB1,E2F1,STAT1,MTOR,PTGS2,FASLG,HDGF,MCL1,ABCC2,XIAP,PTEN,UGT1A1,TLR4,AR,BMI1,IGFBP3,KDM4C,CD274,TP53BP1,SIRT3,HSPD1,XRCC5,BCL2,GSTP1,RUNX3,CDKN1A,IL6,CD44,TNF,CDKN2A,BRCA1,PDK2,EIF4E,H2AFX,HIF1A,ZFP36,BIRC2,VEGFA,MUC1 |
| GO:0042127 | Regulation of cell population proliferation | 63 | 1642 | 1.34E-32 | MAPK1,CSNK2A1,TGFB1,CCND1,IFNG,DUSP1,BMP4,SIRT2,ALDH1A2,MDM2,NOX4,STAT3,TP63,RB1,IGF1R,TP53,ERBB2,EGFR,NOTCH1,PAK1,BAX,BIRC5,TERT,ADAM17,PRKDC,CXCR2,EZH2,SOX2,STK11,NOS2,RARB,CTNNB1,HMGB1,E2F1,STAT1,MTOR,PTGS2,FASLG,XIAP,PTEN,TLR4,AR,BMI1,TP73,IGFBP3,KDM4C,CD274,JAK2,XRCC5,BCL2,GSTP1,RUNX3,CDKN1A,IL6,TNF,CDKN2A,BRCA1,HIF1A,SOD2,ZFP36,BIRC2,CYP1B1,VEGFA |
| GO:0033554 | Cellular response to stress | 64 | 1725 | 1.84E-32 | ERCC1,MAPK1,APEX1,CCND1,MLH1,SIRT2,MDM2,XRCC1,NOX4,EPAS1,TP63,TP53,EGFR,NOTCH1,PAK1,BAX,AXL,MGMT,OGG1,TERT,ERCC4,GSTM1,PRKDC,EZH2,HSPA5,STK11,MTA1,RAD51C,HMGB1,E2F1,ERCC5,FAS,MTOR,PTGS2,HDGF,MCL1,XIAP,PTEN,TP73,JAK2,TP53BP1,RAD51,GPX3,HSPD1,ERCC2,XRCC5,NFE2L2,BCL2,GSTP1,CDKN1A,IL6,TNF,TOP2A,CDKN2A,BRCA1,PDK2,H2AFX,HIF1A,SOD2,XRCC3,ZFP36,CYP1B1,VEGFA,MUC1 |
| GO:0048583 | Regulation of response to stimulus | 89 | 4114 | 1.84E-32 | ERCC1,MAPK1,LGALS1,CSNK2A1,TNFRSF10A,TGFB1,HGF,SERPINE1,IFNG,MLH1,DUSP1,TNFSF10,BMP4,SIRT2,GDF15,MDM2,XRCC1,NOX4,PIK3CA,STAT3,TP63,TIMP3,RB1,IGF1R,TP53,ERBB2,EGFR,NOTCH1,PAK1,BAX,AXL,MGMT,OGG1,TERT,ADAM17,ERCC4,PRKDC,MET,CXCR2,EZH2,SOX2,HSPA5,STK11,NOS2,PDCD1,CTNNB1,HMGB1,E2F1,FAS,CASP8,STAT1,MTOR,PTGS2,FASLG,S100A4,MCL1,XIAP,PTEN,TLR4,AR,TP73,POSTN,IGFBP3,CD274,JAK2,TP53BP1,RAD51,SIRT3,HSPD1,XRCC5,SPP1,YWHAZ,NFE2L2,BCL2,GSTP1,ABCC1,IL6,CD44,TNF,CDKN2A,BRCA1,PDK2,H2AFX,HIF1A,SOD2,BIRC2,CYP1B1,VEGFA,MUC1 |
| GO:0019222 | Regulation of metabolic process | 107 | 6948 | 1.83E-31 | ERCC1,MAPK1,APEX1,CSNK2A1,TNFRSF10A,TGFB1,HGF,SERPINE1,CCND1,IFNG,MLH1,DUSP1,TNFSF10,BMP4,SIRT2,ALDH1A2,GDF15,MDM2,CDH1,XRCC1,NOX4,EPAS1,PIK3CA,STAT3,TP63,TIMP3,RB1,IGF1R,TP53,ERBB2,EGFR,NOTCH1,PAK1,BAX,ALDH1A1,AXL,BIRC5,MGMT,OGG1,TERT,ADAM17,ERCC4,PRKDC,TYMS,USP9X,MET,EZH2,SOX2,HSPA5,STK11,NOS2,RARB,MTA1,BSG,CTNNB1,HMGB1,E2F1,FAS,CASP8,STAT1,MTOR,PTGS2,FASLG,HDGF,MCL1,ABCC2,XIAP,PTEN,UGT1A1,TLR4,AR,BMI1,TP73,POSTN,IGFBP3,KDM4C,CD274,JAK2,TP53BP1,RAD51,SIRT3,HSPD1,ERCC2,XRCC5,SPP1,YWHAZ,NFE2L2,BCL2,GSTP1,RUNX3,CDKN1A,IL6,CD44,TNF,TOP2A,CDKN2A,BRCA1,PDK2,EIF4E,H2AFX,HIF1A,SOD2,ZFP36,BIRC2,CYP1B1,VEGFA,MUC1 |
| GO:0050896 | Response to stimulus | 112 | 8046 | 5.31E-31 | ERCC1,MAPK1,LGALS1,APEX1,CSNK2A1,TNFRSF10A,TGFB1,HGF,SERPINE1,CCND1,IFNG,MLH1,DUSP1,TNFSF10,BMP4,SIRT2,ALDH1A2,GDF15,MDM2,CDH1,XRCC1,NOX4,EPAS1,PIK3CA,STAT3,TP63,TIMP3,RB1,IGF1R,TP53,ERBB2,EGFR,NOTCH1,PAK1,BAX,AXL,BIRC5,MGMT,OGG1,TERT,ADAM17,ERCC4,GSTM1,PRKDC,TYMS,USP9X,MET,CXCR2,EZH2,SOX2,HSPA5,STK11,NOS2,RARB,MTA1,BSG,PDCD1,RAD51C,CTNNB1,HMGB1,E2F1,ERCC5,FAS,CASP8,STAT1,MTOR,PTGS2,FASLG,HDGF,MCL1,ABCC2,XIAP,PTEN,UGT1A1,TLR4,AR,TP73,POSTN,CD274,JAK2,TP53BP1,RAD51,GPX3,HSPD1,ERCC2,XRCC5,SPP1,YWHAZ,NFE2L2,BCL2,GSTP1,ABCC1,RUNX3,CDKN1A,IL6,CD44,TNF,TOP2A,CDKN2A,BRCA1,PDK2,EIF4E,H2AFX,HIF1A,SLC16A1,SOD2,XRCC3,ZFP36,BIRC2,CYP1B1,VEGFA,MUC1 |
| GO:0060255 | Regulation of macromolecule metabolic process | 103 | 6407 | 1.99E-30 | ERCC1,MAPK1,APEX1,CSNK2A1,TNFRSF10A,TGFB1,HGF,SERPINE1,CCND1,IFNG,MLH1,DUSP1,TNFSF10,BMP4,SIRT2,ALDH1A2,GDF15,MDM2,CDH1,XRCC1,NOX4,EPAS1,PIK3CA,STAT3,TP63,TIMP3,RB1,IGF1R,TP53,ERBB2,EGFR,NOTCH1,PAK1,BAX,AXL,BIRC5,MGMT,OGG1,TERT,ADAM17,ERCC4,PRKDC,TYMS,USP9X,MET,EZH2,SOX2,HSPA5,STK11,NOS2,RARB,MTA1,BSG,CTNNB1,HMGB1,E2F1,FAS,CASP8,STAT1,MTOR,PTGS2,FASLG,HDGF,ABCC2,XIAP,PTEN,TLR4,AR,BMI1,TP73,POSTN,IGFBP3,KDM4C,CD274,JAK2,TP53BP1,RAD51,SIRT3,HSPD1,ERCC2,XRCC5,SPP1,YWHAZ,NFE2L2,BCL2,GSTP1,RUNX3,CDKN1A,IL6,CD44,TNF,TOP2A,CDKN2A,BRCA1,EIF4E,H2AFX,HIF1A,SOD2,ZFP36,BIRC2,CYP1B1,VEGFA,MUC1 |
| GO:0009719 | Response to endogenous stimulus | 58 | 1447 | 2.24E-30 | MAPK1,APEX1,TGFB1,CCND1,DUSP1,TNFSF10,BMP4,SIRT2,GDF15,MDM2,CDH1,NOX4,PIK3CA,STAT3,TIMP3,RB1,IGF1R,TP53,ERBB2,EGFR,NOTCH1,PAK1,PRKDC,TYMS,USP9X,EZH2,HSPA5,NOS2,RARB,BSG,CTNNB1,E2F1,STAT1,MTOR,PTGS2,ABCC2,PTEN,UGT1A1,TLR4,AR,POSTN,JAK2,RAD51,HSPD1,SPP1,NFE2L2,BCL2,GSTP1,ABCC1,RUNX3,CDKN1A,IL6,CD44,TNF,BRCA1,PDK2,EIF4E,ZFP36 |
| GO:0031324 | Negative regulation of cellular metabolic process | 72 | 2630 | 1.40E-29 | ERCC1,APEX1,CSNK2A1,TGFB1,HGF,SERPINE1,CCND1,IFNG,MLH1,DUSP1,BMP4,SIRT2,MDM2,XRCC1,PIK3CA,STAT3,TP63,TIMP3,RB1,IGF1R,TP53,NOTCH1,BAX,BIRC5,OGG1,TERT,ERCC4,PRKDC,TYMS,USP9X,MET,EZH2,SOX2,RARB,MTA1,CTNNB1,HMGB1,E2F1,STAT1,MTOR,PTGS2,FASLG,HDGF,MCL1,XIAP,PTEN,UGT1A1,TLR4,AR,BMI1,IGFBP3,KDM4C,TP53BP1,SIRT3,HSPD1,XRCC5,BCL2,GSTP1,RUNX3,CDKN1A,IL6,CD44,TNF,CDKN2A,BRCA1,EIF4E,H2AFX,HIF1A,ZFP36,BIRC2,VEGFA,MUC1 |
| GO:0051246 | Regulation of protein metabolic process | 74 | 2828 | 1.64E-29 | MAPK1,CSNK2A1,TNFRSF10A,TGFB1,HGF,SERPINE1,CCND1,IFNG,DUSP1,TNFSF10,BMP4,SIRT2,GDF15,MDM2,CDH1,XRCC1,NOX4,PIK3CA,STAT3,TP63,TIMP3,RB1,IGF1R,TP53,ERBB2,EGFR,NOTCH1,PAK1,BAX,BIRC5,ADAM17,PRKDC,TYMS,EZH2,SOX2,HSPA5,STK11,NOS2,MTA1,CTNNB1,HMGB1,FAS,CASP8,MTOR,PTGS2,FASLG,XIAP,PTEN,TLR4,AR,BMI1,TP73,IGFBP3,KDM4C,JAK2,RAD51,SIRT3,HSPD1,XRCC5,YWHAZ,NFE2L2,BCL2,GSTP1,CDKN1A,IL6,CD44,TNF,CDKN2A,BRCA1,EIF4E,ZFP36,BIRC2,VEGFA,MUC1 |
| GO:0080134 | Regulation of response to stress | 57 | 1437 | 1.64E-29 | ERCC1,MAPK1,HGF,SERPINE1,IFNG,DUSP1,SIRT2,MDM2,XRCC1,STAT3,TP63,RB1,IGF1R,TP53,EGFR,PAK1,BAX,MGMT,OGG1,TERT,ERCC4,PRKDC,MET,HSPA5,NOS2,CTNNB1,HMGB1,FAS,STAT1,MTOR,PTGS2,MCL1,XIAP,PTEN,TLR4,JAK2,TP53BP1,RAD51,SIRT3,HSPD1,XRCC5,SPP1,NFE2L2,BCL2,GSTP1,ABCC1,IL6,CD44,TNF,CDKN2A,BRCA1,H2AFX,HIF1A,SOD2,BIRC2,VEGFA,MUC1 |
| GO:0031323 | Regulation of cellular metabolic process | 101 | 6239 | 1.91E-29 | ERCC1,MAPK1,APEX1,CSNK2A1,TNFRSF10A,TGFB1,HGF,SERPINE1,CCND1,IFNG,MLH1,DUSP1,TNFSF10,BMP4,SIRT2,GDF15,MDM2,CDH1,XRCC1,NOX4,EPAS1,PIK3CA,STAT3,TP63,TIMP3,RB1,IGF1R,TP53,ERBB2,EGFR,NOTCH1,PAK1,BAX,AXL,BIRC5,MGMT,OGG1,TERT,ADAM17,ERCC4,PRKDC,TYMS,USP9X,MET,EZH2,SOX2,HSPA5,STK11,NOS2,RARB,MTA1,CTNNB1,HMGB1,E2F1,FAS,CASP8,STAT1,MTOR,PTGS2,FASLG,HDGF,MCL1,XIAP,PTEN,UGT1A1,TLR4,AR,BMI1,TP73,IGFBP3,KDM4C,JAK2,TP53BP1,RAD51,SIRT3,HSPD1,ERCC2,XRCC5,SPP1,YWHAZ,NFE2L2,BCL2,GSTP1,RUNX3,CDKN1A,IL6,CD44,TNF,TOP2A,CDKN2A,BRCA1,PDK2,EIF4E,H2AFX,HIF1A,SOD2,ZFP36,BIRC2,CYP1B1,VEGFA,MUC1 |
| GO:0051171 | Regulation of nitrogen compound metabolic process | 98 | 5836 | 5.52E-29 | ERCC1,MAPK1,APEX1,CSNK2A1,TNFRSF10A,TGFB1,HGF,SERPINE1,CCND1,IFNG,MLH1,DUSP1,TNFSF10,BMP4,SIRT2,GDF15,MDM2,CDH1,XRCC1,NOX4,EPAS1,PIK3CA,STAT3,TP63,TIMP3,RB1,IGF1R,TP53,ERBB2,EGFR,NOTCH1,PAK1,BAX,BIRC5,MGMT,OGG1,TERT,ADAM17,ERCC4,PRKDC,TYMS,USP9X,MET,EZH2,SOX2,HSPA5,STK11,NOS2,RARB,MTA1,CTNNB1,HMGB1,E2F1,FAS,CASP8,STAT1,MTOR,PTGS2,FASLG,HDGF,XIAP,PTEN,TLR4,AR,BMI1,TP73,IGFBP3,KDM4C,JAK2,TP53BP1,RAD51,SIRT3,HSPD1,ERCC2,XRCC5,SPP1,YWHAZ,NFE2L2,BCL2,GSTP1,RUNX3,CDKN1A,IL6,CD44,TNF,TOP2A,CDKN2A,BRCA1,PDK2,EIF4E,H2AFX,HIF1A,SOD2,ZFP36,BIRC2,CYP1B1,VEGFA,MUC1 |
| GO:0080090 | Regulation of primary metabolic process | 99 | 6032 | 9.74E-29 | ERCC1,MAPK1,APEX1,CSNK2A1,TNFRSF10A,TGFB1,HGF,SERPINE1,CCND1,IFNG,MLH1,DUSP1,TNFSF10,BMP4,SIRT2,GDF15,MDM2,CDH1,XRCC1,NOX4,EPAS1,PIK3CA,STAT3,TP63,TIMP3,RB1,IGF1R,TP53,ERBB2,EGFR,NOTCH1,PAK1,BAX,BIRC5,MGMT,OGG1,TERT,ADAM17,ERCC4,PRKDC,TYMS,USP9X,MET,EZH2,SOX2,HSPA5,STK11,NOS2,RARB,MTA1,CTNNB1,HMGB1,E2F1,FAS,CASP8,STAT1,MTOR,PTGS2,FASLG,HDGF,XIAP,PTEN,UGT1A1,TLR4,AR,BMI1,TP73,IGFBP3,KDM4C,JAK2,TP53BP1,RAD51,SIRT3,HSPD1,ERCC2,XRCC5,SPP1,YWHAZ,NFE2L2,BCL2,GSTP1,RUNX3,CDKN1A,IL6,CD44,TNF,TOP2A,CDKN2A,BRCA1,PDK2,EIF4E,H2AFX,HIF1A,SOD2,ZFP36,BIRC2,CYP1B1,VEGFA,MUC1 |
| GO:0051094 | Positive regulation of developmental process | 55 | 1389 | 3.08E-28 | MAPK1,TGFB1,HGF,SERPINE1,CCND1,IFNG,MLH1,BMP4,SIRT2,GDF15,MDM2,STAT3,TP63,RB1,TP53,NOTCH1,PAK1,BAX,AXL,TERT,PRKDC,CXCR2,EZH2,SOX2,HSPA5,STK11,RARB,CTNNB1,HMGB1,E2F1,CASP8,STAT1,MTOR,PTGS2,PTEN,AR,TP73,IGFBP3,KDM4C,CD274,JAK2,TP53BP1,XRCC5,NFE2L2,BCL2,RUNX3,IL6,TNF,CDKN2A,BRCA1,HIF1A,SOD2,ZFP36,CYP1B1,VEGFA |
| GO:0014070 | Response to organic cyclic compound | 47 | 911 | 4.37E-28 | MAPK1,LGALS1,APEX1,TGFB1,CCND1,DUSP1,BMP4,SIRT2,ALDH1A2,MDM2,CDH1,NOX4,STAT3,RB1,EGFR,NOTCH1,OGG1,TYMS,EZH2,HSPA5,RARB,BSG,CTNNB1,CASP8,STAT1,MTOR,PTGS2,ABCC2,PTEN,UGT1A1,AR,POSTN,JAK2,RAD51,HSPD1,SPP1,BCL2,GSTP1,CDKN1A,IL6,TNF,BRCA1,EIF4E,SLC16A1,ZFP36,BIRC2,CYP1B1 |
| GO:0032268 | Regulation of cellular protein metabolic process | 71 | 2693 | 4.54E-28 | MAPK1,CSNK2A1,TNFRSF10A,TGFB1,HGF,SERPINE1,CCND1,IFNG,DUSP1,TNFSF10,BMP4,SIRT2,GDF15,MDM2,XRCC1,NOX4,PIK3CA,STAT3,TIMP3,RB1,IGF1R,TP53,ERBB2,EGFR,NOTCH1,PAK1,BAX,BIRC5,ADAM17,PRKDC,TYMS,EZH2,SOX2,HSPA5,STK11,MTA1,CTNNB1,HMGB1,FAS,CASP8,MTOR,PTGS2,FASLG,XIAP,PTEN,TLR4,AR,BMI1,TP73,IGFBP3,KDM4C,JAK2,RAD51,SIRT3,HSPD1,XRCC5,YWHAZ,NFE2L2,BCL2,GSTP1,CDKN1A,IL6,CD44,TNF,CDKN2A,BRCA1,EIF4E,ZFP36,BIRC2,VEGFA,MUC1 |
| GO:0048585 | Negative regulation of response to stimulus | 58 | 1636 | 9.62E-28 | ERCC1,CSNK2A1,TNFRSF10A,TGFB1,HGF,SERPINE1,DUSP1,TNFSF10,BMP4,SIRT2,GDF15,MDM2,XRCC1,TP63,TIMP3,RB1,IGF1R,TP53,ERBB2,EGFR,NOTCH1,BAX,OGG1,TERT,ADAM17,ERCC4,PRKDC,MET,EZH2,SOX2,HSPA5,STK11,PDCD1,CTNNB1,FAS,CASP8,STAT1,MTOR,PTGS2,FASLG,MCL1,PTEN,TLR4,AR,IGFBP3,TP53BP1,SIRT3,SPP1,NFE2L2,BCL2,GSTP1,IL6,CD44,TNF,BRCA1,HIF1A,SOD2,MUC1 |
| GO:0050790 | Regulation of catalytic activity | 67 | 2386 | 1.75E-27 | MAPK1,CSNK2A1,TNFRSF10A,TGFB1,HGF,SERPINE1,CCND1,IFNG,DUSP1,TNFSF10,BMP4,GDF15,MDM2,XRCC1,NOX4,PIK3CA,STAT3,TP63,TIMP3,RB1,IGF1R,TP53,ERBB2,EGFR,NOTCH1,PAK1,BAX,ALDH1A1,AXL,BIRC5,TERT,ADAM17,ERCC4,MET,EZH2,SOX2,STK11,NOS2,CTNNB1,HMGB1,FAS,CASP8,MTOR,PTGS2,FASLG,XIAP,PTEN,UGT1A1,TLR4,BMI1,TP73,IGFBP3,JAK2,SIRT3,HSPD1,XRCC5,BCL2,GSTP1,CDKN1A,CD44,TNF,CDKN2A,PDK2,HIF1A,ZFP36,BIRC2,VEGFA |
| GO:1902531 | Regulation of intracellular signal transduction | 60 | 1807 | 1.75E-27 | MAPK1,LGALS1,CSNK2A1,TNFRSF10A,TGFB1,HGF,DUSP1,TNFSF10,BMP4,GDF15,MDM2,NOX4,PIK3CA,TP63,TIMP3,IGF1R,TP53,ERBB2,EGFR,NOTCH1,PAK1,BAX,AXL,MET,EZH2,SOX2,STK11,CTNNB1,HMGB1,FAS,CASP8,STAT1,MTOR,PTGS2,FASLG,S100A4,MCL1,XIAP,PTEN,TLR4,AR,TP73,IGFBP3,JAK2,SIRT3,YWHAZ,NFE2L2,BCL2,GSTP1,IL6,CD44,TNF,CDKN2A,BRCA1,PDK2,HIF1A,SOD2,BIRC2,VEGFA,MUC1 |
| GO:0080135 | Regulation of cellular response to stress | 43 | 739 | 2.35E-27 | ERCC1,MAPK1,HGF,DUSP1,MDM2,XRCC1,TP63,IGF1R,TP53,EGFR,PAK1,BAX,MGMT,OGG1,TERT,ERCC4,PRKDC,MET,HSPA5,CTNNB1,HMGB1,FAS,MTOR,PTGS2,MCL1,PTEN,TLR4,TP53BP1,RAD51,SIRT3,SPP1,NFE2L2,BCL2,GSTP1,CD44,TNF,CDKN2A,BRCA1,H2AFX,HIF1A,SOD2,VEGFA,MUC1 |
| GO:0051172 | Negative regulation of nitrogen compound metabolic process | 67 | 2429 | 4.67E-27 | ERCC1,APEX1,CSNK2A1,TGFB1,HGF,SERPINE1,CCND1,IFNG,MLH1,DUSP1,BMP4,SIRT2,MDM2,XRCC1,STAT3,TP63,TIMP3,RB1,IGF1R,TP53,EGFR,NOTCH1,BAX,BIRC5,OGG1,TERT,ERCC4,PRKDC,TYMS,USP9X,EZH2,SOX2,NOS2,RARB,MTA1,CTNNB1,HMGB1,E2F1,STAT1,MTOR,PTGS2,FASLG,HDGF,XIAP,PTEN,TLR4,AR,BMI1,IGFBP3,KDM4C,TP53BP1,SIRT3,XRCC5,GSTP1,RUNX3,CDKN1A,IL6,CD44,TNF,CDKN2A,BRCA1,EIF4E,H2AFX,ZFP36,BIRC2,VEGFA,MUC1 |
| GO:0006915 | Apoptotic process | 46 | 918 | 6.31E-27 | MAPK1,LGALS1,CSNK2A1,TNFRSF10A,TGFB1,IFNG,MLH1,TNFSF10,PIK3CA,TP63,RB1,TP53,NOTCH1,PAK1,BAX,BIRC5,PRKDC,HSPA5,STK11,PDCD1,HMGB1,E2F1,FAS,CASP8,MTOR,FASLG,MCL1,XIAP,PTEN,TLR4,TP73,IGFBP3,JAK2,HSPD1,ERCC2,BCL2,CDKN1A,IL6,TNF,TOP2A,CDKN2A,BRCA1,PDK2,SOD2,BIRC2,CYP1B1 |
| GO:0051239 | Regulation of multicellular organismal process | 75 | 3227 | 7.50E-27 | MAPK1,LGALS1,TGFB1,HGF,SERPINE1,CCND1,IFNG,MLH1,BMP4,SIRT2,GDF15,MDM2,EPAS1,PIK3CA,STAT3,TP63,RB1,IGF1R,TP53,ERBB2,EGFR,NOTCH1,PAK1,BAX,ALDH1A1,AXL,TERT,ADAM17,PRKDC,MET,CXCR2,EZH2,SOX2,HSPA5,STK11,NOS2,RARB,BSG,PDCD1,CTNNB1,HMGB1,E2F1,CASP8,STAT1,MTOR,PTGS2,FASLG,PTEN,TLR4,AR,TP73,POSTN,KDM4C,CD274,JAK2,TP53BP1,HSPD1,XRCC5,SPP1,YWHAZ,NFE2L2,BCL2,GSTP1,RUNX3,IL6,TNF,CDKN2A,BRCA1,EIF4E,HIF1A,SOD2,ZFP36,BIRC2,CYP1B1,VEGFA |
| GO:0032270 | Positive regulation of cellular protein metabolic process | 57 | 1635 | 7.79E-27 | MAPK1,TNFRSF10A,TGFB1,HGF,CCND1,IFNG,TNFSF10,BMP4,SIRT2,GDF15,MDM2,NOX4,PIK3CA,STAT3,RB1,IGF1R,TP53,ERBB2,EGFR,NOTCH1,PAK1,BAX,ADAM17,PRKDC,EZH2,SOX2,HSPA5,STK11,MTA1,CTNNB1,HMGB1,FAS,CASP8,MTOR,PTGS2,FASLG,XIAP,PTEN,TLR4,AR,BMI1,TP73,IGFBP3,JAK2,HSPD1,XRCC5,NFE2L2,BCL2,CDKN1A,IL6,CD44,TNF,CDKN2A,BRCA1,BIRC2,VEGFA,MUC1 |
| GO:0051247 | Positive regulation of protein metabolic process | 58 | 1715 | 9.02E-27 | MAPK1,CSNK2A1,TNFRSF10A,TGFB1,HGF,CCND1,IFNG,TNFSF10,BMP4,SIRT2,GDF15,MDM2,NOX4,PIK3CA,STAT3,RB1,IGF1R,TP53,ERBB2,EGFR,NOTCH1,PAK1,BAX,ADAM17,PRKDC,EZH2,SOX2,HSPA5,STK11,MTA1,CTNNB1,HMGB1,FAS,CASP8,MTOR,PTGS2,FASLG,XIAP,PTEN,TLR4,AR,BMI1,TP73,IGFBP3,JAK2,HSPD1,XRCC5,NFE2L2,BCL2,CDKN1A,IL6,CD44,TNF,CDKN2A,BRCA1,BIRC2,VEGFA,MUC1 |
| GO:0010942 | Positive regulation of cell death | 42 | 719 | 9.66E-27 | TNFRSF10A,IFNG,DUSP1,TNFSF10,BMP4,SIRT2,ALDH1A2,NOX4,TP63,TIMP3,TP53,NOTCH1,BAX,PRKDC,CXCR2,RARB,PDCD1,CTNNB1,HMGB1,E2F1,FAS,CASP8,MTOR,PTGS2,FASLG,MCL1,PTEN,TLR4,TP73,IGFBP3,CD274,JAK2,HSPD1,YWHAZ,BCL2,CDKN1A,IL6,TNF,TOP2A,CDKN2A,SOD2,CYP1B1 |
| GO:0050793 | Regulation of developmental process | 69 | 2648 | 9.66E-27 | MAPK1,LGALS1,TGFB1,HGF,SERPINE1,CCND1,IFNG,MLH1,BMP4,SIRT2,GDF15,MDM2,PIK3CA,STAT3,TP63,RB1,TP53,ERBB2,EGFR,NOTCH1,PAK1,BAX,AXL,TERT,PRKDC,CXCR2,EZH2,SOX2,HSPA5,STK11,RARB,PDCD1,CTNNB1,HMGB1,E2F1,CASP8,STAT1,MTOR,PTGS2,FASLG,PTEN,TLR4,AR,TP73,POSTN,IGFBP3,KDM4C,CD274,JAK2,TP53BP1,XRCC5,SPP1,YWHAZ,NFE2L2,BCL2,RUNX3,CDKN1A,IL6,CD44,TNF,CDKN2A,BRCA1,EIF4E,HIF1A,SOD2,ZFP36,BIRC2,CYP1B1,VEGFA |
| GO:1901701 | Cellular response to oxygen-containing compound | 48 | 1055 | 1.42E-26 | MAPK1,LGALS1,APEX1,TGFB1,SERPINE1,SIRT2,ALDH1A2,MDM2,CDH1,NOX4,PIK3CA,STAT3,IGF1R,TP53,EGFR,PAK1,AXL,PRKDC,EZH2,HSPA5,NOS2,CTNNB1,HMGB1,E2F1,STAT1,MTOR,PTGS2,ABCC2,PTEN,UGT1A1,TLR4,AR,CD274,JAK2,RAD51,XRCC5,SPP1,NFE2L2,GSTP1,ABCC1,IL6,TNF,BRCA1,PDK2,EIF4E,SOD2,ZFP36,CYP1B1 |
| GO:0010605 | Negative regulation of macromolecule metabolic process | 71 | 2875 | 1.98E-26 | ERCC1,APEX1,CSNK2A1,TGFB1,HGF,SERPINE1,CCND1,IFNG,MLH1,DUSP1,BMP4,SIRT2,MDM2,XRCC1,STAT3,TP63,TIMP3,RB1,IGF1R,TP53,EGFR,NOTCH1,BAX,AXL,BIRC5,OGG1,TERT,ERCC4,PRKDC,TYMS,USP9X,EZH2,SOX2,NOS2,RARB,MTA1,CTNNB1,HMGB1,E2F1,STAT1,MTOR,PTGS2,FASLG,HDGF,ABCC2,XIAP,PTEN,TLR4,AR,BMI1,IGFBP3,KDM4C,CD274,TP53BP1,SIRT3,XRCC5,GSTP1,RUNX3,CDKN1A,IL6,CD44,TNF,CDKN2A,BRCA1,EIF4E,H2AFX,HIF1A,ZFP36,BIRC2,VEGFA,MUC1 |
| GO:0051240 | Positive regulation of multicellular organismal process | 58 | 1770 | 4.10E-26 | MAPK1,TGFB1,HGF,SERPINE1,CCND1,IFNG,MLH1,BMP4,SIRT2,EPAS1,STAT3,TP63,RB1,IGF1R,EGFR,NOTCH1,PAK1,BAX,AXL,TERT,ADAM17,PRKDC,MET,CXCR2,EZH2,HSPA5,STK11,RARB,BSG,CTNNB1,HMGB1,E2F1,CASP8,STAT1,MTOR,PTGS2,PTEN,TLR4,AR,TP73,POSTN,KDM4C,CD274,JAK2,TP53BP1,HSPD1,XRCC5,SPP1,NFE2L2,BCL2,RUNX3,IL6,TNF,BRCA1,HIF1A,SOD2,CYP1B1,VEGFA |
| GO:0033993 | Response to lipid | 44 | 858 | 5.05E-26 | MAPK1,TGFB1,SERPINE1,CCND1,DUSP1,BMP4,ALDH1A2,MDM2,STAT3,RB1,EGFR,NOTCH1,AXL,OGG1,ADAM17,TYMS,EZH2,NOS2,RARB,CTNNB1,HMGB1,E2F1,CASP8,PTGS2,FASLG,ABCC2,PTEN,UGT1A1,TLR4,AR,POSTN,CD274,JAK2,HSPD1,XRCC5,SPP1,BCL2,GSTP1,CDKN1A,IL6,TNF,BRCA1,EIF4E,ZFP36 |
| GO:0031399 | Regulation of protein modification process | 59 | 1870 | 7.36E-26 | MAPK1,TNFRSF10A,TGFB1,HGF,CCND1,IFNG,DUSP1,BMP4,SIRT2,GDF15,XRCC1,NOX4,PIK3CA,STAT3,TIMP3,RB1,IGF1R,TP53,ERBB2,EGFR,NOTCH1,PAK1,BAX,ADAM17,PRKDC,EZH2,SOX2,HSPA5,STK11,MTA1,CTNNB1,HMGB1,FAS,MTOR,PTGS2,XIAP,PTEN,TLR4,AR,BMI1,TP73,IGFBP3,KDM4C,JAK2,RAD51,SIRT3,XRCC5,YWHAZ,BCL2,GSTP1,CDKN1A,IL6,CD44,TNF,CDKN2A,BRCA1,BIRC2,VEGFA,MUC1 |
| GO:0051093 | Negative regulation of developmental process | 46 | 983 | 8.50E-26 | MAPK1,LGALS1,TGFB1,SERPINE1,CCND1,IFNG,BMP4,SIRT2,GDF15,MDM2,STAT3,TP63,RB1,TP53,ERBB2,EGFR,NOTCH1,PAK1,TERT,PRKDC,EZH2,SOX2,STK11,RARB,PDCD1,CTNNB1,HMGB1,E2F1,STAT1,FASLG,PTEN,TLR4,TP73,POSTN,SPP1,NFE2L2,BCL2,RUNX3,CDKN1A,IL6,TNF,CDKN2A,EIF4E,HIF1A,ZFP36,VEGFA |
| GO:0043068 | Positive regulation of programmed cell death | 40 | 666 | 9.04E-26 | TNFRSF10A,IFNG,DUSP1,TNFSF10,BMP4,SIRT2,ALDH1A2,NOX4,TP63,TIMP3,TP53,NOTCH1,BAX,PRKDC,CXCR2,RARB,PDCD1,CTNNB1,HMGB1,E2F1,FAS,CASP8,PTGS2,FASLG,MCL1,PTEN,TP73,IGFBP3,CD274,JAK2,HSPD1,YWHAZ,BCL2,CDKN1A,IL6,TNF,TOP2A,CDKN2A,SOD2,CYP1B1 |
| GO:2001233 | Regulation of apoptotic signaling pathway | 34 | 409 | 9.48E-26 | CSNK2A1,TNFRSF10A,HGF,SERPINE1,TNFSF10,BMP4,MDM2,TP63,TIMP3,RB1,TP53,BAX,TERT,CTNNB1,E2F1,FAS,CASP8,PTGS2,FASLG,MCL1,PTEN,AR,TP73,JAK2,YWHAZ,NFE2L2,BCL2,GSTP1,CD44,TNF,BRCA1,HIF1A,SOD2,MUC1 |
| GO:0010647 | Positive regulation of cell communication | 58 | 1823 | 1.66E-25 | MAPK1,LGALS1,CSNK2A1,TNFRSF10A,TGFB1,HGF,IFNG,TNFSF10,BMP4,GDF15,NOX4,PIK3CA,STAT3,TP63,TIMP3,IGF1R,TP53,ERBB2,EGFR,NOTCH1,PAK1,BAX,AXL,TERT,ADAM17,MET,EZH2,SOX2,STK11,CTNNB1,HMGB1,E2F1,FAS,CASP8,MTOR,PTGS2,FASLG,S100A4,MCL1,XIAP,PTEN,TLR4,AR,TP73,IGFBP3,JAK2,SIRT3,SPP1,YWHAZ,BCL2,IL6,CD44,TNF,CDKN2A,HIF1A,BIRC2,CYP1B1,VEGFA |
| GO:0023056 | Positive regulation of signaling | 58 | 1831 | 2.03E-25 | MAPK1,LGALS1,CSNK2A1,TNFRSF10A,TGFB1,HGF,IFNG,TNFSF10,BMP4,GDF15,NOX4,PIK3CA,STAT3,TP63,TIMP3,IGF1R,TP53,ERBB2,EGFR,NOTCH1,PAK1,BAX,AXL,TERT,ADAM17,MET,EZH2,SOX2,STK11,CTNNB1,HMGB1,E2F1,FAS,CASP8,MTOR,PTGS2,FASLG,S100A4,MCL1,XIAP,PTEN,TLR4,AR,TP73,IGFBP3,JAK2,SIRT3,SPP1,YWHAZ,BCL2,IL6,CD44,TNF,CDKN2A,HIF1A,BIRC2,CYP1B1,VEGFA |
| GO:2000026 | Regulation of multicellular organismal development | 61 | 2096 | 3.51E-25 | MAPK1,LGALS1,TGFB1,HGF,SERPINE1,CCND1,IFNG,MLH1,BMP4,SIRT2,MDM2,STAT3,TP63,RB1,TP53,ERBB2,EGFR,NOTCH1,PAK1,BAX,AXL,TERT,PRKDC,CXCR2,EZH2,SOX2,HSPA5,STK11,RARB,PDCD1,CTNNB1,HMGB1,E2F1,CASP8,STAT1,MTOR,PTGS2,FASLG,PTEN,TLR4,AR,TP73,KDM4C,CD274,TP53BP1,XRCC5,SPP1,YWHAZ,NFE2L2,BCL2,RUNX3,IL6,TNF,CDKN2A,BRCA1,EIF4E,HIF1A,SOD2,ZFP36,CYP1B1,VEGFA |
| GO:0009628 | Response to abiotic stimulus | 48 | 1147 | 4.17E-25 | ERCC1,TNFRSF10A,CCND1,DUSP1,SIRT2,MDM2,XRCC1,NOX4,EPAS1,TP53,EGFR,NOTCH1,PAK1,BAX,OGG1,TERT,ADAM17,ERCC4,PRKDC,HSPA5,STK11,NOS2,MTA1,E2F1,ERCC5,FAS,CASP8,STAT1,MTOR,PTGS2,ABCC2,PTEN,TLR4,POSTN,TP53BP1,RAD51,HSPD1,ERCC2,XRCC5,NFE2L2,BCL2,CDKN1A,TNF,BRCA1,H2AFX,HIF1A,BIRC2,VEGFA |
| GO:0065008 | Regulation of biological quality | 80 | 4042 | 6.66E-25 | ERCC1,MAPK1,LGALS1,APEX1,TGFB1,SERPINE1,CCND1,IFNG,BMP4,ALDH1A2,MDM2,XRCC1,NOX4,EPAS1,PIK3CA,STAT3,TP63,RB1,IGF1R,TP53,EGFR,NOTCH1,PAK1,BAX,ALDH1A1,AXL,TERT,ADAM17,ERCC4,PRKDC,USP9X,MET,CXCR2,EZH2,HSPA5,STK11,NOS2,RAD51C,CTNNB1,HMGB1,E2F1,FAS,CASP8,STAT1,MTOR,PTGS2,FASLG,MCL1,ABCC2,XIAP,PTEN,UGT1A1,TLR4,AR,TP73,POSTN,JAK2,RAD51,SIRT3,HSPD1,ERCC2,XRCC5,SPP1,YWHAZ,NFE2L2,BCL2,GSTP1,ABCC1,CDKN1A,IL6,TNF,CDKN2A,PDK2,HIF1A,SLC16A1,SOD2,XRCC3,ZFP36,CYP1B1,VEGFA |
| GO:0034097 | Response to cytokine | 47 | 1101 | 7.50E-25 | MAPK1,TGFB1,HGF,CCND1,IFNG,DUSP1,ALDH1A2,PIK3CA,STAT3,TIMP3,TP53,AXL,BIRC5,ADAM17,TYMS,CXCR2,SOX2,HSPA5,NOS2,FAS,CASP8,STAT1,PTGS2,FASLG,HDGF,MCL1,ABCC2,TLR4,POSTN,CD274,JAK2,HSPD1,XRCC5,YWHAZ,NFE2L2,BCL2,CDKN1A,IL6,CD44,TNF,BRCA1,HIF1A,SOD2,ZFP36,BIRC2,VEGFA,MUC1 |
| GO:0043065 | Positive regulation of apoptotic process | 39 | 660 | 7.68E-25 | TNFRSF10A,IFNG,DUSP1,TNFSF10,BMP4,SIRT2,ALDH1A2,NOX4,TP63,TIMP3,TP53,NOTCH1,BAX,PRKDC,CXCR2,RARB,PDCD1,CTNNB1,HMGB1,E2F1,FAS,CASP8,PTGS2,FASLG,MCL1,PTEN,TP73,IGFBP3,CD274,JAK2,HSPD1,YWHAZ,BCL2,IL6,TNF,TOP2A,CDKN2A,SOD2,CYP1B1 |
| GO:0009967 | Positive regulation of signal transduction | 55 | 1654 | 8.48E-25 | MAPK1,LGALS1,CSNK2A1,TNFRSF10A,TGFB1,HGF,IFNG,TNFSF10,BMP4,GDF15,NOX4,PIK3CA,STAT3,TP63,TIMP3,IGF1R,TP53,ERBB2,EGFR,NOTCH1,PAK1,BAX,AXL,TERT,ADAM17,MET,EZH2,SOX2,STK11,CTNNB1,HMGB1,E2F1,FAS,CASP8,MTOR,FASLG,S100A4,MCL1,XIAP,PTEN,TLR4,AR,TP73,IGFBP3,JAK2,YWHAZ,BCL2,IL6,CD44,TNF,CDKN2A,HIF1A,BIRC2,CYP1B1,VEGFA |
| GO:0051128 | Regulation of cellular component organization | 64 | 2402 | 9.46E-25 | ERCC1,MAPK1,LGALS1,CSNK2A1,TGFB1,HGF,SERPINE1,IFNG,DUSP1,TNFSF10,BMP4,SIRT2,GDF15,MDM2,XRCC1,NOX4,TP63,RB1,IGF1R,TP53,ERBB2,EGFR,NOTCH1,PAK1,BAX,AXL,BIRC5,ADAM17,ERCC4,MET,EZH2,HSPA5,STK11,CTNNB1,HMGB1,E2F1,ERCC5,CASP8,MTOR,MKI67,PTEN,TLR4,AR,TP73,IGFBP3,KDM4C,ERCC2,XRCC5,SPP1,YWHAZ,NFE2L2,BCL2,CDKN1A,IL6,CD44,TNF,TOP2A,CDKN2A,BRCA1,HIF1A,XRCC3,BIRC2,VEGFA,MUC1 |
| GO:0010646 | Regulation of cell communication | 75 | 3514 | 1.27E-24 | MAPK1,LGALS1,CSNK2A1,TNFRSF10A,TGFB1,HGF,SERPINE1,IFNG,DUSP1,TNFSF10,BMP4,GDF15,MDM2,CDH1,NOX4,PIK3CA,STAT3,TP63,TIMP3,RB1,IGF1R,TP53,ERBB2,EGFR,NOTCH1,PAK1,BAX,AXL,TERT,ADAM17,MET,EZH2,SOX2,HSPA5,STK11,NOS2,CTNNB1,HMGB1,E2F1,FAS,CASP8,STAT1,MTOR,PTGS2,FASLG,S100A4,MCL1,XIAP,PTEN,TLR4,AR,TP73,POSTN,IGFBP3,JAK2,SIRT3,SPP1,YWHAZ,NFE2L2,BCL2,GSTP1,IL6,CD44,TNF,CDKN2A,BRCA1,PDK2,EIF4E,HIF1A,SLC16A1,SOD2,BIRC2,CYP1B1,VEGFA,MUC1 |
| GO:0048513 | Animal organ development | 72 | 3197 | 1.34E-24 | ERCC1,MAPK1,LGALS1,TGFB1,HGF,SERPINE1,CCND1,IFNG,TNFSF10,BMP4,SIRT2,ALDH1A2,MDM2,CDH1,XRCC1,NOX4,EPAS1,PIK3CA,STAT3,TP63,RB1,TP53,ERBB2,EGFR,NOTCH1,PAK1,BAX,AXL,ADAM17,PRKDC,TYMS,MET,CXCR2,EZH2,SOX2,HSPA5,STK11,RARB,BSG,CTNNB1,HMGB1,E2F1,CASP8,STAT1,MTOR,PTGS2,FASLG,S100A4,PTEN,UGT1A1,AR,BMI1,TP73,JAK2,ERCC2,XRCC5,SPP1,YWHAZ,BCL2,GSTP1,RUNX3,CDKN1A,IL6,CD44,TNF,TOP2A,EIF4E,H2AFX,HIF1A,BIRC2,CYP1B1,VEGFA |
| GO:0019219 | Regulation of nucleobase-containing compound metabolic process | 79 | 3982 | 1.59E-24 | ERCC1,MAPK1,APEX1,TGFB1,HGF,SERPINE1,CCND1,IFNG,MLH1,DUSP1,BMP4,SIRT2,MDM2,CDH1,XRCC1,NOX4,EPAS1,PIK3CA,STAT3,TP63,RB1,TP53,ERBB2,EGFR,NOTCH1,BAX,BIRC5,MGMT,OGG1,TERT,ERCC4,PRKDC,TYMS,USP9X,MET,EZH2,SOX2,HSPA5,NOS2,RARB,MTA1,CTNNB1,HMGB1,E2F1,STAT1,MTOR,FASLG,HDGF,PTEN,TLR4,AR,BMI1,TP73,KDM4C,JAK2,TP53BP1,RAD51,HSPD1,ERCC2,XRCC5,SPP1,YWHAZ,NFE2L2,RUNX3,CDKN1A,IL6,TNF,TOP2A,CDKN2A,BRCA1,PDK2,H2AFX,HIF1A,SOD2,ZFP36,BIRC2,CYP1B1,VEGFA,MUC1 |
| GO:0045935 | Positive regulation of nucleobase-containing compound metabolic process | 58 | 1927 | 2.26E-24 | ERCC1,MAPK1,APEX1,TGFB1,HGF,SERPINE1,IFNG,MLH1,BMP4,SIRT2,CDH1,XRCC1,NOX4,EPAS1,STAT3,TP63,RB1,TP53,ERBB2,EGFR,NOTCH1,BAX,MGMT,TERT,PRKDC,USP9X,MET,SOX2,HSPA5,NOS2,RARB,MTA1,CTNNB1,HMGB1,E2F1,STAT1,MTOR,TLR4,AR,TP73,TP53BP1,RAD51,ERCC2,XRCC5,SPP1,NFE2L2,RUNX3,IL6,TNF,TOP2A,CDKN2A,BRCA1,H2AFX,HIF1A,ZFP36,BIRC2,VEGFA,MUC1 |
| GO:0023051 | Regulation of signaling | 75 | 3553 | 2.44E-24 | MAPK1,LGALS1,CSNK2A1,TNFRSF10A,TGFB1,HGF,SERPINE1,IFNG,DUSP1,TNFSF10,BMP4,GDF15,MDM2,CDH1,NOX4,PIK3CA,STAT3,TP63,TIMP3,RB1,IGF1R,TP53,ERBB2,EGFR,NOTCH1,PAK1,BAX,AXL,TERT,ADAM17,MET,EZH2,SOX2,HSPA5,STK11,NOS2,CTNNB1,HMGB1,E2F1,FAS,CASP8,STAT1,MTOR,PTGS2,FASLG,S100A4,MCL1,XIAP,PTEN,TLR4,AR,TP73,POSTN,IGFBP3,JAK2,SIRT3,SPP1,YWHAZ,NFE2L2,BCL2,GSTP1,IL6,CD44,TNF,CDKN2A,BRCA1,PDK2,EIF4E,HIF1A,SLC16A1,SOD2,BIRC2,CYP1B1,VEGFA,MUC1 |
| GO:0009968 | Negative regulation of signal transduction | 49 | 1271 | 2.87E-24 | CSNK2A1,TNFRSF10A,TGFB1,HGF,SERPINE1,DUSP1,TNFSF10,BMP4,GDF15,MDM2,TP63,TIMP3,RB1,IGF1R,TP53,ERBB2,EGFR,NOTCH1,BAX,TERT,ADAM17,MET,EZH2,SOX2,HSPA5,STK11,CTNNB1,FAS,CASP8,STAT1,MTOR,PTGS2,FASLG,MCL1,PTEN,TLR4,AR,IGFBP3,SIRT3,NFE2L2,BCL2,GSTP1,IL6,CD44,TNF,BRCA1,HIF1A,SOD2,MUC1 |
| GO:0009725 | Response to hormone | 42 | 849 | 3.37E-24 | MAPK1,APEX1,TGFB1,CCND1,DUSP1,TNFSF10,BMP4,MDM2,PIK3CA,STAT3,TIMP3,RB1,IGF1R,EGFR,NOTCH1,PAK1,PRKDC,TYMS,NOS2,RARB,BSG,CTNNB1,STAT1,MTOR,PTGS2,ABCC2,PTEN,UGT1A1,AR,JAK2,HSPD1,SPP1,NFE2L2,BCL2,GSTP1,CDKN1A,IL6,TNF,BRCA1,PDK2,EIF4E,ZFP36 |
| GO:0051726 | Regulation of cell cycle | 48 | 1230 | 6.55E-24 | APEX1,CSNK2A1,TGFB1,CCND1,IFNG,DUSP1,BMP4,SIRT2,MDM2,STAT3,TP63,RB1,TP53,EGFR,NOTCH1,PAK1,BAX,BIRC5,TERT,ADAM17,PRKDC,EZH2,SOX2,STK11,RAD51C,CTNNB1,E2F1,MTOR,PTGS2,MKI67,PTEN,BMI1,TP73,TP53BP1,RAD51,ERCC2,BCL2,RUNX3,CDKN1A,TNF,TOP2A,CDKN2A,BRCA1,EIF4E,H2AFX,XRCC3,BIRC2,MUC1 |
| GO:0045597 | Positive regulation of cell differentiation | 44 | 993 | 1.09E-23 | TGFB1,HGF,SERPINE1,IFNG,BMP4,SIRT2,GDF15,MDM2,STAT3,TP63,RB1,NOTCH1,PAK1,AXL,TERT,PRKDC,EZH2,SOX2,HSPA5,STK11,RARB,CTNNB1,HMGB1,E2F1,CASP8,STAT1,MTOR,PTGS2,PTEN,AR,TP73,IGFBP3,KDM4C,JAK2,XRCC5,NFE2L2,BCL2,RUNX3,IL6,TNF,HIF1A,SOD2,ZFP36,VEGFA |
| GO:0009966 | Regulation of signal transduction | 70 | 3107 | 1.14E-23 | MAPK1,LGALS1,CSNK2A1,TNFRSF10A,TGFB1,HGF,SERPINE1,IFNG,DUSP1,TNFSF10,BMP4,GDF15,MDM2,NOX4,PIK3CA,STAT3,TP63,TIMP3,RB1,IGF1R,TP53,ERBB2,EGFR,NOTCH1,PAK1,BAX,AXL,TERT,ADAM17,MET,EZH2,SOX2,HSPA5,STK11,CTNNB1,HMGB1,E2F1,FAS,CASP8,STAT1,MTOR,PTGS2,FASLG,S100A4,MCL1,XIAP,PTEN,TLR4,AR,TP73,POSTN,IGFBP3,JAK2,SIRT3,YWHAZ,NFE2L2,BCL2,GSTP1,IL6,CD44,TNF,CDKN2A,BRCA1,PDK2,HIF1A,SOD2,BIRC2,CYP1B1,VEGFA,MUC1 |
| GO:0032502 | Developmental process | 92 | 5841 | 1.17E-23 | ERCC1,MAPK1,LGALS1,APEX1,TGFB1,HGF,SERPINE1,CCND1,IFNG,MLH1,DUSP1,TNFSF10,BMP4,SIRT2,ALDH1A2,MDM2,CDH1,XRCC1,NOX4,EPAS1,PIK3CA,STAT3,TP63,RB1,IGF1R,TP53,ERBB2,EGFR,NOTCH1,PAK1,BAX,AXL,OGG1,TERT,ADAM17,PRKDC,TYMS,USP9X,MET,CXCR2,EZH2,SOX2,HSPA5,STK11,RARB,BSG,PDCD1,RAD51C,CTNNB1,HMGB1,E2F1,CASP8,STAT1,MTOR,PTGS2,FASLG,S100A4,MCL1,PTEN,UGT1A1,TLR4,AR,BMI1,TP73,POSTN,IGFBP3,KDM4C,JAK2,SIRT3,HSPD1,ERCC2,XRCC5,SPP1,YWHAZ,NFE2L2,BCL2,GSTP1,RUNX3,CDKN1A,IL6,CD44,TNF,TOP2A,CDKN2A,BRCA1,EIF4E,H2AFX,HIF1A,SOD2,BIRC2,CYP1B1,VEGFA |
| GO:0042325 | Regulation of phosphorylation | 53 | 1626 | 2.36E-23 | MAPK1,TNFRSF10A,TGFB1,HGF,CCND1,IFNG,DUSP1,BMP4,SIRT2,GDF15,NOX4,PIK3CA,STAT3,TIMP3,RB1,IGF1R,TP53,ERBB2,EGFR,NOTCH1,PAK1,BAX,AXL,ADAM17,PRKDC,MET,EZH2,SOX2,STK11,CTNNB1,HMGB1,FAS,MTOR,PTGS2,PTEN,TLR4,AR,TP73,IGFBP3,JAK2,RAD51,SIRT3,XRCC5,YWHAZ,BCL2,GSTP1,CDKN1A,IL6,CD44,TNF,CDKN2A,HIF1A,VEGFA |
| GO:0065009 | Regulation of molecular function | 85 | 4913 | 2.46E-23 | MAPK1,APEX1,CSNK2A1,TNFRSF10A,TGFB1,HGF,SERPINE1,CCND1,IFNG,DUSP1,TNFSF10,BMP4,SIRT2,GDF15,MDM2,XRCC1,NOX4,EPAS1,PIK3CA,STAT3,TP63,TIMP3,RB1,IGF1R,TP53,ERBB2,EGFR,NOTCH1,PAK1,BAX,ALDH1A1,AXL,BIRC5,TERT,ADAM17,ERCC4,MET,EZH2,SOX2,STK11,NOS2,RARB,MTA1,CTNNB1,HMGB1,E2F1,FAS,CASP8,STAT1,MTOR,PTGS2,FASLG,HDGF,XIAP,PTEN,UGT1A1,TLR4,AR,BMI1,TP73,IGFBP3,JAK2,TP53BP1,SIRT3,HSPD1,ERCC2,XRCC5,SPP1,NFE2L2,BCL2,GSTP1,RUNX3,CDKN1A,IL6,CD44,TNF,CDKN2A,BRCA1,PDK2,HIF1A,ZFP36,BIRC2,CYP1B1,VEGFA,MUC1 |
| GO:0045595 | Regulation of cell differentiation | 56 | 1874 | 3.34E-23 | MAPK1,LGALS1,TGFB1,HGF,SERPINE1,CCND1,IFNG,BMP4,SIRT2,GDF15,MDM2,STAT3,TP63,RB1,TP53,ERBB2,EGFR,NOTCH1,PAK1,AXL,TERT,PRKDC,EZH2,SOX2,HSPA5,STK11,RARB,CTNNB1,HMGB1,E2F1,CASP8,STAT1,MTOR,PTGS2,PTEN,TLR4,AR,TP73,POSTN,IGFBP3,KDM4C,JAK2,XRCC5,SPP1,NFE2L2,BCL2,RUNX3,IL6,TNF,CDKN2A,EIF4E,HIF1A,SOD2,ZFP36,BIRC2,VEGFA |
| GO:0010468 | Regulation of gene expression | 84 | 4813 | 3.84E-23 | ERCC1,MAPK1,APEX1,TGFB1,HGF,SERPINE1,CCND1,IFNG,MLH1,BMP4,SIRT2,ALDH1A2,MDM2,CDH1,EPAS1,PIK3CA,STAT3,TP63,RB1,TP53,ERBB2,EGFR,NOTCH1,AXL,BIRC5,OGG1,TERT,ADAM17,PRKDC,TYMS,USP9X,MET,EZH2,SOX2,HSPA5,NOS2,RARB,MTA1,BSG,CTNNB1,HMGB1,E2F1,CASP8,STAT1,MTOR,PTGS2,FASLG,HDGF,ABCC2,XIAP,PTEN,TLR4,AR,BMI1,TP73,POSTN,KDM4C,CD274,JAK2,TP53BP1,HSPD1,ERCC2,XRCC5,SPP1,YWHAZ,NFE2L2,BCL2,GSTP1,RUNX3,CDKN1A,IL6,TNF,TOP2A,CDKN2A,BRCA1,EIF4E,H2AFX,HIF1A,SOD2,ZFP36,BIRC2,CYP1B1,VEGFA,MUC1 |
| GO:0019220 | Regulation of phosphate metabolic process | 55 | 1816 | 5.82E-23 | MAPK1,TNFRSF10A,TGFB1,HGF,CCND1,IFNG,DUSP1,BMP4,SIRT2,GDF15,NOX4,PIK3CA,STAT3,TIMP3,RB1,IGF1R,TP53,ERBB2,EGFR,NOTCH1,PAK1,BAX,AXL,ADAM17,PRKDC,MET,EZH2,SOX2,STK11,NOS2,CTNNB1,HMGB1,FAS,MTOR,PTGS2,PTEN,TLR4,AR,TP73,IGFBP3,JAK2,RAD51,SIRT3,XRCC5,YWHAZ,BCL2,GSTP1,CDKN1A,IL6,CD44,TNF,CDKN2A,PDK2,HIF1A,VEGFA |
| GO:0051241 | Negative regulation of multicellular organismal process | 47 | 1231 | 5.82E-23 | LGALS1,TGFB1,HGF,SERPINE1,IFNG,BMP4,SIRT2,GDF15,MDM2,STAT3,TP63,RB1,TP53,ERBB2,EGFR,NOTCH1,PAK1,ALDH1A1,AXL,TERT,ADAM17,SOX2,STK11,RARB,PDCD1,CTNNB1,HMGB1,STAT1,MTOR,PTGS2,FASLG,PTEN,TLR4,TP73,CD274,JAK2,SPP1,NFE2L2,BCL2,GSTP1,RUNX3,IL6,TNF,CDKN2A,EIF4E,HIF1A,ZFP36 |
| GO:0071495 | Cellular response to endogenous stimulus | 46 | 1181 | 9.58E-23 | MAPK1,APEX1,TGFB1,DUSP1,BMP4,SIRT2,GDF15,MDM2,CDH1,NOX4,PIK3CA,STAT3,RB1,IGF1R,TP53,ERBB2,EGFR,NOTCH1,PAK1,PRKDC,USP9X,EZH2,HSPA5,RARB,CTNNB1,E2F1,STAT1,MTOR,PTGS2,ABCC2,PTEN,UGT1A1,TLR4,AR,POSTN,JAK2,RAD51,NFE2L2,GSTP1,ABCC1,CD44,TNF,BRCA1,PDK2,EIF4E,ZFP36 |
| GO:0001932 | Regulation of protein phosphorylation | 50 | 1459 | 1.02E-22 | MAPK1,TNFRSF10A,TGFB1,HGF,CCND1,IFNG,DUSP1,BMP4,SIRT2,GDF15,NOX4,PIK3CA,STAT3,TIMP3,RB1,IGF1R,TP53,ERBB2,EGFR,NOTCH1,PAK1,BAX,ADAM17,PRKDC,EZH2,SOX2,STK11,CTNNB1,HMGB1,FAS,MTOR,PTGS2,PTEN,TLR4,AR,TP73,IGFBP3,JAK2,RAD51,SIRT3,XRCC5,YWHAZ,BCL2,GSTP1,CDKN1A,IL6,CD44,TNF,CDKN2A,VEGFA |
| GO:0007275 | Multicellular organism development | 85 | 5023 | 1.08E-22 | ERCC1,MAPK1,LGALS1,TGFB1,HGF,SERPINE1,CCND1,IFNG,MLH1,DUSP1,TNFSF10,BMP4,SIRT2,ALDH1A2,MDM2,CDH1,XRCC1,NOX4,EPAS1,PIK3CA,STAT3,TP63,RB1,IGF1R,TP53,ERBB2,EGFR,NOTCH1,PAK1,BAX,AXL,TERT,ADAM17,PRKDC,TYMS,USP9X,MET,CXCR2,EZH2,SOX2,HSPA5,STK11,RARB,BSG,PDCD1,CTNNB1,HMGB1,E2F1,CASP8,STAT1,MTOR,PTGS2,FASLG,S100A4,MCL1,PTEN,UGT1A1,TLR4,AR,BMI1,TP73,POSTN,KDM4C,JAK2,HSPD1,ERCC2,XRCC5,SPP1,YWHAZ,NFE2L2,BCL2,GSTP1,RUNX3,CDKN1A,IL6,CD44,TNF,TOP2A,BRCA1,EIF4E,H2AFX,HIF1A,BIRC2,CYP1B1,VEGFA |
| GO:0031401 | Positive regulation of protein modification process | 47 | 1252 | 1.08E-22 | MAPK1,TNFRSF10A,TGFB1,HGF,CCND1,IFNG,BMP4,GDF15,NOX4,PIK3CA,STAT3,IGF1R,TP53,ERBB2,EGFR,NOTCH1,PAK1,ADAM17,EZH2,SOX2,HSPA5,STK11,MTA1,CTNNB1,HMGB1,FAS,MTOR,PTGS2,XIAP,PTEN,TLR4,AR,BMI1,TP73,IGFBP3,JAK2,XRCC5,BCL2,CDKN1A,IL6,CD44,TNF,CDKN2A,BRCA1,BIRC2,VEGFA,MUC1 |
| GO:0045596 | Negative regulation of cell differentiation | 38 | 728 | 1.94E-22 | MAPK1,LGALS1,TGFB1,CCND1,IFNG,BMP4,SIRT2,MDM2,STAT3,TP63,RB1,TP53,ERBB2,EGFR,NOTCH1,PAK1,TERT,EZH2,SOX2,RARB,CTNNB1,HMGB1,E2F1,STAT1,PTEN,TLR4,TP73,POSTN,SPP1,NFE2L2,BCL2,RUNX3,IL6,TNF,CDKN2A,EIF4E,ZFP36,VEGFA |
| GO:0048731 | System development | 80 | 4426 | 2.20E-22 | ERCC1,MAPK1,LGALS1,TGFB1,HGF,SERPINE1,CCND1,IFNG,MLH1,TNFSF10,BMP4,SIRT2,ALDH1A2,MDM2,CDH1,XRCC1,NOX4,EPAS1,PIK3CA,STAT3,TP63,RB1,TP53,ERBB2,EGFR,NOTCH1,PAK1,BAX,AXL,TERT,ADAM17,PRKDC,TYMS,USP9X,MET,CXCR2,EZH2,SOX2,HSPA5,STK11,RARB,BSG,CTNNB1,HMGB1,E2F1,CASP8,STAT1,MTOR,PTGS2,FASLG,S100A4,PTEN,UGT1A1,TLR4,AR,BMI1,TP73,POSTN,KDM4C,JAK2,HSPD1,ERCC2,XRCC5,SPP1,YWHAZ,NFE2L2,BCL2,GSTP1,RUNX3,CDKN1A,IL6,CD44,TNF,TOP2A,EIF4E,H2AFX,HIF1A,BIRC2,CYP1B1,VEGFA |
| GO:2001234 | Negative regulation of apoptotic signaling pathway | 26 | 232 | 2.20E-22 | CSNK2A1,TNFRSF10A,HGF,SERPINE1,TNFSF10,BMP4,MDM2,RB1,BAX,TERT,CTNNB1,FAS,CASP8,PTGS2,FASLG,MCL1,AR,NFE2L2,BCL2,GSTP1,CD44,TNF,BRCA1,HIF1A,SOD2,MUC1 |
| GO:0022008 | Neurogenesis | 52 | 1657 | 3.87E-22 | MAPK1,LGALS1,TGFB1,HGF,IFNG,BMP4,SIRT2,ALDH1A2,MDM2,CDH1,PIK3CA,STAT3,RB1,TP53,ERBB2,EGFR,NOTCH1,PAK1,BAX,AXL,TERT,USP9X,MET,EZH2,SOX2,HSPA5,STK11,RARB,BSG,CTNNB1,HMGB1,E2F1,MTOR,PTEN,TLR4,TP73,POSTN,KDM4C,JAK2,ERCC2,XRCC5,SPP1,YWHAZ,NFE2L2,BCL2,GSTP1,RUNX3,IL6,TNF,EIF4E,HIF1A,VEGFA |
| GO:0048869 | Cellular developmental process | 74 | 3757 | 4.43E-22 | ERCC1,MAPK1,LGALS1,TGFB1,HGF,SERPINE1,CCND1,IFNG,MLH1,BMP4,SIRT2,ALDH1A2,MDM2,CDH1,NOX4,EPAS1,PIK3CA,STAT3,TP63,RB1,TP53,ERBB2,EGFR,NOTCH1,PAK1,BAX,AXL,TERT,ADAM17,PRKDC,TYMS,USP9X,MET,EZH2,SOX2,HSPA5,STK11,RARB,BSG,CTNNB1,HMGB1,E2F1,CASP8,STAT1,MTOR,PTGS2,FASLG,S100A4,MCL1,PTEN,TLR4,AR,TP73,POSTN,IGFBP3,KDM4C,JAK2,ERCC2,XRCC5,SPP1,YWHAZ,NFE2L2,BCL2,GSTP1,RUNX3,CDKN1A,IL6,TNF,TOP2A,CDKN2A,EIF4E,H2AFX,HIF1A,VEGFA |
| GO:0008285 | Negative regulation of cell population proliferation | 37 | 696 | 4.74E-22 | TGFB1,IFNG,DUSP1,BMP4,SIRT2,ALDH1A2,NOX4,STAT3,RB1,TP53,ERBB2,NOTCH1,PAK1,BAX,TERT,SOX2,STK11,RARB,CTNNB1,E2F1,STAT1,PTGS2,PTEN,AR,TP73,IGFBP3,CD274,JAK2,BCL2,GSTP1,RUNX3,CDKN1A,IL6,TNF,CDKN2A,SOD2,CYP1B1 |
| GO:0008284 | Positive regulation of cell population proliferation | 41 | 919 | 5.10E-22 | MAPK1,CSNK2A1,TGFB1,CCND1,IFNG,BMP4,ALDH1A2,MDM2,STAT3,TP63,IGF1R,ERBB2,EGFR,NOTCH1,PAK1,BIRC5,TERT,ADAM17,PRKDC,CXCR2,EZH2,CTNNB1,HMGB1,E2F1,STAT1,MTOR,PTGS2,FASLG,PTEN,TLR4,AR,BMI1,KDM4C,CD274,JAK2,BCL2,CDKN1A,IL6,TNF,HIF1A,VEGFA |
| GO:0010243 | Response to organonitrogen compound | 42 | 987 | 6.91E-22 | MAPK1,APEX1,CCND1,DUSP1,TNFSF10,SIRT2,MDM2,CDH1,NOX4,PIK3CA,STAT3,IGF1R,TP53,EGFR,NOTCH1,PAK1,OGG1,PRKDC,TYMS,EZH2,HSPA5,BSG,CTNNB1,STAT1,MTOR,PTGS2,ABCC2,PTEN,TLR4,TP73,JAK2,RAD51,HSPD1,NFE2L2,GSTP1,ABCC1,CDKN1A,IL6,TNF,BRCA1,PDK2,BIRC2 |
| GO:0048699 | Generation of neurons | 50 | 1551 | 1.30E-21 | MAPK1,LGALS1,TGFB1,HGF,IFNG,BMP4,SIRT2,ALDH1A2,MDM2,CDH1,PIK3CA,STAT3,RB1,TP53,ERBB2,EGFR,NOTCH1,PAK1,BAX,AXL,TERT,USP9X,MET,EZH2,SOX2,HSPA5,STK11,RARB,BSG,CTNNB1,HMGB1,E2F1,MTOR,PTEN,TP73,POSTN,KDM4C,JAK2,ERCC2,XRCC5,SPP1,YWHAZ,NFE2L2,BCL2,RUNX3,IL6,TNF,EIF4E,HIF1A,VEGFA |
| GO:1901698 | Response to nitrogen compound | 43 | 1070 | 1.45E-21 | MAPK1,APEX1,CCND1,DUSP1,TNFSF10,SIRT2,MDM2,CDH1,NOX4,PIK3CA,STAT3,IGF1R,TP53,EGFR,NOTCH1,PAK1,OGG1,GSTM1,PRKDC,TYMS,EZH2,HSPA5,BSG,CTNNB1,STAT1,MTOR,PTGS2,ABCC2,PTEN,TLR4,TP73,JAK2,RAD51,HSPD1,NFE2L2,GSTP1,ABCC1,CDKN1A,IL6,TNF,BRCA1,PDK2,BIRC2 |
| GO:0045786 | Negative regulation of cell cycle | 34 | 571 | 1.49E-21 | TGFB1,CCND1,IFNG,DUSP1,BMP4,MDM2,RB1,TP53,EGFR,NOTCH1,BAX,BIRC5,PRKDC,EZH2,SOX2,STK11,CTNNB1,E2F1,MTOR,PTGS2,PTEN,BMI1,TP73,TP53BP1,RAD51,BCL2,RUNX3,CDKN1A,TNF,TOP2A,CDKN2A,BRCA1,H2AFX,MUC1 |
| GO:0071345 | Cellular response to cytokine stimulus | 42 | 1013 | 1.74E-21 | MAPK1,TGFB1,HGF,CCND1,IFNG,DUSP1,PIK3CA,STAT3,TP53,AXL,BIRC5,ADAM17,CXCR2,SOX2,HSPA5,NOS2,FAS,STAT1,PTGS2,FASLG,HDGF,MCL1,ABCC2,TLR4,POSTN,JAK2,HSPD1,XRCC5,YWHAZ,NFE2L2,BCL2,CDKN1A,IL6,CD44,TNF,BRCA1,HIF1A,SOD2,ZFP36,BIRC2,VEGFA,MUC1 |
| GO:0051052 | Regulation of dna metabolic process | 29 | 360 | 1.77E-21 | ERCC1,MAPK1,TGFB1,HGF,MLH1,DUSP1,XRCC1,NOX4,PIK3CA,TP53,EGFR,BAX,MGMT,OGG1,ERCC4,PRKDC,USP9X,CTNNB1,HMGB1,TP53BP1,RAD51,ERCC2,XRCC5,CDKN1A,IL6,CDKN2A,BRCA1,H2AFX,VEGFA |
| GO:0009605 | Response to external stimulus | 59 | 2310 | 1.83E-21 | ERCC1,MAPK1,TNFRSF10A,TGFB1,HGF,SERPINE1,CCND1,IFNG,MLH1,BMP4,SIRT2,ALDH1A2,GDF15,MDM2,PIK3CA,TP53,ERBB2,EGFR,NOTCH1,AXL,OGG1,ADAM17,PRKDC,TYMS,MET,CXCR2,HSPA5,NOS2,MTA1,BSG,HMGB1,FAS,CASP8,STAT1,MTOR,PTGS2,FASLG,ABCC2,PTEN,UGT1A1,TLR4,POSTN,CD274,JAK2,HSPD1,XRCC5,SPP1,NFE2L2,BCL2,GSTP1,ABCC1,CDKN1A,IL6,CD44,TNF,PDK2,SLC16A1,ZFP36,VEGFA |
| GO:0009889 | Regulation of biosynthetic process | 77 | 4210 | 2.00E-21 | ERCC1,MAPK1,APEX1,TGFB1,HGF,SERPINE1,CCND1,IFNG,DUSP1,BMP4,SIRT2,MDM2,CDH1,NOX4,EPAS1,STAT3,TP63,RB1,TP53,ERBB2,EGFR,NOTCH1,BIRC5,OGG1,TERT,ERCC4,PRKDC,TYMS,USP9X,MET,EZH2,SOX2,HSPA5,NOS2,RARB,MTA1,CTNNB1,HMGB1,E2F1,STAT1,MTOR,PTGS2,FASLG,HDGF,PTEN,TLR4,AR,BMI1,TP73,KDM4C,JAK2,TP53BP1,SIRT3,HSPD1,ERCC2,XRCC5,SPP1,NFE2L2,BCL2,GSTP1,RUNX3,CDKN1A,IL6,TNF,TOP2A,CDKN2A,BRCA1,PDK2,EIF4E,H2AFX,HIF1A,SOD2,ZFP36,BIRC2,CYP1B1,VEGFA,MUC1 |
| GO:0044260 | Cellular macromolecule metabolic process | 83 | 4976 | 2.12E-21 | ERCC1,MAPK1,LGALS1,APEX1,CSNK2A1,TGFB1,HGF,CCND1,MLH1,DUSP1,BMP4,SIRT2,MDM2,XRCC1,EPAS1,PIK3CA,STAT3,IGF1R,TP53,ERBB2,EGFR,NOTCH1,PAK1,BAX,AXL,BIRC5,MGMT,OGG1,TERT,ADAM17,ERCC4,PRKDC,TYMS,USP9X,MET,CXCR2,EZH2,HSPA5,STK11,NOS2,RARB,MTA1,RAD51C,CTNNB1,HMGB1,E2F1,ERCC5,CASP8,MTOR,XIAP,PTEN,TLR4,AR,BMI1,TP73,IGFBP3,KDM4C,JAK2,TP53BP1,RAD51,SIRT3,HSPD1,ERCC2,XRCC5,SPP1,YWHAZ,NFE2L2,BCL2,RUNX3,CDKN1A,IL6,TNF,TOP2A,CDKN2A,BRCA1,PDK2,EIF4E,H2AFX,HIF1A,XRCC3,ZFP36,BIRC2,MUC1 |
| GO:0050789 | Regulation of biological process | 117 | 11475 | 2.32E-21 | ERCC1,MAPK1,LGALS1,APEX1,CSNK2A1,TNFRSF10A,TGFB1,HGF,SERPINE1,CCND1,IFNG,MLH1,DUSP1,TNFSF10,BMP4,SIRT2,ALDH1A2,GDF15,MDM2,CDH1,XRCC1,NOX4,EPAS1,PIK3CA,STAT3,TP63,TIMP3,RB1,IGF1R,TP53,ERBB2,EGFR,NOTCH1,PAK1,BAX,ALDH1A1,AXL,BIRC5,MGMT,OGG1,TERT,ADAM17,ERCC4,PRKDC,TYMS,USP9X,MET,CXCR2,EZH2,SOX2,HSPA5,STK11,NOS2,RARB,MTA1,BSG,PDCD1,RAD51C,CTNNB1,HMGB1,E2F1,ERCC5,FAS,CASP8,STAT1,MTOR,PTGS2,FASLG,HDGF,MKI67,S100A4,MCL1,ABCC2,XIAP,PTEN,UGT1A1,TLR4,AR,BMI1,TP73,POSTN,IGFBP3,KDM4C,CD274,JAK2,TP53BP1,RAD51,SIRT3,HSPD1,ERCC2,XRCC5,SPP1,YWHAZ,NFE2L2,BCL2,GSTP1,ABCC1,RUNX3,CDKN1A,IL6,CD44,TNF,TOP2A,CDKN2A,BRCA1,PDK2,EIF4E,H2AFX,HIF1A,SLC16A1,SOD2,XRCC3,ZFP36,BIRC2,CYP1B1,VEGFA,MUC1 |
| GO:0010628 | Positive regulation of gene expression | 59 | 2337 | 3.17E-21 | ERCC1,MAPK1,TGFB1,HGF,SERPINE1,IFNG,BMP4,SIRT2,ALDH1A2,MDM2,CDH1,EPAS1,STAT3,TP63,RB1,TP53,ERBB2,EGFR,NOTCH1,TERT,ADAM17,PRKDC,MET,SOX2,HSPA5,RARB,BSG,CTNNB1,HMGB1,E2F1,CASP8,STAT1,MTOR,PTGS2,PTEN,TLR4,AR,TP73,POSTN,KDM4C,CD274,JAK2,TP53BP1,HSPD1,ERCC2,XRCC5,SPP1,NFE2L2,RUNX3,IL6,TNF,TOP2A,CDKN2A,BRCA1,HIF1A,ZFP36,CYP1B1,VEGFA,MUC1 |
| GO:0031326 | Regulation of cellular biosynthetic process | 76 | 4125 | 3.34E-21 | ERCC1,MAPK1,APEX1,TGFB1,HGF,SERPINE1,CCND1,IFNG,DUSP1,BMP4,SIRT2,MDM2,CDH1,NOX4,EPAS1,STAT3,TP63,RB1,TP53,ERBB2,EGFR,NOTCH1,BIRC5,OGG1,TERT,ERCC4,PRKDC,TYMS,USP9X,MET,EZH2,SOX2,HSPA5,NOS2,RARB,MTA1,CTNNB1,HMGB1,E2F1,STAT1,MTOR,PTGS2,FASLG,HDGF,PTEN,TLR4,AR,BMI1,TP73,KDM4C,JAK2,TP53BP1,SIRT3,HSPD1,ERCC2,XRCC5,SPP1,NFE2L2,BCL2,RUNX3,CDKN1A,IL6,TNF,TOP2A,CDKN2A,BRCA1,PDK2,EIF4E,H2AFX,HIF1A,SOD2,ZFP36,BIRC2,CYP1B1,VEGFA,MUC1 |
| GO:0044093 | Positive regulation of molecular function | 53 | 1842 | 5.14E-21 | MAPK1,TNFRSF10A,TGFB1,HGF,CCND1,IFNG,TNFSF10,BMP4,SIRT2,GDF15,XRCC1,NOX4,PIK3CA,STAT3,RB1,IGF1R,ERBB2,EGFR,PAK1,BAX,ALDH1A1,AXL,TERT,ADAM17,MET,EZH2,STK11,NOS2,CTNNB1,HMGB1,FAS,CASP8,MTOR,FASLG,PTEN,TLR4,AR,BMI1,TP73,IGFBP3,JAK2,TP53BP1,SIRT3,HSPD1,ERCC2,XRCC5,BCL2,CDKN1A,IL6,TNF,CDKN2A,HIF1A,VEGFA |
| GO:0010556 | Regulation of macromolecule biosynthetic process | 74 | 3976 | 1.29E-20 | ERCC1,MAPK1,APEX1,TGFB1,HGF,SERPINE1,CCND1,IFNG,DUSP1,BMP4,SIRT2,MDM2,CDH1,NOX4,EPAS1,STAT3,TP63,RB1,TP53,ERBB2,EGFR,NOTCH1,BIRC5,OGG1,TERT,ERCC4,PRKDC,TYMS,USP9X,MET,EZH2,SOX2,HSPA5,RARB,MTA1,CTNNB1,HMGB1,E2F1,STAT1,MTOR,FASLG,HDGF,PTEN,TLR4,AR,BMI1,TP73,KDM4C,JAK2,TP53BP1,SIRT3,HSPD1,ERCC2,XRCC5,SPP1,NFE2L2,BCL2,GSTP1,RUNX3,CDKN1A,IL6,TNF,TOP2A,CDKN2A,BRCA1,EIF4E,H2AFX,HIF1A,SOD2,ZFP36,BIRC2,CYP1B1,VEGFA,MUC1 |
| GO:0006979 | Response to oxidative stress | 29 | 393 | 1.62E-20 | ERCC1,MAPK1,APEX1,DUSP1,SIRT2,MDM2,XRCC1,NOX4,EPAS1,TP53,EGFR,AXL,OGG1,EZH2,STAT1,PTGS2,ABCC2,JAK2,GPX3,HSPD1,ERCC2,NFE2L2,BCL2,GSTP1,IL6,PDK2,HIF1A,SOD2,CYP1B1 |
| GO:0042327 | Positive regulation of phosphorylation | 42 | 1093 | 2.58E-20 | MAPK1,TNFRSF10A,TGFB1,HGF,CCND1,IFNG,BMP4,GDF15,NOX4,PIK3CA,STAT3,IGF1R,TP53,ERBB2,EGFR,NOTCH1,PAK1,AXL,ADAM17,MET,EZH2,SOX2,STK11,CTNNB1,HMGB1,FAS,MTOR,PTGS2,PTEN,TLR4,AR,TP73,IGFBP3,JAK2,XRCC5,BCL2,CDKN1A,IL6,CD44,TNF,HIF1A,VEGFA |
| GO:0045937 | Positive regulation of phosphate metabolic process | 43 | 1164 | 3.01E-20 | MAPK1,TNFRSF10A,TGFB1,HGF,CCND1,IFNG,BMP4,GDF15,NOX4,PIK3CA,STAT3,IGF1R,TP53,ERBB2,EGFR,NOTCH1,PAK1,AXL,ADAM17,MET,EZH2,SOX2,STK11,NOS2,CTNNB1,HMGB1,FAS,MTOR,PTGS2,PTEN,TLR4,AR,TP73,IGFBP3,JAK2,XRCC5,BCL2,CDKN1A,IL6,CD44,TNF,HIF1A,VEGFA |
| GO:0042592 | Homeostatic process | 50 | 1676 | 3.13E-20 | ERCC1,MAPK1,LGALS1,APEX1,BMP4,XRCC1,NOX4,EPAS1,PIK3CA,STAT3,TP63,RB1,IGF1R,EGFR,NOTCH1,BAX,AXL,TERT,ERCC4,PRKDC,MET,CXCR2,EZH2,STK11,NOS2,RAD51C,CTNNB1,HMGB1,FAS,STAT1,PTGS2,FASLG,MCL1,ABCC2,XIAP,TLR4,JAK2,RAD51,ERCC2,XRCC5,NFE2L2,BCL2,GSTP1,IL6,PDK2,HIF1A,SLC16A1,SOD2,XRCC3,VEGFA |
| GO:0071407 | Cellular response to organic cyclic compound | 32 | 537 | 3.25E-20 | MAPK1,LGALS1,APEX1,TGFB1,BMP4,SIRT2,MDM2,CDH1,NOX4,STAT3,RB1,EGFR,EZH2,HSPA5,RARB,CTNNB1,CASP8,STAT1,PTGS2,ABCC2,UGT1A1,AR,JAK2,RAD51,SPP1,GSTP1,TNF,BRCA1,EIF4E,SLC16A1,ZFP36,CYP1B1 |
| GO:0030154 | Cell differentiation | 71 | 3702 | 3.77E-20 | ERCC1,MAPK1,LGALS1,TGFB1,HGF,CCND1,IFNG,MLH1,BMP4,SIRT2,ALDH1A2,MDM2,CDH1,NOX4,EPAS1,PIK3CA,STAT3,TP63,RB1,TP53,ERBB2,EGFR,NOTCH1,PAK1,BAX,AXL,TERT,ADAM17,PRKDC,TYMS,USP9X,MET,EZH2,SOX2,HSPA5,STK11,RARB,BSG,CTNNB1,HMGB1,E2F1,CASP8,STAT1,MTOR,PTGS2,FASLG,S100A4,MCL1,PTEN,TLR4,AR,TP73,POSTN,IGFBP3,KDM4C,JAK2,ERCC2,XRCC5,SPP1,YWHAZ,NFE2L2,BCL2,GSTP1,RUNX3,CDKN1A,IL6,TNF,TOP2A,EIF4E,HIF1A,VEGFA |
| GO:1902533 | Positive regulation of intracellular signal transduction | 41 | 1041 | 3.77E-20 | MAPK1,LGALS1,TNFRSF10A,TGFB1,HGF,TNFSF10,BMP4,GDF15,NOX4,PIK3CA,IGF1R,TP53,ERBB2,EGFR,NOTCH1,PAK1,BAX,AXL,MET,EZH2,SOX2,CTNNB1,HMGB1,CASP8,MTOR,FASLG,S100A4,MCL1,PTEN,TLR4,AR,TP73,IGFBP3,JAK2,BCL2,IL6,CD44,TNF,CDKN2A,BIRC2,VEGFA |
| GO:0044237 | Cellular metabolic process | 98 | 7513 | 4.37E-20 | ERCC1,MAPK1,LGALS1,APEX1,CSNK2A1,TGFB1,HGF,CCND1,MLH1,DUSP1,BMP4,SIRT2,ALDH1A2,MDM2,XRCC1,NOX4,EPAS1,PIK3CA,STAT3,IGF1R,TP53,ERBB2,EGFR,NOTCH1,PAK1,BAX,ALDH1A1,AXL,BIRC5,MGMT,OGG1,TERT,ADAM17,ERCC4,GSTM1,PRKDC,TYMS,USP9X,MET,CXCR2,EZH2,HSPA5,STK11,NOS2,RARB,MTA1,BSG,RAD51C,CTNNB1,HMGB1,E2F1,ERCC5,CASP8,MTOR,PTGS2,ABCC2,XIAP,PTEN,UGT1A1,TLR4,AR,BMI1,TP73,IGFBP3,KDM4C,JAK2,TP53BP1,RAD51,SIRT3,GPX3,HSPD1,ERCC2,XRCC5,SPP1,YWHAZ,NFE2L2,BCL2,GSTP1,ABCC1,RUNX3,CDKN1A,IL6,CD44,TNF,TOP2A,CDKN2A,BRCA1,PDK2,EIF4E,H2AFX,HIF1A,SLC16A1,SOD2,XRCC3,ZFP36,BIRC2,CYP1B1,MUC1 |
| GO:0030334 | Regulation of cell migration | 38 | 865 | 4.90E-20 | MAPK1,APEX1,TGFB1,HGF,SERPINE1,IFNG,DUSP1,BMP4,MDM2,CDH1,NOX4,STAT3,IGF1R,EGFR,NOTCH1,PAK1,TERT,ADAM17,MET,CXCR2,HSPA5,HMGB1,MTOR,PTGS2,PTEN,POSTN,IGFBP3,CD274,JAK2,NFE2L2,BCL2,GSTP1,IL6,TNF,HIF1A,SOD2,CYP1B1,VEGFA |
| GO:0050794 | Regulation of cellular process | 114 | 10932 | 5.61E-20 | ERCC1,MAPK1,LGALS1,APEX1,CSNK2A1,TNFRSF10A,TGFB1,HGF,SERPINE1,CCND1,IFNG,MLH1,DUSP1,TNFSF10,BMP4,SIRT2,ALDH1A2,GDF15,MDM2,CDH1,XRCC1,NOX4,EPAS1,PIK3CA,STAT3,TP63,TIMP3,RB1,IGF1R,TP53,ERBB2,EGFR,NOTCH1,PAK1,BAX,AXL,BIRC5,MGMT,OGG1,TERT,ADAM17,ERCC4,PRKDC,TYMS,USP9X,MET,CXCR2,EZH2,SOX2,HSPA5,STK11,NOS2,RARB,MTA1,BSG,PDCD1,RAD51C,CTNNB1,HMGB1,E2F1,ERCC5,FAS,CASP8,STAT1,MTOR,PTGS2,FASLG,HDGF,MKI67,S100A4,MCL1,XIAP,PTEN,UGT1A1,TLR4,AR,BMI1,TP73,POSTN,IGFBP3,KDM4C,CD274,JAK2,TP53BP1,RAD51,SIRT3,HSPD1,ERCC2,XRCC5,SPP1,YWHAZ,NFE2L2,BCL2,GSTP1,RUNX3,CDKN1A,IL6,CD44,TNF,TOP2A,CDKN2A,BRCA1,PDK2,EIF4E,H2AFX,HIF1A,SLC16A1,SOD2,XRCC3,ZFP36,BIRC2,CYP1B1,VEGFA,MUC1 |
| GO:2000145 | Regulation of cell motility | 39 | 929 | 5.66E-20 | MAPK1,APEX1,TGFB1,HGF,SERPINE1,IFNG,DUSP1,BMP4,MDM2,CDH1,NOX4,STAT3,IGF1R,ERBB2,EGFR,NOTCH1,PAK1,TERT,ADAM17,MET,CXCR2,HSPA5,HMGB1,MTOR,PTGS2,PTEN,POSTN,IGFBP3,CD274,JAK2,NFE2L2,BCL2,GSTP1,IL6,TNF,HIF1A,SOD2,CYP1B1,VEGFA |
| GO:0050678 | Regulation of epithelial cell proliferation | 27 | 339 | 7.97E-20 | TGFB1,CCND1,BMP4,ALDH1A2,STAT3,TP63,RB1,ERBB2,EGFR,NOTCH1,BAX,ADAM17,PRKDC,SOX2,STK11,CTNNB1,HMGB1,STAT1,MTOR,PTEN,TLR4,AR,RUNX3,TNF,HIF1A,ZFP36,VEGFA |
| GO:0043085 | Positive regulation of catalytic activity | 47 | 1489 | 9.21E-20 | MAPK1,TNFRSF10A,TGFB1,HGF,CCND1,IFNG,TNFSF10,BMP4,GDF15,XRCC1,NOX4,PIK3CA,STAT3,IGF1R,ERBB2,EGFR,PAK1,BAX,ALDH1A1,AXL,TERT,ADAM17,MET,EZH2,STK11,NOS2,CTNNB1,HMGB1,FAS,CASP8,MTOR,FASLG,PTEN,TLR4,BMI1,TP73,IGFBP3,JAK2,SIRT3,HSPD1,XRCC5,BCL2,CDKN1A,TNF,CDKN2A,HIF1A,VEGFA |
| GO:0032501 | Multicellular organismal process | 94 | 6933 | 1.21E-19 | ERCC1,MAPK1,LGALS1,TGFB1,HGF,SERPINE1,CCND1,IFNG,MLH1,DUSP1,TNFSF10,BMP4,SIRT2,ALDH1A2,GDF15,MDM2,CDH1,XRCC1,NOX4,EPAS1,PIK3CA,STAT3,TP63,TIMP3,RB1,IGF1R,TP53,ERBB2,EGFR,NOTCH1,PAK1,BAX,AXL,BIRC5,TERT,ADAM17,PRKDC,TYMS,USP9X,MET,CXCR2,EZH2,SOX2,HSPA5,STK11,NOS2,RARB,MTA1,BSG,PDCD1,RAD51C,CTNNB1,HMGB1,E2F1,CASP8,STAT1,MTOR,PTGS2,FASLG,S100A4,MCL1,ABCC2,PTEN,UGT1A1,TLR4,AR,BMI1,TP73,POSTN,IGFBP3,KDM4C,JAK2,HSPD1,ERCC2,XRCC5,SPP1,YWHAZ,NFE2L2,BCL2,GSTP1,RUNX3,CDKN1A,IL6,CD44,TNF,TOP2A,BRCA1,EIF4E,H2AFX,HIF1A,SOD2,BIRC2,CYP1B1,VEGFA |
| GO:0030335 | Positive regulation of cell migration | 31 | 522 | 1.66E-19 | MAPK1,TGFB1,HGF,SERPINE1,IFNG,BMP4,MDM2,NOX4,STAT3,IGF1R,EGFR,NOTCH1,PAK1,TERT,ADAM17,MET,CXCR2,HSPA5,HMGB1,MTOR,PTGS2,POSTN,CD274,JAK2,NFE2L2,BCL2,IL6,TNF,HIF1A,SOD2,VEGFA |
| GO:0002682 | Regulation of immune system process | 47 | 1514 | 1.76E-19 | MAPK1,LGALS1,TGFB1,SERPINE1,IFNG,MLH1,DUSP1,BMP4,PIK3CA,STAT3,RB1,ERBB2,NOTCH1,PAK1,BAX,AXL,ADAM17,PRKDC,CXCR2,STK11,NOS2,PDCD1,CTNNB1,HMGB1,CASP8,STAT1,MTOR,XIAP,TLR4,TP73,CD274,JAK2,TP53BP1,HSPD1,XRCC5,NFE2L2,BCL2,RUNX3,CDKN1A,IL6,TNF,CDKN2A,HIF1A,ZFP36,BIRC2,VEGFA,MUC1 |
| GO:0031328 | Positive regulation of cellular biosynthetic process | 53 | 2005 | 2.01E-19 | ERCC1,MAPK1,APEX1,TGFB1,HGF,SERPINE1,IFNG,BMP4,SIRT2,CDH1,NOX4,EPAS1,STAT3,TP63,RB1,TP53,ERBB2,EGFR,NOTCH1,TERT,PRKDC,MET,SOX2,HSPA5,NOS2,RARB,MTA1,CTNNB1,HMGB1,E2F1,STAT1,MTOR,PTGS2,TLR4,AR,TP73,JAK2,TP53BP1,SIRT3,ERCC2,XRCC5,SPP1,NFE2L2,RUNX3,IL6,TNF,TOP2A,CDKN2A,BRCA1,HIF1A,BIRC2,VEGFA,MUC1 |
| GO:0007399 | Nervous system development | 57 | 2371 | 2.40E-19 | MAPK1,LGALS1,TGFB1,HGF,IFNG,BMP4,SIRT2,ALDH1A2,MDM2,CDH1,XRCC1,PIK3CA,STAT3,TP63,RB1,TP53,ERBB2,EGFR,NOTCH1,PAK1,BAX,AXL,TERT,PRKDC,USP9X,MET,CXCR2,EZH2,SOX2,HSPA5,STK11,RARB,BSG,CTNNB1,HMGB1,E2F1,MTOR,PTEN,TLR4,TP73,POSTN,KDM4C,JAK2,ERCC2,XRCC5,SPP1,YWHAZ,NFE2L2,BCL2,GSTP1,RUNX3,IL6,TNF,EIF4E,H2AFX,HIF1A,VEGFA |
| GO:0006974 | Cellular response to dna damage stimulus | 36 | 793 | 2.54E-19 | ERCC1,MAPK1,APEX1,CCND1,MLH1,MDM2,XRCC1,TP63,TP53,PAK1,BAX,MGMT,OGG1,ERCC4,PRKDC,STK11,MTA1,RAD51C,HMGB1,E2F1,ERCC5,MCL1,XIAP,TP73,TP53BP1,RAD51,ERCC2,XRCC5,BCL2,CDKN1A,TNF,TOP2A,BRCA1,H2AFX,XRCC3,MUC1 |
| GO:2000377 | Regulation of reactive oxygen species metabolic process | 22 | 188 | 3.22E-19 | TGFB1,IFNG,SIRT2,NOX4,STAT3,TP53,EGFR,MTOR,PTGS2,TLR4,JAK2,SIRT3,HSPD1,NFE2L2,BCL2,GSTP1,CDKN1A,TNF,BRCA1,HIF1A,BIRC2,CYP1B1 |
| GO:0048660 | Regulation of smooth muscle cell proliferation | 20 | 136 | 3.82E-19 | IFNG,BMP4,MDM2,EGFR,PAK1,TERT,PRKDC,CTNNB1,STAT1,MTOR,PTGS2,PTEN,IGFBP3,JAK2,XRCC5,GSTP1,CDKN1A,IL6,TNF,SOD2 |
| GO:0048545 | Response to steroid hormone | 26 | 328 | 5.11E-19 | TGFB1,CCND1,DUSP1,BMP4,MDM2,RB1,EGFR,NOTCH1,TYMS,RARB,CTNNB1,PTGS2,ABCC2,UGT1A1,AR,JAK2,HSPD1,SPP1,BCL2,GSTP1,CDKN1A,IL6,TNF,BRCA1,EIF4E,ZFP36 |
| GO:1903506 | Regulation of nucleic acid-templated transcription | 67 | 3447 | 7.04E-19 | ERCC1,MAPK1,APEX1,TGFB1,HGF,SERPINE1,CCND1,IFNG,BMP4,SIRT2,MDM2,CDH1,EPAS1,STAT3,TP63,RB1,TP53,ERBB2,EGFR,NOTCH1,BIRC5,OGG1,TERT,PRKDC,TYMS,USP9X,MET,EZH2,SOX2,HSPA5,RARB,MTA1,CTNNB1,HMGB1,E2F1,STAT1,MTOR,FASLG,HDGF,PTEN,TLR4,AR,BMI1,TP73,KDM4C,JAK2,TP53BP1,HSPD1,ERCC2,XRCC5,SPP1,NFE2L2,RUNX3,CDKN1A,IL6,TNF,TOP2A,CDKN2A,BRCA1,H2AFX,HIF1A,SOD2,ZFP36,BIRC2,CYP1B1,VEGFA,MUC1 |
| GO:0051130 | Positive regulation of cellular component organization | 42 | 1209 | 8.32E-19 | ERCC1,MAPK1,TGFB1,HGF,SERPINE1,IFNG,TNFSF10,SIRT2,GDF15,NOX4,TP63,RB1,IGF1R,TP53,PAK1,BAX,AXL,BIRC5,ERCC4,MET,EZH2,HSPA5,STK11,CTNNB1,E2F1,ERCC5,CASP8,MTOR,TLR4,AR,TP73,ERCC2,XRCC5,YWHAZ,NFE2L2,BCL2,IL6,TNF,BRCA1,HIF1A,VEGFA,MUC1 |
| GO:0051054 | Positive regulation of dna metabolic process | 22 | 200 | 1.03E-18 | ERCC1,MAPK1,TGFB1,HGF,MLH1,XRCC1,NOX4,EGFR,BAX,MGMT,PRKDC,USP9X,CTNNB1,HMGB1,TP53BP1,RAD51,ERCC2,XRCC5,IL6,BRCA1,H2AFX,VEGFA |
| GO:0001934 | Positive regulation of protein phosphorylation | 39 | 1019 | 1.13E-18 | MAPK1,TNFRSF10A,TGFB1,HGF,CCND1,IFNG,BMP4,GDF15,NOX4,PIK3CA,STAT3,IGF1R,TP53,ERBB2,EGFR,NOTCH1,PAK1,ADAM17,EZH2,SOX2,STK11,CTNNB1,HMGB1,FAS,MTOR,PTGS2,PTEN,TLR4,AR,TP73,IGFBP3,JAK2,XRCC5,BCL2,CDKN1A,IL6,CD44,TNF,VEGFA |
| GO:0051248 | Negative regulation of protein metabolic process | 40 | 1096 | 1.61E-18 | CSNK2A1,TGFB1,HGF,SERPINE1,IFNG,DUSP1,BMP4,SIRT2,MDM2,XRCC1,STAT3,TIMP3,RB1,IGF1R,TP53,EGFR,BAX,BIRC5,PRKDC,TYMS,NOS2,CTNNB1,MTOR,PTGS2,XIAP,PTEN,TLR4,IGFBP3,KDM4C,SIRT3,GSTP1,CDKN1A,CD44,TNF,CDKN2A,BRCA1,EIF4E,ZFP36,BIRC2,VEGFA |
| GO:0045934 | Negative regulation of nucleobase-containing compound metabolic process | 46 | 1528 | 1.63E-18 | ERCC1,APEX1,TGFB1,CCND1,IFNG,MLH1,DUSP1,BMP4,SIRT2,MDM2,XRCC1,STAT3,TP63,RB1,TP53,NOTCH1,BIRC5,OGG1,TERT,ERCC4,USP9X,EZH2,SOX2,RARB,MTA1,CTNNB1,HMGB1,E2F1,STAT1,MTOR,FASLG,HDGF,AR,BMI1,TP53BP1,XRCC5,RUNX3,CDKN1A,IL6,TNF,CDKN2A,BRCA1,H2AFX,ZFP36,VEGFA,MUC1 |
| GO:0007346 | Regulation of mitotic cell cycle | 33 | 676 | 1.78E-18 | APEX1,TGFB1,CCND1,DUSP1,BMP4,SIRT2,MDM2,RB1,TP53,EGFR,PAK1,BAX,BIRC5,TERT,ADAM17,PRKDC,EZH2,RAD51C,CTNNB1,E2F1,MKI67,PTEN,TP73,ERCC2,BCL2,CDKN1A,TNF,TOP2A,CDKN2A,BRCA1,EIF4E,XRCC3,MUC1 |
| GO:2000112 | Regulation of cellular macromolecule biosynthetic process | 70 | 3878 | 2.63E-18 | ERCC1,MAPK1,TGFB1,HGF,SERPINE1,CCND1,IFNG,DUSP1,BMP4,SIRT2,MDM2,CDH1,NOX4,EPAS1,STAT3,TP63,RB1,TP53,ERBB2,EGFR,NOTCH1,BIRC5,OGG1,TERT,ERCC4,PRKDC,TYMS,USP9X,MET,EZH2,SOX2,HSPA5,RARB,MTA1,CTNNB1,HMGB1,E2F1,STAT1,MTOR,FASLG,HDGF,PTEN,TLR4,AR,BMI1,TP73,KDM4C,JAK2,TP53BP1,HSPD1,ERCC2,XRCC5,SPP1,NFE2L2,BCL2,RUNX3,CDKN1A,IL6,TNF,TOP2A,CDKN2A,BRCA1,EIF4E,H2AFX,HIF1A,SOD2,ZFP36,CYP1B1,VEGFA,MUC1 |
| GO:0007165 | Signal transduction | 78 | 4876 | 2.94E-18 | MAPK1,CSNK2A1,TNFRSF10A,TGFB1,HGF,CCND1,IFNG,MLH1,DUSP1,TNFSF10,BMP4,SIRT2,ALDH1A2,GDF15,MDM2,EPAS1,PIK3CA,STAT3,TP63,RB1,IGF1R,TP53,ERBB2,EGFR,NOTCH1,PAK1,BAX,AXL,BIRC5,ADAM17,PRKDC,USP9X,MET,CXCR2,SOX2,HSPA5,STK11,NOS2,RARB,MTA1,BSG,CTNNB1,HMGB1,E2F1,FAS,CASP8,STAT1,MTOR,PTGS2,FASLG,HDGF,MCL1,XIAP,PTEN,TLR4,AR,TP73,CD274,JAK2,HSPD1,SPP1,YWHAZ,NFE2L2,BCL2,CDKN1A,IL6,CD44,TNF,CDKN2A,BRCA1,PDK2,HIF1A,SOD2,ZFP36,BIRC2,CYP1B1,VEGFA,MUC1 |
| GO:0007166 | Cell surface receptor signaling pathway | 55 | 2325 | 3.33E-18 | MAPK1,CSNK2A1,TNFRSF10A,TGFB1,HGF,CCND1,IFNG,TNFSF10,BMP4,SIRT2,GDF15,PIK3CA,STAT3,TP63,IGF1R,TP53,ERBB2,EGFR,NOTCH1,PAK1,BAX,AXL,BIRC5,ADAM17,USP9X,MET,CXCR2,SOX2,STK11,NOS2,BSG,CTNNB1,FAS,CASP8,STAT1,PTGS2,FASLG,MCL1,XIAP,PTEN,TLR4,CD274,JAK2,YWHAZ,BCL2,CDKN1A,IL6,CD44,TNF,PDK2,HIF1A,SOD2,BIRC2,VEGFA,MUC1 |
| GO:0043281 | Regulation of cysteine-type endopeptidase activity involved in apoptotic process | 22 | 216 | 4.43E-18 | CSNK2A1,TNFRSF10A,HGF,TNFSF10,MDM2,TP63,BAX,BIRC5,SOX2,HMGB1,FAS,CASP8,PTGS2,FASLG,XIAP,JAK2,HSPD1,CD44,TNF,CDKN2A,BIRC2,VEGFA |
| GO:0097190 | Apoptotic signaling pathway | 24 | 286 | 5.03E-18 | TNFRSF10A,TGFB1,IFNG,MLH1,TP63,TP53,BAX,PRKDC,STK11,E2F1,FAS,CASP8,FASLG,MCL1,TLR4,TP73,JAK2,BCL2,CDKN1A,TNF,BRCA1,PDK2,SOD2,CYP1B1 |
| GO:0010557 | Positive regulation of macromolecule biosynthetic process | 50 | 1906 | 5.55E-18 | ERCC1,MAPK1,APEX1,TGFB1,HGF,SERPINE1,IFNG,BMP4,SIRT2,CDH1,NOX4,EPAS1,STAT3,TP63,RB1,TP53,ERBB2,EGFR,NOTCH1,TERT,PRKDC,MET,SOX2,HSPA5,RARB,MTA1,CTNNB1,HMGB1,E2F1,STAT1,MTOR,TLR4,AR,TP73,JAK2,TP53BP1,ERCC2,XRCC5,SPP1,NFE2L2,RUNX3,IL6,TNF,TOP2A,CDKN2A,BRCA1,HIF1A,BIRC2,VEGFA,MUC1 |
| GO:0032879 | Regulation of localization | 59 | 2740 | 5.74E-18 | MAPK1,APEX1,TGFB1,HGF,SERPINE1,IFNG,DUSP1,BMP4,MDM2,CDH1,NOX4,STAT3,TP63,RB1,IGF1R,TP53,ERBB2,EGFR,NOTCH1,PAK1,BAX,AXL,TERT,ADAM17,MET,CXCR2,HSPA5,STK11,NOS2,CTNNB1,HMGB1,E2F1,CASP8,MTOR,PTGS2,FASLG,PTEN,TLR4,AR,TP73,POSTN,IGFBP3,CD274,JAK2,SIRT3,SPP1,YWHAZ,NFE2L2,BCL2,GSTP1,IL6,TNF,CDKN2A,HIF1A,SLC16A1,SOD2,ZFP36,CYP1B1,VEGFA |
| GO:0002520 | Immune system development | 32 | 652 | 6.14E-18 | ERCC1,MAPK1,LGALS1,TGFB1,IFNG,MLH1,BMP4,EPAS1,STAT3,RB1,TP53,BAX,AXL,ADAM17,PRKDC,STK11,CTNNB1,HMGB1,CASP8,MTOR,BMI1,JAK2,HSPD1,ERCC2,XRCC5,BCL2,RUNX3,IL6,TNF,TOP2A,HIF1A,VEGFA |
| GO:0051252 | Regulation of rna metabolic process | 68 | 3722 | 7.62E-18 | ERCC1,MAPK1,APEX1,TGFB1,HGF,SERPINE1,CCND1,IFNG,BMP4,SIRT2,MDM2,CDH1,EPAS1,STAT3,TP63,RB1,TP53,ERBB2,EGFR,NOTCH1,BIRC5,OGG1,TERT,PRKDC,TYMS,USP9X,MET,EZH2,SOX2,HSPA5,RARB,MTA1,CTNNB1,HMGB1,E2F1,STAT1,MTOR,FASLG,HDGF,PTEN,TLR4,AR,BMI1,TP73,KDM4C,JAK2,TP53BP1,HSPD1,ERCC2,XRCC5,SPP1,YWHAZ,NFE2L2,RUNX3,CDKN1A,IL6,TNF,TOP2A,CDKN2A,BRCA1,H2AFX,HIF1A,SOD2,ZFP36,BIRC2,CYP1B1,VEGFA,MUC1 |
| GO:0006355 | Regulation of transcription, dna-templated | 65 | 3388 | 7.86E-18 | ERCC1,MAPK1,TGFB1,HGF,SERPINE1,CCND1,IFNG,BMP4,SIRT2,MDM2,CDH1,EPAS1,STAT3,TP63,RB1,TP53,ERBB2,EGFR,NOTCH1,BIRC5,OGG1,TERT,PRKDC,TYMS,USP9X,MET,EZH2,SOX2,HSPA5,RARB,MTA1,CTNNB1,HMGB1,E2F1,STAT1,MTOR,FASLG,HDGF,PTEN,TLR4,AR,BMI1,TP73,KDM4C,JAK2,TP53BP1,HSPD1,ERCC2,XRCC5,SPP1,NFE2L2,RUNX3,CDKN1A,IL6,TNF,TOP2A,CDKN2A,BRCA1,H2AFX,HIF1A,SOD2,ZFP36,CYP1B1,VEGFA,MUC1 |
| GO:0010629 | Negative regulation of gene expression | 51 | 2014 | 8.60E-18 | TGFB1,HGF,SERPINE1,CCND1,IFNG,MLH1,BMP4,SIRT2,MDM2,STAT3,TP63,RB1,TP53,NOTCH1,AXL,BIRC5,TERT,TYMS,USP9X,EZH2,SOX2,NOS2,RARB,MTA1,CTNNB1,HMGB1,E2F1,STAT1,FASLG,HDGF,ABCC2,XIAP,TLR4,AR,BMI1,CD274,XRCC5,GSTP1,RUNX3,CDKN1A,IL6,TNF,CDKN2A,BRCA1,EIF4E,H2AFX,HIF1A,ZFP36,BIRC2,VEGFA,MUC1 |
| GO:0006807 | Nitrogen compound metabolic process | 91 | 6852 | 8.73E-18 | ERCC1,MAPK1,LGALS1,APEX1,CSNK2A1,TGFB1,HGF,CCND1,MLH1,DUSP1,BMP4,SIRT2,MDM2,XRCC1,NOX4,EPAS1,PIK3CA,STAT3,IGF1R,TP53,ERBB2,EGFR,NOTCH1,PAK1,BAX,AXL,BIRC5,MGMT,OGG1,TERT,ADAM17,ERCC4,GSTM1,PRKDC,TYMS,USP9X,MET,EZH2,HSPA5,STK11,NOS2,RARB,MTA1,RAD51C,CTNNB1,HMGB1,E2F1,ERCC5,CASP8,MTOR,PTGS2,ABCC2,XIAP,PTEN,UGT1A1,TLR4,AR,BMI1,TP73,IGFBP3,KDM4C,JAK2,TP53BP1,RAD51,SIRT3,HSPD1,ERCC2,XRCC5,SPP1,YWHAZ,NFE2L2,BCL2,GSTP1,ABCC1,RUNX3,CDKN1A,IL6,CD44,TNF,TOP2A,CDKN2A,BRCA1,PDK2,EIF4E,H2AFX,HIF1A,XRCC3,ZFP36,BIRC2,CYP1B1,MUC1 |
| GO:0051338 | Regulation of transferase activity | 38 | 1036 | 1.47E-17 | MAPK1,TNFRSF10A,TGFB1,HGF,CCND1,IFNG,DUSP1,BMP4,GDF15,NOX4,PIK3CA,RB1,IGF1R,TP53,ERBB2,EGFR,PAK1,AXL,ADAM17,ERCC4,MET,EZH2,STK11,CTNNB1,MTOR,PTEN,UGT1A1,TLR4,BMI1,TP73,JAK2,XRCC5,GSTP1,CDKN1A,TNF,CDKN2A,ZFP36,VEGFA |
| GO:0043170 | Macromolecule metabolic process | 86 | 6137 | 1.59E-17 | ERCC1,MAPK1,LGALS1,APEX1,CSNK2A1,TGFB1,HGF,CCND1,MLH1,DUSP1,BMP4,SIRT2,MDM2,XRCC1,NOX4,EPAS1,PIK3CA,STAT3,IGF1R,TP53,ERBB2,EGFR,NOTCH1,PAK1,BAX,AXL,BIRC5,MGMT,OGG1,TERT,ADAM17,ERCC4,PRKDC,TYMS,USP9X,MET,CXCR2,EZH2,HSPA5,STK11,NOS2,RARB,MTA1,RAD51C,CTNNB1,HMGB1,E2F1,ERCC5,CASP8,MTOR,PTGS2,XIAP,PTEN,TLR4,AR,BMI1,TP73,IGFBP3,KDM4C,JAK2,TP53BP1,RAD51,SIRT3,HSPD1,ERCC2,XRCC5,SPP1,YWHAZ,NFE2L2,BCL2,RUNX3,CDKN1A,IL6,CD44,TNF,TOP2A,CDKN2A,BRCA1,PDK2,EIF4E,H2AFX,HIF1A,XRCC3,ZFP36,BIRC2,MUC1 |
| GO:0032269 | Negative regulation of cellular protein metabolic process | 38 | 1043 | 1.81E-17 | CSNK2A1,TGFB1,HGF,SERPINE1,IFNG,DUSP1,BMP4,SIRT2,MDM2,XRCC1,STAT3,TIMP3,RB1,IGF1R,TP53,BAX,BIRC5,PRKDC,TYMS,CTNNB1,MTOR,PTGS2,XIAP,PTEN,TLR4,IGFBP3,KDM4C,SIRT3,GSTP1,CDKN1A,CD44,TNF,CDKN2A,BRCA1,EIF4E,ZFP36,BIRC2,VEGFA |
| GO:0071704 | Organic substance metabolic process | 96 | 7755 | 1.81E-17 | ERCC1,MAPK1,LGALS1,APEX1,CSNK2A1,TGFB1,HGF,CCND1,MLH1,DUSP1,BMP4,SIRT2,ALDH1A2,MDM2,XRCC1,NOX4,EPAS1,PIK3CA,STAT3,IGF1R,TP53,ERBB2,EGFR,NOTCH1,PAK1,BAX,ALDH1A1,AXL,BIRC5,MGMT,OGG1,TERT,ADAM17,ERCC4,GSTM1,PRKDC,TYMS,USP9X,MET,CXCR2,EZH2,HSPA5,STK11,NOS2,RARB,MTA1,BSG,RAD51C,CTNNB1,HMGB1,E2F1,ERCC5,CASP8,MTOR,PTGS2,ABCC2,XIAP,PTEN,UGT1A1,TLR4,AR,BMI1,TP73,IGFBP3,KDM4C,JAK2,TP53BP1,RAD51,SIRT3,HSPD1,ERCC2,XRCC5,SPP1,YWHAZ,NFE2L2,BCL2,GSTP1,ABCC1,RUNX3,CDKN1A,IL6,CD44,TNF,TOP2A,CDKN2A,BRCA1,PDK2,EIF4E,H2AFX,HIF1A,SLC16A1,XRCC3,ZFP36,BIRC2,CYP1B1,MUC1 |
| GO:0007154 | Cell communication | 80 | 5320 | 2.47E-17 | MAPK1,CSNK2A1,TNFRSF10A,TGFB1,HGF,CCND1,IFNG,MLH1,DUSP1,TNFSF10,BMP4,SIRT2,ALDH1A2,GDF15,MDM2,EPAS1,PIK3CA,STAT3,TP63,RB1,IGF1R,TP53,ERBB2,EGFR,NOTCH1,PAK1,BAX,AXL,BIRC5,ADAM17,PRKDC,USP9X,MET,CXCR2,SOX2,HSPA5,STK11,NOS2,RARB,MTA1,BSG,CTNNB1,HMGB1,E2F1,FAS,CASP8,STAT1,MTOR,PTGS2,FASLG,HDGF,MCL1,XIAP,PTEN,TLR4,AR,TP73,POSTN,CD274,JAK2,HSPD1,SPP1,YWHAZ,NFE2L2,BCL2,GSTP1,CDKN1A,IL6,CD44,TNF,CDKN2A,BRCA1,PDK2,HIF1A,SOD2,ZFP36,BIRC2,CYP1B1,VEGFA,MUC1 |
| GO:0006357 | Regulation of transcription by rna polymerase ii | 52 | 2172 | 3.37E-17 | ERCC1,TGFB1,HGF,SERPINE1,CCND1,IFNG,BMP4,SIRT2,MDM2,EPAS1,STAT3,TP63,RB1,TP53,ERBB2,EGFR,NOTCH1,TERT,PRKDC,USP9X,MET,EZH2,SOX2,HSPA5,RARB,MTA1,CTNNB1,HMGB1,E2F1,STAT1,FASLG,HDGF,TLR4,AR,BMI1,TP73,KDM4C,TP53BP1,HSPD1,NFE2L2,RUNX3,CDKN1A,IL6,TNF,TOP2A,CDKN2A,BRCA1,HIF1A,SOD2,ZFP36,VEGFA,MUC1 |
| GO:1903508 | Positive regulation of nucleic acid-templated transcription | 46 | 1670 | 4.55E-17 | ERCC1,MAPK1,APEX1,TGFB1,HGF,SERPINE1,BMP4,SIRT2,CDH1,EPAS1,STAT3,TP63,RB1,TP53,ERBB2,EGFR,NOTCH1,TERT,PRKDC,MET,SOX2,HSPA5,RARB,MTA1,CTNNB1,HMGB1,E2F1,STAT1,MTOR,TLR4,AR,TP73,TP53BP1,ERCC2,SPP1,NFE2L2,RUNX3,IL6,TNF,TOP2A,CDKN2A,BRCA1,HIF1A,BIRC2,VEGFA,MUC1 |
| GO:0051254 | Positive regulation of rna metabolic process | 47 | 1759 | 5.25E-17 | ERCC1,MAPK1,APEX1,TGFB1,HGF,SERPINE1,BMP4,SIRT2,CDH1,EPAS1,STAT3,TP63,RB1,TP53,ERBB2,EGFR,NOTCH1,TERT,PRKDC,MET,SOX2,HSPA5,RARB,MTA1,CTNNB1,HMGB1,E2F1,STAT1,MTOR,TLR4,AR,TP73,TP53BP1,ERCC2,SPP1,NFE2L2,RUNX3,IL6,TNF,TOP2A,CDKN2A,BRCA1,HIF1A,ZFP36,BIRC2,VEGFA,MUC1 |
| GO:0062197 | Cellular response to chemical stress | 23 | 293 | 1.15E-16 | MAPK1,APEX1,SIRT2,MDM2,NOX4,EPAS1,TP53,EGFR,AXL,EZH2,FAS,PTGS2,JAK2,GPX3,XRCC5,NFE2L2,BCL2,GSTP1,IL6,PDK2,HIF1A,SOD2,CYP1B1 |
| GO:0048534 | Hematopoietic or lymphoid organ development | 30 | 619 | 1.36E-16 | ERCC1,MAPK1,LGALS1,TGFB1,IFNG,BMP4,EPAS1,STAT3,RB1,TP53,BAX,AXL,ADAM17,PRKDC,STK11,CTNNB1,HMGB1,CASP8,MTOR,BMI1,JAK2,ERCC2,XRCC5,BCL2,RUNX3,IL6,TNF,TOP2A,HIF1A,VEGFA |
| GO:0070482 | Response to oxygen levels | 25 | 379 | 1.54E-16 | SIRT2,MDM2,XRCC1,NOX4,EPAS1,TP53,NOTCH1,PAK1,TERT,ADAM17,NOS2,E2F1,FAS,MTOR,PTGS2,PTEN,POSTN,HSPD1,ERCC2,NFE2L2,BCL2,CDKN1A,HIF1A,BIRC2,VEGFA |
| GO:0030097 | Hemopoiesis | 29 | 570 | 1.59E-16 | ERCC1,LGALS1,TGFB1,IFNG,BMP4,EPAS1,STAT3,RB1,TP53,BAX,AXL,ADAM17,PRKDC,STK11,CTNNB1,HMGB1,CASP8,MTOR,BMI1,JAK2,ERCC2,XRCC5,BCL2,RUNX3,IL6,TNF,TOP2A,HIF1A,VEGFA |
| GO:0009314 | Response to radiation | 26 | 431 | 2.32E-16 | ERCC1,CCND1,DUSP1,MDM2,NOX4,TP53,EGFR,BAX,OGG1,ERCC4,PRKDC,HSPA5,STK11,MTA1,ERCC5,MTOR,PTGS2,TP53BP1,RAD51,ERCC2,XRCC5,BCL2,CDKN1A,BRCA1,H2AFX,HIF1A |
| GO:0009991 | Response to extracellular stimulus | 27 | 483 | 2.94E-16 | ERCC1,MAPK1,CCND1,SIRT2,ALDH1A2,GDF15,MDM2,TP53,EGFR,AXL,OGG1,TYMS,HSPA5,FAS,STAT1,MTOR,PTGS2,UGT1A1,POSTN,SPP1,NFE2L2,BCL2,GSTP1,CDKN1A,PDK2,SLC16A1,ZFP36 |
| GO:0071417 | Cellular response to organonitrogen compound | 29 | 590 | 3.76E-16 | MAPK1,APEX1,SIRT2,MDM2,CDH1,NOX4,PIK3CA,STAT3,IGF1R,TP53,EGFR,PAK1,PRKDC,EZH2,HSPA5,CTNNB1,STAT1,MTOR,PTGS2,PTEN,TLR4,JAK2,RAD51,NFE2L2,GSTP1,ABCC1,TNF,BRCA1,PDK2 |
| GO:0010564 | Regulation of cell cycle process | 32 | 760 | 3.97E-16 | APEX1,CSNK2A1,CCND1,DUSP1,BMP4,SIRT2,MDM2,TP63,RB1,TP53,EGFR,BAX,BIRC5,TERT,ADAM17,PRKDC,EZH2,RAD51C,CTNNB1,E2F1,MKI67,PTEN,BMI1,TP73,RAD51,ERCC2,BCL2,CDKN1A,CDKN2A,BRCA1,XRCC3,MUC1 |
| GO:0032101 | Regulation of response to external stimulus | 36 | 1013 | 4.02E-16 | MAPK1,TGFB1,HGF,SERPINE1,IFNG,DUSP1,SIRT2,STAT3,RB1,EGFR,NOTCH1,PAK1,ADAM17,PRKDC,MET,CXCR2,NOS2,HMGB1,STAT1,MTOR,PTGS2,XIAP,PTEN,TLR4,JAK2,HSPD1,XRCC5,SPP1,NFE2L2,GSTP1,ABCC1,IL6,TNF,BIRC2,VEGFA,MUC1 |
| GO:0007568 | Aging | 22 | 274 | 4.17E-16 | ERCC1,MAPK1,APEX1,SERPINE1,NOX4,STAT3,TP63,TP53,OGG1,TERT,TYMS,MTOR,PTGS2,PTEN,SIRT3,ERCC2,NFE2L2,BCL2,CDKN1A,CDKN2A,H2AFX,SOD2 |
| GO:0031667 | Response to nutrient levels | 26 | 449 | 5.76E-16 | ERCC1,MAPK1,CCND1,SIRT2,ALDH1A2,GDF15,MDM2,TP53,EGFR,OGG1,TYMS,HSPA5,FAS,STAT1,MTOR,PTGS2,UGT1A1,POSTN,SPP1,NFE2L2,BCL2,GSTP1,CDKN1A,PDK2,SLC16A1,ZFP36 |
| GO:0048468 | Cell development | 44 | 1629 | 6.77E-16 | ERCC1,MAPK1,IFNG,MLH1,BMP4,SIRT2,ALDH1A2,CDH1,EPAS1,PIK3CA,TP63,RB1,ERBB2,EGFR,NOTCH1,PAK1,BAX,AXL,PRKDC,TYMS,USP9X,MET,STK11,RARB,BSG,CTNNB1,HMGB1,MTOR,FASLG,PTEN,TLR4,AR,POSTN,JAK2,ERCC2,YWHAZ,BCL2,GSTP1,RUNX3,CDKN1A,IL6,TNF,HIF1A,VEGFA |
| GO:0048732 | Gland development | 25 | 410 | 8.39E-16 | MAPK1,TGFB1,HGF,CCND1,BMP4,ALDH1A2,CDH1,PIK3CA,TP63,EGFR,NOTCH1,PRKDC,TYMS,MET,EZH2,SOX2,CTNNB1,PTEN,UGT1A1,AR,JAK2,BCL2,TNF,HIF1A,VEGFA |
| GO:0009653 | Anatomical structure morphogenesis | 50 | 2165 | 9.12E-16 | ERCC1,MAPK1,TGFB1,HGF,SERPINE1,DUSP1,BMP4,ALDH1A2,MDM2,CDH1,XRCC1,NOX4,EPAS1,PIK3CA,STAT3,TP63,RB1,TP53,ERBB2,EGFR,NOTCH1,PAK1,BAX,AXL,ADAM17,PRKDC,USP9X,MET,CXCR2,SOX2,HSPA5,STK11,RARB,BSG,CTNNB1,MTOR,PTGS2,FASLG,PTEN,AR,POSTN,KDM4C,ERCC2,YWHAZ,BCL2,CD44,TNF,HIF1A,CYP1B1,VEGFA |
| GO:0043408 | Regulation of mapk cascade | 31 | 725 | 9.24E-16 | MAPK1,TGFB1,HGF,DUSP1,BMP4,GDF15,NOX4,TIMP3,IGF1R,ERBB2,EGFR,NOTCH1,PAK1,EZH2,SOX2,CTNNB1,HMGB1,FAS,PTEN,TLR4,AR,TP73,IGFBP3,JAK2,SIRT3,YWHAZ,GSTP1,IL6,CD44,TNF,VEGFA |
| GO:0044238 | Primary metabolic process | 91 | 7332 | 1.14E-15 | ERCC1,MAPK1,LGALS1,APEX1,CSNK2A1,TGFB1,HGF,CCND1,MLH1,DUSP1,BMP4,SIRT2,ALDH1A2,MDM2,XRCC1,NOX4,EPAS1,PIK3CA,STAT3,IGF1R,TP53,ERBB2,EGFR,NOTCH1,PAK1,BAX,ALDH1A1,AXL,BIRC5,MGMT,OGG1,TERT,ADAM17,ERCC4,GSTM1,PRKDC,TYMS,USP9X,MET,EZH2,HSPA5,STK11,NOS2,RARB,MTA1,RAD51C,CTNNB1,HMGB1,E2F1,ERCC5,CASP8,MTOR,PTGS2,XIAP,PTEN,UGT1A1,TLR4,AR,BMI1,TP73,IGFBP3,KDM4C,JAK2,TP53BP1,RAD51,SIRT3,HSPD1,ERCC2,XRCC5,SPP1,YWHAZ,NFE2L2,BCL2,GSTP1,RUNX3,CDKN1A,IL6,TNF,TOP2A,CDKN2A,BRCA1,PDK2,EIF4E,H2AFX,HIF1A,SLC16A1,XRCC3,ZFP36,BIRC2,CYP1B1,MUC1 |
| GO:0052548 | Regulation of endopeptidase activity | 25 | 418 | 1.26E-15 | CSNK2A1,TNFRSF10A,HGF,SERPINE1,TNFSF10,MDM2,STAT3,TP63,TIMP3,BAX,BIRC5,SOX2,HMGB1,FAS,CASP8,PTGS2,FASLG,XIAP,JAK2,HSPD1,CD44,TNF,CDKN2A,BIRC2,VEGFA |
| GO:0045893 | Positive regulation of transcription, dna-templated | 43 | 1587 | 1.60E-15 | ERCC1,MAPK1,TGFB1,HGF,SERPINE1,BMP4,SIRT2,CDH1,EPAS1,STAT3,TP63,RB1,TP53,ERBB2,EGFR,NOTCH1,TERT,PRKDC,MET,SOX2,HSPA5,RARB,CTNNB1,HMGB1,E2F1,STAT1,MTOR,TLR4,AR,TP73,TP53BP1,ERCC2,SPP1,NFE2L2,RUNX3,IL6,TNF,TOP2A,CDKN2A,BRCA1,HIF1A,VEGFA,MUC1 |
| GO:0030162 | Regulation of proteolysis | 31 | 747 | 2.03E-15 | CSNK2A1,TNFRSF10A,HGF,SERPINE1,IFNG,TNFSF10,SIRT2,MDM2,STAT3,TP63,TIMP3,RB1,TP53,BAX,BIRC5,SOX2,HMGB1,FAS,CASP8,PTGS2,FASLG,XIAP,PTEN,JAK2,HSPD1,NFE2L2,CD44,TNF,CDKN2A,BIRC2,VEGFA |
| GO:0071396 | Cellular response to lipid | 27 | 528 | 2.30E-15 | MAPK1,TGFB1,SERPINE1,BMP4,ALDH1A2,RB1,EGFR,AXL,NOS2,RARB,CTNNB1,HMGB1,E2F1,ABCC2,UGT1A1,TLR4,AR,CD274,JAK2,XRCC5,SPP1,GSTP1,IL6,TNF,BRCA1,EIF4E,ZFP36 |
| GO:0001666 | Response to hypoxia | 23 | 342 | 2.50E-15 | SIRT2,MDM2,XRCC1,NOX4,EPAS1,TP53,NOTCH1,PAK1,TERT,ADAM17,NOS2,E2F1,MTOR,PTGS2,PTEN,POSTN,HSPD1,ERCC2,NFE2L2,BCL2,HIF1A,BIRC2,VEGFA |
| GO:0002684 | Positive regulation of immune system process | 34 | 949 | 3.11E-15 | MAPK1,LGALS1,TGFB1,SERPINE1,IFNG,MLH1,PIK3CA,STAT3,RB1,PAK1,BAX,AXL,ADAM17,PRKDC,CXCR2,STK11,NOS2,PDCD1,HMGB1,CASP8,STAT1,TLR4,CD274,TP53BP1,HSPD1,XRCC5,BCL2,RUNX3,CDKN1A,IL6,TNF,HIF1A,VEGFA,MUC1 |
| GO:0009890 | Negative regulation of biosynthetic process | 43 | 1624 | 3.52E-15 | APEX1,TGFB1,CCND1,IFNG,DUSP1,BMP4,SIRT2,MDM2,STAT3,TP63,RB1,TP53,NOTCH1,BIRC5,ERCC4,TYMS,USP9X,EZH2,SOX2,RARB,MTA1,CTNNB1,HMGB1,E2F1,STAT1,FASLG,HDGF,AR,BMI1,HSPD1,XRCC5,GSTP1,RUNX3,CDKN1A,IL6,TNF,CDKN2A,BRCA1,EIF4E,H2AFX,ZFP36,VEGFA,MUC1 |
| GO:0010035 | Response to inorganic substance | 27 | 538 | 3.52E-15 | ERCC1,MAPK1,APEX1,CCND1,DUSP1,MDM2,CDH1,EGFR,AXL,OGG1,TERT,EZH2,HSPA5,BSG,CASP8,STAT1,PTGS2,ABCC2,PTEN,RAD51,HSPD1,NFE2L2,BCL2,IL6,HIF1A,SOD2,CYP1B1 |
| GO:0051347 | Positive regulation of transferase activity | 30 | 705 | 3.65E-15 | MAPK1,TNFRSF10A,TGFB1,HGF,CCND1,IFNG,BMP4,GDF15,NOX4,PIK3CA,IGF1R,ERBB2,EGFR,PAK1,AXL,ADAM17,MET,EZH2,STK11,CTNNB1,MTOR,PTEN,TLR4,BMI1,TP73,JAK2,XRCC5,CDKN1A,TNF,VEGFA |
| GO:0060284 | Regulation of cell development | 34 | 956 | 3.76E-15 | LGALS1,TGFB1,HGF,IFNG,BMP4,SIRT2,MDM2,STAT3,TP53,NOTCH1,PAK1,TERT,PRKDC,EZH2,SOX2,HSPA5,STK11,RARB,CTNNB1,E2F1,MTOR,PTEN,TP73,POSTN,KDM4C,XRCC5,SPP1,NFE2L2,BCL2,IL6,TNF,EIF4E,HIF1A,VEGFA |
| GO:0045930 | Negative regulation of mitotic cell cycle | 22 | 308 | 3.84E-15 | TGFB1,CCND1,DUSP1,BMP4,MDM2,RB1,TP53,EGFR,BAX,BIRC5,PRKDC,EZH2,CTNNB1,E2F1,PTEN,BCL2,CDKN1A,TNF,TOP2A,CDKN2A,BRCA1,MUC1 |
| GO:0050767 | Regulation of neurogenesis | 32 | 828 | 3.85E-15 | LGALS1,TGFB1,HGF,IFNG,BMP4,SIRT2,MDM2,STAT3,TP53,NOTCH1,PAK1,TERT,EZH2,SOX2,HSPA5,STK11,RARB,CTNNB1,E2F1,MTOR,PTEN,TP73,KDM4C,XRCC5,SPP1,NFE2L2,BCL2,IL6,TNF,EIF4E,HIF1A,VEGFA |
| GO:1901987 | Regulation of cell cycle phase transition | 25 | 452 | 6.67E-15 | APEX1,CCND1,DUSP1,SIRT2,MDM2,TP63,RB1,TP53,EGFR,BAX,BIRC5,TERT,ADAM17,PRKDC,EZH2,RAD51C,E2F1,PTEN,ERCC2,BCL2,CDKN1A,CDKN2A,BRCA1,XRCC3,MUC1 |
| GO:0002376 | Immune system process | 52 | 2481 | 7.87E-15 | ERCC1,MAPK1,LGALS1,TNFRSF10A,TGFB1,IFNG,MLH1,TNFSF10,BMP4,SIRT2,EPAS1,PIK3CA,STAT3,RB1,IGF1R,TP53,NOTCH1,PAK1,BAX,AXL,ADAM17,PRKDC,CXCR2,STK11,NOS2,BSG,PDCD1,CTNNB1,HMGB1,FAS,CASP8,STAT1,MTOR,FASLG,TLR4,BMI1,CD274,JAK2,HSPD1,ERCC2,XRCC5,BCL2,GSTP1,RUNX3,IL6,CD44,TNF,TOP2A,HIF1A,SLC16A1,VEGFA,MUC1 |
| GO:0010212 | Response to ionizing radiation | 17 | 145 | 8.64E-15 | ERCC1,CCND1,MDM2,NOX4,TP53,BAX,PRKDC,HSPA5,STK11,MTA1,TP53BP1,RAD51,XRCC5,BCL2,CDKN1A,BRCA1,H2AFX |
| GO:1902806 | Regulation of cell cycle g1/s phase transition | 18 | 175 | 8.64E-15 | APEX1,CCND1,MDM2,TP63,RB1,TP53,EGFR,BAX,TERT,ADAM17,PRKDC,EZH2,E2F1,PTEN,BCL2,CDKN1A,CDKN2A,MUC1 |
| GO:0034599 | Cellular response to oxidative stress | 20 | 244 | 9.24E-15 | MAPK1,APEX1,SIRT2,MDM2,NOX4,EPAS1,TP53,EGFR,AXL,EZH2,JAK2,GPX3,NFE2L2,BCL2,GSTP1,IL6,PDK2,HIF1A,SOD2,CYP1B1 |
| GO:0019221 | Cytokine-mediated signaling pathway | 29 | 678 | 1.11E-14 | TGFB1,HGF,CCND1,IFNG,PIK3CA,STAT3,TP53,BIRC5,ADAM17,CXCR2,SOX2,NOS2,FAS,STAT1,PTGS2,FASLG,MCL1,JAK2,YWHAZ,BCL2,CDKN1A,IL6,CD44,TNF,HIF1A,SOD2,BIRC2,VEGFA,MUC1 |
| GO:1901990 | Regulation of mitotic cell cycle phase transition | 24 | 416 | 1.17E-14 | APEX1,CCND1,DUSP1,SIRT2,MDM2,RB1,TP53,EGFR,BAX,BIRC5,TERT,ADAM17,PRKDC,EZH2,RAD51C,E2F1,PTEN,ERCC2,BCL2,CDKN1A,CDKN2A,BRCA1,XRCC3,MUC1 |
| GO:0002237 | Response to molecule of bacterial origin | 22 | 330 | 1.42E-14 | MAPK1,TGFB1,SERPINE1,SIRT2,NOTCH1,AXL,ADAM17,NOS2,HMGB1,CASP8,PTGS2,FASLG,ABCC2,UGT1A1,TLR4,CD274,JAK2,HSPD1,GSTP1,IL6,TNF,ZFP36 |
| GO:0010558 | Negative regulation of macromolecule biosynthetic process | 41 | 1534 | 1.66E-14 | APEX1,TGFB1,CCND1,IFNG,DUSP1,BMP4,SIRT2,MDM2,STAT3,TP63,RB1,TP53,NOTCH1,BIRC5,ERCC4,TYMS,USP9X,EZH2,SOX2,RARB,MTA1,CTNNB1,HMGB1,E2F1,STAT1,FASLG,HDGF,AR,BMI1,XRCC5,GSTP1,RUNX3,CDKN1A,TNF,CDKN2A,BRCA1,EIF4E,H2AFX,ZFP36,VEGFA,MUC1 |
| GO:0051960 | Regulation of nervous system development | 33 | 942 | 1.73E-14 | LGALS1,TGFB1,HGF,IFNG,BMP4,SIRT2,MDM2,STAT3,TP53,NOTCH1,PAK1,TERT,EZH2,SOX2,HSPA5,STK11,RARB,CTNNB1,E2F1,MTOR,PTEN,TP73,KDM4C,XRCC5,SPP1,YWHAZ,NFE2L2,BCL2,IL6,TNF,EIF4E,HIF1A,VEGFA |
| GO:0060249 | Anatomical structure homeostasis | 23 | 380 | 1.96E-14 | ERCC1,APEX1,XRCC1,NOX4,EPAS1,RB1,NOTCH1,BAX,TERT,ERCC4,PRKDC,STK11,RAD51C,CTNNB1,PTGS2,TLR4,RAD51,XRCC5,BCL2,IL6,HIF1A,XRCC3,VEGFA |
| GO:0090068 | Positive regulation of cell cycle process | 21 | 294 | 1.96E-14 | APEX1,CCND1,SIRT2,MDM2,TP63,RB1,TP53,EGFR,BAX,BIRC5,TERT,ADAM17,EZH2,RAD51C,E2F1,TP73,CDKN1A,CDKN2A,BRCA1,XRCC3,MUC1 |
| GO:0007049 | Cell cycle | 38 | 1313 | 2.35E-14 | ERCC1,MAPK1,CSNK2A1,TGFB1,HGF,CCND1,IFNG,MLH1,DUSP1,SIRT2,MDM2,RB1,TP53,NOTCH1,BAX,BIRC5,ERCC4,PRKDC,TYMS,USP9X,EZH2,SOX2,STK11,RAD51C,CTNNB1,E2F1,MTOR,MKI67,TP73,RAD51,CDKN1A,TOP2A,CDKN2A,BRCA1,EIF4E,H2AFX,SLC16A1,MUC1 |
| GO:2000045 | Regulation of g1/s transition of mitotic cell cycle | 17 | 156 | 2.52E-14 | APEX1,CCND1,MDM2,RB1,TP53,EGFR,BAX,TERT,ADAM17,PRKDC,EZH2,E2F1,PTEN,BCL2,CDKN1A,CDKN2A,MUC1 |
| GO:1903829 | Positive regulation of cellular protein localization | 22 | 342 | 2.77E-14 | MAPK1,TGFB1,IFNG,MDM2,CDH1,TP63,TP53,ERBB2,EGFR,PAK1,TERT,STK11,E2F1,CASP8,PTGS2,TP73,JAK2,YWHAZ,BCL2,TNF,CDKN2A,VEGFA |
| GO:2001020 | Regulation of response to dna damage stimulus | 19 | 225 | 3.22E-14 | ERCC1,MDM2,XRCC1,TP53,EGFR,MGMT,OGG1,ERCC4,PRKDC,HMGB1,MCL1,TP53BP1,RAD51,BCL2,CD44,CDKN2A,BRCA1,H2AFX,MUC1 |
| GO:2001236 | Regulation of extrinsic apoptotic signaling pathway | 17 | 161 | 4.02E-14 | TNFRSF10A,HGF,SERPINE1,TNFSF10,BMP4,TIMP3,TERT,FAS,CASP8,FASLG,MCL1,PTEN,AR,BCL2,GSTP1,TNF,BRCA1 |
| GO:2001237 | Negative regulation of extrinsic apoptotic signaling pathway | 15 | 106 | 4.34E-14 | TNFRSF10A,HGF,SERPINE1,TNFSF10,BMP4,TERT,FAS,CASP8,FASLG,MCL1,AR,BCL2,GSTP1,TNF,BRCA1 |
| GO:0051222 | Positive regulation of protein transport | 22 | 352 | 4.80E-14 | MAPK1,TGFB1,IFNG,MDM2,CDH1,TP63,TP53,ERBB2,EGFR,PAK1,E2F1,CASP8,PTGS2,TLR4,TP73,JAK2,SIRT3,YWHAZ,BCL2,IL6,TNF,HIF1A |
| GO:0009888 | Tissue development | 43 | 1760 | 5.36E-14 | MAPK1,TGFB1,HGF,CCND1,DUSP1,BMP4,ALDH1A2,MDM2,NOX4,PIK3CA,TP63,RB1,TP53,EGFR,NOTCH1,PAK1,ADAM17,PRKDC,TYMS,MET,CXCR2,EZH2,SOX2,RARB,BSG,CTNNB1,STAT1,MTOR,PTGS2,S100A4,PTEN,AR,POSTN,JAK2,ERCC2,SPP1,BCL2,RUNX3,CDKN1A,CD44,HIF1A,CYP1B1,VEGFA |
| GO:0043549 | Regulation of kinase activity | 32 | 918 | 5.89E-14 | MAPK1,TNFRSF10A,TGFB1,HGF,CCND1,IFNG,DUSP1,BMP4,GDF15,NOX4,PIK3CA,RB1,IGF1R,ERBB2,EGFR,PAK1,AXL,ADAM17,MET,EZH2,STK11,MTOR,PTEN,TLR4,TP73,JAK2,XRCC5,GSTP1,CDKN1A,TNF,CDKN2A,VEGFA |
| GO:0032496 | Response to lipopolysaccharide | 21 | 313 | 6.06E-14 | MAPK1,TGFB1,SERPINE1,NOTCH1,AXL,ADAM17,NOS2,HMGB1,CASP8,PTGS2,FASLG,ABCC2,UGT1A1,TLR4,CD274,JAK2,HSPD1,GSTP1,IL6,TNF,ZFP36 |
| GO:0007417 | Central nervous system development | 33 | 988 | 6.21E-14 | MAPK1,IFNG,BMP4,SIRT2,ALDH1A2,CDH1,XRCC1,STAT3,TP53,ERBB2,EGFR,NOTCH1,PAK1,BAX,AXL,PRKDC,CXCR2,EZH2,SOX2,HSPA5,RARB,CTNNB1,E2F1,MTOR,PTEN,TLR4,ERCC2,XRCC5,BCL2,GSTP1,TNF,H2AFX,HIF1A |
| GO:0040008 | Regulation of growth | 28 | 676 | 8.09E-14 | MAPK1,CSNK2A1,TGFB1,IFNG,BMP4,GDF15,PIK3CA,STAT3,RB1,TP53,ERBB2,EGFR,NOTCH1,PAK1,ADAM17,PRKDC,STK11,MTOR,PTEN,AR,TP73,IGFBP3,SPP1,BCL2,CDKN1A,CDKN2A,HIF1A,VEGFA |
| GO:0032880 | Regulation of protein localization | 32 | 934 | 9.27E-14 | MAPK1,TGFB1,IFNG,BMP4,MDM2,CDH1,TP63,RB1,TP53,ERBB2,EGFR,PAK1,TERT,STK11,NOS2,CTNNB1,E2F1,CASP8,PTGS2,TLR4,AR,TP73,JAK2,SIRT3,YWHAZ,BCL2,IL6,TNF,CDKN2A,HIF1A,SLC16A1,VEGFA |
| GO:0001819 | Positive regulation of cytokine production | 24 | 461 | 9.53E-14 | TGFB1,HGF,SERPINE1,IFNG,STAT3,ADAM17,PRKDC,BSG,CTNNB1,HMGB1,CASP8,STAT1,PTGS2,TLR4,POSTN,CD274,JAK2,HSPD1,XRCC5,IL6,TNF,BRCA1,HIF1A,CYP1B1 |
| GO:0001817 | Regulation of cytokine production | 29 | 742 | 9.72E-14 | TGFB1,HGF,SERPINE1,IFNG,STAT3,AXL,ADAM17,PRKDC,NOS2,BSG,CTNNB1,HMGB1,CASP8,STAT1,PTGS2,TLR4,POSTN,CD274,JAK2,HSPD1,XRCC5,GSTP1,IL6,TNF,BRCA1,HIF1A,ZFP36,BIRC2,CYP1B1 |
| GO:0033674 | Positive regulation of kinase activity | 27 | 624 | 1.00E-13 | MAPK1,TNFRSF10A,TGFB1,HGF,CCND1,IFNG,BMP4,GDF15,NOX4,PIK3CA,IGF1R,ERBB2,EGFR,PAK1,AXL,ADAM17,MET,EZH2,STK11,MTOR,TLR4,TP73,JAK2,XRCC5,CDKN1A,TNF,VEGFA |
| GO:1903827 | Regulation of cellular protein localization | 26 | 568 | 1.00E-13 | MAPK1,TGFB1,IFNG,BMP4,MDM2,CDH1,TP63,RB1,TP53,ERBB2,EGFR,PAK1,TERT,STK11,CTNNB1,E2F1,CASP8,PTGS2,AR,TP73,JAK2,YWHAZ,BCL2,TNF,CDKN2A,VEGFA |
| GO:0032870 | Cellular response to hormone stimulus | 26 | 569 | 1.04E-13 | APEX1,DUSP1,BMP4,MDM2,PIK3CA,STAT3,RB1,IGF1R,EGFR,NOTCH1,PAK1,PRKDC,RARB,CTNNB1,STAT1,ABCC2,PTEN,UGT1A1,AR,JAK2,NFE2L2,GSTP1,BRCA1,PDK2,EIF4E,ZFP36 |
| GO:0035556 | Intracellular signal transduction | 42 | 1712 | 1.09E-13 | MAPK1,TNFRSF10A,TGFB1,HGF,MLH1,DUSP1,SIRT2,MDM2,PIK3CA,TP63,RB1,IGF1R,TP53,ERBB2,EGFR,PAK1,BAX,AXL,PRKDC,MET,CXCR2,STK11,NOS2,E2F1,CASP8,MTOR,MCL1,PTEN,TLR4,TP73,JAK2,YWHAZ,BCL2,CDKN1A,TNF,CDKN2A,BRCA1,PDK2,ZFP36,BIRC2,CYP1B1,MUC1 |
| GO:0032355 | Response to estradiol | 16 | 142 | 1.11E-13 | TGFB1,CCND1,DUSP1,ALDH1A2,STAT3,EGFR,OGG1,EZH2,CTNNB1,CASP8,PTGS2,ABCC2,PTEN,UGT1A1,POSTN,GSTP1 |
| GO:0045859 | Regulation of protein kinase activity | 30 | 812 | 1.19E-13 | MAPK1,TNFRSF10A,TGFB1,HGF,CCND1,IFNG,DUSP1,BMP4,GDF15,NOX4,PIK3CA,RB1,IGF1R,ERBB2,EGFR,PAK1,ADAM17,EZH2,STK11,MTOR,PTEN,TLR4,TP73,JAK2,XRCC5,GSTP1,CDKN1A,TNF,CDKN2A,VEGFA |
| GO:2000113 | Negative regulation of cellular macromolecule biosynthetic process | 39 | 1470 | 1.29E-13 | TGFB1,CCND1,IFNG,DUSP1,BMP4,SIRT2,MDM2,STAT3,TP63,RB1,TP53,NOTCH1,BIRC5,ERCC4,TYMS,USP9X,EZH2,SOX2,RARB,MTA1,CTNNB1,HMGB1,E2F1,STAT1,FASLG,HDGF,AR,BMI1,XRCC5,RUNX3,CDKN1A,TNF,CDKN2A,BRCA1,EIF4E,H2AFX,ZFP36,VEGFA,MUC1 |
| GO:0071900 | Regulation of protein serine/threonine kinase activity | 25 | 521 | 1.31E-13 | MAPK1,TGFB1,HGF,CCND1,IFNG,DUSP1,BMP4,GDF15,NOX4,RB1,IGF1R,ERBB2,EGFR,PAK1,ADAM17,EZH2,PTEN,TLR4,TP73,JAK2,GSTP1,CDKN1A,TNF,CDKN2A,VEGFA |
| GO:0044419 | Interspecies interaction between organisms | 44 | 1899 | 1.34E-13 | MAPK1,TGFB1,SERPINE1,IFNG,MLH1,SIRT2,MDM2,CDH1,STAT3,RB1,TP53,EGFR,NOTCH1,BAX,AXL,ADAM17,PRKDC,MET,NOS2,CTNNB1,HMGB1,E2F1,CASP8,STAT1,PTGS2,FASLG,ABCC2,UGT1A1,TLR4,TP73,CD274,JAK2,HSPD1,ERCC2,XRCC5,NFE2L2,BCL2,GSTP1,IL6,CD44,TNF,EIF4E,H2AFX,ZFP36 |
| GO:0048608 | Reproductive structure development | 23 | 420 | 1.35E-13 | ERCC1,MAPK1,CCND1,TNFSF10,BMP4,EPAS1,TP63,EGFR,NOTCH1,BAX,AXL,HSPA5,BSG,CTNNB1,CASP8,PTGS2,PTEN,AR,SPP1,BCL2,HIF1A,BIRC2,VEGFA |
| GO:0070848 | Response to growth factor | 25 | 524 | 1.47E-13 | MAPK1,TGFB1,HGF,BMP4,SIRT2,GDF15,MDM2,NOX4,TP53,ERBB2,EGFR,NOTCH1,USP9X,SOX2,HSPA5,CTNNB1,E2F1,FASLG,PTEN,POSTN,GSTP1,RUNX3,CD44,ZFP36,VEGFA |
| GO:0045944 | Positive regulation of transcription by rna polymerase ii | 36 | 1253 | 1.95E-13 | ERCC1,TGFB1,HGF,SERPINE1,BMP4,SIRT2,EPAS1,STAT3,TP63,RB1,TP53,EGFR,NOTCH1,TERT,PRKDC,MET,SOX2,HSPA5,RARB,CTNNB1,HMGB1,E2F1,STAT1,TLR4,AR,TP73,TP53BP1,NFE2L2,IL6,TNF,TOP2A,CDKN2A,BRCA1,HIF1A,VEGFA,MUC1 |
| GO:0051253 | Negative regulation of rna metabolic process | 38 | 1422 | 2.57E-13 | APEX1,TGFB1,CCND1,IFNG,BMP4,SIRT2,MDM2,STAT3,TP63,RB1,TP53,NOTCH1,BIRC5,TERT,USP9X,EZH2,SOX2,RARB,MTA1,CTNNB1,HMGB1,E2F1,STAT1,MTOR,FASLG,HDGF,AR,BMI1,XRCC5,RUNX3,IL6,TNF,CDKN2A,BRCA1,H2AFX,ZFP36,VEGFA,MUC1 |
| GO:0097193 | Intrinsic apoptotic signaling pathway | 16 | 151 | 2.57E-13 | MLH1,TP63,TP53,BAX,PRKDC,STK11,E2F1,MCL1,TP73,JAK2,BCL2,CDKN1A,TNF,BRCA1,PDK2,CYP1B1 |
| GO:0030155 | Regulation of cell adhesion | 28 | 712 | 2.61E-13 | LGALS1,TGFB1,SERPINE1,IFNG,DUSP1,BMP4,CDH1,PIK3CA,ERBB2,NOTCH1,PAK1,SOX2,PDCD1,HMGB1,PTEN,POSTN,CD274,JAK2,HSPD1,BCL2,RUNX3,IL6,CD44,TNF,CDKN2A,CYP1B1,VEGFA,MUC1 |
| GO:0031327 | Negative regulation of cellular biosynthetic process | 40 | 1592 | 2.85E-13 | APEX1,TGFB1,CCND1,IFNG,DUSP1,BMP4,SIRT2,MDM2,STAT3,TP63,RB1,TP53,NOTCH1,BIRC5,ERCC4,TYMS,USP9X,EZH2,SOX2,RARB,MTA1,CTNNB1,HMGB1,E2F1,STAT1,FASLG,HDGF,AR,BMI1,XRCC5,RUNX3,CDKN1A,TNF,CDKN2A,BRCA1,EIF4E,H2AFX,ZFP36,VEGFA,MUC1 |
| GO:0045787 | Positive regulation of cell cycle | 22 | 388 | 2.88E-13 | APEX1,CCND1,SIRT2,MDM2,TP63,RB1,TP53,EGFR,BAX,BIRC5,TERT,ADAM17,EZH2,RAD51C,E2F1,TP73,CDKN1A,CDKN2A,BRCA1,EIF4E,XRCC3,MUC1 |
| GO:0043410 | Positive regulation of mapk cascade | 25 | 543 | 3.10E-13 | MAPK1,TGFB1,HGF,BMP4,GDF15,NOX4,IGF1R,ERBB2,EGFR,NOTCH1,PAK1,EZH2,SOX2,CTNNB1,HMGB1,PTEN,TLR4,AR,TP73,IGFBP3,JAK2,IL6,CD44,TNF,VEGFA |
| GO:0035295 | Tube development | 30 | 851 | 3.76E-13 | MAPK1,TGFB1,SERPINE1,BMP4,ALDH1A2,EPAS1,PIK3CA,TP63,RB1,EGFR,NOTCH1,PAK1,BAX,TYMS,MET,CXCR2,RARB,CTNNB1,STAT1,PTGS2,PTEN,AR,YWHAZ,BCL2,CDKN1A,TNF,EIF4E,HIF1A,CYP1B1,VEGFA |
| GO:0010720 | Positive regulation of cell development | 25 | 556 | 5.15E-13 | TGFB1,HGF,IFNG,BMP4,SIRT2,NOTCH1,PAK1,PRKDC,EZH2,HSPA5,STK11,RARB,CTNNB1,E2F1,MTOR,PTEN,TP73,KDM4C,XRCC5,NFE2L2,BCL2,IL6,TNF,HIF1A,VEGFA |
| GO:0007584 | Response to nutrient | 16 | 160 | 5.77E-13 | ERCC1,CCND1,ALDH1A2,MDM2,EGFR,OGG1,TYMS,STAT1,MTOR,PTGS2,UGT1A1,POSTN,SPP1,GSTP1,PDK2,SLC16A1 |
| GO:0051223 | Regulation of protein transport | 26 | 617 | 5.97E-13 | MAPK1,TGFB1,IFNG,BMP4,MDM2,CDH1,TP63,TP53,ERBB2,EGFR,PAK1,NOS2,E2F1,CASP8,PTGS2,TLR4,TP73,JAK2,SIRT3,YWHAZ,BCL2,IL6,TNF,CDKN2A,HIF1A,SLC16A1 |
| GO:2000379 | Positive regulation of reactive oxygen species metabolic process | 14 | 103 | 6.03E-13 | TGFB1,IFNG,NOX4,TP53,EGFR,MTOR,PTGS2,TLR4,JAK2,SIRT3,NFE2L2,GSTP1,CDKN1A,TNF |
| GO:0071214 | Cellular response to abiotic stimulus | 20 | 313 | 6.53E-13 | ERCC1,TNFRSF10A,MDM2,NOX4,TP53,EGFR,BAX,ERCC4,HSPA5,STK11,FAS,CASP8,PTGS2,PTEN,TLR4,TP53BP1,RAD51,XRCC5,CDKN1A,H2AFX |
| GO:0031960 | Response to corticosteroid | 16 | 164 | 8.07E-13 | CCND1,DUSP1,EGFR,NOTCH1,TYMS,PTGS2,ABCC2,UGT1A1,HSPD1,BCL2,GSTP1,CDKN1A,IL6,TNF,EIF4E,ZFP36 |
| GO:0044092 | Negative regulation of molecular function | 34 | 1163 | 8.09E-13 | CSNK2A1,HGF,SERPINE1,IFNG,DUSP1,BMP4,MDM2,TIMP3,RB1,IGF1R,TP53,NOTCH1,BAX,BIRC5,ERCC4,MET,EZH2,E2F1,CASP8,PTGS2,XIAP,PTEN,UGT1A1,JAK2,GSTP1,CDKN1A,CD44,TNF,CDKN2A,PDK2,ZFP36,BIRC2,CYP1B1,VEGFA |
| GO:0022414 | Reproductive process | 37 | 1400 | 8.69E-13 | ERCC1,MAPK1,CCND1,MLH1,TNFSF10,BMP4,SIRT2,EPAS1,STAT3,TP63,EGFR,NOTCH1,BAX,AXL,ERCC4,PRKDC,USP9X,HSPA5,STK11,BSG,RAD51C,CTNNB1,E2F1,CASP8,MTOR,PTGS2,ABCC2,PTEN,AR,RAD51,SPP1,BCL2,TOP2A,H2AFX,HIF1A,BIRC2,VEGFA |
| GO:0009987 | Cellular process | 120 | 15024 | 9.24E-13 | ERCC1,MAPK1,LGALS1,APEX1,CSNK2A1,TNFRSF10A,TGFB1,HGF,SERPINE1,CCND1,IFNG,MLH1,DUSP1,TNFSF10,BMP4,SIRT2,ALDH1A2,GDF15,MDM2,CDH1,XRCC1,NOX4,EPAS1,PIK3CA,STAT3,TP63,TIMP3,RB1,IGF1R,TP53,ERBB2,EGFR,NOTCH1,PAK1,BAX,ALDH1A1,AXL,BIRC5,MGMT,OGG1,TERT,ADAM17,ERCC4,GSTM1,PRKDC,TYMS,USP9X,MET,CXCR2,EZH2,SOX2,HSPA5,STK11,NOS2,RARB,MTA1,BSG,PDCD1,RAD51C,CTNNB1,HMGB1,E2F1,ERCC5,FAS,CASP8,STAT1,MTOR,PTGS2,FASLG,HDGF,MKI67,S100A4,MCL1,ABCC2,XIAP,PTEN,ABCC10,UGT1A1,TLR4,AR,BMI1,TP73,POSTN,IGFBP3,KDM4C,CD274,JAK2,TP53BP1,RAD51,SIRT3,GPX3,HSPD1,ERCC2,XRCC5,SPP1,YWHAZ,NFE2L2,BCL2,GSTP1,ABCC1,RUNX3,CDKN1A,IL6,CD44,TNF,TOP2A,CDKN2A,BRCA1,PDK2,EIF4E,H2AFX,HIF1A,SLC16A1,SOD2,XRCC3,ZFP36,BIRC2,CYP1B1,VEGFA,MUC1 |
| GO:0043412 | Macromolecule modification | 56 | 3277 | 1.63E-12 | MAPK1,LGALS1,APEX1,CSNK2A1,TGFB1,HGF,CCND1,DUSP1,BMP4,SIRT2,MDM2,EPAS1,PIK3CA,IGF1R,TP53,ERBB2,EGFR,PAK1,AXL,BIRC5,MGMT,OGG1,PRKDC,USP9X,MET,EZH2,STK11,NOS2,MTA1,CTNNB1,MTOR,XIAP,PTEN,TLR4,AR,BMI1,IGFBP3,KDM4C,JAK2,SIRT3,ERCC2,SPP1,YWHAZ,NFE2L2,BCL2,RUNX3,CDKN1A,IL6,TNF,CDKN2A,BRCA1,PDK2,HIF1A,ZFP36,BIRC2,MUC1 |
| GO:0022402 | Cell cycle process | 31 | 976 | 1.76E-12 | ERCC1,TGFB1,CCND1,IFNG,MLH1,DUSP1,MDM2,RB1,TP53,NOTCH1,BAX,BIRC5,ERCC4,PRKDC,TYMS,EZH2,SOX2,STK11,RAD51C,CTNNB1,E2F1,MTOR,TP73,RAD51,CDKN1A,TOP2A,CDKN2A,BRCA1,EIF4E,SLC16A1,MUC1 |
| GO:0050769 | Positive regulation of neurogenesis | 23 | 479 | 1.76E-12 | TGFB1,HGF,IFNG,BMP4,NOTCH1,PAK1,EZH2,HSPA5,STK11,RARB,CTNNB1,E2F1,MTOR,PTEN,TP73,KDM4C,XRCC5,NFE2L2,BCL2,IL6,TNF,HIF1A,VEGFA |
| GO:1901342 | Regulation of vasculature development | 20 | 336 | 2.20E-12 | HGF,SERPINE1,BMP4,STAT3,ERBB2,NOTCH1,TERT,CXCR2,CTNNB1,STAT1,PTGS2,FASLG,NFE2L2,IL6,TNF,BRCA1,HIF1A,SOD2,CYP1B1,VEGFA |
| GO:0051384 | Response to glucocorticoid | 15 | 147 | 2.93E-12 | CCND1,DUSP1,EGFR,TYMS,PTGS2,ABCC2,UGT1A1,HSPD1,BCL2,GSTP1,CDKN1A,IL6,TNF,EIF4E,ZFP36 |
| GO:0045860 | Positive regulation of protein kinase activity | 24 | 550 | 3.31E-12 | MAPK1,TNFRSF10A,TGFB1,HGF,CCND1,IFNG,BMP4,GDF15,NOX4,PIK3CA,ERBB2,EGFR,PAK1,ADAM17,EZH2,STK11,MTOR,TLR4,TP73,JAK2,XRCC5,CDKN1A,TNF,VEGFA |
| GO:0033043 | Regulation of organelle organization | 35 | 1306 | 3.53E-12 | ERCC1,MAPK1,HGF,DUSP1,TNFSF10,BMP4,SIRT2,XRCC1,NOX4,TP63,RB1,TP53,PAK1,BAX,BIRC5,ERCC4,MET,CTNNB1,E2F1,CASP8,MTOR,MKI67,TP73,KDM4C,XRCC5,YWHAZ,BCL2,TNF,TOP2A,CDKN2A,BRCA1,HIF1A,XRCC3,VEGFA,MUC1 |
| GO:0051249 | Regulation of lymphocyte activation | 22 | 445 | 3.76E-12 | LGALS1,TGFB1,IFNG,MLH1,BMP4,PIK3CA,ERBB2,PAK1,AXL,PRKDC,PDCD1,CTNNB1,HMGB1,TLR4,CD274,TP53BP1,HSPD1,BCL2,RUNX3,CDKN1A,IL6,CDKN2A |
| GO:0090316 | Positive regulation of intracellular protein transport | 16 | 184 | 4.00E-12 | MAPK1,TGFB1,IFNG,MDM2,CDH1,TP63,TP53,ERBB2,PAK1,E2F1,CASP8,PTGS2,TP73,JAK2,YWHAZ,BCL2 |
| GO:0008630 | Intrinsic apoptotic signaling pathway in response to dna damage | 12 | 71 | 4.65E-12 | MLH1,TP63,TP53,BAX,PRKDC,E2F1,MCL1,TP73,BCL2,CDKN1A,TNF,BRCA1 |
| GO:0043086 | Negative regulation of catalytic activity | 28 | 807 | 4.71E-12 | CSNK2A1,HGF,SERPINE1,IFNG,DUSP1,BMP4,MDM2,TIMP3,RB1,IGF1R,TP53,NOTCH1,BIRC5,ERCC4,CASP8,PTGS2,XIAP,PTEN,UGT1A1,GSTP1,CDKN1A,CD44,TNF,CDKN2A,PDK2,ZFP36,BIRC2,VEGFA |
| GO:0003006 | Developmental process involved in reproduction | 30 | 945 | 4.87E-12 | ERCC1,MAPK1,CCND1,MLH1,TNFSF10,BMP4,EPAS1,TP63,EGFR,NOTCH1,BAX,AXL,PRKDC,HSPA5,STK11,BSG,RAD51C,CTNNB1,E2F1,CASP8,MTOR,PTGS2,PTEN,AR,SPP1,BCL2,H2AFX,HIF1A,BIRC2,VEGFA |
| GO:1903507 | Negative regulation of nucleic acid-templated transcription | 35 | 1324 | 5.11E-12 | APEX1,TGFB1,CCND1,IFNG,BMP4,SIRT2,MDM2,STAT3,TP63,RB1,TP53,NOTCH1,BIRC5,USP9X,EZH2,SOX2,RARB,MTA1,CTNNB1,HMGB1,E2F1,STAT1,FASLG,HDGF,AR,BMI1,XRCC5,RUNX3,TNF,CDKN2A,BRCA1,H2AFX,ZFP36,VEGFA,MUC1 |
| GO:0022603 | Regulation of anatomical structure morphogenesis | 32 | 1095 | 5.46E-12 | TGFB1,HGF,SERPINE1,BMP4,GDF15,STAT3,ERBB2,NOTCH1,PAK1,BAX,TERT,PRKDC,CXCR2,STK11,CTNNB1,STAT1,MTOR,PTGS2,FASLG,PTEN,AR,POSTN,SPP1,NFE2L2,BCL2,IL6,CD44,TNF,BRCA1,HIF1A,CYP1B1,VEGFA |
| GO:0060341 | Regulation of cellular localization | 31 | 1027 | 6.27E-12 | MAPK1,TGFB1,IFNG,BMP4,MDM2,CDH1,TP63,RB1,TP53,ERBB2,EGFR,PAK1,BAX,TERT,STK11,CTNNB1,E2F1,CASP8,PTGS2,FASLG,PTEN,AR,TP73,JAK2,YWHAZ,BCL2,TNF,CDKN2A,HIF1A,ZFP36,VEGFA |
| GO:0033157 | Regulation of intracellular protein transport | 18 | 268 | 6.29E-12 | MAPK1,TGFB1,IFNG,BMP4,MDM2,CDH1,TP63,TP53,ERBB2,PAK1,E2F1,CASP8,PTGS2,TP73,JAK2,YWHAZ,BCL2,CDKN2A |
| GO:0006464 | Cellular protein modification process | 53 | 3063 | 7.23E-12 | MAPK1,LGALS1,CSNK2A1,TGFB1,HGF,CCND1,DUSP1,BMP4,SIRT2,MDM2,EPAS1,PIK3CA,IGF1R,TP53,ERBB2,EGFR,PAK1,AXL,BIRC5,PRKDC,USP9X,MET,EZH2,STK11,NOS2,MTA1,CTNNB1,MTOR,XIAP,PTEN,TLR4,AR,BMI1,IGFBP3,KDM4C,JAK2,SIRT3,ERCC2,SPP1,YWHAZ,NFE2L2,BCL2,RUNX3,CDKN1A,IL6,TNF,CDKN2A,BRCA1,PDK2,HIF1A,ZFP36,BIRC2,MUC1 |
| GO:0010638 | Positive regulation of organelle organization | 25 | 633 | 7.57E-12 | ERCC1,MAPK1,TNFSF10,SIRT2,NOX4,TP63,RB1,TP53,PAK1,BAX,BIRC5,MET,CTNNB1,E2F1,CASP8,MTOR,TP73,XRCC5,YWHAZ,BCL2,TNF,BRCA1,HIF1A,VEGFA,MUC1 |
| GO:0009894 | Regulation of catabolic process | 31 | 1038 | 8.11E-12 | APEX1,CSNK2A1,HGF,IFNG,SIRT2,MDM2,CDH1,PIK3CA,STAT3,TIMP3,RB1,TP53,EGFR,BAX,ERCC4,MET,STK11,NOS2,HMGB1,E2F1,MTOR,MCL1,PTEN,YWHAZ,NFE2L2,BCL2,IL6,TNF,CDKN2A,HIF1A,ZFP36 |
| GO:1901214 | Regulation of neuron death | 19 | 317 | 8.39E-12 | IFNG,PIK3CA,STAT3,TP53,BAX,AXL,TERT,CTNNB1,CASP8,MTOR,FASLG,MCL1,TLR4,JAK2,HSPD1,BCL2,TNF,HIF1A,SOD2 |
| GO:0045892 | Negative regulation of transcription, dna-templated | 34 | 1273 | 9.22E-12 | TGFB1,CCND1,IFNG,BMP4,SIRT2,MDM2,STAT3,TP63,RB1,TP53,NOTCH1,BIRC5,USP9X,EZH2,SOX2,RARB,MTA1,CTNNB1,HMGB1,E2F1,STAT1,FASLG,HDGF,AR,BMI1,XRCC5,RUNX3,TNF,CDKN2A,BRCA1,H2AFX,ZFP36,VEGFA,MUC1 |
| GO:0000302 | Response to reactive oxygen species | 16 | 198 | 1.09E-11 | MAPK1,APEX1,DUSP1,MDM2,EGFR,AXL,EZH2,STAT1,HSPD1,NFE2L2,BCL2,GSTP1,IL6,PDK2,SOD2,CYP1B1 |
| GO:0007162 | Negative regulation of cell adhesion | 18 | 280 | 1.24E-11 | LGALS1,TGFB1,SERPINE1,DUSP1,BMP4,CDH1,ERBB2,NOTCH1,HMGB1,PTEN,POSTN,CD274,JAK2,RUNX3,CDKN2A,CYP1B1,VEGFA,MUC1 |
| GO:1901652 | Response to peptide | 22 | 476 | 1.29E-11 | APEX1,TNFSF10,MDM2,PIK3CA,STAT3,IGF1R,TP53,NOTCH1,PAK1,PRKDC,BSG,STAT1,MTOR,PTGS2,ABCC2,PTEN,TLR4,JAK2,NFE2L2,GSTP1,ABCC1,PDK2 |
| GO:0022407 | Regulation of cell-cell adhesion | 21 | 424 | 1.33E-11 | LGALS1,TGFB1,IFNG,BMP4,CDH1,PIK3CA,ERBB2,NOTCH1,PAK1,SOX2,PDCD1,HMGB1,CD274,JAK2,HSPD1,RUNX3,IL6,CD44,TNF,CDKN2A,VEGFA |
| GO:0010332 | Response to gamma radiation | 11 | 58 | 1.59E-11 | MDM2,NOX4,TP53,BAX,PRKDC,HSPA5,RAD51,XRCC5,BCL2,CDKN1A,H2AFX |
| GO:0010721 | Negative regulation of cell development | 19 | 330 | 1.61E-11 | LGALS1,SIRT2,MDM2,STAT3,TP53,NOTCH1,PAK1,TERT,SOX2,CTNNB1,PTEN,TP73,POSTN,SPP1,BCL2,IL6,TNF,EIF4E,VEGFA |
| GO:0051050 | Positive regulation of transport | 29 | 923 | 1.65E-11 | MAPK1,TGFB1,SERPINE1,IFNG,MDM2,CDH1,TP63,TP53,ERBB2,EGFR,PAK1,BAX,AXL,TERT,E2F1,CASP8,PTGS2,TLR4,TP73,JAK2,SIRT3,SPP1,YWHAZ,NFE2L2,BCL2,IL6,TNF,HIF1A,VEGFA |
| GO:0051251 | Positive regulation of lymphocyte activation | 18 | 286 | 1.70E-11 | LGALS1,TGFB1,IFNG,MLH1,PIK3CA,PAK1,AXL,PRKDC,PDCD1,HMGB1,TLR4,CD274,TP53BP1,HSPD1,BCL2,RUNX3,CDKN1A,IL6 |
| GO:2000134 | Negative regulation of g1/s transition of mitotic cell cycle | 13 | 107 | 1.78E-11 | CCND1,MDM2,RB1,TP53,BAX,PRKDC,EZH2,E2F1,PTEN,BCL2,CDKN1A,CDKN2A,MUC1 |
| GO:0031400 | Negative regulation of protein modification process | 24 | 602 | 1.96E-11 | TGFB1,HGF,IFNG,DUSP1,BMP4,SIRT2,XRCC1,TIMP3,RB1,IGF1R,BAX,PRKDC,CTNNB1,MTOR,PTEN,TLR4,IGFBP3,KDM4C,SIRT3,GSTP1,CDKN1A,TNF,CDKN2A,BRCA1 |
| GO:1903706 | Regulation of hemopoiesis | 22 | 493 | 2.44E-11 | TGFB1,IFNG,BMP4,STAT3,RB1,ERBB2,NOTCH1,AXL,PRKDC,CTNNB1,HMGB1,CASP8,STAT1,MTOR,TLR4,TP73,NFE2L2,RUNX3,TNF,CDKN2A,HIF1A,ZFP36 |
| GO:0071363 | Cellular response to growth factor stimulus | 22 | 494 | 2.53E-11 | MAPK1,TGFB1,HGF,BMP4,SIRT2,GDF15,MDM2,NOX4,TP53,ERBB2,EGFR,NOTCH1,USP9X,HSPA5,CTNNB1,E2F1,PTEN,POSTN,GSTP1,CD44,ZFP36,VEGFA |
| GO:0031329 | Regulation of cellular catabolic process | 28 | 875 | 2.89E-11 | APEX1,CSNK2A1,HGF,IFNG,SIRT2,MDM2,PIK3CA,STAT3,TIMP3,RB1,TP53,BAX,ERCC4,MET,STK11,HMGB1,E2F1,MTOR,MCL1,PTEN,YWHAZ,NFE2L2,BCL2,IL6,TNF,CDKN2A,HIF1A,ZFP36 |
| GO:0014910 | Regulation of smooth muscle cell migration | 11 | 62 | 2.92E-11 | APEX1,SERPINE1,MDM2,NOX4,PAK1,TERT,POSTN,IGFBP3,NFE2L2,BCL2,GSTP1 |
| GO:0050867 | Positive regulation of cell activation | 19 | 346 | 3.44E-11 | LGALS1,TGFB1,IFNG,MLH1,PIK3CA,PAK1,AXL,PRKDC,PDCD1,HMGB1,TLR4,CD274,JAK2,TP53BP1,HSPD1,BCL2,RUNX3,CDKN1A,IL6 |
| GO:0016043 | Cellular component organization | 71 | 5447 | 3.69E-11 | ERCC1,MAPK1,APEX1,TGFB1,SERPINE1,MLH1,SIRT2,ALDH1A2,MDM2,CDH1,XRCC1,EPAS1,PIK3CA,STAT3,TP63,RB1,IGF1R,TP53,ERBB2,EGFR,NOTCH1,PAK1,BAX,BIRC5,TERT,ERCC4,PRKDC,USP9X,EZH2,SOX2,STK11,NOS2,MTA1,BSG,RAD51C,CTNNB1,HMGB1,E2F1,ERCC5,FAS,CASP8,MTOR,FASLG,PTEN,BMI1,TP73,POSTN,KDM4C,JAK2,TP53BP1,RAD51,SIRT3,HSPD1,ERCC2,XRCC5,SPP1,YWHAZ,BCL2,ABCC1,IL6,CD44,TNF,TOP2A,CDKN2A,BRCA1,H2AFX,SLC16A1,SOD2,XRCC3,CYP1B1,VEGFA |
| GO:0006259 | DNA metabolic process | 26 | 750 | 3.83E-11 | ERCC1,APEX1,MLH1,XRCC1,TP53,MGMT,OGG1,TERT,ERCC4,PRKDC,TYMS,EZH2,MTA1,RAD51C,HMGB1,ERCC5,TP73,TP53BP1,RAD51,HSPD1,ERCC2,XRCC5,TOP2A,BRCA1,H2AFX,XRCC3 |
| GO:0009416 | Response to light stimulus | 18 | 302 | 3.90E-11 | ERCC1,CCND1,DUSP1,MDM2,TP53,EGFR,BAX,OGG1,ERCC4,STK11,MTA1,ERCC5,MTOR,PTGS2,ERCC2,BCL2,CDKN1A,HIF1A |
| GO:0033135 | Regulation of peptidyl-serine phosphorylation | 14 | 146 | 4.04E-11 | TGFB1,HGF,IFNG,PIK3CA,EGFR,PAK1,BAX,PTGS2,PTEN,BCL2,IL6,CD44,TNF,VEGFA |
| GO:0045765 | Regulation of angiogenesis | 18 | 303 | 4.09E-11 | HGF,SERPINE1,STAT3,ERBB2,NOTCH1,TERT,CXCR2,CTNNB1,STAT1,PTGS2,FASLG,NFE2L2,IL6,TNF,BRCA1,HIF1A,CYP1B1,VEGFA |
| GO:1902532 | Negative regulation of intracellular signal transduction | 22 | 508 | 4.19E-11 | DUSP1,BMP4,MDM2,TIMP3,IGF1R,MET,STK11,CASP8,STAT1,MTOR,PTGS2,MCL1,PTEN,TLR4,SIRT3,NFE2L2,BCL2,GSTP1,CD44,HIF1A,SOD2,MUC1 |
| GO:0071216 | Cellular response to biotic stimulus | 16 | 219 | 4.28E-11 | MAPK1,TGFB1,SERPINE1,SIRT2,TP53,AXL,HSPA5,NOS2,HMGB1,ABCC2,TLR4,CD274,GSTP1,IL6,TNF,ZFP36 |
| GO:0032103 | Positive regulation of response to external stimulus | 22 | 511 | 4.65E-11 | MAPK1,TGFB1,SERPINE1,IFNG,STAT3,EGFR,PAK1,ADAM17,PRKDC,MET,CXCR2,HMGB1,PTGS2,TLR4,JAK2,HSPD1,XRCC5,ABCC1,IL6,TNF,VEGFA,MUC1 |
| GO:0000122 | Negative regulation of transcription by rna polymerase ii | 28 | 895 | 4.74E-11 | TGFB1,CCND1,IFNG,BMP4,SIRT2,MDM2,STAT3,TP63,RB1,TP53,NOTCH1,USP9X,EZH2,SOX2,RARB,MTA1,CTNNB1,HMGB1,E2F1,STAT1,FASLG,HDGF,AR,BMI1,RUNX3,TNF,ZFP36,VEGFA |
| GO:0050865 | Regulation of cell activation | 23 | 571 | 4.96E-11 | LGALS1,TGFB1,IFNG,MLH1,BMP4,PIK3CA,ERBB2,PAK1,AXL,PRKDC,PDCD1,CTNNB1,HMGB1,TLR4,CD274,JAK2,TP53BP1,HSPD1,BCL2,RUNX3,CDKN1A,IL6,CDKN2A |
| GO:0007569 | Cell aging | 11 | 66 | 5.18E-11 | ERCC1,SERPINE1,NOX4,TP63,TP53,TERT,MTOR,BCL2,CDKN1A,CDKN2A,H2AFX |
| GO:0071496 | Cellular response to external stimulus | 18 | 309 | 5.46E-11 | MAPK1,TNFRSF10A,MDM2,TP53,EGFR,AXL,HSPA5,FAS,CASP8,MTOR,PTGS2,TLR4,POSTN,NFE2L2,BCL2,GSTP1,CDKN1A,PDK2 |
| GO:0044267 | Cellular protein metabolic process | 57 | 3696 | 5.62E-11 | MAPK1,LGALS1,CSNK2A1,TGFB1,HGF,CCND1,DUSP1,BMP4,SIRT2,MDM2,EPAS1,PIK3CA,IGF1R,TP53,ERBB2,EGFR,PAK1,AXL,BIRC5,ADAM17,PRKDC,USP9X,MET,EZH2,HSPA5,STK11,NOS2,MTA1,CTNNB1,CASP8,MTOR,XIAP,PTEN,TLR4,AR,BMI1,IGFBP3,KDM4C,JAK2,SIRT3,ERCC2,SPP1,YWHAZ,NFE2L2,BCL2,RUNX3,CDKN1A,IL6,TNF,CDKN2A,BRCA1,PDK2,EIF4E,HIF1A,ZFP36,BIRC2,MUC1 |
| GO:0051336 | Regulation of hydrolase activity | 33 | 1284 | 5.79E-11 | CSNK2A1,TNFRSF10A,HGF,SERPINE1,IFNG,TNFSF10,MDM2,STAT3,TP63,TIMP3,TP53,ERBB2,EGFR,BAX,ALDH1A1,BIRC5,EZH2,SOX2,HMGB1,FAS,CASP8,MTOR,PTGS2,FASLG,XIAP,IGFBP3,JAK2,HSPD1,CD44,TNF,CDKN2A,BIRC2,VEGFA |
| GO:1901564 | Organonitrogen compound metabolic process | 69 | 5244 | 6.96E-11 | MAPK1,LGALS1,CSNK2A1,TGFB1,HGF,CCND1,DUSP1,BMP4,SIRT2,MDM2,NOX4,EPAS1,PIK3CA,IGF1R,TP53,ERBB2,EGFR,PAK1,BAX,AXL,BIRC5,OGG1,ADAM17,GSTM1,PRKDC,TYMS,USP9X,MET,EZH2,HSPA5,STK11,NOS2,MTA1,CTNNB1,CASP8,MTOR,PTGS2,ABCC2,XIAP,PTEN,UGT1A1,TLR4,AR,BMI1,IGFBP3,KDM4C,JAK2,SIRT3,HSPD1,ERCC2,SPP1,YWHAZ,NFE2L2,BCL2,GSTP1,ABCC1,RUNX3,CDKN1A,IL6,CD44,TNF,CDKN2A,BRCA1,PDK2,EIF4E,HIF1A,ZFP36,BIRC2,MUC1 |
| GO:0051129 | Negative regulation of cellular component organization | 25 | 713 | 8.48E-11 | ERCC1,LGALS1,TGFB1,HGF,BMP4,SIRT2,MDM2,XRCC1,TP53,NOTCH1,BIRC5,ERCC4,MET,HSPA5,HMGB1,PTEN,AR,KDM4C,XRCC5,SPP1,TNF,TOP2A,BRCA1,BIRC2,VEGFA |
| GO:0050679 | Positive regulation of epithelial cell proliferation | 15 | 192 | 8.99E-11 | CCND1,BMP4,STAT3,TP63,ERBB2,EGFR,NOTCH1,ADAM17,CTNNB1,HMGB1,MTOR,TLR4,AR,HIF1A,VEGFA |
| GO:0031099 | Regeneration | 14 | 157 | 9.58E-11 | HGF,CCND1,EGFR,NOTCH1,AXL,TYMS,EZH2,SOX2,UGT1A1,POSTN,JAK2,BCL2,GSTP1,CDKN1A |
| GO:0060322 | Head development | 26 | 788 | 1.07E-10 | MAPK1,BMP4,SIRT2,ALDH1A2,CDH1,XRCC1,TP53,EGFR,NOTCH1,PAK1,BAX,AXL,PRKDC,CXCR2,EZH2,SOX2,HSPA5,RARB,CTNNB1,E2F1,MTOR,PTEN,XRCC5,BCL2,H2AFX,HIF1A |
| GO:0048565 | Digestive tract development | 13 | 128 | 1.32E-10 | BMP4,ALDH1A2,TP63,RB1,EGFR,NOTCH1,TYMS,RARB,CTNNB1,BCL2,CDKN1A,TNF,HIF1A |
| GO:0000075 | Cell cycle checkpoint | 15 | 198 | 1.34E-10 | TGFB1,CCND1,MDM2,RB1,TP53,BAX,BIRC5,PRKDC,E2F1,TP53BP1,CDKN1A,TOP2A,BRCA1,H2AFX,MUC1 |
| GO:0010948 | Negative regulation of cell cycle process | 18 | 328 | 1.36E-10 | CCND1,BMP4,MDM2,RB1,TP53,BAX,BIRC5,PRKDC,EZH2,E2F1,PTEN,BMI1,RAD51,BCL2,CDKN1A,CDKN2A,BRCA1,MUC1 |
| GO:0051049 | Regulation of transport | 38 | 1776 | 1.70E-10 | MAPK1,TGFB1,SERPINE1,IFNG,BMP4,MDM2,CDH1,TP63,TP53,ERBB2,EGFR,NOTCH1,PAK1,BAX,AXL,TERT,NOS2,CTNNB1,HMGB1,E2F1,CASP8,MTOR,PTGS2,PTEN,TLR4,TP73,JAK2,SIRT3,SPP1,YWHAZ,NFE2L2,BCL2,IL6,TNF,CDKN2A,HIF1A,SLC16A1,VEGFA |
| GO:0031347 | Regulation of defense response | 24 | 674 | 1.78E-10 | HGF,SERPINE1,IFNG,SIRT2,STAT3,RB1,EGFR,PAK1,PRKDC,NOS2,HMGB1,STAT1,PTGS2,XIAP,TLR4,JAK2,HSPD1,XRCC5,GSTP1,ABCC1,IL6,TNF,BIRC2,MUC1 |
| GO:0008283 | Cell population proliferation | 21 | 493 | 1.84E-10 | ERCC1,MAPK1,HGF,CCND1,BMP4,STAT3,TP63,TP53,NOTCH1,BAX,CTNNB1,STAT1,MKI67,TLR4,IGFBP3,HSPD1,ERCC2,XRCC5,BCL2,GSTP1,TNF |
| GO:0006468 | Protein phosphorylation | 28 | 952 | 1.87E-10 | MAPK1,CSNK2A1,TGFB1,HGF,CCND1,BMP4,PIK3CA,IGF1R,ERBB2,EGFR,PAK1,AXL,BIRC5,PRKDC,MET,STK11,MTOR,TLR4,IGFBP3,JAK2,ERCC2,YWHAZ,BCL2,RUNX3,CDKN1A,TNF,PDK2,ZFP36 |
| GO:0048872 | Homeostasis of number of cells | 15 | 204 | 1.95E-10 | BMP4,EPAS1,RB1,NOTCH1,BAX,AXL,EZH2,HMGB1,FAS,JAK2,ERCC2,BCL2,IL6,HIF1A,VEGFA |
| GO:0007420 | Brain development | 25 | 745 | 2.04E-10 | BMP4,SIRT2,ALDH1A2,CDH1,XRCC1,TP53,EGFR,NOTCH1,PAK1,BAX,AXL,PRKDC,CXCR2,EZH2,SOX2,HSPA5,RARB,CTNNB1,E2F1,MTOR,PTEN,XRCC5,BCL2,H2AFX,HIF1A |
| GO:0002521 | Leukocyte differentiation | 18 | 338 | 2.13E-10 | LGALS1,IFNG,BMP4,STAT3,TP53,BAX,AXL,ADAM17,PRKDC,STK11,CTNNB1,HMGB1,CASP8,MTOR,BCL2,IL6,TNF,VEGFA |
| GO:0046649 | Lymphocyte activation | 19 | 390 | 2.28E-10 | ERCC1,LGALS1,MLH1,STAT3,TP53,BAX,AXL,ADAM17,PRKDC,STK11,CTNNB1,HMGB1,CASP8,MTOR,TLR4,HSPD1,BCL2,IL6,CD44 |
| GO:0070372 | Regulation of erk1 and erk2 cascade | 17 | 292 | 2.28E-10 | TGFB1,DUSP1,BMP4,NOX4,TIMP3,ERBB2,EGFR,NOTCH1,HMGB1,PTEN,TLR4,SIRT3,YWHAZ,GSTP1,CD44,TNF,VEGFA |
| GO:0071478 | Cellular response to radiation | 14 | 170 | 2.48E-10 | ERCC1,MDM2,NOX4,TP53,BAX,ERCC4,HSPA5,STK11,PTGS2,TP53BP1,RAD51,XRCC5,CDKN1A,H2AFX |
| GO:0045936 | Negative regulation of phosphate metabolic process | 22 | 561 | 2.50E-10 | TGFB1,HGF,IFNG,DUSP1,BMP4,SIRT2,STAT3,TIMP3,RB1,IGF1R,TP53,BAX,PRKDC,MTOR,PTEN,TLR4,IGFBP3,SIRT3,GSTP1,CDKN1A,TNF,CDKN2A |
| GO:0043405 | Regulation of map kinase activity | 18 | 342 | 2.53E-10 | MAPK1,TGFB1,HGF,DUSP1,BMP4,GDF15,NOX4,IGF1R,ERBB2,EGFR,PAK1,EZH2,TLR4,TP73,JAK2,GSTP1,TNF,VEGFA |
| GO:0042326 | Negative regulation of phosphorylation | 20 | 449 | 2.80E-10 | TGFB1,HGF,IFNG,DUSP1,BMP4,SIRT2,STAT3,TIMP3,RB1,IGF1R,BAX,PRKDC,MTOR,PTEN,TLR4,IGFBP3,SIRT3,GSTP1,CDKN1A,CDKN2A |
| GO:0009607 | Response to biotic stimulus | 32 | 1289 | 3.13E-10 | MAPK1,TGFB1,SERPINE1,IFNG,MLH1,SIRT2,TP53,NOTCH1,PAK1,AXL,ADAM17,PRKDC,HSPA5,NOS2,HMGB1,CASP8,STAT1,PTGS2,FASLG,ABCC2,UGT1A1,TLR4,CD274,JAK2,HSPD1,XRCC5,BCL2,GSTP1,IL6,CD44,TNF,ZFP36 |
| GO:0071902 | Positive regulation of protein serine/threonine kinase activity | 18 | 348 | 3.29E-10 | MAPK1,TGFB1,HGF,CCND1,IFNG,BMP4,GDF15,NOX4,ERBB2,EGFR,PAK1,ADAM17,EZH2,TLR4,TP73,JAK2,TNF,VEGFA |
| GO:0009617 | Response to bacterium | 23 | 634 | 3.51E-10 | MAPK1,TGFB1,SERPINE1,MLH1,SIRT2,NOTCH1,AXL,ADAM17,NOS2,HMGB1,CASP8,PTGS2,FASLG,ABCC2,UGT1A1,TLR4,CD274,JAK2,HSPD1,GSTP1,IL6,TNF,ZFP36 |
| GO:0006954 | Inflammatory response | 21 | 515 | 3.86E-10 | TGFB1,IFNG,NOX4,STAT3,EGFR,NOTCH1,AXL,OGG1,CXCR2,NOS2,HMGB1,PTGS2,UGT1A1,TLR4,JAK2,SPP1,NFE2L2,IL6,CD44,TNF,HIF1A |
| GO:0071453 | Cellular response to oxygen levels | 15 | 216 | 4.02E-10 | SIRT2,MDM2,EPAS1,TP53,NOTCH1,TERT,E2F1,FAS,MTOR,PTGS2,PTEN,NFE2L2,BCL2,HIF1A,VEGFA |
| GO:0010001 | Glial cell differentiation | 14 | 178 | 4.28E-10 | MAPK1,IFNG,SIRT2,STAT3,ERBB2,EGFR,NOTCH1,SOX2,CTNNB1,PTEN,TLR4,ERCC2,GSTP1,TNF |
| GO:0051276 | Chromosome organization | 29 | 1066 | 4.33E-10 | ERCC1,APEX1,MLH1,SIRT2,XRCC1,TP63,RB1,TP53,PAK1,TERT,ERCC4,PRKDC,EZH2,SOX2,MTA1,RAD51C,CTNNB1,HMGB1,BMI1,KDM4C,JAK2,RAD51,SIRT3,ERCC2,XRCC5,TOP2A,CDKN2A,H2AFX,XRCC3 |
| GO:1902041 | Regulation of extrinsic apoptotic signaling pathway via death domain receptors | 10 | 59 | 4.36E-10 | TNFRSF10A,HGF,SERPINE1,TNFSF10,TIMP3,FAS,CASP8,FASLG,PTEN,BRCA1 |
| GO:0006310 | DNA recombination | 15 | 219 | 4.78E-10 | ERCC1,APEX1,MLH1,XRCC1,ERCC4,PRKDC,RAD51C,HMGB1,RAD51,HSPD1,XRCC5,TOP2A,BRCA1,H2AFX,XRCC3 |
| GO:0009411 | Response to uv | 13 | 144 | 4.78E-10 | ERCC1,CCND1,MDM2,TP53,EGFR,BAX,ERCC4,STK11,ERCC5,PTGS2,ERCC2,BCL2,CDKN1A |
| GO:1901991 | Negative regulation of mitotic cell cycle phase transition | 15 | 219 | 4.78E-10 | CCND1,MDM2,RB1,TP53,BAX,BIRC5,PRKDC,EZH2,E2F1,PTEN,BCL2,CDKN1A,CDKN2A,BRCA1,MUC1 |
| GO:0006281 | DNA repair | 21 | 522 | 4.81E-10 | ERCC1,APEX1,MLH1,XRCC1,TP53,MGMT,OGG1,ERCC4,PRKDC,MTA1,RAD51C,HMGB1,ERCC5,TP73,TP53BP1,RAD51,ERCC2,XRCC5,BRCA1,H2AFX,XRCC3 |
| GO:2001235 | Positive regulation of apoptotic signaling pathway | 14 | 180 | 4.83E-10 | TNFSF10,TP63,TIMP3,TP53,BAX,E2F1,FAS,CASP8,MCL1,PTEN,TP73,JAK2,YWHAZ,BCL2 |
| GO:0070920 | Regulation of production of small rna involved in gene silencing by rna | 8 | 24 | 4.94E-10 | TGFB1,BMP4,STAT3,TP53,EGFR,TERT,IL6,TNF |
| GO:0001933 | Negative regulation of protein phosphorylation | 19 | 411 | 5.11E-10 | TGFB1,HGF,IFNG,DUSP1,BMP4,SIRT2,TIMP3,RB1,IGF1R,BAX,PRKDC,MTOR,PTEN,TLR4,IGFBP3,SIRT3,GSTP1,CDKN1A,CDKN2A |
| GO:0001775 | Cell activation | 29 | 1075 | 5.15E-10 | ERCC1,MAPK1,LGALS1,IFNG,MLH1,PIK3CA,STAT3,TP53,EGFR,BAX,AXL,ADAM17,PRKDC,CXCR2,STK11,CTNNB1,HMGB1,CASP8,MTOR,TLR4,JAK2,HSPD1,XRCC5,YWHAZ,BCL2,GSTP1,IL6,CD44,TNF |
| GO:0009611 | Response to wounding | 21 | 532 | 6.66E-10 | MAPK1,LGALS1,TGFB1,PIK3CA,ERBB2,EGFR,PAK1,BAX,AXL,ADAM17,SOX2,RAD51C,MTOR,JAK2,YWHAZ,BCL2,IL6,CD44,HIF1A,ZFP36,VEGFA |
| GO:2000106 | Regulation of leukocyte apoptotic process | 11 | 87 | 6.76E-10 | BMP4,TP53,BAX,AXL,ADAM17,CXCR2,PDCD1,PTEN,CD274,CDKN2A,HIF1A |
| GO:0009895 | Negative regulation of catabolic process | 17 | 316 | 6.90E-10 | CSNK2A1,HGF,SIRT2,PIK3CA,STAT3,TIMP3,TP53,EGFR,MET,NOS2,E2F1,MTOR,MCL1,BCL2,TNF,CDKN2A,ZFP36 |
| GO:1901360 | Organic cyclic compound metabolic process | 50 | 3118 | 7.17E-10 | ERCC1,MAPK1,APEX1,TGFB1,CCND1,MLH1,XRCC1,EPAS1,STAT3,TP53,NOTCH1,BAX,MGMT,OGG1,TERT,ERCC4,GSTM1,PRKDC,TYMS,EZH2,RARB,MTA1,RAD51C,HMGB1,E2F1,ERCC5,MTOR,PTGS2,ABCC2,PTEN,UGT1A1,AR,TP73,TP53BP1,RAD51,HSPD1,ERCC2,XRCC5,SPP1,NFE2L2,BCL2,ABCC1,CDKN1A,TOP2A,CDKN2A,BRCA1,H2AFX,XRCC3,ZFP36,CYP1B1 |
| GO:0010038 | Response to metal ion | 18 | 367 | 7.18E-10 | ERCC1,MAPK1,CCND1,DUSP1,MDM2,CDH1,EGFR,OGG1,TERT,HSPA5,BSG,CASP8,PTGS2,ABCC2,PTEN,NFE2L2,BCL2,HIF1A |
| GO:0048661 | Positive regulation of smooth muscle cell proliferation | 11 | 88 | 7.49E-10 | BMP4,MDM2,EGFR,PAK1,TERT,STAT1,MTOR,PTGS2,JAK2,IL6,TNF |
| GO:0030182 | Neuron differentiation | 28 | 1019 | 8.04E-10 | MAPK1,BMP4,ALDH1A2,CDH1,PIK3CA,STAT3,RB1,ERBB2,EGFR,NOTCH1,PAK1,USP9X,MET,HSPA5,STK11,BSG,CTNNB1,HMGB1,PTEN,POSTN,JAK2,ERCC2,YWHAZ,BCL2,RUNX3,IL6,HIF1A,VEGFA |
| GO:0072359 | Circulatory system development | 26 | 872 | 8.38E-10 | MAPK1,TGFB1,SERPINE1,BMP4,ALDH1A2,MDM2,NOX4,EPAS1,PIK3CA,RB1,TP53,ERBB2,NOTCH1,BAX,PRKDC,STK11,RARB,CTNNB1,MTOR,PTGS2,PTEN,YWHAZ,CDKN1A,HIF1A,CYP1B1,VEGFA |
| GO:0071456 | Cellular response to hypoxia | 14 | 189 | 8.59E-10 | SIRT2,MDM2,EPAS1,TP53,NOTCH1,TERT,E2F1,MTOR,PTGS2,PTEN,NFE2L2,BCL2,HIF1A,VEGFA |
| GO:0097305 | Response to alcohol | 15 | 233 | 1.04E-09 | TGFB1,CCND1,CDH1,STAT3,OGG1,TYMS,CTNNB1,CASP8,PTEN,UGT1A1,RAD51,GSTP1,CDKN1A,BRCA1,BIRC2 |
| GO:0045862 | Positive regulation of proteolysis | 18 | 377 | 1.08E-09 | TNFRSF10A,IFNG,TNFSF10,SIRT2,MDM2,STAT3,RB1,BAX,HMGB1,FAS,CASP8,FASLG,PTEN,JAK2,HSPD1,NFE2L2,TNF,CDKN2A |
| GO:0007093 | Mitotic cell cycle checkpoint | 13 | 156 | 1.16E-09 | TGFB1,CCND1,MDM2,RB1,TP53,BAX,BIRC5,PRKDC,E2F1,CDKN1A,TOP2A,BRCA1,MUC1 |
| GO:0071219 | Cellular response to molecule of bacterial origin | 14 | 195 | 1.25E-09 | MAPK1,TGFB1,SERPINE1,SIRT2,AXL,NOS2,HMGB1,ABCC2,TLR4,CD274,GSTP1,IL6,TNF,ZFP36 |
| GO:1905475 | Regulation of protein localization to membrane | 14 | 198 | 1.51E-09 | TGFB1,IFNG,TP63,TP53,ERBB2,EGFR,PAK1,E2F1,CASP8,AR,TP73,YWHAZ,BCL2,TNF |
| GO:0006952 | Defense response | 31 | 1296 | 1.64E-09 | TGFB1,SERPINE1,IFNG,SIRT2,NOX4,STAT3,TP53,EGFR,NOTCH1,AXL,OGG1,ADAM17,PRKDC,CXCR2,NOS2,HMGB1,STAT1,PTGS2,FASLG,UGT1A1,TLR4,JAK2,XRCC5,SPP1,NFE2L2,BCL2,IL6,CD44,TNF,EIF4E,HIF1A |
| GO:0048589 | Developmental growth | 18 | 389 | 1.73E-09 | ERCC1,BMP4,TP53,NOTCH1,TYMS,USP9X,EZH2,SOX2,STK11,RARB,CTNNB1,MTOR,PTEN,AR,POSTN,ERCC2,BCL2,CDKN1A |
| GO:1905477 | Positive regulation of protein localization to membrane | 12 | 129 | 2.06E-09 | IFNG,TP63,TP53,ERBB2,EGFR,PAK1,E2F1,CASP8,TP73,YWHAZ,BCL2,TNF |
| GO:0043434 | Response to peptide hormone | 18 | 394 | 2.10E-09 | APEX1,TNFSF10,MDM2,PIK3CA,STAT3,IGF1R,PAK1,PRKDC,BSG,STAT1,MTOR,PTGS2,ABCC2,PTEN,JAK2,NFE2L2,GSTP1,PDK2 |
| GO:0006302 | Double-strand break repair | 14 | 204 | 2.16E-09 | ERCC1,MLH1,XRCC1,TP53,ERCC4,PRKDC,MTA1,RAD51C,TP53BP1,RAD51,XRCC5,BRCA1,H2AFX,XRCC3 |
| GO:0006725 | Cellular aromatic compound metabolic process | 47 | 2882 | 2.19E-09 | ERCC1,MAPK1,APEX1,TGFB1,CCND1,MLH1,XRCC1,EPAS1,STAT3,TP53,NOTCH1,BAX,MGMT,OGG1,TERT,ERCC4,GSTM1,PRKDC,TYMS,EZH2,RARB,MTA1,RAD51C,HMGB1,E2F1,ERCC5,MTOR,PTGS2,PTEN,UGT1A1,AR,TP73,TP53BP1,RAD51,HSPD1,ERCC2,XRCC5,BCL2,ABCC1,CDKN1A,TOP2A,CDKN2A,BRCA1,H2AFX,XRCC3,ZFP36,CYP1B1 |
| GO:0043254 | Regulation of protein-containing complex assembly | 19 | 451 | 2.19E-09 | ERCC1,TGFB1,IFNG,SIRT2,RB1,TP53,PAK1,BAX,ERCC4,MET,HSPA5,HMGB1,ERCC5,MTOR,TLR4,ERCC2,TNF,BIRC2,VEGFA |
| GO:0045926 | Negative regulation of growth | 15 | 247 | 2.19E-09 | TGFB1,BMP4,GDF15,RB1,TP53,NOTCH1,PAK1,STK11,PTEN,TP73,SPP1,BCL2,CDKN1A,CDKN2A,HIF1A |
| GO:1901989 | Positive regulation of cell cycle phase transition | 11 | 99 | 2.27E-09 | APEX1,CCND1,MDM2,TP63,RB1,EGFR,BIRC5,TERT,ADAM17,EZH2,RAD51C |
| GO:0097191 | Extrinsic apoptotic signaling pathway | 11 | 100 | 2.50E-09 | TNFRSF10A,TGFB1,IFNG,BAX,FAS,CASP8,FASLG,MCL1,JAK2,BCL2,TNF |
| GO:0071480 | Cellular response to gamma radiation | 8 | 31 | 2.57E-09 | MDM2,NOX4,TP53,HSPA5,RAD51,XRCC5,CDKN1A,H2AFX |
| GO:0045321 | Leukocyte activation | 26 | 929 | 3.06E-09 | ERCC1,MAPK1,LGALS1,IFNG,MLH1,STAT3,TP53,BAX,AXL,ADAM17,PRKDC,CXCR2,STK11,CTNNB1,HMGB1,CASP8,MTOR,TLR4,JAK2,HSPD1,XRCC5,BCL2,GSTP1,IL6,CD44,TNF |
| GO:0050727 | Regulation of inflammatory response | 17 | 351 | 3.07E-09 | HGF,SERPINE1,IFNG,STAT3,RB1,EGFR,NOS2,PTGS2,XIAP,TLR4,JAK2,HSPD1,GSTP1,ABCC1,IL6,TNF,BIRC2 |
| GO:0031100 | Animal organ regeneration | 10 | 75 | 3.33E-09 | HGF,CCND1,EGFR,NOTCH1,AXL,TYMS,EZH2,UGT1A1,GSTP1,CDKN1A |
| GO:0043523 | Regulation of neuron apoptotic process | 14 | 212 | 3.40E-09 | PIK3CA,TP53,BAX,AXL,TERT,CTNNB1,FASLG,MCL1,JAK2,HSPD1,BCL2,TNF,HIF1A,SOD2 |
| GO:0051707 | Response to other organism | 30 | 1256 | 3.62E-09 | MAPK1,TGFB1,SERPINE1,IFNG,MLH1,SIRT2,TP53,NOTCH1,AXL,ADAM17,PRKDC,NOS2,HMGB1,CASP8,STAT1,PTGS2,FASLG,ABCC2,UGT1A1,TLR4,CD274,JAK2,HSPD1,XRCC5,BCL2,GSTP1,IL6,CD44,TNF,ZFP36 |
| GO:1904705 | Regulation of vascular associated smooth muscle cell proliferation | 9 | 52 | 3.65E-09 | MDM2,PAK1,TERT,PTEN,JAK2,GSTP1,CDKN1A,TNF,SOD2 |
| GO:0019538 | Protein metabolic process | 58 | 4251 | 4.22E-09 | MAPK1,LGALS1,CSNK2A1,TGFB1,HGF,CCND1,DUSP1,BMP4,SIRT2,MDM2,EPAS1,PIK3CA,IGF1R,TP53,ERBB2,EGFR,PAK1,AXL,BIRC5,ADAM17,PRKDC,USP9X,MET,EZH2,HSPA5,STK11,NOS2,MTA1,CTNNB1,CASP8,MTOR,XIAP,PTEN,TLR4,AR,BMI1,IGFBP3,KDM4C,JAK2,SIRT3,HSPD1,ERCC2,SPP1,YWHAZ,NFE2L2,BCL2,RUNX3,CDKN1A,IL6,TNF,CDKN2A,BRCA1,PDK2,EIF4E,HIF1A,ZFP36,BIRC2,MUC1 |
| GO:0033044 | Regulation of chromosome organization | 17 | 359 | 4.22E-09 | ERCC1,MAPK1,DUSP1,XRCC1,RB1,TP53,BIRC5,ERCC4,CTNNB1,MKI67,KDM4C,XRCC5,TOP2A,BRCA1,XRCC3,VEGFA,MUC1 |
| GO:0034641 | Cellular nitrogen compound metabolic process | 50 | 3282 | 4.24E-09 | ERCC1,MAPK1,APEX1,TGFB1,CCND1,MLH1,XRCC1,EPAS1,STAT3,TP53,EGFR,NOTCH1,BAX,MGMT,OGG1,TERT,ERCC4,GSTM1,PRKDC,TYMS,EZH2,NOS2,RARB,MTA1,RAD51C,HMGB1,E2F1,ERCC5,MTOR,PTGS2,ABCC2,PTEN,UGT1A1,AR,TP73,TP53BP1,RAD51,HSPD1,ERCC2,XRCC5,GSTP1,CDKN1A,TOP2A,CDKN2A,BRCA1,EIF4E,H2AFX,XRCC3,ZFP36,CYP1B1 |
| GO:0030336 | Negative regulation of cell migration | 15 | 262 | 4.59E-09 | APEX1,TGFB1,SERPINE1,DUSP1,CDH1,STAT3,NOTCH1,HMGB1,PTEN,IGFBP3,NFE2L2,BCL2,GSTP1,TNF,CYP1B1 |
| GO:0046483 | Heterocycle metabolic process | 46 | 2840 | 4.75E-09 | ERCC1,MAPK1,APEX1,TGFB1,CCND1,MLH1,XRCC1,EPAS1,STAT3,TP53,NOTCH1,BAX,MGMT,OGG1,TERT,ERCC4,PRKDC,TYMS,EZH2,RARB,MTA1,RAD51C,HMGB1,E2F1,ERCC5,MTOR,PTGS2,ABCC2,PTEN,UGT1A1,AR,TP73,TP53BP1,RAD51,HSPD1,ERCC2,XRCC5,NFE2L2,ABCC1,CDKN1A,TOP2A,CDKN2A,BRCA1,H2AFX,XRCC3,ZFP36 |
| GO:0007050 | Cell cycle arrest | 12 | 141 | 5.07E-09 | TGFB1,IFNG,DUSP1,RB1,TP53,NOTCH1,SOX2,STK11,MTOR,TP73,CDKN1A,CDKN2A |
| GO:0043406 | Positive regulation of map kinase activity | 15 | 265 | 5.30E-09 | MAPK1,TGFB1,HGF,BMP4,GDF15,NOX4,ERBB2,EGFR,PAK1,EZH2,TLR4,TP73,JAK2,TNF,VEGFA |
| GO:0048609 | Multicellular organismal reproductive process | 24 | 807 | 5.36E-09 | ERCC1,MAPK1,MLH1,BMP4,TP63,EGFR,NOTCH1,BAX,AXL,USP9X,HSPA5,STK11,BSG,RAD51C,CTNNB1,E2F1,MTOR,PTGS2,PTEN,AR,SPP1,BCL2,TOP2A,H2AFX |
| GO:0048646 | Anatomical structure formation involved in morphogenesis | 25 | 883 | 5.71E-09 | ERCC1,MAPK1,TGFB1,SERPINE1,DUSP1,BMP4,ALDH1A2,EPAS1,PIK3CA,TP63,TP53,NOTCH1,ADAM17,PRKDC,SOX2,CTNNB1,PTGS2,PTEN,AR,KDM4C,ERCC2,YWHAZ,HIF1A,CYP1B1,VEGFA |
| GO:0034612 | Response to tumor necrosis factor | 15 | 269 | 6.41E-09 | MAPK1,TP53,ADAM17,FAS,CASP8,STAT1,PTGS2,ABCC2,POSTN,JAK2,NFE2L2,TNF,BRCA1,ZFP36,BIRC2 |
| GO:1900407 | Regulation of cellular response to oxidative stress | 10 | 81 | 6.41E-09 | HGF,MET,CTNNB1,MCL1,TLR4,SIRT3,NFE2L2,TNF,HIF1A,SOD2 |
| GO:0048511 | Rhythmic process | 15 | 271 | 7.02E-09 | CSNK2A1,SERPINE1,TP53,EGFR,AXL,PRKDC,TYMS,USP9X,EZH2,HSPA5,NOS2,MTA1,MTOR,PTEN,TOP2A |
| GO:0051896 | Regulation of protein kinase b signaling | 14 | 226 | 7.21E-09 | TGFB1,HGF,GDF15,NOX4,PIK3CA,IGF1R,ERBB2,EGFR,AXL,MET,STK11,MTOR,PTEN,TNF |
| GO:0033138 | Positive regulation of peptidyl-serine phosphorylation | 11 | 112 | 7.25E-09 | TGFB1,IFNG,PIK3CA,EGFR,PAK1,PTGS2,BCL2,IL6,CD44,TNF,VEGFA |
| GO:0010632 | Regulation of epithelial cell migration | 14 | 227 | 7.58E-09 | TGFB1,IFNG,BMP4,NOTCH1,ADAM17,MET,HMGB1,MTOR,PTGS2,PTEN,NFE2L2,TNF,HIF1A,VEGFA |
| GO:0071222 | Cellular response to lipopolysaccharide | 13 | 185 | 7.60E-09 | MAPK1,TGFB1,SERPINE1,AXL,NOS2,HMGB1,ABCC2,TLR4,CD274,GSTP1,IL6,TNF,ZFP36 |
| GO:1901030 | Positive regulation of mitochondrial outer membrane permeabilization involved in apoptotic signaling pathway | 8 | 37 | 8.20E-09 | TP63,TP53,BAX,E2F1,CASP8,TP73,YWHAZ,BCL2 |
| GO:0033273 | Response to vitamin | 10 | 84 | 8.67E-09 | CCND1,ALDH1A2,MDM2,EGFR,OGG1,TYMS,PTGS2,POSTN,SPP1,GSTP1 |
| GO:0071383 | Cellular response to steroid hormone stimulus | 13 | 188 | 9.08E-09 | BMP4,RB1,EGFR,RARB,CTNNB1,ABCC2,UGT1A1,AR,JAK2,GSTP1,BRCA1,EIF4E,ZFP36 |
| GO:0043154 | Negative regulation of cysteine-type endopeptidase activity involved in apoptotic process | 10 | 85 | 9.59E-09 | CSNK2A1,HGF,MDM2,BIRC5,PTGS2,XIAP,CD44,TNF,BIRC2,VEGFA |
| GO:1904018 | Positive regulation of vasculature development | 13 | 189 | 9.60E-09 | HGF,SERPINE1,STAT3,NOTCH1,TERT,CXCR2,PTGS2,NFE2L2,BRCA1,HIF1A,SOD2,CYP1B1,VEGFA |
| GO:0030856 | Regulation of epithelial cell differentiation | 12 | 151 | 1.01E-08 | SERPINE1,CCND1,IFNG,BMP4,TP63,NOTCH1,EZH2,CTNNB1,STAT1,TNF,ZFP36,VEGFA |
| GO:0031570 | DNA integrity checkpoint | 12 | 151 | 1.01E-08 | CCND1,MDM2,TP53,BAX,PRKDC,E2F1,TP53BP1,CDKN1A,TOP2A,BRCA1,H2AFX,MUC1 |
| GO:0090559 | Regulation of membrane permeability | 10 | 86 | 1.05E-08 | STAT3,TP63,TP53,BAX,E2F1,CASP8,MTOR,TP73,YWHAZ,BCL2 |
| GO:1901653 | Cellular response to peptide | 16 | 331 | 1.09E-08 | APEX1,MDM2,PIK3CA,STAT3,IGF1R,TP53,PAK1,PRKDC,STAT1,PTEN,TLR4,JAK2,NFE2L2,GSTP1,ABCC1,PDK2 |
| GO:1902042 | Negative regulation of extrinsic apoptotic signaling pathway via death domain receptors | 8 | 39 | 1.16E-08 | TNFRSF10A,HGF,SERPINE1,TNFSF10,FAS,CASP8,FASLG,BRCA1 |
| GO:0048638 | Regulation of developmental growth | 16 | 333 | 1.18E-08 | MAPK1,BMP4,GDF15,PIK3CA,STAT3,NOTCH1,PAK1,PRKDC,MTOR,PTEN,AR,TP73,SPP1,BCL2,CDKN1A,VEGFA |
| GO:0014013 | Regulation of gliogenesis | 11 | 119 | 1.27E-08 | TGFB1,SIRT2,NOTCH1,TERT,EZH2,CTNNB1,E2F1,MTOR,TP73,IL6,TNF |
| GO:0042542 | Response to hydrogen peroxide | 11 | 119 | 1.27E-08 | APEX1,DUSP1,MDM2,AXL,EZH2,STAT1,HSPD1,NFE2L2,BCL2,IL6,CYP1B1 |
| GO:1902105 | Regulation of leukocyte differentiation | 15 | 285 | 1.30E-08 | TGFB1,IFNG,BMP4,RB1,ERBB2,AXL,PRKDC,CTNNB1,HMGB1,CASP8,MTOR,TLR4,RUNX3,TNF,CDKN2A |
| GO:1903798 | Regulation of production of mirnas involved in gene silencing by mirna | 7 | 23 | 1.40E-08 | TGFB1,BMP4,STAT3,TP53,EGFR,IL6,TNF |
| GO:0050731 | Positive regulation of peptidyl-tyrosine phosphorylation | 13 | 196 | 1.42E-08 | TGFB1,HGF,IFNG,NOX4,STAT3,TP53,ADAM17,MTOR,JAK2,IL6,CD44,TNF,VEGFA |
| GO:0050768 | Negative regulation of neurogenesis | 15 | 287 | 1.42E-08 | LGALS1,SIRT2,MDM2,STAT3,TP53,NOTCH1,TERT,SOX2,CTNNB1,PTEN,TP73,SPP1,IL6,TNF,EIF4E |
| GO:0002683 | Negative regulation of immune system process | 18 | 450 | 1.45E-08 | TGFB1,DUSP1,BMP4,ERBB2,NOTCH1,AXL,PRKDC,CXCR2,PDCD1,CTNNB1,HMGB1,TLR4,CD274,NFE2L2,RUNX3,TNF,CDKN2A,ZFP36 |
| GO:0006919 | Activation of cysteine-type endopeptidase activity involved in apoptotic process | 10 | 90 | 1.54E-08 | TNFRSF10A,TNFSF10,BAX,FAS,CASP8,FASLG,JAK2,HSPD1,TNF,CDKN2A |
| GO:0042110 | T cell activation | 14 | 243 | 1.67E-08 | STAT3,TP53,BAX,ADAM17,PRKDC,STK11,CTNNB1,HMGB1,CASP8,MTOR,HSPD1,BCL2,IL6,CD44 |
| GO:0072331 | Signal transduction by p53 class mediator | 11 | 123 | 1.72E-08 | MDM2,TP63,TP53,BAX,STK11,E2F1,TP73,CDKN1A,BRCA1,PDK2,MUC1 |
| GO:2000637 | Positive regulation of gene silencing by mirna | 7 | 24 | 1.77E-08 | TGFB1,BMP4,STAT3,TP53,EGFR,IL6,ZFP36 |
| GO:0016310 | Phosphorylation | 29 | 1275 | 2.18E-08 | MAPK1,CSNK2A1,TGFB1,HGF,CCND1,BMP4,PIK3CA,STAT3,IGF1R,ERBB2,EGFR,PAK1,AXL,BIRC5,PRKDC,MET,STK11,MTOR,TLR4,IGFBP3,JAK2,ERCC2,YWHAZ,BCL2,RUNX3,CDKN1A,TNF,PDK2,ZFP36 |
| GO:0031334 | Positive regulation of protein-containing complex assembly | 14 | 249 | 2.23E-08 | ERCC1,TGFB1,IFNG,TP53,PAK1,BAX,ERCC4,MET,ERCC5,MTOR,TLR4,ERCC2,TNF,VEGFA |
| GO:0035239 | Tube morphogenesis | 21 | 656 | 2.23E-08 | TGFB1,SERPINE1,BMP4,EPAS1,PIK3CA,TP63,EGFR,NOTCH1,PAK1,BAX,MET,CXCR2,CTNNB1,PTGS2,PTEN,AR,YWHAZ,BCL2,HIF1A,CYP1B1,VEGFA |
| GO:0000722 | Telomere maintenance via recombination | 6 | 12 | 2.26E-08 | ERCC1,XRCC1,ERCC4,RAD51C,RAD51,XRCC3 |
| GO:0048871 | Multicellular organismal homeostasis | 16 | 352 | 2.43E-08 | NOX4,EPAS1,PIK3CA,STAT3,TP63,RB1,NOTCH1,BAX,MET,STK11,CTNNB1,PTGS2,TLR4,BCL2,IL6,VEGFA |
| GO:0071479 | Cellular response to ionizing radiation | 9 | 67 | 2.47E-08 | MDM2,NOX4,TP53,HSPA5,TP53BP1,RAD51,XRCC5,CDKN1A,H2AFX |
| GO:1901216 | Positive regulation of neuron death | 10 | 98 | 3.22E-08 | IFNG,TP53,BAX,CTNNB1,CASP8,MTOR,FASLG,MCL1,TLR4,TNF |
| GO:0031330 | Negative regulation of cellular catabolic process | 14 | 258 | 3.39E-08 | CSNK2A1,HGF,SIRT2,PIK3CA,STAT3,TIMP3,TP53,MET,E2F1,MTOR,MCL1,BCL2,CDKN2A,ZFP36 |
| GO:0050730 | Regulation of peptidyl-tyrosine phosphorylation | 14 | 258 | 3.39E-08 | TGFB1,HGF,IFNG,NOX4,STAT3,TP53,EGFR,ADAM17,MTOR,JAK2,IL6,CD44,TNF,VEGFA |
| GO:0051897 | Positive regulation of protein kinase b signaling | 12 | 170 | 3.39E-08 | TGFB1,HGF,GDF15,NOX4,PIK3CA,IGF1R,ERBB2,EGFR,AXL,MET,MTOR,TNF |
| GO:1900740 | Positive regulation of protein insertion into mitochondrial membrane involved in apoptotic signaling pathway | 7 | 27 | 3.46E-08 | TP63,TP53,E2F1,CASP8,TP73,YWHAZ,BCL2 |
| GO:0044087 | Regulation of cellular component biogenesis | 25 | 971 | 3.47E-08 | ERCC1,TGFB1,IFNG,SIRT2,NOX4,RB1,TP53,NOTCH1,PAK1,BAX,ERCC4,MET,HSPA5,HMGB1,ERCC5,MTOR,PTEN,TLR4,SIRT3,ERCC2,XRCC5,TNF,BRCA1,BIRC2,VEGFA |
| GO:0051402 | Neuron apoptotic process | 8 | 46 | 3.47E-08 | TP63,RB1,TP53,BAX,HSPA5,FAS,XIAP,BCL2 |
| GO:0016202 | Regulation of striated muscle tissue development | 11 | 133 | 3.57E-08 | MAPK1,TGFB1,BMP4,SIRT2,NOTCH1,PAK1,CTNNB1,MTOR,PTEN,TP73,BCL2 |
| GO:0034614 | Cellular response to reactive oxygen species | 11 | 133 | 3.57E-08 | MAPK1,APEX1,MDM2,EGFR,AXL,EZH2,NFE2L2,IL6,PDK2,SOD2,CYP1B1 |
| GO:0016477 | Cell migration | 24 | 896 | 3.66E-08 | TNFRSF10A,TGFB1,HGF,PIK3CA,EGFR,NOTCH1,PAK1,BAX,AXL,ADAM17,USP9X,CXCR2,BSG,CTNNB1,HMGB1,PTEN,ABCC1,IL6,CD44,TNF,HIF1A,SLC16A1,CYP1B1,VEGFA |
| GO:0050776 | Regulation of immune response | 24 | 896 | 3.66E-08 | MAPK1,TGFB1,IFNG,MLH1,PIK3CA,PAK1,BAX,PRKDC,STK11,PDCD1,HMGB1,STAT1,XIAP,TLR4,CD274,JAK2,TP53BP1,HSPD1,XRCC5,BCL2,IL6,TNF,BIRC2,MUC1 |
| GO:0030857 | Negative regulation of epithelial cell differentiation | 8 | 47 | 3.97E-08 | CCND1,IFNG,TP63,NOTCH1,EZH2,CTNNB1,STAT1,VEGFA |
| GO:0031349 | Positive regulation of defense response | 16 | 367 | 4.16E-08 | SERPINE1,IFNG,STAT3,EGFR,PAK1,PRKDC,HMGB1,PTGS2,TLR4,JAK2,HSPD1,XRCC5,ABCC1,IL6,TNF,MUC1 |
| GO:0097300 | Programmed necrotic cell death | 7 | 28 | 4.17E-08 | TP53,BAX,FAS,FASLG,TLR4,TNF,BIRC2 |
| GO:0006955 | Immune response | 32 | 1588 | 4.25E-08 | ERCC1,MAPK1,LGALS1,IFNG,MLH1,TNFSF10,SIRT2,STAT3,IGF1R,TP53,NOTCH1,AXL,ADAM17,PRKDC,CXCR2,NOS2,PDCD1,HMGB1,FAS,STAT1,MTOR,FASLG,TLR4,CD274,JAK2,HSPD1,XRCC5,BCL2,GSTP1,IL6,CD44,TNF |
| GO:0090304 | Nucleic acid metabolic process | 38 | 2178 | 4.29E-08 | ERCC1,APEX1,CCND1,MLH1,XRCC1,EPAS1,STAT3,TP53,NOTCH1,BAX,MGMT,OGG1,TERT,ERCC4,PRKDC,TYMS,EZH2,RARB,MTA1,RAD51C,HMGB1,E2F1,ERCC5,PTEN,AR,TP73,TP53BP1,RAD51,HSPD1,ERCC2,XRCC5,CDKN1A,TOP2A,CDKN2A,BRCA1,H2AFX,XRCC3,ZFP36 |
| GO:0043280 | Positive regulation of cysteine-type endopeptidase activity involved in apoptotic process | 11 | 136 | 4.33E-08 | TNFRSF10A,TNFSF10,BAX,HMGB1,FAS,CASP8,FASLG,JAK2,HSPD1,TNF,CDKN2A |
| GO:0060759 | Regulation of response to cytokine stimulus | 12 | 175 | 4.41E-08 | IFNG,AXL,ADAM17,CASP8,STAT1,TLR4,JAK2,GSTP1,IL6,TNF,HIF1A,BIRC2 |
| GO:1903037 | Regulation of leukocyte cell-cell adhesion | 15 | 315 | 4.41E-08 | LGALS1,IFNG,BMP4,PIK3CA,ERBB2,PAK1,PDCD1,HMGB1,CD274,HSPD1,RUNX3,IL6,CD44,TNF,CDKN2A |
| GO:1902808 | Positive regulation of cell cycle g1/s phase transition | 8 | 48 | 4.50E-08 | APEX1,CCND1,MDM2,TP63,EGFR,TERT,ADAM17,EZH2 |
| GO:0010950 | Positive regulation of endopeptidase activity | 12 | 176 | 4.65E-08 | TNFRSF10A,TNFSF10,STAT3,BAX,HMGB1,FAS,CASP8,FASLG,JAK2,HSPD1,TNF,CDKN2A |
| GO:0006303 | Double-strand break repair via nonhomologous end joining | 9 | 74 | 5.16E-08 | ERCC1,MLH1,XRCC1,ERCC4,PRKDC,TP53BP1,XRCC5,BRCA1,H2AFX |
| GO:0000077 | DNA damage checkpoint | 11 | 140 | 5.66E-08 | CCND1,MDM2,TP53,BAX,PRKDC,E2F1,TP53BP1,CDKN1A,BRCA1,H2AFX,MUC1 |
| GO:0032147 | Activation of protein kinase activity | 16 | 376 | 5.66E-08 | MAPK1,TNFRSF10A,HGF,BMP4,GDF15,PIK3CA,EGFR,PAK1,STK11,MTOR,TLR4,TP73,JAK2,CDKN1A,TNF,VEGFA |
| GO:0046683 | Response to organophosphorus | 11 | 140 | 5.66E-08 | APEX1,DUSP1,NOX4,TYMS,HSPA5,BSG,STAT1,PTGS2,PTEN,HSPD1,BIRC2 |
| GO:0046902 | Regulation of mitochondrial membrane permeability | 9 | 75 | 5.70E-08 | STAT3,TP63,TP53,BAX,E2F1,CASP8,TP73,YWHAZ,BCL2 |
| GO:0040011 | Locomotion | 28 | 1251 | 5.96E-08 | MAPK1,TNFRSF10A,TGFB1,HGF,BMP4,PIK3CA,ERBB2,EGFR,NOTCH1,PAK1,BAX,AXL,ADAM17,USP9X,MET,CXCR2,BSG,CTNNB1,HMGB1,PTEN,ABCC1,IL6,CD44,TNF,HIF1A,SLC16A1,CYP1B1,VEGFA |
| GO:0009790 | Embryo development | 25 | 1002 | 6.12E-08 | ERCC1,MAPK1,TGFB1,DUSP1,BMP4,ALDH1A2,EPAS1,TP63,TP53,EGFR,NOTCH1,BAX,PRKDC,SOX2,RARB,CTNNB1,CASP8,AR,KDM4C,ERCC2,TNF,TOP2A,BRCA1,HIF1A,VEGFA |
| GO:0072332 | Intrinsic apoptotic signaling pathway by p53 class mediator | 8 | 51 | 6.77E-08 | TP63,TP53,BAX,STK11,E2F1,TP73,CDKN1A,PDK2 |
| GO:0006996 | Organelle organization | 49 | 3450 | 6.83E-08 | ERCC1,APEX1,MLH1,SIRT2,XRCC1,EPAS1,STAT3,TP63,RB1,TP53,NOTCH1,PAK1,BAX,BIRC5,TERT,ERCC4,PRKDC,USP9X,EZH2,SOX2,NOS2,MTA1,RAD51C,CTNNB1,HMGB1,E2F1,CASP8,MTOR,FASLG,PTEN,BMI1,TP73,KDM4C,JAK2,RAD51,SIRT3,HSPD1,ERCC2,XRCC5,YWHAZ,BCL2,TNF,TOP2A,CDKN2A,BRCA1,H2AFX,SLC16A1,SOD2,XRCC3 |
| GO:0002262 | Myeloid cell homeostasis | 10 | 108 | 7.10E-08 | BMP4,EPAS1,RB1,BAX,AXL,HMGB1,JAK2,ERCC2,IL6,VEGFA |
| GO:0050729 | Positive regulation of inflammatory response | 11 | 144 | 7.32E-08 | SERPINE1,IFNG,STAT3,EGFR,PTGS2,TLR4,JAK2,HSPD1,ABCC1,IL6,TNF |
| GO:0010165 | Response to x-ray | 7 | 31 | 7.37E-08 | ERCC1,CCND1,TP53,TP53BP1,RAD51,XRCC5,CDKN1A |
| GO:0036003 | Positive regulation of transcription from rna polymerase ii promoter in response to stress | 7 | 31 | 7.37E-08 | TP53,NOTCH1,HSPA5,NFE2L2,HIF1A,VEGFA,MUC1 |
| GO:0006139 | Nucleobase-containing compound metabolic process | 42 | 2659 | 7.83E-08 | ERCC1,MAPK1,APEX1,TGFB1,CCND1,MLH1,XRCC1,EPAS1,STAT3,TP53,NOTCH1,BAX,MGMT,OGG1,TERT,ERCC4,PRKDC,TYMS,EZH2,RARB,MTA1,RAD51C,HMGB1,E2F1,ERCC5,MTOR,PTGS2,PTEN,AR,TP73,TP53BP1,RAD51,HSPD1,ERCC2,XRCC5,CDKN1A,TOP2A,CDKN2A,BRCA1,H2AFX,XRCC3,ZFP36 |
| GO:0030900 | Forebrain development | 16 | 387 | 8.17E-08 | BMP4,ALDH1A2,CDH1,XRCC1,EGFR,NOTCH1,BAX,AXL,EZH2,SOX2,RARB,CTNNB1,E2F1,PTEN,H2AFX,HIF1A |
| GO:0071156 | Regulation of cell cycle arrest | 10 | 110 | 8.27E-08 | CCND1,MDM2,TP53,BAX,E2F1,TP73,CDKN1A,CDKN2A,BRCA1,MUC1 |
| GO:0010634 | Positive regulation of epithelial cell migration | 11 | 147 | 8.85E-08 | TGFB1,IFNG,BMP4,ADAM17,MET,HMGB1,MTOR,PTGS2,NFE2L2,HIF1A,VEGFA |
| GO:0061419 | Positive regulation of transcription from rna polymerase ii promoter in response to hypoxia | 5 | 6 | 8.94E-08 | TP53,NOTCH1,NFE2L2,HIF1A,VEGFA |
| GO:0044403 | Symbiotic process | 23 | 865 | 9.06E-08 | MAPK1,MDM2,CDH1,STAT3,RB1,TP53,EGFR,BAX,AXL,MET,CTNNB1,HMGB1,E2F1,CASP8,STAT1,TP73,HSPD1,ERCC2,XRCC5,NFE2L2,EIF4E,H2AFX,ZFP36 |
| GO:0060429 | Epithelium development | 26 | 1109 | 9.56E-08 | MAPK1,TGFB1,CCND1,BMP4,ALDH1A2,TP63,TP53,EGFR,NOTCH1,PAK1,ADAM17,PRKDC,TYMS,MET,CXCR2,RARB,CTNNB1,STAT1,AR,JAK2,ERCC2,BCL2,CDKN1A,CD44,HIF1A,VEGFA |
| GO:0000723 | Telomere maintenance | 10 | 112 | 9.63E-08 | ERCC1,APEX1,XRCC1,TERT,ERCC4,PRKDC,RAD51C,RAD51,XRCC5,XRCC3 |
| GO:0002285 | Lymphocyte activation involved in immune response | 10 | 112 | 9.63E-08 | ERCC1,LGALS1,MLH1,STAT3,TP53,HMGB1,MTOR,TLR4,HSPD1,IL6 |
| GO:0007167 | Enzyme linked receptor protein signaling pathway | 21 | 720 | 9.91E-08 | MAPK1,TGFB1,HGF,BMP4,SIRT2,GDF15,PIK3CA,STAT3,IGF1R,TP53,ERBB2,EGFR,PAK1,AXL,ADAM17,USP9X,MET,PTEN,JAK2,PDK2,VEGFA |
| GO:0031668 | Cellular response to extracellular stimulus | 13 | 236 | 1.04E-07 | MAPK1,MDM2,TP53,AXL,HSPA5,FAS,MTOR,POSTN,NFE2L2,BCL2,GSTP1,CDKN1A,PDK2 |
| GO:0007169 | Transmembrane receptor protein tyrosine kinase signaling pathway | 18 | 518 | 1.07E-07 | MAPK1,TGFB1,HGF,SIRT2,GDF15,PIK3CA,STAT3,IGF1R,ERBB2,EGFR,PAK1,AXL,ADAM17,MET,PTEN,JAK2,PDK2,VEGFA |
| GO:0010507 | Negative regulation of autophagy | 9 | 82 | 1.11E-07 | HGF,SIRT2,PIK3CA,STAT3,TP53,MET,MTOR,MCL1,BCL2 |
| GO:1900182 | Positive regulation of protein localization to nucleus | 9 | 82 | 1.11E-07 | MAPK1,TGFB1,IFNG,CDH1,TERT,STK11,PTGS2,JAK2,CDKN2A |
| GO:1901992 | Positive regulation of mitotic cell cycle phase transition | 9 | 82 | 1.11E-07 | APEX1,CCND1,MDM2,RB1,EGFR,BIRC5,TERT,ADAM17,RAD51C |
| GO:2000278 | Regulation of dna biosynthetic process | 10 | 114 | 1.11E-07 | MAPK1,HGF,DUSP1,NOX4,TP53,ERCC4,CTNNB1,XRCC5,CDKN1A,VEGFA |
| GO:0000902 | Cell morphogenesis | 21 | 726 | 1.12E-07 | MAPK1,HGF,CDH1,NOX4,PIK3CA,RB1,ERBB2,EGFR,NOTCH1,PAK1,AXL,USP9X,MET,STK11,BSG,CTNNB1,PTEN,AR,POSTN,BCL2,VEGFA |
| GO:0001944 | Vasculature development | 18 | 522 | 1.19E-07 | MAPK1,TGFB1,SERPINE1,BMP4,ALDH1A2,MDM2,EPAS1,PIK3CA,NOTCH1,BAX,STK11,CTNNB1,PTGS2,PTEN,YWHAZ,HIF1A,CYP1B1,VEGFA |
| GO:0007507 | Heart development | 18 | 522 | 1.19E-07 | MAPK1,TGFB1,BMP4,ALDH1A2,MDM2,NOX4,RB1,TP53,ERBB2,NOTCH1,PRKDC,RARB,CTNNB1,MTOR,PTEN,CDKN1A,HIF1A,VEGFA |
| GO:0046822 | Regulation of nucleocytoplasmic transport | 10 | 115 | 1.19E-07 | MAPK1,TGFB1,IFNG,BMP4,MDM2,CDH1,TP53,PTGS2,JAK2,CDKN2A |
| GO:0048762 | Mesenchymal cell differentiation | 11 | 152 | 1.19E-07 | MAPK1,TGFB1,HGF,BMP4,ALDH1A2,NOTCH1,CTNNB1,STAT1,S100A4,BCL2,HIF1A |
| GO:0048145 | Regulation of fibroblast proliferation | 9 | 83 | 1.21E-07 | TP53,EGFR,BAX,PRKDC,CTNNB1,E2F1,BMI1,GSTP1,CDKN1A |
| GO:0048878 | Chemical homeostasis | 26 | 1124 | 1.21E-07 | MAPK1,LGALS1,NOX4,EPAS1,PIK3CA,STAT3,TP63,IGF1R,EGFR,BAX,MET,CXCR2,STK11,HMGB1,STAT1,FASLG,ABCC2,XIAP,JAK2,BCL2,GSTP1,PDK2,HIF1A,SLC16A1,SOD2,VEGFA |
| GO:0032642 | Regulation of chemokine production | 9 | 84 | 1.32E-07 | IFNG,ADAM17,HMGB1,TLR4,POSTN,GSTP1,IL6,TNF,HIF1A |
| GO:0071158 | Positive regulation of cell cycle arrest | 9 | 84 | 1.32E-07 | MDM2,TP53,BAX,E2F1,TP73,CDKN1A,CDKN2A,BRCA1,MUC1 |
| GO:2001021 | Negative regulation of response to dna damage stimulus | 9 | 84 | 1.32E-07 | ERCC1,MDM2,XRCC1,OGG1,ERCC4,TP53BP1,BCL2,CD44,MUC1 |
| GO:0010821 | Regulation of mitochondrion organization | 12 | 196 | 1.33E-07 | HGF,TNFSF10,TP63,TP53,BAX,E2F1,CASP8,TP73,YWHAZ,BCL2,CDKN2A,HIF1A |
| GO:0030098 | Lymphocyte differentiation | 13 | 242 | 1.33E-07 | LGALS1,STAT3,TP53,BAX,AXL,ADAM17,PRKDC,STK11,CTNNB1,HMGB1,MTOR,BCL2,IL6 |
| GO:0051147 | Regulation of muscle cell differentiation | 11 | 154 | 1.33E-07 | TGFB1,BMP4,GDF15,MDM2,NOTCH1,PAK1,EZH2,CTNNB1,MTOR,BCL2,SOD2 |
| GO:2000378 | Negative regulation of reactive oxygen species metabolic process | 8 | 57 | 1.38E-07 | SIRT2,STAT3,TP53,SIRT3,HSPD1,BCL2,BRCA1,HIF1A |
| GO:0045664 | Regulation of neuron differentiation | 20 | 665 | 1.41E-07 | LGALS1,HGF,BMP4,MDM2,NOTCH1,PAK1,EZH2,SOX2,HSPA5,STK11,RARB,MTOR,PTEN,TP73,KDM4C,SPP1,NFE2L2,BCL2,EIF4E,VEGFA |
| GO:0044703 | Multi-organism reproductive process | 24 | 971 | 1.49E-07 | ERCC1,MAPK1,MLH1,BMP4,STAT3,TP63,NOTCH1,BAX,AXL,USP9X,STK11,BSG,RAD51C,CTNNB1,E2F1,MTOR,PTGS2,ABCC2,PTEN,AR,SPP1,BCL2,TOP2A,H2AFX |
| GO:0045931 | Positive regulation of mitotic cell cycle | 11 | 156 | 1.49E-07 | APEX1,CCND1,MDM2,RB1,EGFR,BIRC5,TERT,ADAM17,RAD51C,EIF4E,XRCC3 |
| GO:0071356 | Cellular response to tumor necrosis factor | 13 | 245 | 1.51E-07 | MAPK1,TP53,ADAM17,FAS,STAT1,ABCC2,POSTN,JAK2,NFE2L2,TNF,BRCA1,ZFP36,BIRC2 |
| GO:1903708 | Positive regulation of hemopoiesis | 12 | 199 | 1.55E-07 | TGFB1,IFNG,STAT3,RB1,AXL,PRKDC,HMGB1,CASP8,STAT1,RUNX3,TNF,HIF1A |
| GO:1904035 | Regulation of epithelial cell apoptotic process | 9 | 87 | 1.71E-07 | SERPINE1,TERT,MTOR,FASLG,JAK2,NFE2L2,IL6,TNF,ZFP36 |
| GO:0043388 | Positive regulation of dna binding | 8 | 59 | 1.74E-07 | TGFB1,IFNG,SIRT2,RB1,CTNNB1,HMGB1,JAK2,ERCC2 |
| GO:0010822 | Positive regulation of mitochondrion organization | 10 | 121 | 1.80E-07 | TNFSF10,TP63,TP53,BAX,E2F1,CASP8,TP73,YWHAZ,BCL2,HIF1A |
| GO:0050778 | Positive regulation of immune response | 19 | 607 | 1.87E-07 | MAPK1,TGFB1,IFNG,MLH1,PIK3CA,PAK1,BAX,PRKDC,STK11,HMGB1,TLR4,CD274,TP53BP1,HSPD1,XRCC5,BCL2,IL6,TNF,MUC1 |
| GO:0042113 | B cell activation | 11 | 161 | 2.00E-07 | ERCC1,LGALS1,MLH1,TP53,BAX,ADAM17,PRKDC,CASP8,TLR4,HSPD1,BCL2 |
| GO:0006796 | Phosphate-containing compound metabolic process | 36 | 2107 | 2.06E-07 | MAPK1,APEX1,CSNK2A1,TGFB1,HGF,CCND1,DUSP1,BMP4,PIK3CA,STAT3,IGF1R,ERBB2,EGFR,PAK1,ALDH1A1,AXL,BIRC5,OGG1,PRKDC,TYMS,MET,STK11,MTOR,PTGS2,PTEN,TLR4,IGFBP3,JAK2,ERCC2,YWHAZ,BCL2,RUNX3,CDKN1A,TNF,PDK2,ZFP36 |
| GO:0032722 | Positive regulation of chemokine production | 8 | 61 | 2.19E-07 | IFNG,ADAM17,HMGB1,TLR4,POSTN,IL6,TNF,HIF1A |
| GO:0001959 | Regulation of cytokine-mediated signaling pathway | 11 | 163 | 2.23E-07 | IFNG,AXL,ADAM17,CASP8,STAT1,JAK2,GSTP1,IL6,TNF,HIF1A,BIRC2 |
| GO:0060149 | Negative regulation of posttranscriptional gene silencing | 6 | 20 | 2.23E-07 | TGFB1,STAT3,TP53,TERT,IL6,TNF |
| GO:0060967 | Negative regulation of gene silencing by rna | 6 | 20 | 2.23E-07 | TGFB1,STAT3,TP53,TERT,IL6,TNF |
| GO:0010594 | Regulation of endothelial cell migration | 11 | 164 | 2.37E-07 | TGFB1,BMP4,NOTCH1,ADAM17,MET,HMGB1,PTGS2,NFE2L2,TNF,HIF1A,VEGFA |
| GO:2001242 | Regulation of intrinsic apoptotic signaling pathway | 11 | 164 | 2.37E-07 | MDM2,TP53,BAX,PTGS2,MCL1,NFE2L2,BCL2,CD44,HIF1A,SOD2,MUC1 |
| GO:0001558 | Regulation of cell growth | 16 | 423 | 2.49E-07 | CSNK2A1,TGFB1,RB1,TP53,ERBB2,EGFR,PAK1,ADAM17,STK11,MTOR,IGFBP3,SPP1,BCL2,CDKN1A,CDKN2A,VEGFA |
| GO:0045785 | Positive regulation of cell adhesion | 16 | 423 | 2.49E-07 | LGALS1,IFNG,PIK3CA,ERBB2,PAK1,SOX2,PDCD1,HMGB1,CD274,JAK2,HSPD1,RUNX3,IL6,CD44,TNF,VEGFA |
| GO:0051053 | Negative regulation of dna metabolic process | 10 | 126 | 2.52E-07 | ERCC1,MLH1,DUSP1,XRCC1,TP53,OGG1,ERCC4,TP53BP1,XRCC5,CDKN1A |
| GO:0002360 | T cell lineage commitment | 6 | 21 | 2.81E-07 | STAT3,TP53,PRKDC,MTOR,BCL2,IL6 |
| GO:0043535 | Regulation of blood vessel endothelial cell migration | 9 | 93 | 2.84E-07 | TGFB1,NOTCH1,ADAM17,HMGB1,PTGS2,NFE2L2,TNF,HIF1A,VEGFA |
| GO:0045637 | Regulation of myeloid cell differentiation | 13 | 260 | 2.86E-07 | TGFB1,IFNG,STAT3,RB1,PRKDC,CTNNB1,CASP8,STAT1,MTOR,TLR4,TNF,HIF1A,ZFP36 |
| GO:0051101 | Regulation of dna binding | 10 | 128 | 2.88E-07 | TGFB1,IFNG,SIRT2,RB1,ERCC4,CTNNB1,HMGB1,E2F1,JAK2,ERCC2 |
| GO:0060485 | Mesenchyme development | 12 | 212 | 2.90E-07 | MAPK1,TGFB1,HGF,BMP4,ALDH1A2,MDM2,NOTCH1,CTNNB1,STAT1,S100A4,BCL2,HIF1A |
| GO:0001889 | Liver development | 10 | 129 | 3.08E-07 | HGF,CCND1,ALDH1A2,PIK3CA,EGFR,NOTCH1,TYMS,MET,EZH2,UGT1A1 |
| GO:0006282 | Regulation of dna repair | 10 | 129 | 3.08E-07 | XRCC1,EGFR,MGMT,OGG1,PRKDC,HMGB1,TP53BP1,RAD51,BRCA1,H2AFX |
| GO:0045766 | Positive regulation of angiogenesis | 11 | 169 | 3.09E-07 | HGF,SERPINE1,STAT3,TERT,CXCR2,PTGS2,NFE2L2,BRCA1,HIF1A,CYP1B1,VEGFA |
| GO:0001655 | Urogenital system development | 14 | 315 | 3.14E-07 | BMP4,ALDH1A2,TP63,NOTCH1,BAX,CXCR2,RARB,CTNNB1,STAT1,PTEN,AR,TP73,BCL2,VEGFA |
| GO:0016032 | Viral process | 21 | 776 | 3.18E-07 | MAPK1,MDM2,STAT3,RB1,TP53,EGFR,BAX,AXL,CTNNB1,HMGB1,E2F1,CASP8,STAT1,TP73,HSPD1,ERCC2,XRCC5,NFE2L2,EIF4E,H2AFX,ZFP36 |
| GO:0051098 | Regulation of binding | 15 | 373 | 3.32E-07 | TGFB1,IFNG,BMP4,SIRT2,RB1,BAX,TERT,ERCC4,MET,CTNNB1,HMGB1,E2F1,JAK2,ERCC2,CDKN1A |
| GO:0050680 | Negative regulation of epithelial cell proliferation | 10 | 131 | 3.48E-07 | TGFB1,BMP4,RB1,SOX2,STK11,STAT1,PTEN,AR,RUNX3,TNF |
| GO:1900180 | Regulation of protein localization to nucleus | 10 | 131 | 3.48E-07 | MAPK1,TGFB1,IFNG,BMP4,CDH1,TERT,STK11,PTGS2,JAK2,CDKN2A |
| GO:0002009 | Morphogenesis of an epithelium | 16 | 435 | 3.51E-07 | TGFB1,BMP4,ALDH1A2,TP63,EGFR,NOTCH1,PAK1,ADAM17,MET,CXCR2,CTNNB1,AR,BCL2,CD44,HIF1A,VEGFA |
| GO:0001568 | Blood vessel development | 17 | 500 | 3.67E-07 | MAPK1,TGFB1,SERPINE1,BMP4,ALDH1A2,MDM2,EPAS1,PIK3CA,NOTCH1,BAX,CTNNB1,PTGS2,PTEN,YWHAZ,HIF1A,CYP1B1,VEGFA |
| GO:0010660 | Regulation of muscle cell apoptotic process | 8 | 66 | 3.67E-07 | IFNG,TP53,CXCR2,PTEN,JAK2,NFE2L2,CDKN2A,SOD2 |
| GO:0042060 | Wound healing | 16 | 439 | 3.94E-07 | MAPK1,TGFB1,PIK3CA,ERBB2,EGFR,PAK1,AXL,ADAM17,RAD51C,MTOR,JAK2,YWHAZ,IL6,CD44,HIF1A,VEGFA |
| GO:0001894 | Tissue homeostasis | 12 | 219 | 3.97E-07 | NOX4,EPAS1,RB1,NOTCH1,BAX,STK11,CTNNB1,PTGS2,TLR4,BCL2,IL6,VEGFA |
| GO:0031571 | Mitotic g1 dna damage checkpoint | 8 | 67 | 4.05E-07 | CCND1,MDM2,TP53,BAX,PRKDC,E2F1,CDKN1A,MUC1 |
| GO:0046824 | Positive regulation of nucleocytoplasmic transport | 8 | 67 | 4.05E-07 | MAPK1,TGFB1,IFNG,MDM2,CDH1,TP53,PTGS2,JAK2 |
| GO:0071375 | Cellular response to peptide hormone stimulus | 13 | 269 | 4.05E-07 | APEX1,MDM2,PIK3CA,STAT3,IGF1R,PAK1,PRKDC,STAT1,PTEN,JAK2,NFE2L2,GSTP1,PDK2 |
| GO:1903201 | Regulation of oxidative stress-induced cell death | 8 | 67 | 4.05E-07 | HGF,MET,CTNNB1,MCL1,TLR4,NFE2L2,HIF1A,SOD2 |
| GO:0001936 | Regulation of endothelial cell proliferation | 10 | 134 | 4.16E-07 | BMP4,ALDH1A2,STAT3,ADAM17,HMGB1,STAT1,MTOR,TNF,HIF1A,VEGFA |
| GO:0051099 | Positive regulation of binding | 11 | 175 | 4.20E-07 | TGFB1,IFNG,BMP4,SIRT2,RB1,TERT,MET,CTNNB1,HMGB1,JAK2,ERCC2 |
| GO:0070266 | Necroptotic process | 6 | 23 | 4.24E-07 | TP53,FAS,FASLG,TLR4,TNF,BIRC2 |
| GO:0045639 | Positive regulation of myeloid cell differentiation | 9 | 99 | 4.47E-07 | TGFB1,IFNG,STAT3,RB1,PRKDC,CASP8,STAT1,TNF,HIF1A |
| GO:2001243 | Negative regulation of intrinsic apoptotic signaling pathway | 9 | 99 | 4.47E-07 | MDM2,PTGS2,MCL1,NFE2L2,BCL2,CD44,HIF1A,SOD2,MUC1 |
| GO:0022409 | Positive regulation of cell-cell adhesion | 13 | 272 | 4.52E-07 | LGALS1,IFNG,PIK3CA,PAK1,SOX2,PDCD1,HMGB1,CD274,HSPD1,RUNX3,IL6,CD44,TNF |
| GO:0050920 | Regulation of chemotaxis | 12 | 223 | 4.69E-07 | MAPK1,TGFB1,SERPINE1,DUSP1,NOTCH1,ADAM17,MET,CXCR2,HMGB1,GSTP1,IL6,VEGFA |
| GO:0048568 | Embryonic organ development | 16 | 448 | 5.04E-07 | ERCC1,MAPK1,TGFB1,BMP4,ALDH1A2,EPAS1,TP53,EGFR,NOTCH1,RARB,CTNNB1,CASP8,ERCC2,TNF,HIF1A,VEGFA |
| GO:0050863 | Regulation of t cell activation | 14 | 329 | 5.05E-07 | LGALS1,IFNG,BMP4,PIK3CA,ERBB2,PAK1,PDCD1,CTNNB1,HMGB1,CD274,HSPD1,RUNX3,IL6,CDKN2A |
| GO:0009887 | Animal organ morphogenesis | 23 | 967 | 5.85E-07 | MAPK1,TGFB1,SERPINE1,BMP4,ALDH1A2,MDM2,STAT3,TP63,TP53,EGFR,NOTCH1,BAX,RARB,BSG,CTNNB1,MTOR,FASLG,PTEN,AR,BCL2,TNF,HIF1A,VEGFA |
| GO:0032872 | Regulation of stress-activated mapk cascade | 12 | 228 | 5.89E-07 | MAPK1,HGF,DUSP1,IGF1R,EGFR,PAK1,HMGB1,FAS,TLR4,GSTP1,TNF,VEGFA |
| GO:1903039 | Positive regulation of leukocyte cell-cell adhesion | 12 | 228 | 5.89E-07 | LGALS1,IFNG,PIK3CA,PAK1,PDCD1,HMGB1,CD274,HSPD1,RUNX3,IL6,CD44,TNF |
| GO:1901796 | Regulation of signal transduction by p53 class mediator | 11 | 182 | 6.01E-07 | CSNK2A1,MDM2,TP63,TP53,STK11,TP73,BCL2,CD44,CDKN2A,BRCA1,MUC1 |
| GO:0010595 | Positive regulation of endothelial cell migration | 9 | 103 | 6.03E-07 | TGFB1,BMP4,ADAM17,MET,HMGB1,PTGS2,NFE2L2,HIF1A,VEGFA |
| GO:0022408 | Negative regulation of cell-cell adhesion | 11 | 183 | 6.32E-07 | TGFB1,BMP4,CDH1,ERBB2,NOTCH1,HMGB1,CD274,JAK2,RUNX3,CDKN2A,VEGFA |
| GO:0042493 | Response to drug | 13 | 281 | 6.40E-07 | LGALS1,CCND1,XRCC1,TP53,ADAM17,HSPA5,NOS2,CTNNB1,TP73,XRCC5,BCL2,ABCC1,CDKN1A |
| GO:0030217 | T cell differentiation | 10 | 142 | 6.76E-07 | STAT3,TP53,ADAM17,PRKDC,STK11,CTNNB1,HMGB1,MTOR,BCL2,IL6 |
| GO:1903800 | Positive regulation of production of mirnas involved in gene silencing by mirna | 5 | 11 | 6.88E-07 | TGFB1,BMP4,TP53,EGFR,IL6 |
| GO:0010506 | Regulation of autophagy | 14 | 340 | 7.35E-07 | HGF,IFNG,SIRT2,PIK3CA,STAT3,TP53,ERCC4,MET,STK11,HMGB1,MTOR,MCL1,BCL2,HIF1A |
| GO:0120161 | Regulation of cold-induced thermogenesis | 10 | 144 | 7.62E-07 | EPAS1,RB1,IGF1R,NOTCH1,ALDH1A1,ADAM17,STK11,TLR4,JAK2,VEGFA |
| GO:0010718 | Positive regulation of epithelial to mesenchymal transition | 7 | 47 | 7.83E-07 | TGFB1,BMP4,NOTCH1,EZH2,CTNNB1,MTOR,IL6 |
| GO:0048666 | Neuron development | 21 | 827 | 8.60E-07 | MAPK1,CDH1,PIK3CA,RB1,ERBB2,EGFR,NOTCH1,PAK1,USP9X,STK11,BSG,CTNNB1,HMGB1,PTEN,POSTN,JAK2,YWHAZ,BCL2,RUNX3,IL6,VEGFA |
| GO:0070663 | Regulation of leukocyte proliferation | 12 | 237 | 8.64E-07 | MAPK1,BMP4,ERBB2,CTNNB1,HMGB1,TLR4,CD274,BCL2,GSTP1,CDKN1A,IL6,CDKN2A |
| GO:0072422 | Signal transduction involved in dna damage checkpoint | 8 | 75 | 8.64E-07 | MDM2,TP53,BAX,PRKDC,E2F1,CDKN1A,BRCA1,MUC1 |
| GO:0048546 | Digestive tract morphogenesis | 7 | 48 | 8.85E-07 | BMP4,TP63,EGFR,NOTCH1,CTNNB1,BCL2,HIF1A |
| GO:0010661 | Positive regulation of muscle cell apoptotic process | 6 | 27 | 9.27E-07 | IFNG,TP53,CXCR2,PTEN,CDKN2A,SOD2 |
| GO:0044774 | Mitotic dna integrity checkpoint | 9 | 109 | 9.29E-07 | CCND1,MDM2,TP53,BAX,PRKDC,E2F1,CDKN1A,TOP2A,MUC1 |
| GO:1903747 | Regulation of establishment of protein localization to mitochondrion | 8 | 76 | 9.41E-07 | TP63,TP53,E2F1,CASP8,TP73,YWHAZ,BCL2,CDKN2A |
| GO:0031175 | Neuron projection development | 19 | 680 | 9.55E-07 | MAPK1,CDH1,PIK3CA,RB1,ERBB2,EGFR,NOTCH1,PAK1,USP9X,STK11,BSG,CTNNB1,HMGB1,PTEN,POSTN,JAK2,BCL2,IL6,VEGFA |
| GO:0051348 | Negative regulation of transferase activity | 13 | 294 | 1.03E-06 | IFNG,DUSP1,BMP4,RB1,IGF1R,TP53,ERCC4,PTEN,UGT1A1,GSTP1,CDKN1A,CDKN2A,ZFP36 |
| GO:0010575 | Positive regulation of vascular endothelial growth factor production | 6 | 28 | 1.11E-06 | TGFB1,PTGS2,IL6,BRCA1,HIF1A,CYP1B1 |
| GO:0045861 | Negative regulation of proteolysis | 14 | 356 | 1.24E-06 | CSNK2A1,HGF,SERPINE1,MDM2,TIMP3,TP53,BIRC5,PTGS2,XIAP,CD44,TNF,CDKN2A,BIRC2,VEGFA |
| GO:0030278 | Regulation of ossification | 11 | 197 | 1.25E-06 | MAPK1,TGFB1,HGF,BMP4,TP63,NOTCH1,CTNNB1,BCL2,IL6,TNF,HIF1A |
| GO:0090399 | Replicative senescence | 5 | 13 | 1.30E-06 | SERPINE1,TP53,TERT,CDKN1A,CDKN2A |
| GO:0010951 | Negative regulation of endopeptidase activity | 12 | 248 | 1.36E-06 | CSNK2A1,HGF,SERPINE1,MDM2,TIMP3,BIRC5,PTGS2,XIAP,CD44,TNF,BIRC2,VEGFA |
| GO:0045165 | Cell fate commitment | 12 | 248 | 1.36E-06 | BMP4,EPAS1,STAT3,TP53,NOTCH1,PRKDC,SOX2,CTNNB1,MTOR,MCL1,BCL2,IL6 |
| GO:1902893 | Regulation of pri-mirna transcription by rna polymerase ii | 7 | 52 | 1.43E-06 | TGFB1,BMP4,STAT3,TP53,TERT,TNF,HIF1A |
| GO:0055024 | Regulation of cardiac muscle tissue development | 8 | 81 | 1.46E-06 | MAPK1,TGFB1,BMP4,NOTCH1,PAK1,MTOR,PTEN,TP73 |
| GO:0014074 | Response to purine-containing compound | 10 | 156 | 1.50E-06 | APEX1,DUSP1,NOX4,HSPA5,BSG,STAT1,PTGS2,PTEN,HSPD1,BIRC2 |
| GO:1903707 | Negative regulation of hemopoiesis | 10 | 156 | 1.50E-06 | BMP4,ERBB2,NOTCH1,CTNNB1,HMGB1,TLR4,NFE2L2,RUNX3,CDKN2A,ZFP36 |
| GO:0060251 | Regulation of glial cell proliferation | 6 | 30 | 1.56E-06 | NOTCH1,TERT,E2F1,MTOR,IL6,TNF |
| GO:0060147 | Regulation of posttranscriptional gene silencing | 9 | 117 | 1.60E-06 | TGFB1,BMP4,STAT3,TP53,EGFR,TERT,IL6,TNF,ZFP36 |
| GO:0060966 | Regulation of gene silencing by rna | 9 | 117 | 1.60E-06 | TGFB1,BMP4,STAT3,TP53,EGFR,TERT,IL6,TNF,ZFP36 |
| GO:0048729 | Tissue morphogenesis | 17 | 561 | 1.68E-06 | TGFB1,BMP4,ALDH1A2,MDM2,TP63,EGFR,NOTCH1,PAK1,ADAM17,MET,CXCR2,CTNNB1,AR,BCL2,CD44,HIF1A,VEGFA |
| GO:0031669 | Cellular response to nutrient levels | 11 | 204 | 1.71E-06 | MAPK1,MDM2,TP53,HSPA5,FAS,MTOR,POSTN,NFE2L2,BCL2,CDKN1A,PDK2 |
| GO:0034644 | Cellular response to uv | 8 | 83 | 1.71E-06 | ERCC1,MDM2,TP53,BAX,ERCC4,STK11,PTGS2,CDKN1A |
| GO:0043618 | Regulation of transcription from rna polymerase ii promoter in response to stress | 9 | 118 | 1.71E-06 | SIRT2,EPAS1,TP53,NOTCH1,HSPA5,NFE2L2,HIF1A,VEGFA,MUC1 |
| GO:1903799 | Negative regulation of production of mirnas involved in gene silencing by mirna | 5 | 14 | 1.71E-06 | TGFB1,STAT3,TP53,IL6,TNF |
| GO:0048708 | Astrocyte differentiation | 7 | 55 | 1.99E-06 | MAPK1,IFNG,STAT3,EGFR,NOTCH1,TLR4,TNF |
| GO:0070374 | Positive regulation of erk1 and erk2 cascade | 11 | 209 | 2.14E-06 | TGFB1,BMP4,NOX4,EGFR,NOTCH1,HMGB1,PTEN,TLR4,CD44,TNF,VEGFA |
| GO:0051090 | Regulation of dna-binding transcription factor activity | 15 | 437 | 2.18E-06 | MAPK1,STAT3,RB1,EZH2,CTNNB1,PTEN,TLR4,AR,JAK2,TP53BP1,IL6,TNF,CDKN2A,CYP1B1,VEGFA |
| GO:0043536 | Positive regulation of blood vessel endothelial cell migration | 7 | 56 | 2.21E-06 | TGFB1,ADAM17,HMGB1,PTGS2,NFE2L2,HIF1A,VEGFA |
| GO:0070228 | Regulation of lymphocyte apoptotic process | 7 | 56 | 2.21E-06 | BMP4,TP53,BAX,PDCD1,PTEN,CD274,HIF1A |
| GO:0002250 | Adaptive immune response | 13 | 317 | 2.30E-06 | ERCC1,IFNG,MLH1,STAT3,ADAM17,PDCD1,HMGB1,MTOR,TLR4,CD274,JAK2,HSPD1,IL6 |
| GO:0045471 | Response to ethanol | 9 | 123 | 2.33E-06 | CCND1,STAT3,OGG1,TYMS,CASP8,PTEN,UGT1A1,GSTP1,BIRC2 |
| GO:1901215 | Negative regulation of neuron death | 11 | 211 | 2.33E-06 | PIK3CA,STAT3,BAX,AXL,TERT,CTNNB1,JAK2,HSPD1,BCL2,HIF1A,SOD2 |
| GO:0051149 | Positive regulation of muscle cell differentiation | 8 | 88 | 2.56E-06 | TGFB1,BMP4,GDF15,MDM2,CTNNB1,MTOR,BCL2,SOD2 |
| GO:0019953 | Sexual reproduction | 20 | 810 | 2.75E-06 | ERCC1,MLH1,BMP4,STAT3,TP63,NOTCH1,BAX,AXL,USP9X,STK11,RAD51C,CTNNB1,E2F1,MTOR,PTGS2,PTEN,AR,BCL2,TOP2A,H2AFX |
| GO:1905331 | Negative regulation of morphogenesis of an epithelium | 5 | 16 | 2.91E-06 | BMP4,CTNNB1,STAT1,PTEN,TNF |
| GO:0110111 | Negative regulation of animal organ morphogenesis | 6 | 34 | 2.92E-06 | BMP4,NOTCH1,CTNNB1,STAT1,BCL2,TNF |
| GO:0002244 | Hematopoietic progenitor cell differentiation | 8 | 90 | 2.98E-06 | TGFB1,BMP4,TP53,PRKDC,ERCC2,XRCC5,BCL2,TOP2A |
| GO:0010717 | Regulation of epithelial to mesenchymal transition | 8 | 90 | 2.98E-06 | TGFB1,BMP4,NOTCH1,EZH2,CTNNB1,MTOR,PTEN,IL6 |
| GO:0030324 | Lung development | 10 | 170 | 3.07E-06 | MAPK1,BMP4,ALDH1A2,EPAS1,EGFR,NOTCH1,CTNNB1,YWHAZ,EIF4E,VEGFA |
| GO:0007005 | Mitochondrion organization | 15 | 452 | 3.25E-06 | EPAS1,STAT3,TP63,TP53,BAX,TERT,E2F1,CASP8,TP73,SIRT3,HSPD1,YWHAZ,BCL2,CDKN2A,SOD2 |
| GO:0009636 | Response to toxic substance | 11 | 219 | 3.27E-06 | CDH1,BAX,GSTM1,PTGS2,ABCC2,GPX3,NFE2L2,BCL2,GSTP1,CDKN1A,SOD2 |
| GO:0072431 | Signal transduction involved in mitotic g1 dna damage checkpoint | 7 | 60 | 3.32E-06 | MDM2,TP53,BAX,PRKDC,E2F1,CDKN1A,MUC1 |
| GO:0008625 | Extrinsic apoptotic signaling pathway via death domain receptors | 6 | 35 | 3.34E-06 | TNFRSF10A,BAX,CASP8,FASLG,BCL2,TNF |
| GO:0034248 | Regulation of cellular amide metabolic process | 15 | 456 | 3.57E-06 | MAPK1,IFNG,STAT3,TP53,ERBB2,PRKDC,TYMS,MTOR,SIRT3,NFE2L2,IL6,TNF,PDK2,EIF4E,ZFP36 |
| GO:0042306 | Regulation of protein import into nucleus | 7 | 61 | 3.64E-06 | MAPK1,TGFB1,IFNG,BMP4,CDH1,PTGS2,JAK2 |
| GO:0090322 | Regulation of superoxide metabolic process | 6 | 36 | 3.85E-06 | TGFB1,EGFR,SIRT3,NFE2L2,GSTP1,TNF |
| GO:1900087 | Positive regulation of g1/s transition of mitotic cell cycle | 6 | 36 | 3.85E-06 | APEX1,CCND1,MDM2,EGFR,TERT,ADAM17 |
| GO:0007276 | Gamete generation | 18 | 673 | 3.88E-06 | ERCC1,MLH1,BMP4,TP63,NOTCH1,BAX,AXL,USP9X,STK11,RAD51C,CTNNB1,E2F1,MTOR,PTGS2,AR,BCL2,TOP2A,H2AFX |
| GO:0032755 | Positive regulation of interleukin-6 production | 8 | 94 | 3.96E-06 | IFNG,STAT3,BSG,HMGB1,TLR4,HSPD1,IL6,TNF |
| GO:1903426 | Regulation of reactive oxygen species biosynthetic process | 8 | 95 | 4.26E-06 | IFNG,STAT3,MTOR,PTGS2,TLR4,JAK2,HSPD1,TNF |
| GO:0048771 | Tissue remodeling | 8 | 96 | 4.59E-06 | TGFB1,MDM2,NOX4,EPAS1,BAX,AXL,CTNNB1,HIF1A |
| GO:0051179 | Localization | 61 | 5591 | 4.98E-06 | MAPK1,TNFRSF10A,TGFB1,HGF,SERPINE1,CCND1,MLH1,BMP4,MDM2,CDH1,PIK3CA,STAT3,TP63,TIMP3,RB1,IGF1R,TP53,EGFR,NOTCH1,PAK1,BAX,AXL,BIRC5,TERT,ADAM17,USP9X,CXCR2,EZH2,HSPA5,STK11,NOS2,BSG,CTNNB1,HMGB1,E2F1,CASP8,MTOR,FASLG,MCL1,ABCC2,PTEN,ABCC10,TLR4,TP73,CD274,HSPD1,XRCC5,YWHAZ,BCL2,GSTP1,ABCC1,CDKN1A,IL6,CD44,TNF,EIF4E,HIF1A,SLC16A1,ZFP36,CYP1B1,VEGFA |
| GO:0043491 | Protein kinase b signaling | 6 | 38 | 5.07E-06 | TGFB1,SIRT2,PIK3CA,AXL,PTEN,TNF |
| GO:0002831 | Regulation of response to biotic stimulus | 14 | 406 | 5.33E-06 | IFNG,SIRT2,PAK1,PRKDC,HMGB1,STAT1,XIAP,TLR4,CD274,JAK2,HSPD1,XRCC5,BIRC2,MUC1 |
| GO:0022612 | Gland morphogenesis | 8 | 99 | 5.64E-06 | TGFB1,BMP4,TP63,EGFR,NOTCH1,AR,BCL2,TNF |
| GO:1903047 | Mitotic cell cycle process | 17 | 616 | 5.64E-06 | TGFB1,CCND1,MDM2,RB1,TP53,BAX,BIRC5,PRKDC,TYMS,E2F1,RAD51,CDKN1A,TOP2A,CDKN2A,BRCA1,EIF4E,MUC1 |
| GO:0014911 | Positive regulation of smooth muscle cell migration | 6 | 39 | 5.76E-06 | MDM2,NOX4,PAK1,TERT,POSTN,BCL2 |
| GO:0051345 | Positive regulation of hydrolase activity | 19 | 772 | 5.85E-06 | TNFRSF10A,IFNG,TNFSF10,STAT3,ERBB2,EGFR,BAX,ALDH1A1,EZH2,HMGB1,FAS,CASP8,MTOR,FASLG,JAK2,HSPD1,TNF,CDKN2A,VEGFA |
| GO:0048514 | Blood vessel morphogenesis | 14 | 410 | 5.94E-06 | TGFB1,SERPINE1,BMP4,EPAS1,PIK3CA,NOTCH1,BAX,CTNNB1,PTGS2,PTEN,YWHAZ,HIF1A,CYP1B1,VEGFA |
| GO:0000278 | Mitotic cell cycle | 18 | 695 | 6.01E-06 | TGFB1,HGF,CCND1,MDM2,RB1,TP53,BAX,BIRC5,PRKDC,TYMS,E2F1,RAD51,CDKN1A,TOP2A,CDKN2A,BRCA1,EIF4E,MUC1 |
| GO:1902895 | Positive regulation of pri-mirna transcription by rna polymerase ii | 6 | 40 | 6.54E-06 | TGFB1,STAT3,TP53,TERT,TNF,HIF1A |
| GO:0003013 | Circulatory system process | 14 | 415 | 6.80E-06 | TGFB1,NOX4,PIK3CA,EGFR,CXCR2,NOS2,STAT1,MTOR,PTGS2,ABCC2,AR,POSTN,SOD2,VEGFA |
| GO:0021700 | Developmental maturation | 11 | 238 | 6.89E-06 | ALDH1A2,EPAS1,RB1,AXL,TYMS,CTNNB1,PTEN,ERCC2,CDKN1A,HIF1A,VEGFA |
| GO:0032675 | Regulation of interleukin-6 production | 9 | 142 | 6.89E-06 | HGF,IFNG,STAT3,BSG,HMGB1,TLR4,HSPD1,IL6,TNF |
| GO:0051153 | Regulation of striated muscle cell differentiation | 8 | 102 | 6.89E-06 | TGFB1,BMP4,GDF15,NOTCH1,PAK1,EZH2,MTOR,BCL2 |
| GO:0045844 | Positive regulation of striated muscle tissue development | 7 | 68 | 6.91E-06 | MAPK1,TGFB1,BMP4,NOTCH1,CTNNB1,MTOR,BCL2 |
| GO:0051769 | Regulation of nitric-oxide synthase biosynthetic process | 5 | 20 | 6.96E-06 | IFNG,STAT1,TLR4,JAK2,GSTP1 |
| GO:0042307 | Positive regulation of protein import into nucleus | 6 | 41 | 7.37E-06 | MAPK1,TGFB1,IFNG,CDH1,PTGS2,JAK2 |
| GO:0001776 | Leukocyte homeostasis | 7 | 69 | 7.53E-06 | BAX,AXL,HMGB1,FAS,BCL2,IL6,HIF1A |
| GO:0051983 | Regulation of chromosome segregation | 8 | 104 | 7.83E-06 | CSNK2A1,DUSP1,SIRT2,RB1,BIRC5,CTNNB1,MKI67,XRCC3 |
| GO:0030850 | Prostate gland development | 6 | 42 | 8.34E-06 | BMP4,TP63,NOTCH1,CTNNB1,PTEN,AR |
| GO:0045739 | Positive regulation of dna repair | 7 | 71 | 8.93E-06 | XRCC1,EGFR,MGMT,PRKDC,HMGB1,BRCA1,H2AFX |
| GO:0050921 | Positive regulation of chemotaxis | 9 | 147 | 8.93E-06 | MAPK1,TGFB1,SERPINE1,ADAM17,MET,CXCR2,HMGB1,IL6,VEGFA |
| GO:0070301 | Cellular response to hydrogen peroxide | 7 | 71 | 8.93E-06 | APEX1,MDM2,AXL,EZH2,NFE2L2,IL6,CYP1B1 |
| GO:2001022 | Positive regulation of response to dna damage stimulus | 8 | 106 | 8.93E-06 | XRCC1,EGFR,MGMT,PRKDC,HMGB1,CDKN2A,BRCA1,H2AFX |
| GO:0043009 | Chordate embryonic development | 17 | 640 | 9.15E-06 | MAPK1,TGFB1,BMP4,ALDH1A2,EPAS1,TP53,EGFR,NOTCH1,PRKDC,CTNNB1,CASP8,AR,KDM4C,ERCC2,BRCA1,HIF1A,VEGFA |
| GO:0002460 | Adaptive immune response based on somatic recombination of immune receptors built from immunoglobulin superfamily domains | 9 | 148 | 9.36E-06 | ERCC1,MLH1,STAT3,ADAM17,HMGB1,MTOR,TLR4,HSPD1,IL6 |
| GO:0043524 | Negative regulation of neuron apoptotic process | 9 | 148 | 9.36E-06 | PIK3CA,BAX,AXL,TERT,JAK2,HSPD1,BCL2,HIF1A,SOD2 |
| GO:0002064 | Epithelial cell development | 10 | 195 | 9.46E-06 | BMP4,TP63,NOTCH1,TYMS,MET,RARB,CTNNB1,AR,CDKN1A,HIF1A |
| GO:0014015 | Positive regulation of gliogenesis | 7 | 72 | 9.65E-06 | TGFB1,NOTCH1,E2F1,MTOR,TP73,IL6,TNF |
| GO:0002252 | Immune effector process | 21 | 969 | 9.69E-06 | ERCC1,MAPK1,LGALS1,IFNG,MLH1,PIK3CA,STAT3,TP53,PAK1,ADAM17,CXCR2,HMGB1,STAT1,MTOR,TLR4,HSPD1,XRCC5,BCL2,GSTP1,IL6,CD44 |
| GO:0014912 | Negative regulation of smooth muscle cell migration | 5 | 22 | 1.02E-05 | APEX1,SERPINE1,IGFBP3,NFE2L2,GSTP1 |
| GO:0045429 | Positive regulation of nitric oxide biosynthetic process | 6 | 44 | 1.05E-05 | IFNG,MTOR,PTGS2,TLR4,JAK2,TNF |
| GO:0042594 | Response to starvation | 10 | 198 | 1.07E-05 | MAPK1,TP53,HSPA5,FAS,MTOR,UGT1A1,NFE2L2,BCL2,CDKN1A,ZFP36 |
| GO:0043122 | Regulation of i-kappab kinase/nf-kappab signaling | 11 | 251 | 1.10E-05 | LGALS1,TNFSF10,CTNNB1,CASP8,STAT1,FASLG,S100A4,TLR4,GSTP1,TNF,BIRC2 |
| GO:1901654 | Response to ketone | 10 | 199 | 1.11E-05 | TGFB1,CCND1,DUSP1,EGFR,TYMS,ABCC2,AR,SPP1,CDKN1A,EIF4E |
| GO:0071695 | Anatomical structure maturation | 10 | 200 | 1.16E-05 | ALDH1A2,EPAS1,RB1,AXL,TYMS,CTNNB1,ERCC2,CDKN1A,HIF1A,VEGFA |
| GO:0001890 | Placenta development | 9 | 154 | 1.25E-05 | MAPK1,EPAS1,EGFR,BSG,CASP8,PTGS2,SPP1,HIF1A,BIRC2 |
| GO:0050878 | Regulation of body fluid levels | 15 | 509 | 1.27E-05 | MAPK1,SERPINE1,CCND1,PIK3CA,TP63,AXL,MET,RAD51C,TLR4,JAK2,YWHAZ,NFE2L2,IL6,HIF1A,VEGFA |
| GO:0021782 | Glial cell development | 8 | 112 | 1.28E-05 | IFNG,SIRT2,EGFR,PTEN,TLR4,ERCC2,GSTP1,TNF |
| GO:0031641 | Regulation of myelination | 6 | 46 | 1.31E-05 | HGF,SIRT2,RARB,CTNNB1,MTOR,PTEN |
| GO:0048662 | Negative regulation of smooth muscle cell proliferation | 6 | 46 | 1.31E-05 | IFNG,PTEN,IGFBP3,GSTP1,CDKN1A,SOD2 |
| GO:1903555 | Regulation of tumor necrosis factor superfamily cytokine production | 9 | 155 | 1.31E-05 | IFNG,AXL,HMGB1,TLR4,CD274,JAK2,HSPD1,GSTP1,ZFP36 |
| GO:0001525 | Angiogenesis | 12 | 315 | 1.36E-05 | SERPINE1,BMP4,EPAS1,PIK3CA,NOTCH1,CTNNB1,PTGS2,PTEN,YWHAZ,HIF1A,CYP1B1,VEGFA |
| GO:0045666 | Positive regulation of neuron differentiation | 13 | 377 | 1.36E-05 | HGF,BMP4,PAK1,EZH2,HSPA5,STK11,RARB,MTOR,PTEN,KDM4C,NFE2L2,BCL2,VEGFA |
| GO:1902107 | Positive regulation of leukocyte differentiation | 9 | 156 | 1.37E-05 | TGFB1,IFNG,RB1,AXL,PRKDC,HMGB1,CASP8,RUNX3,TNF |
| GO:0007548 | Sex differentiation | 11 | 259 | 1.44E-05 | ERCC1,CCND1,TNFSF10,TP63,BAX,AXL,HSPA5,CTNNB1,AR,BCL2,VEGFA |
| GO:0060964 | Regulation of gene silencing by mirna | 8 | 114 | 1.44E-05 | TGFB1,BMP4,STAT3,TP53,EGFR,IL6,TNF,ZFP36 |
| GO:0120163 | Negative regulation of cold-induced thermogenesis | 6 | 47 | 1.45E-05 | RB1,NOTCH1,ALDH1A1,ADAM17,STK11,TLR4 |
| GO:1903202 | Negative regulation of oxidative stress-induced cell death | 6 | 47 | 1.45E-05 | HGF,MET,CTNNB1,NFE2L2,HIF1A,SOD2 |
| GO:0048469 | Cell maturation | 9 | 158 | 1.50E-05 | EPAS1,RB1,AXL,TYMS,CTNNB1,ERCC2,CDKN1A,HIF1A,VEGFA |
| GO:0061418 | Regulation of transcription from rna polymerase ii promoter in response to hypoxia | 7 | 78 | 1.53E-05 | SIRT2,EPAS1,TP53,NOTCH1,NFE2L2,HIF1A,VEGFA |
| GO:0061138 | Morphogenesis of a branching epithelium | 9 | 159 | 1.58E-05 | BMP4,TP63,NOTCH1,PAK1,MET,CTNNB1,AR,BCL2,VEGFA |
| GO:0048738 | Cardiac muscle tissue development | 9 | 160 | 1.65E-05 | TGFB1,BMP4,ALDH1A2,NOX4,NOTCH1,RARB,MTOR,PTEN,VEGFA |
| GO:0050870 | Positive regulation of t cell activation | 10 | 209 | 1.65E-05 | LGALS1,IFNG,PIK3CA,PAK1,PDCD1,HMGB1,CD274,HSPD1,RUNX3,IL6 |
| GO:0010817 | Regulation of hormone levels | 15 | 524 | 1.76E-05 | IFNG,ALDH1A2,EGFR,ALDH1A1,NOS2,ABCC2,UGT1A1,JAK2,SIRT3,SPP1,IL6,TNF,HIF1A,SLC16A1,CYP1B1 |
| GO:0032481 | Positive regulation of type i interferon production | 7 | 80 | 1.78E-05 | PRKDC,CTNNB1,HMGB1,STAT1,TLR4,HSPD1,XRCC5 |
| GO:0032990 | Cell part morphogenesis | 15 | 525 | 1.80E-05 | MAPK1,PIK3CA,ERBB2,EGFR,NOTCH1,PAK1,BAX,USP9X,STK11,BSG,CTNNB1,PTEN,POSTN,BCL2,VEGFA |
| GO:0034250 | Positive regulation of cellular amide metabolic process | 9 | 162 | 1.81E-05 | MAPK1,IFNG,ERBB2,PRKDC,MTOR,SIRT3,NFE2L2,IL6,TNF |
| GO:0045927 | Positive regulation of growth | 11 | 267 | 1.89E-05 | MAPK1,CSNK2A1,ERBB2,EGFR,NOTCH1,PAK1,ADAM17,PRKDC,MTOR,BCL2,VEGFA |
| GO:0045843 | Negative regulation of striated muscle tissue development | 6 | 50 | 1.98E-05 | TGFB1,BMP4,SIRT2,PAK1,PTEN,TP73 |
| GO:0090342 | Regulation of cell aging | 6 | 50 | 1.98E-05 | TP63,TP53,TERT,PRKDC,PTEN,CDKN2A |
| GO:0030330 | DNA damage response, signal transduction by p53 class mediator | 7 | 82 | 2.06E-05 | MDM2,TP53,BAX,E2F1,CDKN1A,BRCA1,MUC1 |
| GO:0002761 | Regulation of myeloid leukocyte differentiation | 8 | 121 | 2.14E-05 | TGFB1,IFNG,RB1,CTNNB1,CASP8,MTOR,TLR4,TNF |
| GO:0001822 | Kidney development | 11 | 271 | 2.15E-05 | BMP4,ALDH1A2,NOTCH1,BAX,CXCR2,RARB,CTNNB1,STAT1,TP73,BCL2,VEGFA |
| GO:0060768 | Regulation of epithelial cell proliferation involved in prostate gland development | 4 | 10 | 2.15E-05 | NOTCH1,STK11,CTNNB1,AR |
| GO:0030522 | Intracellular receptor signaling pathway | 9 | 166 | 2.17E-05 | ALDH1A2,STAT3,RB1,RARB,CTNNB1,CASP8,AR,JAK2,BRCA1 |
| GO:0033077 | T cell differentiation in thymus | 6 | 51 | 2.18E-05 | TP53,ADAM17,PRKDC,STK11,CTNNB1,BCL2 |
| GO:0006928 | Movement of cell or subcellular component | 26 | 1501 | 2.27E-05 | MAPK1,TNFRSF10A,TGFB1,HGF,PIK3CA,ERBB2,EGFR,NOTCH1,PAK1,BAX,AXL,ADAM17,USP9X,CXCR2,BSG,CTNNB1,HMGB1,PTEN,ABCC1,IL6,CD44,TNF,HIF1A,SLC16A1,CYP1B1,VEGFA |
| GO:0050714 | Positive regulation of protein secretion | 9 | 167 | 2.27E-05 | TGFB1,IFNG,EGFR,TLR4,JAK2,SIRT3,IL6,TNF,HIF1A |
| GO:0009896 | Positive regulation of catabolic process | 14 | 465 | 2.28E-05 | CSNK2A1,IFNG,SIRT2,MDM2,RB1,BAX,STK11,HMGB1,PTEN,NFE2L2,IL6,TNF,HIF1A,ZFP36 |
| GO:0031331 | Positive regulation of cellular catabolic process | 13 | 397 | 2.28E-05 | IFNG,SIRT2,MDM2,RB1,BAX,STK11,HMGB1,PTEN,NFE2L2,IL6,TNF,HIF1A,ZFP36 |
| GO:0050670 | Regulation of lymphocyte proliferation | 10 | 218 | 2.31E-05 | BMP4,ERBB2,CTNNB1,HMGB1,TLR4,CD274,BCL2,CDKN1A,IL6,CDKN2A |
| GO:0043368 | Positive t cell selection | 5 | 27 | 2.32E-05 | STAT3,STK11,MTOR,BCL2,IL6 |
| GO:0014066 | Regulation of phosphatidylinositol 3-kinase signaling | 8 | 123 | 2.37E-05 | MAPK1,HGF,PIK3CA,IGF1R,EGFR,PTEN,JAK2,TNF |
| GO:0003179 | Heart valve morphogenesis | 6 | 52 | 2.39E-05 | TGFB1,BMP4,MDM2,RB1,NOTCH1,MTOR |
| GO:0032989 | Cellular component morphogenesis | 16 | 614 | 2.40E-05 | MAPK1,PIK3CA,ERBB2,EGFR,NOTCH1,PAK1,BAX,USP9X,STK11,BSG,CTNNB1,PTEN,POSTN,ERCC2,BCL2,VEGFA |
| GO:0002688 | Regulation of leukocyte chemotaxis | 8 | 124 | 2.49E-05 | MAPK1,SERPINE1,DUSP1,ADAM17,CXCR2,HMGB1,IL6,VEGFA |
| GO:0032651 | Regulation of interleukin-1 beta production | 7 | 85 | 2.52E-05 | IFNG,HMGB1,CASP8,TLR4,JAK2,GSTP1,TNF |
| GO:0046427 | Positive regulation of receptor signaling pathway via jak-stat | 7 | 85 | 2.52E-05 | IFNG,STAT3,NOTCH1,JAK2,IL6,TNF,CYP1B1 |
| GO:0050871 | Positive regulation of b cell activation | 7 | 85 | 2.52E-05 | TGFB1,MLH1,TLR4,TP53BP1,BCL2,CDKN1A,IL6 |
| GO:0044089 | Positive regulation of cellular component biogenesis | 15 | 542 | 2.54E-05 | ERCC1,TGFB1,IFNG,NOX4,TP53,PAK1,BAX,ERCC4,MET,ERCC5,MTOR,TLR4,ERCC2,TNF,VEGFA |
| GO:0030099 | Myeloid cell differentiation | 10 | 221 | 2.56E-05 | IFNG,BMP4,EPAS1,RB1,CTNNB1,CASP8,JAK2,ERCC2,TNF,VEGFA |
| GO:0009791 | Post-embryonic development | 7 | 86 | 2.69E-05 | ERCC1,BMP4,BAX,MTOR,ERCC2,BCL2,VEGFA |
| GO:0001818 | Negative regulation of cytokine production | 11 | 280 | 2.83E-05 | TGFB1,HGF,IFNG,AXL,HMGB1,TLR4,CD274,GSTP1,IL6,TNF,ZFP36 |
| GO:0043276 | Anoikis | 4 | 11 | 2.83E-05 | PIK3CA,STK11,E2F1,MTOR |
| GO:2001269 | Positive regulation of cysteine-type endopeptidase activity involved in apoptotic signaling pathway | 4 | 11 | 2.83E-05 | BAX,FAS,CASP8,JAK2 |
| GO:0006839 | Mitochondrial transport | 10 | 225 | 2.97E-05 | STAT3,TP63,TP53,BAX,E2F1,CASP8,TP73,HSPD1,YWHAZ,BCL2 |
| GO:0002366 | Leukocyte activation involved in immune response | 16 | 626 | 3.00E-05 | ERCC1,MAPK1,LGALS1,IFNG,MLH1,STAT3,TP53,CXCR2,HMGB1,MTOR,TLR4,HSPD1,XRCC5,GSTP1,IL6,CD44 |
| GO:0050864 | Regulation of b cell activation | 8 | 128 | 3.06E-05 | TGFB1,MLH1,TLR4,TP53BP1,BCL2,CDKN1A,IL6,CDKN2A |
| GO:0045682 | Regulation of epidermis development | 7 | 88 | 3.07E-05 | BMP4,TP63,NOTCH1,EZH2,CTNNB1,TNF,ZFP36 |
| GO:0050673 | Epithelial cell proliferation | 7 | 88 | 3.07E-05 | MAPK1,HGF,CCND1,BMP4,BAX,IGFBP3,TNF |
| GO:0035116 | Embryonic hindlimb morphogenesis | 5 | 29 | 3.08E-05 | BMP4,TP63,NOTCH1,RARB,CTNNB1 |
| GO:0032731 | Positive regulation of interleukin-1 beta production | 6 | 55 | 3.14E-05 | IFNG,HMGB1,CASP8,TLR4,JAK2,TNF |
| GO:0060688 | Regulation of morphogenesis of a branching structure | 6 | 56 | 3.45E-05 | HGF,BMP4,CTNNB1,AR,TNF,VEGFA |
| GO:0034101 | Erythrocyte homeostasis | 7 | 90 | 3.50E-05 | BMP4,EPAS1,RB1,AXL,JAK2,ERCC2,VEGFA |
| GO:0042176 | Regulation of protein catabolic process | 13 | 415 | 3.51E-05 | CSNK2A1,IFNG,SIRT2,MDM2,CDH1,TIMP3,RB1,EGFR,NOS2,PTEN,NFE2L2,TNF,CDKN2A |
| GO:0033032 | Regulation of myeloid cell apoptotic process | 5 | 30 | 3.54E-05 | ADAM17,CXCR2,PTEN,BCL2,CDKN2A |
| GO:2000108 | Positive regulation of leukocyte apoptotic process | 5 | 30 | 3.54E-05 | TP53,BAX,PDCD1,CD274,CDKN2A |
| GO:0048754 | Branching morphogenesis of an epithelial tube | 8 | 131 | 3.55E-05 | BMP4,NOTCH1,PAK1,MET,CTNNB1,AR,BCL2,VEGFA |
| GO:0032868 | Response to insulin | 10 | 231 | 3.64E-05 | TNFSF10,PIK3CA,IGF1R,PAK1,PRKDC,STAT1,MTOR,PTEN,GSTP1,PDK2 |
| GO:0048012 | Hepatocyte growth factor receptor signaling pathway | 4 | 12 | 3.66E-05 | HGF,SIRT2,PAK1,MET |
| GO:0060742 | Epithelial cell differentiation involved in prostate gland development | 4 | 12 | 3.66E-05 | TP63,NOTCH1,CTNNB1,AR |
| GO:2000343 | Positive regulation of chemokine (c-x-c motif) ligand 2 production | 4 | 12 | 3.66E-05 | HMGB1,TLR4,POSTN,TNF |
| GO:0042633 | Hair cycle | 7 | 91 | 3.71E-05 | TP63,EGFR,NOTCH1,CTNNB1,PTGS2,ERCC2,BCL2 |
| GO:0008637 | Apoptotic mitochondrial changes | 6 | 57 | 3.73E-05 | TP53,BAX,HSPD1,BCL2,CDKN2A,SOD2 |
| GO:0002697 | Regulation of immune effector process | 13 | 418 | 3.74E-05 | TGFB1,IFNG,MLH1,PRKDC,NOS2,HMGB1,STAT1,TLR4,TP53BP1,HSPD1,IL6,TNF,BIRC2 |
| GO:0007423 | Sensory organ development | 15 | 563 | 3.85E-05 | MAPK1,BMP4,ALDH1A2,STAT3,EGFR,NOTCH1,BAX,SOX2,RARB,CTNNB1,FASLG,BCL2,HIF1A,CYP1B1,VEGFA |
| GO:1905330 | Regulation of morphogenesis of an epithelium | 9 | 180 | 3.85E-05 | HGF,BMP4,CTNNB1,STAT1,MTOR,PTEN,AR,TNF,VEGFA |
| GO:0045598 | Regulation of fat cell differentiation | 8 | 133 | 3.89E-05 | TGFB1,SIRT2,E2F1,MTOR,PTGS2,IL6,TNF,ZFP36 |
| GO:0043525 | Positive regulation of neuron apoptotic process | 6 | 58 | 4.07E-05 | TP53,BAX,CTNNB1,FASLG,MCL1,TNF |
| GO:0000904 | Cell morphogenesis involved in differentiation | 15 | 566 | 4.08E-05 | MAPK1,PIK3CA,RB1,ERBB2,NOTCH1,AXL,USP9X,MET,STK11,BSG,CTNNB1,PTEN,AR,BCL2,VEGFA |
| GO:0031647 | Regulation of protein stability | 11 | 293 | 4.14E-05 | MAPK1,MDM2,TP53,TERT,PRKDC,USP9X,PTEN,HSPD1,BCL2,CDKN1A,CDKN2A |
| GO:0048812 | Neuron projection morphogenesis | 14 | 495 | 4.35E-05 | MAPK1,PIK3CA,ERBB2,EGFR,NOTCH1,PAK1,USP9X,STK11,BSG,CTNNB1,PTEN,POSTN,BCL2,VEGFA |
| GO:0006977 | DNA damage response, signal transduction by p53 class mediator resulting in cell cycle arrest | 6 | 59 | 4.44E-05 | MDM2,TP53,BAX,E2F1,CDKN1A,MUC1 |
| GO:0071385 | Cellular response to glucocorticoid stimulus | 6 | 59 | 4.44E-05 | EGFR,ABCC2,UGT1A1,GSTP1,EIF4E,ZFP36 |
| GO:0001938 | Positive regulation of endothelial cell proliferation | 7 | 94 | 4.47E-05 | BMP4,STAT3,ADAM17,HMGB1,MTOR,HIF1A,VEGFA |
| GO:0007292 | Female gamete generation | 8 | 136 | 4.51E-05 | ERCC1,MLH1,USP9X,RAD51C,CTNNB1,PTGS2,BCL2,TOP2A |
| GO:2001252 | Positive regulation of chromosome organization | 9 | 184 | 4.51E-05 | ERCC1,MAPK1,RB1,TP53,CTNNB1,XRCC5,BRCA1,VEGFA,MUC1 |
| GO:1902253 | Regulation of intrinsic apoptotic signaling pathway by p53 class mediator | 5 | 32 | 4.57E-05 | MDM2,TP53,BCL2,CD44,MUC1 |
| GO:1902692 | Regulation of neuroblast proliferation | 5 | 32 | 4.57E-05 | TP53,NOTCH1,CTNNB1,HIF1A,VEGFA |
| GO:0006266 | DNA ligation | 4 | 13 | 4.65E-05 | XRCC1,MGMT,HMGB1,TOP2A |
| GO:0009650 | UV protection | 4 | 13 | 4.65E-05 | ERCC1,ERCC4,ERCC5,ERCC2 |
| GO:2001251 | Negative regulation of chromosome organization | 8 | 137 | 4.71E-05 | ERCC1,XRCC1,BIRC5,ERCC4,KDM4C,XRCC5,TOP2A,BRCA1 |
| GO:0030308 | Negative regulation of cell growth | 9 | 187 | 5.08E-05 | TGFB1,RB1,TP53,PAK1,STK11,SPP1,BCL2,CDKN1A,CDKN2A |
| GO:0071354 | Cellular response to interleukin-6 | 5 | 33 | 5.19E-05 | STAT3,STAT1,ABCC2,JAK2,IL6 |
| GO:0045088 | Regulation of innate immune response | 11 | 301 | 5.21E-05 | IFNG,PAK1,PRKDC,HMGB1,STAT1,XIAP,TLR4,JAK2,XRCC5,BIRC2,MUC1 |
| GO:0055021 | Regulation of cardiac muscle tissue growth | 6 | 61 | 5.22E-05 | MAPK1,NOTCH1,PAK1,MTOR,PTEN,TP73 |
| GO:1903320 | Regulation of protein modification by small protein conjugation or removal | 10 | 242 | 5.22E-05 | HSPA5,MTA1,CTNNB1,MTOR,XIAP,PTEN,BMI1,CDKN2A,BRCA1,BIRC2 |
| GO:0001654 | Eye development | 12 | 365 | 5.23E-05 | BMP4,ALDH1A2,STAT3,EGFR,BAX,SOX2,RARB,CTNNB1,FASLG,HIF1A,CYP1B1,VEGFA |
| GO:0042593 | Glucose homeostasis | 9 | 188 | 5.23E-05 | LGALS1,NOX4,PIK3CA,STAT3,IGF1R,STK11,PDK2,HIF1A,SLC16A1 |
| GO:0090092 | Regulation of transmembrane receptor protein serine/threonine kinase signaling pathway | 10 | 244 | 5.55E-05 | TGFB1,BMP4,GDF15,TP53,NOTCH1,ADAM17,HSPA5,STK11,XIAP,JAK2 |
| GO:0002690 | Positive regulation of leukocyte chemotaxis | 7 | 98 | 5.62E-05 | MAPK1,SERPINE1,ADAM17,CXCR2,HMGB1,IL6,VEGFA |
| GO:0010812 | Negative regulation of cell-substrate adhesion | 6 | 62 | 5.62E-05 | LGALS1,SERPINE1,NOTCH1,PTEN,POSTN,CDKN2A |
| GO:0051591 | Response to camp | 7 | 98 | 5.62E-05 | APEX1,DUSP1,NOX4,HSPA5,BSG,STAT1,BIRC2 |
| GO:0002363 | Alpha-beta t cell lineage commitment | 4 | 14 | 5.79E-05 | STAT3,MTOR,BCL2,IL6 |
| GO:0071236 | Cellular response to antibiotic | 4 | 14 | 5.79E-05 | MDM2,TP53,EZH2,HSPA5 |
| GO:0045601 | Regulation of endothelial cell differentiation | 5 | 34 | 5.80E-05 | BMP4,NOTCH1,CTNNB1,TNF,VEGFA |
| GO:0060562 | Epithelial tube morphogenesis | 11 | 307 | 6.10E-05 | TGFB1,BMP4,NOTCH1,PAK1,MET,CXCR2,CTNNB1,AR,BCL2,HIF1A,VEGFA |
| GO:0002687 | Positive regulation of leukocyte migration | 8 | 144 | 6.49E-05 | MAPK1,SERPINE1,ADAM17,CXCR2,HMGB1,IL6,TNF,VEGFA |
| GO:0002562 | Somatic diversification of immune receptors via germline recombination within a single locus | 5 | 35 | 6.54E-05 | ERCC1,MLH1,PRKDC,HMGB1,HSPD1 |
| GO:0010665 | Regulation of cardiac muscle cell apoptotic process | 5 | 35 | 6.54E-05 | TP53,CXCR2,PTEN,JAK2,NFE2L2 |
| GO:0043123 | Positive regulation of i-kappab kinase/nf-kappab signaling | 9 | 194 | 6.54E-05 | LGALS1,TNFSF10,CTNNB1,CASP8,FASLG,S100A4,TLR4,TNF,BIRC2 |
| GO:0060135 | Maternal process involved in female pregnancy | 6 | 64 | 6.54E-05 | MAPK1,BSG,MTOR,PTGS2,AR,SPP1 |
| GO:1901031 | Regulation of response to reactive oxygen species | 5 | 35 | 6.54E-05 | HGF,MET,SIRT3,NFE2L2,TNF |
| GO:1904707 | Positive regulation of vascular associated smooth muscle cell proliferation | 5 | 35 | 6.54E-05 | MDM2,PAK1,TERT,JAK2,TNF |
| GO:1905332 | Positive regulation of morphogenesis of an epithelium | 5 | 35 | 6.54E-05 | BMP4,CTNNB1,MTOR,AR,VEGFA |
| GO:0061819 | Telomeric dna-containing double minutes formation | 3 | 3 | 6.58E-05 | ERCC1,XRCC1,ERCC4 |
| GO:1905765 | Negative regulation of protection from non-homologous end joining at telomere | 3 | 3 | 6.58E-05 | ERCC1,XRCC1,ERCC4 |
| GO:0002065 | Columnar/cuboidal epithelial cell differentiation | 7 | 101 | 6.61E-05 | BMP4,TP63,NOTCH1,TYMS,RARB,CDKN1A,HIF1A |
| GO:0002824 | Positive regulation of adaptive immune response based on somatic recombination of immune receptors built from immunoglobulin superfamily domains | 7 | 101 | 6.61E-05 | TGFB1,MLH1,CD274,TP53BP1,HSPD1,IL6,TNF |
| GO:0003007 | Heart morphogenesis | 10 | 251 | 6.85E-05 | TGFB1,BMP4,ALDH1A2,MDM2,TP53,NOTCH1,RARB,MTOR,HIF1A,VEGFA |
| GO:0000724 | Double-strand break repair via homologous recombination | 7 | 102 | 7.01E-05 | XRCC1,ERCC4,RAD51C,RAD51,BRCA1,H2AFX,XRCC3 |
| GO:0006978 | DNA damage response, signal transduction by p53 class mediator resulting in transcription of p21 class mediator | 4 | 15 | 7.10E-05 | TP53,CDKN1A,BRCA1,MUC1 |
| GO:0043369 | CD4-positive or CD8-positive, alpha-beta T cell lineage commitment | 4 | 15 | 7.10E-05 | STAT3,MTOR,BCL2,IL6 |
| GO:0060252 | Positive regulation of glial cell proliferation | 4 | 15 | 7.10E-05 | E2F1,MTOR,IL6,TNF |
| GO:0014065 | Phosphatidylinositol 3-kinase signaling | 5 | 36 | 7.26E-05 | SIRT2,PIK3CA,IGF1R,ERBB2,PTEN |
| GO:0048645 | Animal organ formation | 5 | 36 | 7.26E-05 | MAPK1,BMP4,TP63,CTNNB1,AR |
| GO:0070232 | Regulation of t cell apoptotic process | 5 | 36 | 7.26E-05 | BMP4,TP53,PDCD1,CD274,HIF1A |
| GO:0016458 | Gene silencing | 8 | 147 | 7.31E-05 | SIRT2,STAT3,TERT,EZH2,HMGB1,BMI1,H2AFX,ZFP36 |
| GO:1903322 | Positive regulation of protein modification by small protein conjugation or removal | 8 | 147 | 7.31E-05 | HSPA5,MTA1,XIAP,PTEN,BMI1,CDKN2A,BRCA1,BIRC2 |
| GO:0062012 | Regulation of small molecule metabolic process | 13 | 449 | 7.35E-05 | TGFB1,IFNG,STAT3,TP53,NOS2,MTOR,PTGS2,UGT1A1,IGFBP3,TNF,BRCA1,PDK2,HIF1A |
| GO:0051346 | Negative regulation of hydrolase activity | 13 | 450 | 7.52E-05 | CSNK2A1,HGF,SERPINE1,MDM2,TIMP3,TP53,BIRC5,PTGS2,XIAP,CD44,TNF,BIRC2,VEGFA |
| GO:0050900 | Leukocyte migration | 11 | 316 | 7.71E-05 | TNFRSF10A,TGFB1,PIK3CA,CXCR2,BSG,HMGB1,IL6,CD44,TNF,SLC16A1,VEGFA |
| GO:0120036 | Plasma membrane bounded cell projection organization | 21 | 1122 | 7.71E-05 | MAPK1,CDH1,PIK3CA,RB1,IGF1R,ERBB2,EGFR,NOTCH1,PAK1,USP9X,STK11,BSG,CTNNB1,HMGB1,MTOR,PTEN,POSTN,JAK2,BCL2,IL6,VEGFA |
| GO:0001701 | In utero embryonic development | 12 | 382 | 7.79E-05 | MAPK1,EPAS1,TP53,EGFR,NOTCH1,CTNNB1,CASP8,AR,KDM4C,ERCC2,HIF1A,VEGFA |
| GO:1902106 | Negative regulation of leukocyte differentiation | 7 | 104 | 7.79E-05 | BMP4,ERBB2,CTNNB1,HMGB1,TLR4,RUNX3,CDKN2A |
| GO:0019752 | Carboxylic acid metabolic process | 18 | 853 | 8.06E-05 | TGFB1,HGF,ALDH1A2,NOX4,GSTM1,TYMS,NOS2,BSG,PTGS2,ABCC2,UGT1A1,GSTP1,ABCC1,CD44,BRCA1,HIF1A,SLC16A1,CYP1B1 |
| GO:0021537 | Telencephalon development | 10 | 257 | 8.17E-05 | BMP4,XRCC1,EGFR,BAX,EZH2,RARB,CTNNB1,PTEN,H2AFX,HIF1A |
| GO:2000027 | Regulation of animal organ morphogenesis | 10 | 257 | 8.17E-05 | HGF,BMP4,NOTCH1,BAX,CTNNB1,STAT1,AR,BCL2,TNF,VEGFA |
| GO:0002822 | Regulation of adaptive immune response based on somatic recombination of immune receptors built from immunoglobulin superfamily domains | 8 | 150 | 8.28E-05 | TGFB1,MLH1,HMGB1,CD274,TP53BP1,HSPD1,IL6,TNF |
| GO:0048592 | Eye morphogenesis | 8 | 151 | 8.61E-05 | BMP4,STAT3,BAX,RARB,CTNNB1,FASLG,HIF1A,VEGFA |
| GO:0048640 | Negative regulation of developmental growth | 7 | 106 | 8.61E-05 | BMP4,GDF15,PAK1,PTEN,TP73,SPP1,CDKN1A |
| GO:0051770 | Positive regulation of nitric-oxide synthase biosynthetic process | 4 | 16 | 8.61E-05 | IFNG,STAT1,TLR4,JAK2 |
| GO:0070102 | interleukin-6-mediated signaling pathway | 4 | 16 | 8.61E-05 | STAT3,STAT1,JAK2,IL6 |
| GO:1900119 | Positive regulation of execution phase of apoptosis | 4 | 16 | 8.61E-05 | SIRT2,TP53,BAX,IL6 |
| GO:0072091 | Regulation of stem cell proliferation | 6 | 68 | 8.65E-05 | TP53,NOTCH1,TERT,CTNNB1,HIF1A,VEGFA |
| GO:0032680 | Regulation of tumor necrosis factor production | 8 | 152 | 8.97E-05 | IFNG,AXL,HMGB1,TLR4,JAK2,HSPD1,GSTP1,ZFP36 |
| GO:0014823 | Response to activity | 6 | 69 | 9.32E-05 | PRKDC,MTOR,PTEN,POSTN,HSPD1,HIF1A |
| GO:0009267 | Cellular response to starvation | 8 | 154 | 9.81E-05 | MAPK1,TP53,HSPA5,FAS,MTOR,NFE2L2,BCL2,CDKN1A |
| GO:0030902 | Hindbrain development | 8 | 154 | 9.81E-05 | ALDH1A2,XRCC1,TP53,PAK1,EZH2,HSPA5,CTNNB1,BCL2 |
| GO:0032733 | Positive regulation of interleukin-10 production | 5 | 39 | 9.99E-05 | HGF,HMGB1,TLR4,CD274,HSPD1 |
| GO:0001503 | Ossification | 10 | 265 | 0.0001 | TGFB1,BMP4,EGFR,SOX2,PTGS2,IGFBP3,ERCC2,SPP1,BCL2,RUNX3 |
| GO:0001837 | Epithelial to mesenchymal transition | 6 | 70 | 0.0001 | TGFB1,HGF,NOTCH1,CTNNB1,S100A4,HIF1A |
| GO:0002637 | Regulation of immunoglobulin production | 6 | 70 | 0.0001 | TGFB1,MLH1,PRKDC,TP53BP1,IL6,TNF |
| GO:0008015 | Blood circulation | 12 | 394 | 0.0001 | TGFB1,PIK3CA,EGFR,CXCR2,NOS2,STAT1,MTOR,PTGS2,AR,POSTN,SOD2,VEGFA |
| GO:0010639 | Negative regulation of organelle organization | 12 | 395 | 0.0001 | ERCC1,HGF,BMP4,XRCC1,TP53,BIRC5,ERCC4,MET,KDM4C,XRCC5,TOP2A,BRCA1 |
| GO:0035051 | Cardiocyte differentiation | 7 | 109 | 0.0001 | MAPK1,BMP4,NOX4,NOTCH1,RARB,MTOR,VEGFA |
| GO:0038127 | ERBB signaling pathway | 6 | 70 | 0.0001 | MAPK1,TGFB1,PIK3CA,ERBB2,EGFR,ADAM17 |
| GO:0070373 | Negative regulation of erk1 and erk2 cascade | 6 | 70 | 0.0001 | DUSP1,TIMP3,PTEN,TLR4,SIRT3,GSTP1 |
| GO:2000573 | Positive regulation of dna biosynthetic process | 6 | 70 | 0.0001 | MAPK1,HGF,NOX4,CTNNB1,XRCC5,VEGFA |
| GO:0001816 | Cytokine production | 8 | 158 | 0.00011 | NOTCH1,NOS2,HMGB1,PTGS2,TLR4,HSPD1,TNF,HIF1A |
| GO:0002685 | Regulation of leukocyte migration | 9 | 209 | 0.00011 | MAPK1,SERPINE1,DUSP1,ADAM17,CXCR2,HMGB1,IL6,TNF,VEGFA |
| GO:0002703 | Regulation of leukocyte mediated immunity | 9 | 209 | 0.00011 | TGFB1,MLH1,NOS2,HMGB1,TLR4,TP53BP1,HSPD1,IL6,TNF |
| GO:0006289 | Nucleotide-excision repair | 7 | 111 | 0.00011 | ERCC1,XRCC1,TP53,OGG1,ERCC4,ERCC5,ERCC2 |
| GO:0033683 | Nucleotide-excision repair, dna incision | 5 | 40 | 0.00011 | ERCC1,OGG1,ERCC4,ERCC5,ERCC2 |
| GO:0045685 | Regulation of glial cell differentiation | 6 | 71 | 0.00011 | TGFB1,NOTCH1,CTNNB1,MTOR,TP73,IL6 |
| GO:0048863 | Stem cell differentiation | 8 | 156 | 0.00011 | MAPK1,ALDH1A2,TP63,TP53,NOTCH1,ERCC2,XRCC5,HIF1A |
| GO:0051341 | Regulation of oxidoreductase activity | 7 | 111 | 0.00011 | IFNG,EGFR,TERT,SIRT3,TNF,PDK2,HIF1A |
| GO:0060574 | Intestinal epithelial cell maturation | 3 | 4 | 0.00011 | TYMS,CDKN1A,HIF1A |
| GO:0090100 | Positive regulation of transmembrane receptor protein serine/threonine kinase signaling pathway | 7 | 110 | 0.00011 | TGFB1,BMP4,GDF15,NOTCH1,ADAM17,STK11,JAK2 |
| GO:0097296 | Activation of cysteine-type endopeptidase activity involved in apoptotic signaling pathway | 3 | 4 | 0.00011 | BAX,CASP8,JAK2 |
| GO:0010810 | Regulation of cell-substrate adhesion | 9 | 212 | 0.00012 | LGALS1,SERPINE1,NOTCH1,PTEN,POSTN,JAK2,BCL2,CDKN2A,VEGFA |
| GO:0031396 | Regulation of protein ubiquitination | 9 | 212 | 0.00012 | HSPA5,MTA1,MTOR,XIAP,PTEN,BMI1,CDKN2A,BRCA1,BIRC2 |
| GO:0050708 | Regulation of protein secretion | 11 | 333 | 0.00012 | TGFB1,IFNG,EGFR,NOS2,TLR4,JAK2,SIRT3,IL6,TNF,HIF1A,SLC16A1 |
| GO:0051091 | Positive regulation of dna-binding transcription factor activity | 10 | 271 | 0.00012 | STAT3,CTNNB1,PTEN,TLR4,AR,JAK2,TP53BP1,IL6,TNF,VEGFA |
| GO:0060571 | Morphogenesis of an epithelial fold | 4 | 18 | 0.00012 | TP63,EGFR,AR,HIF1A |
| GO:0072593 | Reactive oxygen species metabolic process | 7 | 112 | 0.00012 | NOX4,EGFR,NOS2,GPX3,BCL2,SOD2,CYP1B1 |
| GO:1900117 | Regulation of execution phase of apoptosis | 5 | 41 | 0.00012 | SIRT2,TP53,BAX,IL6,CDKN2A |
| GO:1904706 | Negative regulation of vascular associated smooth muscle cell proliferation | 4 | 18 | 0.00012 | PTEN,GSTP1,CDKN1A,SOD2 |
| GO:2000811 | Negative regulation of anoikis | 4 | 18 | 0.00012 | PIK3CA,NOTCH1,MCL1,BCL2 |
| GO:0001942 | Hair follicle development | 6 | 74 | 0.00013 | TP63,EGFR,NOTCH1,CTNNB1,ERCC2,BCL2 |
| GO:0006325 | Chromatin organization | 16 | 713 | 0.00013 | SIRT2,TP63,RB1,TP53,PAK1,EZH2,SOX2,MTA1,CTNNB1,HMGB1,BMI1,KDM4C,JAK2,SIRT3,CDKN2A,H2AFX |
| GO:0006367 | Transcription initiation from rna polymerase ii promoter | 8 | 162 | 0.00013 | CCND1,NOTCH1,BAX,RARB,PTEN,AR,ERCC2,CDKN1A |
| GO:0043627 | Response to estrogen | 6 | 74 | 0.00013 | MAPK1,CCND1,MDM2,ABCC2,HSPD1,BRCA1 |
| GO:0071346 | Cellular response to interferon-gamma | 8 | 161 | 0.00013 | IFNG,TP53,NOS2,STAT1,FASLG,TLR4,JAK2,CD44 |
| GO:0006109 | Regulation of carbohydrate metabolic process | 9 | 216 | 0.00014 | TGFB1,IFNG,STAT3,TP53,MTOR,UGT1A1,IGFBP3,PDK2,HIF1A |
| GO:0019725 | Cellular homeostasis | 18 | 895 | 0.00014 | MAPK1,LGALS1,APEX1,NOX4,PIK3CA,IGF1R,BAX,CXCR2,NOS2,HMGB1,FASLG,MCL1,ABCC2,JAK2,NFE2L2,BCL2,IL6,HIF1A |
| GO:0044281 | Small molecule metabolic process | 26 | 1684 | 0.00014 | MAPK1,TGFB1,HGF,ALDH1A2,NOX4,PIK3CA,ALDH1A1,OGG1,GSTM1,TYMS,NOS2,BSG,MTOR,PTGS2,ABCC2,PTEN,UGT1A1,GSTP1,ABCC1,CD44,TNF,BRCA1,PDK2,HIF1A,SLC16A1,CYP1B1 |
| GO:0022607 | Cellular component assembly | 32 | 2359 | 0.00015 | ERCC1,TGFB1,MLH1,SIRT2,ALDH1A2,MDM2,CDH1,TP63,TP53,NOTCH1,BIRC5,TERT,PRKDC,CTNNB1,ERCC5,FAS,CASP8,PTEN,TP73,JAK2,TP53BP1,RAD51,HSPD1,ERCC2,XRCC5,YWHAZ,BCL2,TNF,CDKN2A,H2AFX,SOD2,XRCC3 |
| GO:0042752 | Regulation of circadian rhythm | 7 | 117 | 0.00015 | TP53,PRKDC,USP9X,EZH2,MTA1,MTOR,TOP2A |
| GO:0045667 | Regulation of osteoblast differentiation | 7 | 117 | 0.00015 | HGF,BMP4,TP63,NOTCH1,CTNNB1,IL6,TNF |
| GO:0060749 | Mammary gland alveolus development | 4 | 19 | 0.00015 | CCND1,AR,HIF1A,VEGFA |
| GO:0070230 | Positive regulation of lymphocyte apoptotic process | 4 | 19 | 0.00015 | TP53,BAX,PDCD1,CD274 |
| GO:0070317 | Negative regulation of g0 to g1 transition | 5 | 43 | 0.00015 | EZH2,E2F1,BMI1,RAD51,BRCA1 |
| GO:0071466 | Cellular response to xenobiotic stimulus | 7 | 117 | 0.00015 | RB1,GSTM1,E2F1,ABCC2,UGT1A1,GSTP1,CYP1B1 |
| GO:0098542 | Defense response to other organism | 18 | 900 | 0.00015 | SERPINE1,IFNG,SIRT2,TP53,AXL,ADAM17,PRKDC,NOS2,HMGB1,STAT1,FASLG,TLR4,JAK2,XRCC5,BCL2,IL6,CD44,TNF |
| GO:2000648 | Positive regulation of stem cell proliferation | 5 | 43 | 0.00015 | NOTCH1,TERT,CTNNB1,HIF1A,VEGFA |
| GO:2000772 | Regulation of cellular senescence | 5 | 43 | 0.00015 | TP63,TP53,TERT,PRKDC,CDKN2A |
| GO:0014706 | Striated muscle tissue development | 10 | 280 | 0.00016 | TGFB1,BMP4,ALDH1A2,NOX4,RB1,NOTCH1,RARB,MTOR,PTEN,VEGFA |
| GO:0033088 | Negative regulation of immature t cell proliferation in thymus | 3 | 5 | 0.00016 | BMP4,ERBB2,CDKN2A |
| GO:0034103 | Regulation of tissue remodeling | 6 | 77 | 0.00016 | TGFB1,TP53,EGFR,BAX,SPP1,IL6 |
| GO:0043401 | Steroid hormone mediated signaling pathway | 7 | 118 | 0.00016 | BMP4,RB1,RARB,CTNNB1,AR,JAK2,BRCA1 |
| GO:0046677 | Response to antibiotic | 5 | 44 | 0.00016 | MDM2,TP53,EZH2,HSPA5,JAK2 |
| GO:0051106 | Positive regulation of dna ligation | 3 | 5 | 0.00016 | XRCC1,HMGB1,RAD51 |
| GO:0055025 | Positive regulation of cardiac muscle tissue development | 5 | 44 | 0.00016 | MAPK1,TGFB1,BMP4,NOTCH1,MTOR |
| GO:0071681 | Cellular response to indole-3-methanol | 3 | 5 | 0.00016 | CDH1,CTNNB1,BRCA1 |
| GO:0090287 | Regulation of cellular response to growth factor stimulus | 10 | 280 | 0.00016 | TGFB1,BMP4,TP53,NOTCH1,ADAM17,HSPA5,STK11,CTNNB1,XIAP,HIF1A |
| GO:2000241 | Regulation of reproductive process | 8 | 166 | 0.00016 | DUSP1,BMP4,SIRT2,NOTCH1,STK11,CTNNB1,AR,VEGFA |
| GO:2000736 | Regulation of stem cell differentiation | 7 | 119 | 0.00016 | BMP4,STAT3,NOTCH1,PRKDC,TP73,KDM4C,NFE2L2 |
| GO:0002312 | B cell activation involved in immune response | 5 | 45 | 0.00017 | ERCC1,LGALS1,MLH1,TLR4,HSPD1 |
| GO:0002699 | Positive regulation of immune effector process | 9 | 223 | 0.00017 | TGFB1,IFNG,MLH1,NOS2,TLR4,TP53BP1,HSPD1,IL6,TNF |
| GO:0032869 | Cellular response to insulin stimulus | 8 | 169 | 0.00017 | PIK3CA,IGF1R,PAK1,PRKDC,STAT1,PTEN,GSTP1,PDK2 |
| GO:0043409 | Negative regulation of mapk cascade | 8 | 168 | 0.00017 | DUSP1,BMP4,TIMP3,IGF1R,PTEN,TLR4,SIRT3,GSTP1 |
| GO:0045581 | Negative regulation of t cell differentiation | 5 | 45 | 0.00017 | BMP4,ERBB2,HMGB1,RUNX3,CDKN2A |
| GO:0051641 | Cellular localization | 37 | 2967 | 0.00017 | MAPK1,TGFB1,HGF,SERPINE1,MLH1,BMP4,MDM2,CDH1,STAT3,TIMP3,RB1,TP53,EGFR,BAX,BIRC5,TERT,USP9X,CXCR2,EZH2,HSPA5,STK11,NOS2,BSG,CTNNB1,HMGB1,MTOR,FASLG,HSPD1,XRCC5,YWHAZ,GSTP1,CDKN1A,CD44,TNF,EIF4E,HIF1A,VEGFA |
| GO:0061448 | Connective tissue development | 9 | 223 | 0.00017 | TGFB1,BMP4,PIK3CA,NOTCH1,TYMS,RARB,RUNX3,CD44,HIF1A |
| GO:1900409 | Positive regulation of cellular response to oxidative stress | 4 | 20 | 0.00017 | MCL1,TLR4,SIRT3,TNF |
| GO:0071260 | Cellular response to mechanical stimulus | 6 | 79 | 0.00018 | TNFRSF10A,EGFR,FAS,CASP8,PTGS2,TLR4 |
| GO:0009056 | Catabolic process | 29 | 2042 | 0.00019 | CSNK2A1,TGFB1,MLH1,SIRT2,MDM2,TP53,ALDH1A1,OGG1,GSTM1,USP9X,HSPA5,STK11,NOS2,MTA1,CTNNB1,HMGB1,CASP8,MTOR,ABCC2,PTEN,UGT1A1,GPX3,SPP1,NFE2L2,CD44,CDKN2A,ZFP36,BIRC2,CYP1B1 |
| GO:0010608 | Posttranscriptional regulation of gene expression | 14 | 574 | 0.00019 | MAPK1,APEX1,STAT3,ERBB2,TERT,PRKDC,TYMS,E2F1,MTOR,YWHAZ,IL6,TNF,EIF4E,ZFP36 |
| GO:0021543 | Pallium development | 8 | 171 | 0.00019 | XRCC1,EGFR,BAX,EZH2,CTNNB1,PTEN,H2AFX,HIF1A |
| GO:0030307 | Positive regulation of cell growth | 8 | 171 | 0.00019 | CSNK2A1,ERBB2,EGFR,PAK1,ADAM17,MTOR,BCL2,VEGFA |
| GO:0034401 | Chromatin organization involved in regulation of transcription | 7 | 122 | 0.00019 | SIRT2,EZH2,CTNNB1,HMGB1,BMI1,CDKN2A,H2AFX |
| GO:1900015 | Regulation of cytokine production involved in inflammatory response | 5 | 46 | 0.00019 | STAT3,NOS2,TLR4,IL6,TNF |
| GO:0006293 | Nucleotide-excision repair, preincision complex stabilization | 4 | 21 | 0.0002 | ERCC1,ERCC4,ERCC5,ERCC2 |
| GO:0042475 | Odontogenesis of dentin-containing tooth | 6 | 81 | 0.0002 | SERPINE1,BMP4,TP63,BAX,BSG,CTNNB1 |
| GO:1900017 | Positive regulation of cytokine production involved in inflammatory response | 4 | 21 | 0.0002 | STAT3,TLR4,IL6,TNF |
| GO:0006970 | Response to osmotic stress | 6 | 82 | 0.00021 | TP53,EGFR,BAX,PTGS2,XRCC5,TNF |
| GO:0009743 | Response to carbohydrate | 8 | 174 | 0.00021 | ERCC1,LGALS1,NOX4,PIK3CA,IGF1R,PTGS2,PTEN,GSTP1 |
| GO:0030218 | Erythrocyte differentiation | 6 | 82 | 0.00021 | BMP4,EPAS1,RB1,JAK2,ERCC2,VEGFA |
| GO:0033209 | Tumor necrosis factor-mediated signaling pathway | 7 | 125 | 0.00021 | TP53,ADAM17,FAS,STAT1,JAK2,TNF,BIRC2 |
| GO:0070849 | Response to epidermal growth factor | 5 | 47 | 0.00021 | MAPK1,ERBB2,EGFR,GSTP1,ZFP36 |
| GO:0098754 | Detoxification | 7 | 125 | 0.00021 | GSTM1,PTGS2,ABCC2,GPX3,NFE2L2,GSTP1,SOD2 |
| GO:0030879 | Mammary gland development | 7 | 126 | 0.00022 | MAPK1,CCND1,BMP4,AR,JAK2,HIF1A,VEGFA |
| GO:0031398 | Positive regulation of protein ubiquitination | 7 | 126 | 0.00022 | HSPA5,MTA1,XIAP,PTEN,BMI1,BRCA1,BIRC2 |
| GO:0032204 | Regulation of telomere maintenance | 6 | 83 | 0.00022 | ERCC1,MAPK1,XRCC1,ERCC4,CTNNB1,XRCC5 |
| GO:0001836 | Release of cytochrome c from mitochondria | 4 | 22 | 0.00023 | TP53,BAX,BCL2,SOD2 |
| GO:0002326 | B cell lineage commitment | 3 | 6 | 0.00023 | TP53,PRKDC,BCL2 |
| GO:0006295 | Nucleotide-excision repair, dna incision, 3-to lesion | 4 | 22 | 0.00023 | ERCC1,ERCC4,ERCC5,ERCC2 |
| GO:0030326 | Embryonic limb morphogenesis | 7 | 127 | 0.00023 | BMP4,ALDH1A2,TP63,NOTCH1,BAX,RARB,CTNNB1 |
| GO:0045599 | Negative regulation of fat cell differentiation | 5 | 48 | 0.00023 | TGFB1,SIRT2,E2F1,IL6,TNF |
| GO:0048806 | Genitalia development | 5 | 48 | 0.00023 | TP63,BAX,AXL,CTNNB1,AR |
| GO:0060440 | Trachea formation | 3 | 6 | 0.00023 | MAPK1,BMP4,CTNNB1 |
| GO:0060740 | Prostate gland epithelium morphogenesis | 4 | 22 | 0.00023 | BMP4,TP63,NOTCH1,AR |
| GO:0061046 | Regulation of branching involved in lung morphogenesis | 3 | 6 | 0.00023 | BMP4,CTNNB1,TNF |
| GO:0000082 | G1/S transition of mitotic cell cycle | 7 | 128 | 0.00024 | CCND1,RB1,TYMS,E2F1,CDKN1A,CDKN2A,EIF4E |
| GO:0003206 | Cardiac chamber morphogenesis | 7 | 128 | 0.00024 | TGFB1,BMP4,MDM2,TP53,NOTCH1,RARB,HIF1A |
| GO:0045619 | Regulation of lymphocyte differentiation | 8 | 178 | 0.00024 | IFNG,BMP4,ERBB2,AXL,PRKDC,HMGB1,RUNX3,CDKN2A |
| GO:0006469 | Negative regulation of protein kinase activity | 9 | 236 | 0.00025 | IFNG,DUSP1,BMP4,RB1,IGF1R,PTEN,GSTP1,CDKN1A,CDKN2A |
| GO:0019748 | Secondary metabolic process | 5 | 49 | 0.00025 | ABCC2,UGT1A1,NFE2L2,BCL2,CYP1B1 |
| GO:0045646 | Regulation of erythrocyte differentiation | 5 | 49 | 0.00025 | STAT3,PRKDC,STAT1,HIF1A,ZFP36 |
| GO:1905207 | Regulation of cardiocyte differentiation | 5 | 49 | 0.00025 | TGFB1,BMP4,EGFR,PAK1,MTOR |
| GO:0002052 | Positive regulation of neuroblast proliferation | 4 | 23 | 0.00026 | NOTCH1,CTNNB1,HIF1A,VEGFA |
| GO:0009651 | Response to salt stress | 4 | 23 | 0.00026 | TP53,BAX,XRCC5,TNF |
| GO:0035264 | Multicellular organism growth | 6 | 86 | 0.00026 | ERCC1,TP53,RARB,MTOR,AR,ERCC2 |
| GO:1904752 | Regulation of vascular associated smooth muscle cell migration | 4 | 23 | 0.00026 | MDM2,PAK1,TERT,NFE2L2 |
| GO:2000351 | Regulation of endothelial cell apoptotic process | 5 | 50 | 0.00027 | SERPINE1,TERT,FASLG,NFE2L2,TNF |
| GO:0007565 | Female pregnancy | 8 | 183 | 0.00028 | MAPK1,BSG,MTOR,PTGS2,ABCC2,AR,SPP1,BCL2 |
| GO:0051234 | Establishment of localization | 48 | 4479 | 0.00028 | MAPK1,TGFB1,HGF,SERPINE1,CCND1,MLH1,MDM2,PIK3CA,STAT3,TP63,TIMP3,IGF1R,TP53,EGFR,PAK1,BAX,AXL,BIRC5,TERT,USP9X,CXCR2,HSPA5,NOS2,CTNNB1,HMGB1,E2F1,CASP8,FASLG,MCL1,ABCC2,ABCC10,TLR4,TP73,CD274,HSPD1,XRCC5,YWHAZ,BCL2,GSTP1,ABCC1,CDKN1A,IL6,CD44,EIF4E,HIF1A,SLC16A1,ZFP36,VEGFA |
| GO:0051972 | Regulation of telomerase activity | 5 | 51 | 0.00029 | MAPK1,TP53,ERCC4,CTNNB1,XRCC5 |
| GO:1901343 | Negative regulation of vasculature development | 7 | 132 | 0.00029 | SERPINE1,BMP4,NOTCH1,CTNNB1,STAT1,FASLG,TNF |
| GO:0045778 | Positive regulation of ossification | 6 | 88 | 0.0003 | TGFB1,HGF,BMP4,TP63,CTNNB1,IL6 |
| GO:0061311 | Cell surface receptor signaling pathway involved in heart development | 4 | 24 | 0.0003 | TGFB1,BMP4,NOTCH1,CTNNB1 |
| GO:1902254 | Negative regulation of intrinsic apoptotic signaling pathway by p53 class mediator | 4 | 24 | 0.0003 | MDM2,BCL2,CD44,MUC1 |
| GO:2000177 | Regulation of neural precursor cell proliferation | 6 | 88 | 0.0003 | SIRT2,TP53,NOTCH1,CTNNB1,HIF1A,VEGFA |
| GO:0048146 | Positive regulation of fibroblast proliferation | 5 | 52 | 0.00031 | EGFR,PRKDC,E2F1,BMI1,CDKN1A |
| GO:0097306 | Cellular response to alcohol | 6 | 89 | 0.00031 | CDH1,CTNNB1,PTEN,UGT1A1,RAD51,BRCA1 |
| GO:0110110 | Positive regulation of animal organ morphogenesis | 6 | 89 | 0.00031 | BMP4,NOTCH1,BAX,CTNNB1,AR,VEGFA |
| GO:0018193 | Peptidyl-amino acid modification | 17 | 867 | 0.00032 | MAPK1,CSNK2A1,SIRT2,MDM2,IGF1R,ERBB2,EGFR,AXL,PRKDC,MET,EZH2,NOS2,MTOR,JAK2,SIRT3,BCL2,CDKN2A |
| GO:0007131 | Reciprocal meiotic recombination | 5 | 53 | 0.00034 | MLH1,ERCC4,RAD51C,RAD51,TOP2A |
| GO:0016447 | Somatic recombination of immunoglobulin gene segments | 4 | 25 | 0.00034 | ERCC1,MLH1,PRKDC,HSPD1 |
| GO:0046632 | Alpha-beta t cell differentiation | 5 | 53 | 0.00034 | STAT3,HMGB1,MTOR,BCL2,IL6 |
| GO:0046697 | Decidualization | 4 | 25 | 0.00034 | MAPK1,BSG,PTGS2,SPP1 |
| GO:0050918 | Positive chemotaxis | 5 | 53 | 0.00034 | HGF,BMP4,MET,HMGB1,VEGFA |
| GO:0071675 | Regulation of mononuclear cell migration | 5 | 53 | 0.00034 | MAPK1,SERPINE1,DUSP1,HMGB1,TNF |
| GO:0071901 | Negative regulation of protein serine/threonine kinase activity | 7 | 136 | 0.00034 | DUSP1,BMP4,RB1,PTEN,GSTP1,CDKN1A,CDKN2A |
| GO:0002705 | Positive regulation of leukocyte mediated immunity | 7 | 137 | 0.00035 | TGFB1,MLH1,NOS2,TP53BP1,HSPD1,IL6,TNF |
| GO:0018130 | Heterocycle biosynthetic process | 19 | 1061 | 0.00035 | MAPK1,TGFB1,CCND1,EPAS1,STAT3,TP53,NOTCH1,BAX,OGG1,TERT,TYMS,RARB,E2F1,MTOR,PTGS2,PTEN,AR,ERCC2,CDKN1A |
| GO:0098727 | Maintenance of cell number | 7 | 137 | 0.00035 | STAT3,NOTCH1,EZH2,SOX2,CTNNB1,KDM4C,EIF4E |
| GO:1903532 | Positive regulation of secretion by cell | 10 | 312 | 0.00035 | TGFB1,IFNG,EGFR,TLR4,JAK2,SIRT3,SPP1,IL6,TNF,HIF1A |
| GO:0002066 | Columnar/cuboidal epithelial cell development | 5 | 54 | 0.00036 | BMP4,TYMS,RARB,CDKN1A,HIF1A |
| GO:0006342 | Chromatin silencing | 5 | 54 | 0.00036 | SIRT2,EZH2,HMGB1,BMI1,H2AFX |
| GO:0002253 | Activation of immune response | 11 | 385 | 0.00038 | MAPK1,PIK3CA,PAK1,BAX,PRKDC,STK11,HMGB1,TLR4,XRCC5,BCL2,MUC1 |
| GO:0030225 | Macrophage differentiation | 4 | 26 | 0.00039 | IFNG,BMP4,CASP8,VEGFA |
| GO:0031295 | T cell costimulation | 5 | 55 | 0.00039 | LGALS1,PIK3CA,PAK1,PDCD1,CD274 |
| GO:0032727 | Positive regulation of interferon-alpha production | 4 | 26 | 0.00039 | HMGB1,STAT1,TLR4,HSPD1 |
| GO:0045661 | Regulation of myoblast differentiation | 5 | 55 | 0.00039 | TGFB1,BMP4,NOTCH1,IGFBP3,TNF |
| GO:0071887 | Leukocyte apoptotic process | 4 | 26 | 0.00039 | BAX,FAS,FASLG,IL6 |
| GO:0090344 | Negative regulation of cell aging | 4 | 26 | 0.00039 | TP63,TERT,PRKDC,PTEN |
| GO:1904353 | Regulation of telomere capping | 4 | 26 | 0.00039 | ERCC1,MAPK1,XRCC1,ERCC4 |
| GO:0035635 | Entry of bacterium into host cell | 3 | 8 | 0.00042 | CDH1,MET,CTNNB1 |
| GO:0097527 | Necroptotic signaling pathway | 3 | 8 | 0.00042 | FAS,FASLG,TNF |
| GO:0007623 | Circadian rhythm | 7 | 142 | 0.00043 | SERPINE1,TP53,EGFR,TYMS,NOS2,MTA1,PTEN |
| GO:0006935 | Chemotaxis | 13 | 545 | 0.00044 | MAPK1,HGF,BMP4,PIK3CA,ERBB2,NOTCH1,MET,CXCR2,BSG,HMGB1,ABCC1,IL6,VEGFA |
| GO:0045662 | Negative regulation of myoblast differentiation | 4 | 27 | 0.00044 | TGFB1,BMP4,NOTCH1,TNF |
| GO:1902175 | Regulation of oxidative stress-induced intrinsic apoptotic signaling pathway | 4 | 27 | 0.00044 | MCL1,NFE2L2,HIF1A,SOD2 |
| GO:2000679 | Positive regulation of transcription regulatory region dna binding | 4 | 27 | 0.00044 | TGFB1,IFNG,RB1,CTNNB1 |
| GO:0010803 | Regulation of tumor necrosis factor-mediated signaling pathway | 5 | 57 | 0.00045 | ADAM17,CASP8,GSTP1,TNF,BIRC2 |
| GO:0032768 | Regulation of monooxygenase activity | 5 | 57 | 0.00045 | IFNG,EGFR,TERT,TNF,HIF1A |
| GO:0051353 | Positive regulation of oxidoreductase activity | 5 | 57 | 0.00045 | IFNG,TERT,SIRT3,TNF,HIF1A |
| GO:0061180 | Mammary gland epithelium development | 5 | 57 | 0.00045 | MAPK1,CCND1,AR,JAK2,HIF1A |
| GO:0023014 | Signal transduction by protein phosphorylation | 11 | 395 | 0.00046 | MAPK1,TGFB1,HGF,ERBB2,EGFR,PAK1,MET,JAK2,YWHAZ,TNF,ZFP36 |
| GO:0000079 | Regulation of cyclin-dependent protein serine/threonine kinase activity | 6 | 97 | 0.00047 | CCND1,EGFR,ADAM17,PTEN,CDKN1A,CDKN2A |
| GO:0002763 | Positive regulation of myeloid leukocyte differentiation | 5 | 58 | 0.00049 | TGFB1,IFNG,RB1,CASP8,TNF |
| GO:0003180 | Aortic valve morphogenesis | 4 | 28 | 0.00049 | TGFB1,BMP4,RB1,NOTCH1 |
| GO:0031069 | Hair follicle morphogenesis | 4 | 28 | 0.00049 | TP63,NOTCH1,CTNNB1,BCL2 |
| GO:0034654 | Nucleobase-containing compound biosynthetic process | 18 | 995 | 0.00049 | MAPK1,TGFB1,CCND1,EPAS1,STAT3,TP53,NOTCH1,BAX,OGG1,TERT,TYMS,RARB,E2F1,PTGS2,PTEN,AR,ERCC2,CDKN1A |
| GO:0042093 | T-helper cell differentiation | 4 | 28 | 0.00049 | STAT3,HMGB1,MTOR,IL6 |
| GO:1903203 | Regulation of oxidative stress-induced neuron death | 4 | 28 | 0.00049 | CTNNB1,MCL1,TLR4,HIF1A |
| GO:0065003 | Protein-containing complex assembly | 21 | 1293 | 0.0005 | TGFB1,ALDH1A2,MDM2,TP63,TP53,TERT,PRKDC,CTNNB1,ERCC5,FAS,CASP8,TP73,JAK2,TP53BP1,RAD51,HSPD1,ERCC2,XRCC5,TNF,H2AFX,SOD2 |
| GO:0070665 | Positive regulation of leukocyte proliferation | 7 | 146 | 0.0005 | MAPK1,HMGB1,TLR4,CD274,BCL2,CDKN1A,IL6 |
| GO:0003008 | System process | 27 | 1942 | 0.00051 | MAPK1,TGFB1,BMP4,NOX4,EPAS1,PIK3CA,TIMP3,EGFR,BIRC5,CXCR2,EZH2,NOS2,STAT1,MTOR,PTGS2,ABCC2,PTEN,TLR4,AR,POSTN,YWHAZ,BCL2,TNF,HIF1A,SOD2,CYP1B1,VEGFA |
| GO:0035148 | Tube formation | 7 | 147 | 0.00052 | TGFB1,BMP4,NOTCH1,CTNNB1,YWHAZ,HIF1A,VEGFA |
| GO:0040029 | Regulation of gene expression, epigenetic | 8 | 202 | 0.00052 | SIRT2,EZH2,MTA1,CTNNB1,HMGB1,BMI1,BRCA1,H2AFX |
| GO:0051155 | Positive regulation of striated muscle cell differentiation | 5 | 59 | 0.00052 | TGFB1,BMP4,GDF15,MTOR,BCL2 |
| GO:0090596 | Sensory organ morphogenesis | 9 | 264 | 0.00053 | MAPK1,BMP4,STAT3,BAX,RARB,CTNNB1,FASLG,HIF1A,VEGFA |
| GO:0034284 | Response to monosaccharide | 7 | 148 | 0.00054 | LGALS1,NOX4,PIK3CA,IGF1R,PTGS2,PTEN,GSTP1 |
| GO:0072217 | Negative regulation of metanephros development | 3 | 9 | 0.00054 | BMP4,CTNNB1,STAT1 |
| GO:0032660 | Regulation of interleukin-17 production | 4 | 29 | 0.00055 | TGFB1,IFNG,TLR4,IL6 |
| GO:0045669 | Positive regulation of osteoblast differentiation | 5 | 60 | 0.00055 | HGF,BMP4,TP63,CTNNB1,IL6 |
| GO:0008406 | Gonad development | 8 | 205 | 0.00057 | ERCC1,CCND1,TNFSF10,BAX,HSPA5,AR,BCL2,VEGFA |
| GO:0007059 | Chromosome segregation | 9 | 268 | 0.00059 | MLH1,RB1,BIRC5,ERCC4,USP9X,RAD51C,ERCC2,TOP2A,BRCA1 |
| GO:0009058 | Biosynthetic process | 34 | 2788 | 0.00059 | MAPK1,CSNK2A1,TGFB1,CCND1,ALDH1A2,NOX4,EPAS1,PIK3CA,STAT3,TP53,EGFR,NOTCH1,BAX,OGG1,TERT,GSTM1,TYMS,NOS2,RARB,E2F1,MTOR,PTGS2,PTEN,AR,RAD51,ERCC2,GSTP1,CDKN1A,TNF,BRCA1,EIF4E,HIF1A,CYP1B1,MUC1 |
| GO:0046883 | Regulation of hormone secretion | 9 | 268 | 0.00059 | IFNG,EGFR,NOS2,JAK2,SIRT3,SPP1,TNF,HIF1A,SLC16A1 |
| GO:0044271 | Cellular nitrogen compound biosynthetic process | 23 | 1522 | 0.0006 | MAPK1,TGFB1,CCND1,EPAS1,STAT3,TP53,EGFR,NOTCH1,BAX,OGG1,TERT,TYMS,NOS2,RARB,E2F1,MTOR,PTGS2,PTEN,AR,ERCC2,CDKN1A,EIF4E,CYP1B1 |
| GO:0042771 | Intrinsic apoptotic signaling pathway in response to dna damage by p53 class mediator | 4 | 30 | 0.00061 | TP63,TP53,TP73,CDKN1A |
| GO:0043457 | Regulation of cellular respiration | 4 | 30 | 0.00061 | IFNG,PIK3CA,NOS2,HIF1A |
| GO:0002700 | Regulation of production of molecular mediator of immune response | 7 | 152 | 0.00063 | TGFB1,MLH1,PRKDC,TLR4,TP53BP1,IL6,TNF |
| GO:0002706 | Regulation of lymphocyte mediated immunity | 7 | 152 | 0.00063 | TGFB1,MLH1,HMGB1,TP53BP1,HSPD1,IL6,TNF |
| GO:0030198 | Extracellular matrix organization | 10 | 338 | 0.00063 | TGFB1,SERPINE1,CDH1,BSG,POSTN,ERCC2,SPP1,CD44,TNF,CYP1B1 |
| GO:0045604 | Regulation of epidermal cell differentiation | 5 | 62 | 0.00063 | BMP4,TP63,NOTCH1,EZH2,ZFP36 |
| GO:0051250 | Negative regulation of lymphocyte activation | 7 | 152 | 0.00063 | BMP4,ERBB2,AXL,HMGB1,CD274,RUNX3,CDKN2A |
| GO:0097659 | Nucleic acid-templated transcription | 13 | 568 | 0.00063 | CCND1,EPAS1,STAT3,TP53,NOTCH1,BAX,TERT,RARB,E2F1,PTEN,AR,ERCC2,CDKN1A |
| GO:0002708 | Positive regulation of lymphocyte mediated immunity | 6 | 104 | 0.00066 | TGFB1,MLH1,TP53BP1,HSPD1,IL6,TNF |
| GO:0048598 | Embryonic morphogenesis | 13 | 571 | 0.00066 | MAPK1,TGFB1,DUSP1,BMP4,ALDH1A2,TP63,TP53,NOTCH1,BAX,SOX2,RARB,CTNNB1,HIF1A |
| GO:0007610 | Behavior | 13 | 572 | 0.00067 | MAPK1,GDF15,STAT3,TP53,EGFR,MTA1,MTOR,PTGS2,PTEN,BCL2,EIF4E,HIF1A,SLC16A1 |
| GO:0032770 | Positive regulation of monooxygenase activity | 4 | 31 | 0.00068 | IFNG,TERT,TNF,HIF1A |
| GO:0042246 | Tissue regeneration | 5 | 63 | 0.00068 | NOTCH1,EZH2,SOX2,POSTN,CDKN1A |
| GO:0046686 | Response to cadmium ion | 5 | 63 | 0.00068 | ERCC1,MAPK1,EGFR,OGG1,TERT |
| GO:0097421 | Liver regeneration | 4 | 31 | 0.00068 | CCND1,EGFR,TYMS,EZH2 |
| GO:0002295 | T-helper cell lineage commitment | 3 | 10 | 0.00069 | STAT3,MTOR,IL6 |
| GO:0010623 | Programmed cell death involved in cell development | 3 | 10 | 0.00069 | BAX,PRKDC,FASLG |
| GO:0070424 | Regulation of nucleotide-binding oligomerization domain containing signaling pathway | 3 | 10 | 0.00069 | XIAP,TLR4,BIRC2 |
| GO:1903431 | Positive regulation of cell maturation | 3 | 10 | 0.00069 | SIRT2,MTOR,BCL2 |
| GO:0009612 | Response to mechanical stimulus | 8 | 212 | 0.0007 | TNFRSF10A,EGFR,FAS,CASP8,STAT1,PTGS2,TLR4,POSTN |
| GO:0044248 | Cellular catabolic process | 25 | 1758 | 0.0007 | CSNK2A1,TGFB1,MLH1,SIRT2,MDM2,TP53,OGG1,GSTM1,USP9X,HSPA5,STK11,NOS2,MTA1,CTNNB1,HMGB1,CASP8,ABCC2,UGT1A1,GPX3,NFE2L2,CD44,CDKN2A,ZFP36,BIRC2,CYP1B1 |
| GO:0032649 | Regulation of interferon-gamma production | 6 | 106 | 0.00072 | AXL,HMGB1,TLR4,CD274,HSPD1,TNF |
| GO:0046661 | Male sex differentiation | 7 | 156 | 0.00072 | ERCC1,CCND1,TNFSF10,BAX,CTNNB1,AR,BCL2 |
| GO:0071559 | Response to transforming growth factor beta | 7 | 156 | 0.00072 | TGFB1,GDF15,NOX4,TP53,USP9X,POSTN,RUNX3 |
| GO:0001974 | Blood vessel remodeling | 4 | 32 | 0.00075 | MDM2,EPAS1,BAX,AXL |
| GO:0045648 | Positive regulation of erythrocyte differentiation | 4 | 32 | 0.00075 | STAT3,PRKDC,STAT1,HIF1A |
| GO:0051145 | Smooth muscle cell differentiation | 4 | 32 | 0.00075 | BMP4,NOTCH1,CTNNB1,VEGFA |
| GO:0090276 | Regulation of peptide hormone secretion | 8 | 215 | 0.00075 | IFNG,EGFR,NOS2,JAK2,SIRT3,TNF,HIF1A,SLC16A1 |
| GO:0061564 | Axon development | 11 | 421 | 0.00076 | MAPK1,PIK3CA,ERBB2,NOTCH1,USP9X,STK11,BSG,PTEN,JAK2,BCL2,VEGFA |
| GO:1905818 | Regulation of chromosome separation | 5 | 65 | 0.00076 | CSNK2A1,DUSP1,RB1,BIRC5,XRCC3 |
| GO:0097549 | Chromatin organization involved in negative regulation of transcription | 6 | 108 | 0.00078 | SIRT2,EZH2,HMGB1,BMI1,CDKN2A,H2AFX |
| GO:0002286 | T cell activation involved in immune response | 5 | 66 | 0.00081 | STAT3,TP53,HMGB1,MTOR,IL6 |
| GO:0010039 | Response to iron ion | 4 | 33 | 0.00082 | CCND1,MDM2,BCL2,HIF1A |
| GO:0010464 | Regulation of mesenchymal cell proliferation | 4 | 33 | 0.00082 | BMP4,CTNNB1,STAT1,VEGFA |
| GO:0090398 | Cellular senescence | 4 | 33 | 0.00082 | TP53,CDKN1A,CDKN2A,H2AFX |
| GO:0001780 | Neutrophil homeostasis | 3 | 11 | 0.00085 | AXL,HMGB1,IL6 |
| GO:0007267 | Cell-cell signaling | 19 | 1145 | 0.00085 | MAPK1,CSNK2A1,HGF,CCND1,TNFSF10,BMP4,GDF15,STAT3,TP63,STK11,NOS2,CTNNB1,HMGB1,FASLG,XIAP,PTEN,AR,JAK2,IL6 |
| GO:0030183 | B cell differentiation | 6 | 110 | 0.00085 | LGALS1,TP53,BAX,ADAM17,PRKDC,BCL2 |
| GO:0030518 | Intracellular steroid hormone receptor signaling pathway | 5 | 67 | 0.00085 | RB1,CTNNB1,AR,JAK2,BRCA1 |
| GO:0033483 | Gas homeostasis | 3 | 11 | 0.00085 | GSTP1,HIF1A,SOD2 |
| GO:0043467 | Regulation of generation of precursor metabolites and energy | 7 | 161 | 0.00085 | IFNG,PIK3CA,STAT3,TP53,NOS2,MTOR,HIF1A |
| GO:0043619 | Regulation of transcription from rna polymerase ii promoter in response to oxidative stress | 3 | 11 | 0.00085 | EPAS1,NFE2L2,HIF1A |
| GO:0045348 | Positive regulation of mhc class ii biosynthetic process | 3 | 11 | 0.00085 | IFNG,TLR4,JAK2 |
| GO:0045670 | Regulation of osteoclast differentiation | 5 | 67 | 0.00085 | IFNG,CTNNB1,MTOR,TLR4,TNF |
| GO:0046660 | Female sex differentiation | 6 | 110 | 0.00085 | TP63,BAX,AXL,HSPA5,BCL2,VEGFA |
| GO:0070106 | interleukin-27-mediated signaling pathway | 3 | 11 | 0.00085 | STAT3,STAT1,JAK2 |
| GO:0070757 | interleukin-35-mediated signaling pathway | 3 | 11 | 0.00085 | STAT3,STAT1,JAK2 |
| GO:1901222 | Regulation of nik/nf-kappab signaling | 6 | 110 | 0.00085 | TNFRSF10A,EGFR,HMGB1,TLR4,TNF,BIRC2 |
| GO:2000121 | Regulation of removal of superoxide radicals | 3 | 11 | 0.00085 | SIRT3,NFE2L2,TNF |
| GO:0051321 | Meiotic cell cycle | 8 | 220 | 0.00086 | ERCC1,MLH1,SIRT2,ERCC4,RAD51C,RAD51,TOP2A,H2AFX |
| GO:0007088 | Regulation of mitotic nuclear division | 7 | 162 | 0.00087 | DUSP1,BMP4,SIRT2,RB1,BIRC5,MKI67,XRCC3 |
| GO:0010955 | Negative regulation of protein processing | 4 | 34 | 0.0009 | SERPINE1,MDM2,XIAP,BIRC2 |
| GO:0010975 | Regulation of neuron projection development | 12 | 510 | 0.0009 | LGALS1,HGF,MDM2,PAK1,EZH2,HSPA5,STK11,MTOR,PTEN,SPP1,NFE2L2,VEGFA |
| GO:0031663 | Lipopolysaccharide-mediated signaling pathway | 4 | 34 | 0.0009 | MAPK1,TGFB1,TLR4,TNF |
| GO:0042531 | Positive regulation of tyrosine phosphorylation of stat protein | 5 | 68 | 0.0009 | IFNG,STAT3,JAK2,IL6,TNF |
| GO:0044249 | Cellular biosynthetic process | 32 | 2611 | 0.0009 | MAPK1,CSNK2A1,TGFB1,CCND1,ALDH1A2,EPAS1,PIK3CA,STAT3,TP53,EGFR,NOTCH1,BAX,OGG1,TERT,GSTM1,TYMS,NOS2,RARB,E2F1,MTOR,PTGS2,PTEN,AR,RAD51,ERCC2,GSTP1,CDKN1A,BRCA1,EIF4E,HIF1A,CYP1B1,MUC1 |
| GO:1904031 | Positive regulation of cyclin-dependent protein kinase activity | 4 | 34 | 0.0009 | CCND1,EGFR,ADAM17,CDKN1A |
| GO:0045665 | Negative regulation of neuron differentiation | 8 | 222 | 0.00091 | LGALS1,MDM2,NOTCH1,SOX2,PTEN,TP73,SPP1,EIF4E |
| GO:0071241 | Cellular response to inorganic substance | 8 | 222 | 0.00091 | MAPK1,CDH1,EGFR,OGG1,HSPA5,PTGS2,RAD51,NFE2L2 |
| GO:0002573 | Myeloid leukocyte differentiation | 6 | 113 | 0.00096 | IFNG,BMP4,CTNNB1,CASP8,TNF,VEGFA |
| GO:0010633 | Negative regulation of epithelial cell migration | 5 | 69 | 0.00096 | TGFB1,NOTCH1,HMGB1,PTEN,TNF |
| GO:0042129 | Regulation of t cell proliferation | 7 | 165 | 0.00096 | BMP4,ERBB2,CTNNB1,HMGB1,CD274,IL6,CDKN2A |
| GO:0032689 | Negative regulation of interferon-gamma production | 4 | 35 | 0.00098 | AXL,HMGB1,TLR4,CD274 |
| GO:0043516 | Regulation of dna damage response, signal transduction by p53 class mediator | 4 | 35 | 0.00098 | MDM2,TP53,CD44,CDKN2A |
| GO:0043537 | Negative regulation of blood vessel endothelial cell migration | 4 | 35 | 0.00098 | TGFB1,NOTCH1,HMGB1,TNF |
| GO:0097192 | Extrinsic apoptotic signaling pathway in absence of ligand | 4 | 35 | 0.00098 | BAX,FAS,MCL1,BCL2 |
| GO:0022037 | Metencephalon development | 6 | 115 | 0.001 | XRCC1,TP53,PAK1,EZH2,HSPA5,BCL2 |
| GO:0033629 | Negative regulation of cell adhesion mediated by integrin | 3 | 12 | 0.001 | SERPINE1,CYP1B1,MUC1 |
| GO:0060333 | Interferon-gamma-mediated signaling pathway | 5 | 70 | 0.001 | IFNG,TP53,STAT1,JAK2,CD44 |
| GO:0061982 | Meiosis i cell cycle process | 6 | 114 | 0.001 | ERCC1,MLH1,ERCC4,RAD51C,RAD51,TOP2A |
| GO:1902510 | Regulation of apoptotic dna fragmentation | 3 | 12 | 0.001 | BAX,IL6,CDKN2A |
| GO:0016525 | Negative regulation of angiogenesis | 6 | 117 | 0.0011 | SERPINE1,NOTCH1,CTNNB1,STAT1,FASLG,TNF |
| GO:0018107 | Peptidyl-threonine phosphorylation | 5 | 72 | 0.0011 | MAPK1,CSNK2A1,PRKDC,MTOR,BCL2 |
| GO:0030521 | Androgen receptor signaling pathway | 4 | 36 | 0.0011 | RB1,CTNNB1,AR,BRCA1 |
| GO:0032102 | Negative regulation of response to external stimulus | 10 | 367 | 0.0011 | HGF,SERPINE1,DUSP1,SIRT2,RB1,NOTCH1,PTEN,SPP1,GSTP1,TNF |
| GO:0050868 | Negative regulation of t cell activation | 6 | 117 | 0.0011 | BMP4,ERBB2,HMGB1,CD274,RUNX3,CDKN2A |
| GO:0060560 | Developmental growth involved in morphogenesis | 6 | 116 | 0.0011 | BMP4,NOTCH1,USP9X,STK11,CTNNB1,POSTN |
| GO:1903034 | Regulation of response to wounding | 7 | 169 | 0.0011 | SERPINE1,MTOR,PTEN,TLR4,SPP1,NFE2L2,TNF |
| GO:2000725 | Regulation of cardiac muscle cell differentiation | 4 | 36 | 0.0011 | TGFB1,BMP4,PAK1,MTOR |
| GO:0000165 | MAPK cascade | 10 | 370 | 0.0012 | MAPK1,TGFB1,HGF,ERBB2,EGFR,MET,JAK2,YWHAZ,TNF,ZFP36 |
| GO:0002320 | Lymphoid progenitor cell differentiation | 3 | 13 | 0.0012 | BMP4,PRKDC,BCL2 |
| GO:0002437 | Inflammatory response to antigenic stimulus | 4 | 37 | 0.0012 | NOTCH1,CXCR2,HMGB1,TNF |
| GO:0003151 | Outflow tract morphogenesis | 5 | 73 | 0.0012 | BMP4,NOTCH1,RARB,HIF1A,VEGFA |
| GO:0006296 | Nucleotide-excision repair, dna incision, 5-to lesion | 4 | 37 | 0.0012 | ERCC1,ERCC4,ERCC5,ERCC2 |
| GO:0010666 | Positive regulation of cardiac muscle cell apoptotic process | 3 | 13 | 0.0012 | TP53,CXCR2,PTEN |
| GO:0014002 | Astrocyte development | 4 | 37 | 0.0012 | IFNG,EGFR,TLR4,TNF |
| GO:0031016 | Pancreas development | 5 | 73 | 0.0012 | BMP4,ALDH1A2,MET,CTNNB1,IL6 |
| GO:0042178 | Xenobiotic catabolic process | 3 | 13 | 0.0012 | GSTM1,ABCC2,UGT1A1 |
| GO:0045445 | Myoblast differentiation | 4 | 37 | 0.0012 | LGALS1,EPAS1,RB1,NOTCH1 |
| GO:0048667 | Cell morphogenesis involved in neuron differentiation | 11 | 445 | 0.0012 | MAPK1,PIK3CA,RB1,ERBB2,NOTCH1,USP9X,STK11,BSG,PTEN,BCL2,VEGFA |
| GO:0051216 | Cartilage development | 7 | 171 | 0.0012 | TGFB1,BMP4,TYMS,RARB,RUNX3,CD44,HIF1A |
| GO:1902947 | Regulation of tau-protein kinase activity | 3 | 13 | 0.0012 | HGF,IFNG,RB1 |
| GO:2001240 | Negative regulation of extrinsic apoptotic signaling pathway in absence of ligand | 4 | 37 | 0.0012 | TERT,MCL1,BCL2,TNF |
| GO:0001952 | Regulation of cell-matrix adhesion | 6 | 120 | 0.0013 | SERPINE1,PTEN,POSTN,BCL2,CDKN2A,VEGFA |
| GO:0001953 | Negative regulation of cell-matrix adhesion | 4 | 38 | 0.0013 | SERPINE1,PTEN,POSTN,CDKN2A |
| GO:0002526 | Acute inflammatory response | 5 | 74 | 0.0013 | STAT3,OGG1,CXCR2,UGT1A1,IL6 |
| GO:0003156 | Regulation of animal organ formation | 4 | 38 | 0.0013 | BMP4,NOTCH1,CTNNB1,AR |
| GO:0006283 | Transcription-coupled nucleotide-excision repair | 5 | 75 | 0.0013 | ERCC1,XRCC1,ERCC4,ERCC5,ERCC2 |
| GO:0007259 | Receptor signaling pathway via jak-stat | 4 | 38 | 0.0013 | IFNG,STAT3,STAT1,JAK2 |
| GO:0021536 | Diencephalon development | 5 | 74 | 0.0013 | BMP4,ALDH1A2,CDH1,BAX,SOX2 |
| GO:0032205 | Negative regulation of telomere maintenance | 4 | 38 | 0.0013 | ERCC1,XRCC1,ERCC4,XRCC5 |
| GO:0035270 | Endocrine system development | 6 | 120 | 0.0013 | MAPK1,BMP4,ALDH1A2,CDH1,SOX2,IL6 |
| GO:0048709 | Oligodendrocyte differentiation | 5 | 74 | 0.0013 | ERBB2,NOTCH1,PTEN,ERCC2,GSTP1 |
| GO:0060411 | Cardiac septum morphogenesis | 5 | 74 | 0.0013 | BMP4,MDM2,TP53,NOTCH1,RARB |
| GO:0090305 | Nucleic acid phosphodiester bond hydrolysis | 9 | 302 | 0.0013 | ERCC1,APEX1,XRCC1,OGG1,ERCC4,RAD51C,HMGB1,ERCC5,ERCC2 |
| GO:2000058 | Regulation of ubiquitin-dependent protein catabolic process | 7 | 174 | 0.0013 | CSNK2A1,SIRT2,MDM2,RB1,PTEN,NFE2L2,CDKN2A |
| GO:0007492 | Endoderm development | 5 | 76 | 0.0014 | DUSP1,BMP4,NOTCH1,SOX2,CTNNB1 |
| GO:0010870 | Positive regulation of receptor biosynthetic process | 3 | 14 | 0.0014 | IFNG,JAK2,HIF1A |
| GO:0034393 | Positive regulation of smooth muscle cell apoptotic process | 3 | 14 | 0.0014 | IFNG,CDKN2A,SOD2 |
| GO:0035112 | Genitalia morphogenesis | 3 | 14 | 0.0014 | TP63,CTNNB1,AR |
| GO:0045732 | Positive regulation of protein catabolic process | 8 | 240 | 0.0014 | CSNK2A1,IFNG,SIRT2,MDM2,RB1,PTEN,NFE2L2,TNF |
| GO:0046903 | Secretion | 18 | 1097 | 0.0014 | MAPK1,TGFB1,HGF,SERPINE1,CCND1,TIMP3,PAK1,AXL,CXCR2,NOS2,HMGB1,ABCC2,XRCC5,GSTP1,IL6,CD44,HIF1A,VEGFA |
| GO:0048639 | Positive regulation of developmental growth | 7 | 176 | 0.0014 | MAPK1,NOTCH1,PAK1,PRKDC,MTOR,BCL2,VEGFA |
| GO:0070243 | Regulation of thymocyte apoptotic process | 3 | 14 | 0.0014 | BMP4,TP53,HIF1A |
| GO:0002067 | Glandular epithelial cell differentiation | 4 | 40 | 0.0015 | BMP4,TP63,NOTCH1,RARB |
| GO:0002891 | Positive regulation of immunoglobulin mediated immune response | 4 | 40 | 0.0015 | TGFB1,MLH1,TP53BP1,TNF |
| GO:0007409 | Axonogenesis | 10 | 384 | 0.0015 | MAPK1,PIK3CA,ERBB2,NOTCH1,USP9X,STK11,BSG,PTEN,BCL2,VEGFA |
| GO:0032735 | Positive regulation of interleukin-12 production | 4 | 40 | 0.0015 | IFNG,HMGB1,TLR4,HSPD1 |
| GO:0048713 | Regulation of oligodendrocyte differentiation | 4 | 40 | 0.0015 | NOTCH1,CTNNB1,MTOR,TP73 |
| GO:1903530 | Regulation of secretion by cell | 13 | 630 | 0.0015 | TGFB1,IFNG,EGFR,NOTCH1,NOS2,TLR4,JAK2,SIRT3,SPP1,IL6,TNF,HIF1A,SLC16A1 |
| GO:0033146 | Regulation of intracellular estrogen receptor signaling pathway | 4 | 41 | 0.0016 | TP63,PAK1,AR,BRCA1 |
| GO:0042692 | Muscle cell differentiation | 8 | 244 | 0.0016 | BMP4,NOX4,RB1,NOTCH1,RARB,CTNNB1,MTOR,VEGFA |
| GO:0050796 | Regulation of insulin secretion | 7 | 182 | 0.0016 | IFNG,NOS2,JAK2,SIRT3,TNF,HIF1A,SLC16A1 |
| GO:0061900 | Glial cell activation | 4 | 41 | 0.0016 | IFNG,EGFR,JAK2,TNF |
| GO:1901362 | Organic cyclic compound biosynthetic process | 19 | 1211 | 0.0016 | MAPK1,TGFB1,CCND1,EPAS1,STAT3,TP53,NOTCH1,BAX,OGG1,TERT,TYMS,RARB,E2F1,MTOR,PTGS2,PTEN,AR,ERCC2,CDKN1A |
| GO:1903053 | Regulation of extracellular matrix organization | 4 | 41 | 0.0016 | TGFB1,RB1,NOTCH1,IL6 |
| GO:0002833 | Positive regulation of response to biotic stimulus | 8 | 247 | 0.0017 | PAK1,PRKDC,HMGB1,TLR4,CD274,HSPD1,XRCC5,MUC1 |
| GO:0014031 | Mesenchymal cell development | 5 | 80 | 0.0017 | MAPK1,ALDH1A2,NOTCH1,BCL2,HIF1A |
| GO:0045605 | Negative regulation of epidermal cell differentiation | 3 | 15 | 0.0017 | TP63,NOTCH1,EZH2 |
| GO:0045651 | Positive regulation of macrophage differentiation | 3 | 15 | 0.0017 | TGFB1,RB1,CASP8 |
| GO:0046328 | Regulation of jnk cascade | 7 | 183 | 0.0017 | IGF1R,EGFR,PAK1,HMGB1,TLR4,GSTP1,TNF |
| GO:0048569 | Post-embryonic animal organ development | 3 | 15 | 0.0017 | ERCC1,BAX,VEGFA |
| GO:0070234 | Positive regulation of t cell apoptotic process | 3 | 15 | 0.0017 | TP53,PDCD1,CD274 |
| GO:0003018 | Vascular process in circulatory system | 7 | 185 | 0.0018 | TGFB1,EGFR,CXCR2,PTGS2,ABCC2,SOD2,VEGFA |
| GO:0014014 | Negative regulation of gliogenesis | 4 | 42 | 0.0018 | SIRT2,NOTCH1,TERT,CTNNB1 |
| GO:0021983 | Pituitary gland development | 4 | 42 | 0.0018 | BMP4,ALDH1A2,CDH1,SOX2 |
| GO:0034620 | Cellular response to unfolded protein | 6 | 129 | 0.0018 | CCND1,BAX,HSPA5,HDGF,HSPD1,NFE2L2 |
| GO:0042149 | Cellular response to glucose starvation | 4 | 42 | 0.0018 | TP53,HSPA5,NFE2L2,BCL2 |
| GO:0061326 | Renal tubule development | 5 | 81 | 0.0018 | BMP4,NOTCH1,CTNNB1,STAT1,BCL2 |
| GO:0072073 | Kidney epithelium development | 6 | 130 | 0.0018 | BMP4,NOTCH1,CXCR2,RARB,CTNNB1,BCL2 |
| GO:1904036 | Negative regulation of epithelial cell apoptotic process | 4 | 42 | 0.0018 | SERPINE1,TERT,MTOR,NFE2L2 |
| GO:2000279 | Negative regulation of dna biosynthetic process | 4 | 42 | 0.0018 | DUSP1,TP53,ERCC4,CDKN1A |
| GO:0042100 | B cell proliferation | 4 | 43 | 0.0019 | BAX,TLR4,HSPD1,BCL2 |
| GO:0045687 | Positive regulation of glial cell differentiation | 4 | 43 | 0.0019 | TGFB1,NOTCH1,MTOR,TP73 |
| GO:0045727 | Positive regulation of translation | 6 | 131 | 0.0019 | MAPK1,ERBB2,PRKDC,MTOR,IL6,TNF |
| GO:0046651 | Lymphocyte proliferation | 5 | 82 | 0.0019 | TP53,BAX,TLR4,HSPD1,BCL2 |
| GO:0060043 | Regulation of cardiac muscle cell proliferation | 4 | 43 | 0.0019 | MAPK1,NOTCH1,PTEN,TP73 |
| GO:0071364 | Cellular response to epidermal growth factor stimulus | 4 | 43 | 0.0019 | ERBB2,EGFR,GSTP1,ZFP36 |
| GO:1901224 | Positive regulation of nik/nf-kappab signaling | 5 | 82 | 0.0019 | TNFRSF10A,EGFR,HMGB1,TLR4,TNF |
| GO:0002837 | Regulation of immune response to tumor cell | 3 | 16 | 0.002 | HMGB1,CD274,HSPD1 |
| GO:0006206 | Pyrimidine nucleobase metabolic process | 3 | 16 | 0.002 | MAPK1,TYMS,MTOR |
| GO:0006417 | Regulation of translation | 10 | 398 | 0.002 | MAPK1,STAT3,ERBB2,PRKDC,TYMS,MTOR,IL6,TNF,EIF4E,ZFP36 |
| GO:0008584 | Male gonad development | 6 | 133 | 0.002 | ERCC1,CCND1,TNFSF10,BAX,AR,BCL2 |
| GO:0010225 | Response to uv-c | 3 | 16 | 0.002 | MDM2,TP53,ERCC5 |
| GO:0010463 | Mesenchymal cell proliferation | 3 | 16 | 0.002 | BMP4,CTNNB1,STAT1 |
| GO:0033045 | Regulation of sister chromatid segregation | 5 | 83 | 0.002 | DUSP1,RB1,BIRC5,CTNNB1,XRCC3 |
| GO:0033365 | Protein localization to organelle | 14 | 743 | 0.002 | BMP4,MDM2,STAT3,RB1,TP53,BAX,TERT,USP9X,EZH2,HSPA5,NOS2,HSPD1,XRCC5,CDKN1A |
| GO:0043117 | Positive regulation of vascular permeability | 3 | 16 | 0.002 | TGFB1,CXCR2,VEGFA |
| GO:0060218 | Hematopoietic stem cell differentiation | 3 | 16 | 0.002 | TP53,ERCC2,XRCC5 |
| GO:0060391 | Positive regulation of smad protein signal transduction | 3 | 16 | 0.002 | TGFB1,BMP4,JAK2 |
| GO:0061061 | Muscle structure development | 11 | 479 | 0.002 | LGALS1,TGFB1,BMP4,NOX4,EPAS1,RB1,NOTCH1,RARB,CTNNB1,MTOR,VEGFA |
| GO:0070231 | T cell apoptotic process | 3 | 16 | 0.002 | BAX,FAS,FASLG |
| GO:0071850 | Mitotic cell cycle arrest | 3 | 16 | 0.002 | DUSP1,TP53,CDKN1A |
| GO:0140352 | Export from cell | 17 | 1028 | 0.002 | MAPK1,TGFB1,HGF,SERPINE1,TIMP3,PAK1,AXL,CXCR2,NOS2,HMGB1,ABCC2,XRCC5,GSTP1,ABCC1,IL6,CD44,VEGFA |
| GO:1905564 | Positive regulation of vascular endothelial cell proliferation | 3 | 16 | 0.002 | STAT3,ADAM17,HMGB1 |
| GO:0001656 | Metanephros development | 5 | 84 | 0.0021 | BMP4,CXCR2,CTNNB1,STAT1,BCL2 |
| GO:0003169 | Coronary vein morphogenesis | 2 | 2 | 0.0021 | NOTCH1,VEGFA |
| GO:0006351 | Transcription, dna-templated | 12 | 567 | 0.0021 | CCND1,EPAS1,STAT3,TP53,NOTCH1,BAX,RARB,E2F1,PTEN,AR,ERCC2,CDKN1A |
| GO:0046666 | Retinal cell programmed cell death | 2 | 2 | 0.0021 | BAX,FASLG |
| GO:0046777 | Protein autophosphorylation | 7 | 192 | 0.0021 | IGF1R,ERBB2,EGFR,PAK1,STK11,MTOR,JAK2 |
| GO:0050671 | Positive regulation of lymphocyte proliferation | 6 | 134 | 0.0021 | HMGB1,TLR4,CD274,BCL2,CDKN1A,IL6 |
| GO:0055007 | Cardiac muscle cell differentiation | 5 | 84 | 0.0021 | BMP4,NOX4,RARB,MTOR,VEGFA |
| GO:0071103 | DNA conformation change | 9 | 328 | 0.0021 | SIRT2,TP53,HMGB1,RAD51,ERCC2,XRCC5,TOP2A,CDKN2A,H2AFX |
| GO:0071716 | Leukotriene transport | 2 | 2 | 0.0021 | ABCC2,ABCC1 |
| GO:1901963 | Regulation of cell proliferation involved in outflow tract morphogenesis | 2 | 2 | 0.0021 | BMP4,CTNNB1 |
| GO:1902728 | Positive regulation of growth factor dependent skeletal muscle satellite cell proliferation | 2 | 2 | 0.0021 | STAT3,JAK2 |
| GO:2000779 | Regulation of double-strand break repair | 5 | 84 | 0.0021 | MGMT,OGG1,PRKDC,TP53BP1,RAD51 |
| GO:0000712 | Resolution of meiotic recombination intermediates | 3 | 17 | 0.0022 | MLH1,ERCC4,TOP2A |
| GO:0002643 | Regulation of tolerance induction | 3 | 17 | 0.0022 | PDCD1,HMGB1,CD274 |
| GO:0006366 | Transcription by rna polymerase ii | 10 | 406 | 0.0022 | CCND1,EPAS1,STAT3,NOTCH1,BAX,RARB,PTEN,AR,ERCC2,CDKN1A |
| GO:0008631 | Intrinsic apoptotic signaling pathway in response to oxidative stress | 3 | 17 | 0.0022 | JAK2,BCL2,CYP1B1 |
| GO:0019827 | Stem cell population maintenance | 6 | 135 | 0.0022 | STAT3,NOTCH1,SOX2,CTNNB1,KDM4C,EIF4E |
| GO:0030540 | Female genitalia development | 3 | 17 | 0.0022 | TP63,BAX,AXL |
| GO:0034198 | Cellular response to amino acid starvation | 4 | 45 | 0.0022 | MAPK1,FAS,MTOR,CDKN1A |
| GO:0042177 | Negative regulation of protein catabolic process | 6 | 135 | 0.0022 | CSNK2A1,SIRT2,TIMP3,EGFR,NOS2,CDKN2A |
| GO:0045603 | Positive regulation of endothelial cell differentiation | 3 | 17 | 0.0022 | BMP4,NOTCH1,CTNNB1 |
| GO:0045911 | Positive regulation of dna recombination | 4 | 45 | 0.0022 | TGFB1,MLH1,TP53BP1,ERCC2 |
| GO:0046889 | Positive regulation of lipid biosynthetic process | 5 | 85 | 0.0022 | IFNG,MTOR,PTGS2,SIRT3,TNF |
| GO:0071248 | Cellular response to metal ion | 7 | 193 | 0.0022 | MAPK1,CDH1,EGFR,OGG1,HSPA5,PTGS2,NFE2L2 |
| GO:1902166 | Negative regulation of intrinsic apoptotic signaling pathway in response to dna damage by p53 class mediator | 3 | 17 | 0.0022 | BCL2,CD44,MUC1 |
| GO:1903206 | Negative regulation of hydrogen peroxide-induced cell death | 3 | 17 | 0.0022 | HGF,MET,NFE2L2 |
| GO:1904754 | Positive regulation of vascular associated smooth muscle cell migration | 3 | 17 | 0.0022 | MDM2,PAK1,TERT |
| GO:2000136 | Regulation of cell proliferation involved in heart morphogenesis | 3 | 17 | 0.0022 | BMP4,NOTCH1,CTNNB1 |
| GO:2000269 | Regulation of fibroblast apoptotic process | 3 | 17 | 0.0022 | PIK3CA,TP63,TP53 |
| GO:0002639 | Positive regulation of immunoglobulin production | 4 | 46 | 0.0023 | TGFB1,MLH1,TP53BP1,IL6 |
| GO:0014068 | Positive regulation of phosphatidylinositol 3-kinase signaling | 5 | 87 | 0.0023 | HGF,PIK3CA,IGF1R,JAK2,TNF |
| GO:0048538 | Thymus development | 4 | 46 | 0.0023 | MAPK1,PRKDC,CTNNB1,BCL2 |
| GO:0050999 | Regulation of nitric-oxide synthase activity | 4 | 46 | 0.0023 | EGFR,TERT,TNF,HIF1A |
| GO:0070664 | Negative regulation of leukocyte proliferation | 5 | 87 | 0.0023 | BMP4,ERBB2,CD274,GSTP1,CDKN2A |
| GO:0001892 | Embryonic placenta development | 5 | 88 | 0.0025 | MAPK1,EPAS1,EGFR,CASP8,HIF1A |
| GO:0002673 | Regulation of acute inflammatory response | 4 | 47 | 0.0025 | PTGS2,GSTP1,IL6,TNF |
| GO:0010224 | Response to uv-b | 3 | 18 | 0.0025 | STK11,BCL2,CDKN1A |
| GO:0032760 | Positive regulation of tumor necrosis factor production | 5 | 88 | 0.0025 | IFNG,HMGB1,TLR4,JAK2,HSPD1 |
| GO:0033194 | Response to hydroperoxide | 3 | 18 | 0.0025 | XRCC1,JAK2,GPX3 |
| GO:1900544 | Positive regulation of purine nucleotide metabolic process | 4 | 47 | 0.0025 | IFNG,STAT3,NOS2,HIF1A |
| GO:1902275 | Regulation of chromatin organization | 7 | 198 | 0.0025 | TP53,CTNNB1,MKI67,KDM4C,BRCA1,VEGFA,MUC1 |
| GO:0042220 | Response to cocaine | 4 | 48 | 0.0026 | MDM2,HSPA5,MTOR,HSPD1 |
| GO:0061041 | Regulation of wound healing | 6 | 140 | 0.0026 | SERPINE1,MTOR,PTEN,TLR4,NFE2L2,TNF |
| GO:0002218 | Activation of innate immune response | 6 | 142 | 0.0027 | PAK1,PRKDC,HMGB1,TLR4,XRCC5,MUC1 |
| GO:0010467 | Gene expression | 26 | 2056 | 0.0027 | CCND1,NOX4,EPAS1,STAT3,TP53,EGFR,NOTCH1,BAX,TERT,PRKDC,NOS2,RARB,HMGB1,E2F1,CASP8,PTGS2,PTEN,TLR4,AR,HSPD1,ERCC2,CDKN1A,TNF,CDKN2A,EIF4E,HIF1A |
| GO:0051262 | Protein tetramerization | 5 | 90 | 0.0027 | ALDH1A2,TP63,TP53,TP73,SOD2 |
| GO:0009746 | Response to hexose | 6 | 143 | 0.0028 | LGALS1,NOX4,PIK3CA,IGF1R,PTGS2,PTEN |
| GO:0090199 | Regulation of release of cytochrome c from mitochondria | 4 | 49 | 0.0028 | HGF,TNFSF10,TP53,BAX |
| GO:2000107 | Negative regulation of leukocyte apoptotic process | 4 | 49 | 0.0028 | BMP4,AXL,CXCR2,HIF1A |
| GO:0016331 | Morphogenesis of embryonic epithelium | 6 | 144 | 0.0029 | TGFB1,BMP4,ALDH1A2,TP63,CTNNB1,HIF1A |
| GO:0062013 | Positive regulation of small molecule metabolic process | 6 | 144 | 0.0029 | IFNG,STAT3,NOS2,PTGS2,TNF,HIF1A |
| GO:1902176 | Negative regulation of oxidative stress-induced intrinsic apoptotic signaling pathway | 3 | 19 | 0.0029 | NFE2L2,HIF1A,SOD2 |
| GO:2000727 | Positive regulation of cardiac muscle cell differentiation | 3 | 19 | 0.0029 | TGFB1,BMP4,MTOR |
| GO:0007173 | Epidermal growth factor receptor signaling pathway | 4 | 50 | 0.003 | TGFB1,PIK3CA,EGFR,ADAM17 |
| GO:0019216 | Regulation of lipid metabolic process | 10 | 424 | 0.003 | TGFB1,IFNG,RB1,MTOR,PTGS2,UGT1A1,SIRT3,TNF,BRCA1,PDK2 |
| GO:0022412 | Cellular process involved in reproduction in multicellular organism | 9 | 345 | 0.003 | ERCC1,MLH1,BMP4,BAX,RAD51C,CTNNB1,MTOR,BCL2,TOP2A |
| GO:0034613 | Cellular protein localization | 22 | 1610 | 0.003 | TGFB1,BMP4,MDM2,CDH1,STAT3,RB1,TP53,EGFR,BAX,TERT,USP9X,EZH2,HSPA5,NOS2,BSG,CTNNB1,HSPD1,XRCC5,YWHAZ,CDKN1A,TNF,EIF4E |
| GO:0006338 | Chromatin remodeling | 7 | 206 | 0.0031 | SIRT2,TP63,RB1,PAK1,HMGB1,KDM4C,CDKN2A |
| GO:0032206 | Positive regulation of telomere maintenance | 4 | 51 | 0.0032 | ERCC1,MAPK1,CTNNB1,XRCC5 |
| GO:0045580 | Regulation of t cell differentiation | 6 | 147 | 0.0032 | IFNG,BMP4,ERBB2,HMGB1,RUNX3,CDKN2A |
| GO:0048678 | Response to axon injury | 4 | 51 | 0.0032 | LGALS1,BAX,JAK2,BCL2 |
| GO:0120035 | Regulation of plasma membrane bounded cell projection organization | 13 | 687 | 0.0032 | LGALS1,HGF,MDM2,PAK1,EZH2,HSPA5,STK11,MTOR,PTEN,SPP1,NFE2L2,CD44,VEGFA |
| GO:2000142 | Regulation of dna-templated transcription, initiation | 4 | 51 | 0.0032 | ERCC1,TP53,CTNNB1,HMGB1 |
| GO:0003340 | Negative regulation of mesenchymal to epithelial transition involved in metanephros morphogenesis | 2 | 3 | 0.0033 | CTNNB1,STAT1 |
| GO:0006808 | Regulation of nitrogen utilization | 2 | 3 | 0.0033 | BAX,BCL2 |
| GO:0031058 | Positive regulation of histone modification | 5 | 95 | 0.0033 | TP53,CTNNB1,BRCA1,VEGFA,MUC1 |
| GO:0032930 | Positive regulation of superoxide anion generation | 3 | 20 | 0.0033 | TGFB1,EGFR,GSTP1 |
| GO:0034462 | Small-subunit processome assembly | 2 | 3 | 0.0033 | PRKDC,XRCC5 |
| GO:0042088 | T-helper 1 type immune response | 3 | 20 | 0.0033 | HMGB1,MTOR,TLR4 |
| GO:0045190 | Isotype switching | 3 | 20 | 0.0033 | ERCC1,MLH1,HSPD1 |
| GO:0048143 | Astrocyte activation | 3 | 20 | 0.0033 | IFNG,EGFR,TNF |
| GO:0060197 | Cloacal septation | 2 | 3 | 0.0033 | BMP4,TP63 |
| GO:0060559 | Positive regulation of calcidiol 1-monooxygenase activity | 2 | 3 | 0.0033 | IFNG,TNF |
| GO:0060769 | Positive regulation of epithelial cell proliferation involved in prostate gland development | 2 | 3 | 0.0033 | CTNNB1,AR |
| GO:0060948 | Cardiac vascular smooth muscle cell development | 2 | 3 | 0.0033 | NOTCH1,VEGFA |
| GO:0071930 | Negative regulation of transcription involved in g1/s transition of mitotic cell cycle | 2 | 3 | 0.0033 | RB1,E2F1 |
| GO:0072717 | Cellular response to actinomycin d | 2 | 3 | 0.0033 | MDM2,TP53 |
| GO:1901655 | Cellular response to ketone | 5 | 95 | 0.0033 | EGFR,ABCC2,AR,SPP1,EIF4E |
| GO:1902263 | Apoptotic process involved in embryonic digit morphogenesis | 2 | 3 | 0.0033 | NOTCH1,BAX |
| GO:1902948 | Negative regulation of tau-protein kinase activity | 2 | 3 | 0.0033 | IFNG,RB1 |
| GO:1903984 | Positive regulation of trail-activated apoptotic signaling pathway | 2 | 3 | 0.0033 | TIMP3,PTEN |
| GO:2000137 | Negative regulation of cell proliferation involved in heart morphogenesis | 2 | 3 | 0.0033 | BMP4,NOTCH1 |
| GO:2000635 | Negative regulation of primary mirna processing | 2 | 3 | 0.0033 | STAT3,IL6 |
| GO:2000773 | Negative regulation of cellular senescence | 3 | 20 | 0.0033 | TP63,TERT,PRKDC |
| GO:0032787 | Monocarboxylic acid metabolic process | 11 | 515 | 0.0034 | ALDH1A2,GSTM1,BSG,PTGS2,ABCC2,UGT1A1,GSTP1,BRCA1,HIF1A,SLC16A1,CYP1B1 |
| GO:0035265 | Organ growth | 5 | 96 | 0.0034 | NOTCH1,RARB,PTEN,AR,BCL2 |
| GO:0071560 | Cellular response to transforming growth factor beta stimulus | 6 | 149 | 0.0034 | TGFB1,GDF15,NOX4,TP53,USP9X,POSTN |
| GO:0016579 | Protein deubiquitination | 8 | 281 | 0.0035 | MDM2,TP53,USP9X,PTEN,AR,BRCA1,HIF1A,BIRC2 |
| GO:0044772 | Mitotic cell cycle phase transition | 8 | 280 | 0.0035 | CCND1,RB1,BIRC5,TYMS,E2F1,CDKN1A,CDKN2A,EIF4E |
| GO:0000186 | Activation of mapkk activity | 4 | 53 | 0.0036 | MAPK1,BMP4,EGFR,JAK2 |
| GO:0002260 | Lymphocyte homeostasis | 4 | 53 | 0.0036 | BAX,FAS,BCL2,HIF1A |
| GO:0031056 | Regulation of histone modification | 6 | 151 | 0.0036 | TP53,CTNNB1,KDM4C,BRCA1,VEGFA,MUC1 |
| GO:0035282 | Segmentation | 5 | 97 | 0.0036 | BMP4,ALDH1A2,TP53,PRKDC,BMI1 |
| GO:0042116 | Macrophage activation | 4 | 53 | 0.0036 | IFNG,TLR4,JAK2,TNF |
| GO:0051701 | Interaction with host | 6 | 151 | 0.0036 | CDH1,EGFR,AXL,MET,CTNNB1,CASP8 |
| GO:1903055 | Positive regulation of extracellular matrix organization | 3 | 21 | 0.0036 | TGFB1,RB1,IL6 |
| GO:2000737 | Negative regulation of stem cell differentiation | 3 | 21 | 0.0036 | STAT3,NOTCH1,NFE2L2 |
| GO:2001026 | Regulation of endothelial cell chemotaxis | 3 | 21 | 0.0036 | NOTCH1,MET,VEGFA |
| GO:0045834 | Positive regulation of lipid metabolic process | 6 | 152 | 0.0037 | TGFB1,IFNG,MTOR,PTGS2,SIRT3,TNF |
| GO:0010675 | Regulation of cellular carbohydrate metabolic process | 6 | 153 | 0.0038 | STAT3,TP53,MTOR,UGT1A1,IGFBP3,PDK2 |
| GO:0045087 | Innate immune response | 13 | 703 | 0.0038 | IFNG,SIRT2,TP53,AXL,PRKDC,NOS2,HMGB1,STAT1,FASLG,TLR4,JAK2,XRCC5,CD44 |
| GO:0050792 | Regulation of viral process | 7 | 215 | 0.0038 | LGALS1,NOTCH1,STAT1,BCL2,TNF,TOP2A,ZFP36 |
| GO:0007283 | Spermatogenesis | 11 | 525 | 0.0039 | ERCC1,MLH1,TP63,NOTCH1,BAX,AXL,STK11,RAD51C,E2F1,AR,H2AFX |
| GO:1901576 | Organic substance biosynthetic process | 31 | 2734 | 0.0039 | MAPK1,CSNK2A1,TGFB1,CCND1,ALDH1A2,EPAS1,PIK3CA,STAT3,TP53,EGFR,NOTCH1,BAX,OGG1,TERT,GSTM1,TYMS,RARB,E2F1,MTOR,PTGS2,PTEN,AR,RAD51,ERCC2,GSTP1,CDKN1A,TNF,BRCA1,EIF4E,HIF1A,MUC1 |
| GO:0002224 | Toll-like receptor signaling pathway | 5 | 100 | 0.004 | HMGB1,CASP8,TLR4,HSPD1,BIRC2 |
| GO:0032757 | Positive regulation of interleukin-8 production | 4 | 55 | 0.004 | SERPINE1,HMGB1,TLR4,TNF |
| GO:0051000 | Positive regulation of nitric-oxide synthase activity | 3 | 22 | 0.004 | TERT,TNF,HIF1A |
| GO:0090183 | Regulation of kidney development | 4 | 55 | 0.004 | BMP4,CTNNB1,STAT1,VEGFA |
| GO:0090312 | Positive regulation of protein deacetylation | 3 | 22 | 0.004 | IFNG,TP53,VEGFA |
| GO:0010976 | Positive regulation of neuron projection development | 8 | 288 | 0.0041 | HGF,PAK1,EZH2,HSPA5,STK11,MTOR,NFE2L2,VEGFA |
| GO:0019217 | Regulation of fatty acid metabolic process | 5 | 101 | 0.0042 | MTOR,PTGS2,UGT1A1,BRCA1,PDK2 |
| GO:0050804 | Modulation of chemical synaptic transmission | 10 | 446 | 0.0042 | MAPK1,CDH1,STAT3,EGFR,MTOR,PTGS2,PTEN,JAK2,TNF,EIF4E |
| GO:1990748 | Cellular detoxification | 5 | 101 | 0.0042 | GSTM1,PTGS2,GPX3,GSTP1,SOD2 |
| GO:0000187 | Activation of mapk activity | 6 | 157 | 0.0043 | MAPK1,HGF,GDF15,TLR4,TP73,TNF |
| GO:0030071 | Regulation of mitotic metaphase/anaphase transition | 4 | 56 | 0.0043 | DUSP1,RB1,BIRC5,XRCC3 |
| GO:0036297 | Interstrand cross-link repair | 4 | 56 | 0.0043 | ERCC1,ERCC4,RAD51,XRCC3 |
| GO:0006810 | Transport | 43 | 4353 | 0.0044 | MAPK1,TGFB1,HGF,SERPINE1,CCND1,PIK3CA,STAT3,TP63,TIMP3,IGF1R,TP53,PAK1,BAX,AXL,USP9X,CXCR2,HSPA5,NOS2,CTNNB1,HMGB1,E2F1,CASP8,FASLG,MCL1,ABCC2,ABCC10,TLR4,TP73,CD274,HSPD1,XRCC5,YWHAZ,BCL2,GSTP1,ABCC1,CDKN1A,IL6,CD44,EIF4E,HIF1A,SLC16A1,ZFP36,VEGFA |
| GO:0006359 | Regulation of transcription by rna polymerase iii | 3 | 23 | 0.0045 | MTOR,AR,BRCA1 |
| GO:0008593 | Regulation of notch signaling pathway | 5 | 103 | 0.0045 | STAT3,TP63,EGFR,NOTCH1,POSTN |
| GO:0010830 | Regulation of myotube differentiation | 4 | 57 | 0.0045 | GDF15,NOTCH1,MTOR,BCL2 |
| GO:0030111 | Regulation of wnt signaling pathway | 9 | 370 | 0.0045 | CSNK2A1,TGFB1,EGFR,NOTCH1,TERT,SOX2,STK11,CTNNB1,XIAP |
| GO:0032331 | Negative regulation of chondrocyte differentiation | 3 | 23 | 0.0045 | BMP4,RARB,CTNNB1 |
| GO:0044321 | Response to leptin | 3 | 23 | 0.0045 | CCND1,STAT3,PTEN |
| GO:0044409 | Entry into host | 5 | 103 | 0.0045 | CDH1,EGFR,AXL,MET,CTNNB1 |
| GO:0035306 | Positive regulation of dephosphorylation | 4 | 58 | 0.0047 | TGFB1,IFNG,MTOR,JAK2 |
| GO:0060760 | Positive regulation of response to cytokine stimulus | 4 | 58 | 0.0047 | AXL,ADAM17,TLR4,HIF1A |
| GO:0061044 | Negative regulation of vascular wound healing | 2 | 4 | 0.0047 | SERPINE1,TNF |
| GO:0061047 | Positive regulation of branching involved in lung morphogenesis | 2 | 4 | 0.0047 | BMP4,CTNNB1 |
| GO:0062014 | Negative regulation of small molecule metabolic process | 5 | 104 | 0.0047 | TGFB1,STAT3,TP53,UGT1A1,BRCA1 |
| GO:0098771 | Inorganic ion homeostasis | 13 | 723 | 0.0047 | MAPK1,EPAS1,EGFR,BAX,CXCR2,HMGB1,FASLG,ABCC2,XIAP,JAK2,BCL2,PDK2,HIF1A |
| GO:0099547 | Regulation of translation at synapse, modulating synaptic transmission | 2 | 4 | 0.0047 | MTOR,EIF4E |
| GO:1901671 | Positive regulation of superoxide dismutase activity | 2 | 4 | 0.0047 | SIRT3,TNF |
| GO:1903076 | Regulation of protein localization to plasma membrane | 5 | 104 | 0.0047 | TGFB1,IFNG,EGFR,AR,TNF |
| GO:0002702 | Positive regulation of production of molecular mediator of immune response | 5 | 105 | 0.0048 | TGFB1,MLH1,TLR4,TP53BP1,IL6 |
| GO:0070647 | Protein modification by small protein conjugation or removal | 16 | 1021 | 0.0048 | MDM2,EPAS1,TP53,PRKDC,USP9X,CTNNB1,XIAP,PTEN,AR,BMI1,NFE2L2,BCL2,CDKN2A,BRCA1,HIF1A,BIRC2 |
| GO:0001782 | B cell homeostasis | 3 | 24 | 0.0049 | BAX,BCL2,HIF1A |
| GO:0051043 | Regulation of membrane protein ectodomain proteolysis | 3 | 24 | 0.0049 | IFNG,TIMP3,TNF |
| GO:0000018 | Regulation of dna recombination | 5 | 106 | 0.005 | TGFB1,MLH1,TP53BP1,RAD51,ERCC2 |
| GO:0016567 | Protein ubiquitination | 13 | 729 | 0.005 | MDM2,EPAS1,PRKDC,USP9X,CTNNB1,XIAP,BMI1,NFE2L2,BCL2,CDKN2A,BRCA1,HIF1A,BIRC2 |
| GO:0021549 | Cerebellum development | 5 | 106 | 0.005 | XRCC1,TP53,PAK1,EZH2,HSPA5 |
| GO:0043279 | Response to alkaloid | 5 | 106 | 0.005 | MDM2,HSPA5,MTOR,RAD51,HSPD1 |
| GO:0090303 | Positive regulation of wound healing | 4 | 59 | 0.005 | SERPINE1,MTOR,TLR4,NFE2L2 |
| GO:0120254 | Olefinic compound metabolic process | 5 | 106 | 0.005 | ALDH1A2,GSTM1,PTGS2,GSTP1,CYP1B1 |
| GO:0045912 | Negative regulation of carbohydrate metabolic process | 4 | 60 | 0.0053 | TGFB1,STAT3,TP53,UGT1A1 |
| GO:2001244 | Positive regulation of intrinsic apoptotic signaling pathway | 4 | 60 | 0.0053 | TP53,BAX,MCL1,BCL2 |
| GO:0002053 | Positive regulation of mesenchymal cell proliferation | 3 | 25 | 0.0054 | CTNNB1,STAT1,VEGFA |
| GO:0048512 | Circadian behavior | 3 | 25 | 0.0054 | TP53,MTA1,PTEN |
| GO:0055022 | Negative regulation of cardiac muscle tissue growth | 3 | 25 | 0.0054 | PAK1,PTEN,TP73 |
| GO:1903409 | Reactive oxygen species biosynthetic process | 3 | 25 | 0.0054 | NOX4,NOS2,CYP1B1 |
| GO:0006690 | Icosanoid metabolic process | 5 | 109 | 0.0056 | GSTM1,PTGS2,GSTP1,ABCC1,CYP1B1 |
| GO:0042733 | Embryonic digit morphogenesis | 4 | 61 | 0.0056 | BMP4,NOTCH1,BAX,CTNNB1 |
| GO:2000514 | Regulation of cd4-positive, alpha-beta t cell activation | 4 | 61 | 0.0056 | IFNG,HMGB1,CD274,RUNX3 |
| GO:0030858 | Positive regulation of epithelial cell differentiation | 4 | 62 | 0.0059 | SERPINE1,BMP4,NOTCH1,CTNNB1 |
| GO:1903556 | Negative regulation of tumor necrosis factor superfamily cytokine production | 4 | 62 | 0.0059 | AXL,TLR4,CD274,GSTP1 |
| GO:0060334 | Regulation of interferon-gamma-mediated signaling pathway | 3 | 26 | 0.006 | IFNG,STAT1,JAK2 |
| GO:0070911 | Global genome nucleotide-excision repair | 3 | 26 | 0.006 | ERCC1,ERCC4,ERCC2 |
| GO:0006805 | Xenobiotic metabolic process | 5 | 112 | 0.0062 | GSTM1,ABCC2,UGT1A1,GSTP1,CYP1B1 |
| GO:0001302 | Replicative cell aging | 2 | 5 | 0.0063 | ERCC1,TP63 |
| GO:0002248 | Connective tissue replacement involved in inflammatory response wound healing | 2 | 5 | 0.0063 | TGFB1,HIF1A |
| GO:0002840 | Regulation of t cell mediated immune response to tumor cell | 2 | 5 | 0.0063 | HMGB1,HSPD1 |
| GO:0014042 | Positive regulation of neuron maturation | 2 | 5 | 0.0063 | MTOR,BCL2 |
| GO:0030644 | Cellular chloride ion homeostasis | 2 | 5 | 0.0063 | FASLG,ABCC2 |
| GO:0034349 | Glial cell apoptotic process | 2 | 5 | 0.0063 | RB1,TP53 |
| GO:0040016 | Embryonic cleavage | 2 | 5 | 0.0063 | ERCC2,TOP2A |
| GO:0048298 | Positive regulation of isotype switching to iga isotypes | 2 | 5 | 0.0063 | TGFB1,MLH1 |
| GO:0097350 | Neutrophil clearance | 2 | 5 | 0.0063 | AXL,HMGB1 |
| GO:1901299 | Negative regulation of hydrogen peroxide-mediated programmed cell death | 2 | 5 | 0.0063 | HGF,MET |
| GO:1902512 | Positive regulation of apoptotic dna fragmentation | 2 | 5 | 0.0063 | BAX,IL6 |
| GO:1990414 | Replication-born double-strand break repair via sister chromatid exchange | 2 | 5 | 0.0063 | XRCC1,RAD51 |
| GO:0030888 | Regulation of b cell proliferation | 4 | 64 | 0.0065 | TLR4,BCL2,CDKN1A,CDKN2A |
| GO:0034502 | Protein localization to chromosome | 4 | 64 | 0.0065 | RB1,TERT,EZH2,XRCC5 |
| GO:0055082 | Cellular chemical homeostasis | 13 | 753 | 0.0065 | MAPK1,LGALS1,NOX4,PIK3CA,IGF1R,BAX,CXCR2,HMGB1,FASLG,ABCC2,JAK2,BCL2,HIF1A |
| GO:0071677 | Positive regulation of mononuclear cell migration | 3 | 27 | 0.0065 | SERPINE1,HMGB1,TNF |
| GO:1904019 | Epithelial cell apoptotic process | 3 | 27 | 0.0065 | RB1,E2F1,TNF |
| GO:1904356 | Regulation of telomere maintenance via telomere lengthening | 4 | 64 | 0.0065 | MAPK1,ERCC4,CTNNB1,XRCC5 |
| GO:1990138 | Neuron projection extension | 4 | 64 | 0.0065 | USP9X,STK11,CTNNB1,POSTN |
| GO:0016101 | Diterpenoid metabolic process | 5 | 114 | 0.0066 | ALDH1A2,EGFR,ALDH1A1,UGT1A1,CYP1B1 |
| GO:0032392 | DNA geometric change | 5 | 114 | 0.0066 | TP53,HMGB1,RAD51,ERCC2,XRCC5 |
| GO:0051100 | Negative regulation of binding | 6 | 173 | 0.0066 | BAX,ERCC4,MET,E2F1,JAK2,CDKN1A |
| GO:2000060 | Positive regulation of ubiquitin-dependent protein catabolic process | 5 | 115 | 0.0069 | SIRT2,MDM2,RB1,PTEN,NFE2L2 |
| GO:0021987 | Cerebral cortex development | 5 | 116 | 0.0071 | EGFR,BAX,CTNNB1,H2AFX,HIF1A |
| GO:0030194 | Positive regulation of blood coagulation | 3 | 28 | 0.0071 | SERPINE1,TLR4,NFE2L2 |
| GO:0033137 | Negative regulation of peptidyl-serine phosphorylation | 3 | 28 | 0.0071 | HGF,BAX,PTEN |
| GO:0042130 | Negative regulation of t cell proliferation | 4 | 66 | 0.0071 | BMP4,ERBB2,CD274,CDKN2A |
| GO:0043010 | Camera-type eye development | 8 | 318 | 0.0071 | BMP4,ALDH1A2,EGFR,BAX,CTNNB1,HIF1A,CYP1B1,VEGFA |
| GO:0045830 | Positive regulation of isotype switching | 3 | 28 | 0.0071 | TGFB1,MLH1,TP53BP1 |
| GO:0060795 | Cell fate commitment involved in formation of primary germ layer | 3 | 28 | 0.0071 | BMP4,SOX2,CTNNB1 |
| GO:0070168 | Negative regulation of biomineral tissue development | 3 | 28 | 0.0071 | TGFB1,NOTCH1,HIF1A |
| GO:0090025 | Regulation of monocyte chemotaxis | 3 | 28 | 0.0071 | SERPINE1,DUSP1,HMGB1 |
| GO:0090200 | Positive regulation of release of cytochrome c from mitochondria | 3 | 28 | 0.0071 | TNFSF10,TP53,BAX |
| GO:0051781 | Positive regulation of cell division | 5 | 117 | 0.0073 | TGFB1,SIRT2,RB1,HDGF,VEGFA |
| GO:0033036 | Macromolecule localization | 28 | 2473 | 0.0075 | TGFB1,BMP4,MDM2,CDH1,STAT3,RB1,TP53,EGFR,BAX,BIRC5,TERT,USP9X,EZH2,HSPA5,NOS2,BSG,CTNNB1,HMGB1,MCL1,ABCC2,HSPD1,XRCC5,YWHAZ,ABCC1,CDKN1A,TNF,EIF4E,ZFP36 |
| GO:0043542 | Endothelial cell migration | 4 | 67 | 0.0075 | PIK3CA,PTEN,CYP1B1,VEGFA |
| GO:0002675 | Positive regulation of acute inflammatory response | 3 | 29 | 0.0077 | PTGS2,IL6,TNF |
| GO:0042634 | Regulation of hair cycle | 3 | 29 | 0.0077 | TERT,CTNNB1,TNF |
| GO:0045736 | Negative regulation of cyclin-dependent protein serine/threonine kinase activity | 3 | 29 | 0.0077 | PTEN,CDKN1A,CDKN2A |
| GO:0048147 | Negative regulation of fibroblast proliferation | 3 | 29 | 0.0077 | TP53,BAX,GSTP1 |
| GO:0070570 | Regulation of neuron projection regeneration | 3 | 29 | 0.0077 | HGF,PTEN,SPP1 |
| GO:2000352 | Negative regulation of endothelial cell apoptotic process | 3 | 29 | 0.0077 | SERPINE1,TERT,NFE2L2 |
| GO:0017015 | Regulation of transforming growth factor beta receptor signaling pathway | 5 | 119 | 0.0078 | TGFB1,TP53,ADAM17,HSPA5,STK11 |
| GO:0030855 | Epithelial cell differentiation | 12 | 673 | 0.0078 | BMP4,TP63,NOTCH1,TYMS,MET,RARB,CTNNB1,AR,ERCC2,CDKN1A,HIF1A,VEGFA |
| GO:1900542 | Regulation of purine nucleotide metabolic process | 5 | 119 | 0.0078 | IFNG,STAT3,NOS2,PDK2,HIF1A |
| GO:1901575 | Organic substance catabolic process | 22 | 1750 | 0.0078 | TGFB1,MLH1,SIRT2,MDM2,ALDH1A1,OGG1,USP9X,HSPA5,NOS2,MTA1,CTNNB1,HMGB1,CASP8,MTOR,PTEN,UGT1A1,SPP1,NFE2L2,CD44,ZFP36,BIRC2,CYP1B1 |
| GO:0000019 | Regulation of mitotic recombination | 2 | 6 | 0.008 | MLH1,ERCC2 |
| GO:0006348 | Chromatin silencing at telomere | 2 | 6 | 0.008 | SIRT2,EZH2 |
| GO:0006925 | Inflammatory cell apoptotic process | 2 | 6 | 0.008 | FASLG,IL6 |
| GO:0007182 | Common-partner smad protein phosphorylation | 2 | 6 | 0.008 | TGFB1,BMP4 |
| GO:0015732 | Prostaglandin transport | 2 | 6 | 0.008 | NOS2,ABCC2 |
| GO:0036462 | TRAIL-activated apoptotic signaling pathway | 2 | 6 | 0.008 | TNFRSF10A,CASP8 |
| GO:0060398 | Regulation of growth hormone receptor signaling pathway | 2 | 6 | 0.008 | GDF15,JAK2 |
| GO:0060685 | Regulation of prostatic bud formation | 2 | 6 | 0.008 | BMP4,AR |
| GO:0070141 | Response to uv-a | 2 | 6 | 0.008 | CCND1,EGFR |
| GO:0072540 | T-helper 17 cell lineage commitment | 2 | 6 | 0.008 | STAT3,IL6 |
| GO:1902339 | Positive regulation of apoptotic process involved in morphogenesis | 2 | 6 | 0.008 | NOTCH1,BAX |
| GO:1903223 | Positive regulation of oxidative stress-induced neuron death | 2 | 6 | 0.008 | MCL1,TLR4 |
| GO:1903689 | Regulation of wound healing, spreading of epidermal cells | 2 | 6 | 0.008 | MTOR,PTEN |
| GO:1904683 | Regulation of metalloendopeptidase activity | 2 | 6 | 0.008 | STAT3,TIMP3 |
| GO:0040014 | Regulation of multicellular organism growth | 4 | 69 | 0.0081 | GDF15,PIK3CA,STAT3,BCL2 |
| GO:0051301 | Cell division | 10 | 493 | 0.0081 | CCND1,SIRT2,TP63,RB1,NOTCH1,BIRC5,USP9X,ERCC2,TOP2A,CDKN2A |
| GO:0006298 | Mismatch repair | 3 | 30 | 0.0083 | ERCC1,MLH1,TP73 |
| GO:0007143 | Female meiotic nuclear division | 3 | 30 | 0.0083 | MLH1,RAD51C,TOP2A |
| GO:0007435 | Salivary gland morphogenesis | 3 | 30 | 0.0083 | TGFB1,EGFR,TNF |
| GO:0009059 | Macromolecule biosynthetic process | 21 | 1643 | 0.0083 | CCND1,EPAS1,STAT3,TP53,EGFR,NOTCH1,BAX,TERT,TYMS,RARB,E2F1,PTEN,AR,RAD51,ERCC2,CDKN1A,TNF,BRCA1,EIF4E,HIF1A,MUC1 |
| GO:0032940 | Secretion by cell | 15 | 979 | 0.0083 | MAPK1,TGFB1,HGF,SERPINE1,TIMP3,PAK1,AXL,CXCR2,NOS2,HMGB1,XRCC5,GSTP1,IL6,CD44,VEGFA |
| GO:0035666 | TRIF-dependent toll-like receptor signaling pathway | 3 | 30 | 0.0083 | CASP8,TLR4,BIRC2 |
| GO:0045737 | Positive regulation of cyclin-dependent protein serine/threonine kinase activity | 3 | 30 | 0.0083 | CCND1,EGFR,ADAM17 |
| GO:0048593 | Camera-type eye morphogenesis | 5 | 121 | 0.0083 | BMP4,BAX,CTNNB1,HIF1A,VEGFA |
| GO:0048873 | Homeostasis of number of cells within a tissue | 3 | 30 | 0.0083 | NOTCH1,BAX,BCL2 |
| GO:0071542 | Dopaminergic neuron differentiation | 3 | 30 | 0.0083 | CTNNB1,HIF1A,VEGFA |
| GO:2000515 | Negative regulation of cd4-positive, alpha-beta t cell activation | 3 | 30 | 0.0083 | HMGB1,CD274,RUNX3 |
| GO:0001501 | Skeletal system development | 10 | 499 | 0.0087 | TGFB1,BMP4,TP63,TP53,TYMS,RARB,CTNNB1,RUNX3,CD44,HIF1A |
| GO:0001764 | Neuron migration | 5 | 123 | 0.0088 | BAX,AXL,USP9X,CTNNB1,VEGFA |
| GO:0003002 | Regionalization | 8 | 332 | 0.0089 | BMP4,ALDH1A2,TP63,TP53,NOTCH1,PRKDC,CTNNB1,BMI1 |
| GO:0030212 | Hyaluronan metabolic process | 3 | 31 | 0.0089 | TGFB1,HGF,CD44 |
| GO:0071333 | Cellular response to glucose stimulus | 4 | 71 | 0.0089 | LGALS1,NOX4,PIK3CA,IGF1R |
| GO:0071549 | Cellular response to dexamethasone stimulus | 3 | 31 | 0.0089 | EGFR,ABCC2,EIF4E |
| GO:0007281 | Germ cell development | 7 | 256 | 0.009 | ERCC1,MLH1,BMP4,BAX,CTNNB1,MTOR,BCL2 |
| GO:0034976 | Response to endoplasmic reticulum stress | 7 | 256 | 0.009 | CCND1,TP53,BAX,HSPA5,HDGF,NFE2L2,BCL2 |
| GO:0050821 | Protein stabilization | 6 | 186 | 0.009 | TP53,USP9X,PTEN,HSPD1,CDKN1A,CDKN2A |
| GO:0090101 | Negative regulation of transmembrane receptor protein serine/threonine kinase signaling pathway | 5 | 124 | 0.009 | TGFB1,TP53,NOTCH1,ADAM17,HSPA5 |
| GO:0008104 | Protein localization | 25 | 2139 | 0.0092 | TGFB1,BMP4,MDM2,CDH1,STAT3,RB1,TP53,EGFR,BAX,BIRC5,TERT,USP9X,EZH2,HSPA5,NOS2,BSG,CTNNB1,HMGB1,MCL1,HSPD1,XRCC5,YWHAZ,CDKN1A,TNF,EIF4E |
| GO:0001570 | Vasculogenesis | 4 | 72 | 0.0093 | TGFB1,NOTCH1,CTNNB1,VEGFA |
| GO:0034754 | Cellular hormone metabolic process | 5 | 125 | 0.0093 | ALDH1A2,ALDH1A1,UGT1A1,SPP1,CYP1B1 |
| GO:0001774 | Microglial cell activation | 3 | 32 | 0.0097 | IFNG,JAK2,TNF |
| GO:0038128 | ERBB2 signaling pathway | 3 | 32 | 0.0097 | PIK3CA,ERBB2,EGFR |
| GO:0071715 | Icosanoid transport | 3 | 32 | 0.0097 | NOS2,ABCC2,ABCC1 |
| GO:0010742 | Macrophage derived foam cell differentiation | 2 | 7 | 0.0099 | TGFB1,STAT1 |
| GO:0031077 | Post-embryonic camera-type eye development | 2 | 7 | 0.0099 | BAX,VEGFA |
| GO:0031622 | Positive regulation of fever generation | 2 | 7 | 0.0099 | PTGS2,TNF |
| GO:0032667 | Regulation of interleukin-23 production | 2 | 7 | 0.0099 | IFNG,TLR4 |
| GO:0035234 | Ectopic germ cell programmed cell death | 2 | 7 | 0.0099 | BAX,PRKDC |
| GO:0050882 | Voluntary musculoskeletal movement | 2 | 7 | 0.0099 | XRCC1,MTOR |
| GO:0071494 | Cellular response to uv-c | 2 | 7 | 0.0099 | MDM2,TP53 |
| GO:0090240 | Positive regulation of histone h4 acetylation | 2 | 7 | 0.0099 | BRCA1,MUC1 |
| GO:1904429 | Regulation of t-circle formation | 2 | 7 | 0.0099 | ERCC1,XRCC5 |
| GO:1904798 | Positive regulation of core promoter binding | 2 | 7 | 0.0099 | IFNG,CTNNB1 |
| GO:1990001 | Inhibition of cysteine-type endopeptidase activity involved in apoptotic process | 2 | 7 | 0.0099 | XIAP,BIRC2 |
| GO:0009954 | Proximal/distal pattern formation | 3 | 33 | 0.0103 | ALDH1A2,TP63,CTNNB1 |
| GO:0010831 | Positive regulation of myotube differentiation | 3 | 33 | 0.0103 | GDF15,MTOR,BCL2 |
| GO:0016242 | Negative regulation of macroautophagy | 3 | 33 | 0.0103 | PIK3CA,TP53,MTOR |
| GO:0034105 | Positive regulation of tissue remodeling | 3 | 33 | 0.0103 | EGFR,BAX,SPP1 |
| GO:0035115 | Embryonic forelimb morphogenesis | 3 | 33 | 0.0103 | ALDH1A2,TP63,CTNNB1 |
| GO:0042759 | Long-chain fatty acid biosynthetic process | 3 | 33 | 0.0103 | GSTM1,PTGS2,GSTP1 |
| GO:0002576 | Platelet degranulation | 5 | 129 | 0.0105 | TGFB1,HGF,SERPINE1,TIMP3,VEGFA |
| GO:0003231 | Cardiac ventricle development | 5 | 129 | 0.0105 | TGFB1,BMP4,MDM2,NOTCH1,HIF1A |
| GO:0030279 | Negative regulation of ossification | 4 | 75 | 0.0105 | NOTCH1,BCL2,TNF,HIF1A |
| GO:0071229 | Cellular response to acid chemical | 4 | 75 | 0.0105 | EGFR,MTOR,TNF,VEGFA |
| GO:0045995 | Regulation of embryonic development | 5 | 130 | 0.0108 | BMP4,NOTCH1,CTNNB1,AR,NFE2L2 |
| GO:0001101 | Response to acid chemical | 5 | 131 | 0.0111 | EGFR,MTOR,GSTP1,TNF,VEGFA |
| GO:0010837 | Regulation of keratinocyte proliferation | 3 | 34 | 0.0111 | TP63,TLR4,ZFP36 |
| GO:0010922 | Positive regulation of phosphatase activity | 3 | 34 | 0.0111 | IFNG,MTOR,JAK2 |
| GO:0032212 | Positive regulation of telomere maintenance via telomerase | 3 | 34 | 0.0111 | MAPK1,CTNNB1,XRCC5 |
| GO:0035633 | Maintenance of blood-brain barrier | 3 | 34 | 0.0111 | PTGS2,IL6,VEGFA |
| GO:0043029 | T cell homeostasis | 3 | 34 | 0.0111 | BAX,FAS,BCL2 |
| GO:0046685 | Response to arsenic-containing substance | 3 | 34 | 0.0111 | ABCC2,PTEN,CDKN1A |
| GO:0048536 | Spleen development | 3 | 34 | 0.0111 | ADAM17,PRKDC,BCL2 |
| GO:0048566 | Embryonic digestive tract development | 3 | 34 | 0.0111 | ALDH1A2,RARB,TNF |
| GO:0055023 | Positive regulation of cardiac muscle tissue growth | 3 | 34 | 0.0111 | MAPK1,NOTCH1,MTOR |
| GO:0070059 | Intrinsic apoptotic signaling pathway in response to endoplasmic reticulum stress | 3 | 34 | 0.0111 | TP53,BAX,BCL2 |
| GO:0007389 | Pattern specification process | 9 | 432 | 0.0114 | BMP4,ALDH1A2,TP63,TP53,NOTCH1,PRKDC,CTNNB1,BMI1,HIF1A |
| GO:0046887 | Positive regulation of hormone secretion | 5 | 132 | 0.0114 | EGFR,JAK2,SIRT3,SPP1,HIF1A |
| GO:0046890 | Regulation of lipid biosynthetic process | 6 | 197 | 0.0115 | IFNG,MTOR,PTGS2,SIRT3,TNF,BRCA1 |
| GO:0051302 | Regulation of cell division | 6 | 197 | 0.0115 | TGFB1,SIRT2,TP63,RB1,HDGF,VEGFA |
| GO:0072594 | Establishment of protein localization to organelle | 9 | 433 | 0.0116 | STAT3,TP53,BAX,TERT,USP9X,HSPA5,NOS2,HSPD1,CDKN1A |
| GO:0055080 | Cation homeostasis | 12 | 712 | 0.0117 | MAPK1,EPAS1,EGFR,BAX,CXCR2,HMGB1,FASLG,XIAP,JAK2,BCL2,PDK2,HIF1A |
| GO:0000083 | Regulation of transcription involved in g1/s transition of mitotic cell cycle | 3 | 35 | 0.0119 | RB1,TYMS,E2F1 |
| GO:0032364 | Oxygen homeostasis | 2 | 8 | 0.0119 | HIF1A,SOD2 |
| GO:0034983 | Peptidyl-lysine deacetylation | 2 | 8 | 0.0119 | SIRT2,SIRT3 |
| GO:0038114 | interleukin-21-mediated signaling pathway | 2 | 8 | 0.0119 | STAT3,STAT1 |
| GO:0045003 | Double-strand break repair via synthesis-dependent strand annealing | 2 | 8 | 0.0119 | RAD51,XRCC3 |
| GO:0050851 | Antigen receptor-mediated signaling pathway | 6 | 199 | 0.0119 | MAPK1,PIK3CA,PAK1,BAX,STK11,BCL2 |
| GO:0070244 | Negative regulation of thymocyte apoptotic process | 2 | 8 | 0.0119 | BMP4,HIF1A |
| GO:0071394 | Cellular response to testosterone stimulus | 2 | 8 | 0.0119 | AR,SPP1 |
| GO:0071493 | Cellular response to uv-b | 2 | 8 | 0.0119 | STK11,CDKN1A |
| GO:0071550 | Death-inducing signaling complex assembly | 2 | 8 | 0.0119 | CASP8,TNF |
| GO:0072080 | Nephron tubule development | 4 | 78 | 0.0119 | BMP4,NOTCH1,CTNNB1,BCL2 |
| GO:0072161 | Mesenchymal cell differentiation involved in kidney development | 2 | 8 | 0.0119 | BMP4,STAT1 |
| GO:0090400 | Stress-induced premature senescence | 2 | 8 | 0.0119 | TP53,CDKN1A |
| GO:1900222 | Negative regulation of amyloid-beta clearance | 2 | 8 | 0.0119 | IFNG,TNF |
| GO:1901533 | Negative regulation of hematopoietic progenitor cell differentiation | 2 | 8 | 0.0119 | NOTCH1,NFE2L2 |
| GO:1903376 | Regulation of oxidative stress-induced neuron intrinsic apoptotic signaling pathway | 2 | 8 | 0.0119 | MCL1,HIF1A |
| GO:0051146 | Striated muscle cell differentiation | 6 | 200 | 0.0122 | BMP4,NOX4,RB1,RARB,MTOR,VEGFA |
| GO:0030168 | Platelet activation | 5 | 135 | 0.0123 | MAPK1,PIK3CA,AXL,YWHAZ,IL6 |
| GO:0035304 | Regulation of protein dephosphorylation | 5 | 135 | 0.0123 | TGFB1,MTOR,IGFBP3,JAK2,TNF |
| GO:0031670 | Cellular response to nutrient | 3 | 36 | 0.0126 | MDM2,POSTN,PDK2 |
| GO:0033198 | Response to atp | 3 | 36 | 0.0126 | PTGS2,PTEN,HSPD1 |
| GO:0034645 | Cellular macromolecule biosynthetic process | 20 | 1592 | 0.0126 | CCND1,EPAS1,STAT3,TP53,EGFR,NOTCH1,BAX,TERT,TYMS,RARB,E2F1,PTEN,AR,RAD51,ERCC2,CDKN1A,BRCA1,EIF4E,HIF1A,MUC1 |
| GO:0048008 | Platelet-derived growth factor receptor signaling pathway | 3 | 36 | 0.0126 | PTEN,JAK2,VEGFA |
| GO:0051973 | Positive regulation of telomerase activity | 3 | 36 | 0.0126 | MAPK1,CTNNB1,XRCC5 |
| GO:0030512 | Negative regulation of transforming growth factor beta receptor signaling pathway | 4 | 81 | 0.0133 | TGFB1,TP53,ADAM17,HSPA5 |
| GO:0060326 | Cell chemotaxis | 6 | 204 | 0.0133 | HGF,CXCR2,HMGB1,ABCC1,IL6,VEGFA |
| GO:0006508 | Proteolysis | 17 | 1256 | 0.0134 | TGFB1,HGF,SIRT2,MDM2,TP53,ADAM17,USP9X,HSPA5,MTA1,CTNNB1,CASP8,PTEN,AR,NFE2L2,BRCA1,HIF1A,BIRC2 |
| GO:0009749 | Response to glucose | 5 | 138 | 0.0134 | LGALS1,NOX4,PIK3CA,IGF1R,PTEN |
| GO:0002701 | Negative regulation of production of molecular mediator of immune response | 3 | 37 | 0.0135 | TGFB1,PRKDC,TNF |
| GO:0006801 | Superoxide metabolic process | 3 | 37 | 0.0135 | NOX4,NOS2,SOD2 |
| GO:0010259 | Multicellular organism aging | 3 | 37 | 0.0135 | ERCC1,TP63,TP53 |
| GO:0032965 | Regulation of collagen biosynthetic process | 3 | 37 | 0.0135 | TGFB1,BMP4,IL6 |
| GO:0051154 | Negative regulation of striated muscle cell differentiation | 3 | 37 | 0.0135 | NOTCH1,PAK1,EZH2 |
| GO:0071276 | Cellular response to cadmium ion | 3 | 37 | 0.0135 | MAPK1,EGFR,OGG1 |
| GO:0035303 | Regulation of dephosphorylation | 6 | 205 | 0.0136 | TGFB1,IFNG,MTOR,IGFBP3,JAK2,TNF |
| GO:2000243 | Positive regulation of reproductive process | 4 | 82 | 0.0137 | SIRT2,CTNNB1,AR,VEGFA |
| GO:0003266 | Regulation of secondary heart field cardioblast proliferation | 2 | 9 | 0.0141 | NOTCH1,CTNNB1 |
| GO:0006265 | DNA topological change | 2 | 9 | 0.0141 | HMGB1,TOP2A |
| GO:0010835 | Regulation of protein adp-ribosylation | 2 | 9 | 0.0141 | IFNG,XRCC1 |
| GO:0038113 | interleukin-9-mediated signaling pathway | 2 | 9 | 0.0141 | STAT3,STAT1 |
| GO:0038155 | interleukin-23-mediated signaling pathway | 2 | 9 | 0.0141 | STAT3,JAK2 |
| GO:0043129 | Surfactant homeostasis | 2 | 9 | 0.0141 | EPAS1,VEGFA |
| GO:0045063 | T-helper 1 cell differentiation | 2 | 9 | 0.0141 | HMGB1,MTOR |
| GO:0048087 | Positive regulation of developmental pigmentation | 2 | 9 | 0.0141 | BAX,BCL2 |
| GO:0048477 | Oogenesis | 4 | 83 | 0.0141 | ERCC1,MLH1,CTNNB1,BCL2 |
| GO:0051103 | DNA ligation involved in DNA repair | 2 | 9 | 0.0141 | XRCC1,HMGB1 |
| GO:0051122 | Hepoxilin biosynthetic process | 2 | 9 | 0.0141 | GSTM1,GSTP1 |
| GO:0051593 | Response to folic acid | 2 | 9 | 0.0141 | OGG1,TYMS |
| GO:0060601 | Lateral sprouting from an epithelium | 2 | 9 | 0.0141 | TP63,AR |
| GO:0060693 | Regulation of branching involved in salivary gland morphogenesis | 2 | 9 | 0.0141 | HGF,TNF |
| GO:0060982 | Coronary artery morphogenesis | 2 | 9 | 0.0141 | NOTCH1,VEGFA |
| GO:0099527 | Postsynapse to nucleus signaling pathway | 2 | 9 | 0.0141 | STAT3,JAK2 |
| GO:0006984 | ER-nucleus signaling pathway | 3 | 38 | 0.0142 | TP53,HSPA5,NFE2L2 |
| GO:0044255 | Cellular lipid metabolic process | 14 | 939 | 0.0142 | CSNK2A1,SIRT2,ALDH1A2,PIK3CA,EGFR,BAX,ALDH1A1,GSTM1,PTGS2,PTEN,UGT1A1,GSTP1,BRCA1,CYP1B1 |
| GO:0090049 | Regulation of cell migration involved in sprouting angiogenesis | 3 | 38 | 0.0142 | NOTCH1,PTGS2,VEGFA |
| GO:1900744 | Regulation of p38mapk cascade | 3 | 38 | 0.0142 | HGF,DUSP1,VEGFA |
| GO:0072527 | Pyrimidine-containing compound metabolic process | 4 | 84 | 0.0147 | MAPK1,OGG1,TYMS,MTOR |
| GO:0006909 | Phagocytosis | 6 | 210 | 0.0149 | MAPK1,PIK3CA,PAK1,AXL,HMGB1,TLR4 |
| GO:0002443 | Leukocyte mediated immunity | 11 | 641 | 0.0151 | ERCC1,MAPK1,MLH1,ADAM17,CXCR2,HMGB1,HSPD1,XRCC5,GSTP1,IL6,CD44 |
| GO:0006693 | Prostaglandin metabolic process | 3 | 39 | 0.0151 | GSTM1,PTGS2,GSTP1 |
| GO:0033574 | Response to testosterone | 3 | 39 | 0.0151 | DUSP1,AR,SPP1 |
| GO:1900371 | Regulation of purine nucleotide biosynthetic process | 3 | 39 | 0.0151 | STAT3,NOS2,PDK2 |
| GO:1904646 | Cellular response to amyloid-beta | 3 | 39 | 0.0151 | IGF1R,TLR4,ABCC1 |
| GO:1903035 | Negative regulation of response to wounding | 4 | 85 | 0.0152 | SERPINE1,PTEN,SPP1,TNF |
| GO:0009057 | Macromolecule catabolic process | 15 | 1058 | 0.0157 | TGFB1,MLH1,SIRT2,MDM2,USP9X,HSPA5,MTA1,CTNNB1,HMGB1,CASP8,MTOR,NFE2L2,CD44,ZFP36,BIRC2 |
| GO:0001657 | Ureteric bud development | 4 | 86 | 0.0158 | BMP4,RARB,CTNNB1,BCL2 |
| GO:0035315 | Hair cell differentiation | 3 | 40 | 0.0161 | NOTCH1,CTNNB1,ERCC2 |
| GO:0009952 | Anterior/posterior pattern specification | 6 | 214 | 0.0162 | BMP4,ALDH1A2,TP53,NOTCH1,PRKDC,CTNNB1 |
| GO:0000280 | Nuclear division | 7 | 291 | 0.0164 | MLH1,RB1,BIRC5,ERCC4,RAD51C,RAD51,TOP2A |
| GO:0007440 | Foregut morphogenesis | 2 | 10 | 0.0164 | NOTCH1,CTNNB1 |
| GO:0010749 | Regulation of nitric oxide mediated signal transduction | 2 | 10 | 0.0164 | EGFR,VEGFA |
| GO:0045132 | Meiotic chromosome segregation | 4 | 87 | 0.0164 | MLH1,ERCC4,RAD51C,TOP2A |
| GO:0045792 | Negative regulation of cell size | 2 | 10 | 0.0164 | MTOR,PTEN |
| GO:0048103 | Somatic stem cell division | 2 | 10 | 0.0164 | NOTCH1,CDKN2A |
| GO:0060068 | Vagina development | 2 | 10 | 0.0164 | BAX,AXL |
| GO:0060828 | Regulation of canonical wnt signaling pathway | 7 | 291 | 0.0164 | TGFB1,EGFR,NOTCH1,SOX2,STK11,CTNNB1,XIAP |
| GO:0071481 | Cellular response to x-ray | 2 | 10 | 0.0164 | TP53BP1,XRCC5 |
| GO:0072111 | Cell proliferation involved in kidney development | 2 | 10 | 0.0164 | BMP4,STAT1 |
| GO:1901532 | Regulation of hematopoietic progenitor cell differentiation | 4 | 87 | 0.0164 | NOTCH1,PRKDC,TP73,NFE2L2 |
| GO:1902749 | Regulation of cell cycle g2/m phase transition | 6 | 215 | 0.0164 | CCND1,TP53,RAD51C,CDKN1A,CDKN2A,BRCA1 |
| GO:2000048 | Negative regulation of cell-cell adhesion mediated by cadherin | 2 | 10 | 0.0164 | NOTCH1,VEGFA |
| GO:0051649 | Establishment of localization in cell | 26 | 2375 | 0.0165 | MAPK1,TGFB1,HGF,SERPINE1,MLH1,STAT3,TIMP3,TP53,BAX,BIRC5,USP9X,CXCR2,HSPA5,NOS2,CTNNB1,HMGB1,FASLG,HSPD1,XRCC5,YWHAZ,GSTP1,CDKN1A,CD44,EIF4E,HIF1A,VEGFA |
| GO:0050806 | Positive regulation of synaptic transmission | 5 | 147 | 0.0167 | MAPK1,EGFR,PTGS2,PTEN,TNF |
| GO:0006284 | Base-excision repair | 3 | 41 | 0.0169 | APEX1,XRCC1,OGG1 |
| GO:0007566 | Embryo implantation | 3 | 41 | 0.0169 | BSG,PTGS2,SPP1 |
| GO:0030901 | Midbrain development | 4 | 88 | 0.0169 | SIRT2,CXCR2,HSPA5,CTNNB1 |
| GO:0045684 | Positive regulation of epidermis development | 3 | 41 | 0.0169 | BMP4,NOTCH1,TNF |
| GO:0050853 | B cell receptor signaling pathway | 3 | 41 | 0.0169 | MAPK1,BAX,BCL2 |
| GO:0060260 | Regulation of transcription initiation from rna polymerase ii promoter | 3 | 41 | 0.0169 | ERCC1,TP53,HMGB1 |
| GO:1904037 | Positive regulation of epithelial cell apoptotic process | 3 | 41 | 0.0169 | FASLG,JAK2,IL6 |
| GO:1901565 | Organonitrogen compound catabolic process | 15 | 1070 | 0.0171 | TGFB1,SIRT2,MDM2,OGG1,USP9X,HSPA5,NOS2,MTA1,CTNNB1,CASP8,MTOR,UGT1A1,NFE2L2,CD44,BIRC2 |
| GO:0002718 | Regulation of cytokine production involved in immune response | 4 | 89 | 0.0174 | TGFB1,TLR4,IL6,TNF |
| GO:0003158 | Endothelium development | 4 | 89 | 0.0174 | BMP4,NOTCH1,MET,CTNNB1 |
| GO:0051492 | Regulation of stress fiber assembly | 4 | 89 | 0.0174 | NOX4,PAK1,MET,MTOR |
| GO:0001709 | Cell fate determination | 3 | 42 | 0.0179 | BMP4,CTNNB1,MCL1 |
| GO:0045616 | Regulation of keratinocyte differentiation | 3 | 42 | 0.0179 | TP63,NOTCH1,ZFP36 |
| GO:0046825 | Regulation of protein export from nucleus | 3 | 42 | 0.0179 | MDM2,TP53,CDKN2A |
| GO:0098761 | Cellular response to interleukin-7 | 3 | 42 | 0.0179 | STAT3,HDGF,HSPD1 |
| GO:2000144 | Positive regulation of dna-templated transcription, initiation | 3 | 42 | 0.0179 | ERCC1,TP53,CTNNB1 |
| GO:0030509 | BMP signaling pathway | 4 | 90 | 0.018 | TGFB1,BMP4,GDF15,USP9X |
| GO:0043470 | Regulation of carbohydrate catabolic process | 4 | 90 | 0.018 | IFNG,STAT3,TP53,HIF1A |
| GO:0006629 | Lipid metabolic process | 16 | 1190 | 0.0184 | CSNK2A1,SIRT2,ALDH1A2,PIK3CA,EGFR,BAX,ALDH1A1,GSTM1,PTGS2,PTEN,UGT1A1,SPP1,GSTP1,BRCA1,SLC16A1,CYP1B1 |
| GO:0006304 | DNA modification | 4 | 91 | 0.0187 | APEX1,MGMT,OGG1,EZH2 |
| GO:0008585 | Female gonad development | 4 | 91 | 0.0187 | BAX,HSPA5,BCL2,VEGFA |
| GO:0030890 | Positive regulation of b cell proliferation | 3 | 43 | 0.0188 | TLR4,BCL2,CDKN1A |
| GO:0033327 | Leydig cell differentiation | 2 | 11 | 0.0188 | CCND1,AR |
| GO:0045124 | Regulation of bone resorption | 3 | 43 | 0.0188 | EGFR,SPP1,IL6 |
| GO:0045945 | Positive regulation of transcription by rna polymerase iii | 2 | 11 | 0.0188 | MTOR,AR |
| GO:0060272 | Embryonic skeletal joint morphogenesis | 2 | 11 | 0.0188 | BMP4,CTNNB1 |
| GO:0060525 | Prostate glandular acinus development | 2 | 11 | 0.0188 | TP63,NOTCH1 |
| GO:0097011 | Cellular response to granulocyte macrophage colony-stimulating factor stimulus | 2 | 11 | 0.0188 | MAPK1,ZFP36 |
| GO:1903054 | Negative regulation of extracellular matrix organization | 2 | 11 | 0.0188 | TGFB1,NOTCH1 |
| GO:0050764 | Regulation of phagocytosis | 4 | 92 | 0.0192 | IFNG,HMGB1,PTEN,TNF |
| GO:1903900 | Regulation of viral life cycle | 5 | 153 | 0.0193 | LGALS1,NOTCH1,BCL2,TNF,TOP2A |
| GO:0050890 | Cognition | 7 | 302 | 0.0195 | MAPK1,EGFR,MTOR,PTGS2,PTEN,TNF,HIF1A |
| GO:0001658 | Branching involved in ureteric bud morphogenesis | 3 | 44 | 0.0199 | BMP4,CTNNB1,BCL2 |
| GO:0003197 | Endocardial cushion development | 3 | 44 | 0.0199 | BMP4,MDM2,NOTCH1 |
| GO:0006953 | Acute-phase response | 3 | 44 | 0.0199 | STAT3,UGT1A1,IL6 |
| GO:0007596 | Blood coagulation | 7 | 303 | 0.0199 | MAPK1,PIK3CA,AXL,RAD51C,JAK2,YWHAZ,IL6 |
| GO:0016575 | Histone deacetylation | 3 | 44 | 0.0199 | SIRT2,MTA1,SIRT3 |
| GO:0032715 | Negative regulation of interleukin-6 production | 3 | 44 | 0.0199 | HGF,TLR4,TNF |
| GO:0035307 | Positive regulation of protein dephosphorylation | 3 | 44 | 0.0199 | TGFB1,MTOR,JAK2 |
| GO:0060425 | Lung morphogenesis | 3 | 44 | 0.0199 | MAPK1,BMP4,CTNNB1 |
| GO:0061005 | Cell differentiation involved in kidney development | 3 | 44 | 0.0199 | BMP4,NOTCH1,STAT1 |
| GO:0070167 | Regulation of biomineral tissue development | 4 | 93 | 0.0199 | TGFB1,BMP4,NOTCH1,HIF1A |
| GO:1901184 | Regulation of erbb signaling pathway | 4 | 94 | 0.0206 | ERBB2,EGFR,ADAM17,FASLG |
| GO:0010799 | Regulation of peptidyl-threonine phosphorylation | 3 | 45 | 0.021 | MAPK1,TGFB1,SIRT2 |
| GO:0014003 | Oligodendrocyte development | 3 | 45 | 0.021 | PTEN,ERCC2,GSTP1 |
| GO:0046849 | Bone remodeling | 3 | 45 | 0.021 | TGFB1,NOX4,CTNNB1 |
| GO:0048255 | mRNA stabilization | 3 | 45 | 0.021 | E2F1,MTOR,ZFP36 |
| GO:0062207 | Regulation of pattern recognition receptor signaling pathway | 4 | 95 | 0.0212 | HMGB1,XIAP,TLR4,BIRC2 |
| GO:0006983 | ER overload response | 2 | 12 | 0.0214 | TP53,HSPA5 |
| GO:0033148 | Positive regulation of intracellular estrogen receptor signaling pathway | 2 | 12 | 0.0214 | PAK1,AR |
| GO:0033598 | Mammary gland epithelial cell proliferation | 2 | 12 | 0.0214 | MAPK1,CCND1 |
| GO:0036499 | PERK-mediated unfolded protein response | 2 | 12 | 0.0214 | HSPA5,NFE2L2 |
| GO:0045475 | Locomotor rhythm | 2 | 12 | 0.0214 | MTA1,PTEN |
| GO:0046689 | Response to mercury ion | 2 | 12 | 0.0214 | BSG,ABCC2 |
| GO:0051573 | Negative regulation of histone h3-k9 methylation | 2 | 12 | 0.0214 | KDM4C,BRCA1 |
| GO:0051712 | Positive regulation of killing of cells of other organism | 2 | 12 | 0.0214 | IFNG,NOS2 |
| GO:0060736 | Prostate gland growth | 2 | 12 | 0.0214 | PTEN,AR |
| GO:0061154 | Endothelial tube morphogenesis | 2 | 12 | 0.0214 | BMP4,CTNNB1 |
| GO:0090656 | T-circle formation | 2 | 12 | 0.0214 | ERCC1,XRCC3 |
| GO:1990440 | Positive regulation of transcription from rna polymerase ii promoter in response to endoplasmic reticulum stress | 2 | 12 | 0.0214 | TP53,HSPA5 |
| GO:2000304 | Positive regulation of ceramide biosynthetic process | 2 | 12 | 0.0214 | SIRT3,TNF |
| GO:0090288 | Negative regulation of cellular response to growth factor stimulus | 5 | 158 | 0.0215 | TGFB1,TP53,NOTCH1,ADAM17,HSPA5 |
| GO:0010769 | Regulation of cell morphogenesis involved in differentiation | 7 | 309 | 0.0216 | PAK1,PRKDC,STK11,PTEN,POSTN,SPP1,VEGFA |
| GO:0006636 | Unsaturated fatty acid biosynthetic process | 3 | 46 | 0.022 | GSTM1,PTGS2,GSTP1 |
| GO:0007369 | Gastrulation | 5 | 159 | 0.022 | DUSP1,BMP4,TP53,SOX2,CTNNB1 |
| GO:0007595 | Lactation | 3 | 46 | 0.022 | CCND1,HIF1A,VEGFA |
| GO:0042572 | Retinol metabolic process | 3 | 46 | 0.022 | ALDH1A2,ALDH1A1,CYP1B1 |
| GO:0060976 | Coronary vasculature development | 3 | 46 | 0.022 | BMP4,NOTCH1,VEGFA |
| GO:0002429 | Immune response-activating cell surface receptor signaling pathway | 7 | 311 | 0.0223 | MAPK1,PIK3CA,PAK1,BAX,STK11,BCL2,MUC1 |
| GO:0120162 | Positive regulation of cold-induced thermogenesis | 4 | 97 | 0.0224 | EPAS1,IGF1R,JAK2,VEGFA |
| GO:0021915 | Neural tube development | 5 | 160 | 0.0225 | TGFB1,BMP4,ALDH1A2,NOTCH1,HIF1A |
| GO:0002274 | Myeloid leukocyte activation | 10 | 585 | 0.023 | MAPK1,IFNG,CXCR2,HMGB1,TLR4,JAK2,XRCC5,GSTP1,CD44,TNF |
| GO:0007179 | Transforming growth factor beta receptor signaling pathway | 4 | 98 | 0.0231 | TGFB1,GDF15,TP53,USP9X |
| GO:0035722 | interleukin-12-mediated signaling pathway | 3 | 47 | 0.0231 | IFNG,JAK2,SOD2 |
| GO:0043124 | Negative regulation of i-kappab kinase/nf-kappab signaling | 3 | 47 | 0.0231 | CASP8,STAT1,GSTP1 |
| GO:0043370 | Regulation of cd4-positive, alpha-beta t cell differentiation | 3 | 47 | 0.0231 | IFNG,HMGB1,RUNX3 |
| GO:0050829 | Defense response to gram-negative bacterium | 4 | 98 | 0.0231 | SERPINE1,NOS2,TLR4,IL6 |
| GO:1903580 | Positive regulation of atp metabolic process | 3 | 47 | 0.0231 | IFNG,STAT3,HIF1A |
| GO:0006887 | Exocytosis | 12 | 789 | 0.0235 | MAPK1,TGFB1,HGF,SERPINE1,TIMP3,PAK1,CXCR2,HMGB1,XRCC5,GSTP1,CD44,VEGFA |
| GO:0045069 | Regulation of viral genome replication | 4 | 99 | 0.0239 | NOTCH1,BCL2,TNF,TOP2A |
| GO:0001711 | Endodermal cell fate commitment | 2 | 13 | 0.024 | SOX2,CTNNB1 |
| GO:0002839 | Positive regulation of immune response to tumor cell | 2 | 13 | 0.024 | CD274,HSPD1 |
| GO:0006809 | Nitric oxide biosynthetic process | 2 | 13 | 0.024 | NOS2,CYP1B1 |
| GO:0007494 | Midgut development | 2 | 13 | 0.024 | ALDH1A2,EGFR |
| GO:0010838 | Positive regulation of keratinocyte proliferation | 2 | 13 | 0.024 | TP63,TLR4 |
| GO:0032700 | Negative regulation of interleukin-17 production | 2 | 13 | 0.024 | IFNG,TLR4 |
| GO:0035278 | miRNA mediated inhibition of translation | 2 | 13 | 0.024 | STAT3,ZFP36 |
| GO:0042635 | Positive regulation of hair cycle | 2 | 13 | 0.024 | TERT,TNF |
| GO:0042762 | Regulation of sulfur metabolic process | 2 | 13 | 0.024 | NFE2L2,PDK2 |
| GO:0043568 | Positive regulation of insulin-like growth factor receptor signaling pathway | 2 | 13 | 0.024 | AR,IGFBP3 |
| GO:0048641 | Regulation of skeletal muscle tissue development | 3 | 48 | 0.024 | TGFB1,CTNNB1,BCL2 |
| GO:0048715 | Negative regulation of oligodendrocyte differentiation | 2 | 13 | 0.024 | NOTCH1,CTNNB1 |
| GO:0051259 | Protein complex oligomerization | 6 | 236 | 0.024 | ALDH1A2,TP63,TP53,TP73,TP53BP1,SOD2 |
| GO:0072077 | Renal vesicle morphogenesis | 2 | 13 | 0.024 | BMP4,CTNNB1 |
| GO:0072182 | Regulation of nephron tubule epithelial cell differentiation | 2 | 13 | 0.024 | CTNNB1,STAT1 |
| GO:1903894 | Regulation of ire1-mediated unfolded protein response | 2 | 13 | 0.024 | BAX,HSPA5 |
| GO:1990000 | Amyloid fibril formation | 2 | 13 | 0.024 | MDM2,CDKN2A |
| GO:0032436 | Positive regulation of proteasomal ubiquitin-dependent protein catabolic process | 4 | 100 | 0.0243 | SIRT2,MDM2,RB1,NFE2L2 |
| GO:0090277 | Positive regulation of peptide hormone secretion | 4 | 100 | 0.0243 | EGFR,JAK2,SIRT3,HIF1A |
| GO:0032874 | Positive regulation of stress-activated mapk cascade | 5 | 164 | 0.0244 | PAK1,HMGB1,TLR4,TNF,VEGFA |
| GO:0044057 | Regulation of system process | 10 | 592 | 0.0244 | HGF,MDM2,EPAS1,EGFR,NOTCH1,PAK1,MTOR,PTGS2,PTEN,JAK2 |
| GO:0061024 | Membrane organization | 12 | 796 | 0.0247 | STAT3,TP63,TP53,EGFR,BAX,E2F1,CASP8,PTEN,TP73,YWHAZ,BCL2,ABCC1 |
| GO:0034504 | Protein localization to nucleus | 5 | 165 | 0.0249 | BMP4,MDM2,STAT3,TP53,CDKN1A |
| GO:0009408 | Response to heat | 4 | 101 | 0.0251 | PTGS2,ABCC2,HSPD1,CDKN1A |
| GO:0010862 | Positive regulation of pathway-restricted smad protein phosphorylation | 3 | 49 | 0.0252 | TGFB1,BMP4,GDF15 |
| GO:0097194 | Execution phase of apoptosis | 3 | 49 | 0.0252 | HMGB1,CASP8,TOP2A |
| GO:0045055 | Regulated exocytosis | 11 | 697 | 0.0257 | MAPK1,TGFB1,HGF,SERPINE1,TIMP3,CXCR2,HMGB1,XRCC5,GSTP1,CD44,VEGFA |
| GO:0045621 | Positive regulation of lymphocyte differentiation | 4 | 102 | 0.0258 | IFNG,AXL,PRKDC,RUNX3 |
| GO:0043161 | Proteasome-mediated ubiquitin-dependent protein catabolic process | 7 | 323 | 0.0264 | SIRT2,MDM2,HSPA5,MTA1,CTNNB1,NFE2L2,BIRC2 |
| GO:0001961 | Positive regulation of cytokine-mediated signaling pathway | 3 | 50 | 0.0265 | AXL,ADAM17,HIF1A |
| GO:0035094 | Response to nicotine | 3 | 50 | 0.0265 | MAPK1,BCL2,TNF |
| GO:0045747 | Positive regulation of notch signaling pathway | 3 | 50 | 0.0265 | STAT3,TP63,NOTCH1 |
| GO:1990090 | Cellular response to nerve growth factor stimulus | 3 | 50 | 0.0265 | HSPA5,E2F1,PTEN |
| GO:0010763 | Positive regulation of fibroblast migration | 2 | 14 | 0.0266 | TGFB1,PAK1 |
| GO:0021781 | Glial cell fate commitment | 2 | 14 | 0.0266 | SOX2,CTNNB1 |
| GO:0033147 | Negative regulation of intracellular estrogen receptor signaling pathway | 2 | 14 | 0.0266 | TP63,BRCA1 |
| GO:0042574 | Retinal metabolic process | 2 | 14 | 0.0266 | ALDH1A2,CYP1B1 |
| GO:0045898 | Regulation of rna polymerase ii transcription preinitiation complex assembly | 2 | 14 | 0.0266 | TP53,HMGB1 |
| GO:0048681 | Negative regulation of axon regeneration | 2 | 14 | 0.0266 | PTEN,SPP1 |
| GO:0051547 | Regulation of keratinocyte migration | 2 | 14 | 0.0266 | MTOR,PTEN |
| GO:0051645 | Golgi localization | 2 | 14 | 0.0266 | STK11,YWHAZ |
| GO:0051974 | Negative regulation of telomerase activity | 2 | 14 | 0.0266 | TP53,ERCC4 |
| GO:0060253 | Negative regulation of glial cell proliferation | 2 | 14 | 0.0266 | NOTCH1,TERT |
| GO:0060397 | Growth hormone receptor signaling pathway via jak-stat | 2 | 14 | 0.0266 | STAT3,JAK2 |
| GO:0060546 | Negative regulation of necroptotic process | 2 | 14 | 0.0266 | CASP8,BIRC2 |
| GO:0072044 | Collecting duct development | 2 | 14 | 0.0266 | BMP4,NOTCH1 |
| GO:0090266 | Regulation of mitotic cell cycle spindle assembly checkpoint | 2 | 14 | 0.0266 | DUSP1,XRCC3 |
| GO:1901201 | Regulation of extracellular matrix assembly | 2 | 14 | 0.0266 | TGFB1,NOTCH1 |
| GO:1902916 | Positive regulation of protein polyubiquitination | 2 | 14 | 0.0266 | XIAP,BIRC2 |
| GO:0006885 | Regulation of ph | 4 | 104 | 0.0272 | MAPK1,FASLG,BCL2,PDK2 |
| GO:0033559 | Unsaturated fatty acid metabolic process | 4 | 104 | 0.0272 | GSTM1,PTGS2,GSTP1,CYP1B1 |
| GO:0002011 | Morphogenesis of an epithelial sheet | 3 | 51 | 0.0274 | NOTCH1,ADAM17,CD44 |
| GO:2001238 | Positive regulation of extrinsic apoptotic signaling pathway | 3 | 51 | 0.0274 | TNFSF10,TIMP3,PTEN |
| GO:0009266 | Response to temperature stimulus | 5 | 170 | 0.0275 | CASP8,PTGS2,ABCC2,HSPD1,CDKN1A |
| GO:0032886 | Regulation of microtubule-based process | 6 | 245 | 0.0277 | ERBB2,PAK1,MET,CTNNB1,BRCA1,XRCC3 |
| GO:0010921 | Regulation of phosphatase activity | 5 | 171 | 0.0281 | IFNG,MTOR,IGFBP3,JAK2,TNF |
| GO:0001706 | Endoderm formation | 3 | 52 | 0.0288 | DUSP1,SOX2,CTNNB1 |
| GO:0030968 | Endoplasmic reticulum unfolded protein response | 4 | 106 | 0.0288 | CCND1,HSPA5,HDGF,NFE2L2 |
| GO:0031648 | Protein destabilization | 3 | 52 | 0.0288 | MDM2,PRKDC,CDKN2A |
| GO:0001667 | Ameboidal-type cell migration | 5 | 173 | 0.0294 | PIK3CA,PTEN,HIF1A,CYP1B1,VEGFA |
| GO:0009404 | Toxin metabolic process | 2 | 15 | 0.0295 | NFE2L2,CYP1B1 |
| GO:0010935 | Regulation of macrophage cytokine production | 2 | 15 | 0.0295 | TGFB1,TLR4 |
| GO:0033151 | V(D)J recombination | 2 | 15 | 0.0295 | PRKDC,HMGB1 |
| GO:0043518 | Negative regulation of dna damage response, signal transduction by p53 class mediator | 2 | 15 | 0.0295 | MDM2,CD44 |
| GO:0060044 | Negative regulation of cardiac muscle cell proliferation | 2 | 15 | 0.0295 | PTEN,TP73 |
| GO:0071361 | Cellular response to ethanol | 2 | 15 | 0.0295 | PTEN,UGT1A1 |
| GO:0090343 | Positive regulation of cell aging | 2 | 15 | 0.0295 | TP53,CDKN2A |
| GO:1900225 | Regulation of nlrp3 inflammasome complex assembly | 2 | 15 | 0.0295 | SIRT2,TLR4 |
| GO:1903140 | Regulation of establishment of endothelial barrier | 2 | 15 | 0.0295 | TNF,VEGFA |
| GO:2001028 | Positive regulation of endothelial cell chemotaxis | 2 | 15 | 0.0295 | MET,VEGFA |
| GO:0001541 | Ovarian follicle development | 3 | 53 | 0.03 | BAX,BCL2,VEGFA |
| GO:0032835 | Glomerulus development | 3 | 53 | 0.03 | BMP4,NOTCH1,BCL2 |
| GO:0051496 | Positive regulation of stress fiber assembly | 3 | 53 | 0.03 | NOX4,PAK1,MTOR |
| GO:0001523 | Retinoid metabolic process | 4 | 108 | 0.0304 | ALDH1A2,ALDH1A1,UGT1A1,CYP1B1 |
| GO:0044344 | Cellular response to fibroblast growth factor stimulus | 4 | 108 | 0.0304 | MAPK1,POSTN,CD44,ZFP36 |
| GO:0010469 | Regulation of signaling receptor activity | 5 | 176 | 0.0312 | SERPINE1,IFNG,ADAM17,HMGB1,PTEN |
| GO:0021761 | Limbic system development | 4 | 109 | 0.0313 | XRCC1,BAX,EZH2,PTEN |
| GO:0010611 | Regulation of cardiac muscle hypertrophy | 3 | 54 | 0.0314 | NOTCH1,PAK1,MTOR |
| GO:0071320 | Cellular response to camp | 3 | 54 | 0.0314 | APEX1,NOX4,HSPA5 |
| GO:0018105 | Peptidyl-serine phosphorylation | 5 | 177 | 0.0318 | MAPK1,CSNK2A1,PRKDC,MTOR,BCL2 |
| GO:0001759 | Organ induction | 2 | 16 | 0.0325 | BMP4,CTNNB1 |
| GO:0010715 | Regulation of extracellular matrix disassembly | 2 | 16 | 0.0325 | TGFB1,IL6 |
| GO:0030214 | Hyaluronan catabolic process | 2 | 16 | 0.0325 | TGFB1,CD44 |
| GO:0033033 | Negative regulation of myeloid cell apoptotic process | 2 | 16 | 0.0325 | CXCR2,BCL2 |
| GO:0033599 | Regulation of mammary gland epithelial cell proliferation | 2 | 16 | 0.0325 | CCND1,BAX |
| GO:0034116 | Positive regulation of heterotypic cell-cell adhesion | 2 | 16 | 0.0325 | CD44,TNF |
| GO:0035729 | Cellular response to hepatocyte growth factor stimulus | 2 | 16 | 0.0325 | HGF,SIRT2 |
| GO:0051044 | Positive regulation of membrane protein ectodomain proteolysis | 2 | 16 | 0.0325 | IFNG,TNF |
| GO:0070198 | Protein localization to chromosome, telomeric region | 2 | 16 | 0.0325 | TERT,XRCC5 |
| GO:0099170 | Postsynaptic modulation of chemical synaptic transmission | 2 | 16 | 0.0325 | CDH1,EIF4E |
| GO:1902894 | Negative regulation of pri-mirna transcription by rna polymerase ii | 2 | 16 | 0.0325 | TGFB1,BMP4 |
| GO:0006081 | Cellular aldehyde metabolic process | 3 | 55 | 0.0326 | ALDH1A2,ALDH1A1,CYP1B1 |
| GO:0043030 | Regulation of macrophage activation | 3 | 55 | 0.0326 | TLR4,HSPD1,IL6 |
| GO:0055114 | Oxidation-reduction process | 13 | 939 | 0.0326 | APEX1,ALDH1A2,NOX4,ALDH1A1,NOS2,MTOR,PTGS2,KDM4C,SIRT3,GPX3,GSTP1,SOD2,CYP1B1 |
| GO:0001676 | Long-chain fatty acid metabolic process | 4 | 111 | 0.0329 | GSTM1,PTGS2,GSTP1,CYP1B1 |
| GO:0042552 | Myelination | 4 | 111 | 0.0329 | SIRT2,ERBB2,PTEN,ERCC2 |
| GO:0002931 | Response to ischemia | 3 | 56 | 0.0341 | TP53,HSPD1,BCL2 |
| GO:0043392 | Negative regulation of dna binding | 3 | 56 | 0.0341 | ERCC4,E2F1,JAK2 |
| GO:0046324 | Regulation of glucose import | 3 | 56 | 0.0341 | TERT,NFE2L2,TNF |
| GO:0007611 | Learning or memory | 6 | 259 | 0.0348 | MAPK1,EGFR,MTOR,PTGS2,PTEN,HIF1A |
| GO:0043666 | Regulation of phosphoprotein phosphatase activity | 4 | 113 | 0.0348 | MTOR,IGFBP3,JAK2,TNF |
| GO:0055065 | Metal ion homeostasis | 10 | 629 | 0.0348 | EPAS1,EGFR,BAX,CXCR2,HMGB1,FASLG,XIAP,JAK2,BCL2,HIF1A |
| GO:0008217 | Regulation of blood pressure | 5 | 182 | 0.0352 | NOS2,PTGS2,AR,POSTN,SOD2 |
| GO:0030177 | Positive regulation of wnt signaling pathway | 5 | 182 | 0.0352 | CSNK2A1,TGFB1,EGFR,TERT,XIAP |
| GO:0035194 | Post-transcriptional gene silencing by rna | 3 | 57 | 0.0355 | STAT3,TERT,ZFP36 |
| GO:0003184 | Pulmonary valve morphogenesis | 2 | 17 | 0.0356 | BMP4,NOTCH1 |
| GO:0007096 | Regulation of exit from mitosis | 2 | 17 | 0.0356 | SIRT2,BIRC5 |
| GO:0032740 | Positive regulation of interleukin-17 production | 2 | 17 | 0.0356 | TGFB1,IL6 |
| GO:0043200 | Response to amino acid | 4 | 114 | 0.0356 | EGFR,MTOR,GSTP1,TNF |
| GO:0072189 | Ureter development | 2 | 17 | 0.0356 | BMP4,ALDH1A2 |
| GO:0008361 | Regulation of cell size | 5 | 183 | 0.0357 | PAK1,MTOR,PTEN,SPP1,VEGFA |
| GO:0043488 | Regulation of mrna stability | 5 | 183 | 0.0357 | APEX1,E2F1,MTOR,YWHAZ,ZFP36 |
| GO:0072330 | Monocarboxylic acid biosynthetic process | 5 | 183 | 0.0357 | ALDH1A2,GSTM1,PTGS2,GSTP1,BRCA1 |
| GO:0050922 | Negative regulation of chemotaxis | 3 | 58 | 0.0369 | DUSP1,NOTCH1,GSTP1 |
| GO:0051438 | Regulation of ubiquitin-protein transferase activity | 3 | 58 | 0.0369 | PTEN,BMI1,CDKN2A |
| GO:2000242 | Negative regulation of reproductive process | 3 | 58 | 0.0369 | DUSP1,BMP4,STK11 |
| GO:0001704 | Formation of primary germ layer | 4 | 116 | 0.0376 | DUSP1,BMP4,SOX2,CTNNB1 |
| GO:0016570 | Histone modification | 7 | 351 | 0.0383 | SIRT2,EZH2,MTA1,BMI1,KDM4C,JAK2,SIRT3 |
| GO:0002040 | Sprouting angiogenesis | 3 | 59 | 0.0385 | BMP4,NOTCH1,VEGFA |
| GO:0021885 | Forebrain cell migration | 3 | 59 | 0.0385 | EGFR,AXL,CTNNB1 |
| GO:0060395 | SMAD protein signal transduction | 3 | 59 | 0.0385 | TGFB1,BMP4,GDF15 |
| GO:0007498 | Mesoderm development | 4 | 117 | 0.0386 | BMP4,TP63,JAK2,VEGFA |
| GO:0050770 | Regulation of axonogenesis | 5 | 187 | 0.0386 | PAK1,STK11,PTEN,SPP1,VEGFA |
| GO:0002902 | Regulation of b cell apoptotic process | 2 | 18 | 0.0389 | BAX,PTEN |
| GO:0007221 | Positive regulation of transcription of notch receptor target | 2 | 18 | 0.0389 | NOTCH1,STAT1 |
| GO:0030224 | Monocyte differentiation | 2 | 18 | 0.0389 | BMP4,VEGFA |
| GO:0031065 | Positive regulation of histone deacetylation | 2 | 18 | 0.0389 | TP53,VEGFA |
| GO:0031293 | Membrane protein intracellular domain proteolysis | 2 | 18 | 0.0389 | TGFB1,ADAM17 |
| GO:0042026 | Protein refolding | 2 | 18 | 0.0389 | HSPA5,HSPD1 |
| GO:0044320 | Cellular response to leptin stimulus | 2 | 18 | 0.0389 | STAT3,PTEN |
| GO:0048384 | Retinoic acid receptor signaling pathway | 2 | 18 | 0.0389 | ALDH1A2,RARB |
| GO:0051023 | Regulation of immunoglobulin secretion | 2 | 18 | 0.0389 | IL6,TNF |
| GO:0051571 | Positive regulation of histone h3-k4 methylation | 2 | 18 | 0.0389 | CTNNB1,BRCA1 |
| GO:0061050 | Regulation of cell growth involved in cardiac muscle cell development | 2 | 18 | 0.0389 | PAK1,MTOR |
| GO:0061298 | Retina vasculature development in camera-type eye | 2 | 18 | 0.0389 | HIF1A,CYP1B1 |
| GO:0072202 | Cell differentiation involved in metanephros development | 2 | 18 | 0.0389 | BMP4,STAT1 |
| GO:2001185 | Regulation of cd8-positive, alpha-beta t cell activation | 2 | 18 | 0.0389 | CD274,RUNX3 |
| GO:0006633 | Fatty acid biosynthetic process | 4 | 118 | 0.0392 | GSTM1,PTGS2,GSTP1,BRCA1 |
| GO:0010906 | Regulation of glucose metabolic process | 4 | 118 | 0.0392 | TP53,MTOR,IGFBP3,PDK2 |
| GO:0032720 | Negative regulation of tumor necrosis factor production | 3 | 60 | 0.0397 | AXL,TLR4,GSTP1 |
| GO:0034605 | Cellular response to heat | 3 | 60 | 0.0397 | PTGS2,HSPD1,CDKN1A |
| GO:0035690 | Cellular response to drug | 3 | 60 | 0.0397 | TP53,HSPA5,NOS2 |
| GO:0045454 | Cell redox homeostasis | 3 | 60 | 0.0397 | APEX1,NOS2,NFE2L2 |
| GO:0046605 | Regulation of centrosome cycle | 3 | 60 | 0.0397 | CTNNB1,BRCA1,XRCC3 |
| GO:0071806 | Protein transmembrane transport | 3 | 60 | 0.0397 | HSPA5,MCL1,HSPD1 |
| GO:0006914 | Autophagy | 6 | 270 | 0.0409 | CSNK2A1,SIRT2,TP53,STK11,HMGB1,CDKN2A |
| GO:0007219 | Notch signaling pathway | 4 | 120 | 0.0413 | TP63,NOTCH1,ADAM17,STAT1 |
| GO:0048844 | Artery morphogenesis | 3 | 61 | 0.0413 | BMP4,NOTCH1,VEGFA |
| GO:0002407 | Dendritic cell chemotaxis | 2 | 19 | 0.0421 | CXCR2,HMGB1 |
| GO:0009219 | Pyrimidine deoxyribonucleotide metabolic process | 2 | 19 | 0.0421 | OGG1,TYMS |
| GO:0010042 | Response to manganese ion | 2 | 19 | 0.0421 | HSPA5,PTGS2 |
| GO:0032026 | Response to magnesium ion | 2 | 19 | 0.0421 | CCND1,MDM2 |
| GO:0033189 | Response to vitamin a | 2 | 19 | 0.0421 | ALDH1A2,TYMS |
| GO:0045780 | Positive regulation of bone resorption | 2 | 19 | 0.0421 | EGFR,SPP1 |
| GO:0050765 | Negative regulation of phagocytosis | 2 | 19 | 0.0421 | HMGB1,PTEN |
| GO:0070507 | Regulation of microtubule cytoskeleton organization | 5 | 192 | 0.0421 | PAK1,MET,CTNNB1,BRCA1,XRCC3 |
| GO:0071498 | Cellular response to fluid shear stress | 2 | 19 | 0.0421 | PTGS2,NFE2L2 |
| GO:0072012 | Glomerulus vasculature development | 2 | 19 | 0.0421 | BMP4,NOTCH1 |
| GO:0060627 | Regulation of vesicle-mediated transport | 9 | 550 | 0.0424 | MAPK1,SERPINE1,IFNG,NOTCH1,AXL,HMGB1,PTEN,TNF,VEGFA |
| GO:0043687 | Post-translational protein modification | 7 | 360 | 0.0425 | LGALS1,BMP4,EPAS1,IGFBP3,SPP1,IL6,HIF1A |
| GO:0055006 | Cardiac cell development | 3 | 62 | 0.0427 | NOTCH1,MTOR,VEGFA |
| GO:1903078 | Positive regulation of protein localization to plasma membrane | 3 | 62 | 0.0427 | IFNG,EGFR,TNF |
| GO:1903902 | Positive regulation of viral life cycle | 3 | 62 | 0.0427 | LGALS1,NOTCH1,TOP2A |
| GO:1903578 | Regulation of atp metabolic process | 4 | 122 | 0.0432 | IFNG,STAT3,TP53,HIF1A |
| GO:0006959 | Humoral immune response | 6 | 275 | 0.0439 | IFNG,NOTCH1,PDCD1,BCL2,IL6,TNF |
| GO:0007411 | Axon guidance | 6 | 275 | 0.0439 | MAPK1,PIK3CA,ERBB2,NOTCH1,BSG,VEGFA |
| GO:0001838 | Embryonic epithelial tube formation | 4 | 123 | 0.0443 | TGFB1,BMP4,CTNNB1,HIF1A |
| GO:0007178 | Transmembrane receptor protein serine/threonine kinase signaling pathway | 5 | 195 | 0.0444 | TGFB1,BMP4,GDF15,TP53,USP9X |
| GO:0042742 | Defense response to bacterium | 6 | 277 | 0.0452 | SERPINE1,ADAM17,NOS2,TLR4,IL6,TNF |
| GO:0002068 | Glandular epithelial cell development | 2 | 20 | 0.0455 | BMP4,RARB |
| GO:0009299 | mRNA transcription | 2 | 20 | 0.0455 | STAT3,TP53 |
| GO:0032682 | Negative regulation of chemokine production | 2 | 20 | 0.0455 | GSTP1,IL6 |
| GO:0051412 | Response to corticosterone | 2 | 20 | 0.0455 | CCND1,CDKN1A |
| GO:1902004 | Positive regulation of amyloid-beta formation | 2 | 20 | 0.0455 | IFNG,TNF |
| GO:1904996 | Positive regulation of leukocyte adhesion to vascular endothelial cell | 2 | 20 | 0.0455 | IL6,TNF |
| GO:0048857 | Neural nucleus development | 3 | 64 | 0.046 | SIRT2,HSPA5,BCL2 |
| GO:0005996 | Monosaccharide metabolic process | 5 | 198 | 0.0468 | PIK3CA,ALDH1A1,UGT1A1,TNF,PDK2 |
| GO:0010389 | Regulation of g2/m transition of mitotic cell cycle | 5 | 198 | 0.0468 | CCND1,RAD51C,CDKN1A,CDKN2A,BRCA1 |
| GO:1902905 | Positive regulation of supramolecular fiber organization | 5 | 198 | 0.0468 | NOX4,RB1,PAK1,MET,MTOR |
| GO:0016070 | RNA metabolic process | 18 | 1584 | 0.0477 | APEX1,CCND1,MLH1,EPAS1,STAT3,TP53,NOTCH1,BAX,TERT,PRKDC,RARB,E2F1,PTEN,AR,ERCC2,CDKN1A,CDKN2A,ZFP36 |
| GO:0001649 | Osteoblast differentiation | 4 | 127 | 0.0488 | BMP4,SOX2,IGFBP3,SPP1 |
| GO:0009799 | Specification of symmetry | 4 | 127 | 0.0488 | BMP4,ALDH1A2,NOTCH1,HIF1A |
| GO:0001756 | Somitogenesis | 3 | 66 | 0.0492 | ALDH1A2,TP53,PRKDC |
| GO:0010888 | Negative regulation of lipid storage | 2 | 21 | 0.0492 | IL6,TNF |
| GO:0022010 | Central nervous system myelination | 2 | 21 | 0.0492 | PTEN,ERCC2 |
| GO:0032516 | Positive regulation of phosphoprotein phosphatase activity | 2 | 21 | 0.0492 | MTOR,JAK2 |
| GO:0042698 | Ovulation cycle | 3 | 66 | 0.0492 | EGFR,AXL,HSPA5 |
| GO:0043371 | Negative regulation of cd4-positive, alpha-beta t cell differentiation | 2 | 21 | 0.0492 | HMGB1,RUNX3 |
| GO:0045943 | Positive regulation of transcription by rna polymerase i | 2 | 21 | 0.0492 | ERBB2,MTOR |
| GO:0046827 | Positive regulation of protein export from nucleus | 2 | 21 | 0.0492 | MDM2,TP53 |
| GO:0048485 | Sympathetic nervous system development | 2 | 21 | 0.0492 | TP63,CTNNB1 |
| GO:0051797 | Regulation of hair follicle development | 2 | 21 | 0.0492 | CTNNB1,TNF |
| GO:0051966 | Regulation of synaptic transmission, glutamatergic | 3 | 66 | 0.0492 | EGFR,PTGS2,TNF |
| GO:0090026 | Positive regulation of monocyte chemotaxis | 2 | 21 | 0.0492 | SERPINE1,HMGB1 |
| GO:0090050 | Positive regulation of cell migration involved in sprouting angiogenesis | 2 | 21 | 0.0492 | PTGS2,VEGFA |
| GO:1901687 | Glutathione derivative biosynthetic process | 2 | 21 | 0.0492 | GSTM1,GSTP1 |
| GO:1903204 | Negative regulation of oxidative stress-induced neuron death | 2 | 21 | 0.0492 | CTNNB1,HIF1A |
| GO:1905208 | Negative regulation of cardiocyte differentiation | 2 | 21 | 0.0492 | EGFR,PAK1 |
| GO:2000178 | Negative regulation of neural precursor cell proliferation | 2 | 21 | 0.0492 | SIRT2,TP53 |
| GO:0010508 | Positive regulation of autophagy | 4 | 128 | 0.0494 | IFNG,STK11,HMGB1,HIF1A |

**S7: Top ten Hub genes via 12 different topological analysis methods**

| Betweenness | Bottle neck | Closeness | Clustering coefficient | Degree | DMNC | EcCentricity | EPC | MCC | MNC | Radiality | Stress |
| --- | --- | --- | --- | --- | --- | --- | --- | --- | --- | --- | --- |
| TP53 | CDKN2A | TP53 | ABCC2 | TP53 | MET | TNF | TP53 | TP53 | TP53 | TP53 | TP53 |
| EGFR | TP53 | EGFR | HDGF | EGFR | AXL | CDH1 | PTEN | CTNNB1 | EGFR | EGFR | EGFR |
| HIF1A | TNF | CTNNB1 | SLC16A1 | CTNNB1 | IGF1R | STAT3 | HIF1A | STAT3 | CTNNB1 | CTNNB1 | CTNNB1 |
| CTNNB1 | VEGFA | PTEN | KDM4C | PTEN | HGF | AR | EGFR | CDH1 | PTEN | PTEN | HIF1A |
| TNF | IL6 | STAT3 | PAK1 | STAT3 | MUC1 | CTNNB1 | STAT3 | HIF1A | STAT3 | STAT3 | PTEN |
| PTEN | CTNNB1 | HIF1A | CXCR2 | HIF1A | ERCC4 | TERT | CTNNB1 | PTEN | HIF1A | HIF1A | TNF |
| VEGFA | HIF1A | VEGFA | ERCC5 | VEGFA | ERCC5 | VEGFA | CCND1 | EGFR | VEGFA | VEGFA | VEGFA |
| MTOR | EGFR | CCND1 | ERCC4 | CCND1 | IGFBP3 | PIK3CA | VEGFA | ERBB2 | CCND1 | CCND1 | STAT3 |
| IL6 | UGT1A1 | TNF | GDF15 | TNF | JAK2 | CCND1 | CDH1 | VEGFA | TNF | TNF | IL6 |
| STAT3 | CD44 | ERBB2 | AXL | IL6 | BMP4 | EZH2 | IL6 | CCND1 | IL6 | ERBB2 | ERBB2 |

**S8: Cytohubba calculation using 12 different topological analysis methods**

| Betweenness | BottleNeck | Closeness | ClusteringCoefficient | Degree | DMNC | EcCentricity | EPC | MCC | MNC | Radiality | Stress |
| --- | --- | --- | --- | --- | --- | --- | --- | --- | --- | --- | --- |
| 0 | 1 | 49.41667 | 0 | 1 | 0 | 0.25 | 1.492 | 1 | 1 | 2.5339 | 0 |
| 7.25963 | 1 | 65.66667 | 0.82857 | 15 | 0.87129 | 0.33333 | 8.385 | 5.08E+07 | 15 | 3.12712 | 170 |
| 1.77199 | 1 | 58.33333 | 0.78571 | 8 | 0.64146 | 0.33333 | 4.202 | 792 | 8 | 2.87288 | 20 |
| 0.28571 | 1 | 55.16667 | 0.66667 | 3 | 0.30898 | 0.33333 | 2.484 | 4 | 3 | 2.79661 | 2 |
| 0.64006 | 1 | 56.33333 | 0.73333 | 6 | 0.52304 | 0.33333 | 3.724 | 122 | 6 | 2.80508 | 14 |
| 0 | 1 | 57.16667 | 1 | 3 | 0.46346 | 0.33333 | 2.225 | 6 | 3 | 2.89831 | 0 |
| 0 | 1 | 57.5 | 1 | 4 | 0.56839 | 0.33333 | 3.627 | 24 | 4 | 2.89831 | 0 |
| 16.40863 | 1 | 68.66667 | 0.69474 | 20 | 0.81063 | 0.33333 | 10.205 | 1.05E+09 | 20 | 3.19492 | 240 |
| 10.13559 | 1 | 67.66667 | 0.70588 | 18 | 0.79334 | 0.33333 | 8.314 | 5.12E+07 | 18 | 3.17797 | 180 |
| 1.23672 | 1 | 65.5 | 0.89524 | 15 | 0.9414 | 0.33333 | 8.832 | 5.59E+08 | 15 | 3.11864 | 22 |
| 8.29286 | 1 | 65 | 0.55238 | 15 | 0.58086 | 0.33333 | 7.384 | 6726 | 15 | 3.09322 | 104 |
| 1.29786 | 1 | 61 | 0.71429 | 7 | 0.54881 | 0.33333 | 4.468 | 150 | 7 | 3.02542 | 18 |
| 1.00785 | 1 | 64.5 | 0.92381 | 15 | 0.97144 | 0.33333 | 7.682 | 1.04E+09 | 15 | 3.0678 | 16 |
| 25.26379 | 2 | 67.33333 | 0.82353 | 18 | 0.92557 | 0.33333 | 9.689 | 1.75E+11 | 18 | 3.16102 | 274 |
| 1.0677 | 1 | 66.83333 | 0.92647 | 17 | 1.02002 | 0.33333 | 9.39 | 9.40E+10 | 17 | 3.15254 | 24 |
| 0 | 1 | 64.16667 | 1 | 12 | 0.96591 | 0.33333 | 7.918 | 4.79E+08 | 12 | 3.10169 | 0 |
| 8.71979 | 1 | 69 | 0.74762 | 21 | 0.88742 | 0.33333 | 9.553 | 1.29E+10 | 21 | 3.19492 | 136 |
| 0.30159 | 1 | 63.66667 | 0.94545 | 11 | 0.88234 | 0.33333 | 6.381 | 1088640 | 11 | 3.09322 | 6 |
| 13.68216 | 2 | 67.33333 | 0.78431 | 18 | 0.88149 | 0.33333 | 8.512 | 7.66E+09 | 18 | 3.16102 | 310 |
| 17.36415 | 1 | 72.16667 | 0.74644 | 27 | 0.96601 | 0.33333 | 11.359 | 9.22E+13 | 27 | 3.25424 | 250 |
| 35.64746 | 1 | 76.83333 | 0.67302 | 36 | 0.95863 | 0.33333 | 14.668 | 9.22E+13 | 36 | 3.33898 | 498 |
| 0 | 1 | 53.08333 | 1 | 2 | 0.30779 | 0.25 | 1.913 | 2 | 2 | 2.70339 | 0 |
| 45.28258 | 1 | 78.83333 | 0.67564 | 40 | 0.99611 | 0.33333 | 16.567 | 9.22E+13 | 40 | 3.37288 | 692 |
| 22.35656 | 2 | 62.5 | 0.76364 | 11 | 0.71266 | 0.33333 | 6.398 | 85682 | 11 | 3.0339 | 228 |
| 5.28894 | 1 | 61.83333 | 0.53571 | 8 | 0.43736 | 0.33333 | 5.271 | 54 | 8 | 3.05085 | 66 |
| 27.41638 | 1 | 74.83333 | 0.69355 | 32 | 0.95017 | 0.33333 | 13.49 | 9.22E+13 | 32 | 3.30508 | 408 |
| 37.4783 | 1 | 74.33333 | 0.67527 | 31 | 0.91541 | 0.33333 | 12.91 | 9.22E+13 | 31 | 3.29661 | 448 |
| 123.5712 | 3 | 82.5 | 0.57817 | 47 | 0.89808 | 0.5 | 16.808 | 9.22E+13 | 47 | 3.44068 | 1566 |
| 16.2408 | 1 | 68.16667 | 0.83626 | 19 | 0.9582 | 0.33333 | 10.126 | 2.09E+13 | 19 | 3.18644 | 388 |
| 1.33899 | 1 | 63.33333 | 0.81818 | 11 | 0.76356 | 0.33333 | 6.701 | 367944 | 11 | 3.07627 | 24 |
| 4.4925 | 1 | 67.16667 | 0.81699 | 18 | 0.91822 | 0.33333 | 8.915 | 6.14E+08 | 18 | 3.15254 | 66 |
| 12.6091 | 1 | 69.5 | 0.72727 | 22 | 0.87739 | 0.33333 | 10.035 | 7.10E+09 | 22 | 3.20339 | 180 |
| 14.47043 | 1 | 71 | 0.76333 | 25 | 0.96236 | 0.33333 | 11.332 | 4.83E+11 | 25 | 3.22881 | 188 |
| 0.58265 | 1 | 62.66667 | 0.91111 | 10 | 0.81806 | 0.33333 | 5.623 | 85680 | 10 | 3.05932 | 12 |
| 64.06727 | 1 | 80.33333 | 0.63677 | 43 | 0.96111 | 0.33333 | 16.513 | 9.22E+13 | 43 | 3.39831 | 844 |
| 17.41974 | 1 | 68.66667 | 0.61429 | 21 | 0.72915 | 0.33333 | 9.443 | 1.21E+07 | 21 | 3.17797 | 218 |
| 38.69385 | 2 | 76.83333 | 0.67143 | 36 | 0.95637 | 0.33333 | 15.07 | 9.22E+13 | 36 | 3.33898 | 554 |
| 11.14887 | 1 | 72.66667 | 0.77513 | 28 | 1.01554 | 0.33333 | 12.122 | 2.31E+13 | 28 | 3.26271 | 204 |
| 20.21766 | 1 | 72.33333 | 0.66138 | 28 | 0.86651 | 0.33333 | 11.889 | 4.68E+09 | 28 | 3.24576 | 292 |
| 4.87681 | 1 | 68.5 | 0.81053 | 20 | 0.94574 | 0.33333 | 9.684 | 1.87E+11 | 20 | 3.18644 | 98 |
| 4.93731 | 1 | 69.16667 | 0.81818 | 22 | 0.98706 | 0.33333 | 9.547 | 4.66E+09 | 22 | 3.18644 | 90 |
| 12.68663 | 1 | 69.83333 | 0.74704 | 23 | 0.91522 | 0.33333 | 10.885 | 1.32E+12 | 23 | 3.20339 | 180 |
| 38.57192 | 2 | 78.83333 | 0.71538 | 40 | 1.05471 | 0.33333 | 16.981 | 9.22E+13 | 40 | 3.37288 | 524 |
| 38.80093 | 1 | 69.66667 | 0.72294 | 22 | 0.87216 | 0.33333 | 10.229 | 1.87E+11 | 22 | 3.21186 | 408 |
| 35.54184 | 1 | 78.83333 | 0.69359 | 40 | 1.02258 | 0.33333 | 16.657 | 9.22E+13 | 40 | 3.37288 | 552 |
| 17.57873 | 1 | 75.83333 | 0.77184 | 34 | 1.07888 | 0.33333 | 13.838 | 9.22E+13 | 34 | 3.32203 | 316 |
| 1.53885 | 1 | 69.33333 | 0.92857 | 21 | 1.1022 | 0.33333 | 10.949 | 9.22E+13 | 21 | 3.21186 | 34 |
| 0 | 1 | 55 | 1 | 3 | 0.46346 | 0.33333 | 2.6 | 6 | 3 | 2.78814 | 0 |
| 183.3014 | 14 | 89.5 | 0.51913 | 61 | 0.87632 | 0.5 | 19.947 | 9.22E+13 | 61 | 3.55932 | 2478 |
| 93.38845 | 1 | 84.33333 | 0.58196 | 51 | 0.92797 | 0.33333 | 19.163 | 9.22E+13 | 51 | 3.4661 | 1336 |
| 10.77049 | 1 | 72.16667 | 0.81766 | 27 | 1.05819 | 0.33333 | 12.509 | 9.22E+13 | 27 | 3.25424 | 184 |
| 44.17329 | 1 | 79.83333 | 0.67015 | 42 | 1.00381 | 0.33333 | 16.305 | 9.22E+13 | 42 | 3.38983 | 652 |
| 24.51723 | 1 | 75.5 | 0.7178 | 33 | 0.99349 | 0.5 | 14.555 | 9.22E+13 | 33 | 3.32203 | 400 |
| 171.9556 | 1 | 85.5 | 0.55951 | 53 | 0.9032 | 0.5 | 18.843 | 9.22E+13 | 53 | 3.49153 | 2406 |
| 355.0566 | 2 | 87.83333 | 0.54144 | 58 | 0.92648 | 0.33333 | 19.983 | 9.22E+13 | 57 | 3.52542 | 3312 |
| 101.0935 | 1 | 83.83333 | 0.58122 | 50 | 0.92094 | 0.33333 | 18.199 | 9.22E+13 | 50 | 3.45763 | 1254 |
| 15.91048 | 1 | 72.66667 | 0.74868 | 28 | 0.98088 | 0.33333 | 12.375 | 9.22E+13 | 28 | 3.26271 | 286 |
| 27.10557 | 1 | 73.33333 | 0.67734 | 29 | 0.89796 | 0.33333 | 13.559 | 9.22E+13 | 29 | 3.27966 | 402 |
| 149.2728 | 2 | 83.5 | 0.58759 | 49 | 0.92501 | 0.5 | 17.786 | 9.22E+13 | 49 | 3.45763 | 1774 |
| 26.51753 | 1 | 78.33333 | 0.74629 | 39 | 1.09123 | 0.33333 | 16.201 | 9.22E+13 | 39 | 3.36441 | 442 |
| 19.79163 | 1 | 73.16667 | 0.73399 | 29 | 0.97306 | 0.33333 | 13.137 | 9.22E+13 | 29 | 3.27119 | 324 |
| 99.06838 | 1 | 84 | 0.57143 | 50 | 0.90542 | 0.5 | 18.438 | 9.22E+13 | 50 | 3.4661 | 1438 |
| 88.5645 | 1 | 80 | 0.65621 | 42 | 0.98293 | 0.5 | 16.432 | 9.22E+13 | 42 | 3.39831 | 1214 |
| 14.69915 | 1 | 68.66667 | 0.74704 | 23 | 0.91522 | 0.33333 | 8.555 | 1.41E+12 | 23 | 3.14407 | 184 |
| 17.52362 | 1 | 68.16667 | 0.72294 | 22 | 0.87216 | 0.33333 | 8.339 | 1.41E+12 | 22 | 3.13559 | 176 |
| 0.54384 | 1 | 65.5 | 0.96324 | 17 | 1.06049 | 0.33333 | 6.744 | 1.41E+12 | 17 | 3.08475 | 10 |
| 40.00824 | 1 | 71.83333 | 0.60582 | 28 | 0.79372 | 0.33333 | 11.594 | 1.42E+12 | 28 | 3.22034 | 450 |
| 13.75985 | 1 | 68.66667 | 0.75494 | 23 | 0.9249 | 0.33333 | 8.98 | 1.42E+12 | 23 | 3.14407 | 172 |
| 90.82447 | 1 | 79 | 0.56098 | 41 | 0.83373 | 0.33333 | 15.611 | 9.22E+13 | 41 | 3.36441 | 1122 |
| 47.99186 | 1 | 74 | 0.63656 | 31 | 0.86293 | 0.33333 | 11.722 | 1.42E+12 | 31 | 3.27966 | 638 |
| 28.32247 | 1 | 73.5 | 0.66897 | 30 | 0.89699 | 0.33333 | 12.257 | 7.50E+09 | 30 | 3.27119 | 476 |
| 0.18333 | 1 | 65 | 0.98333 | 16 | 1.05896 | 0.33333 | 6.792 | 1.39E+12 | 16 | 3.07627 | 4 |
| 12.33039 | 1 | 69.16667 | 0.78788 | 22 | 0.9505 | 0.33333 | 7.956 | 1.41E+12 | 22 | 3.18644 | 228 |
| 69.56267 | 1 | 75.83333 | 0.5916 | 35 | 0.83489 | 0.33333 | 12.024 | 1.42E+12 | 35 | 3.30508 | 724 |
| 80.35301 | 1 | 77.5 | 0.56472 | 38 | 0.81876 | 0.33333 | 14.135 | 2.90E+12 | 38 | 3.33898 | 1040 |
| 43.30188 | 1 | 72.33333 | 0.6069 | 30 | 0.81376 | 0.33333 | 11.116 | 1.32E+12 | 30 | 3.21186 | 400 |
| 206.4586 | 1 | 88.83333 | 0.49096 | 60 | 0.82445 | 0.33333 | 18.725 | 9.22E+13 | 60 | 3.54237 | 2374 |
| 10.03455 | 1 | 69.16667 | 0.72857 | 21 | 0.86481 | 0.33333 | 11.001 | 1.92E+10 | 21 | 3.20339 | 146 |
| 97.4402 | 1 | 76 | 0.56684 | 34 | 0.79234 | 0.5 | 14.163 | 2.16E+13 | 34 | 3.33051 | 1138 |
| 104.383 | 1 | 78.66667 | 0.50897 | 40 | 0.75039 | 0.33333 | 14.125 | 1.35E+11 | 40 | 3.36441 | 1144 |
| 68.05544 | 2 | 72.33333 | 0.52991 | 27 | 0.6858 | 0.33333 | 10.607 | 3.40E+09 | 27 | 3.26271 | 842 |
| 10.36659 | 1 | 68 | 0.75325 | 22 | 0.90872 | 0.33333 | 8.107 | 1.31E+12 | 22 | 3.12712 | 118 |
| 9.20953 | 1 | 65.33333 | 0.70476 | 15 | 0.7411 | 0.33333 | 7.495 | 792006 | 15 | 3.11017 | 114 |
| 160.5821 | 2 | 81.33333 | 0.56061 | 45 | 0.85868 | 0.33333 | 15.624 | 9.22E+13 | 45 | 3.41525 | 1958 |
| 68.83479 | 1 | 73.66667 | 0.55632 | 30 | 0.74595 | 0.33333 | 10.972 | 1.40E+12 | 30 | 3.27966 | 784 |
| 23.82956 | 1 | 66 | 0.66667 | 15 | 0.70104 | 0.33333 | 7.447 | 217442 | 15 | 3.14407 | 272 |
| 25.65015 | 1 | 69.16667 | 0.86667 | 21 | 1.02872 | 0.33333 | 10.644 | 9.22E+13 | 21 | 3.20339 | 308 |
| 57.12165 | 2 | 78.33333 | 0.65047 | 39 | 0.95112 | 0.33333 | 16.556 | 9.22E+13 | 39 | 3.36441 | 680 |
| 11.46118 | 1 | 71.33333 | 0.80308 | 26 | 1.02609 | 0.33333 | 12.935 | 9.22E+13 | 26 | 3.22881 | 164 |
| 10.70159 | 1 | 72.16667 | 0.82336 | 27 | 1.06556 | 0.33333 | 12.738 | 9.22E+13 | 27 | 3.25424 | 178 |
| 310.6253 | 2 | 90.5 | 0.46749 | 63 | 0.79725 | 0.5 | 19.398 | 9.22E+13 | 63 | 3.57627 | 3088 |
| 373.36 | 1 | 98 | 0.42624 | 78 | 0.77741 | 0.5 | 22.486 | 9.22E+13 | 78 | 3.70339 | 4434 |
| 13.78814 | 1 | 76.83333 | 0.79683 | 36 | 1.13498 | 0.33333 | 14.967 | 9.22E+13 | 36 | 3.33898 | 288 |
| 3.62932 | 1 | 56.5 | 0.4 | 5 | 0.25931 | 0.33333 | 2.125 | 8 | 5 | 2.83051 | 18 |
| 230.3867 | 1 | 93.5 | 0.47997 | 69 | 0.84235 | 0.5 | 21.47 | 9.22E+13 | 69 | 3.62712 | 2948 |
| 349.61 | 5 | 92.83333 | 0.44337 | 68 | 0.77456 | 0.33333 | 20.639 | 9.22E+13 | 68 | 3.61017 | 4040 |
| 680.8836 | 4 | 97.33333 | 0.41046 | 77 | 0.7456 | 0.33333 | 22.309 | 9.22E+13 | 77 | 3.68644 | 5888 |
| 370.47 | 8 | 96 | 0.43169 | 74 | 0.77447 | 0.5 | 20.999 | 9.22E+13 | 74 | 3.66949 | 4158 |
| 56.44339 | 2 | 78.33333 | 0.59649 | 39 | 0.87219 | 0.33333 | 14.869 | 9.22E+13 | 39 | 3.36441 | 734 |
| 650.0535 | 4 | 99 | 0.39873 | 80 | 0.73302 | 0.5 | 21.884 | 9.22E+13 | 80 | 3.72034 | 6146 |
| 97.2217 | 1 | 85.83333 | 0.59119 | 54 | 0.96007 | 0.33333 | 18.991 | 9.22E+13 | 54 | 3.49153 | 1542 |
| 20.79314 | 1 | 69.5 | 0.67965 | 22 | 0.81994 | 0.33333 | 10.923 | 1.25E+10 | 22 | 3.20339 | 306 |
| 337.4484 | 1 | 97.5 | 0.42891 | 77 | 0.77913 | 0.5 | 21.994 | 9.22E+13 | 77 | 3.69492 | 4058 |
| 5.14332 | 1 | 66 | 0.75238 | 15 | 0.79117 | 0.33333 | 8.204 | 4400640 | 15 | 3.14407 | 98 |
| 259.0147 | 1 | 92 | 0.48531 | 66 | 0.8399 | 0.5 | 20.933 | 9.22E+13 | 66 | 3.60169 | 3052 |
| 2.14885 | 1 | 64.5 | 0.85714 | 14 | 0.87837 | 0.33333 | 8.269 | 7.99E+07 | 14 | 3.08475 | 38 |
| 61.02814 | 1 | 66.33333 | 0.43382 | 17 | 0.47763 | 0.33333 | 6.473 | 10512 | 17 | 3.12712 | 752 |
| 0 | 1 | 50.5 | 1 | 5 | 0.64826 | 0.25 | 2.442 | 120 | 5 | 2.51695 | 0 |
| 378.5324 | 11 | 93 | 0.43503 | 68 | 0.75999 | 0.5 | 20.619 | 9.22E+13 | 68 | 3.61864 | 4220 |
| 123.4002 | 3 | 86.33333 | 0.56027 | 55 | 0.91518 | 0.33333 | 18.68 | 9.22E+13 | 55 | 3.5 | 1666 |
| 178.829 | 2 | 74.16667 | 0.42796 | 31 | 0.58015 | 0.33333 | 10.958 | 1.98E+07 | 31 | 3.28814 | 2122 |
| 34.74934 | 3 | 62.83333 | 0.45455 | 11 | 0.4242 | 0.33333 | 4.153 | 266 | 11 | 3.05085 | 330 |
| 106.4967 | 1 | 72 | 0.55556 | 27 | 0.71898 | 0.33333 | 10.219 | 2.12E+08 | 27 | 3.24576 | 1382 |
| 37.71736 | 1 | 68.5 | 0.73684 | 20 | 0.85976 | 0.33333 | 9.242 | 1.49E+12 | 20 | 3.18644 | 390 |
| 929.7102 | 4 | 101.5 | 0.37087 | 85 | 0.69483 | 0.5 | 22.176 | 9.22E+13 | 85 | 3.76271 | 7808 |
| 289.5782 | 1 | 93 | 0.47103 | 68 | 0.82288 | 0.5 | 20.564 | 9.22E+13 | 68 | 3.61864 | 3694 |
| 1514.885 | 13 | 111 | 0.31273 | 104 | 0.62382 | 0.5 | 24.21 | 9.22E+13 | 104 | 3.92373 | 10022 |
| 107.7068 | 1 | 73.33333 | 0.61379 | 30 | 0.82301 | 0.33333 | 10.095 | 1.42E+12 | 30 | 3.26271 | 1308 |
| 4.73271 | 1 | 63.5 | 0.68889 | 10 | 0.61853 | 0.33333 | 5.415 | 1086 | 10 | 3.10169 | 64 |

**S9: P-value for expression and survival analysis of hub genes**

| Genes | P-value expression | P-value survival |
| --- | --- | --- |
| VEGFA | <1E-12 | 0.73 |
| CTNNB1 | 1.74E-02 | 0.91 |
| EGFR | 2.35E-12 | 0.4 |
| HIF1A | 1.62E-12 | 0.71 |
| TP53 | 1.29E-06 | 0.12 |
| STAT3 | 5.39E-02 | 0.12 |
| TNF | 4.15E-08 | 0.33 |
| PTEN | 1.24E-06 | 0.46 |
| CCND1 | 4.31E-09 | 0.0073 |
| IL6 | 6.39E-02 | 0.047 |
| ERBB2 | 3.42E-01 | 0.023 |
| CDH1 | 2.28E-03 | 0.65 |
| ERCC4 | 5.37E-11 | 0.73 |
| ERCC5 | 1.68E-03 | 0.93 |
| AXL | 1.63E-12 | 0.016 |
| MTOR | 4.44E-13 | 0.97 |
| CD44 | <1E-12 | 0.49 |
| CDKN2A | <1E-12 | 0.00038 |
| UGT1A1 | 6.48E-05 | 0.77 |
| HDGF | <1E-12 | 0.72 |
| CXCR2 | 1.86E-07 | 0.36 |
| SLC16A1 | 1.62E-12 | 0.84 |
| PAK1 | 1.62E-12 | 0.83 |
| ABCC2 | 3.61E-05 | 0.77 |
| GDF15 | 4.88E-01 | 0.35 |
| KDM4C | 8.07E-07 | 0.95 |
| BMP4 | 3.68E-01 | 0.81 |
| IGFBP3 | 1.38E-11 | 0.11 |
| JAK2 | 4.07E-02 | 0.18 |
| IGF1R | <1E-12 | 0.99 |
| MET | <1E-12 | 0.54 |
| MUC1 | 6.32E-03 | 0.76 |
| HGF | 1.65E-01 | 0.71 |
| TERT | <1E-12 | 0.015 |
| PIK3CA | <1E-12 | 0.76 |
| EZH2 | <1E-12 | 0.0029 |
| AR | 5.14E-03 | 0.52 |

**S10: Protein expression of hub genes for different tissue of Head and Neck**

| **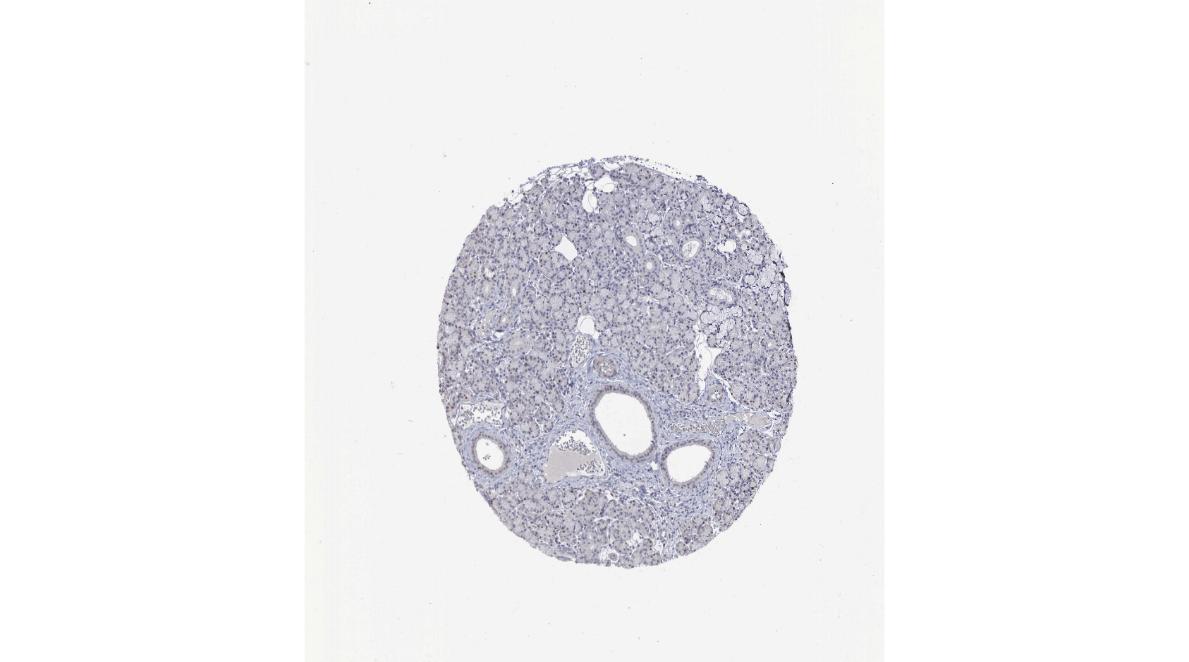** | **Salivary gland**  **CAB000024** Male, age 54 Salivary gland (T-55100) Normal tissue, NOS (M-00100) Patient id: 1682  Glandular cells   | Staining: | **Low** | | --- | --- | |  |  | | Intensity: | **Moderate** | |  |  | | Quantity: | **<25%** | |  |  | | Location: | **Nuclear** | |  |  | | **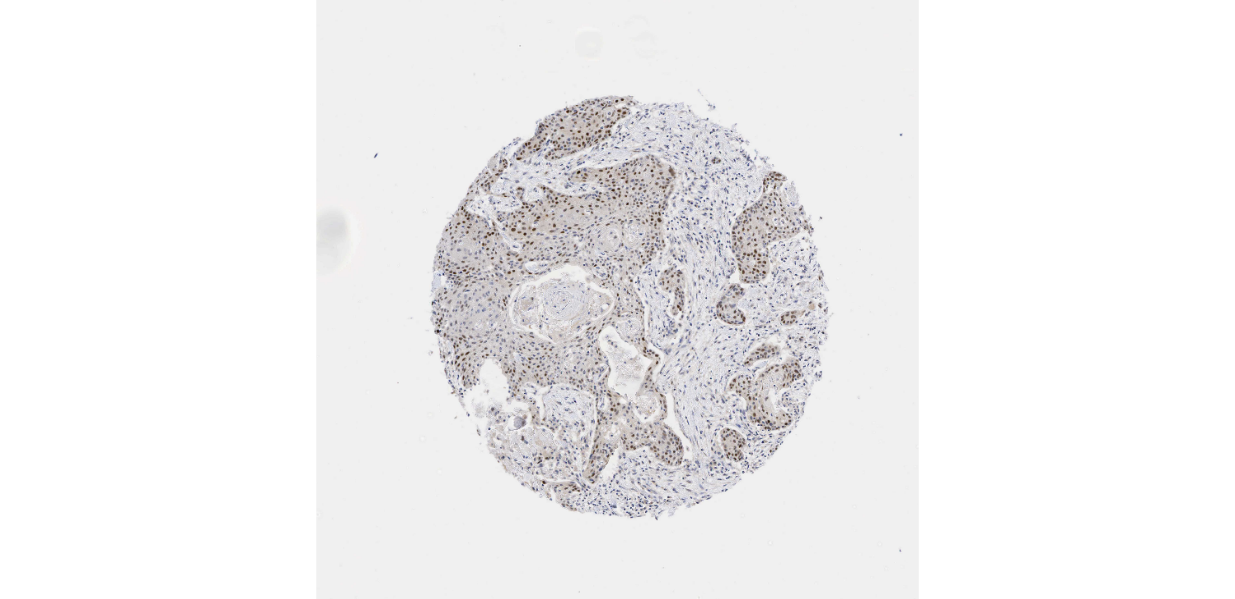** | **Head and neck cancer**  **CAB000024** Female, age 74 Head-Neck (T-Y0000) Salivary gland (T-55100) Lymph node (T-08000) Squamous cell carcinoma, metastatic, NOS (M-80706) Squamous cell carcinoma, NOS (M-80703) Patient id: 807  Tumor cells   | Staining: | **High** | | --- | --- | |  |  | | Intensity: | **Strong** | |  |  | | Quantity: | **75%-25%** | |  |  | | Location: | **Nuclear** | |
| --- | --- | --- | --- | --- | --- | --- | --- | --- | --- | --- | --- | --- | --- | --- | --- | --- | --- | --- | --- | --- | --- | --- | --- | --- | --- | --- | --- | --- | --- | --- | --- | --- | --- |
| **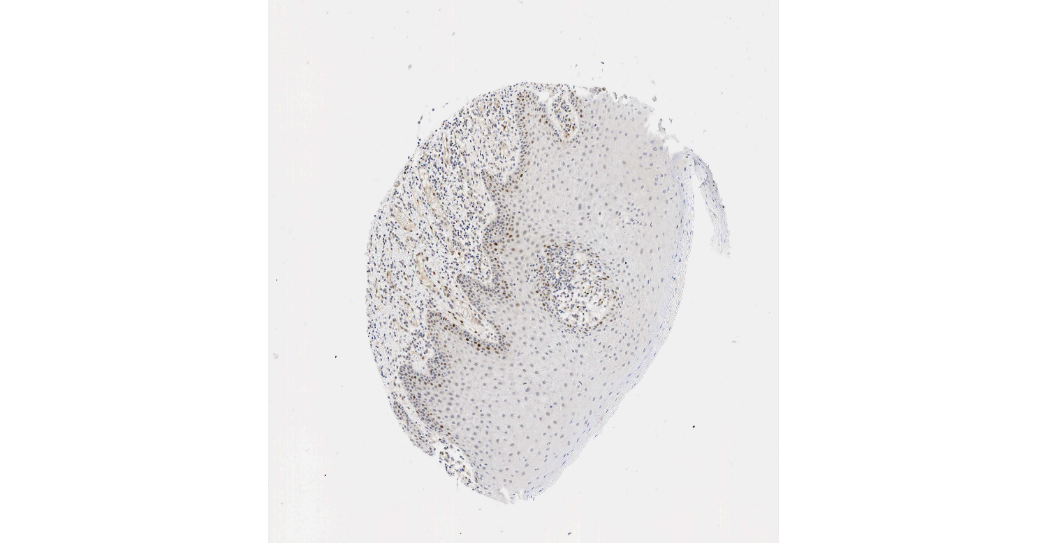** | **Oral mucosa**  **CAB000024** Female, age 56 Oral tissue (T-51000) Normal tissue, NOS (M-00100) Patient id: 201  Squamous epithelial cells   | Staining: | **Low** | | --- | --- | |  |  | | Intensity: | **Moderate** | |  |  | | Quantity: | **<25%** | |  |  | | Location: | **Nuclear** | | **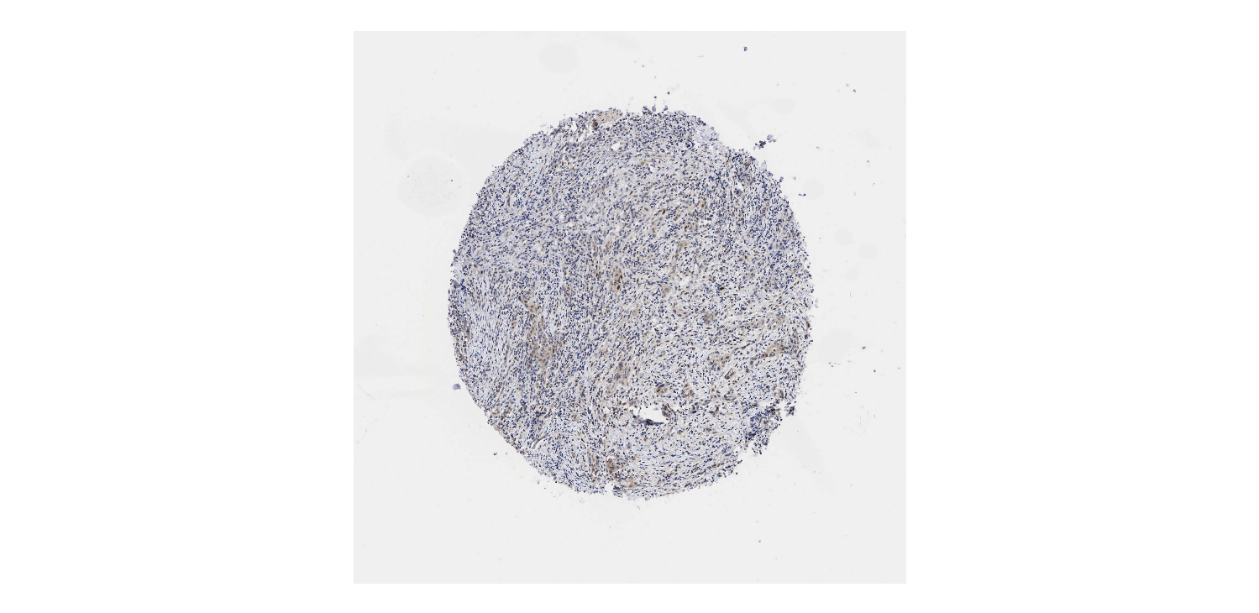** | **Head and neck cancer**  **CAB000024** Female, age 82 Head-Neck (T-Y0000) Oral tissue (T-51000) Squamous cell carcinoma, NOS (M-80703) Patient id: 903  Tumor cells   | Staining: | **Low** | | --- | --- | |  |  | | Intensity: | **Moderate** | |  |  | | Quantity: | **<25%** | |  |  | | Location: | **Nuclear** | |
| **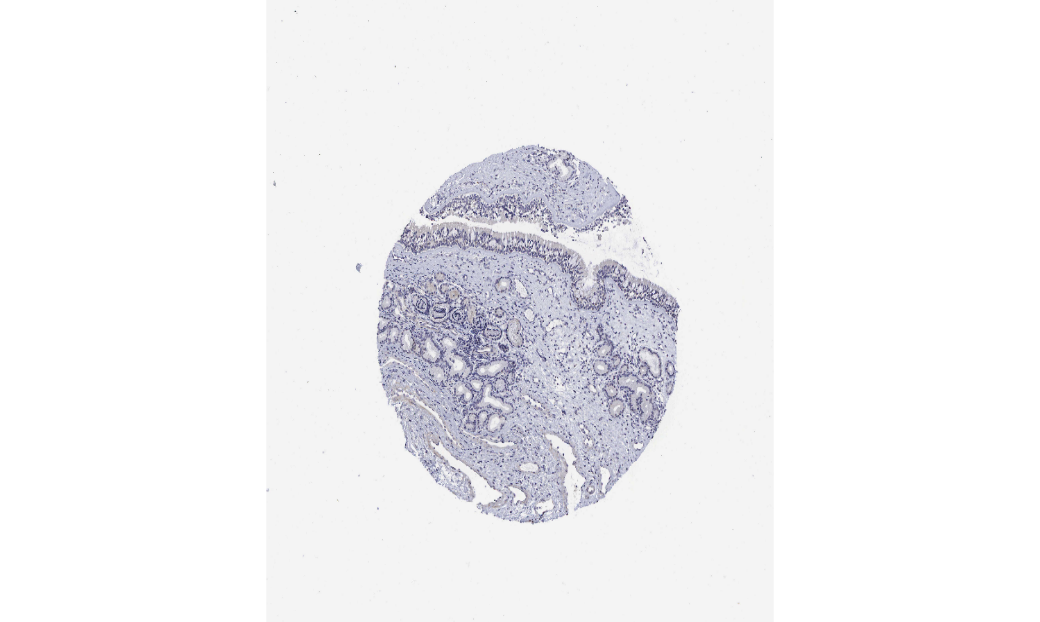** | **Nasopharynx**  **CAB000024** Female, age 78 Nasopharynx (T-23000) Normal tissue, NOS (M-00100) Patient id: 2630  Respiratory epithelial cells   | Staining: | **Low** | | --- | --- | |  |  | | Intensity: | **Moderate** | |  |  | | Quantity: | **<25%** | |  |  | | Location: | **Nuclear** | | **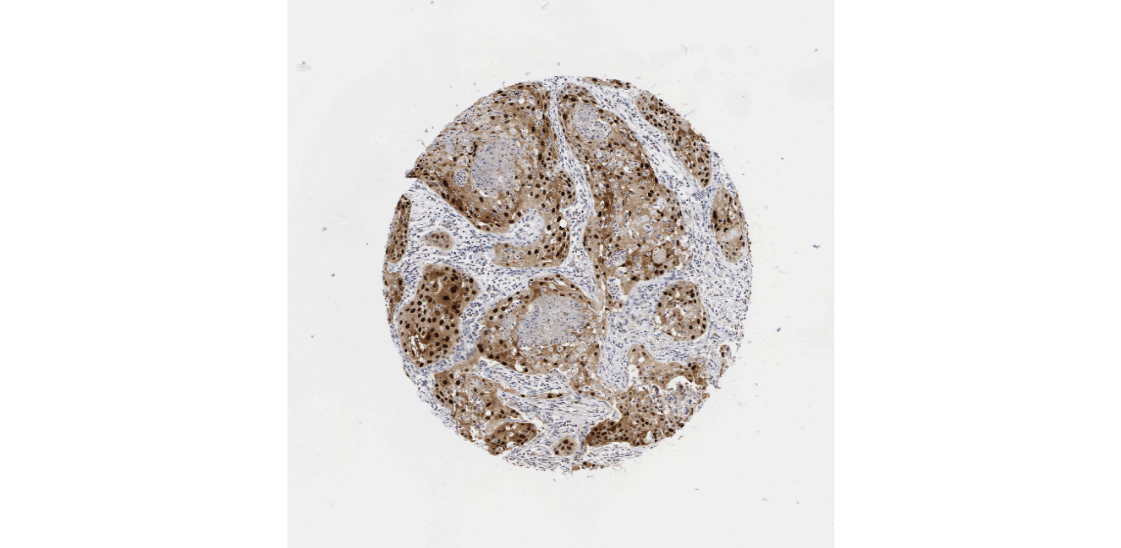** | **Head and neck cancer**  **CAB000024** Male, age 64 Head-Neck (T-Y0000) Squamous cell carcinoma, NOS (M-80703) Patient id: 1130  Tumor cells   | Staining: | **High** | | --- | --- | |  |  | | Intensity: | **Strong** | |  |  | | Quantity: | **>75%** | |  |  | | Location: | **Cytoplasmic/ membranous nuclear** | |
| 1. Immunohistochemical staining of CCND1 | | | |
| **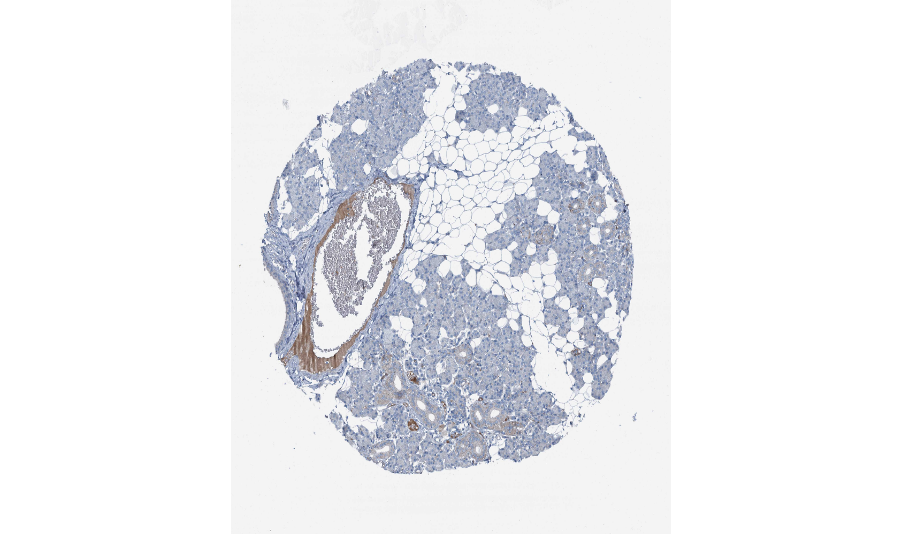** | **Salivary gland**  **HPA037422** Female, age 33 Salivary gland (T-55100) Normal tissue, NOS (M-00100) Patient id: 2048  Glandular cells   | Staining: | **Low** | | --- | --- | |  |  | | Intensity: | **Moderate** | |  |  | | Quantity: | **<25%** | |  |  | | Location: | **Cytoplasmic/ membranous** | | **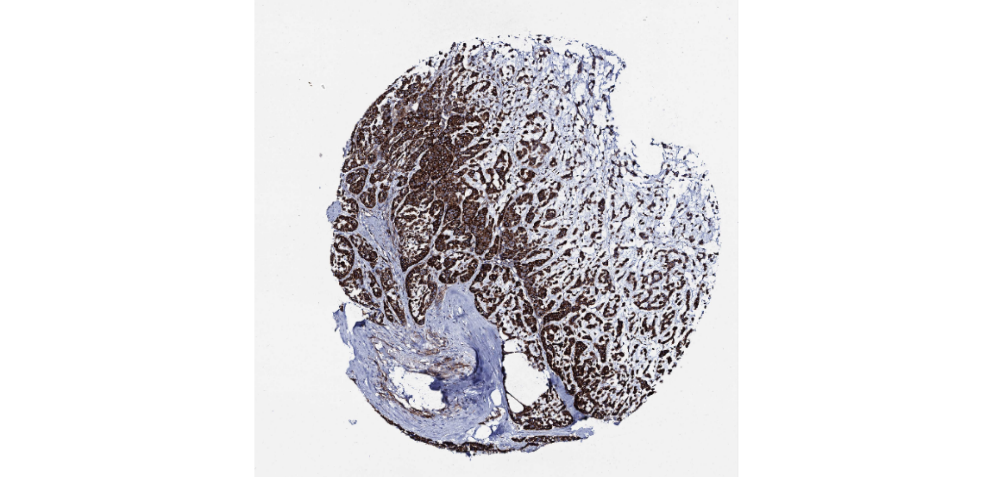** | **Head and neck cancer**  **HPA037423** Female, age 55 Head-Neck (T-Y0000) Adenoma, NOS (M-84000) Adenocarcinoma, NOS (M-81403) Patient id: 797  Tumor cells   | Staining: | **High** | | --- | --- | |  |  | | Intensity: | **Strong** | |  |  | | Quantity: | **>75%** | |  |  | | Location: | **Cytoplasmic/ membranous** | |
| **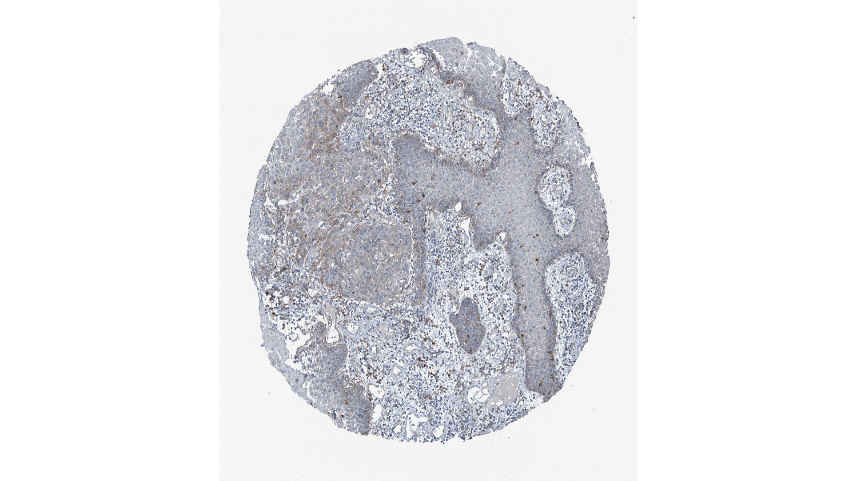** | **Oral mucosa**  **CAB032501** Male, age 54 Skeletal muscle (T-13000) Salivary gland (T-55100) Peripheral nerve tissue (T-X0500) Oral tissue (T-51000) Normal tissue, NOS (M-00100) Patient id: 1682  Squamous epithelial cells   | Staining: | **Low** | | --- | --- | |  |  | | Intensity: | **Moderate** | |  |  | | Quantity: | **<25%** | |  |  | | Location: | **Cytoplasmic/ membranous** | | **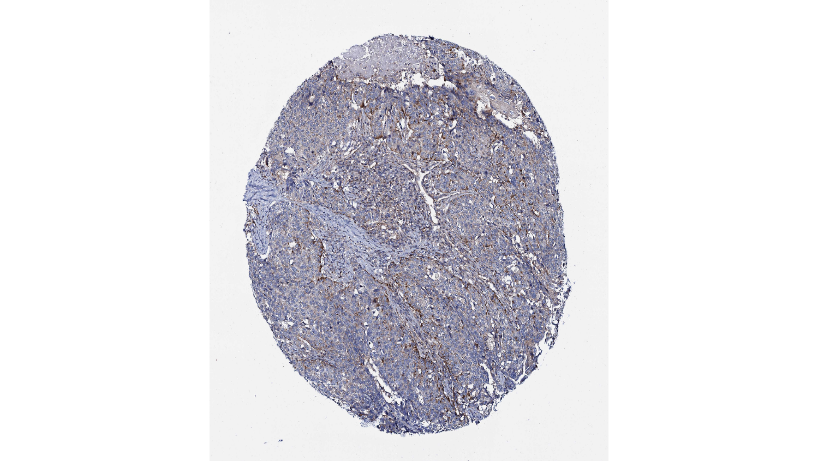** | **Head and neck cancer**  **HPA037423** Male, age 66 Head-Neck (T-Y0000) Squamous cell carcinoma, NOS (M-80703) Patient id: 2547  Tumor cells   | Staining: | **Low** | | --- | --- | |  |  | | Intensity: | **Moderate** | |  |  | | Quantity: | **<25%** | |  |  | | Location: | **Cytoplasmic/ membranous** | |
| **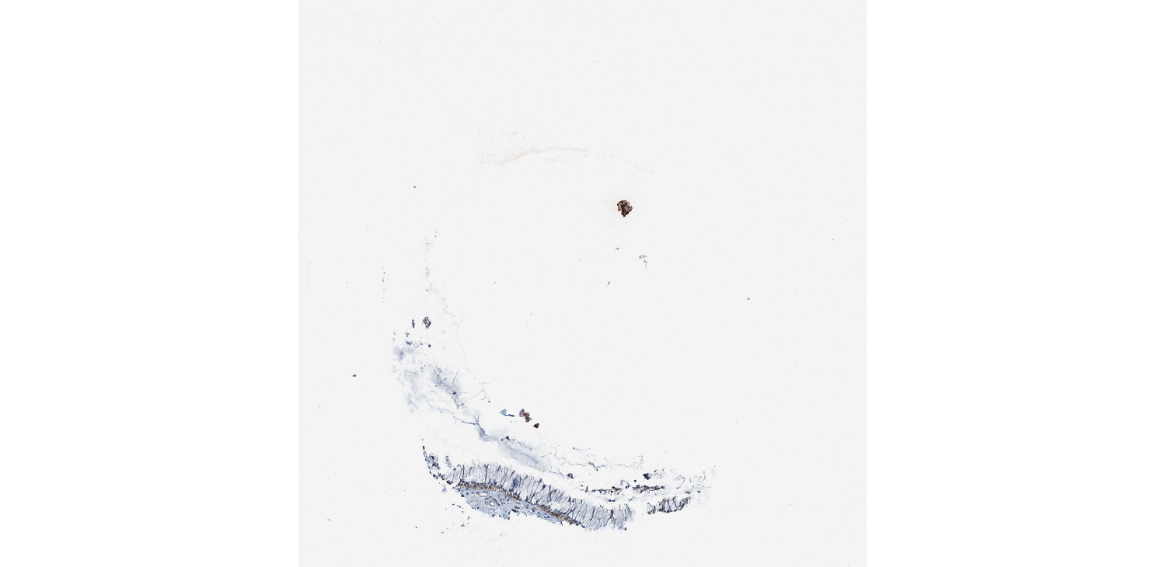** | **Nasopharynx**  **CAB032501** Male, age 29 Nasopharynx (T-23000) Inflammation, NOS (M-40000) Normal tissue, NOS (M-00100) Patient id: 2413  Respiratory epithelial cells   | Staining: | **Low** | | --- | --- | |  |  | | Intensity: | **Weak** | |  |  | | Quantity: | **>75%** | |  |  | | Location: | **Cytoplasmic/ membranous** | | **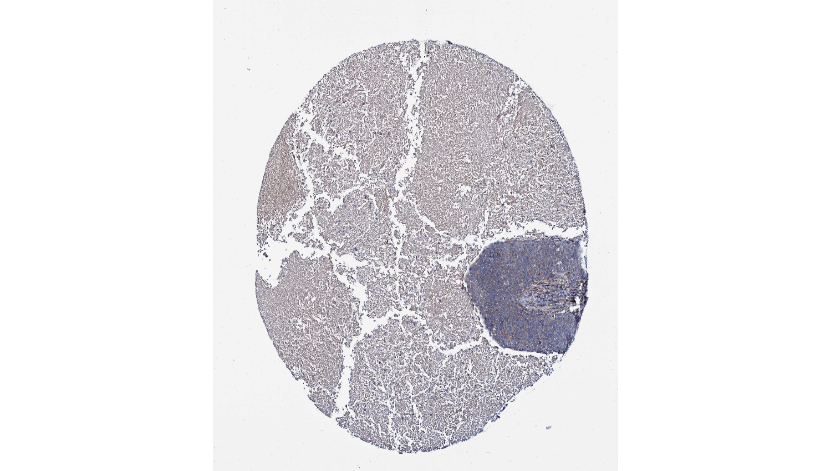** | **Head and neck cancer**  **HPA037423** Male, age 62 Head-Neck (T-Y0000) Lymph node (T-08000) Squamous cell carcinoma, metastatic, NOS (M-80706) Squamous cell carcinoma, NOS (M-80703) Patient id: 1743  Tumor cells   | Staining: | **Low** | | --- | --- | |  |  | | Intensity: | **Weak** | |  |  | | Quantity: | **>75%** | |  |  | | Location: | **Cytoplasmic/ membranous** | |
| 1. Immunohistochemical staining of AXL | | | |
| **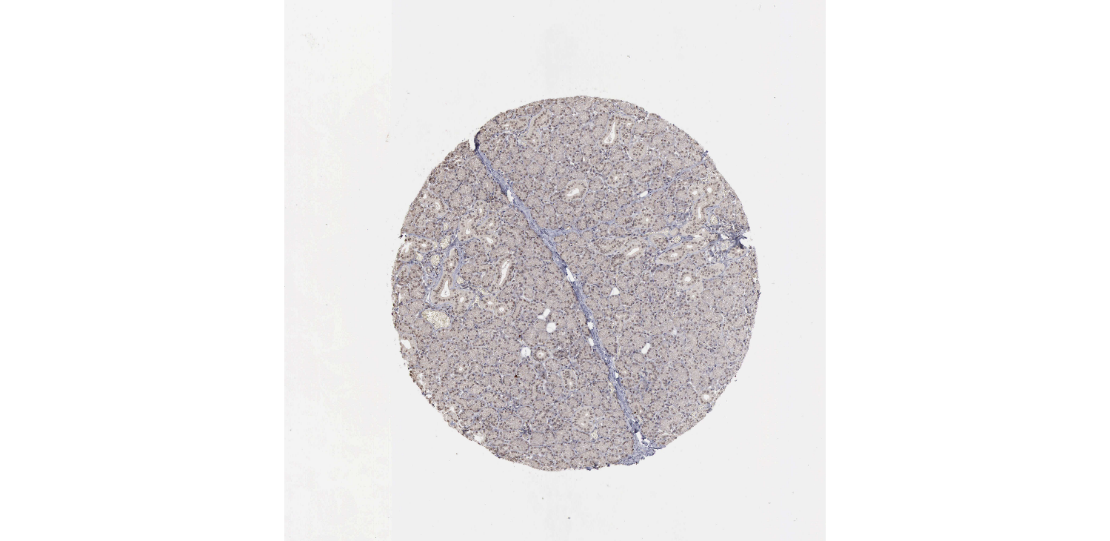** | **Salivary gland**  **CAB000445** Female, age 24 Salivary gland (T-55100) Normal tissue, NOS (M-00100) Patient id: 2323  Glandular cells   | Staining: | **Low** | | --- | --- | |  |  | | Intensity: | **Weak** | |  |  | | Quantity: | **>75%** | |  |  | | Location: | **Cytoplasmic/ membranous nuclear** | |  |  | | **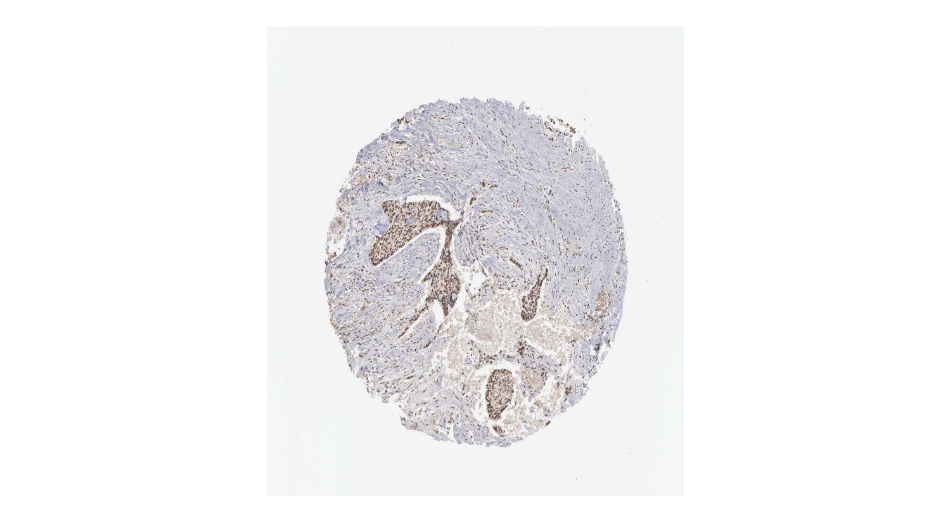** | **Head and neck cancer**  **CAB000445** Male, age 70 Head-Neck (T-Y0000) Salivary gland (T-55100) Squamous cell carcinoma, NOS (M-80703) Patient id: 1060  Tumor cells   | Staining: | **Medium** | | --- | --- | |  |  | | Intensity: | **Moderate** | |  |  | | Quantity: | **>75%** | |  |  | | Location: | **Nuclear** | |
| **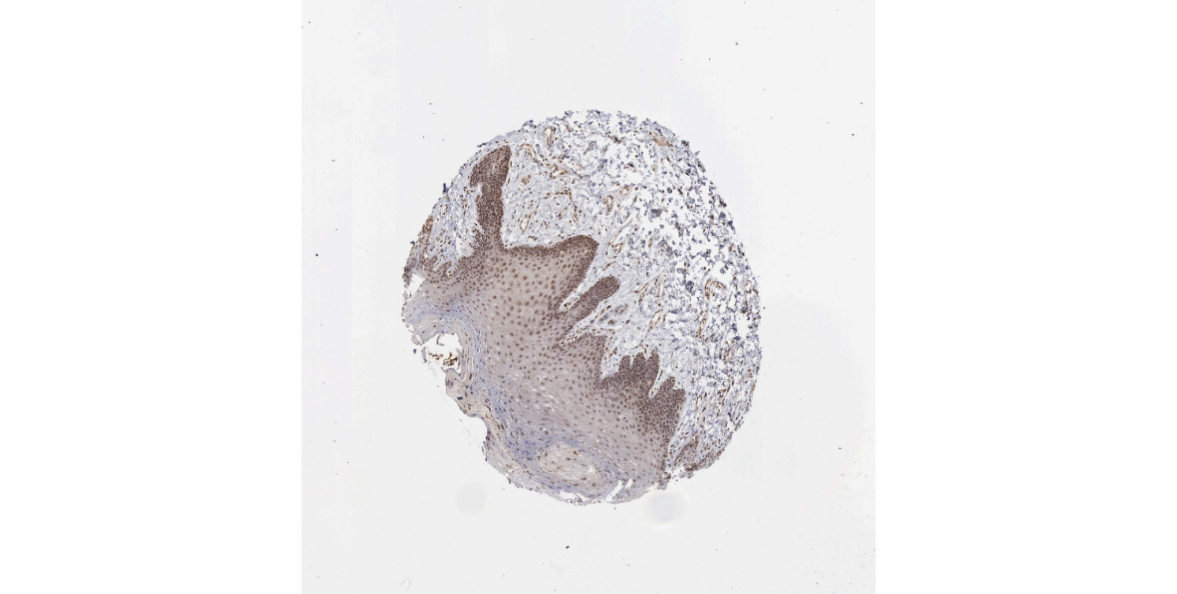** | **Oral mucosa**  **CAB000445** Male, age 62 Oral tissue (T-51000) Normal tissue, NOS (M-00100) Patient id: 1505  Squamous epithelial cells   | Staining: | **Medium** | | --- | --- | |  |  | | Intensity: | **Moderate** | |  |  | | Quantity: | **>75%** | |  |  | | Location: | **Cytoplasmic/ membranous nuclear** | | **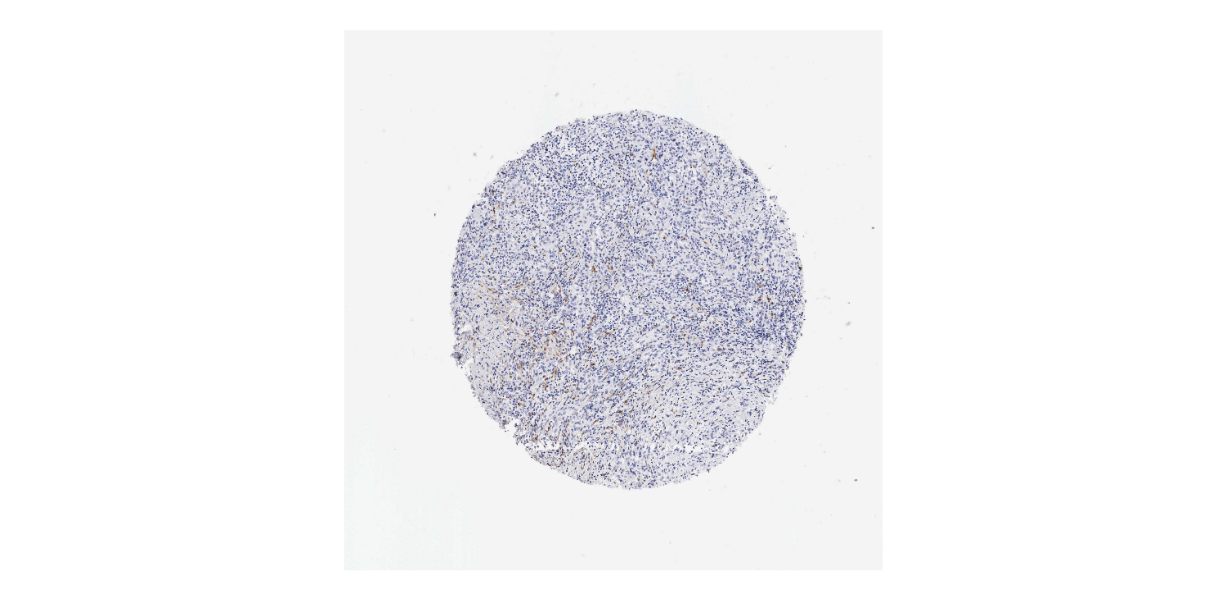** | **Head and neck cancer**  **CAB000093** Female, age 82 Head-Neck (T-Y0000) Oral tissue (T-51000) Squamous cell carcinoma, NOS (M-80703) Patient id: 903  Tumor cells   | Staining: | **Low** | | --- | --- | |  |  | | Intensity: | **Moderate** | |  |  | | Quantity: | **<25%** | |  |  | | Location: | **Cytoplasmic/ membranous** | |
| **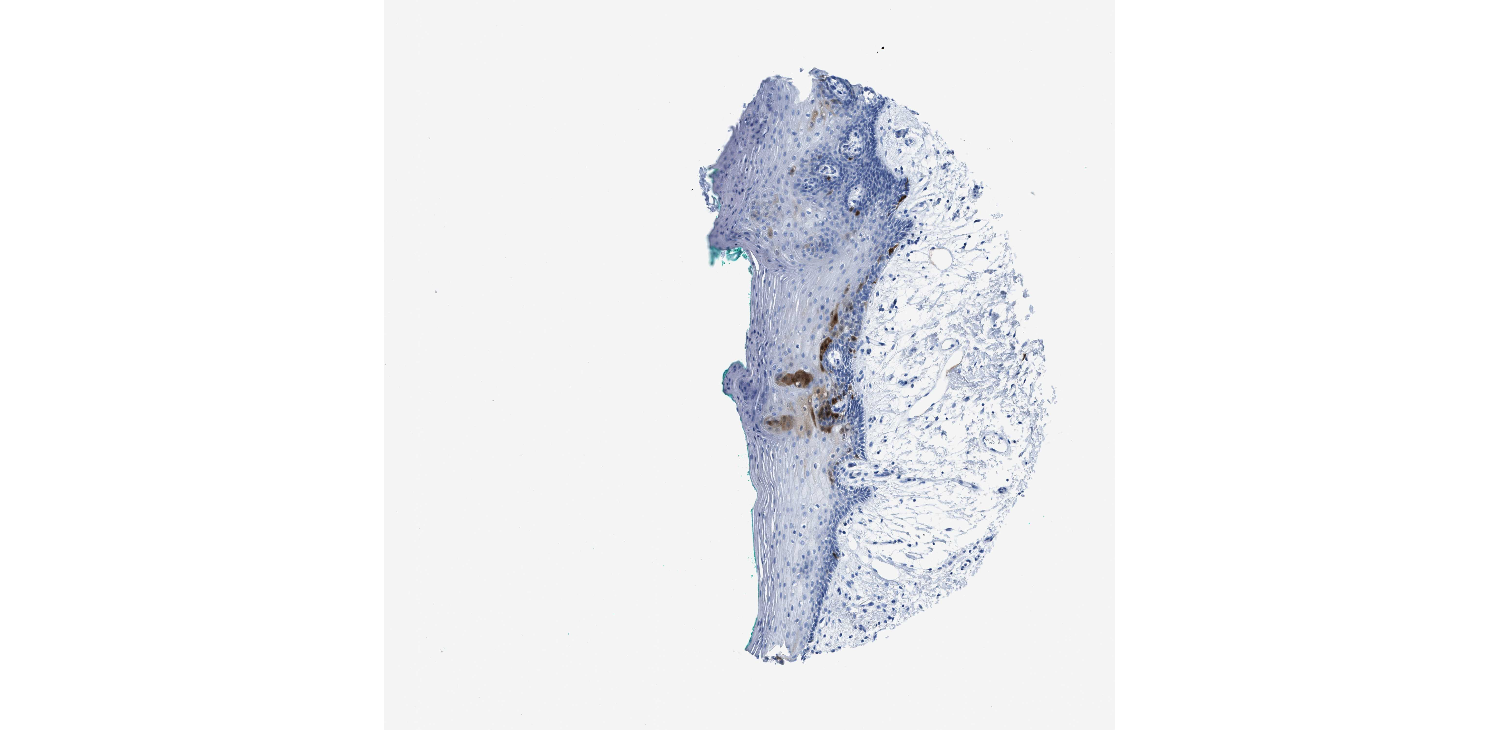** | **Oral mucosa**  **CAB018232** Female, age 84 Skeletal muscle (T-13000) Peripheral nerve tissue (T-X0500) Oral tissue (T-51000) Normal tissue, NOS (M-00100) Patient id: 2406  Squamous epithelial cells   | Staining: | **Low** | | --- | --- | |  |  | | Intensity: | **Moderate** | |  |  | | Quantity: | **<25%** | |  |  | | Location: | **Cytoplasmic/ membranous** | | **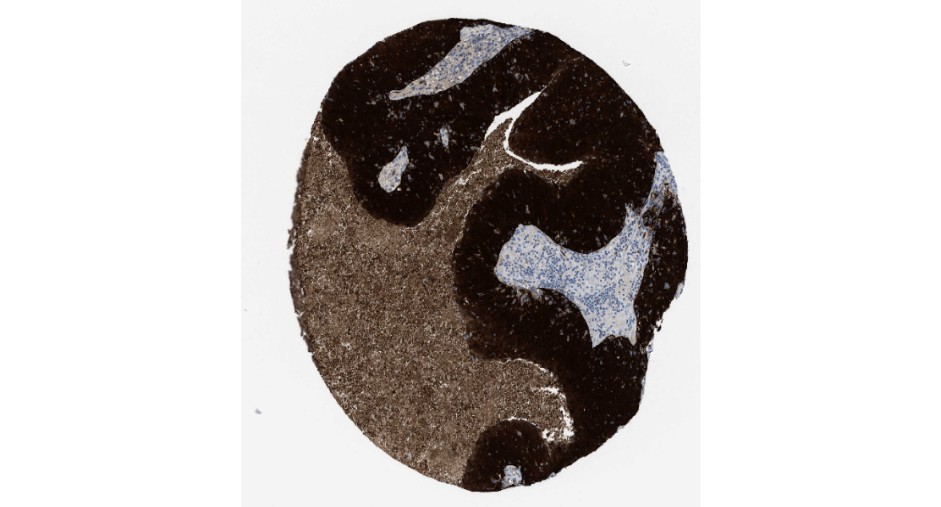** | **Head and neck cancer**  **CAB018232** Male, age 62 Head-Neck (T-Y0000) Lymph node (T-08000) Squamous cell carcinoma, metastatic, NOS (M-80706) Squamous cell carcinoma, NOS (M-80703) Patient id: 1743  Tumor cells   | Staining: | **High** | | --- | --- | |  |  | | Intensity: | **Strong** | |  |  | | Quantity: | **>75%** | |  |  | | Location: | **Cytoplasmic/ membranous nuclear** | |
| 1. Immunohistochemical staining of CDKN2A | | | |
| **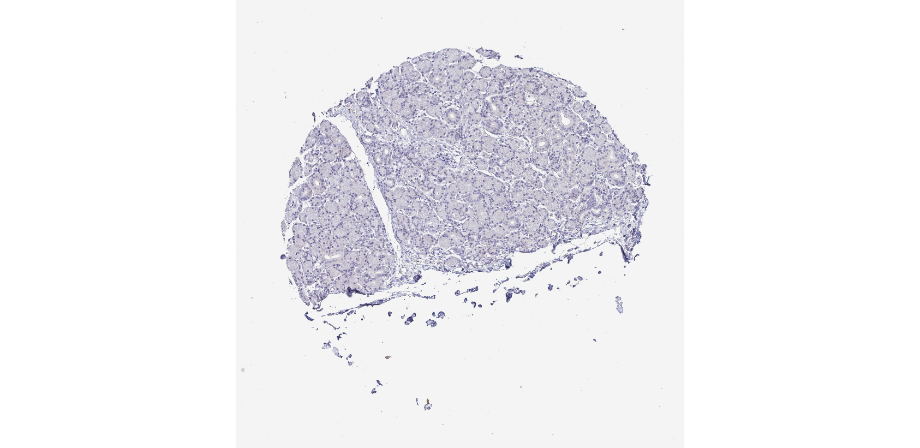** | **Salivary gland**  **HPA054641** Female, age 24 Salivary gland (T-55100) Normal tissue, NOS (M-00100) Patient id: 2323  Glandular cells   | Staining: | **Not detected** | | --- | --- | |  |  | | Intensity: | **Negative** | |  |  | | Quantity: | **None** | | **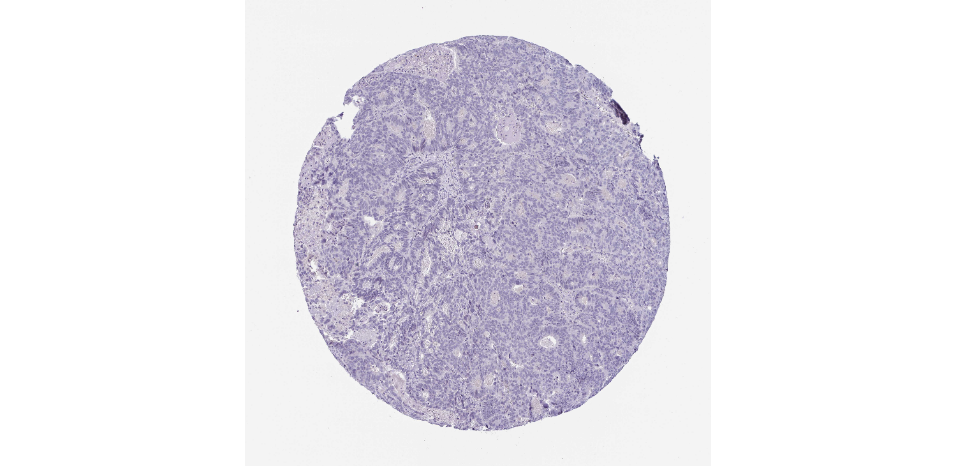** | **Head and neck cancer**  **HPA054641** Male, age 62 Head-Neck (T-Y0000) Adenocarcinoma, NOS (M-81403) Patient id: 4515  Tumor cells   | Staining: | **Not detected** | | --- | --- | |  |  | | Intensity: | **Negative** | |  |  | | Quantity: | **None** | |
| **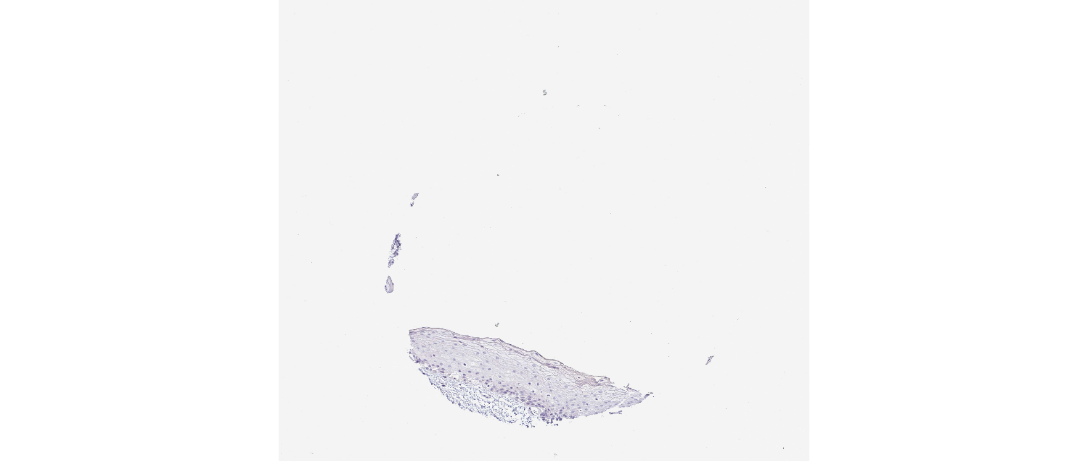** | **Oral mucosa**  **HPA054641** Female, age 82 Oral tissue (T-51000) Normal tissue, NOS (M-00100) Patient id: 4417  Squamous epithelial cells   | Staining: | **Not detected** | | --- | --- | |  |  | | Intensity: | **Negative** | |  |  | | Quantity: | **None** | | **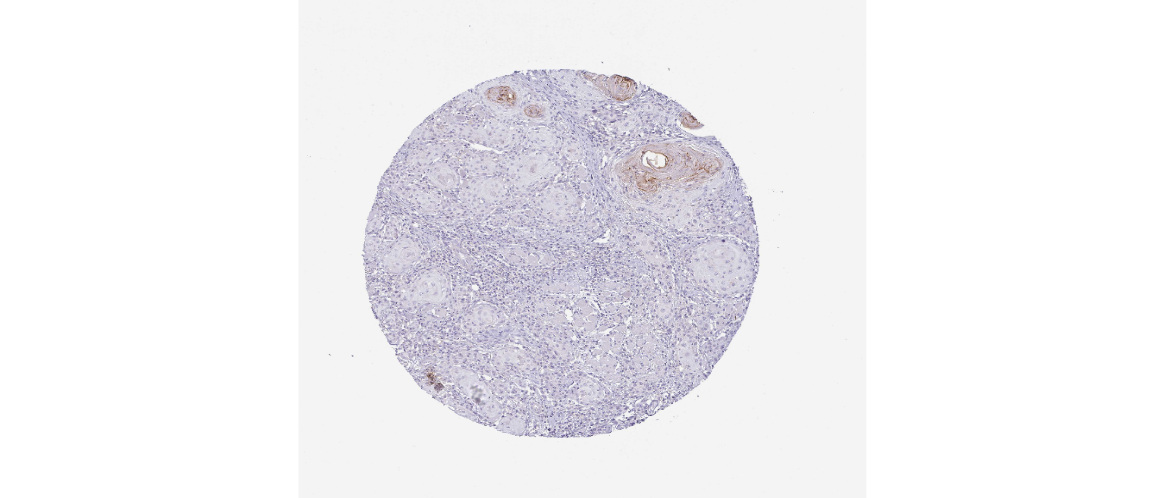** | **Head and neck cancer**  **HPA054641** Female, age 80 Head-Neck (T-Y0000) Squamous cell carcinoma, NOS (M-80703) Patient id: 3722  Tumor cells   | Staining: | **Not detected** | | --- | --- | |  |  | | Intensity: | **Negative** | |  |  | | Quantity: | **None** | |
[truncated: 2,771 more chars]
